# A social media intervention to improve nutrition knowledge and behaviors of low income, pregnant adolescents and adult women

**DOI:** 10.1371/journal.pone.0223120

**Published:** 2019-10-24

**Authors:** Kiley B. Vander Wyst, Megan E. Vercelli, Kimberly O. O’Brien, Elizabeth M. Cooper, Eva K. Pressman, Corrie M. Whisner

**Affiliations:** 1 College of Health Solutions, Arizona State University, Phoenix, Arizona, United States of America; 2 Division of Nutritional Sciences, Cornell University, Ithaca, New York, United States of America; 3 School of Medicine and Dentistry, University of Rochester, Rochester, New York, United States of America; 4 University of Rochester Medical Center Midwifery Group, Rochester, New York, United States of America; University of Leeds, UNITED KINGDOM

## Abstract

Pregnant adolescents are at increased risk of adverse pregnancy outcomes compared to adult women, necessitating a need for early and comprehensive health care. This study aimed to evaluate the effectiveness of a social media intervention (i.e. weekly prenatal health messages) on improving diet quality, and health beliefs and knowledge. Participants (10 adolescents and 12 adults) completed pre-post intervention interviews, nutrition knowledge and health belief questionnaires, and 24-hour diet recalls. Participants entering pregnancy as overweight or obese were more likely to experience excessive GWG during the intervention. The adults had greater participation during the study despite high levels of social media access among both groups. Participants were able to identify sugar-sweetened foods and acknowledged the benefits of whole grains; however, overall knowledge of MyPlate Guidelines was limited. Social media-based education was well received by participants but did not result in large changes in dietary intake and knowledge. Although larger studies are needed, social media appears to have the potential to reach high-risk women.

## Introduction

Excessive gestational weight gain (EGWG), is more prevalent among pregnant adolescents than pregnant adults [1,2]. EGWG contributes to negative outcomes such as preterm birth[3], gestational diabetes [4,5], maternal hypertensive disorders [3,4], cesarean section [4,6], macrosomia [4,6,7], and postpartum weight retention [8] in both populations [9]. Low-income, African-American adolescents [1] and women with less education [10] are at greater risk for EGWG and consume lower-quality diets [11]. Furthermore, it has been shown that growing adolescents gain more gestational weight compared to non-growing adolescents and adults [11]. Motivating adolescents to maintain healthy lifestyle habits is a challenge, especially among low-income teens who often lack access to high-quality prenatal care and healthful foods [12].

The majority of studies that have focused on improving dietary intake have been in low-income, adult women. These studies primarily used in-home counseling sessions and websites to improve gestational weight gain and postpartum weight loss [13,14]. Other studies using educational newsletters to encourage appropriate weight gain showed that adult women who participated more actively in the intervention gained a more appropriate amount of gestational weight [15]. Research among low-income, pregnant adolescents has utilized in-person counseling and website-based education [16,17]; however, interventions remain sparse and results are not consistent [16]. Using new technologies such as text messaging and websites have proven helpful for promoting healthy behaviors among non-pregnant teens [18] but these methods have not been widely used to improve the dietary intake and gestational weight gain of pregnant adolescents.

Most adolescents, including those from lower socioeconomic groups, have greater access to social media and text messaging programs [12,19,20], suggesting these are viable methods of targeting this hard to reach population. The purpose of this pilot study was to investigate dietary changes and to examine the nutrition knowledge and behaviors of low-income, pregnant, adolescents in comparison to adult women, residing in Rochester, New York after receiving prenatal nutrition, fitness and health information via social media. We hypothesized that (1) dietary intake would improve (lower fat and sugar and greater fiber and micronutrient intakes) after receiving the social media intervention, (2) pregnant adults would have better overall dietary intake compared to pregnant adolescents, (3) sugar intake would positively correlate with pre-pregnancy BMI and excessive gestational weight gain, and (4) attitudes and beliefs about the impacts of prenatal nutrition and fitness on fetal growth would improve across the intervention for both groups.

## Materials and methods

### Study design and subject recruitment

**Fig 1:**
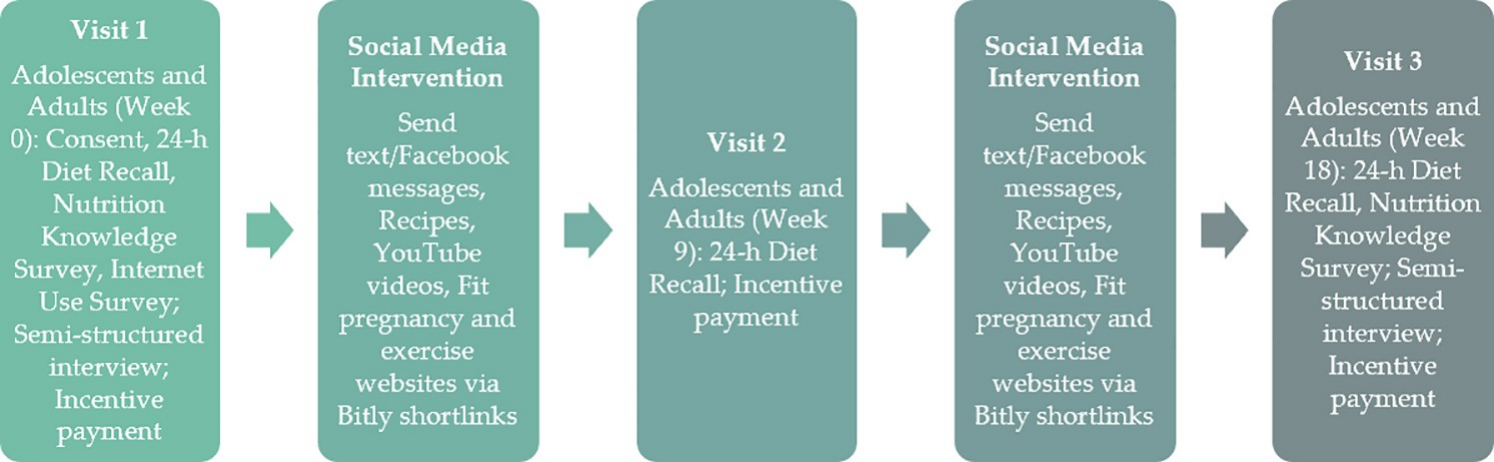
Flowchart of study design for an 18-week social media intervention among pregnant adolescents and adult women.

This pilot study was an 18-week longitudinal social media intervention addressing nutrition knowledge and behaviors of low-income, pregnant adolescents and adult women (**Fig 1**). The conceptual model used to design the intervention was Social Cognitive Theory.

The intervention included health information (pregnancy fitness, healthy recipes, nutrition, pregnancy fun facts, and stress management) sent out via Facebook (6 messages/week) and/or cellular text message (SMS; 6 messages/week). **S1 Table** and **S1 Fig.** provides the messaging content used for both text and Facebook messages. Messaging platforms were selected based on previous findings from adult women [21] and previous research demonstrating that pregnant adolescents prefer to receive prenatal nutrition and health information via digital media platforms [22]. For those without social media access (no participants reported a lack of social media access), a weekly paper mailing option was offered to assure that all participants could benefit from the intervention (n = 1 adult and n = 1 teen asked to receive mailings in addition to social media messages because they wanted to have physical copies of the messages). All messages were written at ≤ 6th grade reading level. Participants began receiving messages at ≤ 28 weeks of gestation.

Low income, pregnant adolescents aged 14–18 years of age and low-to-medium income pregnant adults aged 19+ were recruited from the same clinic in Rochester, NY. All adolescent participants were recruited from the Rochester Adolescent Maternity Program (RAMP) at a Rochester, NY inner-city prenatal clinic, from a cohort of teens already participating in a larger study designed to assess the dose-response effects of vitamin D supplementation on inflammation and infection during pregnancy. The adult women were not participants in the vitamin D study but were recruited from the clinic by flyers. The sample size for this study was based on recruiting until information/message redundancy was achieved. As described previously [23], redundancy was expected to be reached before the enrollment of 40 participants. In total, 12 adolescents and 12 adults were enrolled and sufficient saturation was reached.

All study data were collected at the clinic or via Skype with participants who were unable to meet in person. Inclusion criteria included pregnant women carrying a single fetus who were between 12–28 weeks of gestation (as specified in the parent vitamin D study) [24]. Exclusion criteria for the study included having a history of malabsorptive diseases, eating disorders, HIV infection, diabetes, high blood pressure, or current cigarette use. The Cornell University and Arizona State University Institutional Review Boards approved the collection and analyses of data. All adult participants provided written informed consent. In the state of New York, pregnant adolescents (<18 years) are treated as emancipated minors and are therefore, permitted to provide informed consent without a parent/guardian, pending IRB approval. All adolescents ≥15 years signed the same consent form as the adults.

### Measures

Maternal demographic and anthropometric data included race, ethnicity, height (cm), and weight (kg). Maternal BMI was calculated in kg/m^2^. Neonatal length (cm) and weight (g), as well as participation in the Special Supplemental Nutrition Program for Women, Infants and Children (WIC), were obtained from medical charts by the study coordinator. Maternal gestational weight gain (GWG) was calculated by subtracting the self-reported pre-pregnancy weight, collected at entry into the study, from the hospital delivery weight. The 2009 Institute of Medicine (IOM) gestational weight gain guidelines, based on pre-pregnancy BMI, were used to assess the adequacy of weight gain during pregnancy. These guidelines have been recognized as appropriate for use with both pregnant adults and adolescents [23,25].

All participants completed pre- and post-intervention nutrition knowledge questionnaires (S1 File), one electronic data usage survey (S2 File) and up to three 24-h dietary recalls across pregnancy. Data were collected at or near weeks 0, 9 and 18 of the intervention. Passive and active participation on the private Facebook group page were measured by tracking the number of messages viewed (passive) and “liked” (active) by participants. Additionally, active participation was assessed by sharing periodic poll questions via Facebook or text message and recording individual responses. Bitly (https://bitly.com) shortlinks to health websites and videos were included in SMS and Facebook messages sent to all participants. Study-wide shortlink use was summarized as number of clicks (active participation) on each link within the Bitly built-in analytics system. We complied with the terms of service for both Facebook and Bitly shortlink websites. Messages and web links sent via Facebook and SMS were categorized into ten themes; exercise, recipes, healthy diet, weight gain, pregnancy cravings, relaxation, hydration, fruits and vegetables, fun, and science.

A survey was used to evaluate and assess participant knowledge of basic nutrition concepts as described by the MyPlate guidelines [26] and 2010 Dietary Guidelines for Americans [27]. The survey consisted of 15 multiple-choice questions and was given to participants at the first and last study visits. This survey was developed specifically for this pilot intervention and was modeled after a previously validated nutrition knowledge survey [28]; however, it was not previously validated in this specific population. The survey aimed to assess nutritional behaviors, nutritional knowledge, and general health knowledge. Specific questions were also included to assess knowledge of dietary fiber and added sugar-containing foods.

Three 24-hour diet recalls were administered to participants by the study coordinator and/or study staff at clinic visits closest to 0, 9 and 18 weeks of the social media intervention. The mean gestational age of participants at these three time points was 23.2±5.1, 31.4±4.7, and 36.0±3.7 weeks, respectively. Each participant described their dietary intake during the previous day and study staff probed for easily forgotten foods such as beverages and condiments using the multi-pass method. Questions regarding whether reported intakes were characteristic of a typical day and experience of pica behaviors were also included at the end of the diet recall. The program Food Processor (ESHA Research. Food Pro Version 10.14.0. Salem, OR)[29] was used to calculate mean daily habitual macronutrient and micronutrient composition.

Two in-depth formative interviews (S3 File) were conducted with each participant, one at baseline and the other after receiving the social media intervention. The primary purposes of the baseline interviews were to characterize maternal attitudes and beliefs about prenatal health and to perform a brief needs assessment for the development of Facebook message and SMS content. The goals of the follow-up interview were to evaluate changes in attitudes and beliefs, as well as to gain feedback on the intervention (i.e. message topic, tone, frequency, and mode of delivery). Interview topics at both time points included beliefs and behaviors related to weight gain and fetal development, prenatal diet, physical activity, and stress management. Additionally, participants were asked to describe their social support and how this impacted health behaviors using Social Cognitive Theory as a guide for question development.

One researcher performed all participant interviews which were audio-recorded. Digital transcriptions of each audio-recording were completed by a single person and confirmed by a second to ensure accuracy of data. The same researcher who initially transcribed the recordings also undertook the initial identification of themes and corresponding coding. All coding was done by hand using color-coding in digital documents and a spreadsheet key. Theme identification and coding were reviewed and corroborated by a second researcher.

### Statistical analysis

Descriptive statistics were computed for all variables. Data were checked for outliers (± 3 SD’s from the mean) and missing values in order to meet assumptions of the statistical tests. No outliers were identified. Macro- and micronutrient intakes were adjusted to the mean caloric intake according to the residual method [30]. This was done in order to adjust for variation in individual diets and reduce the chance of bias due to differences in total caloric consumption between people. Adjusted nutrient intakes were used in all subsequent analyses. Dietary changes for all variables were computed by subtracting the pre- intervention dietary values from the respective post-intervention dietary values. Data were tested for normality using the Shapiro-Wilk test and data that were not normally distributed were assessed using non-parametric tests. Data are presented as mean ± SD or median (IQR) depending on the normality of each variable.

A two-way repeated measures general linear model was used to assess dietary change over time with a time by group interaction term and time and group (adolescents, adults) as fixed variables. One adolescent had an underweight pre-pregnancy BMI and her data were combined with the normal weight group after confirming that findings did not differ as a result of BMI grouping. Additionally, a univariate general linear model was run to compare the change in sugar intake by gestational weight gain categories (within recommended limits or excessive gestational weight gain) while controlling for race, pre-pregnancy BMI, and WIC participation.

Nutrition knowledge and behavior survey responses were summarized using frequencies and percentages. Pre- and post-intervention percentages for both adolescents and adults were compared within and between subjects using Pearson Chi-square. Statistical significance was fixed at P<0.05. The system used for all analyses was SPSS statistical software (SPSS, Version 25.0. Armonk, NY: IBM Corp.) [31].

## Results

### Descriptive characteristics

Twenty-four women were recruited for this study (12 adolescents and 12 adults) in July 2013 and followed through July 2014. All 12 adults finished the study; however, two adolescents were lost to follow-up due to delivering at hospitals not affiliated with the University of Rochester or RAMP. Of the 22 participants included in the final analysis, seven adolescents and three adults were enrolled in WIC. Seven of the adolescents were Black, two were Hispanic White, and one was Non-Hispanic White. Of the adult participants, four were Black, six were Non-Hispanic White, and one was Hispanic White.

Maternal and neonatal characteristics are found in **Table 1**. Upon comparison of maternal and delivery characteristics, age prior to intervention was the only statistically significant differences between the adults and adolescents. Based on the IOM weight gain guidelines, 40% (three of the four obese teens and one of the two overweight teens) of adolescents gained excessive gestational weight.

**Table 1:** Subject characteristics of pregnant adolescents and adult women participating in an 18-week prenatal health promotion social media intervention.

Mean	Adolescents (N = 10)	Adults (N = 12)	P-value
**Pre-Intervention Age (y)**^**2**^	16.97 (16.40, 17.73)	29.20 (23.71, 33.75)	**<0.001**
**Pre-Pregnancy BMI (kg/m**^**2**^**)**^**1**^^,^^**3**^	26.97±6.53	26.71±4.84	0.915
**Delivery BMI (kg/m**^**2**^**)**^**1**^	31.33±6.17	31.53±4.49	0.930
**GWG (kg)**^**2**^	11.14 (7.84, 15.18)	12.5 (11.14, 17.16)	0.197
**GA at Delivery (wks)**^**1**^	40.24±0.88	39.89±1.04	0.409
**Infant Birth Weight (kg)**^**1**^	3.42±0.44	3.37±0.39	0.773
**Infant Birth Length (cm)**^**1**^	51.23±2.61	50.86±2.51	0.740

^1^Ind. T-Test: Mean ± SD

^2^Mann-Whitney: Median (25%, 75%)

^3^Pre-pregnancy BMI was self-reported

GWG, gestational weight gain; GA, gestational age; Bolded text denotes significant p-value.

Two teens with a normal pre-pregnancy BMI gained less than the recommended amount of weight while three (30%) gained within recommendations. Nearly 67% (n = 8; 3 obese, 2 overweight, and 3 normal-weight) of adult women gained excessive weight during gestation. None of the adult participants gained less than the recommended amount of weight. There was no significant difference in the GWG category (excessive, within, or under) between adolescents and adults (Χ^2^(2) = 3.178, p = 0.204).

### Dietary changes

Over the 18-week intervention mean caloric consumption did not change significantly in adolescents (pre: 2218.00±906.59 kcal/day, post: 2595.10±1128.88 kcal/day, P = 0.263) or adults (pre: 2336.75±641.52 kcal/day, post: 2323.67±566.98 kcal/day, P = 0.958). For both adolescents and adults, reported intakes for protein (10–35% of kcals) and carbohydrates (45–65% of kcals) were within the acceptable macronutrient distribution ranges (AMDRs) at both pre- and post-intervention time points. For both adolescents and adults, the pre-intervention fat intake was higher than the AMDR (20–35% of kcals), with a reported average of 39.7% for adolescents and 37.6% for adults. Fat intake at the end of the intervention dropped for both adolescents and adult women; however, it remained slightly above the AMDR for adult women. **Table 2** shows the macronutrient composition for both adolescents and adult women pre- and post-intervention. **Table 3** depicts group, time and time*group interaction effects for dietary intake variables. Carbohydrate intake significantly increased over the 18-week intervention period [F(1,20) = 5202, p<0.001]. Micronutrients that increased significantly over the intervention period were calcium [F(1,20) = 17780, p<0.001], magnesium [F(1,16) = 34.8, p<0.001), and iron [F(1,20) = 7.394, p = 0.013], while folate intake significantly [F(1,20) = 3619, p<0.001) decreased. A significant interaction was observed between time and group for sugar consumption [F(1,20) = 7.61, p = 0.012]. While sugar consumption increased for both the adolescent and adult groups, the increase was slightly greater for the adolescents (7.4±0.2 vs. 6.3±0.1 g/d, P = 0.005, respectively).

**Table 2:** Pre- and Post-Intervention Macronutrient Distributions of Pregnant Adolescents and Adult Women Participating in an 18-Week Prenatal Health Promotion Social Media Intervention.

	Pregnant Adolescents (N = 10)		Pregnant Adults (N = 12)		AMDR Range
Macronutrient	Pre-Intervention Mean	Post-Intervention Mean	Pre-Intervention Mean	Post-Intervention Mean	
Protein (%)	15.3±1.34	13.8±1.27	14.4±1.08	15.6±0.92	10–35%
Fat (%)	39.7±2.95	31.2±2.10	37.6±4.78	35.1±6.26	20–35%
Carbohydrate (%)	57.5±1.23	51.8±1.49	54.6±2.07	57.8±1.24	45–65%

Calories reported in Mean ± SD; All variables were energy-adjusted based on the mean caloric intake using the residual method.

**Table 3:** Group differences and time effects of a social media intervention on macro- and micronutrient consumption during pregnancy among adolescents and adult women.

	Adolescent (N = 10)		Adults (N = 12)		Group P-Value	Time P-Value	Time* Group P-Value
Daily Dietary Intake Variable	Pre-Intervention	Post-Intervention	Pre-Intervention	Post-Intervention			
**Calories (kcal)**	1937 (1623, 2885)	2638 (1470, 3287)	2265 (1752, 2681)	2218 (1832, 2811)	0.796	0.364	0.331
**Fat (g)**	97.9±0.9	90.0±1.1	97.7±1.0	90.7±0.8	0.507	**<0.001**	0.070
**Protein (g)**	84.7±1.1	90.0±1.1	84.3±0.9	90.7±0.8	0.753	**<0.001**	0.055
**Carbohydrate (g)**	318.7 (318.0, 319.5)	335.9 (335.5, 336.5)	318.9 (318.4, 320.3)	335.5 (335.2, 336.1)	0.940	**<0.001**	0.075
**Sugar (g)**	124.6±0.9	132.0±1.1	125.2±1.0	131.5±0.9	0.881	**<0.001**	**0.012**
**Fiber (g)**	21.3 (21.0, 22.3)	21.1 (20.8, 21.1)	21.9 (21.3, 23.0)	21.2 (20.9, 21.8)	0.173	**0.013**	0.747
**Folate (μg)**	537.6 (537.3, 537.8)	531.5 (530.3, 532.4)	537.7 (537.0, 537.7)	530.7 (530.2, 531.3)	0.386	**<0.001**	0.170
**Iron (mg)**	19.5 (19.2, 20.5)	21.2 (20.9, 21.6)	19.5 (19.3, 20.5)	21.2 (21.0, 21.3)	0.568	**<0.001**	0.425
**Calcium (mg)**	851.0 (850.4, 851.9)	893.6 (892.9, 894.1)	851.2 (850.6, 851.6)	892.9 (892.1, 894.2)	0.693	**<0.001**	0.477
**Magnesium (mg)**	212.4 (212.0, 213.9)	227.5 (226.9, 227.9)	212.6 (212.2, 214.2)	227.7 (226.7, 228.7)	0.423	**<0.001**	0.169

General Linear Model, Two-Way Repeated Measures: Mean ± SD, Median (25%, 75%); Group (adolescents, adults) was a fixed variable and analyses were adjusted for the following covariates: race, pre-pregnancy BMI, participation in WIC, and maternal age at study enrollment; All variables were energy-adjusted based on the mean caloric intake using the residual method [30]; The following are the AMDR for protein, fat, and carbohydrate respectively: 10–35%, 20–35%, 45–65%. The following are the Dietary Reference Intakes (DRI) for folate (μg), iron (mg), calcium (mg), and magnesium (mg), respectively: 600 μg/d, 27 mg/d, 1300 mg/d, 400 mg/d; Bolded text denotes significant p-value

### Nutrition behaviors & knowledge

Results from pre- and post-intervention nutrition knowledge and behavior surveys demonstrated behavioral differences among adults and adolescents. Adolescents tended to consume fast-food less often with 83.3% and 71.4% reporting consumption ≤once per week at pre- and post-intervention time points, respectively, as compared to 54.5% and 44.4% of adults. Adults were significantly more likely to do both the shopping (pre: p<0.001; post: p = 0.002) and cooking (pre: p = 0.014; post: p = 0.054) in the home as compared to adolescents who relied on a parent or guardian for these tasks. Both adults and adolescents skipped breakfast most often as compared to other meals. The remaining results from the nutrition behavior survey questions are summarized in **Table 4**.

**Table 4:** Nutrition knowledge and dietary behaviors of pregnant adolescents and adult women before and after receiving a prenatal health social media intervention for 18 weeks.

Survey Question	Adolescents (N = 7)		Adults (N = 11)	
	Pre	Post	Pre	Post
How many meals do you typically eat in a day? (meals per day)	3.92±1.0	3.29±0.76	3.00±0.5	3.00±0.6
How many snacks do you eat each day? (meals per day)	3.38±1.5	3.00±1.2	2.55±0.4	2.39±0.7
Do you skip meals on a regular basis?				
Yes to Breakfast, % (n)	17.0 (2)	29.0 (2)	18.0 (2)	33.0 (3)
Yes to Lunch, % (n)	0.0 (0)	0.0 (0)	18.0 (2)	0.0 (0)
Yes to Dinner, % (n)	0.0 (0)	0.0 (0)	0.0 (0)	0.0 (0)
Skip one or more meals, % (n)	8.0 (1)	0.0 (0)	0.0 (0)	0.0 (0)
I do not skip meals	75.0 (9)	71.0 (5)	64.0 (7)	67.0 (6)
Who cooks your meals at home? *^ǂ^				
Parent or guardian, % (n)	33.0 (4)	43.0 (3)	0.0 (0)	0.0 (0)
Participant, % (n)	17.0 (2)	14.0 (1)	73.0 (8)	67.0 (6)
More than one person, % (n)	50.0 (6)	43.0 (3)	18.0 (2)	22.0 (2)
Significant Other, % (n)	0.0 (0)	0.0 (0)	9.0 (1)	11.0 (1)
How often do you eat at fast food restaurants during the week?				
1 time or less, % (n)	83.3 (10)	71.4 (5)	54.5 (6)	44.4 (4)
2–3 times, % (n)	8.3 (1)	28.6 (2)	45.5 (5)	44.4 (4)
4–6 times, % (n)	0.0 (0)	0.0 (0)	0.0 (0)	11.1 (1)
Daily, % (n)	0.0 (0)	0.0 (0)	0.0 (0)	0.0 (0)
More than once a day, % (n)	8.3 (1)	0.0 (0)	0.0 (0)	0.0 (0)
Who does the grocery shopping in your home? *^ǂ^				
Parent or guardian, % (n)	75.0 (9)	86.0 (6)	0.0 (0)	0.0 (0)
Participant, % (n)	8.0 (1)	14.0 (1)	82.0 (9)	89.0 (8)
More than one person in the home, % (n)	17.0 (2)	0.0 (0)	(2)	11.0 (1)

* Indicates a significant difference at the P<0.05 level among adolescents and adults pre-intervention.

^ǂ^ Indicates a significant difference at the P<0.05 level among adolescents and adults post-intervention.

**Fig 2:**
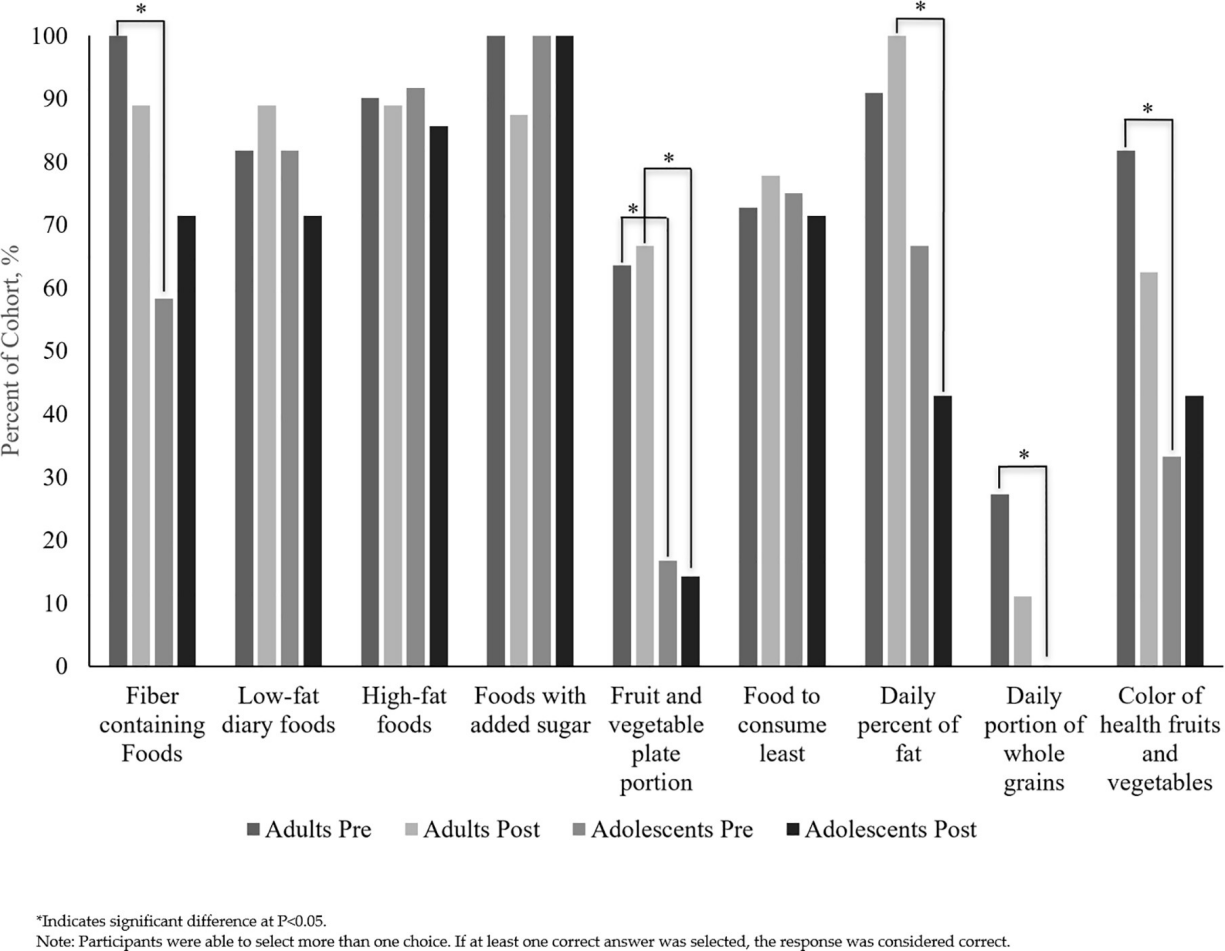
Percentage of correct responses on pre- and post-study nutrition knowledge surveys by pregnant adolescent and adult participants participating in 18-week social media intervention.

Adolescents and adults both lacked knowledge of nutrition recommendations. Prior to the social media intervention, adults were significantly better at identifying fiber-rich foods (p = 0.016), recommended fruit and vegetable intakes (p = 0.021), proportion of daily whole grain consumption (p = 0.052), and that a variety of fruit and vegetable colors are healthy (p = 0.019). After the intervention adults remained better at identifying recommended fruit and vegetable intakes (p = 0.036) and the maximum recommended percentage of daily fat intake (p = 0.009). Remaining nutrition knowledge survey responses are summarized in **Fig 2**.

Following interview analyses, some common health attitude themes emerged (**Table 5**). These themes included encouragement, attitudes regarding weight gain or loss, breastfeeding practices, helpfulness of the intervention, disinterest in cooking, helpfulness of other pregnancy/maternal programs, and hopes or dreams for the baby.

**Table 5:** Frequency of major themes and topics expressed in in-depth interviews by adolescent and adult participants of an 18-week social media intervention aimed at improving health attitudes, knowledge and behaviors.

	Adolescents						Adults					
	Pre (N = 5)		Post (N = 9)		Total		Pre (N = 7)		Post (N = 10)		Total	
	#	%	#	%	#	%	#	%	#	%	#	%
**Health Behaviors**												
Cravings												
*Food Items or Smells*	8	4.0	6	1.9	14	2.7	12	3.5	22	5.5	34	4.6
*Non-Food Items or Smell*	1	0.5	8	2.5	9	1.8	1	0.3	4	1.0	5	0.7
Aversions	3	1.5	6	1.9	9	1.8	10	2.9	3	0.8	13	1.8
Lack of Support	4	2.0	2	0.6	6	1.2	2	0.6	4	1.0	6	0.8
Get Support from Others	10	5.0	18	5.7	28	5.5	17	5.0	15	3.8	32	4.3
Pregnancy Discomforts	18	9.0	27	8.6	45	8.8	23	6.8	22	5.5	45	6.1
Solutions for Discomforts	2	1.0	0	0.0	2	0.4	5	1.5	10	2.5	15	2.0
Disliking Pregnancy	1	0.5	6	1.9	7	1.4	0	0.0	2	0.5	2	0.3
Exercise	**28**	**14.1**	**35**	**11.1**	**63**	**12.3**	**52**	**15.3**	**37**	**9.3**	**89**	**12.1**
Health Information												
*Online*	15	7.5	16	5.1	31	6.0	**40**	**11.8**	**40**	**10.1**	**80**	**10.9**
*Friends and Family*	12	6.0	6	1.9	18	3.5	17	5.0	10	2.5	27	3.7
*Mobile Applications*	1	0.5	10	3.2	11	2.1	17	5.0	12	3.0	29	3.9
*Healthcare Providers*	7	3.5	9	2.9	16	3.1	13	3.8	16	4.0	29	3.9
Dietary Changes												
*Made*	**58**	**29.1**	**64**	**20.4**	**122**	**23.8**	**67**	**19.7**	**67**	**16.9**	**134**	**18.2**
*Reason*	1	0.5	4	1.3	4	0.8	8	2.4	8	2.0	16	2.2
**Health Attitudes**												
Encouragement	2	1.0	6	1.9	8	1.6	4	1.2	15	3.8	19	2.6
Weight Gain/Loss	**21**	**10.6**	**33**	**10.5**	**54**	**10.5**	37	10.9	33	8.3	69	9.4
Breastfeeding Practices	3	1.5	2	0.6	5	1.0	0	0.0	5	1.3	5	0.7
Helpfulness of Study Messages	0	0.0	**42**	**13.4**	42	8.2	0	0.0	**53**	**13.4**	**53**	**7.2**
Uninterest in Cooking	1	0.5	0	0.0	1	0.2	3	0.9	2	0.5	5	0.7
Helpfulness of Other Programs	1	0.5	8	2.5	9	1.8	7	2.1	9	2.3	16	2.2
Hopes and/or Dreams for Baby	2	1.0	6	1.9	8	1.6	5	1.5	8	2.0	13	1.8
**Total**	**199**	**100**	**314**	**100**	**513**	**100**	**340**	**100**	**397**	**100**	**737**	**100**

# represents the number of statements identified in pre- and post-intervention interviews that were categorized into a respective theme. Bolded text represents themes and/or topics that were mentioned more than 10% of the time.

Although all of the adolescent and adult participants reported finding the messages they received helpful during the post-interview, statements related to the usefulness of messages accounted for 13.4% of both adolescent and adult participants in-depth interview discussion themes. Dietary suggestions and recipes in particular were well-received. Specifically, one adult reported adding milk to her coffee after reading a message about the importance of milk. Other messages pertaining to comfortable sleeping and health reminders were also enjoyed. While many messages related to the health benefits of breastfeeding were sent to participants, breastfeeding was only mentioned by three adults and four adolescents during the participant interviews. Both age groups responded well to the format of the messages and enjoyed the friendly, relaxed tone.

The most commonly reported health attitude related to weight gain concerns (10.5% vs. 9.4% for adolescents and adults, respectively). The adults tended to think more long-term about pregnancy, whereas the adolescents’ thoughts were focused in the present on personal feelings related to weight. The adult participants seemed to think about the long-term health consequences of their own weight gain and the impact of their health choices on their growing babies. **Table 6** provides representative participant quotes for each of the major themes for both adolescents and adults.

**Table 6:** Representative quotes expressed by adolescent and adult participants regarding health attitudes and behaviors while participating in a social media intervention to improve health during 18 months of pregnancy.

Theme	Adolescents (N = 10)	Adults (N = 12)
**Health Behaviors**		
Cravings for food or food smells	“Like I had to have a big mac and some fries. And if I didn’t I’m going to scream and yell until I do.”	“I’ve started to crave sweet things more which I didn’t at all before, and I didn’t really tolerate well…”
Cravings for non-food items or smell	“And when something smells good to the brain, well the brain tellin’ you know, your body that it smell good.”	“Um I actually tried baby powder one time, so that's how strong it was but then I talked to the doctor and then I had to really just not, not do it.”
Aversions	“I didn’t drink coffee at all in the beginning of my pregnancy, I didn’t want it.”	“But it was just the sauce smell that got to me and I threw it all up, and that was the first time in my whole pregnancy, so I know it was because of that.”
Lack of support from others	“Yeah, but, I don’t talk to people about that. To me it’s strange.”	“My husband’s not as excited to change. If I had a very pliable family, I probably would’ve changed a little bit more.”
Support	“Yeah. My mom went overboard. Like she bought him a pair of sneakers he can’t wear until he about one.”	“Um, He was very involved in a good way, very nervous as well, so.”
Solutions for discomforts	“At work not really, well I am cause if not I’ll fall asleep, so I’ll get up and take a bathroom break and walk around a little.”	“Really just laying in bed overnight makes the ankles go back, but they just swell again. Uhm and then with the hips and stuff, I just put Icy Hot on a lot of the time and hope for the best.”
Disliking pregnancy	“I’m just really excited to not be pregnant anymore cause it’s hard to move around and stuff.”	“Oh boy. To be perfectly honest, I hate being pregnant, so (laughs) that’s a tricky one.”
Pregnancy discomforts	“I was tired, falling asleep in class, like I’m trying to stay up and I’m just like dosing off and it was hard so.”	“Uhm I would like to say my hips and tailbone have hurt much, much more than last time, and I’ve also swollen more than I ever did.”
Exercise	“But I do try to take walks like when I go to school. They got like the treadmill. And I walk on that for a whole period, and stuff like that.”	“Um, and you’ve also got the free gym membership for the duration of the class, so, um…I’ve always been someone who’s joined in a- who’s been a gym member and who has worked out.”
Pregnancy-Related Health Information		
*Online*	“And then I usually if I have a question… I just Google it and read a whole bunch of different takes on it so I go to like multiple websites.”	“Oh, and I also looked up different …um…like prenatal exercises, that’s a big one that I’ve looked up online, for like back stretches and stuff like that.”
*Friends and Family*	“My sister, she has a son and that was one of the things that she used so I just downloaded it.”	“Uhm, so my friends have said that parent groups, like Facebook groups have been really helpful for them.”
*Mobile Applications*	“No, I have this app downloaded that gives me info called pregnancy tracker it updates me on things about pregnancy.”	“And uhm it tells you like the baby growth, what the baby should be doing, it also tells you like what type of foods and stuff to eat that would be good.”
*Healthcare Providers*	“I felt like… guided and comforted cause they knew what to do with me, and I didn’t have to go through a lot of trouble.”	“I think I really like talking to my midwife, just cause there’s so many things that you’ll read about or like you’d be told….so yeah, she’s like my last my arbiter of truth.”
Dietary changes	“Um, before we probably ate out like once a week, but now it’s probably only like one or twice a month.”	“Uhm well just taking care of your body in general. I don’t like to eat a lot of… super processed candy or food… or in general I don’t really like food like that, but I’m more stringent about it when I have a baby.”
Reason for change	“But I been trying to drink the cow milk just because, you know, they add the more vitamins.”	“But I know that mercury level fish and like different sea life have different mercury levels… are the ones that you’re supposed to avoid.”
**Health Attitudes**		
Encouragement	“It depends if they’re (exercise or physical activity) fun or not, cause some of them be boring so you just don’t want to do them.”	“Um, I’m hoping to be motivated just because it makes me feel better. And I know that from running before, that working out makes me feel better, so.”
Weight Gain/Loss	“It’s like I got a phobia to be over 200 pounds. Like it’s just, I just don’t want to be fat. I’m scared.”	“Weight is not really an issue to me because I know that it can come off.”
Breastfeeding practices	“Breast feeding, but I don’t, I’m not doing that. That to me feels uncomfortable.”	“And it’s all the things I hear about breastfeeding does for the baby and you, I was like, I definitely gotta do that.”
Helpfulness of Intervention	“I used to read the little picture things, that came with the picture, I used to read them all the time. And like give those a try.”	“Most of the time I’ll say, “Oh yeah, I think I knew most of it.” But there’s always a couple things you learn about and little tips or ideas you get.”
Uninterest in cooking	“Probably it the hours that I work make me tired, so I don’t cook anything or prepare anything, so I order food.”	“I used to be, you know, very enthusiastic about looking up new recipes and trying new things, and at this point, I kind of lack in that kind of enthusiasm.”
Helpfulness of other apps/websites/texting programs	“But like the questions that I really be looking for, most the time they have the answer for me on here. I just click on a whole bunch of different sites to see like if this one is not right and this one. Like if these two are not the same, something not right here.”	“Um, I mean I, it’s not the best app but the most interesting thing for me was the, the countdown, which is helpful when you get you know to the uncomfortable stage and you’re saying, ‘only 70 days left!’”
Hopes/Dreams for baby	“I don’t really think about the future. I like to live in the present.”	“(I) hope that the baby is healthy, and um grows up feeling safe and loved by you know family and friends.”

In an analysis of the interviews conducted with study participants, some common health behavior themes emerged (**Table 5**). These themes included cravings for food and non-food items or smells, aversions, lack of support or feelings of support, pregnancy discomfort and solutions, general dislike of pregnancy (not related to discomfort), exercise, and sources for pregnancy-related health information, dietary changes and reason for change. Among adult study participants, the most common themes were dietary changes (18.2%), exercise (12.1%), and using the internet as a source for pregnancy related health information (10.9%). The most frequently reported themes among adolescents were dietary change (19.7%), exercise (12.3%), and pregnancy discomfort (8.8%).

Both adults and adolescents mentioned making dietary changes during pregnancy. The majority (69%) of the adult participants stated making dietary changes for the health of their baby. These changes included cutting out soda or sugary drinks, avoiding fried foods, reducing consumption of desserts, and increasing meat consumption for protein. Whereas, adolescents reported that their dietary choices were driven more by time, convenience, and food preferences, rather than for health reasons (personal or for the baby). Adolescents reported healthier dietary changes such as limiting fast food and high-fat foods, trying new vegetables, increasing fruit intake, and eliminating coffee.

Both adolescents and adults mentioned engaging in exercise during pregnancy. Adults mentioned working out either at home using videos, working out at gyms, or attending group workout classes whereas adolescents mentioned working out at school through the Young Mothers Program or going for walks around the neighborhood, to work, or during school. Adult women stated that they exercised during pregnancy not only for the health of themselves and their babies but also to help with postpartum weight loss. On the other hand, adolescents tended to exercise because they believed that it would make labor easier. Regardless of reasons, all adult and adolescent participants reported walking or other exercise during pregnancy.

Another large theme that emerged from the interviews was how health information was obtained. The majority (>85%) of the adult population reported using the internet, family/friends, phone applications, and healthcare providers prior to the intervention. However, the internet, mobile phone applications, and reaching out to family or friends for health information were each used by only 50% of the adult population post-intervention. All adolescents used the internet to obtain health information whereas only 60% went to family and/or friends and 20% used mobile phone applications pre-intervention. Adolescents used the internet (75%), family or friends (50%), healthcare providers (50%), and mobile phone applications (38%) for health information more often by the intervention end.

High levels of access to social media were reported for all participants. The majority (91%, N = 20) of participants had a cell phone that could receive SMS. Only one adolescent and adult participant had to pay for their text messaging service. Teens and adults received 55 (IQR: 51 to 62) and 42 (IQR: 37 to 45) SMS, respectively, over the course of the intervention. High levels of access to Facebook were also reported; all participants except one adolescent had a Facebook account. A median of 86 (IQR: 74 to 88) messages were sent to adolescents with each teen viewing a median of 40 (IQR: 24 to 63) of these messages. Of the 65 (IQR: 58 to 66) Facebook messages that each adult participant received during the intervention, a median of 60 (IQR: 31 to 64) of the messages were viewed by each participant.

Study-wide shortlink use during the study was tracked by viewing the number of clicks on each link within the Bitly built-in analytics system. Total clicks were low for adolescents with only 11 overall interactions. Messages pertaining to recipes and weight gain were the most popular, with 63% of clicks falling within these categories. Numerous messages regarding exercise were sent out but only one click was recorded. Participation by adults was much higher with a total of 49 clicks recorded across all 10 message categories. Links with the most adult interaction contained exercise and weight gain messages. Other categories of interest to adult women were healthy snacks, recipes, and relaxation/music.

## Discussion

This longitudinal pilot study demonstrated that social media messaging is an effective mechanism to deliver nutrition and health-related information to low-income, pregnant adolescents and adult women. During the intervention period, we observed reductions in fat and folate consumption, and slight increases in sugar, magnesium and calcium intake among participants. Furthermore, this study found differences regarding dietary changes with adults making changes for the health of their baby and adolescents making changes due to time and convenience. To our knowledge, this is the first study to assess the effects of social media on dietary intake and nutritional attitudes during teen pregnancy. Overall, both adolescent and adult participants enjoyed receiving study messages via social media and found the information useful for prenatal health.

This study found that adolescents had a statistically larger percent decrease in fat consumption than the adult women across the intervention. This is interesting considering the literature indicating that adolescents tend to consume high-fat diets [32]. At baseline, adolescents and adults both reported fat intakes greater than the AMDR, which decreased to within the AMDR for adolescents at the end of the intervention. Previous literature suggests conflicting results for macronutrient consumption. One study found no significant changes in macronutrient composition among healthy adult women [33], while another study observed an approximate 6% increase in saturated fat between the first and second trimester of healthy pregnant women [34]. Unfortunately, these studies did not assess fat consumption in relation to pregnancy health including data on weight related outcomes. This is important because high-fat diets during pregnancy have been associated with increased fetal mid-thigh adiposity [35], while increased carbohydrate and low-protein diets have been associated with greater fetal abdominal adiposity [35,36]. Thus, the replacement of high-fat foods with lower fat, nutrient-dense alternatives may be a positive dietary strategy that merits investigation in relation to pregnancy health outcomes.

Over the course of the intervention, total daily sugar intake increased by 8 g and 6 g for adolescents and adults, respectively. Although the increase in sugar consumption among study participants was very small, this was the opposite of our study hypotheses. Sugar intake among adolescents contributed approximately 25% of total calories which is higher than the national average (16.6%) [37] and WHO recommendation (<10%) for consumption of added sugars [38] but lower than the 44% of a similar adolescent cohort participating in the Camden Study [11]. Although the high-sugar intake among participants in the current study included foods such as fruit, many of the teens consumed sugar from cakes, cookies, and sugary cereals. High sugar consumption during pregnancy has been linked to an increased risk for small-for-gestational age [39] and low birthweight deliveries [39], greater fetal abdominal fat [36], excessive maternal weight gain [40,41], large-for-gestational age fetuses [39,40], and greater maternal complications [40]. Previous research has found that extreme sugar intake (i.e. both low and high) was positively associated with fetal abdominal fat accumulation with minimal net effect on birth weight [36]. This shift in fetal body composition related to maternal sugar consumption suggests the possibility of an optimal intake range for specific nutrients during pregnancy. Thus, accurate tracking of total daily consumption of sugars during pregnancy may potentially reduce maternal and neonatal complications.

EGWG has been associated with a multitude of negative health outcomes in adolescents [1,42–44], with research showing more gestational weight gain among adolescents than adults [1]. However, we did not find a significant difference in weight gain among the women in our cohort which may be due to the intervention, sample size, or quality of prenatal care received. Overweight or obese women tend to gain greater amounts of gestational weight than women with a normal pre-pregnancy BMI [14,42,45] which was apparent among our participants with 67% and 71% of overweight/obese adolescent and adult women gaining excessive weight, respectively. The rate of excessive gestational weight gain was higher among adult women (67%, N = 8) when compared to adolescents (40%, N = 4). Among overweight and obese participants, 69% (N = 9) experienced EGWG as compared to only 60% of their normal-weight counterparts. Overweight and obese women are already at increased risk for short- and long- term maternal and fetal health consequences. Therefore, appropriate gestational weight gain among adults and adolescents is imperative in order to manage poor clinical outcomes and reduce long-term disease burden.

Currently, most prenatal nutrition research focuses on adult women, with few studies examining the diet quality and nutrition knowledge of low-income, pregnant, adolescents [16]. Research using social media to target diet quality and nutrition knowledge among low-income, pregnant adolescents has not been explored, despite data suggesting that teens have access to and respond well to social media [12,18–20]. Congruent with current literature, the adults and adolescents in this study had regular access to Facebook and text messaging [12,19,20,46,47], indicating a potential mechanism to deliver nutrition and dietary information. Studies have shown that nutrition information provided via social media or text message is an effective means of education for low-income teens and young mothers [12,48,49], as well as WIC participants [47,48].

Our finding of mild positive dietary changes (reduced fat and increased mineral consumption) among participants is consistent with a recent study concluding that a text message intervention increased healthy behaviors among pregnant adults [50]. However, the current study did not demonstrate an improvement in nutrition knowledge indicating a potential need for a more multifaceted approach. A recent study of pregnant teens found that 25% of participants received healthy eating information from school [51], indicating that both school and social media interventions may be needed in order to fill this education gap. Additionally, previous research has found consistent use of online weight-trackers among low-income minority pregnant women [13,52], thereby demonstrating the effectiveness of digital communication for improving pregnancy health behaviors.

Many adolescents in this cohort did not report being encouraged to make healthy dietary changes by others, an attitude that has been previously reported [12]; however, they did mention that they had made some dietary changes to improve their health during pregnancy. Previous research has demonstrated that pregnant adolescents with an interest in healthful eating behaviors still consumed fast food and soda [51]. Our data mirror this finding with the majority of adolescents reporting consistent consumption of fast food once per week across the intervention. Further, at the end of the intervention two adolescents were reporting fast-food consumption 2–3 times per week. Despite the knowledge of healthy eating habits during pregnancy, adolescents seem to have difficulty comprehending the information, overcoming cravings or personal food preferences, and applying new nutrition knowledge into action [12]. Therefore, social media in combination with other tools may be effective agents of change for improved pregnancy outcomes among adolescents.

The present study showed a mix of attitudes regarding pregnancy weight gain, with mostly positive attitudes among adolescents. Attitudinal data regarding pregnancy weight gain have been inconsistent with higher rates of positive attitudes among teens reporting greater family support or having lower pre-pregnancy weights [46]. Other teens report negative feelings about pregnancy weight gain including feelings of embarrassment and unattractiveness [46]. Past studies reported more positive attitudes towards pregnancy weight gain among pregnant adults [46,53]; however, women with greater weight gain did report more negative feelings [53]. Appropriate gestational weight gain is crucial for adequate growth and development of the neonate; therefore, interventions that deliver positive messages regarding maternal body composition changes during pregnancy may improve feelings and attitudes among the women.

This study targeted low-income, pregnant adolescents and adult women with a social media intervention, which is an area of research with little prior exploration. Strengths of this study include the adult comparison group recruited from the same low-income serving clinic which allowed us to obtain relatively equal demographics between the two groups of women. Additionally, the study was cost-effective, providing health and nutrition information to participants via fast, accessible, and timely media sources. Limitations of this study were implementing 24-hour diet recalls during scheduled clinic visits. Sharing clinic time with practitioners could have limited our ability to capture all forgotten or missing food and beverage items ultimately influencing the quality of recall data obtained. Additionally, only total sugar intake was provided by Food Processor, which resulted in the inability to accurately calculate added sugar consumption. The higher sugar intakes in adolescents may have been confounded as many of the teens received fruit and vegetable supplements from the WIC program. Furthermore, the small sample size used in the study may have limited the ability to obtain significant results. Many participants in the study were already 25–28 weeks pregnant at the time of enrollment limiting the intervention time. Additionally, we did not obtain gravidity or parity data for the adult participants. Previous prenatal visits may or may not have included nutrition counseling and/or exposure, which might have impacted baseline data for this group. Lastly, findings from this intervention may not be generalizable to a larger population as this cohort was recruited from a single clinic.

## Conclusions

This pilot study indicated that poor overall diet quality persisted among low-income, pregnant adolescents and adults. Despite increases in calcium and magnesium, these and other important nutrients (e.g. fiber and iron) fell short of recommendations in both age groups. Despite the participants enjoying receiving social media information regarding pregnancy health, further efforts are needed to educate this high-risk community on prenatal nutrition and to motivate dietary change for maternal and child health outcomes. This is especially important considering that the majority of participants who entered pregnancy as overweight or obese were more likely to experience EGWG during the intervention. Although teens have increased access to social media, the adults had higher levels of participation during this study. Future interventions are needed to further evaluate the type, frequency and sources of sugar consumed by this population and the best way to implement social media interventions in an effective and enjoyable way.

## Supporting information

S1 Fig Photo messages shared with participants. (DOCX)

S1 File Pre- and post-intervention nutrition knowledge questionnaire. (PDF)

S2 File Electronic data usage survey. (PDF)

S3 File Formative interview guide. (PDF)

S4 File Social media intervention data. (XLSX)

S5 File Nutrition knowledge pre- and post-intervention survey data. (XLSX)

S6 File Pre-Interview transcripts. (PDF)

S7 File Post-Interview transcripts. (PDF)

S1 Table Message content for social media posts. (DOCX)
