# Supplementary material for: A social media intervention to improve nutrition knowledge and behaviors of low income, pregnant adolescents and adult women
Source: PLoS One. 2019 Oct 24;14(10):e0223120. doi: 10.1371/journal.pone.0223120 (PMC6812786; doi:10.1371/journal.pone.0223120)
Supplement: S1 Fig — (DOCX) [file pone.0223120.s001.docx]

| **S1 Figure** Sample Photo Messages | | |
| --- | --- | --- |
| 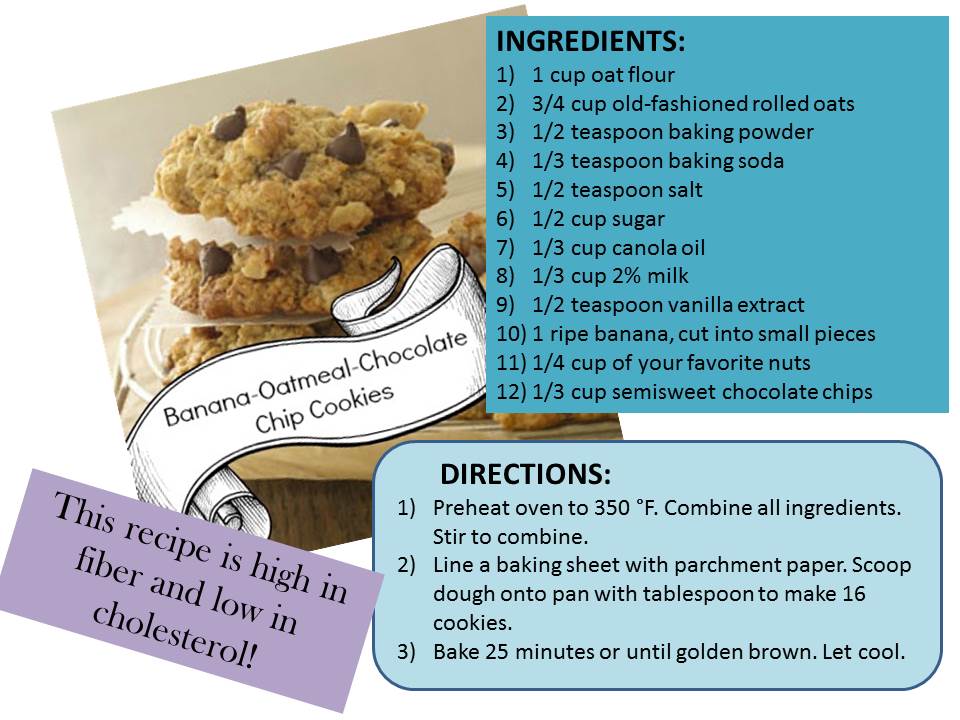 |  | 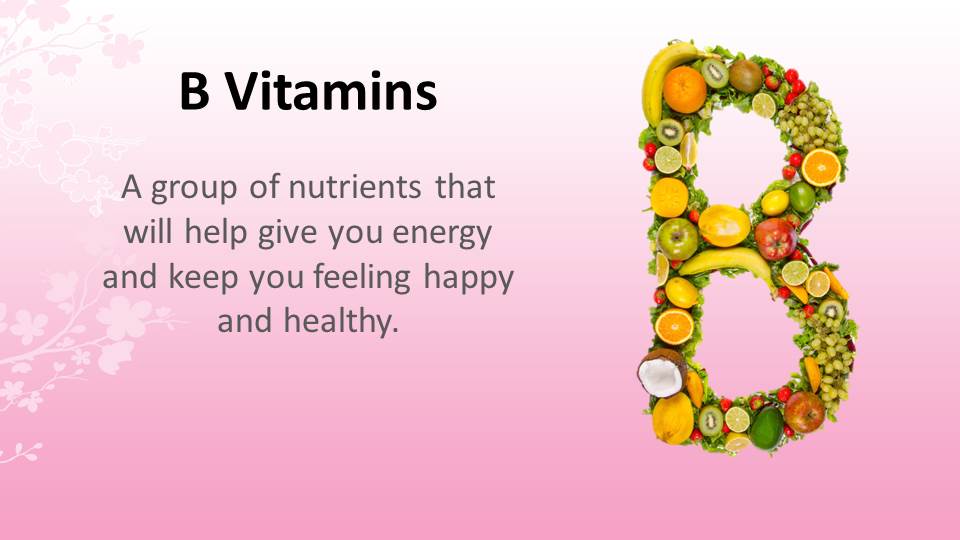 |
| 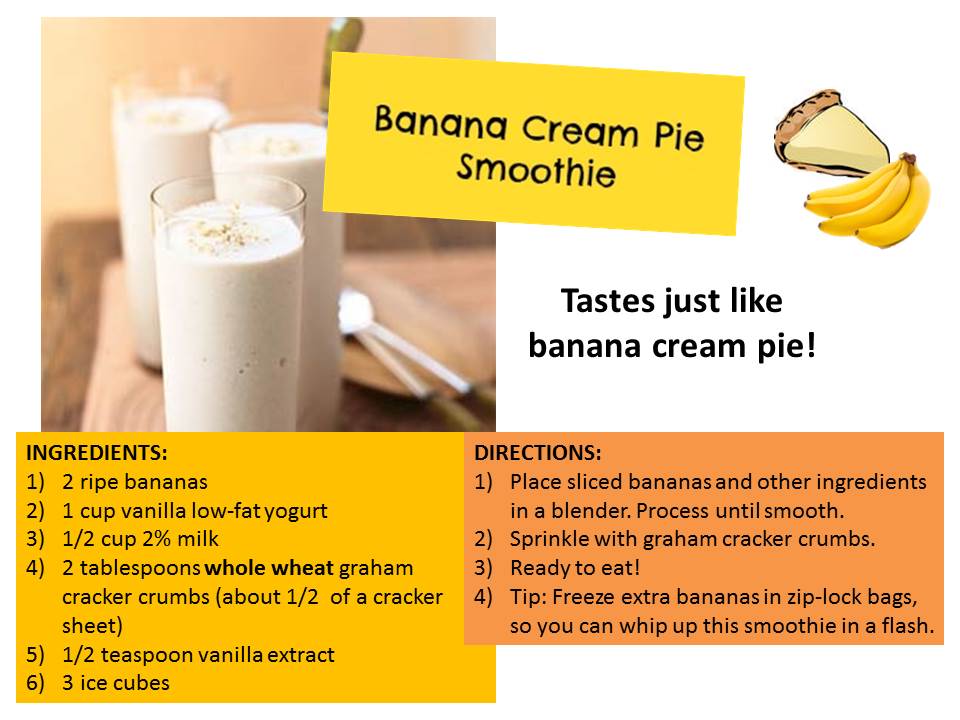 |  | 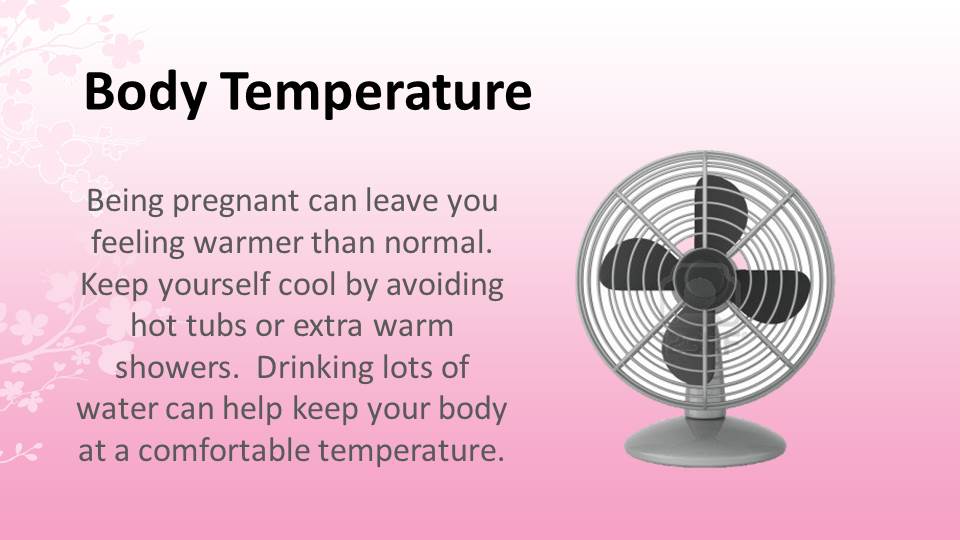 |
| 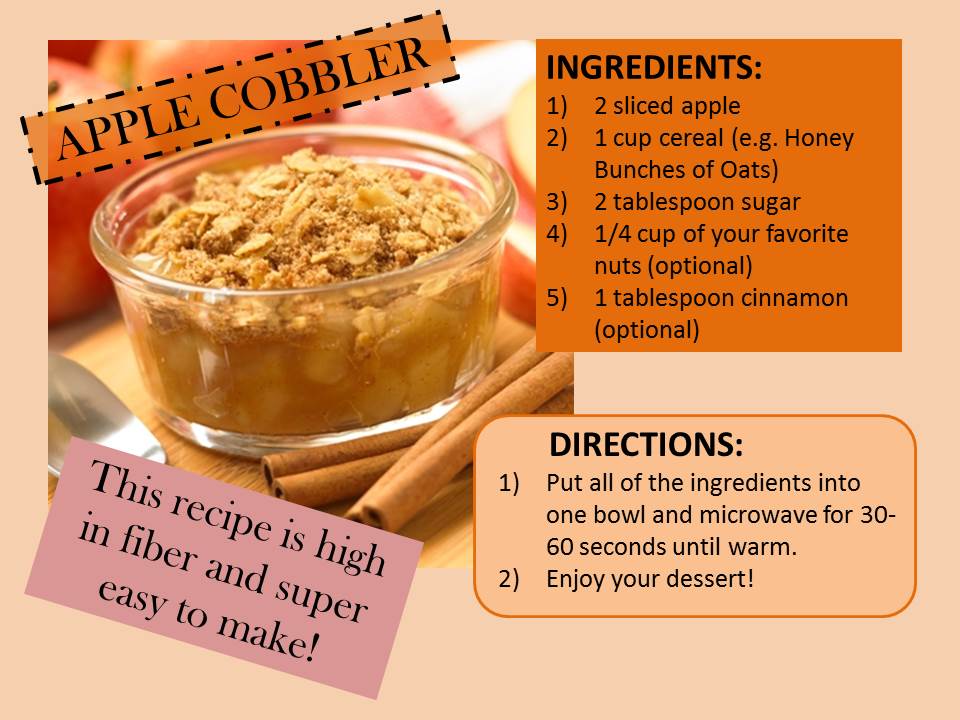 | 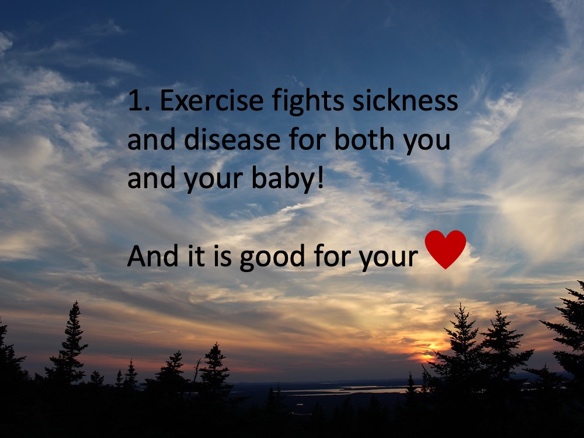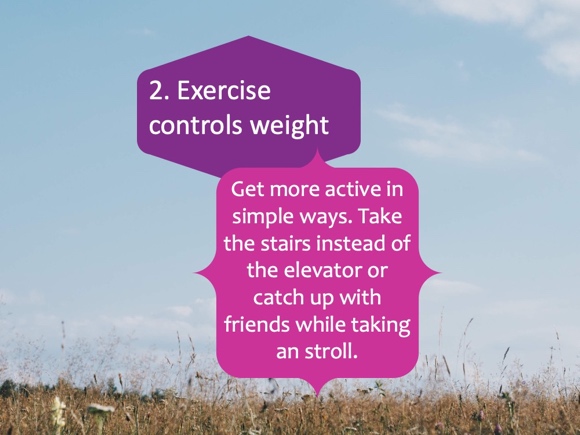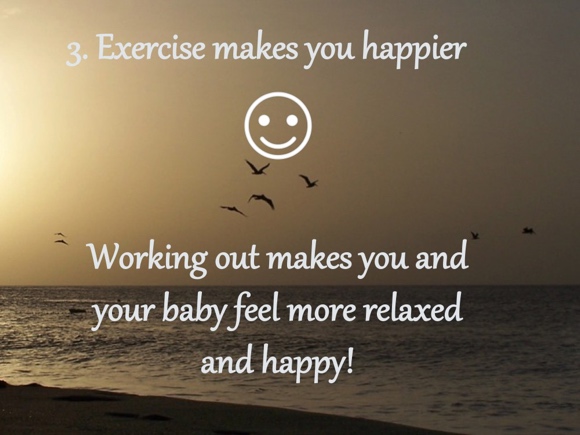 | 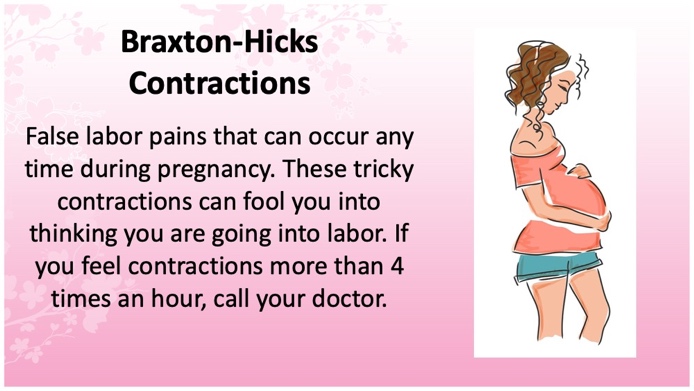 |
| 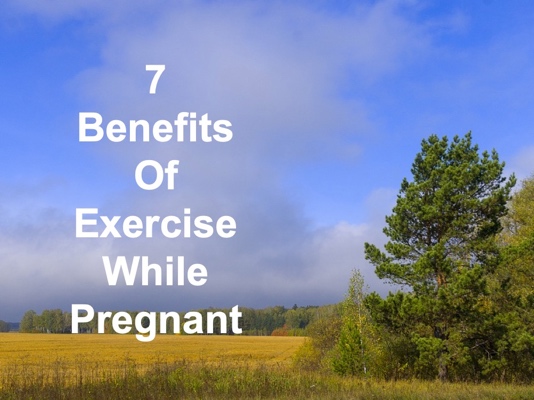 |  | 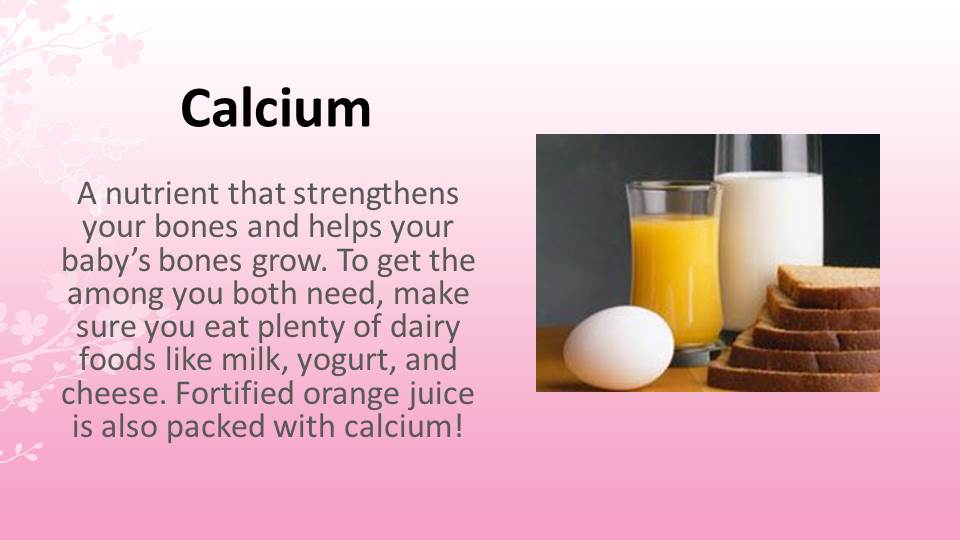 |
| 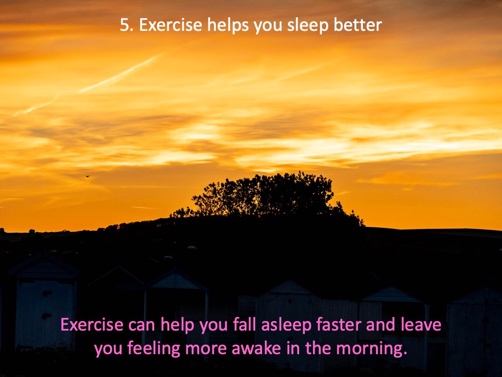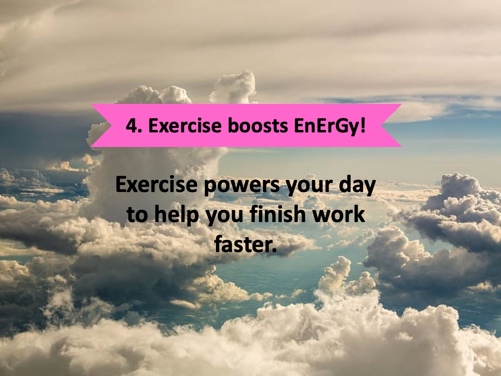 | 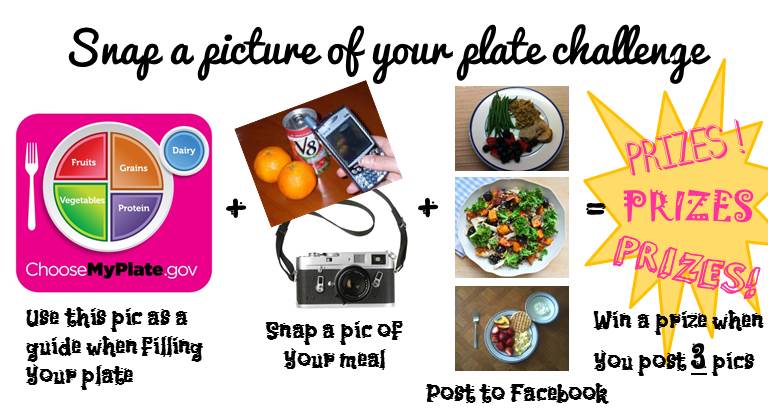 | 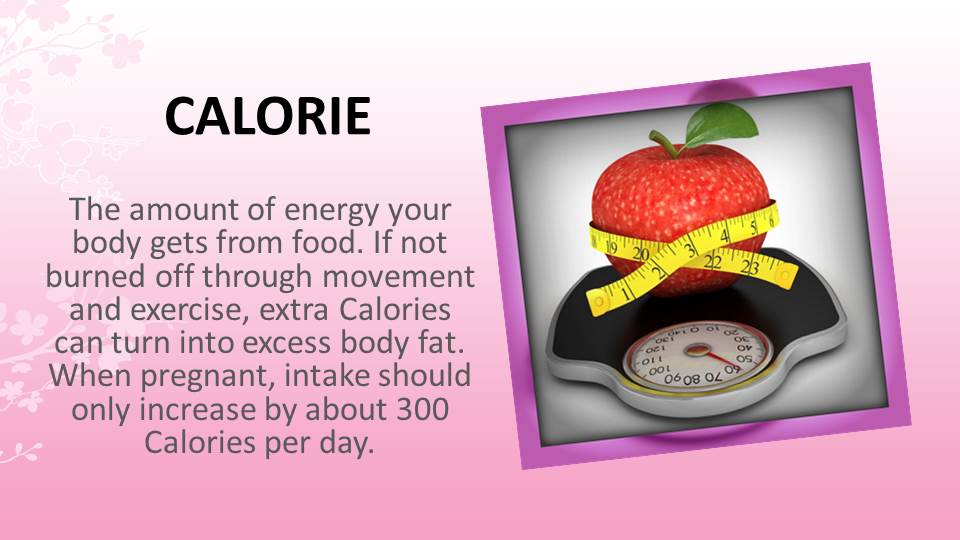 |
|  | 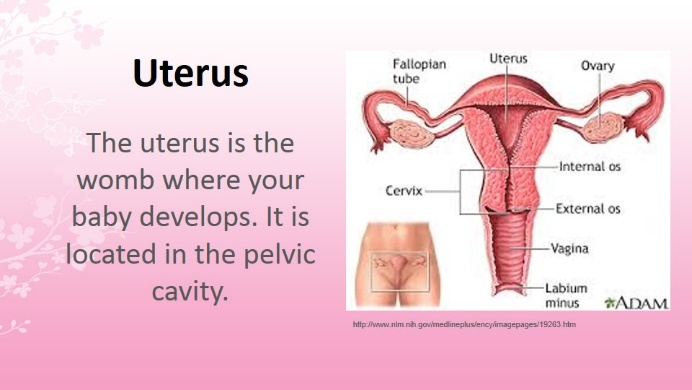 | 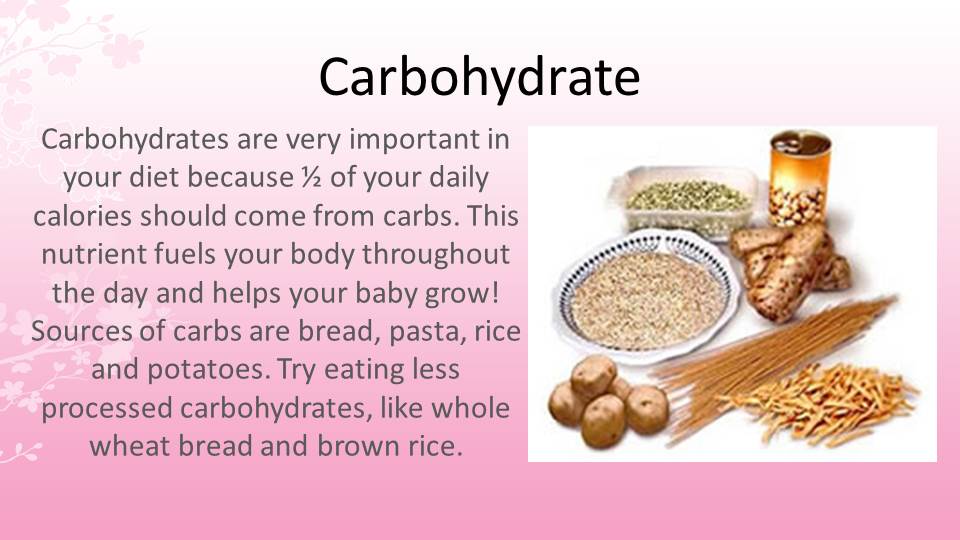 |
| 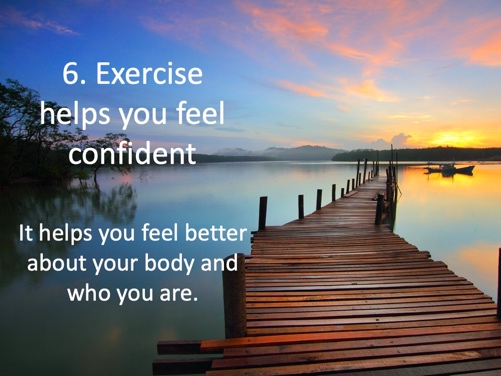  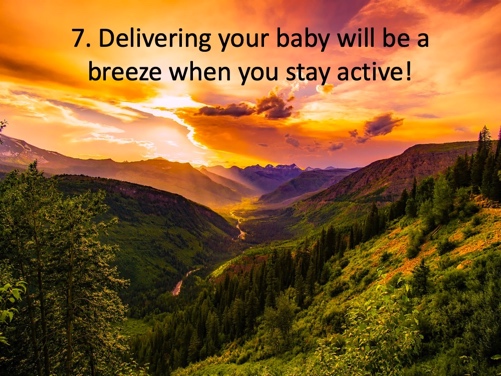 |  | 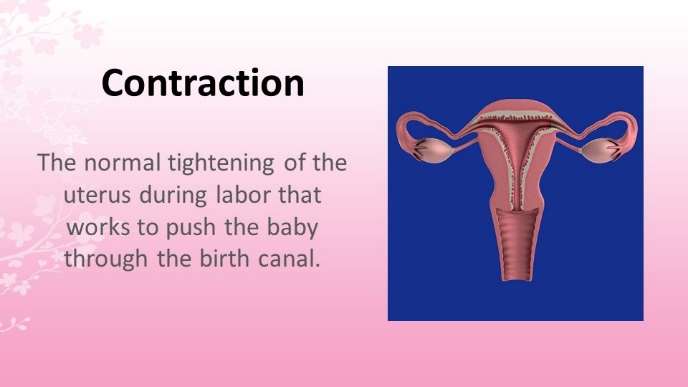 |
| 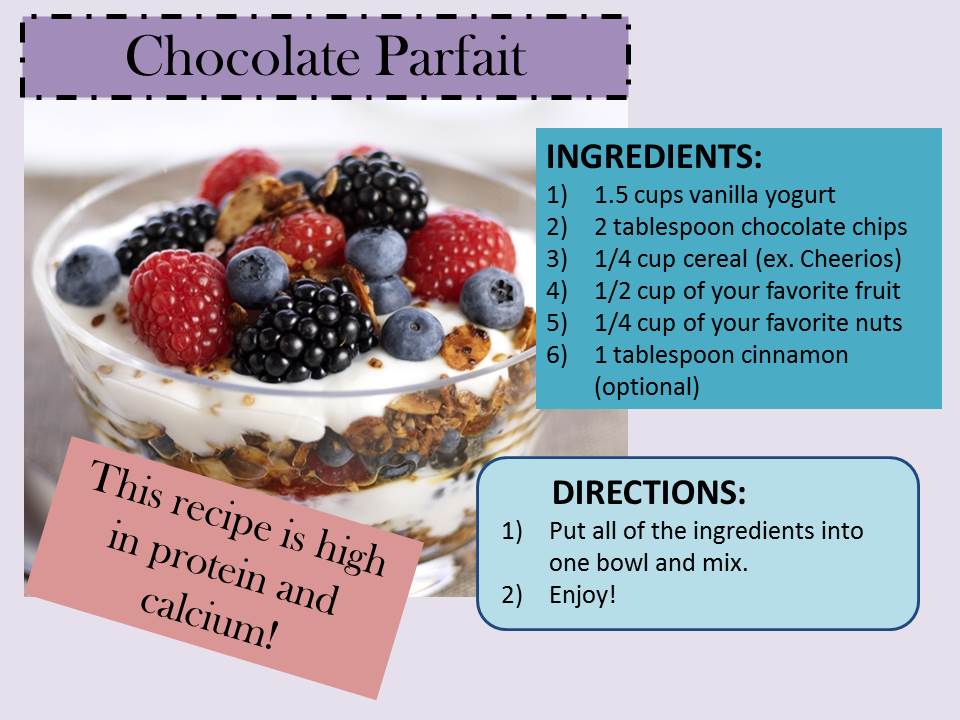 | 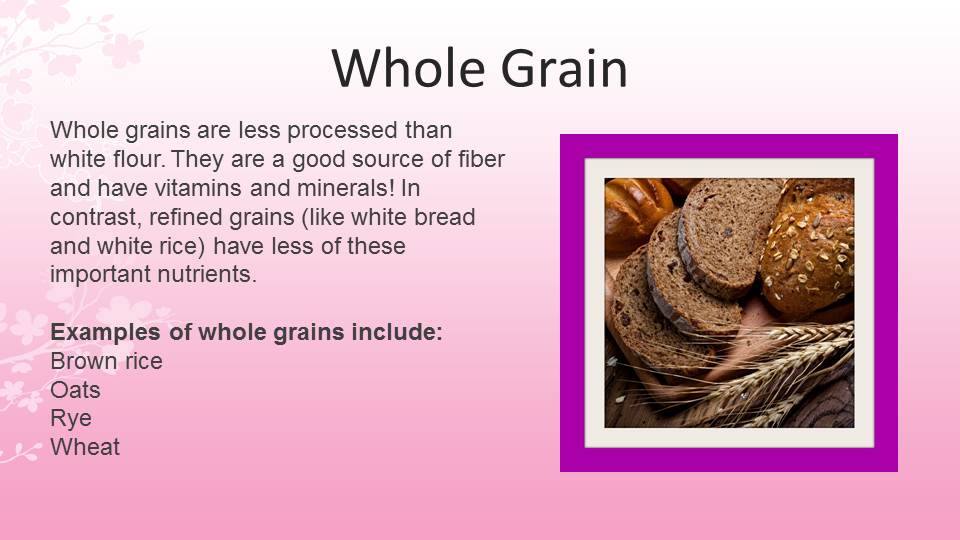 | 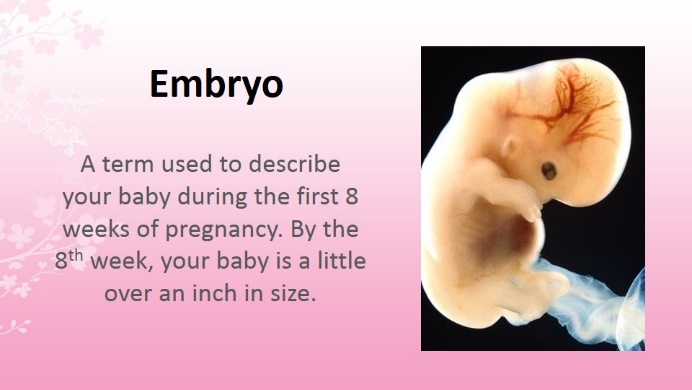 |
| 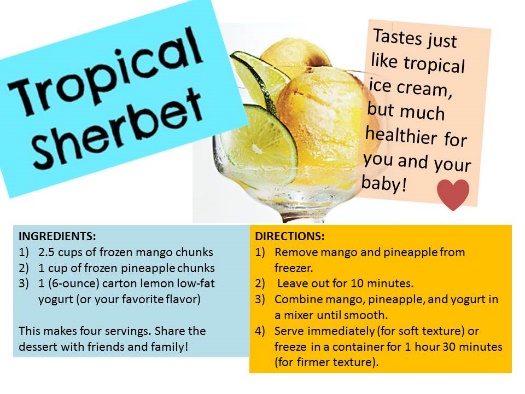 | 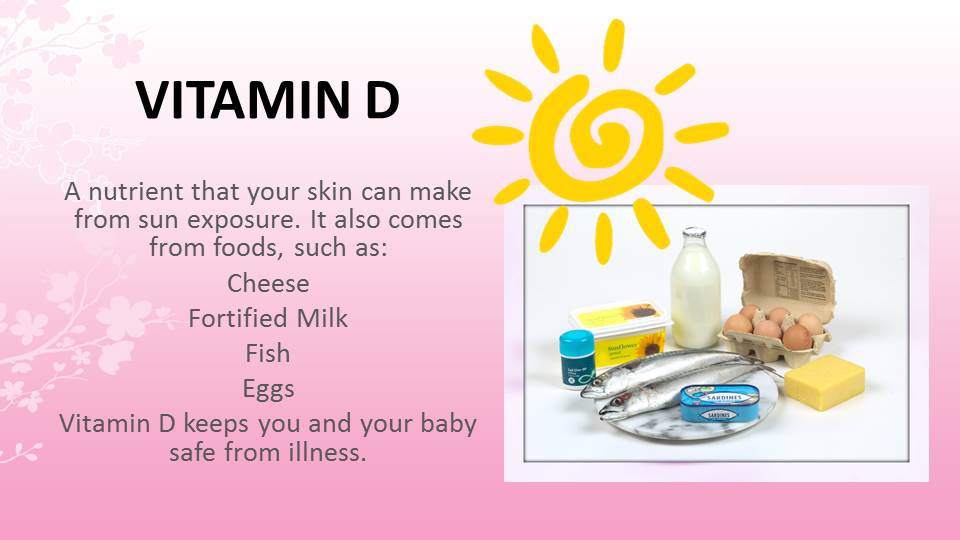 | 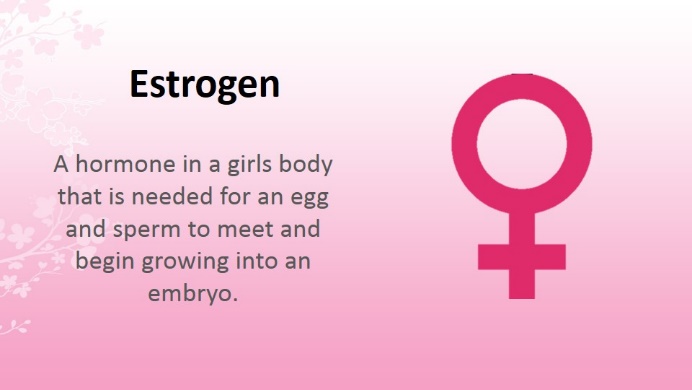 |
| 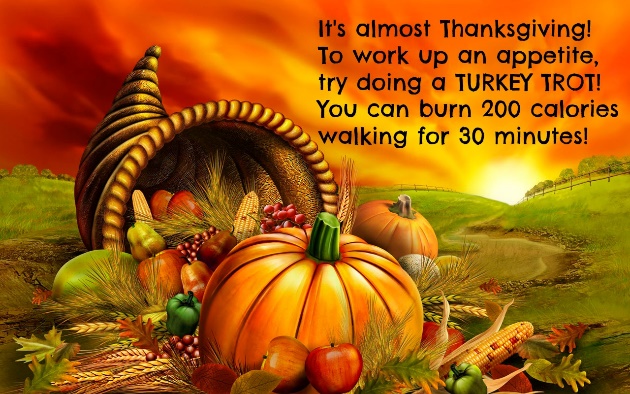 | 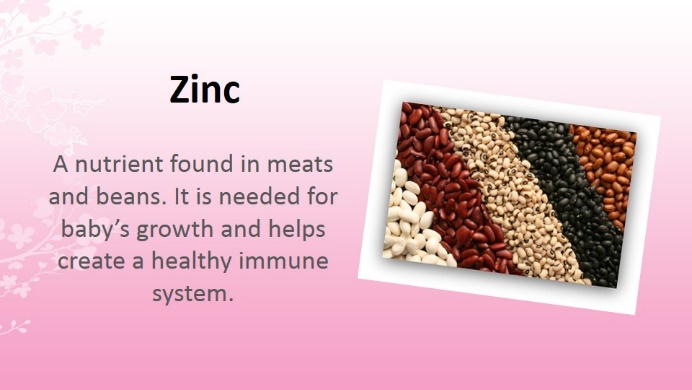 | 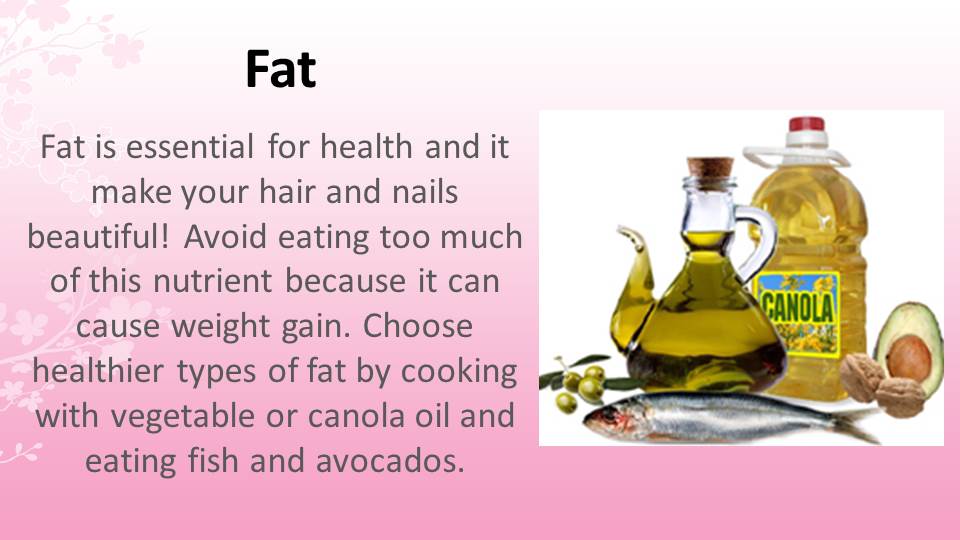 |
| 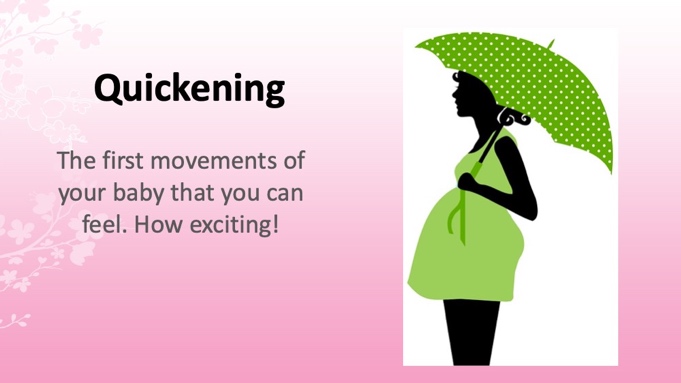 | 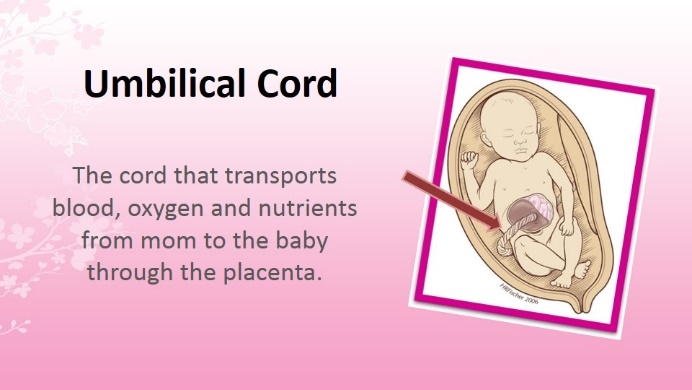 | 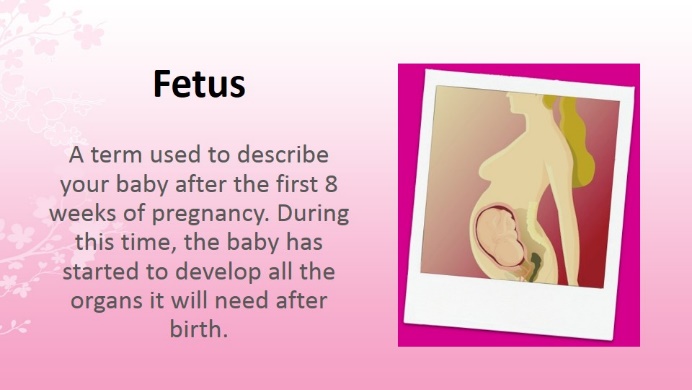 |
| 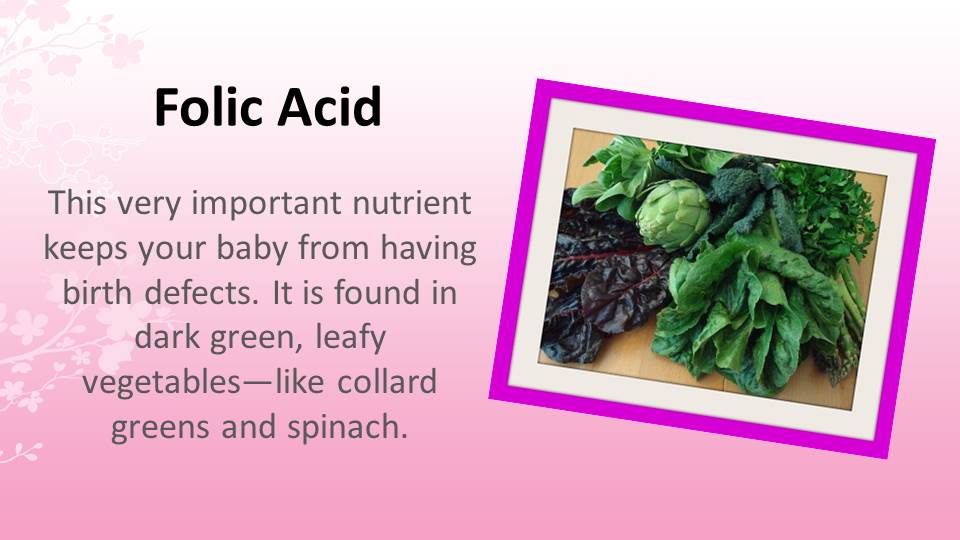 | 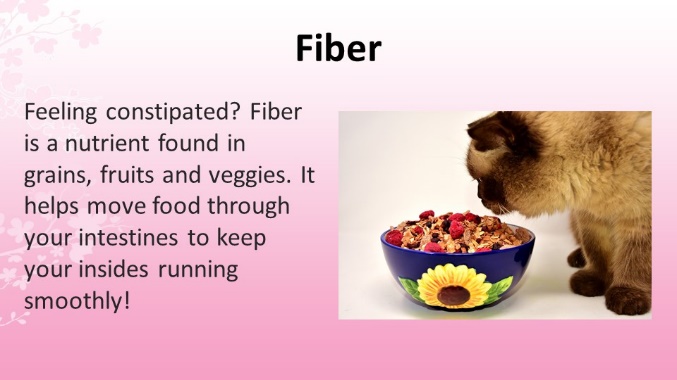 | 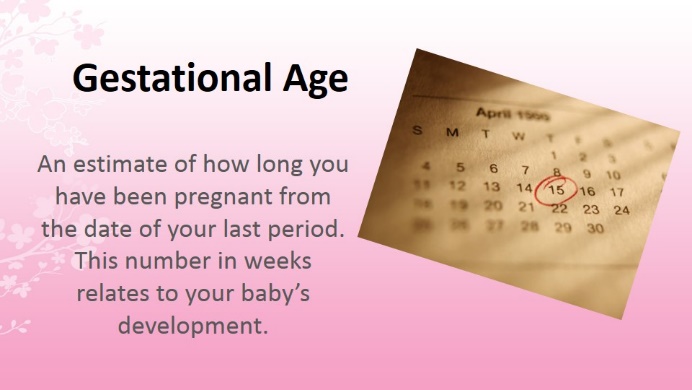 |
| 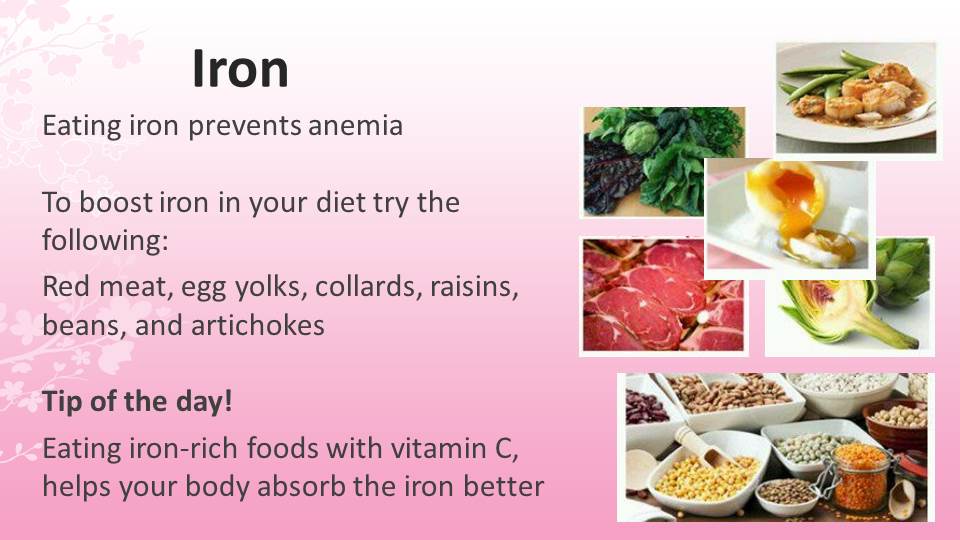 | 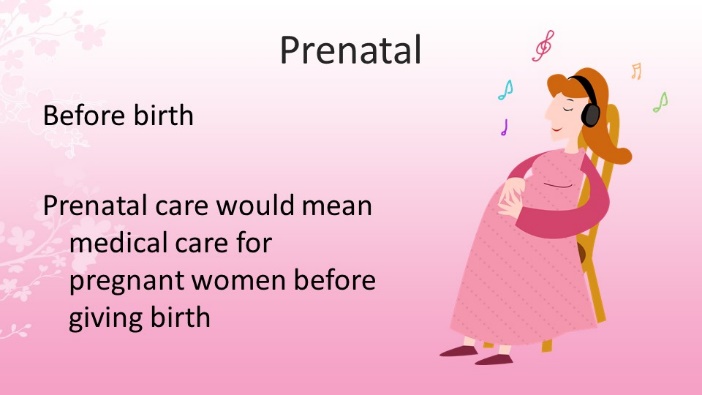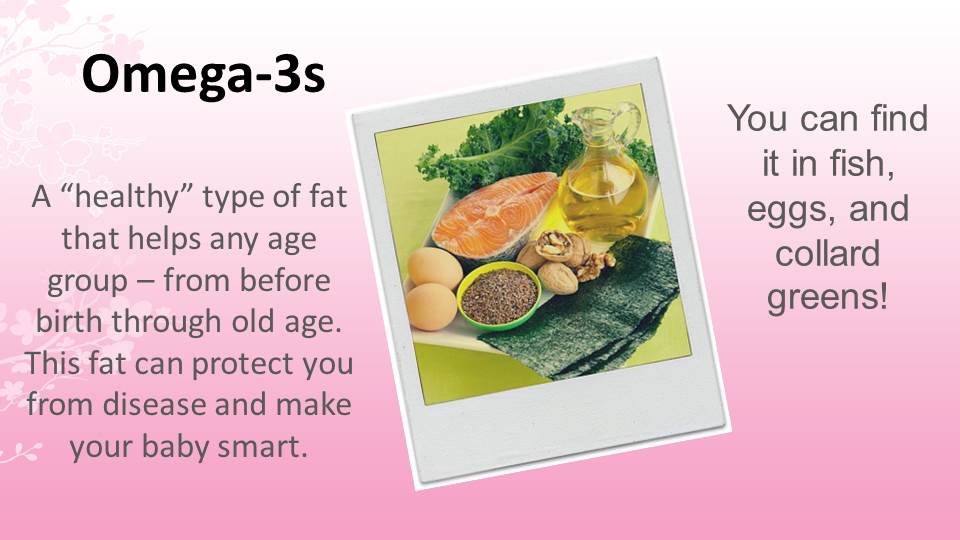 | 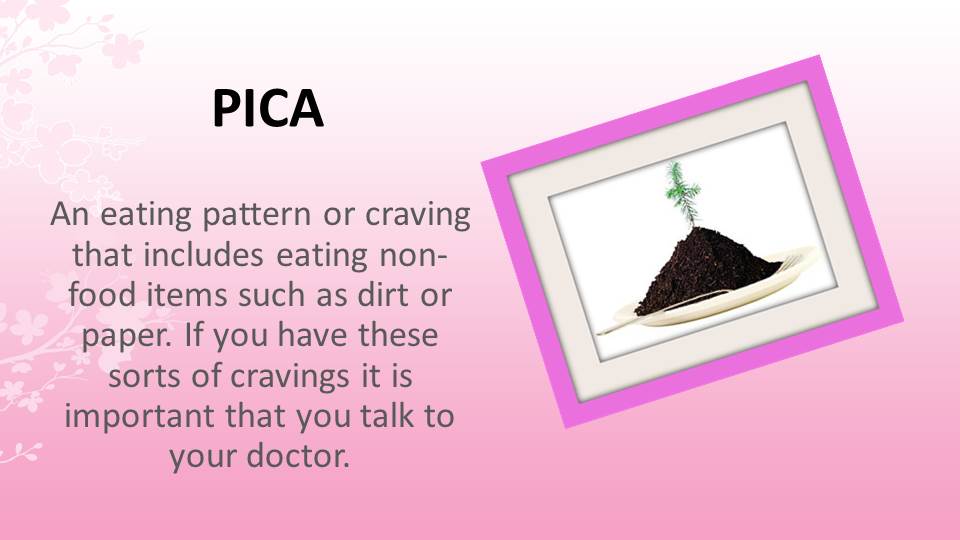 |
| 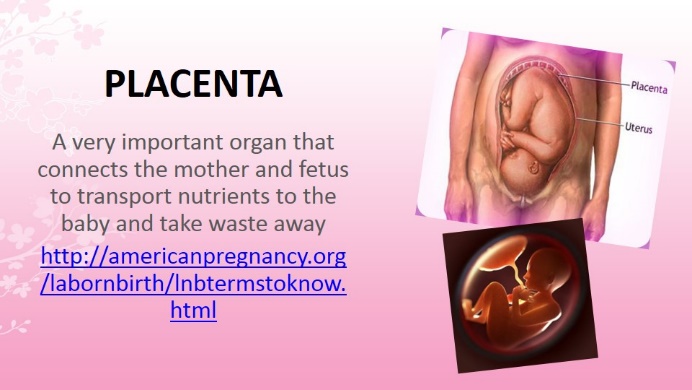 |  | 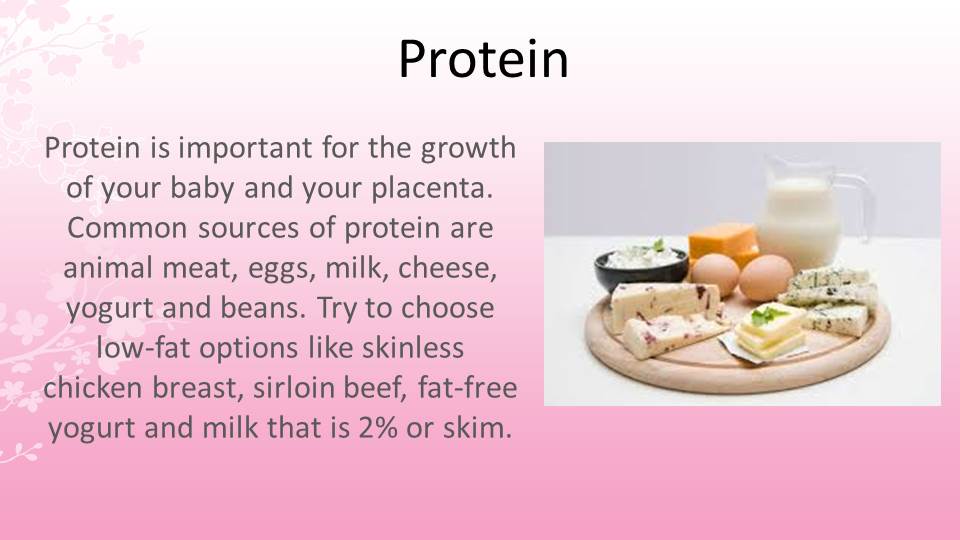 |
|  |  |  |
|  |  |  |

All images were obtained from Microsoft clipart or stock image databases accessed through search.creativecommons.org. Images used were either released to the public domain or were available through creative commons licensing that permitted use without a requirement for attribution.
