# Supplementary material for: A social media intervention to improve nutrition knowledge and behaviors of low income, pregnant adolescents and adult women
Source: PLoS One. 2019 Oct 24;14(10):e0223120. doi: 10.1371/journal.pone.0223120 (PMC6812786; doi:10.1371/journal.pone.0223120)
Supplement: S1 File — (PDF) [file pone.0223120.s002.pdf]

Text for Prenatal Health Study: Nutrition Knowledge and Behaviors Survey

How many meals do you typically eat during a day? ________

How many snacks do you eat each day? ________

Do you skip any meals on a regular basis? (Circle the answer that best describes your eating behavior)

a) Breakfast
b) Lunch
c) Dinner
d) I do not skip meals

Who cooks your meals at home?

a) Parent or guardian
b) Grandparent
c) Brother or sister
d) You
e) Other ________________________

How often do you eat at fast food restaurants during the week?

a) 1 time or less
b) 2-3 times
c) 4-6 times
d) Daily
e) More than once a day

Who does the grocery shopping in your home?

a) Parent or guardian
b) Grandparent
c) Brother or sister
d) You
e) Other ________________________

How much of a dinner plate should someone fill with fruits and vegetables?

a) 1/8
b) 1/4
c) 1/2
d) 3/4

What food group should you consume the least?

a) Meats and beans
b) Vegetables
c) Fruit
d) Fats, oils, sweets
e) Dairy
f) Bread, pasta, cereal, rice

Which of the following foods do you think contain fiber (check all that apply)?

a) Broccoli
b) Oatmeal
c) Steak
d) Yogurt

Which of the following foods is a low-fat source of dairy?

a) Whole milk
b) Ice cream
c) 2% milk
d) Regular cheese

What percent of daily calories should come from fat?

a) 30% or less
b) 30-50%
c) More than 50%

Which of the following foods are high in fat?

a) Soda pop
b) Fried chicken
c) Milk
d) Jelly beans
e) Baked potato
f) Bread

How much of the grains you eat should be whole grains?

a) 1/4
b) 1/2
c) 3/4
d) All grains should be whole

Which of the following foods has added sugar?

a) 100% orange juice
b) Soda pop
c) Bananas
d) White bread
e) Milk

Which color of fruits and vegetables is most healthy?

a) Red
b) Green
c) Yellow
d) Orange
e) Brown
f) White
g) All colors are important

TEKNO ID: _________________ Date: _____________ Visit Number: 1 ____ 2 ____
