# Supplementary material for: A social media intervention to improve nutrition knowledge and behaviors of low income, pregnant adolescents and adult women
Source: PLoS One. 2019 Oct 24;14(10):e0223120. doi: 10.1371/journal.pone.0223120 (PMC6812786; doi:10.1371/journal.pone.0223120)
Supplement: S2 File — (PDF) [file pone.0223120.s003.pdf]

Text for Prenatal Health Study: Electronic Data Usage Survey

1. How old are you? __________

2. Where do you access the internet?

   a. Home computer
   b. School
   c. Friend/Boyfriend/Relative's house
   d. Library
   e. Other: _____________________
   f. I do not have access to the internet

3. How often do you use the internet?

   a. Less than once per month?
   b. A few times per month
   c. Once per week
   d. A few times per week
   e. Daily
   f. I do not use the internet

4. Do you have a Facebook account? ____Yes ____No

5. Do you have an email account that you check weekly? ____Yes ____No

6. Do you own a personal cell phone? ____Yes ____No

7. Do you have a phone that sends and receives text messages? ____Yes ____No

8. Do you pay a fee for each text message received? ____Yes ____No

9. Does your phone have a data plan to access the internet? ____Yes ____No

10. If yes to #9, how do you use the internet on your phone (i.e. Facebook, Google search, Email, etc.)?

11. If yes to # 9, how often do you access the internet on your phone?

   a. Less than once per month?
   b. A few times per month
   c. Once per week
   d. A few times per week
   e. Daily
   f. I do not use / have internet on my phone

TEKNO ID:_____________ Date:____________
