# Supplementary material for: A social media intervention to improve nutrition knowledge and behaviors of low income, pregnant adolescents and adult women
Source: PLoS One. 2019 Oct 24;14(10):e0223120. doi: 10.1371/journal.pone.0223120 (PMC6812786; doi:10.1371/journal.pone.0223120)
Supplement: S3 File — (PDF) [file pone.0223120.s004.pdf]

Text for Prenatal Health Study: Pre-Intervention Interview Questions

I want to thank you for taking the time to meet with me today. My name is ______________________ and I would like to talk to you about your experiences while pregnant. Specifically, I want to find out what motivates you to make decisions related to nutrition and weight gain and how social media/internet may play a role in your decision-making process while you are pregnant.

The interview should take about an hour and a half. I will be taping the session because I don't want to miss any of your comments. Although, I will be taking some notes during the session I will not be able to write down everything we discuss so we both need to be sure to speak up so that the recorder doesn't miss our comments. All responses will be kept confidential which means that once your responses are typed, they will not be associated with your name. Only our research team will have access to your responses and your name will never be mentioned in any report or publications that come from this research.

Are there any questions about what I have just explained?
Are you willing to participate in this interview?

General Introduction and Media Access Questions

1. I am going to start by asking you questions to describe you and your pregnancy which will be important when we look at your answers later on.

    a. How old are you?

    b. How tall are you? About how much do you weigh?

    c. Is this your first pregnancy?

    d. Was your pregnancy planned?

    e. What hopes or goals do you have for your unborn child?

    f. What challenges do you face as you plan to be a mother?

    g. What do you think are important things you should be doing to make sure your baby is healthy?

2. Where or who do you ask for advice or information about your pregnancy?

    a. Do you ever use the internet for information?

        i. How do you gain access to the internet? Do you have a computer at home?

        ii. What types of information have you searched for?

        iii. Are there any sites that you have found helpful for information?

            1. How often do you visit these sites?

        iv. Do you use or have access to a cell phone?

            1. Do you own your own phone?

            2. How do you use your phone?

            3. Do you ever search for health information on your phone? If so, where do you search for information?

3. Have you joined any email, text message, twitter, blog, or facebook groups for additional information?

    a. What are they?

    b. What information did you get from these sources?

    c. How often do you receive information or search these sites for information?

4. Based on your experiences, is there a place or person that provides you with the best information on pregnancy?

Questions related to weight gain

5. Have you been given any advice about weight gain during pregnancy?

    a. If so, from whom?

    b. What was the advice?

    c. How did they tell you?

    d. Are you following this advice? Why or why not?

6. Do you know how much weight you are supposed to gain during pregnancy?

    a. How do you know?

    b. If you don’t know, what do you think about these? (show weight gain guidelines)

7. Have you been told how much weight you are supposed to gain during pregnancy?

    a. Do you think those recommendations are good for you? Why or why not?

8. Do you ever use the internet to learn about weight gain you might be experiencing?

    a. What information did you find?

    b. Where did you find this information?

    c. How helpful did you find the information?

    d. Do you feel the information was accurate? How do you know?

    e. Did you change your behaviors after finding this information?

Questions on diet during pregnancy

1. Who prepares the meals you eat?

2. Who goes shopping for the food to make those meals?

3. Do you help in making decisions about what groceries to buy?

4. How much money do you think your household spends on food each week?

5. Can you tell me how you have been eating during your pregnancy?

6. Have you made any modifications from what you did before you became pregnant? What specifically and why?

7. In terms of the quantity of food that you are eating, how does it compare to the amount you ate pre-pregnancy? (quantities per meal and number of times)

8. In terms of what foods you are eating, have you made any changes? What foods have changed and why? Probe about what foods might be eliminated and what specifically might be added.

9. Are you hungrier now that you are pregnant or do you seem to have less of an appetite? Why do you think this is the case?

10. Do you think a pregnant woman should be careful about what she eats? Why?

a. If yes, what have you heard that pregnant women should be careful about eating?

b. Are there any foods that are good or bad for a growing fetus?

c. Is there anything that you are avoiding or adding to your diet now that you are pregnant?

d. Does a pregnant woman’s diet need to change at different times during pregnancy?

11. Have you changed any of the ways that you prepare food since you have been pregnant?

f. For example, what about fried foods?

g. What about foods with salt or sugar?

h. How much dairy food are you consuming?

i. Have you been craving any non-food items? (PICA feeler)

j. What smells do you like more or less now that you are pregnant?

12. Are there any special foods or preparations or products that you are taking as diet supplements while you are pregnant?

a. Vitamin pills?

b. Foods that are fortified like a cereal product?

13. What advice have you received about what to eat during pregnancy?

a. Who offered this information / where did you learn about this recommendation?

b. Where else have you received information about eating for a healthy pregnancy?

14. What source has provided you with the most helpful information?

a. Why do you think this source gave the best information about having a healthy pregnancy?

15. Do you ever use the internet to find information about diet and nutrition?

a. What kind of information have you searched for?

b. What websites did you find most helpful?

c. How helpful did you find the information?

d. Do you feel the information was accurate? How do you know?

e. Did you change your behaviors after finding this information?

Questions on physical activity during pregnancy

1. Now let's talk about physical activity during pregnancy. Does a woman need to change her physical activity levels during pregnancy?

    a. Why do you feel the way you do?

2. What do you think the recommendations are for a pregnant woman?

    a. Have you heard of anything specific that you should or should not do?

    b. Has anyone tried to give you advice on how to exercise during pregnancy?

        i. Do you trust this advice?

        ii. Do you plan to follow this advice?

    c. If you wanted to know more about exercising during pregnancy, where or who would you go to for information?

3. Are you currently exercising?

    a. What kind of exercise do you enjoy most?

    b. How much have you exercised after becoming pregnant?

4. Have you changed your physical activity behaviors since you became pregnant?

    a. Why do you think your activity level has changed or not changed?

    b. How much did you exercise before becoming pregnancy?

    c. How much do you exercise now that you are pregnant?

    d. For those currently not exercising, what is the reason for not exercising?

        i. Do you want to exercise?

ii. What would motivate you to begin exercising?

5. What effects does your physical activity have on your developing baby?
    a. How do your current actions affect your baby?
    b. What do you think are the best exercises to do while pregnant?
    c. What activities should pregnant women avoid during pregnancy?

References used to create questions:

Tovar et al. Beliefs regarding the main contributors to pregnancy weight gain. *Matern Child Health J*. PMC 2012 February 13.

USAID. Guidance for Formative Research on Maternal Nutrition. USAID's Infant and Young Child Nutrition Project. February 2012.

Text for Prenatal Health Study: Post-Intervention Interview Questions

I want to thank you for taking the time to meet with me today. My name is ______________________ and I would like to talk to you about experiences while pregnant. Specifically, I want to find out what motivated you during your pregnancy to make decisions related to nutrition and weight gain and how receiving text messages and information of the Facebook site played a role in your decision-making process.

The interview should take about an hour and a half. I will be taping the session because I don't want to miss any of your comments. Although, I will be taking some notes during the session I will not be able to write down everything we discuss so we both need to be sure to speak up so that the recorder doesn't miss our comments. All responses will be kept confidential which means that once your responses are typed, they will not be associated with your name. Only our research team will have access to your responses and your name will never be mentioned in any report or publications that come from this research.

Are there any questions about what I have just explained?
Are you willing to participate in this interview?

General Introduction and Media Access Questions

1. It has been a while since we last talked, so I want to catch up on what has been happening. I am going to start by asking you questions to describe your pregnancy which will be important when we look at your answers later on.

    a. How has your pregnancy been going?

    b. What has been the best part of your pregnancy?

    c. Last time we talked, I asked you what hopes and dreams you had for your unborn child. How have these hopes and dreams changed?

2. Have you changed any of the ways that you prepare food since you have been pregnant?

    a. For example, what about fried foods?

    b. What about foods with salt or sugar?

    c. How much dairy food are you consuming?

    d. Have you been craving any non-food items? (PICA feeler)

    e. What smells do you like more or less now that you are pregnant?

    f. What challenges have you faced during your pregnancy?

        i. How did you overcome this challenge?

        ii. What helped you through the challenge?

    g. What things have you done throughout your pregnancy to make sure your baby is born healthy?

3. Where or who did you ask for advice or information about your pregnancy?

    a. Did you ever use the internet for information?

        i. How did you gain access to the internet? Did you have a computer at home?

        ii. What types of information did you search for most often?

        iii. Are there any sites that you thought were really helpful?

            1. How often did you visit these sites?

        iv. How often did you use the Facebook site that you joined at the beginning of your pregnancy?

            1. How helpful was this group?

            2. How often did you visit the site?

            3. What was your favorite part?

            4. What was your least favorite part?

            5. What motivated you to visit the site or kept you from logging in?

            6. Which information was most relevant to you?

            7. Did you change any of your behaviors after seeing something posted on the Facebook site?

            8. Did anyone disagree with the information you received?

            9. If you did not like the information on the Facebook site, where did you find better information?

            10. Did you change your view about weight gain during pregnancy after seeing the Facebook site?

a. Did you gain more or less weight during pregnancy because of information on the Facebook site?

11. Did you change any of your eating habits after seeing the Facebook site?

    a. What behaviors did you change?

    b. How did you change what you eat?

    c. How did your role change in preparing and shopping for food?

    d. How did the Facebook site affect how and when you take your prenatal vitamins?

12. Did you change your physical activity level after seeing the Facebook site?

    a. What behaviors did you change?

    b. How did you change your activity level?

    c. How did this make you feel?

    d. What specifically, motivated you to change your physical activity behaviors?

v. Did you have access to a cell phone during your pregnancy?

1. Did you have your own phone or were you borrowing / sharing a phone?

2. Did you read the text messages that were sent to you about pregnancy?

3. What text messages had the biggest effect on your pregnancy?

4. What messages were your favorites?

5. What messages were your least favorites?

6. Did any of the messages inspire you to change your behaviors during pregnancy?

    a. What behaviors did you change?

b. How did you change your behaviors?

7. How did these messages influence how you felt about weight gain during pregnancy?

   a. Did you gain more or less weight during pregnancy because of information in a text message? Why?

8. How did these messages influence how you felt about the foods you were eating?

   a. How did your diet change because of these messages?

   b. What foods did you add?

   c. What foods did you avoid?

   d. How did you change how you prepared foods?

   e. How did text messages change how you take your prenatal vitamins?

9. How did text messages change your physical activity behaviors?

   a. How did the text messages motivate you to change your behavior?

   b. What messages specifically influenced these changes?

   c. What affect do you think these changes will have on your baby?

10. Did you ever search for health information on your phone? If so, where did you search for information?

    a. What kind of information did you search for?

    b. How helpful is it to be able to look up health information on your phone?

    c. Who do you share this information with?

       i. Did you ever share this information with teens you met at RAMP or on the Facebook site?

ii. What did you share? Why?

4. Have you joined any other email, text message, twitter, blog, or Facebook groups for additional information?

    a. What were they?

    b. What information did you get from these methods?

    c. How often did you receive information or search these sites for information?

5. Based on your experiences, is there a place or person that provides you with the best information on pregnancy?

6. What is a good number of posts to add to a webpage like Facebook to improve your pregnancy health?

7. What is a good number of text messages to receive by phone for improved pregnancy health?

8. How often do you think a healthcare provider should send you information?

    a. What information is most helpful?

    b. What information motivates you to make healthy choices?

    c. Would you recommend using text messages for other teens in the future?

    d. Are text messages or Facebook a good way to reach teens that are pregnant?

    e. What other digital or mobile apps would be good for reaching pregnant teens?

References used to create questions:

Tovar et al. Beliefs regarding the main contributors to pregnancy weight gain. *Matern Child Health J*. PMC 2012 February 13.

USAID. Guidance for Formative Research on Maternal Nutrition. USAID's Infant and Young Child Nutrition Project. February 2012.
