# Supplementary material for: A social media intervention to improve nutrition knowledge and behaviors of low income, pregnant adolescents and adult women
Source: PLoS One. 2019 Oct 24;14(10):e0223120. doi: 10.1371/journal.pone.0223120 (PMC6812786; doi:10.1371/journal.pone.0223120)
Supplement: S6 File — (PDF) [file pone.0223120.s007.pdf]

S6 File Pre-Interview Transcripts

INTERVIEWER: So the first couple questions are just really basic are for me to get to know you and a little bit about your pregnancy. Uhm so the first question is how old are you?

ADL05: 24

INTERVIEWER: Okay. And how tall are you?

ADL05: 4’11”

INTERVIEWER: And about how much do you weigh?

ADL05: 130

INTERVIEWER: And is this your first pregnancy?

ADL05: No.

INTERVIEWER: Uhm, what number pregnancy is it?

ADL05: Uhm, uh four I believe.

INTERVIEWER: And is it a girl or a boy?

ADL05: Uhm I have… four of this?

INTERVIEWER: Well you can tell me about all of them.

ADL05: Well I have a daughter and this is, uhm I’m having a son so yeah.

INTERVIEWER: Okay. Uhm have you picked out a name yet?

ADL05: I was going to name him [Name Redacted].

INTERVIEWER: Ohh cute (laughs) I like that. And uhm this is one of the questions that you don’t have to answer if you don’t want to, but we’re just curious and gauging how many women are planning to become pregnant or uhm they became pregnant and decided to keep the baby. So would you define this pregnancy as a planned pregnancy?

ADL05: Yes.

INTERVIEWER: Okay and uhm so. Kind of thinking about the future for your baby, what things do you kind of think about or imagine… any goals or dreams that you have for your baby in the future?

1:34

ADL05: For now, I'm just thinking about him coming out healthy. That's all I really think of right now, as of right now. I didn't really think too far out to the future yet.

INTERVIEWER: Uhm have you thought what it would be like to have a little boy and how they would work out with your daughter?

2:00
ADL05: Yeah uhm… I don't know how it's going to be. That's why I don't know how it's going to be with a little boy, but I'm going to learn. And uhm I'm excited. She's excited so, she's going to be a good big sister and (mumbles) I'm not a boyish type person. I don't play football, I don't do none of that kind of stuff but, I'll learn.

INTERVIEWER: Is your daughter excited to have a little brother?

ADL05: Yes, she is.

INTERVIEWER: (overlap) aww, that's so cute. That's awesome. Uhm… so the next question is what challenges have you faced uhm as you plan to become a mother to a little boy?

ADL05: Uhm just knowing the difference is different from having a girl. Just how you'll be with a boy. So I'm just not sure how that is yet.

INTERVIEWER: Have you heard anything from friends or family about raising boys versus girls?

ADL05: Yeah, like that you have to be more harder on a boy and a girl they're like sensitive so you can't really, like holler at them. They'll cry. Something like that. A boy you have to be tough with. That's why.

INTERVIEWER: And what are some of the important things that you've been trying to do to have a healthy baby?

ADL05: Uhm just trying to stay stress free. Smiling. The thing is just to be happy.

INTERVIEWER: And are there any things that have been really challenging for you to maintain that stress-free environment? Things that kind of make it difficult?

ADL05: A lot of things changed, you know. Uhm I guess you can say that. (sighs) You know relationships and just life in general.

INTERVIEWER: And any challenges that you faced related to finding time for exercise or eating healthily? Things that maybe you did before you were pregnant?

ADL05: Yeah. I wouldn't work out that much. I tried to go to the gym but then I do, now that I'm pregnant I want to work out, but I can't really work out like that. Just waitin' to have the baby and lose the weight. Just have to lose the weight. That's all I can think about.

INTERVIEWER: Weight seems to be an important issue for women whether they're pregnant or not. Uhm so can you tell me a little bit more about how you've approached knowing that you have to gain weight to have a healthy baby but also not wanting to maybe gain too much?

4:47
ADL05: Uhm… uh… I just eat. I don't really—if I gain a lot, I gain a lot. I—I just… I always think I'm not going to get so big, you know. I don't—I didn't lose weight my first time? But you never know (laughs) I'm just… I don't really think about it. All I really worry about is, personally, my boobs because they be getting so big, and you can't do nothing about that so I'm just like so sad. (laughs) weight you can take off, but these… you can't lose them or something. It's like oh my goodness.

INTERVIEWER: (laughs) have you been given any advice about weight gain during pregnancy?

ADL05: No.

INTERVIEWER: Is it something that your midwife has ever talked to you about?

ADL05: Just that we have to gain at least 25 to 30 pounds. I don't really have a problem with that. Cause all that can just go away after I have (laughs)

INTERVIEWER: UHm and is that uhm… that amount of weight similar to things that you've seen other places or heard from other people?

ADL05: No.

INTERVIEWER: Or maybe just only talked to about that with your midwife?

6:17
ADL05: Yeah.

INTERVIEWER: Okay. And is that something that you feel comfortable asking questions about with… at one of your regular checkups and I know they weigh you at every checkup so has that been helpful with gauging whether you're in that range?

ADL05: Yeah.

INTERVIEWER: Okay. Have you ever felt worried that you weren't going to gain enough or that you were going to gain too much?

ADL05: I was thinking I wasn't gaining enough at first, but I see that, I felt like it was my coat and everything I had on at my last visit, but I guess not cause I took everything off this time and

like, I was the same kind of (laughs) So I gained a lot I guess. But at first I was only gaining like one pound and I was supposed to gain more but I think I'm doing good now. I'm excited?? So.

INTERVIEWER: That's good. Uhm so you've mentioned some concerns about losing the weight after uhm you deliver. Uhm this pregnancy has it been different for weight gain compared to your previous pregnancy?

7:31
ADL05: Uhm, well cause I never lost kind of the weight from my first, uhm daughter so my stomach always had a little pudge after. So the first pregnancy was like uhm… I didn't really gain a lot til like the end. The last eight, nine months I wasn't big. So like the whole pregnancy, nobody know that I was pregnant until I got like seven, eight months it was like what? So, I still look small though. I'm about six months and I still look small so I'm not really worried about anything because I'm still small so as long as the baby is growing good uhm… huh… I don't know. Weight is not really an issue to me because I know that it can come off. Really.

INTERVIEWER: Mhm. Uhm, have you ever searched for anything about weight gain on the Internet?

ADL05: No.

INTERVIEWER: Okay. Uhm let's see. So let's talk a little bit about uhm… food. So what was… how has your diet changed from when it was before pregnancy compared to now?

ADL05: Uhm… I believe I probably eat more before because I don't even much now because of heart burn. That's the only reason why I won't eat as much as I would have eaten.

INTERVIEWER: Is heartburn something that's been bothering you since the beginning of pregnancy or is it more recent?

9:19
ADL05: It's been since the beginning of pregnancies. This one is a (???) with heartburn.

INTERVIEWER: Have you talked to anyone about foods that are better to eat with heartburn?

ADL05: I look it up on the Internet and stuff. Uhm. But you know I'm going to eat what I wanna eat so (laughs)

INTERVIEWER: What foods did you see though that might be helpful for heartburn?

ADL05: Uhm I didn't really see what was going to be helpful. I just seen the stuff that you should stay away from like citrus and all that type of stuff and I know usually red things you know if I eat sauce and any type of red popsicle or anything like red juice. I like red juice and everything but it always gives me heartburn. But I still… I still eat it. I still do. I still like (laughs)

INTERVIEWER: I do the same thing, especially if you're not feeling well. You're like well, I'm going to get sick. I might as well enjoy what I'm going to eat (laughs) So, who prepares the meals that you eat?

ADL05: I do.

INTERVIEWER: And do you do all the shopping for groceries?

ADL05: Yes.

INTERVIEWER: Uhm does anyone else help in making any of the grocery decisions?

ADL05: Uhm sometimes my boyfriend, maybe.

10:48
INTERVIEWER: Okay. And does he go to the store with you or just offers advice on what things to get?

ADL05: Sometimes he comes with me.

INTERVIEWER: UHm and on average, how much do you think you spend each week on groceries?

ADL05: Uhm. Probably 50?

INTERVIEWER: Okay. Uhm okay so you mentioned that you've been having heartburn. Has anything else changed about your diet? So foods that you've added or removed for reasons other than heartburn?

ADL05: Uhm I added more apples cause it's just an easy snack. Like grapes. I'll keep some out—I'll keep grapes uhm by my bed, just in case I get hungry. And (mumbles)

INTERVIEWER: Have you heard about any foods that are really good for pregnancy or really bad for pregnancy?

ADL05: I heard that peanuts are good.

INTERVIEWER: Peanuts ARE good?

ADL05: Yeah.

INTERVIEWER: Okay.

ADL05: But I don't eat peanuts. So that's why (laughs)

INTERVIEWER: And any other foods like, sometimes I know they call them like superfoods and uhm, they can boost baby's brain development or things like that? Have you ever seen any articles about foods like that?

ADL05: No, but I seen—I read about the foods you shouldn't eat. Like the peaches if it's not organic and uhm because they call it the dirty dozen or something. Or something like cause… I know it's on my app. The pregnancy app says make you sure you buy like the peaches and grapes that are organic because they're dirty or something.

13:03
INTERVIEWER: So have you tried to buy organic grapes and apples then because of that advice?

ADL05: I've tried. But…

INTERVIEWER: Do they taste different?

ADL05: I bought something that tasted different. It was… it was organic chicken and it was so disgusting so I just… I wasn't following what they say, no. (laughs) So I didn't listen no more. I'm like, no.

INTERVIEWER: (laughs) Uhm anything else that uhm you've gotten a messages about that recommended eating something or not eating something? Maybe about vegetables or certain types of meat?

ADL05: Not that I can recall.

INTERVIEWER: Uhm let's see. So you mentioned that you heard about the dirty dozen from an app. Do you know what app that is?

ADL05: It's Pregnancy app. Uhm… I think it's just called pregnancy app.

INTERVIEWER: Okay. And what kind of messages does that app usually send?

14:20
ADL05: Uhm… it just says like, you know, like what your baby should be doing at a certain time. Like, during pregnancy and how your body should feel. And the things you should eat. It says stuff like that. Like yogurt, you should eat yogurt.

INTERVIEWER: And how often do you get updates from that app?

ADL05: Once a week.

INTERVIEWER: Have you joined any other apps like uhm What to Expect? And I think there's one… uhm maybe called Baby Bump?

ADL05: I think I tried What to Expect.

INTERVIEWER: You didn’t like it?

ADL05: Uhm I think it needs Internet every time you go on it, and I don’t have Internet on my phone. I use go by Wi-Fi. So as long as it has Internet, I can’t see the updates.

INTERVIEWER: Okay. Gotcha. So the pregnancy one that you have, is it like a text message then?

ADL05: Yeah, like it appears. Like a notification will come… when your new week starts.

INTERVIEWER: Have you joined any other sorts of programs like I know there’s uhm Baby Bump does a weekly e-mail and then there’s Twitter groups… uh Facebook groups. Anything else you can think of?

ADL05: No.

INTERVIEWER: Okay. Do you think that overall a pregnant woman has to be really careful about what she eats during pregnancy?

ADL05: Yes. I did hear about that. I know you have to make sure your meat is cooked, y’know… through. Not pink. I think I got sick recently cause I was at Alby’s and they had a little pink in it. And after I was through my stomach was killing me. So I was like okay, that’s why your doctor said make sure you eat food at home and cook your own food! I was like okay. But then I eat Alby’s and I had my worst stomachache ever.

INTERVIEWER: Uhm anything else you heard that is not good for developing baby?

16:46
ADL05: Uhm just, you know the… moltrine? And ibuprofen, stuff like that. Medication…

INTERVIEWER: I know we’ve talked a little bit about some things you’ve added, like the apples and the grapes, but is there anything that you’ve been avoiding now that you’re pregnant because it either bothers you or you feel it’s not as good of a choice to be eating?

ADL05: No (mumbles????) the first three months I wouldn’t eat a lot of stuff because I didn’t feel good but now I can eat anything.

INTERVIEWER: Did you have morning sickness?

ADL05: Uhm, not really, I just… I wouldn’t throw up or anything like that. I just, I just had this weird feeling all the time.

INTERVIEWER: Uhm and what things were you avoiding?

ADL05: I would really avoid meat as meat was like… cause I had to cook my own food and cooking the food makes me feel like not good, so I wouldn't really eat meat or anything like that. And… I would really just eat fruit and like uhm cucumbers, and salad. And like the healthy stuff. That's all I wanted in the beginning.

INTERVIEWER: When did that change?

ADL05: When I got four months (laughs)

INTERVIEWER: And then what sorts of things did you start eating?

18:33

ADL05: I started eating everything. I didn't have no feeling towards nothing. Like I could eat whatever was cooked if I wanted, but before I couldn't. I just couldn't eat anything.

INTERVIEWER: Have you had any cravings for certain foods?

ADL05: Uhm I was having a craving for banana pudding.

INTERVIEWER: Was it a regular craving or one that just popped up and once you had it, you felt like, "Oh. I'm good now."

ADL05: Uhm it was a craving I had for about two weeks and I finally had to make it for me to not have that craving anymore. (laughs) Like I don't really like banana pudding. That's why I was so shocked by it.

INTERVIEWER: (laughs) Have you changed the way you prepare food at all? So maybe uhm frying versus sautéing, or uhm baking. Anything like that?

ADL05: Uhm I don't really fry anymore like that. The reason why I don't think so is I just don't usually fry like I would usually fry my chicken like that. I haven't had any fried chicken. I haven't fried in so long. I don't think since this whole pregnancy I don't think I have. I don't think I've eaten fried chicken. But I don't know why, though.

20:04
INTERVIEWER: So you haven't fried foods since before this pregnancy started?

ADL05: No, I used to always like stuff that I was so used to, I don't want that anymore. Something like I would always eat fried chicken, I would always cook fried chicken.

INTERVIEWER: Ohhh so you did before, but you're not really interested in eating it now?

ADL05: Yeah.

INTERVIEWER: Okay. So what method of cooking do you usually use to cook meats?

ADL05: I usually eat like, if I'm going to eat chicken, I'll bake chicken. I usually, yeah. Or… if I want chicken, sauté. Stuff like that.

INTERVIEWER: And, uhm, for veggies, how do you usually cook your veggies?

ADL05: Uhm, I usually boil them in butter. Or I guess canned, I'll just put iton the stove and (mumbles)

INTERVIEWER: And what do you normally season veggies with?

ADL05: Uhm salt and pepper.

INTERVIEWER: Are spices something that you've heard about during pregnancy that you should change spices that you eat or don't eat?

ADL05: No

INTERVIEWER: Okay. And if I tell you… I'm going to tell you a food item. I want you to tell me the first thing that pops into your head. Uhm in relation to pregnancy. So what you've heard, or what you personally think. Salt.

ADL05: Salt… I would think would make (laughs) would be not good or it would make you not gain weight, but… I don't know. I think of salt would make your feet fat (laughs) I don't know why; that just popped into my head (laughs) (mumbles)

INTERVIEWER: No, that's perfect. Thank you. And what are your—what's the first thing you think about when someone says sugar?

22:23
ADL05: Sugar… I think about uhm, diabetes. That's just what pops in my head.

INTERVIEWER: Nope, no answer's wrong. That's perfect. And is diabetes something that you've been concerned about during this pregnancy?

ADL05: No.

INTERVIEWER: And what about dairy foods? What are the things the first things that pop into your head about those?

ADL05: Uhm… it sounds nasty but I don't drink milk, but I like orange juice. And yogurt sometimes, uhm. Yeah I just think about milk and (mumbles)

INTERVIEWER: Is that something you haven't liked even from before pregnancy?

ADL05: Mm yeah. I never drank milk. I would think since I was a baby, probably. I don't know what's wrong with me and milk.

INTERVIEWER: Uhm, what about cheese? Do you eat cheese and things?

ADL05: Yeah. I like cheese.

INTERVIEWER: Okay. And is that something, the amount of cheese that you eat, has changed at all this pregnancy?

ADL05: No

INTERVIEWER: Okay. Uhm and we already talked about smells and your diet record, so just the baby powder was the smell that you like?

ADL05: Yes

INTERVIEWER: Uhm, let's see. Are you taking any vitamin pills at all?

ADL05: Uhm, I'm supposed to take the prenatal. Uhm I don't really take them though.

INTERVIEWER: How come?

ADL05: They're nasty. And I can't—I can't… pills. I can't do pills. That's why I have chewables and they just so disgusting. It's hard for me to swallow pills.

INTERVIEWER: Uhm, so you said you don't really take them? Do you take them occasionally?

ADL05: No. I tried them and I just couldn't do it. I should… my first pregnancy, I never took them either. They were so big, and I just couldn't do them either. So I just—and nothing happened this—that pregnancy, and I'm thinking like (laughs)

INTERVIEWER: That's okay. Uhm, do you eat cereal at all?

ADL05: Yes.

INTERVIEWER: What kind of cereal?

ADL05: Uhm I like Frosted Flakes.

INTERVIEWER: Uhm anything else?

ADL05: Uhm Cap'n Crunch

INTERVIEWER: Are those cereals you've been eating during this pregnancy?

ADL05: Yes.

INTERVIEWER: So those are uhm, those can be referred to something called fortified foods, so they have a lot of vitamins and minerals added, so it's kind of like getting a vitamin. Uhm, I don't know if you knew that or not. Do you ever look at the food labels on the boxes that have all the lists of nutrients?

ADL05: Uhm, not for the cereal. I never did for cereal.

INTERVIEWER: Have you looked at those on other boxes? What—what things are you looking for when you look at those labels?

ADL05: I usually look for the juice, like 100% juice. I don't notice like the 100% like vitamins. I like the 100% juice. That's what I usually go for. Yeah. I don't know what else, really.

INTERVIEWER: See, you're doing good. Lots of vitamins in there. Let's see. Have you received any advice from family or friends about what you should or shouldn't eat during pregnancy?

ADL05: Not really, they don't. No.

INTERVIEWER: Do you talk to your family much about experiences with your pregnancy? Doesn't have to be food-related. Just anything.

ADL05: Uhm, not really.

INTERVIEWER: Okay. Uhm… and do you have any close friends who have also had children?

26:53
ADL05: Uhm, my friends don't have children, but I have one friend who's not that close, but a friend. She has a child.

INTERVIEWER: Have you ever swapped stories about experiences or things that you've tried while pregnant?

ADL05: Uhm yeah, we talk somewhat. She has a littler baby so she don't really know too much. My-my child, she's five, so. We don't really have that much in common. She had a boy, so. (laughs) It's different. So we don't really, I don't know. We don't really swap stories like that; no.

INTERVIEWER: Okay. Uhm and have you ever searched the Internet for information about food or other things during pregnancy?

ADL05: Yeah, I search a lot about pregnancy on the internet.

INTERVIEWER: What kinds of things do you look for?

ADL05: Uhm, let’s see. I would search before probably for; I know I searched about the boob situation. I searched about that. And uhm, probably I guess the heartburn and… I can’t remember. I haven’t searched recently, but I’m not sure.

INTERVIEWER: When you searched the web, how do you search? Do you use Google?

ADL05: Yeah, I go to Google.

INTERVIEWER: And so let’s say you type something into Google and it gives you that whole big list of sites and different things and… how do you decide which links to click?

ADL05: I usually go to the first one—the most… that’s just the first one that pops. Or I usually go to all of them that’s on the first page.

INTERVIEWER: Okay. And… in your searches, are there some websites that you recognize from previous searches that keep popping up as options in the list?

29:24

ADL05: BabyCenter I think has (mumbles) one that’s always there.

INTERVIEWER: And have you ever thought that some of the information that you got from a site was not true or unhelpful?

ADL05: Yeah, cause you know everyone has their opinions on the sites or… something like that. So I don’t really like, pay attention. I don’t really like take it to heart, this got to be true. I don’t believe it cause they’re not doctors. If it is a doctor, I don’t really know if they’re a real doctor. So I just look and just… just examine stuff, really.

INTERVIEWER: So it sounds like you kind of screen and make up a decision about whether it’s a good source. And there any sites that you know are good sources and you can always trust what they have?

ADL05: Mm I like BabyCenter a lot, I do. But it’s like everybody’s talking like what they been through and stuff like that. It’s like that. But I’m not sure if. I don’t know who runs the site. I don’t know who posts the stuff up, so I don’t know.

INTERVIEWER: So it sounds like a lot of the things that you found on BabyCenter are like forums where other moms can post their experiences. Uhm have you ever looked at any of the articles that are posted where it’s not written by a pregnant mom but it’s written by someone who works for BabyCenter?

ADL05: Yeah, I probably have.

INTERVIEWER: Okay.

ADL05: I’m not sure.

INTERVIEWER: Uhm, and are there any other sites that you can think of that you’ve found helpful in your searches? Like maybe when you searched for heartburn?

ADL05: Uhm, no. I can’t think of any.

INTERVIEWER: Okay. Have you ever used the Internet to look up information about uhm food specifically? Whether it be a recipe or uhm what’s safe, what’s not safe during pregnancy?

31:34

ADL05: Probably recipes.

INTERVIEWER: And were those during this pregnancy when you were looking up recipes?

ADL05: Yeah. I looked up banana pudding.

INTERVIEWER: So you made banana pudding from scratch?

ADL05: Yeah

INTERVIEWER: Whoaaa, that’s awesome. (laughs) any other recipes?

ADL05: Uhm… I’m not sure. I probably didn’t make it, though. I know I was looking up how to make empanadas. I don’t really make it, though.

INTERVIEWER: And was that something you had been craving, or…?

ADL05: Yeah.

INTERVIEWER: Okay. Is that… a craving that usually prompts you to do a search for a recipe?

ADL05: Yeah.

INTERVIEWER: Okay. Okay, so let’s change gears and talk a little bit about physical activity or exercise, which ever word you prefer. Uhm, so I know that you mentioned that you didn’t have a lot of time before pregnancy to get to the gym and work out. So, but how has your physical activity level changed from before pregnancy to now? Would you say it’s the same, or different?

33:10
ADL05: It’s the same, I guess. I don’t really work out, and uhm I didn’t really then, either. So…

INTERVIEWER: And all the messages that you've received from your pregnancy app, your doctor, uhm… acquaintances that you may have met, has anyone told you anything about the importance of exercise or uhm.

ADL05: Yeah, like the app would say about the kegel exercise, yknow, to keep the uhm, to keep that part tight for birth… when you're having birth, stuff like that. And you should walk, keep your energy up, stuff like that. Cause I'll be really out of breath so that's why I need to walk and do stuff but… I should, but I don't.

INTERVIEWER: What things are keeping you from being able to get out there and do stuff?

ADL05: I don't know. I… I just need to prioritize and find the time to really be focused and say okay. Maybe it's because it was cold, I don't know. I'm not going to walk in the cold (laughs) Maybe that was it. It's getting warmer, so I might go to the park and walk on the trail. I haven't done that before.

34:46

INTERVIEWER: It must be more difficult to find time for yourself, too, when you have another little one at home—

ADL05: Yeah

INTERVIEWER: So how do you work around schedules with her and then also trying to fit in time for you?

ADL05: That was a difficult task for a long time, but you know now, before I was working in a school and all that, but now I have my own y'know registered day care so I can be home. And I can watch children and be with my daughter, so now it's way easier, you know. And… go to school online instead of going to class, you know. So… I just had to make ways to be with her and to not neglect her. Time for myself? I guess when she's at school, like now. That's my time.

INTERVIEWER: Okay, can I just make sure that I understood what you said? So right now you're doing school online?

ADL05: Well, right now's the semester off, though. But when… last year, I had to go online. Yeah.

INTERVIEWER: And you said something about a registered daycare? So you are watching your daughter as well as other children?

ADL05: Yeah.

INTERVIEWER: Okay. That's awesome.

ADL05: Thanks

INTERVIEWER: And so, it seems having a little daycare program at home, that could be good. Chasing little kids around for exercise (laughs) so yeah, don't sell yourself short. You're doing stuff. Uhm, so what kinds of activities were you doing with them?

ADL05: Well its been cold recently, so I'm, I'm not going to take them outside when it's really cold. We only have one in my car right now, cause I need a bigger car so I can't—I'm like oh my goodness, I'm going to have another child so it fit the kids. I don't know. This is too much. But it's another girl, so they just like to play baby dolls, stuff like that. So we don't really do too much. We like to dance, stuff like that, so that's about it, really. But it's getting warm so we'll be going to the park. We'll be doing that.

INTERVIEWER: That'll be fun. And you said they like to dance. Do you ever dance with them?

ADL05: Yeah.

INTERVIEWER: I'm not very coordinated, so my dancing usually looks like a chicken trying to escape the farm (laughs) I envy you. Uhm… so has your midwife ever talked to you about exercising during pregnancy? Things you should or shouldn't do?

ADL05: No… we don't talk about it.

INTERVIEWER: And on the app that you have, have you ever received any messages about things that are safe to do or not safe to do?

ADL05: Yeah, I know it said about swimming, you should go swimming. That's the best thing to do when you're pregnant, stuff like that. And it said that don't like weights, don't do anything like that. Any type of exercise that's like that, so.

INTERVIEWER: And if you could go to the gym, what things would you prefer to do?

ADL05: Treadmill… I like the treadmill though. And I do like to go swimming when I do. I have to get a membership, too. I don't have one. I got to get that, too. But I don't know if I want to go into the swimming pool right now. I'll just—I don't know if I could do it.

INTERVIEWER: What do you mean?

ADL05: I don't know. Just big ol' stomach going into the pool, I just don't know (laughs) I don't know. Unless they have the pregnancy class with all pregnant women, then I'll feel comfortable, but (laughs)

INTERVIEWER: Uhm let's see. So I know you said you're not doing a ton right now, but what things are you doing? Are you getting any walking in? Or maybe like you mentioned some dancing with your daughter? Chasing her down for bedroom? All that stuff counts. So think through a typical day and tell me what kinds of things you do where you're just moving around. It could even be just standing, cause standing could make a big difference.

ADL05: Okay, what do I do? When I braid hair, I stand. I just braided hair yesterday, and I was standing and my feet were killing me (laughs) I think me wearing heels, that's a job, and I was just wearing heels yesterday (laughs) So… what else? I know my boyfriend has a son, and he's one, so chasing him around, oh my goodness. I was sweating. My whole—everything was sweating. I'm like oh my goodness. I didn't know what I do. I just know—that was exercise. I mean, when I say exercise, I was out of breath. Like (laughs) I just followed him around. He's so active. I was like oh my goodness.

INTERVIEWER: Practice for your boy.

ADL05: Right, oh my goodness. Little boys. Whooo. Active.

INTERVIEWER: And can you tell me one more time what your boyfriend was doing when you were chasing him around?

ADL05: Well, he was gone. He was like can you watch him really quick? He left, then I was like… he was going everywhere. I was like ohhh my goodness. Oh my goodness. He went on and on, he didn't want to sit down. He didn't want to watch TV. Oh my goodness, Jesus. Whooo

40:45

INTERVIEWER: And uhm, uhm are these activities that you just mentioned similar to things you were doing before pregnancy as well, like braiding hair, walking around in heels, chasing the boyfriend around?

ADL05: Yeah (laughs)

INTERVIEWER: Do you have Internet at home?

ADL05: Something like that. Yeah, I have a spot of Internet, you could say. I just put my phone right there, and there's Internet.

INTERVIEWER: Oh, okay. And is that how you search for things?

ADL05: Yeah

INTERVIEWER: Do you have a computer at home or do you use your?

ADL05: I have a computer, but it's—in that spot, there's no Internet. So… I don't (??) get Internet in the whole house. I have a random computer with no Internet on it. I don't know.

INTERVIEWER: That's okay. Do you use your phone mostly to do searching?

ADL05: Yeah

INTERVIEWER: Do you ever search for YouTube videos?

ADL05: Mhm.

INTERVIEWER: Uhm, would you… have you ever thought about looking for like short little exercise videos on YouTube that you could do in your house?

ADL05: Uhm, I haven't really thought about it.

INTERVIEWER: Is that something that uhm, if someone sent something to you, that you would be interested in trying, or not so much?

ADL05: Probably. I… maybe more of a relaxation type of, you know, video I would rather do. I don't know why I don't want to work out right now not 'til I'm done. You know like I haven't been working out because I'm just going to be getting bigger anyways. I'd rather work out after. That's like my mindset or something like… I just want to wait. That's like the first day I'm going to the gym. Hopefully I really stay like this and, I want to get my body right. I do.

INTERVIEWER: Uhm, what things would you say motivate you to start those sorts of activities? Like after pregnancy, what would motivate you to go to the gym?

ADL05: I'm just seeing… my stomach go away. That's probably just going to motivate me (laughs) Like oh my goodness, whoa… I can't fit in any clothes, like that's going to motivate me. Really, probably.

INTERVIEWER: And do you have anyone that would go to the gym with you? Like a friend that'd keep you motivated?

43:13
ADL05: Yeah. My sisters, and my mom. They would come.

INTERVIEWER: And have you heard of any positive benefits of exercise for developing babies?

ADL05: No

INTERVIEWER: And I know you mentioned the Kegels and also the uhm, you have more energy when you exercise. But have you heard of any additional benefits that you might not have thought of earlier for the mom, or for you?

ADL05: Mmm no, I'm not sure.

INTERVIEWER: Okay. And… do you think that your current activity level and current diet have any sorts of effects on your baby?

ADL05: Uhm... I don't think so.

INTERVIEWER: Uhm, I think that’s pretty much everything, actually. Are there any questions that you maybe thought of an answer to from earlier in our conversation that you want to add quickly or maybe discuss further?

ADL05: Uhm… not that I could think of.

INTERVIEWER: Okay. So for this study, you’re going to be receiving messages from Facebook as well as text message. And uhm I just had a few questions about that. Does your cellphone receive picture text messages?

ADL05: Mhm

INTERVIEWER: And does it cost you anything for you to receive text messages?

ADL05: No.

INTERVIEWER: Okay. And are there any things that you had thought about… let me start over. So I know that you enrolled in the study and know that you’re going to receive health information. Uhm when you heard about that, what kinds of things did you think you would be receiving?

ADL05: Uhm… about health?

INTERVIEWER: Mhm.

ADL05: I’m not sure. Probably just, you know, what’s happening during pregnancy. Normal stuff. Yeah.

INTERVIEWER: Are there any things that you are most interested in learning about that maybe you didn’t know a lot, and you weren’t sure where to look for the information?

ADL05: Uhm… not really.

INTERVIEWER: Okay.

End 46:36

ADL06

(Skype Ringtone)

ADL06: Hi!

Interviewer: Hi, hello, how are you?

ADL06: I'm doing okay, how are you Interviewer?

Interviewer: I'm good.

ADL06: can you see me?

Interviewer: I cannot…

ADL06: okay let me adjust, I have a back camera, 'cause I have an iphone…

Interviewer: oh, okay…

ADL06: um…

Interviewer: oh! There you are.

ADL06: let me move so the sun glare isn't blinding us…

Interviewer: (laughs)

ADL06: I hope you don't mind I didn't get out of my pajamas…(laughs)

Interviewer: oh, I can't lie, me too…(laughs)…so we'll be pajama party interview today…(laughs)…um…so hopefully this won't take more than an hour, um, normally it's a little bit shorter but it depends on the, the person who's interviewing and how talkative they are, so …

ADL06: okay

Interviewer: um, so the goal is to just really get a gauge for what sort of, um, things that you might have changed about what you're eating and how you're exercising, um, once you found out that you're pregnant, and how those things differed from before, your attitude or beliefs about eating certain foods changed because you thought they were healthier, or less healthier for the baby, um and then also what resources you that used to look for information about pregnancy, so, um, specifically internet resources or cell phone resources like apps, or facebook, those sorts of things…

ADL06: okay

Interviewer: okay. um, so, just a quick little disclaimer, anything that you say is confidential, so, um we do record all the conversations, just so that we can remember exactly what everyone said, and then we type them out and…(audio problems)…we remove your name um so it will just appear as your subject ID and then um if we were to ever publish any results from this research your name will never appear in any papers or anything like that, um, we would use quotes, or, for general themes among all the women in the study, um, and then use the quotes to kind of support the themes that we've identified…

ADL06: okay

Interviewer: um, so if that all sounds good then we can go ahead and get started if you don't have any questions…

ADL06: I do not

Interviewer: okay, awesome! Um, so I like to just start out with sort of a general introduction to, kind of characterize your pregnancy and get to know a little bit more about a, like, what your hopes and dreams are for the baby…

ADL06: okay

Interviewer: so these first couple questions are kind of demographic, so the first is: "how old are you?"

ADL06: I'm 33

Interviewer: okay

ADL06: I keep saying that, I'll be 33 this summer in like 8 weeks…(laughs)

Interviewer: okay, I feel like once you get pass like through twenties you're like "oh, I…(laughs)…don't pay attention to how old I am anymore…(laughs)"

ADL06: um

Interviewer: and how tall are you?

ADL06: 5 foot 8

Interviewer: and how much do you weigh?

ADL06: yesterday at the midwife I weighed 204

Interviewer: okay, and is this your first pregnancy?

ADL06: it is not my first pregnancy, but it's my first pregnancy that's gone this far, I had previous miscarriages…

Interviewer: okay…and would you define this pregnancy as being planned?

ADL06: yes.

Interviewer: okay, and this is always kind of the doozy of a question, so, um, you don't have to have a profound answer or anything but, um, I think that most people…(audio is distorted)…like to think what it would be like in the future once the baby's here, and even like further into the future when they're grown up, um, so what hopes and goals do you have for your baby?

ADL06: you know I've actually given this a lot of thought, um, you know my hopes for our baby is that he or she can be who ever they are meant to be…(audio distorted)…being themselves and that my husband and I can, kind of, be a safe place for our child to…just develop into who he or she is meant to be…so, an, and I think my, my dreams and goals for the baby would be like many people, um, you know to get a good education and be successful, and you know potentially more successful than we are, and maybe struggle less than we do…(Interviewer laughs)…these sorts of things…

Interviewer: those are good goals

ADL06: thank you

Interviewer: um, and…what do you think are the important things any pregnant mother should be doing to ensure the health of their baby?

ADL06: um, that's a huge question, I…I think that you don't have to have a planned pregnancy to have a happy and successful and safe pregnancy but I think that putting your baby first in all of your major decisions, um, is a key component to a successful pregnancy, and I think um, no whether is nutrition, lifestyle, um, you know dealing with stressors, sleep, everything you just have to think of baby first…

Interviewer: um, so since this is not your first time being pregnant, I'm sure in the past you've looked up information, so when I ask a lot of the questions feel free to think back to any of the pregnancies, 'cause I mean sometime resources that you use might change over time depending on what's available, so um, think broadly I guess, um, so do you ever use the internet to look up information?

ADL06: I do

Interviewer: okay, um and how do you typically gain access to the Internet

ADL06: um…I have a smartphone, but I also have, um, just Internet access at home on my laptop…(voice in the background)…so it depends on where I am.

Interviewer: um what types of information do you recall looking up?

ADL06: um, I recall looking up things about food safety, what foods were and were not safe in pregnancy…or medications, um what was and wasn't safe to take especially in the 1st trimester…

Interviewer: mhm

ADL06: and also because, and I'm not sure if I shared this with you or, or with any of the other ladies, my husband and I did really extensive fertility treatment to get pregnant and so I also use the internet to kind of get tips and tricks from other women who've been through infertility procedures, um sort of in those early stages like you don't even know if you're pregnant yet, you know, some of those…you know if, if you pee on a stick after seven days and it's not positive, you know that kind of stuff…

Interviewer: uh huh…(agrees)

ADL06: which is not necessarily pregnancy related but more conception, you know, and all of that…(coughs) excuse me

Interviewer: and are there any specific sites that you've found to be most helpful?

ADL06: um, well I'm a nurse so I tend not to go to you know like the "bump.com," or something I'm looking for you know an NIH article, or something that has a study associated with it so I can't say that there's one website that I look to, um, in particular…

Interviewer: okay, um, do most of your searches then maybe are with a search engine like google or yahoo?

ADL06: yeah definitely, we're kind of a google family, so…(laughs)…that's, that's our first step is to, to google it, you know, that's become a new, a new voca, part of our vocabulary, to google it

Interviewer:…(laughs)…um so when you google something, do you have a method or a process of weeding through all the results that you get and how will you decide which source may be a reliable source or a not reliable source.

ADL06: well, um, so I married a computer engineer who kind of taught me some rules about internet search, like don't worry about words like the and is just kind of use your main nouns or verbs…

Interviewer: ehm ehm…(agrees)

ADL06: and um you know in terms searching down a list, you know, I'm looking for a website that ends in .edu or.gov, you know, something that might have a more reliable source than a .com…

Interviewer: okay

(skype ringtones)

ADL06: sorry about that I just got a phone call I had to hit decline

Interviewer: oh, that's okay

ADL06: actually that was probably the midwives' office calling about my three hour glucose tolerance test which I had yesterday…

Interviewer: oh…well if you want to take that we can always…

ADL06: no, it's totally fine, I'll get it later, I think it was negative, so…

Interviewer: well that's good, I hope it was…

ADL06: me too

Interviewer: um, let's see, so how often do you think that you search the internet for pregnancy related information?

ADL06: um, probably I would say like in the early stages um it would have been maybe a couple times a week, now at this point, at 30 weeks, maybe once a week something would come to mind and I'll wonder about it, um, for example the other day, actually last week, I started having some pretty significant rib pain, which I thought was related to my bronchitis and you know, the more I waited and it kind of still stuck around I ended up kind of looking online for some information and finding out yeah it's pretty common to have rib pain as you move into your third trimester, and so, you know, I was looking for resources about some stretches or exercises that might help, so something like that, so it's not as frequent as it was in the beginning

Interviewer: okay

ADL06:…(coughs)…excuse me

Interviewer: and you mentioned that you use your phone as well for internet, have you downloaded any pregnancy apps at all?

ADL06: I do have a pregnancy app, um, I can look at what it's called but I'll have like kinda close down skype for a sec because I don't know what it's called…

Interviewer: okay

ADL06: um, but the idea behind it is more, it's more like a countdown, you know, like how many days you've been pregnant and how many days you have left, and it does give a little tip of

the day, um most of which is information I already knew, um but it also kind of gives you a weekly reminder of how big the baby is, and um, you know what developmentally the baby is doing this week…

Interviewer: mhm…(agrees)

ADL06: can you see me because it looked like my picture froze?

Interviewer: yeah it did, but I can still hear you fine, so that's…

ADL06: alright…

Interviewer: that's okay…(laughs)

ADL06: do you need the name of it?

Interviewer: um, if you have it I'm more than happy and excited to know what it is, but if not that's okay too…

ADL06: alright let me take a look…hmm…I think it's just called "iPregnancy"

Interviewer: ok

ADL06: and, um, it it kind of, I think it must have a a British publisher because some of the language seems more like European, in terms of how they speak or how they write on there, um, and their spelling is, I think, more European with the ae use…

Interviewer: oh…okay

ADL06: um, I mean I, it's not the best app but the most interesting thing for me was the, the countdown, which is helpful when you get you know to the uncomfortable stage and you're saying "only 70 days left!" you know that kind of thing, um, let me try my camera again, (clears throat) okay looks like my camera doesn't want to work, I'm sorry…

Interviewer: that's okay, no worries...um, so, so that's the only phone app that you've added?

ADL06: I've tried a few others but I, I just thought they were too…(audio interrupted)…they were too simple in terms of their description, that was already information I knew both from, you know, reading a few books in the past as well as my education as a nurse, so I was looking for something that had a little bit more to offer than, you know, "your baby is the size of a blueberry."

Interviewer:…(laughs)…um, are there any specific types of information that you were really hoping to get that you haven't really found apps?

ADL06: um, yeah I think that, so me, I think it would be valuable to have information about, let’s see, stress management or mental health in pregnancy that I haven’t really found any, you know, it’s not like my, I guess, I guess my, my app really talks about things, you know, this week you should make sure you, you know, you have your car seat…(incomprehensible)…and these are car seat safety tips, or this week you should make sure that you ask your doctor or midwife about, um, you know when they’re gonna test for group B strep., they don’t really have things like “this part of pregnancy can bring on new physical discomforts, and these are some ways to handle them, or you know, they’re, they’re more fact based than sort of individualized to kind of deal with the mental part of pregnancy, which I think is pretty substantial…

Interviewer: um, have you joined any e-mail, newsletter sorts of pregnancy programs, text messaging programs?

ADL06: uh no I have not

Interviewer: okay, and do you follow any blogs or facebook groups about pregnancy?

ADL06: no

Interviewer: okay…and do you have twitter?

ADL06: I do not

Interviewer: okay…based on your experiences as…(distorted)…having you know a family, friends, doctors, the internet, is there one person or place that you find has the best information about pregnancy and health?

ADL06: yeah absolutely, I mean as much as, I don’t know, I might check the internet for some information ultimately, if I’m uncertain or concerned I’m definitely gonna call my midwife, you know, I think of them as the ultimate resource, so um, I’ve sought out some advice from friends but ultimately checked with my provider in terms of whether or not that was something they would support or agree with.

Interviewer: so changing gears a little bit I wanted to talk about, um, weight gain, and I’m curious if you were given any advice about how much weight to gain during pregnancy and who gave that advice?

ADL06: absolutely, um, that was something that was on my mind from the very first visit, um, for two reasons, one, um, I’ve always struggled with my weight, um, I’ve never, I’ve never in my whole life been petite or thin, I’ve always struggled to keep my weight under control, and um secondarily um because I did fertility treatment for three years before actually getting pregnant I actually gained a fair amount of weight in that process due to all those hormones, so I was very concerned about what would be a safe and appropriate weight for me to gain, um, both you know to make sure that the baby was getting what he or she needed, um as well as making sure that I wasn’t putting myself in a new category of risk, um, so I asked that at my very first appointment

with (Redacted Name) one of the midwives and her suggestion was that um I gain no more than 20 pounds in my pregnancy.

Interviewer: and how did they, how did they, er, how did (Redacted Name) present that information to you?

ADL06: really just kind of a straightforward number, because I came to her with a direct question, um, and you know one other challenge for me is that I struggled with hyperemesis, pretty severely in the beginning, um, so it came up in terms of me asking about nutritional needs in general, because I'd already lost weight by the time I'd gone in for my first OB appointment.

Interviewer: and do you feel that the weight advice that you were given is accurate for your specific pregnancy?

ADL06: I do, although there was some confusion for me since I did lose weight, you know, my concern was "am I talking about 20 lbs from the, what I weighed on the day I found out I was pregnant or are we talking about 20 lbs in total from what I lost?" Um, because I lost 12 lbs it was pretty significant, and in that case if it had been from, you know, my lowest weight I would have already surpassed that in terms of what I've gained…um…so, um, there was no guidelines in terms of, you know, in you first trimester you'll gain this, in your second you'll gain this…um…I think because we struggled so much with my dehydration and…vomiting there was just the goal of getting calories in, so we didn't talk um about breaking it down much further than that. It is something I worry about though, even now, um, not to the point of, you know, not eating when I'm hungry or anything like that but I do weigh myself weekly and keep track of it…actually on the, the pregnancy app that I have, um because I wanna be conscientious of what I gain, and have an awareness of it…um…knowing that I will have to lose this weight afterwards and I'm, as I said I was already above a weight that I was comfortable with, and I worry about the risk factors of obesity.

Interviewer: and are you aware of any specific issues that can occur for the baby with excessive or not enough weight gain?

ADL06: absolutely! Um, obviously both directions is really bad for baby…um…you don't need baby to have an intrauterine growth restriction or or struggle to gain weight because obviously…um…baby needs all the fat that he or she can get, especially at this time in my pregnancy, ahm, which is gonna help them, you know, work on things like maintaining body temperature and heat after birth, um, but at the same time, with my family history of diabetes, and having failed my first glucose test, you know, I'm also concerned about baby being excessively large, which puts me at risk for, um, you know, not being able to have a vaginal delivery, and having to have a C-section which is not something that I want at all, as well as baby struggling to manage his or her um glucose after birth, I mean that's a valid issue.

Interviewer: and is weight gain something that you have looked up online as well to sort of add extra information to you've already learned from the midwives?

ADL06: I think I looked it up really early on, to get an idea of, um, what range of weight would be normal or acceptable for somebody my height and my body mass, um, and what I found online was consistent with [Midwife's Name] had shared with me.

Interviewer: and are there any things that you changed about your diet to help regulate your weight gain during pregnancy?

ADL06: um, yes. So, because I struggled with the hyperemesis in the beginning the goal was just, you know, eat anything that would stay down, um but as I've transitioned out of that period I've worked really hard to make sure that I'm conscientious of eating my carbohydrates with protein, in order to avoid high blood sugars, and, um I've also really only consumed water…um, I'm, I'm not, I'm lactose intolerant so I can't drink a glass of milk, um, I can tolerate yogurt here and there, but, um, you know I would enjoy like a lemonade or an iced tea or, you know, coffee with some cream in it before, and I've really worked hard to avoid any sugary beverages which I know are one of the worst offenders of empty calories and spiking your blood sugars.

Interviewer: who typically prepares the meals that you eat?

ADL06: I do.

Interviewer: and do you also do the shopping for those meals?

ADL06: yes

Interviewer: does your husband help in making decisions on what groceries to buy or do you make most of those decisions?

ADL06: I make most of those decisions by default, he kind of says I don't care and I think he just, he'll eat anything, he doesn't want to have to worry about it…(laughs)

Interviewer:…(laughs)…that sounds like my fiancee…um, how much money do you think that your household spends each week on food?

ADL06: Oh so much money, I feel like I give all my money to wegmans…(laughs)…I would say that our grocery budget is between 75 and 100$ a week.

Interviewer: And can you just tell me a little but more about how you've been eating during pregnancy given that you had that hyperemesis early on and…so I guess what things were working, what things were not working...

ADL06: sure, um…I think because I worked so hard to get pregnant I didn't want to complain, and so I kind of didn't know in the beginning how bad my hyperemesis was, I thought it was kind of normal, um, to feel the way I was feeling, and um…so I, I kind of just struggled along without asking for any help or medications 'till about 13 weeks, um, and so in those, really in those early weeks, between like 7 and 13 weeks to be honest with you I barely consumed anything, it was crackers and gatorade, and sometimes plain pasta or toast, it was mostly

carbohydrate-based, um, and like I said before the goal was just for to keep, get something that would stay down, because so much would not stay down, and I was very concerned about not getting enough protein, um, and I also struggled with constipation obviously because I wasn't getting much fiber…so um at 13 weeks, when I went in and said this is a big problem, um, they were able to give me some medications to help, and then after that I was able to really focus on making sure that I was getting good protein sources 3 times a day, and eating every few hours and, um, making sure I was getting enough fiber and whole grains, so…um…and, and since then my nausea has kind of stopped around 18 weeks, um, so really for the past 12 weeks I have been well enough to eat meat again, which was a really tough thing for me at the beginning, and, um eat, eat a lot of, a lot more vegetables, so I've been able to focus on getting those in for baby.

Interviewer: In adding in more protein and veggies were there any foods that you specifically chose that 'cause you thought that they would be better for your pregnancy that you may not have eaten a lot of before pregnancy?

ADL06: absolutely, um, I've never been a beef eater but I know that beef is a really good source of iron, um, and because, um, I do have a, a personal history of iron deficiency anemia I was very conscious of the risks of developing that again in pregnancy, so I've eaten, purposely eaten a lot more beef than I would have before pregnancy. Um, additionally I've been able to encourage my husband to try different greens that normally we wouldn't eat, um, like kale and collards…(advertisement goes on)… I'm sorry minerals and vitamins and that, maybe I might not get that much access to, so um, yeah we've tried some new things.

Interviewer: Has it been easy to incorporate those new things into meals or would having extra recipes be something that you would like to have?

ADL06: yeah recipes would be great because, um, obviously when you're pregnant you don't have as much energies as you used to, I used to be, you know, very enthusiastic about looking up new recipes and trying new things, and at this point, I kind of lack in that kind of enthusiasm so that would be wonderful!

Interviewer: okay…and now that you're able to eat more, would you say that the quantity of food that you've eaten in just the last 12 weeks is more than pre-pregnancy or does it feel about the same?

ADL06: it feels about the same to be honest with you…um…I feel like maybe during my, during my 5th and 6th month, um, after the nausea subsided, before I had this, you know, bigger belly where my stomach seemed to be smaller, I was able to consume more food, and I would be more hungry more regularly and I kind of gave weight to that, um, and I did have a big jump in my weight during those 2 months, um, but now that I'm 30 weeks, um, my eyes are bigger than my stomach, literally, so I, I'll make a big dinner but I usually can't actually finish it because I feel to full, so ultimately, I may be eating better than I was before pregnancy but I don't know that I'm eating more…

Interviewer: okay…and you mentioned frequency earlier what does the frequency of eating look like, so number of meals and snacks…

ADL06: um, right now my goal is three meals and three snacks a day, so if I go more than three hours without eating I find that my energy is low and I'm sluggish and I'm a little bit cranky, so um, I eat breakfast in the morning, um maybe around 7, then have a snack between 9 and 10, eat lunch between 12 and 1, have a snack around 3, and dinner in the evening and then that last snack of the day, um, after dinner is usually, it's not always consistent and if it is it's just fruit or vegetables…(coughs)

Interviewer: okay…you've talked a little bit about foods that you've added to your diet, what about foods that you have removed from your diet?

ADL06: um, sort of the standard things that I, I was told and read previously that I, I should avoid any soft cheeses or um, you know, no deli meats, um, um, making sure, like any undercooked or you know less cooked meat, um, I'm trying to think what else, I've, I've also, not that I was ever a soda drinker, I wasn't, um, but once in a while I, I might enjoy having, you know, a diet pepsi or something out at dinner, and I've completely stopped any soda intake whatsoever, um…I'm trying to think what else…oh, you know, we used to be, my husband loves candy, and off course he's like 110 lbs, so he can eat it without having the risks but, um, we've just avoided having any candy in our house, just because of the temptation to just have that sugar rush.

Interviewer: um, and you mentioned that you stopped drinking soda…

ADL06: yeah…

Interviewer: and so, even if it's diet, um, I'm just curious, what your, your reason was for choosing not to drink soda…

ADL06: well I actually think that diet soda is worse for you than regular soda, I'm, I'm a big believer that those fake sugars actually ultimately are just as unhealthy as the corn syrup, um, but I kind of am of that age where like I grew up drinking diet pepsi in college and that's kind of like what tastes good to me, so I would have, I always would have thought of it as sort of a treat once in a while…

Interviewer: ehm ehm…(agrees)

ADL06: um, but I just…I'm just not interested in exposing the baby to those chemicals.

Interviewer: okay…that makes sense…and, are there any other foods or drinks that you have heard that you should be careful about eating during pregnancy that you didn't mention yet?

ADL06: yeah I just realized, alcohol obviously, I'm abstaining from any alcohol, um, you know I've heard that some seafood is not considered safe in pregnancy due to mercury content; we're not really big seafood eaters so that's not as, um, big of a deal for us…and…I mean, I'm aware of the risks of Listeria, you know, so that's a lot of those meats and cured meats and cheeses like I talked about, um, I can't think of anything else that I was told to avoid…

Interviewer: have you ever heard that a pregnant woman's diet needsto change at different times during pregnancy?

ADL06: yes I have, um, in fact the phone app that I have really made it clear that any increase in calories really only needs to happen in the third trimester, um, because I, I do have some older books that I've read that said you should be eating an extra 300 calories a day during pregnancy but, um, I am aware that, that there are some more recent research that says that that may not be as necessary as we once thought, and it's gonna be at the end of pregnancy when that's necessary, which is ironic because it's when it's been hardest I think to get it in…

Interviewer: right, as you're feeling fuller…(laughs)

ADL06:…(laughs)

Interviewer:… not so much space to fit the food…

ADL06: oh not at all…

Interviewer: um…let's see…so on those same lines, are there any specific foods that you've heard should be added or removed at different times of pregnancy?

ADL06: um…I think, I think really what I've heard is, you know, you really want make sure you're getting good protein, 'cause protein is, um, sort of the building block of basically all cells, so that's part of the reason why your protein needs in pregnancy increase, and then, um, just talk about the increased need for fiber, and your fruits and vegetables just because the hormones in your body slow down your gastrointestinal system which can cause constipation, so, um, the proteins more for the baby, but I've been told the increase in, in fiber is really more for the comfort of the mom, um, and then of course you know your hydration needs increase, um, which was something that, you know, I've struggled with in the beginning with the vomiting…um…but I've worked really hard in the last couple of months to really get a lot of water in everyday. I know there's some research about the importance of, um, DHEA, which is a vitamin component, um, that it's important in the third trimester for brain development in babies, but my prenatal vitamin has it in there so I'm, I don't worry about food sources of that.

Interviewer: okay…and have you changed the way that you prepare food since you became pregnant?

ADL06: um…I would not say that I did, no, my husband and I are, are relatively, um, conscientious, of, of how we prepare foods in terms of um using olive I-, olive oil or a non-cooked, or I'm sorry a non what is it? Like the cooking spray….and you know we don't fry things, or cook things in butter and, um we, you know, we we work on using good fats in our diets, and, um, I mean I don't think I have changed the way I prepare anything.

Interviewer: and you mentioned that you're lactose intolerant, has dairy been something that's hard to get the number of servings that you feel that you need each day?

ADL06: um…probably yes, you know, I, I do, like I said I can tolerate yogurt quite well, so I, I have a greek yogurt every single day, just because it's my calcium as well as it's a good source of protein, um, and I can tolerate some hard cheeses but things like sour cream or cream cheese, you know I'll have them every once in a while but they usually cause some GI discomfort, so I do take an extra calcium supplement, calcium with vitamin D, um, to kind of help with my calcium needs, but I definitely don't meet the requirements for dairy intake.

Interviewer: and it sounds that you've been very conscious about foods that have sugar, um, but what about foods high in salt, is that something that you think a lot about now that you're pregnant or…

ADL06: well, um, we've always been sort of low sodium conscious because my husband has blood pressure issues, so um, we don't…we, we salt our food very sparingly and I do check sodium content but because we don't eat a lot of prepared foods, which are really some of the major sources of sodium, it's not that big of a deal, I think our, our, um high sodium content comes from like, um, maybe tortilla chips and salsa, that's sort of a treat we enjoy and those tends to be pretty salty, um, if we go out somewhere and eat somewhere then you know obviously your sodium intake higher…

Interviewer: mhm…(agrees)…and have you had any cravings so far?

ADL06: I have! Um I've always liked pickles but definitely dill pickles, um, taste good to me all the time even when I was vomiting in my first trimester I would throw up and then I would say, um, I really want a pickle and my husband…(laughs)

Interviewer:…(laughs)

ADL06: um, but actually I read an interesting article that said sometimes when your…your stomach acid is…is, like the balance isn't there that you crave something more acidic to help balance it out, I mean I don't remember the details, um, but yeah pickles always sound good to me.

Interviewer: and have you had any cravings for non food items, like ice, clay, dirt?

ADL06: no, no play dough, I'm okay!

Interviewer: …(laughs)…yeyy! Um, what smells do you like more or less now that you're pregnant?

ADL06: um…cooking smells that never bothered me before bother me now, so onions and garlic and broccoli, even though I can eat those things just the smell of them cooking I usually can't handle so my husband has to come take over for that kind of stuff. Um…so…um one of the things that helped with my nausea is that I had some orange essential oil, I would put in this little diffuser and it would, would make like wherever I was smell like oranges, and so oranges have always smelled good to me this whole pregnancy so, um, not orange juice but like a fresh orange,

so that has been something that if I was nauseated I would, would try to eat and smell a fresh orange, or, or use my essential oil.

Interviewer: um, and so you've mentioned that you're taking your prenatals and that you have a calcium vitamin D supplement…

ADL06: yes

Interviewer: are there any other supplements that you've added for benefits that they might have for your pregnancy?

ADL06: you know I try not to go too crazy with it because I know that you can kind of go down the slippery slope of taking everything…um…my prenatal is pretty comprehensive, um it has an omega-3 fish oil in it and it has some major important, you know, B12 complex, um, and nobody really told me to take the calcium and vitamin D but I just know that, you know, basically that baby is gonna, the baby is going to strip my bones of calcium if he or she isn't getting enough and I'm already at risk for not getting enough just because I haven't been a milk drinker in fifteen years so, um really, partially that's for my benefit too…um…but nope I've just stuck to those two things.

Interviewer: and have you specifically chosen any foods that are fortified, like cereal products where vitamins and minerals are added?

ADL06: um, yes and no, I mean I, I'm not a big cereal person just because the carbs in the morning usually end up making me feel like I'm gonna crash, I do, um, let me see what I had this morning…I did have some granola this morning, it's an organic Cascadian farms granola, and it's been one of my favorites throughout pregnancy, and it is fortified, you know, with vitamin A, C, D, calcium and iron, things like that, um and in addition I do have almond milk, that I would put on, you know, my granola, and that is fortified as well…(coughs)…but I haven't specifically chosen anything because it was fortified, I have more by default chosen things that I like that happen to be fortified.

Interviewer: okay! And have you received any advice on things to eat during pregnancy um from family, friends, the midwives…

ADL06: definitely, you know, family and friends always wanna give you their ridiculous, unsolicited advice, so, I haven't really listened to it too much, um, I think my mom tried to give me a lot of advice in the beginning when I was very sick because she never felt that way when she was pregnant so she would give me things like "oh you just need this or you just need that…" um, I haven't really listened to any of it, at all actually…and I haven't asked too many questions of the midwives about, um, about nutrition of food too much just because I feel like I have a pretty decent knowledge base as a nurse, um, of understanding you know obviously what are healthy and unhealthy choices…and you know my biggest concern in terms of nutrition in this pregnancy was not gaining weight or gaining too much so I didn't ask too many specifics of them…

Interviewer: okay…um, has the internet been a place that you've gone to look up any like random questions that might have popped up along the way in relation to nutrition?

ADL06: um, yeah I guess so, I think, um like I said before I think I did use it to look up things like, you know, how much weight gain would be appropriate based on, you know, my pre-pregnancy weight, um, or what specific foods, you know, checking for foods safety on certain things…but, I wouldn't say that I regularly looked for answers to nutritional questions on there…

Interviewer: um, and so the last thing I wanted to talk a little bit about is physical activity, and I'm just curious if you've heard of any specific guidelines for physical activity during pregnancy…

ADL06: yes! Um… I've definitely heard about certain exercises that are good for pregnant woman and certain exercises to avoid, um, you know I've been told that, you know, due to balance changes you shouldn't be on a bike or, you know, rollerblading, things like that, um, or do the kind of sport where, you know, a projectile or a ball or something could be coming towards you or your stomach…um…and that exercises like walking and swimming, which have less stress on the joints, uh are good for pregnant women…

Interviewer: and where did you hear about that advice?

ADL06: um, I think it was probably a combination of a few books that I've read in the past and, um, probably the pregnancy app that I have…

Interviewer: if you wanted to know more about exercise in general, or a specific exercise, where would you go to look that up?

ADL06: um, I'm not sure, I mean I'd probably…you know, google it, like I talked about before…

Interviewer: ehm ehm…(agrees)

ADL06: and look for a reputable source…I think I did ask early in my pregnancy about making sure that certain exercises that I wanted to do were safe, I think I asked about yoga and walking and swimming…

Interviewer: and who did you ask?

ADL06: I believe I asked [NAME of the Midwife], in one of my early appointments…

Interviewer: okay…and are you currently exercising?

ADL06: yes but not as much as I would like…(laughs)

Interviewer: and what sorts of things are you doing?

ADL06: at this point I am primarily walking, and um, I'm doing my yoga stretches more than anything else for comfort, um not necessarily for exercise, I'm doing them for the benefits of stretching and, you know, how they can help keep me more comfortable…

Interviewer: ehm ehm…(agrees)…and how does the amount that you're exercising now differ from before pregnancy?

ADL06: um, I would say it's less, um, before pregnancy I was going to maybe 2 intense yoga classes a week, and, you know, trying to take at least one long bike ride a week, um, and I'm just not getting that level of cardio or of sweating um, so, I am…the frequency is, is similar but the intensity is less.

Interviewer: okay…and do you think that having the hyperemesis in the beginning of pregnancy, how did that affect you, and I guess like also…and it sounds like, you know, you've had fertility issues that have been going on…how does that change you perspective about exercising and diet?

ADL06: well, you know, to speak of the hyperemesis…when you're not eating you have no energy…can't keep anything down so I was surprised at how quickly I felt as terrible as I did and had so little energy, and so really from about 6 to 7 weeks all the way to about 18, um, I did almost nothing, I did the bare minimum so that I could get to work and get home, um, because you just don't have any physical resources…and so that really kind of put me behind on where I thought I would be in terms of fitness in pregnancy…um…so that was really tough, um, but in terms of the fertility, um, a huge component of infertility treatment is talking about nutrition, and I did everything from dairy-free, gluten-free, vegan, I tried every diet in the world to try to, you know, see if I was intolerant to something that was causing some kind of immune response so that I couldn't get pregnant…ultimately it it turned out to be just…(laughs)…circumstantial I guess, that it just happened when it did, but um, I've been very aware of the important role of nutrition and diet in pre-conception…so that's part of the reason I was going to yoga so regularly and trying to stay fit, um I have polycystic ovaries which, you know, is a frustrating disorder that makes it hard for you to lose weight and stay fit to begin with…um, and then the more weight you put on, the harder it is for your ovaries to function normally, so, it's it's been a long battle of trying to keep nutrition and exercise in balance during this process.

Interviewer: ohm…have you heard of any certain amounts of exercise that a woman should do during pregnancy, so time and um frequency during days?

ADL06: um, I feel like what I know is maybe like, you know, half an hour three times a week…(incomprehensible)…should be trying to get some kind, kind of physical exercise, um but I have to say I don't know where I'm coming up with that number, I don't know, I don't remember if someone told me of if I read it somewhere…

Interviewer: okay…and now that you're feeling a little bit better are you feeling like you have more energy and more drive to exercise?

ADL06: um, I did sort of in those middle months, in in the second trimester, um, we were still having you know the worst winter of our lives…(laughs)

Interviewer:…(laughs)…unfortunately this has been a long one…

ADL06: it's crazy, um…but I did have more enthusiasm for it, and, you know, and the last couple of weeks I've struggled with having bronchitis which has made it hard for me to exercise because when it hurts breathe the last thing you're gonna wanna do is breathe harder…

Interviewer: oh definitely…

ADL06: (coughs)…excuse me, um, but you know, more than having energy for it, I have more enthusiasm for it because I'm also aware that being as fit as I can be um going into labor if gonna help me to have the labor experience that I want which is an unmedicated experience, so, you know it's kind of like training for a marathon…just on a smaller scale…(laughs)

Interviewer:…(laughs)…um, is there anything that really motivates you to exercise?

ADL06: um, really, it, it is being fit for labor, that kind of motivates me at this point, um, I don't necessarily find it energizing or, um, as relaxing as I did before, (distortion) uncomfortable but I keep thinking about…wanting to be able to be physically active, enough to, you know, have a good birth.

Interviewer: and have you heard of any effects that physical activity can have on the baby?

ADL06: um, actually no I haven't.

Interviewer: okay…and if you, in talking to people and reading on websites…are there any exercises that you've heard are the best exercises to do during pregnancy?

ADL06: yes, I've been told that a couple of yoga positions, um, called cat and cow really help with your back and basically help baby get aligned properly and help with your discomforts and, um, the majority of my friends who had babies said you need to walk, you need to walk, you need to walk…

Interviewer: okay, um, so I think that's all the questions that I had, but I want, just wanted to ask you is there anything that you thought of as a response to a previous question after we had moved on to other topics that you wanna go back to briefly?

ADL06: um, not that I can think of…

Interviewer: okay, um well I just wanna thank you so much for your time, this is been really nice chatting, um I did have a couple of things that I wanted to talk about before I let you go…

ADL06: sure

Interviewer: as part of the study you had also, um, you said that you were interested in the focus groups that we're going to offer, and due to lots of scheduling conflicts with me and the study recruiters I believe that you met [NAME REDACTED]…

ADL06: yes I did…

Interviewer: um, so we're actually going to host our focus groups next week…

ADL06: yes…

Interviewer: um, [date redacted] and then [date redacted], and I can send you some text messages with the times and the dates, um, and then you are free…we're for sure doing both so, um, you could go to either one if you're interested, it's not required, it's optional, um, but I definitely wanted to remind you about that since it's right around the corner…

ADL06: yeah, no I'm definitely interested, um so I am a (MISSING WORD) nurse, and I usually get my schedule on Thursday afternoon or Friday mornings, so I don't know if I'm scheduled yet, um but I do have your phone number so after I get my schedule I can text you to let you know if and when I'm gonna be available.

Interviewer: sure, that would be great, um, and you can even decide up till the last, the last minute really, um, so then we'll have some snacks for the one on Thursday in the morning, to like…(audio distorted)…snacks like fruit, or maybe some of that (MISSING WORD), and then on Tuesday it's at dinner time 5.30 to 7.30 so we're providing, um, a dinner, so, um just so that you know that ahead of time…and the other thing was I have, I already tried to look up your name on facebook and it does not give me the option to add you as a friend so that I can add you to the groups so I was wondering if you can search for me instead.

ADL06: I can do that, yeah I…so I used to work at the pediatric clinic at the hospital and a couple of my patients tried to facebook me…little akward for me…

Interviewer: yeah…I would agree…(laughs)

ADL06: so I've added all the blocks you can so that people can't find me, so I can happily find you…so do I need to just search under your name or is it under a group name?

Interviewer: um, no, so, in order to, I made the group private, so the only way I can add someone to it is if they're actually friends with me first, and I'll, um, as part of all the rules of the study once the study is over I, I'm de-friending everyone, but just temporarily you have to be friends with me…my, facebook name is just my full name is…[NAME REDACTED]…and I can send that to you in a text also, um…

ADL06: okay

Interviewer: but I have a, I should probably change my profile picture so it looks more like me…(laughs)…it's when I went on a whale watching tour and it was really cold so we have

these like whole-suits on and my head is covered by a giant red hood with a yellow piece and, um, so it just sorts of looks…(INCOMPREHENSIBLE)

ADL06:…(laughs)…I actually have my computer up right here, I think I found you…

Interviewer: oh okay…

ADL06: I will add you…and then, um, you'll be able to add me to the group.

Interviewer: perfect!

ADL06: great!

Interviewer: I will keep my eyes open and, oh, I think actually I just got it…ok…so I'm confirming and then I will add you to the group right now.

ADL06: great!

Interviewer: all right…so do you have any questions about the study that I can answer?

ADL06: no, I, I think I'm all set.

Interviewer: okay, well perfect? Um, I'll just say have a wonderful day and I look forward to chatting again soon and hopefully I'll see you next week.

ADL06: alright sounds good, thanks [NAME REDACTED]

Interviewer: yeah bye bye…

ADL06: bye bye

Skype disconnects

ADL07

INTERVIEWER: So I always start with just some like really basic introductory questions to get to know you and a little about your pregnancy. So the first question is how old are you?

ADL07: I'm 34.

INTERVIEWER: Okay. And how tall are you?

ADL07: I'm five foot nine.

INTERVIEWER: And about how much do you weigh right now?

ADL07: Right now? I weigh a hundred and fifty-one.

INTERVIEWER: Okay. And do you remember what your pre-pregnancy weight was?

ADL07: 133.

INTERVIEWER: And is this your first pregnancy?

ADL07: This is my first.

INTERVIEWER: And would you describe your pregnancy as planned?

ADL07: Yes.

INTERVIEWER: Okay.

ADL07: Very much so.

INTERVIEWER: And um what hopes and goals do you have for your baby?

ADL07: Just want them to be healthy and happy, and I don't know, I mean I think that's the base thing.

INTERVIEWER: Um have you ch-, or faced any challenges with this pregnancy at all?

ADL07: No, not really. I mean, you know, being like the first time I've been pregnant and you think you kind of have an idea of what to expect from like reading things and hearing from people and stuff. But you know you never really know what to expect. And then um, you know, overall I felt pretty good, so, nothing too bad. And then we had one kind of like mini scare on an ultrasound, which was a little stressful but overall I would say it's been a pretty easy pregnancy. Knock on wood. So.

INTERVIEWER: That's good. Yeah!

ADL07: Nothing too bad.

INTERVIEWER: That's wonderful.

ADL07: I'm still working, I'm still functional, overall I feel pretty good so.

INTERVIEWER: Um and what do you think are the most important things that you should be doing to have a healthy baby?

ADL07: Um, I should probably be eating well and eating better than I am (laughs). Um and definitely exercising. Um making sure I'm sleeping enough, taking care of myself basically. If I take care of myself then I figure I'm probably taking care of the baby.

INTERVIEWER: Yeah!

ADL07: So.

INTERVIEWER: That makes sense. Um so for… let's talk about food first.

ADL07: Okay.

INTERVIEWER: That seems like an area of interest. Um just tell me a little about I guess how you've been eating and how like that has changed over the course of pregnancy.

ADL07: So, I'm typically a pretty, I consider myself a pretty healthy eater, overall. And most people I think see me as a pretty healthy eater. Um I'm, I've always been a vegetarian or at least for you know a really long time. And um I try and eat like whole foods, non-processed foods, I don't, I mean I can't remember the last time I've had McDonalds. Um and I don't drink sodas, and I don't you know, I pretty much just like drink water all day long, and stuff like that. And I actually right before, the month before I got pregnant, I had a little breast cancer scare. And I was like, oh, I need to you know, make sure that I don't get cancer and I'm gonna kind of try to prevent it with nutrition, so, I revamped my whole… I'm gonna eat super healthy and whole foods and all that stuff. And like I didn't eat any sugar, and no flour, and no junk. And then all of a sudden, I was like, I have no willpower, and at work, the doctors bring in donuts, and on weekends there are bagels and things. So all these things that I hadn't been eating for a really long time—things that I don't buy for myself ever. I just realized, like oh, I need to eat that. And I was hungry all the time, and I was craving all this stuff that I hadn't really eaten and I found out I was pregnant. And that kind of continued with, I just, you know, like I used to eat a salad every day, and I think I've probably had two salads since I've been pregnant. Like I just, I don't know. They don't sound good anymore. Um so things have definitely changed and I've been a little disappointed in how I've been eating during the pregnancy. Um you know it's when, I go through like my little what did I eat yesterday, I feel really bad and then she's like no, you're not really doing too bad compared to other people. But for me, what I'm used to, it it doesn't seem as healthy as what I used to eat so…its been a little bit of a struggle. It didn't, it's just not quite what I was expecting. I was like yeah, when I get pregnant I'm gonna be like super healthy, and

only eating healthy things and of course my body, or my brain, or whatever, like, you want cheese and crackers (Interviewer laughs), and pizza three times a day. So, that's been a struggle. Then I seem to have lost my willpower. And things that I never even thought of eating, um like, oh, that looks good I'll take one of those. You know. So, that's…

INTERVIEWER: Do you think that the, the cravings mean something? In relation to what you or the baby need?

ADL07: I don't know, I mean that's what everybody says… Potentially, I guess so, you know that there's something that your body's needing or the baby needs. Um, I've been craving a lot of dairy products, so that's probably a sign… I don't know. Like yogurt all of a sudden. I wasn't eating any dairy prior to getting pregnant. I don't, I'm lactose intolerant and I don't normally eat a lot of dairy. Except for yogurt sometimes and I just I don't know, I kind of pretty much stopped, but now I find that I eat at least one yogurt every day, if not like some cheese and other dairy things, so.

INTERVIEWER: Has that bothered you at all?

ADL07: Um not really.

INTERVIEWER: That's good (overlap).

ADL07: I seem to be tolerating dairy better than I was pre pregnancy.

INTERVIEWER: That's good.

ADL07: For some reason.

INTERVIEWER: That would be awful if you craved it and it wasn't really working out so well.

ADL07: Yeah. Yes. No, it seems to be fine, so.

INTERVIEWER: That's good.

ADL07: Yes.

INTERVIEWER: Um and you're still a vegetarian now?

ADL07: I'm still a vegetarian, I have not, everybody was like, oh wait til you get pregnant, you're gonna want to eat meat. But I haven't had any cravings for anything meaty. So.

INTERVIEWER: That's good, I have heard of that happening also.

ADL07: I just for some reason, I like just don't want to eat vegetables anymore. So I've kind of compromised with like eating like I find that if I bring in to work, especially like I'll cut up just

veggies and dip em in hummus or something and that will do it for me. But I can't like sit down and eat a salad anymore for some reason. I don't know why.

INTERVIEWER: Is it smell or taste or?

ADL07: It's just, it doesn't sound good. I mean it's not like it's revolting or makes me feel like I'm gonna throw up or something. And I just have no motivation to cook or put stuff together, so like the easier the better. So cutting up veggies, it's easy to eat, it can you know, be kind of snack throughout the day. And uh, I know it's healthy, so. That's been my compromise.

INTERVIEWER: Well it sounds like you're still…

ADL07: I'm trying.

INTERVIEWER: trying to fit them in wherever you can.

ADL07: Yes.

INTERVIEWER: And whatever sounds palatable.

ADL07: Yes.

INTERVIEWER: Um, have you had any cravings for any like specific food items that have been really regular throughout your pregnancy?

ADL07: No, I find that if I see something…well for, I guess for like the first couple of weeks before I even knew I was pregnant, um, all of a sudden I wanted like cheese and veggie subs, which I couldn't remember last time I had had one. Um, and it's kind of like, at this point, or I guess as things progressed, I would see somebody eating something, and I'd be like, oh my gosh that looks so good. Like somebody at work had stuffed shells, so I called my mother in law who's Italian, and she made like three thing—you know three giant things of stuffed shells. And I basically lived off of them for a week. And I was like okay, I'm done with that. And then like the next week I saw somebody else eat something, so it it's it seems like I'll be inspired by someone else, and then I get fixated on it, it's like I can't stop thinking about it 'til I eat it. And then I'll have to eat it a couple of times, and then I like move on to the next thing. But the one thing that I really seem to like this—this whole time, I would say I pizza, I guess. I like pizza.

INTERVIEWER: It's good.

ADL07: Yeah. That's the big one.

INTERVIEWER: Um.

ADL07: But I haven't had any like 2 AM oh my gosh you have to go out and buy me ice cream cravings or anything like that. So.

INTERVIEWER: Well that's good. Um, who typically prepares the meals that you eat?

ADL07: Mmm…I would say, I prepare most of what I eat, and then sometimes like my mother in law will cook for us occasionally, she lives in town. My husband's been really busy, so he's not been cooking at all. And then, we probably have been doing like convenience foods than we used to. Um but I try to do, like we'll do the like Amy's frozen meals or something, or try to get low sodium and those sorts of things. You know still have vegetables and organic stuff in them. So you know, again it's like I'm not driving through McDonald's, but it's not like I'm cooking it fresh right then and there like I used to, so. But yeah I pretty much am in charge of my own food.

INTERVIEWER: Um and do you are you normally the one that does the shopping for groceries as well?

ADL07: Pretty much. Yeah.

INTERVIEWER: Okay. And how much do you think that your household spends each week on groceries?

ADL07: Oh gosh…that's a really tough question. Because we, I end up going a couple times a week usually. Um…I don't know, probably at least a hundred dollars. Especially again, if we're buying more convenience foods.

INTERVIEWER: Um have you ever tried to make any modifications to your diet because you've heard that something is better during pregnancy or worse during pregnancy?

ADL07: Um I stopped I mean I've stopped eating those things that you say you shouldn't eat, like certain cheeses, um I stopped drinking coffee. Actually prior to getting pregnant. I pretty much stopped having even like caffeinated teas. And then I was drinking green tea and then I heard that can interrupt the folic acid absorption. So, I stopped drinking green tea. Um, and um, no I mean, not really. At this point, I'm just I guess I'm in the beginning of the pregnancy, I just, like nothing sounded good, and then the things that sounded good were really like, high fat, comfort foods, and I kind of gave in to that for a while. And now I'm just trying to get back on track with eating better, cuz I know I should. My friend gave me a book, you know like what to expect when you're expec-
INTERVIEWER: Mhm.

ADL07: No it wasn't like by the author, but it was like what to eat when you're expecting maybe or something like that. So, I've kind of flipped through a little bit. But it seems like for the most part, and again, my like pre pregnancy views on food, you know like I wasn't eating dairy, and things like that. But for the most part, people thing that that's a healthy thing to eat when you're pregnant. That you're, you know, gonna need more calcium from things like dairy and stuff like that. So, ultimately I guess maybe I'm not doing so bad after all.

INTERVIEWER: Yeah! I know it sounds like you're doing well. Um, what about the quantity of food that you're eating? How does that compare to pre pregnancy?

ADL07: Mmm…I find that I… Well, I mean every day is kind of different, and especially like if I'm at work, I find that I get really hungry, and I'm like hungry every hour, every two hours.

INTERVIEWER: Mhm.

ADL07: Um, and there's some days where it's like I just, you know, I can eat likea little yogurt, or, like I'll go to lunch and I'll eat some of my hummus and veggies, and then I'll be like I can't finish the whole thing so I like bring the rest out with me and just kind of try and snack throughout the day. So, it's hard to say. And then there's some days where I feel like I've eaten an entire meal and then like an hour later I'm hungry again so I'm having a snack. So um some ways I feel like I'm eating more than I usually do, and I think some days that's the case, and um I just don't tolerate being hungry anymore. Like I used to before I was pregnant, you know, oh okay I'll eat before I go to work, and then I might eat a little snack like mid-morning. And then lunch would be any time between you know noon and three o'clock whenever I got a chance to eat. And if I got hungry at noon but I couldn't eat til three, it was kind of like oh that's okay, you know. Like I was busy enough and I could kind of ignore it and I knew I was gonna eat eventually. And now it's like as soon as I get hungry, I have to just like stop what I'm doing and eat. So, and I'm, I think I'm nervous that I'm like not gonna eat enough sometimes. You know it's like oh if I'm hungry, I need, the baby needs to eat. So I think some days, there are some days where I probably eat more than I need to. So. But I I think it's better to I don't know eat a little too much than not enough, so it's like. When I get hungry I usually.

INTERVIEWER: So what makes you feel that way?

ADL07: I don't know, because I think, I mean I know it's not too much and you don't want to gain too much weight, like both are bad you don't wanna be one extreme or the other. But I feel like if you eat a little bit too much, you know at least, the baby's still getting all the nutrients it needs and it's growing, and if you don't eat what you need, then you're starving the baby. And I feel like that would be worse.

INTERVIEWER: That makes sense. Um… I know that you said it kind of varies with like how much you're eating every day, but do you feel like gen-, in general you're hungrier?

ADL07: I think so.

INTERVIEWER: Okay.

ADL07: Yeah. I would say so.

INTERVIEWER: And…let's see. And you mentioned some foods like um some cheeses (ADL07: Mhm) that are not safe to eat. Um what other things have- are you aware of that you shouldn't have, or should have?

ADL07: Um, I know like, sandwich meats you're not supposed to have.

INTERVIEWER: Mhm.

ADL7: Um sushi, you know like raw fish, you just have to more careful of like bacteria. And things like that. And then obviously other toxins.

INTERVIEWER: Have you had any cravings for non-food items at all? Like…

ADL07: No…

INTERVIEWER: Okay. Just making sure.

ADL07: Sarah’s asked me that, each time I met with her. I was like, no, thank goodness, not yet.

INTERVIEWER: Yeah.

ADL07: So.

INTERVIEWER: Um, let’s see. And have you changed how you’re preparing foods at all? Whether you’re baking, broiling, frying?

ADL07: Not really. But I mean I’ve always been a pretty like simple, you know, food prep person. Like I usually steam my vegetables. Either steam em or like roast em in the oven or something like that. Like I don’t fry stuff very often. And so, um yeah, no I haven’t really changed except that I just I’m not preparing things as much for myself as I used to. So. And I think also being pregnant in the winter didn’t help. Cuz during the summer we are like members of the CSA.

INTERVIEWER: Mhm.

ADL07: So we get tons of fresh fruits and vegetables and it’s a lot more inspiring to cook good healthy foods during the summer when you have all the stuff in abundance. And I feel like during the winter it was just like hibernation mode, and...

INTERVIEWER: Yeah, and this winter was not pleasant.

ADL07: This winter was horrible. So, but no, I haven’t purposefully changed the way I prepare things.

16:46
INTERVIEWER: Are there any smells that you like more or less now?

ADL07: I can’t stand coffee breath. That’s like the one thing my husband drinks coffee. And I’m like I can’t even be in the same car. So but no. There’s not really anything else that I’ve noticed that I really like or don’t like.

INTERVIEWER: And um have you added any like special foods or products to your diet that have, that you know have a lot of vitamins or minerals added? So like an example would be prenatal vitamins.

ADL07: Um I started taking prenatal vitamins before I got pregnant, and just obviously continued with that.

INTERVIEWER: Mhm.

ADL07: Um, but I don't think that there's really anything else. Again, I just try to do my best to like have a good variety of foods and make sure I'm getting protein and vegetables and fruits and stuff like that so.

INTERVIEWER: And um do you eat cereal products at all?

ADL07: Yes. Yeah.

INTERVIEWER: Are, are they ones that are fortified with vitamins and minerals or…

ADL07: You know, I don't know. I'm not sure.

INTERVIEWER: Organic or?

ADL07: Yeah. That's like some organic…

INTERVIEWER: Oh okay.

ADL07: …multigrain, flake.

INTERVIEWER: I had an organic cereal called um Peanut Butter Bumpers.

ADL07: Oh.

INTERVIEWER: It's probably like kids' cereal (ADL07: Yes), which was really good. And the Puffins?

ADL07: Mhm.

INTERVIEWER: Have you ever had those?

ADL07: I have

INTERVIEWER: Those are good too. I really like those a lot. Um have you received any advice about what to eat? Um during pregnancy because it has some sort of health benefit for the baby?

ADL07: Um, not really, just people, people just like at work are just very like they're very focused on making sure that the pregnant ladies eat, so it's like, you know they want us to get to lunch and they're like oh, it's good for the baby. Feed the baby. Um, but I don't think anybody's really commented too much. Like my friend gave me the book, the you know on what to eat when you're expecting just to make sure that I was eating some of the right things. Um, cuz she knew that I had kind of changed my diet a little bit especially in the beginning; I was eating not so great. Um, but yeah no I haven't really gotten a lot of advice on food.

INTERVIEWER: Um what about um weight gain? Have you received any advice about that?

ADL07: Um I mean obviously I had like the recommendations you know from the midwife that I should gain about 25 pounds.

INTERVIEWER: Mhm.

ADL07: And then you know everyone tells their stories, oh I gained, I only gained 22, or I gained sixty and here is the picture of me. And um you know so everyone has a different story. And kind of a different viewpoint on that, and this month, the, I guess it was the last months, not this appointment I just had but the one like four weeks before that, that I had gained like 8 pounds in one month. Um I was like, oh that might be a bit much, but the midwife said that um you know obviously if I continue on that path, it we would have to intervene in some way, but she figured a lot of times she sees in like that time period of growth spurt and then of course this last month I didn't gain nearly as much so. I think things have settled out a little bit.

INTERVIEWER: Um so how do you feel about the recommendation that you received of 25 pounds, do you think that is right for you? Or…

ADL07: I have a feeling I'm gonna gain more than that. Um which is you know I think as long as I don't gain like 60 pounds, I think it's probably okay.

INTERVIEWER: Mhm.

ADL07: Um hopefully I can stave off gestational diabetes and preeclampsia and stuff I mean those would be my biggest issues. Um and I don't wanna have a giant baby. Um cuz I still have to push it out. So you know, so I don't wanna do anything that's gonna harm, ultimately I don't wanna do anything that's gonna harm the baby. Um but if I gain 5 pounds over like the you know if I end up gaining 30 pounds I don't think it's the end of the world and I think in the grand scheme of things, like to stress over every pound is probably worse for me than just trying my best to eat the best that I can and making sure that I'm eating enough and…

INTERVIEWER: Yeah.

ADL07: You know if it's 5 pounds over, it's 5 pounds over, so.

INTERVIEWER Um have you seen advice that's different from what the midwife said anywhere else from things that you've read or people that you've spoken to?

ADL07: Mmm, not really, I mean it sounds like fo- you know kind of the common advice for women who go into pregnancy like at a healthy weight is the 25-30 pound recommended weight gain.

INTERVIEWER: Mhm.

ADL07: So, that seems about right. Although that doesn't give me much more to go. And I feel like the baby still has like a lot of time to grow, so I'm a little, we'll see. That's why I think it's gonna be more than 25 pounds.

INTERVIEWER: Mm how far along are you right now?

ADL07: I'm 26 weeks.

INTERVIEWER: Okay. So I think that's over halfway!

ADL07: I know I start my third trimester…in like two days.

INTERVIEWER: It goes by fast doesn't it?

ADL07: Yeah… Well the first trimester was soo slow. Just because I think I was really nervous and it was like I need to get past these certain kind of points you know just cuz I was, I've heard stories about people miscarrying and stuff like that, so it was like, every week that we got through was kind of a good thing, and then every little milestone, like when we first heard the heartbeat and all that stuff, it was kind of like okay, I can relax a little more. And you know you're never gonna stop worrying about your kids you know.

INTERVIEWER: Yeah.

ADL07: Before they're born, after they're born, when they're 50 like you know, I realize that I'm in for a life of worrying about children. Um, but I've I'm much more like I don't worry about it as much as I did in the beginning. And like every little thing that I felt or didn't feel. At the beginning I was like, (gasps) uh oh, you know what does this mean? And now I'm just kind of like whatever, I'm used to being pregnant now so I don't stress out about it as much. Um but in the second trimester was just like, I feel like that has flown by. Like kind of once I felt more comfortable and I felt like we were past kind of the big milestones and we had our ultrasound, and everything looked good, and you know that sort of thing. And now I'm like in panic mode, where I'm like, we don't have the nursery ready! And this baby's gonna be here any minute you know! So…

INTERVIEWER: Do you know if it's a boy or a girl?

ADL07: Mm mm.

INTERVIEWER: Oh, surprise?

ADL07: We're gonna wait. Yeah.

INTERVIEWER: Have you guys picked names for a boy or a girl?

ADL07: We have, and we both agree on a girl's name, and we can't decide on boy's name. So. It'll probably be a boy. Just gotta…

INTERVIEWER: That's how it always works out isn't it?

ADL07: We'll see.

INTERVIEWER: Um so you were saying, I mean along the way you know

ADL07: Mhm.

INTERVIEWER: you kind of like have these moments where milestones

ADL07: Mhm.

INTERVIEWER: and you're worrying about little things here and there. So what do you do um to look up information to kind of make yourself feel better when you have a worry?

ADL07: Um I have the book, what to expect when you're expecting, which one person told me to throw it away, another person was like oh you have to get this! You know so I know that there are different viewpoints on it.

INTERVIEWER: Mhm.

ADL07: And I think um you know the big thing with that, sh- the author always says, like keep in mind that no two pregnancies are alike, and you know…so its been, it's kind of been reassuring, it's been fun to kind of see like how is the baby growing? And what can I expect? And um okay this is normal, you know. And then I have a couple apps on my phone, pregnancy apps, so they like tell me how the baby is growing every week and you know, how my body's changing, and how the baby is changing, and stuff like that. So those have been helpful too with some of the little you know just symptoms and the side effects of being pregnant. Um, and then I think I've just like Google searched certain things, you know, we had a concern with the ultrasound and um the baby had chorioplexus cysts that were seen in the brain, and so you know I wasn't really sure what that was so I had to look that up, and…

INTERVIEWER: Mhm.

ADL07: You know in my research it sounded like it's not all that um uncommon, and that usually it's nothing, so that kind of (INTERVIEWER: Well that's…) helped to reassure me. And also food wise like I had to you know you, I was like okay well I can't drink caffeinated tea anymore. Well, I could potentially, I know you can have a little bit of caffeine but I decided just

to stop drinking all caffeine. So I was like well can I have herbal tea, so I was trying to look up which herbal teas are safe. And that was really confusing, so I was just like, I'm not gonna drink tea while I'm pregnant. Cuz it seemed like I could drink ginger, I drank ginger tea. And that was pretty much it. Because it was like one place I loved chamomile, okay another place no, chamomile's not okay, so um, yeah so Ill just kind of Google things. And then I've since slowly kind of added more like, everyday family has baby information, and they send me like daily emails or weekly emails about the baby's growth and development, stuff like that. And then there's the bump, I think that one too, I'm part of that, so.

INTERVIEWER: Mhm.

ADL07: There are a lot of things out there that send me emails about what to do and what not to do and. If I have questions like I can go and look at the little discussions and stuff like that so. That's usually pretty reassuring.

INTERVIEWER: Um for the apps…

ADL07: Mhm.

INTERVIEWER: Do you recall the names of the apps that you're using right now?

ADL07: I have…I have Pregnancy…and Baby Bump as one. And then I had sprout pregnancy, but that was just like a free trial and I ended up not buying it so it stopped at week 12.

INTERVIEWER: Um and did you join the apps right away when you found out that you were pregnant?

ADL07: Um, pretty soon after. Cuz actually I ended up getting a- the iPhone shortly after I found out I was pregnant.
27:48

INTERVIEWER: Mhm.

ADL07: My husband was kind of like alright you have to get the iPhone now. So um, and then I realized oh look they have all this useful stuff. Which is somewhat useful. Um so yea I guess it was pretty close after I found out I was pregnant.

INTERVIEWER: Mhm. Were there any that you joined and quit apart from the sprout pregnancy since that one was a, you had to pay for it?

ADL07: Um, no. cuz there were, the two other ones I usually look at weekly or deaily, like one of them has like daily updates and the other one is mostly just weekly updates.

INTERVIEWER: Mhm.

ADL07: So it’s fun, cuz Saturday morning is like when that’s when my new week starts so I’m always excited to see like, how big is the baby? And how much does it weigh? And how is it growing? And all that stuff so. I, I use them.

INTERVIEWER: Did those um apps and like email membership things

ADL07: Mhm.

INTERVIEWER: that you’re a part of, did they mostly talk about development of the baby?

ADL07: Pretty much.

INTERVIEWER: Okay.

ADL07: Yeah, it’s mostly like your baby is the size of some kind of produce, and this is how long it is, and then it’s like oh its eyes are you know, sensitive this week, or that sort of thing so.

INTERVIEWER: Not a lot on exercise or diet though?

ADL07: Mm…I’ve actually gotten more some of the like internet ones, probably like the Everyday Family, or the Bump, they have helped with kind of getting me back and exercising. And realizing that yeah, I really do need to start moving and tells you why exercise is important and stuff like that so. And they give you little exercise tips, and stuff like that, like easy exercises you can do or suggestions, stuff like that so they’ve been helpful in, in the exercise department.

INTERVIEWER: So what things have you learned about exercise during pregnancy so far?

ADL07: Um, I wasn’t really sure going into this, like how active I can be, should be, would be. And I hadn’t been, I used to be, like a super exercise person. I would run every day, and I would do like high impact aerobics and stuff. And then, I hadn’t quite figured, since we moved back to Rochester a couple of years ago, I hadn’t quite figured out to like fit in to my schedule on a regular basis.

INTERVIEWER: Mhm.

ADL07: So I’ve been very sporadic about exercising, other than just like walking the dog. Um and then I got pregnant, and then it was the middle of winter, and then I didn’t want to do anything. So, it’s been kind of you know, I’ve kind of gradually gotten back into, cuz I know that you know you shouldn’t just go from zero to 60 you know you’re not gonna be able to exercise the way that you maybe did before and that your heart rate shouldn’t get too high, and you shouldn’t overheat, and things like that. Um, and, so you know, but like stretching and and a little bit of s- you know exercise is good so. I’m sticking to like walking and like water aerobics, and yoga, things like that. I got a couple prenatal DVDs, joined some classes so. And it feels good to move. Again. Go. But it’s again you know I have friends who had like issues during their pregnancy so they couldn’t exercise at all.

INTERVIEWER: Mhm.

ADL07: And like my brother's wife, she was very conservative when it comes to exercising during pregnancy, but she was also pregnant with twins, and ended up in the hospital for a, like the last month of her pregnancy, so. I don't have a lot of like, pregnant exercising role models, I would say, so I didn't really know what should I be doing. And I think I kind of gave myself a little free pass in the beginning, like ah, you know you don't have to exercise you're fine.

INTERVIEWER: Um are there any exercises that you've heard are really great during pregnancy?

ADL07: Um it seems like walking is good. And then people keep you know I keep reading things about like prenatal yoga. Or water aerobics, like water exercises just kind of the low impact, things you know with a little bit of resistance, but they're not super strenuous and you're not gonna get injured or anything like that. Um and then obviously yoga to help with stretching and hopefully prevent back problems.

INTERVIEWER: And so how um much do you think that you're exercising now in comparison to before pregnancy?

ADL07: Um just recently I joined a class so I'm doing that like Monday nights. And then again since like the weather has gotten better and my energy is up, um, I don't know I mean I guess, it's hard to say cuz I was walking a lot before pregnancy…

INTERVIEWER: Mhm.

ADL07: but that was pretty much all I was doing. Um, but there'd be the occasional trip to the gym where I'd do some cardio and stuff like that. Um I don't know I mean at this point I guess maybe I'm exercising more, just cuz I'm really trying to put an effort into it and I've joined some classes, and like my mom's going to water aerobics with me tonight you know so.

INTERVIEWER: Aww that's fun.

ADL07: I'm trying. So yeah, I mean over the last couple of weeks, like I'm slo- you know gradually increasing my activity I guess, as I can, and I guess maybe it's more than I was doing before.

INTERVIEWER: Um have you heard of any benefits that exercise has for the baby?

ADL07: I did, I read and I have to keep reading to remind myself that exercise can actually like boost baby's brain power and things like that, so. I have heard that it is good for the baby. Cuz I don't know if it's like the increased blood flow, or something, I'm not really sure exactly what it's supposed to do but it makes sense. And it's good for me.

INTERVIEWER: Yeah.

ADL07: And our bodies were meant to move, so.

INTERVIEWER: Is, are reading things like motivating for you to…

ADL07: Yes.

INTERVIEWER:…get out there and.

ADL07: Yes.

INTERVIEWER: Cuz I know we all have days where we're like ugh, I just don't want to.

ADL07: Yes. Yeah. And for me especially like having like a set you know something set up where it's like I have a class I have to go to every Monday night, it's like okay I'm gonna show up you know.

INTERVIEWER: Mhm.

ADL07: And it's fun. And I like it, and I feel having it be I think like a specific pregnancy class helps too. Because it's it still feels a little awkward like being pregnancy like I don't wanna do too much, I don't want to do too little, I don't really know where I fit in, but knowing I'm surrounded by a group pregnant women it's kind of like a safe place to be to exercise. So.

INTERVIEWER: Mhm.

ADL07: And then I'm gonna go do water aerobics with senior citizens. So I figure that's pretty safe.

INTERVIEWER: Um if you had a question about exercise and weren't sure if something was safe or not safe, where would you go to look for more information?

ADL07: Um, I might s- like look online and search there. Um and I would also potentially talk to my midwife (INTERVIEWER: Mhm) you know. I figure they're gonna be the main people to ask if you have an important question.

INTERVIEWER: Are there any websites that you frequently go to, or do you just Google and then search through the listings that it gives you?

ADL07: Mmm…yeah if there's a specific question I usually just like type in that specific question and then kind of look at you know, sort through all the different answers.

INTERVIEWER: How do you decide which are the best answers?

ADL07: Depends on what I'm looking for. I don't know. Um, I guess if it comes from you know if it's just like a discussion or comments from someone else (INTERVIEWER: Mhm) I use those more just kind of in reassurance about certain things. But if I really need to know like, is this

safe, is this not safe, I probably look for more of, you know something that's like from a more reputable site. Or I go to a bigger kind of more medical website, or something like that, I guess I look for those for that kind of information.

INTERVIEWER: That makes sense. Um have you found any sites so far that always seem to be popping up at the top of the list for things?

ADL07: I don't know. Not that I can think of. And again, like I haven't really, I don't think I've looked anything up recently like specifically. So I basically just kind of click on the site you know they send me.

INTERVIEWER: Mhm.

ADL07: The daily whatever. The, you know, topic is. And I'll like click and look through that web-, you know the everyday family one or whatever like fit- oh I think I have like a Fit Pregnancy one as well. So that talks about exercise obviously all the time. Um and other things. So, usually I just kind of read through those things.

INTERVIEWER: Mhm.

ADL07: You know on a daily basis. Um, but yeah I think it's been a while since I've actually looked up anything specific. Um but I think yeah, the m- I don't know when I was looking at the chorioplexus cysts, I think I was looking up like, maybe I ended up getting information from like the Mayo Clinic or something you know one of the larger like medical institutions that usually have good patient information so.

INTERVIEWER: Um and have you heard of any specific recommendations for the amount of exercise that a pregnant woman should be doing?

ADL07: Um again, I think, you know I've seen stuff um kind of just on those like daily websites. And even I think on my phone apps where it recommends I think usually like about 30 minutes a day.

INTERVIEWER: Mhm.

ADL07: Somewhere around there. I think some of them have been different. Some are like, oh you should do 20 minutes twice a day, or um but yeah. I, I usually aim to for at least between 20 minutes and an hour.

INTERVIEWER: Mhm.

ADL07: Depending on what I'm doing so.

INTERVIEWER: And are there any activities that you've heard are not safe during pregnancy?

ADL07: Um, I think you have to use your judgment, and I not being like a super active person, am not gonna go join crossfit and do that, but I know someone who did crossfit throughout her whole pregnancy, and was fine. And again, I'm sure that was something that like you know she had been, that was her baseline, she had been doing it for a long time and, (INTERVIEWER: Mhm) her doctor okay'ed it. Um but I would guess that most people would not recommend doing any major, you know, intense workouts like that for just for risk of injury, and your body is changing and all that stuff. Um, I don't know, I mean I think…I don't know what else there is. Like I probably wouldn't do major high impact aerobics or riding a bike potentially, but again you know your center of gravity is different, your balance is off, so activities that can risk you like falling or something like that I probably would stay away from.

INTERVIEWER: That makes sense.

ADL07: So. And again, I don't think there's any, since I don't do anything going into pregnancy, I wasn't like going and looking to be like, oh is it safe that I continue hang gliding while I'm pregnant, you know? Like horseback riding. I might be nervous to horseback ride when I'm pregnant, but there are probably women who own horses, and they ride horses every day, and it's not big deal for them I don't know.

INTERVIEWER: Mhm.

ADL07: But I haven't looked to see if that's safe or not, I don't know.

INTERVIEWER: Have you felt like you wanted to exercise? I know that you said, like in the winter it was hard.

ADL07: Yes. Yeah, I definitely hit a point where it was like oh my gosh, all of a sudden energy is back, and I feel like I need to move again, and I actually kind of wanted to go running, but I'm not really comfortable with that at this point, just because I haven't been running.

INTERVIEWER: Mhm.

ADL07: Um, so walking is good, bouncing around a little in the pool, that's you know that works for now, and then hopefully I'll get back into running once the baby's born, but.

INTERVIEWER: Mhm!

ADL07: But yeah I've definitely felt like I need to move and exercise.

INTERVIEWER: And what benefits have you heard that there are for the pregnant mother rather than the baby, we already talked about that.

ADL07: Um, I think you know it sounds like from what I've read that I think that it can help maybe with prevention of gestational diabetes.

INTERVIEWER: Mhm.

ADL07: Um obviously it can help hopefully maintain a healthy weight gain, um, and I don't know if it does anything with preeclampsia or not. Um, but it certainly can't hurt I don't think. Um, and then I think just probably it helps you know if you're keeping somewhat active and in shape, it's gonna be easier to give birth hopefully (INTERVIEWER: Mhm) you know, keeps things fit and toned and flexible and stuff like that so. Hopefully less risk of birthing issues.

INTERVIEWER: Um. Was weight gain something that you've been…was that like a major concern, about something that you think about regularly during pregnancy?

ADL07: Um, clearly not enough cuz I still can't even keep the… I, you know, it's definitely something that I'm conscious of, um because I know that again, I wanna gain weight and I don't wanna gain too much. So it's definitely something like you know, going into pregnancy like I knew I was gonna gain weight and I'm fine with that. Um, and I wanna make sure that I gain weight cuz that means my baby's growing. Um but it's also you know there's some again, like that month that I gain too much and I was like oh, this could be bad. But it's not something that I think I think about all the time, and there's some days where I'm about to eat something bad and I'm like oh maybe I shouldn't do that. And there are other days where it's like whatever, you know, this is how it is. Um, so, I don't know did that answer your question?

INTERVIEWER: Mhm!

ADL07: Okay.

INTERVIEWER: Yeah. Um and then for after pregnancy is losing the weight something that you think a lot about now or is it not something that you're really worrying about?

ADL07: Not really worrying about it at this point. I mean, it's gonna be nice weather and I have a dog that needs to be walked, I have a gym membership, I have a baby that I'm gonna wanna to get out of the house with, so you know I figure um, it's definitely something that eventually I'm gonna wanna hopefully get back to my pre pregnancy size, but at this point I'm not really thinking about it too much or worried about it.

INTERVIEWER: That makes sense. Um is there anything else that we talked about along the way that maybe you thought of another response to that we could go back to?

ADL07: No, tonight I probably will. Laying in bed I'll be like, oh I forgot about that. Um, I don't think so.

INTERVIEWER: Do you have any questions about the study at all?

ADL07: Um yeah I guess, what are you, I mean I know probably in the beginning when I signed up for it, she told me a little bit about it, but um, what exactly like what is the goal exactly and…

INTERVIEWER: Um so our goal is to provide prenatal health information ranging from like emotional wellbeing to nutrition to fitness, and um, so we send messages usually six days a

week, it depends on the week, sometimes we do like weekly challenges so there might be less messages and it’s more about we’re looking for people to participate. Um, but our goal is to really change people’s attitude and beliefs about nutrition and fitness during pregnancy. But because it would maybe be boring to only talk about that for you know 9 months, or however long people are enrolled in this study, that um we try to break it up and add fun facts, and other sorts of things that ar-, we ho- think are motivating. And so our goal is kind of to see our messages motivating, did anyone try any of the ideas that we sent, um, the study was originally designed for pregnant adolescents (ADL07: Yes) and so this is… we recruited adults as a comparison group to see how adult women versus pregnant teens think about nutrition and fitness and I mean I think there’s probably some obvious expectations there. Um in terms of I think it’s, it’s a maturity thing too, and I think , maybe teens often think that they’re invincible cuz that age group that kind of goes along with it. So.

ADL07: When you’re still kind of a kid yourself and very self focused so it’s like you know.

INTERVIEWER: Yeah, yeah.

ADL07: Although sometimes, I feel that way, but I’m like this is my last chance to be selfish. But yes I mean a lot of what I do I’m still very focused on like how is it gonna affect the baby day to day so. And it’s good to keep hearing information like oh you need to exercise cuz it’s gonna help you and the baby. And it’s like oh yeah that’s right, and get off the couch.

INTERVIEWER: Yeah, I think that’s one thing that we were thinking when we said we’re gonna send 6 messages a week (ADL07: Mhm) , because if I feel like it’s easy you have so many things going on (ADL07: Yeah) it’s easy to get distracted so, with those constant reminders (ADL07: Mhm) maybe it’s the little voice in the back of your mind saying, oh yeah maybe I should get up and do something and do whatever. Um so that’s kind of our goal, um, so with the Facebook it was meant to be more of a social (ADL07: Mhm) environment where you know obviously you’re in a group with other women who are also pregnant. Um, I will say that we have not had like super great participation. It seems like most people participate passively, like you can see that you viewed the messages, but if you ask a question they don’t always respond so. Um, we’re just kind of feeling it out and seeing what happens.

ADL07: Okay.

INTERVIEWER: Um, which actually reminds of one other question I didn’t ask.

ADL07: Yeah!

INTERVIEWER: Um, have you ever heard of any of like text messaging programs that already exist for pregnant women?

ADL07: …No.

INTERVIEWER: Okay.

ADL07: Text messaging programs.

INTERVIEWER: Yeah.

ADL07: No, I don't think so.

INTERVIEWER: Just like cell phone apps, and I guess like the email things that you get, mostly are the things that you're familiar with?

ADL07: Yeah. Just the, yeah. The apps or like the websites that I have like a subscription to.

INTERVIEWER: Um I don't know, and did that answer your question, about…?

ADL07: Yeah!

INTERVIEWER: Um yeah so, mostly, it's a comparison group (ADL07: Mhm) to see how teens respond cuz I think a lot of things are targeted towards adult women (ADL07: Mhm), and so our goal is to eventually use all the information that we've gained to make something that really caters towards an adolescents' needs so.

ADL07: I did notice um in some of like one of the discussions, I think on the baby bump I just kind of you know looked at some of the discussion boards and stuff and I did notice that a lot of the discussion seemed to be kind of younger women, you know, 18, 19, um, with like their first pregnancies and you know they just had like a million questions about every symptom, and is the father still around, and you know, like I noticed there was like a lot of participation in those types of things from you know, I mean again, I don't know if their life, like I would say the majority that I saw were around 18, 19, so I didn't I don't know if there are any like 16, 17 year olds or anything like that, but um, but it seemed like they were kind of younger girls, that were participating in that sort of thing as opposed to you know, in their 30s. So.

INTERVIEWER: Yeah, it's different, and they're so young too that they grew up in like that social media world, (ADL07: Mhm) and I mean, yeah. I'm in my 30s too so I'm thinking about like, I mean we were in it (ADL07: Yeah) but not, not as in-depth as they were (ADL07: No.).

ADL07: Yeah, cuz I don't… I mean they're… I remember one of my coworkers started around the same time that I did at the job that I'm at. Currently she is like in her early 20s and she said that she remembered riding around the neighborhood on her bike with her sister, and like their cell phones, you know, in their little bike baskets or something. And I'm like I didn't have a cell phone until I was in college, and it was you know, I mean it was it's totally different now, that they're just completely plugged in all the time.

INTERVIEWER: Mhm.

ADL07: And I mean you know obviously people in their 30s, like I have my phone, that I lug around with me all the time, or I have a laptop computer or something, but um, yeah, it's interesting you know I don't know, cuz like my friends who are pregnant now. I don't know that

they really participate in the discussions so much, and seek advice from strangers, it’s like they seek advice from like their mothers and their sisters who’ve gone through it before and stuff like that so. I don’t know. Maybe that’s the difference.

INTERVIEWER: Yeah. I think something that I’ve definitely noticed is they don’t really talk to people (ADL07: Mhm) about things. It’s interesting.

ADL07: Uh huh.

ADL09

2:50
Interviewer: Do you have any questions before we start?

ADL09: Uh uh, no

Interviewer: Okay, so I guess we can go ahead and get started then, and hopefully we can get you out before an hour, [okay I know] so I know it's Friday

ADL09: Yeah it's most beautiful out today [garbled] where you are, are you, are you in [location redacted]?

Interviewer: No, I'm in [location redacted]

ADL09: Oh okay, well it's probably warm there too it's like 82 degrees here today,

Interviewer: Yeah, Yeah, it's gorgeous here, so yes outside would be nice, a nice place to be [laughs] .

ADL09: I have to work , whichever. [laughs]

Interviewer: Or that. [laughs] Um, so I generally start just by asking a couple questions to learn about you and your pregnancy um so the first question is how old are you?

ADL09: Uh, thirty-five

Interviewer: Okay, and how tall are you

ADL09: Five-two

Interviewer: And how much do you weigh right now

ADL09: Uh, I think it was 161.8 today, or 162

Interviewer: Um, and is this your first pregnancy?

ADL09: No, this is my, let's see I've had three miscarriages and two healthy and this one, so this is my sixth pregnancy

Interviewer: Okay, and then this will be your third child?

ADL09: Yes

Interviewer: Okay [pause] And was this pregnancy planned?

ADL09: Yes

Interviewer: Okay. And, what hopes and dreams do you have for your unborn child?

ADL09: Oh geez

Interviewer: [laughs] That's a loaded question

ADL09: It's a good question. Well first of all I hope the baby is healthy and stays healthy and um I say it's happy and um successful at whatever they want to do in their life. Just a good person; I just want my kids to be good people

Interviewer: Those are all wonderful wishes, uh…

ADL09: Pretty general but

Interviewer: No, I think that's pretty standard

ADL09: I have two at home so I can't be too specific cause I, they're all so different so

Interviewer: That's true

ADL09: Yeah, as long as they're good kids.

Interviewer: Um have you had any challenges that you've faced with this pregnancy specifically?

ADL09: Um no actually this has been my smoothest pregnancy yet, so I am grateful for that

Interviewer: Good, that's awesome to hear, and um what do you think are the most important things that any woman should be doing to ensure that she has a healthy pregnancy?

ADL09: Um, to take care of themselves, um you know eating as healthy as they can and not, you know, abstaining from alcohol or drugs, smoking um, you know trying to stay as stress free as you can, mm hmm.

Interviewer: Mmm hmm.

ADL09: I have three kids and working, so that doesn't always happen but, um, you know that's it, I think

Interviewer: Um and so when you have a, I know you have other children so you can maybe pull from previous experiences too, um but where do you normally go for advice or information about pregnancy related questions?

ADL09: Um, usually my friends, who've had kids, or are pregnant, or have been pregnant. Um this pregnancy I'm actually on a couple of um pregnancy boards, like, Birth Center Board and um What To Expect board so I've been going to those um online if I'm, I want to know if my heart rate goes low or high I'll look it up onGgoogle or um actually you know the practice I'm at the [midwifery group redacted]…I can email them and they've pretty good about emailing me back right away if I have questions so that's been really helpful

Interviewer: And when you Google a question, how do you decide which of the resources that come up to look at?

ADL09: Um I'll look and see if I recognize the name of the website first from previous posts or from previous, I don't know, Google site or Google searches I've done ….if they're reputable, you know, otherwise , um I usually go to the ones that come up first, cause those usually have been the ones that have been looked at the most.

Interviewer: And where do you typically access the Internet from?

ADL09: On my iPhone.

Interviewer: Ok. And do you also have a home computer with Internet?

ADL09: Um we have laptops, but we, I mean, I can't remember the last time I went on them.

Interviewer: Ok.

ADL09: I also have an iPad at home, too.

Interviewer: And do you use that fairly frequently?

ADL09: I do, yeah you know I'll have a laptop at work that I'll use, during work time [laughs].

Interviewer: I totally do the same thing. [laughs] Um, do you recall what some of your more recent searches were about?

ADL09: Yeah I looked up what type of, so, I have a home Doppler, so I want to know what the heart rate was, it was lower than it had been in the past, so I looked that up. Normal heart rate for seventeen weeks.

Interviewer: And does that measure your heart rate or the baby's?

ADL09: Uh the baby's heart rate, so I have a fetal Doppler that I—that I use.

Interviewer: Ok. Um and any other searches that you can think of?

ADL09: Um, no not really. Maternity clothes but that doesn't really count [laughs].

Interviewer: It’s fun to shop online though.

ADL09: It is.

Interviewer: Um, let’s see. Um, are there any sites that, if you’re not Googling, what the question that you might go to because you feel that they’re a reputable website?

ADL09: Yeah I’ll go to BabyCenter, mostly. Right this is all related to my pregnancy, right?

Interviewer: Uh huh.

ADL09: Yeah. BabyCenter.

Interviewer: Ok. And do you usually visit the forums on BabyCenter, or try to read articles, or, a combination.

ADL09: Um, probably a combination, but I’m more of just, I’m one of those stalkers that just reads all the posts, you know, I don’t necessarily post anything ever but I just read to roam.

Interviewer: Mm hmm. Um and so when you read forum posts like that, how do you decide, you know, which things are good, which things are not good, you know it could be a challenge.

ADL09: Um, I think because in, this, this not being a first time mom, I’m like, no, you did not feel the baby move at 12 weeks, like that kind of stuff, I’m like, whatever, it’s just kind of um, I don’t know, ignore? Or read them but I’m like, that’s not true, so like, as a first time mom, I probably would’ve been like (gasp) you know, I’m 18 weeks I haven’t felt the baby yet, what’s wrong, so that kind of stuff, I think, I’ve done and um if they’re helpful, or if they’re things I’m kind of going through myself, or, um deciding to get the fetal Doppler, I did go on there and, kind of, see what other moms have said, so I used it for that purpose too.

Interviewer: So do you feel that, when you’re using resources on BabyCenter, is it more about finding that, affirmation that your feelings are similar to other women or is it more that you’re looking for information on the topic?

ADL09: Um, probably a little of both, although I mostly just do it for entertainment. Honestly, if I have a, I mean, my coworker just had a baby, so I’ve, we've, we’re in the same boat and she had two kids as well, so I feel a lot of my questions I just will talk to her about, or text her at night, you know, oh, you know, I’ve finally felt the baby move, or it’s just this turns normal, or that kind of stuff. Or my levels were this, is that normal, that kind of stuff I will talk to her about, and but in the beginning I did do a lot of Google searches on HCG levels, because I’ve had 3 miscarriages, so I felt like I kinda use that a lot to gauge where I was at.

Interviewer: Uh huh. So it sounds like you have a really good friend support, and your doctor’s office has the nice aspect of letting you e-mail in answering questions. What role does your family play in preparing for pregnancy and raising a baby?

ADL09: Um well my mom watches my kids once a week so she's always at my house to talk to. Um my sister has only had one and she's younger than me, so I don't typically go to her for advice, uh on being pregnant, but they're all really supportive, like, they're all happy, so my sister-in-law just had a miscarriage, so we can kind of talk about that, you know, that's supportive, but , yeah, they're pretty helpful.

Interviewer: Um and is your current cellphone your own personal phone, or is it a shared phone?

ADL09: No, it's mine.

Interviewer: Ok. And it has a data plan as well as text messaging, is that included in the package?

ADL09: Yes

Interviewer: Ok. And in addition to the BabyCenter and What To Expect that you mentioned, um have you joined any email, text message, twitter, blogger, Facebook groups?

ADL09: Uh, well I'm trying to get some formula and diapers, so I've gone to the Pampers , huggies, similac, and enphamil websites to join theirs, but I don't do anything on those websites, I'm just waiting for coupons and samples to be honest

Interviewer: Well that would definitely be helpful.

ADL09: Yeah, I mean sometimes they'll email me, like, 18 weeks, What to Expect, and I'll kind of browse up on it but it's the same information I'll get on BabyCenter or whatever, so.

Interviewer: Mm hmm. Uh, have you heard of any Twitter or text messaging programs for pregnant women?

ADL09: No, well I'm not on Twitter, so I wouldn't know about that one, but text messaging, no, mm mmm.

Interviewer: Ok. Um and then, what about Facebook groups?

ADL09: Um none that are, like, pregnancy related, but I do, I'm on like, mom swap board, to buy and sell kid stuff, and uh sometimes they'll ask for advice or questions, and sometimes you can put a question out there to them but it's not specific to being pregnant.

Interviewer: Ok. And how often do you receive information from BabyCenter and what to expect?

ADL09: Um, I get weekly updates. Like, "you're at week eighteen!" That kind of stuff.

Interviewer: And, given all the resources that you have told me about, if you had to pick one person or one place like an internet site, which would you say would provide you with the best information on pregnancy?

14:47

ADL09: Probably BabyCenter.

Interviewer: Okay. Um, do you feel Babycenter offers good information about prenatal care in terms of nutrition and fitness and weight gain?

ADL09: Um, yeah I think it's on there. I don't necessarily go to it for those kind of questions, but mostly I just want to see what the baby looks like at this point, what's developing, I've been through it before, so I kind of know what my doctors have told me. My first pregnancy I was all over what to eat, what not eat. But now I've been over that, I kind of just know what not to eat, but before I got pregnant I was working hard on losing weight so I kind of know what's healthy to eat, that kind of stuff, so not necessarily all the time, so I had the baseline for it so I didn't need to look that up, necessarily… But it's on there, if I want the information, I think.

Interviewer: Um, have you been given any advice about weight gain during your pregnancy?

ADL09: Actually I just talked to the midwife about it today, cause I haven't gained any weight yet, so I was asking her how much I should gain, cause I was, I might have a high BMI before I got pregnant, I was like 27. So I'm trying… I just want to see how much she would recommend me to gain throughout the rest of my pregnancy.

Interviewer: And what was the recommendation that you were given?

ADL09: She said twenty pounds, give or take a few.

Interviewer: And do you feel like that that's a good amount for you?

ADL09: Uh, yeah I think with my other pregnancies I probably gained, I don't, I don't really remember but I think between fifteen and thirty-five pounds I gained, so I feel like it's probably realistic at this point. Cause I feel like with my other pregnancies I gained earlier than I have with this one, so I don't know what that means, I will all of a sudden, like, blow up. I also, um, am a runner, so I do exercise, obviously not anywhere near where I was before, but I felt like my body was in better shape with this pregnancy, getting pregnant, than I was with the other two. Hopefully that helps.

Interviewer: Um and what role do you think that diet and exercise play in maintaining weight gain throughout pregnancy?

ADL09: Um, well I wouldn't go get a Whopper every day, you know, that's not healthy. Um, I don't have cravings, like I wanted to eat, or it's fruit, so that's actually good, but, wait what was the question again? I feel like I went totally off tangent with that.

Interviewer: Um, no, so the question was what role do you think that nutrition and fitness play in managing the weight gain within the recommendation?

ADL09: Yeah I think you can't just lay on the couch all day, you know, and then not expect to gain a lot of weight and eat ice cream, a bowl of ice cream every night, so I think everything in moderation. You know, you have to take in more calories than you normally would, being pregnant, but that's not a reason to eat a bowl of ice cream every night either, so.

Interviewer: Right.

ADL09: I do… I have pulled the pregnancy card a couple of times, but not very often.

Interviewer: Um, have you ever heard of any sort of negative impacts that weight gain can have on either you or the baby?

ADL09: Um, just that diabetes, gestational diabetes, and um, like high birth weight. Or not eating enough, low birth weight, for babies.

Interviewer: Mmm hmm. Um and you mentioned you weren't craving a lot during this pregnancy, but in your previous two pregnancies that you carried full to term, did you have weird cravings at all, or…

ADL09: Umm, I don't know if I craved them, or if I just allowed myself to eat more of the food, um, I don't think so, I'm trying to think. I don't know I feel like it was almost five years ago when I had my four year old, so honestly I don't, I don't really remember. I just know but this one, it's fruit. I just really want fruit. Watermelon in particular. I don't know why, but I don't have any specific cravings. Maybe salty foods.

Interviewer: And in terms of cravings, do you feel like those are telling you something specific about you or the baby, or what you need or anything like that?

ADL09: Um. No I mean I know it's a little old wives' tales like, oh if you're craving salty that means boy if you're craving sweet that means girl or whatever, but I know, in far as lacking vitamins and minerals, I don't think so. Unless you're eating dirt, them maybe you're craving, you know, or your body is needing a mineral, but, no, I don't know.

Interviewer: So no weird cravings or anything like that?

ADL09: No.

Interviewer: What about smells? Are there any smells that you like a lot more or less?

ADL09: Umm…. I think that this point I'm a lot better, when I was first pregnant at this time, um like cigarette smoke, although that bothered me all the time, but I feel like I'm even more so turned off from it. Uh, my dog, I hate, can't stand, be around the dog smells, like that wet dog smell after a rain or something. That, I'm really sensitive to that this time around, uh, but that's it, I think.

Interviewer: Um, and who typically prepares the meals that you eat?

ADL09: Um, I would say my husband is the typical cook in our house, but if I don't like what he's making, I'll just make my own dinner.

Interviewer: And who does the shopping for most of the groceries?

ADL09: Um, my husband, unless I'm like "oh I really want some mango today" and then I'll stop by and get random stuff, but he's in charge of grocery shopping.

Interviewer: And, does everyone in the household provide, or offer suggestions when making decisions about what groceries to buy?

ADL09: Um, well we kind of know what the kids like to eat, so we try to cater to that a little bit for snacks and stuff, but we'll make decisions on meals, and they'll either eat what we fix, or they don't eat. But we don't, we also don't make crazy meals, either, you know. We have tacos every single week, so they know what to expect with some, a little bit of variation but not a lot.

Interviewer: Mm hmm. And how much money do you think your household spends each week on groceries?

ADL09: So we're on a budget for groceries, and our budget is $150 a week.

Interviewer: Okay.

ADL09: And we also have a 13-year-old stepdaughter at home, who's with us full-time. So, I don't know if that factored into any of this, but.

Interviewer: Um, and so, thinking through all of the pregnancies you've had, what modifications did you specifically make because you knew that you were pregnant to your diet?

ADL09: Um, really nothing, I don't think, this time around. I mean I feel like I was eating healthier, uhm so I haven't made any modifications yet, this time. I mean, when I was first pregnant, I mean, the first eleven weeks or so I was nauseous at night, so I typically didn't eat any dinner, um or if I had dinner, I ate it earlier and then I couldn't eat for the rest of the night. So that, I had to modify my diet for that, but. I'm more aware that I need to eat, cause I'm on the road a lot for my job, so sometimes it's easier to be like "ugh, no time to eat", so I just won't get like anything, and now I'm like, no, you're pregnant, you have to eat something, so. I modified it now a little bit, but other than that, no not really.

Interviewer: And, what do you typically eat when you have those times when you need a snack?

ADL09: Um, wherever the fastest fast food place is. Like, I haven't had lunch yet today, so I'm really wanting some Taco Bell tacos and Wendy's French fries, which are not great, but that's what I want right now, so., that's what I'll grab. Or sometimes I'll have, in the morning, I'll put some things in my purse, like an apple or a banana, or whatever snacks we have in the house I'll

throw ‘em in my bag and eat them during the day, but. I’ll go to, I have been eating more Wendy’s lately, so.

Interviewer: I love the spicy chicken sandwich; that’s my favorite from there.

ADL09: It’s so good, I know. Their new fries are really good, I don’t know if you, I love their lemonades, their lemonade is really good too, but.

Interviewer: Oh I haven’t had that. I don’t know if I’ve had the new fries yet, either. But its been a while since I’ve had it, so I’ve.

ADL09: They’re good.

Interviewer: We don’t have a Wendy’s in [location redacted], it’s very unfortunate.

ADL09: That’s a little good and bad, I guess. [laughs] I’m lucky I don’t have a Chick-fil-A ‘cause I’d be there every day, so.

Interviewer: So um are there any food that you’ve actively stopped eating because you knew they were not good for pregnancy?

ADL09:, I stopped eating tuna fish for a little while, but I have gotten a couple of tuna subs from subway over the past month or so. But I don’t eat a lot of, I don’t eat any shellfish anyway, so I don’t have a lot of seafood, uh, I don’t eat any of those fancy cheeses, so I haven’t done that. I don’t eat lunch meat, so I haven’t had lunch meat, but that’s not a change for me, so. So not really. I told you, we don’t have a lot of variation in our diet.

Interviewer: That could be a good thing, though. It makes it (garbled). Um, do you think that you’re hungrier now that you’re pregnant, or do you have less of an appetite?

ADL09: I think I have less of an appetite, well, I think it’s changing. Um, I’ve definitely have had less of an appetite since I’ve been pregnant than before, um, but I get full really fast now though, but the past week I’ve noticed I’m definitely hungry. Like right now, I’m starving.

Interviewer: Well I’ll try to go fast.

ADL09: I haven’t felt hungry in a long time, so. This week has been kind of nice to start feeling hungry again, so. Good and bad, I guess.

Interviewer: Um, let’s see. And I know that you mentioned that in your previous pregnancies you heard a lot about good things to eat for the baby, bad things to eat for the baby, uhm so can you tell me a little bit about the things that you know might be good for the baby and why, and bad for the baby and why?

ADL09: Well good. I know, well I don't know if it changes a lot when you're pregnant, what's good to eat, you know. I think eating a lot of fruits and vegetables, protein is critical, uhm, I don't know, just not eating a lot of crap, you know?

Interviewer: Mm hmm.

ADL09: Fast food. Uh, anything that wouldn't be good for you not being pregnant, wouldn't be good being pregnant. The bad stuff I think, I guess would be, the bad things I think I heard would be shellfish I think, a lot of tuna fish, lunch meat, I think there was certain cheeses you're not supposed to eat. I don't know.

27:34
Interviewer: And do you know why they recommended not to eat those?

ADL09: I know that mercury, and the tuna fish, and fish, but I don't know about, and unpasteurized things you're not supposed to eat, lyseria I guess? I don't know. I don't pay too much attention because I don't eat those foods, so I guess.

Interviewer: That makes sense.

ADL09: I'm naïve about them because I don't eat them, so.

Interviewer: Um and have you changed how you're preparing these foods? And so, or, foods that you might choose at a restaurant now that you're pregnant, you know fried versus baked or grilled, and those sorts of things.

ADL09: No, I just get whatever I'm hungry for.

Interviewer: Um and, have your interests in eating salty or sugary foods changed at all?

ADL09: Um, I guess a little bit more sugary because I've been eating a lot more fruit. Um, and when I choose cereal I've been choosing more, like, sweet cereals. So, I guess, I guess, maybe I don't know, cause at night I've been eating pickles, or I'll have, uhm, I don't know, I'm not a potato chip fan anyways but uhm, so I guess not really, a little bit of both.
Sorry that's like, that's kind of confusing.

Interviewer: No, that makes sense. Um, and what's your dairy consumption like?

ADL09: Um, I don't like milk. So I don't actually drink milk, uhm. Sometimes I'll have smoothies with like, um the soymilk, vanilla soymilk, and in my cereal, I just have it for the cereal, I don't drink the milk afterwards. If you can count ice cream and cheese, I do that for dairy. I haven't really had a lot of yogurt since I've been pregnant . So I guess no, not a lot of dairy.

Interviewer: Do you normally eat more yogurt?

ADL09: Mm, not really. Yogurt's hard for me to (garbled) like some kinds of it, so I normally put it in my smoothies than eat it plain.

Interviewer: And, do you know what is it about milk that you don't like?

ADL09: I don't know, you know, that really, I don't know if I have ever liked it. I just can't imagine sitting here having a glass of milk. I don't like it.

Interviewer: Um and have you ever heard that, anything about a pregnant woman needing to change how or what she eats as her pregnancy progresses?

ADL09: I think what I've heard is you have to keep, be careful about your weight gain.
And if you have, if you do have gestational diabetes, you have to really be on top of your carb consumptions, your grains, that kind of stuff.

Interviewer: Have you received any advice from friends or family on things you should eat or shouldn't eat?

ADL09: Um, not really. In the beginning they'd be all I need to eat more because in the beginning, I'm always getting full or I'm always feeling nauseous, but other than that not really.

Interviewer: Um, and are there any foods you have to buy because you feel that they have extra nutrients in them, that would be good for you or the baby?

31:42
ADL09: Um, no.

Interviewer: Then, are you taking any vitamin pills?

ADL09: Um, I'm reaaally bad at taking my prenatal vitamins. But I do have them.

Interviewer. Okay. And any other supplements along with the prenatal that you have?

ADL09: No, mm mm (no).

Interviewer. Okay. Um if you had a question about nutrition or food, food-specific food items, where would you go to find information?

ADL09: Um, if I needed to know quick, I would just ask, I would go online and Google it. If it was just a question I had I would wait for my appointments and ask my midwife about it. Or email her I guess. Or if I go out to dinner and I wanted to know "oh, can I have that? Can I have that?" I would just ask whoever I was with if they knew, I guess.

Interviewer: Um and so I know you mentioned in the past you looked up more of what to eat, what not to eat, um but, you're more experienced now so it's probably less of an issue for this

pregnancy, but thinking back to when you did look things up where were you looking for that information?

ADL09: Um, I probably just Googled it. Or it came in the emails that I got about being pregnant, off BabyCenter, cause I've always signed up for those weekly updates.

Interviewer: Um, and is nutrition something that you frequently look up questions about, or--

ADL09: No.

Interviewer: --or more of the development that you had mentioned earlier.

ADL09: Yeah, mm-hmm

Interviewer: Uhm, so earlier that you mentioned that you enjoy running, so I did want to talk to you a little about uhm exercise or fitness, and so I'm just curious what your activity level's been like during this pregnancy and how it differs from before, and any changes.

ADL09: Yeah, a lot of changes. I was going to boot camp 5 days a week before I got pregnant. And um, I was running a lot. I ran a half marathon. Um so I was training for that. And I've been doing a lot of 5Ks. So when I got pregnant, my goal was that, oh, I'm going to to keep running, I'm going to keep running. I was soooo tired that I had no energy to run. So I've only actually run 3 times since I've been pregnant. My goal now, um, is to, um walk a race. More so to keep my body healthy but I also want to have a VBAC. So I want to keep my body in the best shape to do that.

Interviewer: How do you stay motivated to go walking each morning?

ALD09: Well I haven't started yet. I was waiting for the weather to be warmer so hopefully that means now, starting now, so. Um, I'm hoping to be motivated just because it makes me feel better. And I know that from running before, that working out makes me feel better, so.

Interviewer: Mm hmm, and what about when you were doing the boot camp and training for your half marathon, where there people or things that kept you motivated to go?

ALD09: Uh, at boot camp, yeah the people I would go with were very motivated. If you didn't show up you got a lot of slack for it the next time. Um and mostly myself because it felt, good, like I felt good about myself. I was actually noticing differences in my body, so that was motivating all by itself. I would say my family was definitely supportive of it, it kinda gets in the way cause I'm gone a lot, running and boot camp, but they're as supportive as they could be while he had to stay home with the kids, you know? So.

Interviewer: Yeah. Um, have you heard of any specific recommendations on fitness for pregnant women?

ADL09: Um yeah I've heard, I mean I've heard just everything in moderation. So they keep telling do… you can do what you can as long as you feel okay. So if you start feeling off, then you need to stop. That's your body telling you to stop, so mostly what I've gotten is to follow your body's, um, views. But the exercise is good, while you're pregnant, to do.

36:39
Interviewer: Um and what reasons have you heard on why it's good during pregnancy?

ADL09: Uh, I think cause it keeps you from gaining too much weight, um, it keeps you in better shape for the baby, for labor. It's easier to lose weight afterwards if you stay in shape while you're pregnant. Those are what I've come up with.

Interviewer: And, have you ever heard of things that a woman should not do during pregnancy?

ADL09: Yeah, things that you could fall like, bike riding or horseback riding, um, that kind of stuff, so there's a risk of falling, I mean, I wouldn't go running on a trail in the middle of the woods, you know. Not tripping over, you know, roots and stuff, but. But yeah anywhere you could fall and, like, injure your stomach and the baby I've been told not to do.

Interviewer: Um, and have you received any advice from friends, family, doctors about exercise during pregnancy?

ADL09: Um yeah they've all encouraged me to do so. I mean, some of my family is like, you shouldn't be running, you shouldn't be doing this, blah blah blah, but. That's old school, my grandma, mostly. Other than that, of course here, they've been really supportive of exercising and getting your body in shape.

Interviewer: Um and if you wanted to know more about a specific exercise or anything in relation to fitness, where would you go for information?

38:23
ADL09: Um I would probably Google it, or even fo a post on one of the forums. Or I'd email one of the nurses or the midwives here about it. And I also work in health care, so I work with a lot of doctors and nurses that I can you know ask questions to if I need to as well.

Interviewer: Well that sounds great.

ADL09: Well I mean yeah, they don't work in OB/GYN but, you know, they would have some of that training.

Interviewer: Um, so earlier you said that you've run three times since you've became pregnant, and you have the goal of walking each morning, so currently what does your daily activity look like?

ADL09: Well, I work, I work, I do a lot of home visits, and I work with kids, I'm a child specialist, so I'm constantly in and out of my car, getting stuff out of my car, you know, up and

off the floor, working with kids, uh. Not heavy lifting, but I'm lifting and carrying bags, and that kind of stuff. I mean I feel like it's pretty active, I mean I'm not not running at my job, but I'm definitely not sitting down.

Interviewer: Um and are there any activities that you most enjoy doing?

ADL09: Um, I really like to, while I'm pregnant you mean?

Interviewer: Um, no just in general.

ADL09: Oh. Like specific activities, like I really like the bootcamp classes. Like flipping tires and that stuff, I love it.

Interviewer: Is that a very team-oriented activity?

ADL09: No, I mean people are supporting you, but you're kind of doing your own thing during it.

Interviewer: Uh, and so I know another thing you mentioned earlier was you weren't running as much as you wanted because you felt tired? Do you know why some women feel tired early in pregnancy, have you heard about that?

ADL09: Well I think, I mean, you have something growing in you, so it takes some energy to do that from you know, whatever else you're doing. Uhm, for me it was partly because I wasn't eating a lot at night so I felt nauseous, so I never threw up but I felt sick to my stomach, you feel like you might throw up but you don't. Uhm so I think I probably didn't have enough energy to do that. (mumbles)

Interviewer: Um and if you could add a quantity to the amount of exercise you did before pregnancy to now, how would those numbers compare?

ADL09: Like percentage wise or?

Interviewer: Um I was thinking like, hours per day, maybe.

ADL09: Um, my work is the same, I'm the same active level at work, that hasn't changed. But after work I'm more apt to be on the couch, watching tv or being with my kids than like playing baseball with them or running around in the yard so , I don't know, I think then I worked out 5 days a week that was an hour I was active that I'm not now, that helps answer the question? I don't know.

Interviewer: Yeah, um now that it's getting nicer out, do you think you'll be out and about with your other two children?

ADL09: Yeah, well they love to go for bike rides so obviously I will walk beside them, but they also um, play sometimes baseball, so you know, we're at the game, and I'm helping them to get

ready and throwing the ball around with them or something like that, so yeah definitely I will be a lot more active. Cause in the winter, I don't know, I don't even like to run, be outside in the winter at all. Like, I don't even like to be outside with my kids in the winter so. Now that it's nice out, its definitely more motivating to be outside and be active.

Interviewer: I definitely agree. It was a long winter out here.

ADL09: Yeah it was. Really long.

Interviewer: Uh, are there any effects that you heard physical activity can have on the baby's development?

ADL09: Um not really, I'm assuming if you're healthy and your body's healthyit's going to be more likely you could have a healthy baby. I mean, obviously, I could be the healthiest person in the world and I could still have a baby with a heart defect or something like that but, I mean I'm sure there's reasons why, but I don't know them. Maybe action levels, I don't know.

Interviewer: And so would you say when you choose to do something for exercise, you do it more because it makes you feel good, or its less driven on effects it would have on the baby?

ADL09: yeah.

Interviewer: Ok.

ADL09: So. Selfish, I think. But yeah. No I can't give you a direct correlation I just assume that if its good for me, it's gonna be good for the baby, so.

Interviewer: I think that makes sense.

ADL09: Okay.

Interviewer: I'm just looking for internal motivators.

ADL09: Okay.

Interviewer: Um, cause I know some people have a hard time making changes so if you understand what motivates someone then you can focus your messages towards that, so.

ADL09: That makes sense.

Interviewer: That's why I'm getting super nitpicky so I apologize.

ADL09: It's okay.

Interviewer: Um, so let's see, I think we actually talked about that, have you heard of any exercises that are good for preparing for labor or delivery or…

ADL09: Yeah yoga and squats. Two of my least favorite things to do.

Interviewer: [laughs] Um I think those are actually all the questions. We went through those really fast. Um so are there any answers to any question that I had asked earlier that maybe you thought of after the fact and we can go back to and discuss a little more?

ADL09: Um, don't think so. Mmm mm (no).

Interviewer: Okay. Um well, do you have any questions for me about the study at all?

ADL09: Um no but I'm realizing the more I'm getting asked these questions the less I know, so is there any information booklet or is this just a study for you to find out what we do or don't know.

Interviewer: Um so the first phase is just to kind of see, like, baseline, where you are, what things you've heard, what things you might be trying, um or believe are healthy or not healthy. And then, um, so the next phase you'll get messages on Facebook and through text message um and we send 6 messages a day through each platform so you had signed up for both so you'd potentially be getting 12 messages a day. And so our goal is to share information about weight gain, um creative ideas to be healthy in terms of diet and exercise, um things that might be easy to fit into busy schedules cause a lot of people have always had busy schedules these days, um and we try to , like, mix-match lots of things cause it gets boring if it's just like, eat healthy eat healthy eat healthy. Um so we also talk a lot about mental health, and you know like, if you have baby blues, like things you could do for that, or so it's kind of a hodgepodge of all sorts of things but we just try to share interesting facts, anything that's out there in recent research that you might find interesting, we share links to Youtube videos, websites, that kind of stuff, so now it's the educational part of the study, and towards the end, at the end of your pregnancy we interview you again, and then that's geared less toward seeing what you think about nutrition and health and what you're doing, but more to see like what about the messages made you change something, or how you thought about something , and um, if they were written in a good way that kind of stuff, so. That's the plan, um, any other questions?

ADL09: Um, no I don't think so.

Interviewer: Okay. Well thank you so so much for taking the time out of your day for this. I really appreciate it.

ADL09: Thank you.

Interviewer: I will probably start sending messages this evening, if I can't find you on Facebook I'll just send you a text and let you know and maybe you could look for me instead. Sometimes people have really rigorous privacy settings so they're hard to find, but, I will double check and then let you know but otherwise you'll just start getting messages, and um there will be a couple diet records that [name redacted] will do with you along the way and then we'll talk in a couple months.

ADL09: Okay, great. Thanks.

Interviewer: Yup. Have a nice weekend.

ADL09: You too. Thanks.

Interviewer: Bye.

ADL10

Interviewer: The plan for today is to do your 1st interview and the goal is just to kind of see what you think about prenatal care in terms of nutrition and exercise, where you look for information especially on the internet, whether you joined groups or text messaging programs or apps or anything like that on your cell phone. There is no right or wrong answer. Anything that you say, I just want to know what you think, don't be shy. If you don't think I asked a question or asked it, feel free to be like whoa I didn't get that and I'll say it again. Cause sometimes I get excited and I don't make any sense. Do you have any questions before we start?

ALD10: no

Interviewer: okay well I will hit record and we will get started, okay, I wanted to start by getting to know a little about you some basic questions to describe you and your pregnancy and get to know you and your baby. So the first question is how old are
you?

ALD10: 23

Interviewer: okay, and how tall are you?

ALD10: 5'4" and a half

Interviewer: Ok, and how much do you weigh?

ALD10: the last time I just went today, 210

Interviewer: okay, and is this your first pregnancy?

ALD10: yes

Interviewer: oOay, and the next question is we like to try to gauge what people if they were planning for pregnancy so if you're uncomfortable that's fine. I was just wondering if this pregnancy was planned?

ALD10: um no

Interviewer: have you planned to have children in the future?

ALD10: yes, I just didn't think it was possible

Interviewer: how so?

ALD10: because the partner that I have like before we talked about having babies, when I thought about it, it was never happening, then it happened and it happened

Interviewer: I think that things happen when you least expect I definitely think that. So um what hopes and goals do you have for your unborn child?

ALD10: umm (breaks up) I don't know I don't really think about stuff like that

Interviewer: do you ever imagine what things will be like when she's a little older and able to waddle and talk?

ALD10: it's a he

Interviewer: sorry

ALD10: I don't think too much about the long run I think about the baby phase like when he first talks or walk and stuff like that

Interviewer: and how does that make you feel?

ALD10: good, happy, anxious

Interviewer: how come um is there one big milestone that you're most exited about witnessing?

ALD10: hmm just the birth I'm most excited about that time and just the first steps and being able to talk to him

Interviewer: and um do you feel like you have a pretty good support network to help you in preparing for the things you feel anxious about?

ALD10: Yes

Interviewer: could you tell me a little about the people you have in your support network, what kinds of things you talk about?

ALD10: well um, my baby's father, my aunt, and we just talk about the things I just was telling you, not so much I think about it all day (breaking up)

Interviewer: mhmm do you feel less anxious when you talk to them about things?

ALD10: um yes

Interviewer: and so given the things that you're looking forward and some of the things that you're nervous about, what are the things have you been thinking about or have you been doing that you think are important to having a healthy baby

ALD10: um, you mean on a basis or what do you mean by that?

Interviewer: I guess the questions lead into things you may have changed once you found out you were pregnant

ALD10: I didn’t really change too many things, I guess cause I never like vegetables I just something I eat that I changed other than that my daily routine is the same I work come home and am doing the same things I’ve been doing for a while

Interviewer: um you said that you changed some of the things that you eat could you tell me a little about that

ALD10: um like the vegetables, I never liked them before so I try to eat more vegetables now and I try to eat more fruit and milk and stuff I never really drank those types of things I do now

Interviewer: could you give me an idea of how many vegetables you ate before and how many you eat now?

ALD10: um I didn’t eat any before daily before at all but now I eat them daily, I never ate like vegetables I only ate like broccoli

Interviewer: and you said that you mostly eat broccoli

ALD10: mhmm

Interviewer: okay and is that a vegetable that you do like

ALD10: oh yeah I like broccoli a lot

Interviewer: do you have a favorite way of preparing it?

ALD10: no I just put a little water and salt and like boil it

Interviewer: and are there any other vegetables that you’ve tried while pregnant that you did or didn’t like but you gave them a shot

ALD10: I’m sorry the camera kind of broke down

Interviewer: oh okay um you mentioned that you’ve eaten a lot of broccoli are there any other vegetables that you’ve tried while pregnant that you found out that you did or didn’t like

ALD10: um I don’t like carrots, I never liked (breaking up) and I don’t really like salad probably because I don’t put the right things in it, um I don’t like peas, yeah I really only like broccoli

Interviewer: and what about fruit

ALD10: I like fruit I like pineapples and apples, um and strawberries um, what else, oranges, um yeah those are the majority of fruits I eat are apples and pineapples and oranges and mixed fruit and I like peaches and pears and I just eat a lot of mixed fruit

Interviewer: are those things that are from the little cups or cans?

ALD10: no I like fresh fruit like um the little (break up) so I can have fresh fruit from Wegmans

Interviewer: and you said you're also drinking more milk now, is that because you're craving it or because you heard it was good to have during pregnancy

ALD10: because it was good to have I don't crave it

Interviewer: and do you remember who or where you read milk was an important nutrient

ALD10: the doctor um and the WIC lady told me

Interviewer: um and how much milk do you think that you're drinking a day now?

ALD10: hmm it's really just in the morning time maybe just a cup or a cup and a half

Interviewer: do you have it with cereal or do you just drink it

ALD10: with cereal

Interviewer: um so it sounds like you made some changes to you r diet so I'm just curious in how you prepare food so if you used to bake something do you fry it now or broil it did you change how you prepare it

ALD10: um more baked food than fried food but sometimes I still have fried food (breaks up)

Interviewer: do you crave fried foods?

ALD10: sometimes

Interviewer: um and what kinds of things are you baking now than you did before

ALD10: chicken, fish, like pork, um that's the majority of stuff I like and like shrimp and just put in on what is it called, not when you um I boil it or what is it? When you put in on a pan (break up) satay

Interviewer: oh satay gottcha that s good I like that. So we talked about dairy are there any other foods that you've changed or thought more about now that you're pregnant

ALD10: um no, did you say what I think about when I'm pregnant?

Interviewer: mhm

ALD10: um I don't really have a lot of cravings and stuff (breaks up)

Interviewer: are there any smells that you like more or dislike more now that you're pregnant

ALD10: no my mom says it smells really high but (mumbles I cant understand!)

Interviewer: okay, um and do you ever like look at the labels on food items where it says what the nutrition content is

ALD10: not really I do now kind of more than I used to and I look at the sugar and stuff in there I don't like to eat a bunch of stuff with sugar in it and I buy Splenda now

Interviewer: is there a particular reason that you've switched to Splenda and changed your sugar intake?

ALD10: um I guess I heard about the glucose and diabetes stuff when being pregnant (breaks up) you can get the diabetes more when you're pregnant which is why I don't eat so much sugar

Interviewer: do you have an example of a food that you looked at and you weren't happy with the sugar content so you put that food back?

ALD10: umm ice cream would be one of them, um the candy that I eat like the fruit snacks and stuff (breaks up) um and like cupcakes I eat I try to eat more granola bars and stuff the ice cream I try to do the light frozen yogurt and stuff like that

Interviewer: are you taking any vitamin supplements at all?

ALD10: yeah prenatal

Interviewer: any other additional supplements like iron and calcium and stuff?

ALD10: no cause the fiber in there it has all I need, I have the amount that I need

Interviewer: perfect so you mentioned that you look at the labels on the food sometimes do you ever try to choose foods that have certain things in the besides sugar maybe from the vitamin mineral section

ALD10: um no

Interviewer: and you said that you eat cereal is there a kind that you eat more often?

ALD10: um no like I eat like cheerios or um they give you certain kind of cereal now on the WIC thing for the babies like Chex and cheerios is the other one they give you like Corn flakes stuff like that

Interviewer: okay is sounds like WIC has been a good resource for information and food choices

ALD10: yeah like the beans and stuff

Interviewer: oh yeah so what other advice have you received about pregnancy

ALD10: um the drinking stuff that too with the um like I used to drink pop I used to drink a lot of soda and now I drink 100% what WIC gives to you and water and with the food yeah they tell me to eat a couple of smart meals a day instead of bigger meals you'll be hungry more

Interviewer: and that all came from the WIC program?

ALD10: and the doctor

Interviewer: okay. If you had to pick one person or even if it was an Internet site who or where has given you the best information about prenatal health

ALD10: um maybe I mean all the information I got has been the same with the doctor and I use Google a lot if I have questions and see what other people got to say if I have food questions

Interviewer: could you talk a little bit more about things you have Google searched?

ALD10: hmm yeah I Google weird things I don't even know I Google so much things I Google stuff about food before and baby movements and how far a long I mean I Google everything ha-ha anything that come to my mind I Google I don't even remember half the things I Google

Interviewer: do you usually Google right away when the question pops in your head

ALD10: yeah

Interviewer: what do you use to access the Internet?

ALD10: my phone

Interviewer: okay, and what kind of phone do you have

ALD10: Samsung galaxy S4

Interviewer: and on that you have texting, Internet and the ability to download apps?

ALD10: yeah

Interviewer: okay, so when you do Google something and you get that list of choices that you kind of pick which website you go to, how do you decide what links to click on?

ALD10: I just click all of them I just click them and see what different stuff they have on there. Not every page if it’s a page on info I click on the first couple of links I wont go through all the pages

Interviewer: are there any sites that have come up a lot when you search for a question that you find are really useful?

ALD10: um I don’t even pay attention to the site names I just read the stuff I don’t ever go back to the sites I just read the stuff and click off of it or with the apps that I have I just baby app and other apps they have forums where people talk about things and I go there daily

Interviewer: okay, um so just one more question, lets say that you Googled a question and you went to the first few links how do you decide if its good information or not

ALD10: majority of time the stuff be the same like the little doctor stuff I remember this one it be like the WebMD one and its like a couple of other ones and if I see em Parents I think I would know if I see the website the majority of them have the same info so I just take what I see and I assume that its right cause it comes from doctors and stuff.

Interviewer: okay

ALD10: and I look at comments too the main page thing I go down and scroll and see what other people think about it

Interviewer: do you have any friends who have been pregnant in the past or who are currently pregnant that you swap stories with?

ALD10: um I have one friend who is pregnant now and I talk to her daily but we really do talk as much about it

Interviewer: okay, um and you mentioned that you have the Baby bump app and you access that daily, are there any other apps that you downloaded?

ALD10: yeah I have the um let me see I have Baby Bump I’m Expecting and a pregnancy one

Interviewer: and do you use those as regularly as the baby bump one?

ALD10: just the baby bump and I’m expecting because they have the same info so I don’t click on them that much baby bump is the one I use a lot way more than the other two

Interviewer: and I heard that there are cell phone texting programs have you joined those things

ALD10: no

Interviewer: okay and what about twitter?

ALD10: I have twitter but I don’t use it

Interviewer: okay and just a couple more questions about food. Who usually does the shopping?

ALD10: me I live by myself so me

Interviewer: on average how much do you spend a week on groceries?

ALD10: I don’t go weekly I go monthly and its almost 200, its only me

Interviewer: okay, so you live alone?

ALD10: yes

Interviewer: okay, and do you feel like you’re getting good support from your baby’s dad

ALD10: mhm he comes over he just doesn’t live with me

Interviewer: is he excited?

ALD10: yeah

Interviewer: have you guys talked about names at all?

ALD10: oh yeah he think the baby name is going to be after him but I’m not naming the baby after him he already a junior and I don’t want a third

Interviewer: still up in the air about the name to pick

ALD10: yeah people give me names its just hard to choose

Interviewer: yeah, sometimes when you see their little faces all the sudden you know

ALD10: yeah

Interviewer: um so lets switch gears and talk a little about exercise and physical fitness, what have you heard about the importance of exercise during pregnancy?

ALD10: well the doctor tell me all the time that its good to exercise because it make the labor easier and the whole pregnancy easier and that’s really all the doctor tell me about it. Actually she gave me a paper today about the second half of pregnancy and it’s about exercising and I just got it today

Interviewer: and that was from your midwife

ALD10: yes

Interviewer: have you ever read about that in any of your Google searches or read about it in baby bump?

ALD10: oh yeah baby bump but that’s about it

Interviewer: have any messages really stuck with you and had you change anything you were doing

ALD10: no they just really say the same thing like just walk and make sure you active they don’t say too much just make sure you walking and you active and just not being lazy

Interviewer: have you made a change since before you were pregnant?

ALD10: no really but somewhat not as much as I wanted too. At first I was trying to walk I have a gym at my complex so I was trying to do treadmills and I stopped doing it and I know I need to start now

Interviewer: I know sometimes women say when they’re pregnant they have less energy and its hard to get out and do things, would you say that’s been happening for you as well?

ALD10: yeah, I’m tired a lot

Interviewer: um so how much would you say that you walk on average per day?

ALD10: um, I don’t know, you mean like how much time or what do you mean?

Interviewer: time is probably easier to add up then distance so do you think you could time out like a day

ALD10: umm not really cause I don’t really do the treadmill and stuff no more so I cant really say that cause I don’t do it no more like when I’m out and about of course I walk so I don’t really know its different

Interviewer: and do you walk to work or drive

ALD10: drive and I’m active a lot more when I’m working

Interviewer: does your job require you to be standing more or sitting?

ALD10: it’s a standing job I work with people with mental disabilities.

Interviewer: have you heard anything about a certain number of minutes that a woman who is pregnant should exercise?

ALD10: no the doctor just told me that I should do what I feel comfortable with or don't do it too much that I cant take (breaking up) not telling me to do a certain time or anything like that

Interviewer: have you um heard about anything that exercise can do for the baby?

ALD10: hmm no just about me and staying healthy I haven't heard too much about what it can do for the baby

Interviewer: okay and in addition to walking have you heard of any other exercises that are good to do during pregnancy?

ALD10: umm no

Interviewer: and on the opposite end of the spectrum what activities are probably not good for a pregnant woman to do?

ALD10: as in exercise, hm the main one I've seen in sit up and pushups you know stuff that would strain you

Interviewer: okay. I did forget to ask a question earlier about um do you think that you're eating more now than you did before you were pregnant?

ALD10: no it's the same

Interviewer: what about the number of times you eat throughout the day has that changed?

ALD10: um no but I though it was going to change a lot maybe just not yet maybe towards the end I might have cravings towards tings I don't have cravings and I'm not that hungry but I have to eat a lot

Interviewer: did you struggle with nausea or something at the beginning of your pregnancy?

ALD10: no, not at all.

Interviewer: one last question about diet and eating. Are there any things that you've heard a pregnant woman should be careful about eating?

ALD10: yes, um lunch meat because the listeriosis in it, the blue cheese, tuna fish, like sea food basically (breaks up) dishes of stuff beef tilapia stuff like that I got a little list at home but I cant really remember from the top of my head

Interviewer: any food that are really really good for the baby's growth and development

ALD10: um not really just like vegetables and stuff like that the doctor is telling me a lot of fiber and calcium and stuff like that I don't hear so much about meats or anything like that

Interviewer: okay so and then the third major thing I wanted to talk about was weight gain. What sort of tings have you heard about how much a woman should gain during her pregnancy?

ALD10: um stuff that I read in Google and like the um little apps and doctor said the same amount it said different for different people there is a certain amount you should gain if you're already big it's a different amount. The doctor told me that I should be any more than like 20-25 and some people gain way more than that so I be careful about the stuff that I eat. So far I'm 6 months she told me that I gained 13 and that's not bad so I'm past the first half, usually it'd be 15 in the beginning and 15 like normal around 30.

Interviewer: um and is that something that you and your midwife talk about each checkup to make sure everything's going well?

ALD10: they check my weight every check up so yes they do tell me how much I gain or what I didn't gain stuff like that. Last time I went it was the most that I gained I gained like 8 lbs. that month, and last time the 8 snuck up on me from the time before but this time I went I lost weight instead on gaining it.

Interviewer: um and have you heard like what not gaining enough can do to you or the baby or what gaining too much can do to you or the baby

ALD10: no

Interviewer: has your midwife talked about why they have those recommendations?

ALD10: so they just tell me to stay healthy they never get into what it does

Interviewer: and do you agree with the range of weight gain they paired with you or have you heard things from other places that give a different amount of weight

ALD10: (breaking up) I agree with it but she said its going to be very hard to do because anyone that I know that has kids gain a lot but they loose it right after so

Interviewer: mhm and as far as loosing it after, is that something that you think a lot about or have you had to think about

ALD10: mhm I think about that often

Interviewer: what kinds of things do you think about that?

ALD10: I think about how fast it's going to go away haha so I wouldn't have to do too much

Interviewer: do you have a plan in mind?

ALD10: basically a crash diet

Interviewer: okay and have you changed any of your eating/exercise patterns to stay within the range your midwife gave you?

ALD10: not really the exercise the eating I've been trying to do that trying

Interviewer: and what are the major decision that you've made about eating that have helped you stay within your range?

ALD10: the fried food and soda a lot of the stuff I already said

Interviewer: okay, and the vegies?

ALD10: yeah

Interviewer: okay. Is weight gain something that you've Googled at all?

ALD10: yeah before but not really like before at the beginning of the pregnancy like how much should you gain but the doctor basically said the information

Interviewer: did you ever see any of the weight gain trackers online where you type in your height and weight and it can track your weight during pregnancy?

ALD10: no but the doctor showed me something like that

Interviewer: okay. Um lets see. Um and then the last thing I want to talk about is to go back to talking about the Internet a little more. Do you have a computer that you go to for the Internet?

ALD10: um I have a laptop but the screen cracked an I never get it fixed

Interviewer: okay. So you mostly use your cell phone for the Internet?

ALD10: my cell phone or if I'm at work I use my work computer

Interviewer: and have you signed up for any email programs where a site will email you information

ALD10: yeah so same stuff that I basically got out on the apps they email me too or if I'm on Google and I go onto a website some of the sites will email me too

Interviewer: okay, and so far you have mentioned baby bump

ALD10: Parents, there's a couple that email me I cant remember all the names they email me I cant remember their names

Interviewer: okay is text messaging free on your phone or included in your plan?

ALD10: yeah is all in that

Interviewer: okay and what about Facebook groups, have you ever joined any pregnancy groups on Facebook?

ALD10: no

Interviewer: okay, um well I think those are all of my questions for you is there anything that we talked about that you want to go back to?

ALD10: um no

Interviewer: okay well let me stop this and the only other thing is the form that Lauren was going to have you sign, talk about what's on it. The texting Facebook agreement, the dos and don'ts that we hope people do on site and text messages. Facebook, no personal information common sense, were required to hand out the sheet by IRB.
If you do post something it has to do with the topics not random updates that would be better on your own page

The group is private only other women in the clinic, supposed to feel comfortable commenting and commenting back, ask people to be cognitive don't be mean

Text messaging we ask that you try to answer. We understand that people are busy we like to emphasize that you try.

Right cell phone number and right Facebook name.

ADL11

Interviewer: Okay so uh I generally start out with just some really basic questions to sort of describe your pregnancy and uhm where you are with like uhm some I guess emotional planning sorts of things.

ADL11: Right.

Interviewer: So the first question is how old are you?

ADL11: 34.

Interviewer: Okay, and how tall are you?

ADL11: 5’4”

Interviewer: And how much do you weight?

ADL11: Currently, or pre?

Interviewer: Uh currently. It can be an estimate.

ADL11: I think about 136.

Interviewer: Okay. And is this your first pregnancy?

ADL11: Yes

Interviewer: And was your pregnancy planned?

ADL11: Yes

Interviewer: And what hopes and goals do you have for your unborn child?

ADL11: Oh my god (laughs) Uhm… uh that they will be born healthy. Uhm and that my husband and I will provide them a loving upbringing?

Interviewer: That’s a pretty bold question (laughs) it can encompass many things. Uhm… What challenges have you faced so far as you’ve planned to become a mother?

ADL11: With the pregnancy?

Interviewer: Mhm

ADL11: Uhm… I’ve been fatigued. I’ve had a loss of appetite. Uhm also, just finicky appetite. Uh…just… heart burn. That’s kind of it.

Interviewer: Uhm and then what do you think are important things that a pregnant woman should be doing to make sure that she has a healthy baby?

ADL11: Uhm take your pre-natal vitamins. Don't drink or smoke. Exercise regularly. Uhm… eat a wide range of food, especially protein.

Interviewer: Okay, awesome. Thank you. Okay so… let's… uhm so the first thing I wanted to talk about was uhm… weight gain. And I always ask if you've been given advice about weight gain during pregnancy.

ADL11: By anyone?

Interviewer: By anyone.

ADL11: Uhm… yeah. So… and you want to know what that advice is?

Interviewer: Yeah.

ADL11: Sure. Uhm that you're supposed to gain between like 25 and 35 pounds for a single gestation. Not twins. Uhm… but you don't really gain that much weight in your first trimester, but then it sort of picks up over time and… you still gain a lot in your third. Uhm… what else? To be patient in post-partum period with your weight coming off.

Interviewer: And is there a specific source that you received that advice from?

ADL11: The 25 to 35…or any of it?

Interviewer: Any of it.

ADL11: The be patient was more of my mom. And then I think the specific 25 to 35 I looked up on like… ohhh what's the federal baby site? It's not WebMD… it's uh… Healthy Baby? Is that it?

Interviewer: I think that it might…

ADL11: And it's from the CDC or something. Yeah.

Interviewer: Okay.

ADL11: And I think What to Expect When You're Expecting. That as well.

Interviewer: And is that range something that you've also heard from other people? Or your midwife? Or… health care provider?

ADL11: Uh yeah. My midwife said that.

Interviewer: Okay. And what are your thoughts on that range? Do you feel like that fits you?

ADL11: Yeah I mean I'm going to try to stick to it. If I'm hungry I might eat but um… so far I haven't gained a lot or lost a lot or… I think I can do it. We'll see (laughs).

Interviewer: And have you heard about anything that can happen to mom or baby if she gains outside of sort of a healthy weight gain range?

5:17
ADL11: Mhm. So if you've gained too much, it ups your risk for gestational diabetes and preeclampsia. Uhm which I think then ups your risk of preterm birth. Uhm underweight I haven't read as much on, cause I don't think I'll be underweight. Uhm but that probably I mean that probably just means your fetus isn't developing. Well, and that you're not getting all the nutrients that you need.

Interviewer: So it sounds like you've used the Internet as a resource for weight gain recommendations. Have you ever run across anything that you thought seemed that it wasn't a great source of information on weight gain?

ADL11: Uhm I don't really see anything specifically on weight gain, but there is times that I'll like do Google search and like chat rooms come up, and it's always like the most extreme situations come up on those sites. And you read them and you're like oh my god, stop, stop reading this!

Interviewer: Uhm so when you Google a question, how do you go about picking what sources that you're going to click on for information?

ADL11: Uhm usually if it's a government website, like CDC uhm… if it's an American college, it would be (indistinguishable) Uhm WebMD, What to Expect When You're Expecting, sort of try to pick more reputable sources. I don't know why I consider them reputable though.

Interviewer: Um and has anything you found on weight gain changed your behaviors at all, in terms of how you eat or how often you exercise?

ADL11: Uhm I mean I try to exercise frequently and eat well. I feel like I know what I should be eating, but that's just been harder to like actually eat. Like I don't think I've eaten vegetables in awhile (laughs) but I know I should eat them.

Interviewer: Is that just because of…

ADL11: They just seem gross.

Interviewer: The smell and the taste? Or--

ADL11: Yeah. I just… I'd rather have bread. (laughs)

Interviewer: Uhm so let's talk more about diet. Uhm so just as a quick introduction, who generally prepares the meals that you eat?

ADL11: Uhm I used to. Since I became pregnant, my husband's doing it more.

Interviewer: And is that um because of issues that you've had with the pregnancy?

8:40
ADL11: Yeah, yeah. I mean I make my own breakfast and lunch, but by the time I get home for dinner, if I'm just left to do dinner, I just like have a bowl of yogurt and crackers.

Interviewer: that sounds (overlap)

ADL11: My dinners have gotten less complicated, let's say that (laughs).

Interviewer: And who generally does the shopping for the food that makes those meals?

ADL11: Uhm it's really 50/50

Interviewer: Okay. And given that it's a 50/50 split, would you say that both are involved in the decision making, or do you, does one person always kind of make a list and then you just stick to the list?

ADL11: I mean we kind of do it together. Like there's certain like standard stuff, like we always have cereal for breakfast. And I know he also likes fruit for his lunch. So, I don't think every time we need to sit down and like go through the list together, but.

Interviewer: And how much money do you think that you spend per week on food?

ADL11: Food shopping, or like take out food? Like restaurant.

Interviewer: Um I guess… let's just say groceries.

ADL11: Groceries? Uhm… 90 bucks?

Interviewer: And so you suggested that things have changed a lot with how you've been eating, so can you just tell me a little bit more about how you've been eating during pregnancy and how that's changed?

ADL11: Yep. Uhm so before I was pregnant, I was a vegetarian who ate fish and seafood. Uhm and so while that hasn't… like I would still eat vegetables, you know still eat that stuff. I tend to eat vegetables a lot less. I love bread, I love fruit. Uhm I've started eating meat just because with the amount of protein that you need during pregnancy doubles. And uhm because I'm already finicky about what I eat, if I was going to remain vegetarian and try to get like all the protein like

every (kind?) would have to have protein in it. Which is just a pain. I had steak this weekend for the first time in like 20 years. It tasted really good. (laughs) I was like this is amazing!

Interviewer: Uhm, and you said bread. And so is that the changes—I mean it sounds like you're really conscious of protein and uhm. But is there, was there any influence on eating meat because that's something that you've been craving?

ADL11: No, not at all.

Interviewer: Okay. And… what about snack type foods? Like granola bars, chips, candy.

ADL11: Yep! (laughs) Uh so yeah, I usually have a couple granola bars, cause they have some protein in them. Uhm I try to have fruit as snacks, but if like I'm around… like my husband brought home cookies the other day, and I was like, "I hate you!" And then I inhaled them. So we just try to keep junk food away from me cause I will immediately eat it. Uhm so yeah, I'm trying to do fruit. I'm trying to do like granola bars. Healthier stuff. But yeah. If I'm around like chips or junk food, I'll absolutely eat it.

Interviewer: Me too. (laughs) I was going to say, I'm the same, but I'm not pregnant. (laughs) Uhm are there any other modifications that you can think of that you've made to how you prepare food or things that you've added or removed from your diet from before?

12:43
ADL11: Oh so yeah. I won't eat blue cheese, I won't eat runny eggs, uhm… if I'm having fish that has high mercury, like I won't have it more than twice a week. I'm not drinking. What else could I not eat? Oh anything unpasteurized.

Interviewer: Were there things that you ate before that weren't pasteurized?

ADL11: Not that I know of. Yeah I know it was funny. When I read that, I was like okay. And then everything I look at, I'm like, oh, it's pasteurized. Ok (laughs).

Interviewer: (laughs) Um and then in terms of the quantity of food that you've been eating, how does that compare to pre-pregnancy?

ADL11: Uhm I would say initially, initially I actually lost weight cause I had no appetite. And so I would say in the past couple of weeks, that's come back. And I find that I can't—like before, before I was pregnant I would have meals and you know like eat a full plate of food whereas now, my appetite is back but it's more of like… eating constantly throughout the day. Like I find I can't eat… like I can't eat a full plate of food quickly or even like at a normal pace. I'm a very slow eater now.

Interviewer: And so since you're not eating like actual meals per say, you're getting full faster?

ADL11: I don't know if I'm getting full. It's that I don't feel super hungry.

INTERVIEWER: Okay.
ADL11: Like I'll know I should eat, and I'm like okay. And I just have to eat really slow. Cause if I get full, I'll feel gross.

Interviewer: Mhm. That makes sense.

ADL11: Yeah (laughs)

Interviewer: Uhm and so it sounds like you've changed a lot of things and I know—I know we talked about protein because you know there's an increased need for protein um for the baby, but what are some of the other reasons that you've uhm changed foods and reduced fish consumption and blue cheese, those sorts of things?

ADL11: Cause you're not supposed to eat them while you're pregnant.

Interviewer: Ok.

ADL11: I would eat runny eggs if I could. (both laugh)
15:12

Interviewer: Uhm, and would you describe yourself as being hungrier now even though, I know that you said that you're eating—

ADL11: It's weird. I get hungry at times when I never used to be hungry. Like I'll wake up in the middle of the night hungry, which like never used to happen. Like I have a bag of pretzels by my bed. Like my husband will wake up at 2am and be like (mumbles) Uhm so yeah, so maybe it's like I'm constantly a little bit hungry. Or like hungry at times I wasn't before.

Interviewer: And through the days, since it sounds like you're snacking more regularly, do you notice that you're hungry? Or are you sort of just always eating a little bit here and there?

16:00

ADL11: It's weird. It's not like if I don't eat, I feel hungry. It's like if I don't eat, I feel gross.

Interviewer: Like nauseous, sort of?

ADL11: Yeah like heartburn, nauseous… so I don't know if that's nat— like I assume it's because I'm hungry, but I just feel like blugh. But I don't feel that feeling of oh, my stomach's empty and I need to eat cause I'm hungry.

Interviewer: And have you heard of any foods that are extremely good or extremely bad for a growing fetus?

ADL11: Uhm… yes. Well there's all the foods you're not supposed to eat cause they have bacterias in them, like the blue cheese, the runny yolk, the smoked fish, the pate, blah blah blah.

Food that’s really good for the baby? Anything with folic acid. Some beans. Uhm sort of you know… wide varieties of foods so you’re getting a balanced diet. Uhm food with iron, calcium, protein. Uhm… omega fatty acids? Actually the one thing I can have that doesn’t gross me out, so my friend told me about cod liver oil that actually is lemon-flavored.

Interviewer: Does that work?

ADL11: Yes! And it’s just like lemon-tasting oil, and like… it’s so good for you, but like it’d be so disgusting if it tasted like cod liver! But yes, that’s the only thing I’ve been able to take like every day. Like sometimes I can’t take my prenatal, but I can always do that.

Interviewer: Well that’s good.

ADL11: Yeah, it’s really weird.

Interviewer: And um what have you heard the benefits of the omega fatty acids are?

ADL11: That it makes babies smart. It helps their brain function, and yours as well. And it’s good for your hair and skin (laughs)

Interviewer: Just as a last ditch probe, are there any other things that you can think of that maybe you’ve removed from your diet or added to your diet?

ADL11: I’ve added meat, specifically steak, I love it. (mumbles). That I’ve removed? I haven’t like mindfully removed vegetables. I just feel gross about them. And then I’ve just removed all the stuff you’re not supposed to eat.

Interviewer: Are there any vegetables that you’ve been able to tolerate or found that maybe like…

ADL11: I had some salad the other day, and I think I like crunch or something that’s crispy. So the salad was good, and I was like, oh. Maybe I could start eating this. And I had some asparagus. And it was the same type of thing; it was crunchy.

Interviewer: Was it raw asparagus or cooked?

ADL11: It was cooked but it wasn’t like super cooked.

19:15
Interviewer: Like lightly steamed?

ADL11: Yeah, yeah. It still had a crunch to it.

Interviewer: Uhm and have you ever heard that a pregnant woman’s diet should change throughout pregnancy so that you’re providing different sorts of nutrients to the baby?

ADL11: No.

Interviewer: Okay.

ADL11: Are you supposed to?

Interviewer: I don’t know. (chuckles) Um, let’s see. And have you changed how you’ve been preparing foods at all? So like frying versus baking, um adding salt or not adding salt?

ADL11: Oh. Yeah. I mean I’ll try to always make it healthier, you know like not fried. Bake it. You know… not put a ton of butter on it. But you know, I mean I’ve always tried to do that. Maybe I’m a little more conscious of it, especially as I start to gain weight.

Interviewer: Um so these are things you’ve tried even before pregnancy, just as more of a healthy lifestyle?

ADL11: Yeah.

Interviewer: Okay. Um. And if I say a nutrient, I want you to say the first thing that pops into your head in relation to pregnancy.

ADL11: Okay.

Interviewer: Um so the first word is salt.

ADL11: Thirsty?

Interviewer: Okay. And sugar?

ADL11: Uhm… tea.

Interviewer: And are those uh nutrients you think a lot about being important

ADL11: NO

Interviewer: …to monitor during pregnancy?

ADL11: Oh. Yeah. To not have too much of.

Interviewer: And what about dairy food? I know sometimes that can be hard to stomach while pregnant.

ADL11: Dairy’s been fine.

Interviewer: Okay.

ADL11: Yeah, no. Dairy's great.

Interviewer: And do—it sounds like you eat cheese. You drink milk and have yogurt?

ADL11: Yeah.

Interviewer: Okay. Um and have you been craving any nonfood items like chewing on ice, um interest in eating baby powder or cornstarch?

ADL11: Pica? No. (laughs) I like chewing gum, but that's—

Interviewer: And that's just—after you became pregnant (overlap)

ADL11: That's what you would put in your mouth. And that's because I have heartburn.

Interviewer: So it helps with that?

ADL11: So a minty gum helps me feel cooler…

Interviewer: That makes sense. And are there any smells that you like more or less now that you're pregnant?

21:52

ADL11: Smells just seem stronger to me.

Interviewer: Mhm.

ADL11: Uhm… so it's not like I smell coffee and can't stand it. It's just that I smell, it seems so much more overwhelming. But there isn't a specific smell that I just love or hate.

Interviewer: Okay.

ADL11: Like I walked into the cafeteria the other day and was like oh my god… I feel like it's all pizza in this room. Like there's just this really strong smell.

Interviewer: Mhm. And nothing you like the smell enough of to get it out and smell it? Um so… an example, some people that I've spoken to will actually pull out the bottle of Pinesol and just smell Pinesol.

ADL11: Oh really?

Interviewer: Because they really like the smell of—

ADL11: Lemon or whatever—

Interviewer: Pine or I'm not sure what it smells like.

ADL11: I don't know.

Interviewer: So nothing like that?

ADL11: No, I haven't done that.

Interviewer: That's good. All signs of pica, which doesn't sound like you have, so that's good. Uh that's what we hope for.

ADL11: That's what you get if you don't have enough nutrients, right?

Interviewer: Yeah, enough iron.

ADL11: Iron, yeah.

Interviewer: Specifically, usually. So yeah. It can be really common, so yep. So that's good.

ADL11: Good!

Interviewer: Cause people who don't have it are like, why are you asking this?

ADL11: Right, they're like that's so weird.

Interviewer: Uhm and then so it sounds like you're taking a prenatal unless your stomach's just not really in the mood for that. Uh and then you said also uhm the…

ADL11: The cod liver oil.

Interviewer: The cod liver oil.

ADL11: And then um. I've just been taking like calcium chews just cause my prenatals don't have that much calcium… you know they have like 40% of the calcium you need.

Interviewer: Mhm. And has anyone in your family or close friends who've had children offered any advice on things you should or shouldn't eat?

ADL11: Uhm, maybe not should or shouldn't eat. My mom was really surprised when I started eating beef in front of her the other weekend. And we had a conversation where she was like, why can't you just eat more yogurt? More nuts? And I was like, then I would constantly be eating everything protein in it…you know like.

Interviewer: Is your mother a vegetarian?

ADL11: No, she's not at all. She's just used to me being a vegetarian. And she always used to cook, like growing up, she made sure like there was complete protein and (mumbles)

Interviewer: Was it a hard decision to switch back? Cause um.

ADL11: mm no. I mean it's more convenient. Like if I want to eat an apple, I'm going to eat an apple. You know, I don't want every time I want to eat something, it has to be like nuts or like granola bar, yogurt. You know, like I'm already fickle about what I eat, so I'll just eat some meat.

Interviewer: Do you think that you'll go back to being a vegetarian after?

ADL11: I don't know. That steak tasted really good (laughs). So, we'll see.

Interviewer: Um, let's see. Um so earlier you mentioned you were using the CDC site for information about weight gain, um and sounds like you've Googled some things. Are there any other sites that you may have not have mentioned that you can think of in relation to food or weight gain that you've used as a resource?

ADL11: mm. Not that I could think of. I mean if there were, I probably looked at them quickly and dismissed it cause I don't remember what else.

Interviewer: Ok. And do you recall any of the food-related searches that you've done recently?

25:40

ADL11: Uhm… I don't know if I've really done any recently. Like initially when I became pregnant, and I like made sure I knew what I was supposed to do and not do. Uhm I do randomly will be like, huh I wonder how much protein an egg is in—how much protein is in an egg. So I Google that. Just like randomly when I think of a food and their protein content (laughs) I'll look that up, but… I mean my prenatal vitamins have a lot of iron… I kind of fixed the calcium thing with the chews. So like those are always the things I know I don't get enough of. Is usually protein, iron, calcium like even before I was pregnant. Uhm… so yeah, I'm just kind of fixated on protein.

Interviewer: (laughs) And is that because someone told you it was really important?

ADL11: Just cause I knew I wouldn't get enough just because I don't really eat meat. You know like before it's like 40 or 50 grams that you need when you're not pregnant, and it goes up to 75 when you're pregnant so… like after a couple weeks of like trying to figure out what I was eating. I was like yeah, I'm not anywhere close to 70, 75 so. And protein is not in my prenatal vitamin, so…
26:48

Interviewer: Right

ADL11: It’s not. Cause iron I’m not worried about cause my levels always come back fine. It’s in my vitamin.

Interviewer: Mhm

ADL11: But yeah. I need to get some protein.

Interviewer: Um so do you frequently look at labels when you’re choosing foods? What—what are the key things that you’re really looking for when you look at a label?

ADL11: Uhm… usually like how much sodium is in it. Um how caloric it is. Uh… maybe like how much calcium or iron or protein is in it.

Interviewer: Uhm, and you mentioned that you eat cereal. What kinds of cereals do you typically eat?

ADL11: Uhm like the Kashi, like granola-y… granola-based cereal pretty much.

Interviewer: And do you try to choose cereals that have a lot of um nutrients added?

ADL11: Uhm yeah, I mean I try to look for things that have a lot of nuts or dried fruit in it or uhm… make sure it’s not just like a… make sure it’s got a lot of stuff in it.

Interviewer: Uhm so let’s switch gears and talk a little bit about physical activity. Uhm have you—what have you heard about physical activity during pregnancy?

ADL11: Uhm that it’s good to be physically active. Uhm… that especially if you were active beforehand, you know like you don’t, not that you don’t need to worry, but basically… the main thing is that your pregnancy hormones are going to sort of mellow out your muscles so it’s uh… it’s easier for you to pull a muscle.

Interviewer: Mhm

ADL11: So if you’re someone who never exercised before and you start, you know you might be at an even more increased risk for pulling a muscle. But if you’re someone who exercised beforehand, then you’re just carrying that over then your muscles are going to be a little more relaxed, but you’re probably not going to have like… someone who is getting back an exercise injury that they would have like to that extent.

Interviewer: And have you heard of any recommendations for pregnant women?

ADL11: About how much to exercise?

Interviewer: Mhm

ADL11: Uhm, I think you’re supposed to do about half an hour every day, ideally. Uhm… if you are taking some sort of class or something like led by an instructor, it’s good to let them know that you’re pregnant, and they will guide you as needed if there’s something you should or should not do… uhm, you know you probably don’t want to do like extreme sports like… you know… scuba diving or parasailing. Uh you don’t really want to do contact sports, you know like you wouldn’t want to do rugby. But yeah, you can continue your routine for the most part.

Interviewer: Have you heard of any exercises that are better for pregnant women than others?

ADL11: Uhm, especially if you… like as you gain weight, I’ve heard more things like swimming is really helpful, you know like things that make you weightless. Just cause you’re more comfortable when you do it, um are good. Uh yoga, stretching, those are all good. Uhm… you know like if you start to feel out of breath or you don’t feel well, you know just stop. Take it easy, whatever you’re doing.

Interviewer: And have you received any advice personally about what you should be doing for your pregnancy?

ADL11: Uhm, just that I should exercise. Not something specifically.

Interviewer: Okay. Is that something that your health care provider talks about at health care visits?

ADL11: Uh, yeah. I think we talked a little bit about exercise and how I did it before, and it was important to continue what I was doing.

Interviewer: Uhm if you wanted to learn more about exercise during pregnancy, where would you go to look up information?

ADL11: Mmm… probably the same sites as before. CDC, ACA?, What to Expect…

Interviewer: And what’s your current exercise regimen like?

ADL11: Um I try to exercise like 4 to 5 times a week. It’s usually a combination of running, yoga, jazzercise I’ve gotten really into (laughs). Uhm… gardening now that the weather’s better.

Interviewer: Finally!

ADL11: I know, seriously. (laughs)

Interviewer: And do you have a favorite activity that makes you—

ADL11: I really like Jazzercise! (laughs) it’s really fun.

Interviewer: I’m not coordinated. I could imagine if I did that, it would result in me like falling on the coffee table or something.

ADL11: Luckily there's no—so I take a class here and there's no mirrors in the room, so you're like yeah. I look like the instructor. I'm not trying to look crazy and like—it's perfect.

Interviewer: Uhm has the total amount of exercise that you're doing now changed from before pregnancy?

ADL11: Uhm initially it went down because I was really tired, um but my energy's come back, so… it's probably back to normal. Maybe I'm a little bit more tentative in what I do now… like I was in yoga the other day… and like something that I would previously try to stretch or like twist, I was like no, I'm good here. Like I'm not as…

Interviewer: that makes sense.

ADL11: I'm more moderate I guess.

33:27
Interviewer: And are you happy with the amount that you're able to exercise right now, or do you wish that it was more or less?

ADL11: Uhm… maybe good to always do more, but I'm not super worried about it.

Interviewer: Do you have um friends that you typically go to classes with?

ADL11: Mhm

Interviewer: Okay. Do you think that's an important aspect of like motivation?

ADL11: Yeah, it really is. One of my friend—like all my friends are pregnant right now, actually, so it's nice. Like after this I'm going go meet her and she's pregnant and we're going to go for a walk.

Interviewer: Oh, nice.

ADL11: Yeah. She's making me do 50 squats whenever I see her. It's terrible.

Interviewer: (laughs) Any particular reason why?

ADL11: She's like oh it's good to have strong legs and strong thighs like when you give birth. And I was like sure. And if it does, if it doesn't impact it at all, then at least I have strong legs so that's fine. But she does like 50 squats every day when she's pregnant.

Interviewer: that's a lot of squats

ADL11: Yeah, she's crazy. I can only do it like with her. It's nuts, I don't know why.

Interviewer: (laughs) I have to spread them out like 5 at a time. Like every hour

ADL11: And they're so boring, too. You're just like…

Interviewer: Yeah, I agree.

ADL11: Yeah, she's crazy.

Interviewer: Hum and have you heard of any positive effects that physical activity can have on a developing baby?

ADL11: Uhm, on a developing baby… I mean heard it's good for the mom and it's stress relieving. And it makes me feel better. Uhm, I'm hoping it will make my labor a little better, and that I… shed my pregnancy weight a little easier. I can't imagine that it's bad for the baby. I don't know specifically what's good for it, though. Maybe increased blood flow?

35:45

Interviewer: Uhm, and are there any specific exercises that you've heard that are really good for preparing for labor or also for the baby?

ADL11: Apparently 50 squats a day (laughs). I've heard Kegels are good. Any sort of uhm core exercise is good for… labor.

Interviewer: And when you say core exercise, can you tell me what that means?

ADL11: Um so anything that exercises from here to here, basically. So maybe like sit ups or plank… uh any sort of thigh workout. Back workout.

Interviewer: And do you lift weights as well?

ADL11: Uhm… I mean I don't like bench press, but yeah I'll lift like hand weights.

Interviewer: Okay. Uhm so we talked a lot about the Internet and some sources that you found beneficial. But what about other um social media? So uhm have you joined any Twitter accounts, Facebook groups, text messaging applications?

ADL11: Uhm I haven't yet. I'm kind of… I probably will within the next couple weeks become a little bit more active with that. Like we were kind of low-key in our first trimester… you know just try to get through the first trimester. Uhm… we just started telling a lot of people in the past week, so I'll probably kick that up more.

Interviewer: And so, I'm just curious, uh as you plan to do that, what things have you sort of thought about joining? Are there anything specific that you're looking for in a program?

ADL11: Uhm, so my friends have said that parent groups, like Facebook groups have been really helpful for them. My friend keeps telling me about this Due Date club that she's a part of, so like basically you're grouped with all the women who are due around the same time. And it's nice you kind of chat with them and talk. Uhm a lot of my friends have said that it's a waste of time to spend a lot of money on buying stuff because a lot of times you know, you can just join these groups, and people have used… you know second-hand stuff that you can just trade.

Interviewer: Mhm

ADL11: Do that.

Interviewer: And would that be like books, what to expect…

ADL11: Like baby clothes.

Interviewer: Oh ok.

ADL11: Yeah. Like baby stuff. My mom is so old school. She's like you don't need anything! Okay. Strollers are a waste of time! (laughs) I think I need a stroller.

Interviewer: Could be helpful.

ADL11: Yeah, might be helpful (laughs). I was her last kid, so she had another kid for 35 years. Her memory's like a little fragmented from raising children.

Interviewer: Uhm, and are you considering joining any phone apps like uhm…

ADL11: Oh, yeah. Um I did that right when we got pregnant. I forgot about that. And I—my husband put it on his phone too because I'm trying to include him as much as possible.

Interviewer: (laughs)

ADL11: Why is it not coming up… but yeah. It's a free app, Pregnancy… Baby Center.

Interviewer: Okay. And is that the only one you're currently a part of?

ADL11: Yep

Interviewer: Okay. And how often do you get updates from that app?

ADL11: Mm I mean it kind of updates itself. Like I don't get notifications saying like, yeah there's a new message. So I probably check it like once a week. I mean, it's nice. It kinda gives you like a week-by-week of where your baby is or where your body is. And it has some other stuff. So it's kind of helpful like just to see uhm… I get the What to Expect When You're Expecting e-mail newsletter. Uhm… I think that's kind of it. Like I think like initially I was

excited to look around. And I was like wow, you can get overwhelmed really quickly. So I’m just going to pick like two or three things.

Interviewer: There’s definitely a lot of stuff out there.

ADL11: Yeah, I was like…

Interviewer: And uhm, have you joined any text messaging groups?

ADL11: Nope.

Interviewer: Okay. And it looks like you own a cellphone. And you have a data plan?

ADL11: Mhm

Interviewer: And um does it cost you money to receive text messages?

ADL11: uh well no. We just pay for the plan each month.

Interviewer: Okay. So it’s unlimited.

ADL11: It’s not a la carte.

Interviewer: And can you receive photo or image text messages at all?

ADL11: Mhm.

Interviewer: And do you own a home computer that you—

ADL11: Mhm

Interviewer: Is that where you do a lot of your Google searching, or would you say that you use your phone more often?

ADL11: Um probably yeah my laptop more.

41:44
Interviewer: And if you did use your cellphone to search the Internet for something pregnancy-related, how do you think that search would differ from a search that you would do at home?

ADL11: Uhm almost because it’s a smaller screen, so I kind of lose patience more.

Interviewer: Mhm

ADL11: Like I probably wouldn’t do an in-depth search on my phone. I’d save that for home. Like if I just need to look up one quick thing on my phone, I would do that.

Interviewer: Okay.

ADL11: But… I would do searches where I could find the information quickly and easily. It wouldn't be like PDFs or things too big to do deal with on the phone.
Interviewer: And how often is the What to Expect newsletter?

ADL11: I think the news, like the actual newsletter is like weekly. But then I feel like there's always these offshoot things that I'm sent. Like I realize I suddenly have like, baby clothes sale, like those types of emails in my inbox.

Interviewer: uh huh

ADL11: I'm assuming that that's related to signing up for that newsletter.

Interviewer: Okay. Uhm so is there anything that you can think of that maybe you thought of another answer to an earlier question that you didn't get to say that we could go back to?

ADL11: Mm… Nope. I don't think so.

Interviewer: Okay. And in signing up for this study, you know that you're gonna receive text messages uhm on a semi regular basis. We kind of send about six a week, actually. It varies. Up to six. So it's like 3 to 6 a week. Because we also have teens in the study, so we're trying to find some sort of middle ground that's good for all age groups. And that's one thing that we talk about in the post-interview as well. Like was that too much? What's good about it? That kind of stuff.

ADL11: What is the age range in the study?

Interviewer: Uhm so the minimum age is 13, and no age cap.

ADL11: That's interesting.

Interviewer: So I guess one last question that I have is, I mean in signing up for the study was there anything that you were hoping to learn or receive through messages or something that maybe you haven't found online that you thought maybe you would get messages that we sent, having to do with mental health…

ADL11: Hm!

Interviewer: uhm general prenatal care, diet, and exercise are kind of the overarching themes.

ADL11: Right. I don't think there's something specific. I just I never qualify for research studies, and so now I'm just scientifically interesting. So I'm like whoa, I can contribute to science. That's great. Uhm… I can't think of anything specific. Like I just thought it was kind of interesting to look at, like how people receive information.

Interviewer: Yeah. That makes sense.

ADL11: Yeah!
Interviewer: Well we appreciate you joining. Um so I think that’s it. Any other questions or thoughts before we close out?

ADL11: Not that I could think of.

Interviewer: Okay!

ADL11: Thank you!

ADL12

Interviewer: So I wanna start by just asking a couple basic questions to describe you and your pregnancy. So the first is, how old are you?
ADL12: 30.
INTERVIEWER: Okay! And how tall are you?
ADL12: 5’4.
INTERVIEWER: And how much do you weigh?
ADL12: 129.
INTERVIEWER: Okay. And this is your second pregnancy?
ADL12: Mhm.
INTERVIEWER: Okay…and…uhm I just always ask this question but, was this pregnancy planned?
ADL12: No.
INTERVIEWER: Okay. Uhm, and what hopes and dreams do you have for your unborn child?
ADL12: Uhm, I’m not quite sure. We just thought if it’s a boy—my—I have a daughter so, we’re just excited to explore the other end of parenting from a boy perspective!
[Laughter]
INTERVIEWER: It’s probably very different.
ADL12: Yeah!
[Laughter]
INTERVIEWER: Uhm, and then, what, what challenges do you feel that you face in planning to become a mother for the, the second time.
ADL12: Uhm well they’re going to be very close in age. They’re going to be 17 months apart. So just challenges of basically having two babies. Uhm, and I stopped working full time when I had my daughter (I worked pretty young) and I’m gonna stay home for the first six months and just work some weekends. So I’m a little worried about being home full time with two babies (laughs), since I do enjoy working. But uhm…So yeah, just the challenges of going to be having two—
INTERVIEWER: What, what types of challenges do you think about most in terms of being home and not at work?
ADL12: Uhm I feel I’m a physical therapist so I feel like when I’m home all the time I’m like (laughing) losing knowledge and like I just feel like I become dumb because I’m not using any of my skills or any of my, you know, years and years of education. Uhm, so sometimes it’s just hard for me in that aspect of not engaging in you know, and just doing what I like went to school all those years for. Uhm, that’s probably the biggest thing. [Laughter] Keeping that identity.
INTERVIEWER: I can understand that, yeah. Uhm, do you have the same sorts of feelings from like a social standpoint as well? Like for maybe not being able to interact with people your own age or—?
ADL12: Yeah, and I love—like I work in a hospital, so I work with lots of people and, you know, the staff that I work with are all great so it’s nice to have that interaction as well. So, yeah. That definitely plays a role.
[Laughter]
INTERVIEWER: Uhm, and last question for just the kind of intro things: what do you think are important things that moms should do to have a healthy baby?
ADL12: Uhm, in pregnancy or…?

INTERVIEWER: Mhm.
ADL12: In pregnancy? Uhm, I mean, just being conscious of, you know, like the dos and donts like alcohol, smoking, you know the basic things. But then also, you know, eating well, exercising, uhm you know, getting enough rest. Uhm I guess kind of just all those basic things.
INTERVIEWER: Yeah. Uhm, and are there any things that you think about that are important to do before you're, you're pregnant as well or is it—?
ADL12: Uhm, I mean I think for me just trying to be physically healthy before because I know like a lot of people feel like they're overweight or something before they get pregnant and then they don't want to gain too much weight during pregnancy, so it's like extra pressure. So I think if you're healthier before, just kind of in general, then you'll feel better about, you know gaining weight, your body changing, and everything like that.
INTERVIEWER: Makes sense.
ADL12: So yeah, nothing specific, but just kind of, you know, being in a healthy place before I think would make it easier (at least I see it that way for me).
[Laughter]
INTERVIEWER: Uhm, so where do you normally go to find advice or information about anything pregnancy related?
ADL12: Uhm, well I have the What to Expecting book which I read with my first daughter—I still have it but I haven't gotten it out this time [laughter] but it wasn't that long ago. Uhm and then I have the What to Expecting app as well, so that like has stuff every day. Uhm, and, you know, a lot of times if I'm like hmm I wonder if I can eat this, I'll just like google it. Like, is it okay to eat salmon during pregnancy, or something like that. (INTERVIEWER: Mhm) Uhm, but probably—I read the book with my first pregnancy so that was like the biggest thing.
INTERVIEWER: And are there any individuals that you kind of go to for support or based on, ask about their experiences in the past?
ADL12: Uhm, I have two older sisters who both have kids, so usually I'll ask them.
INTERVIEWER: And what about uhm your mother, or your mother-in-law?
ADL12: Uhm, probably not my mother-in-law.
[Laughter]
ADL12: Uhm, yeah, I ask my mom but usually things were so different back then and it was so long ago so usually she's like "I don't remember." [Laughter] Uhm, I mean I definitely talk to her about all the aspects of my pregnancy and things like that but usually for questions I'll go to my sisters.
INTERVIEWER: Uhm, so, for the, going back to the internet, you mentioned that you'll google something that you aren't sure about, or have uh, you know want to know more about. How do you, once you have like the list of options it gives you, how do you decide which links to go to?
ADL12: Uhm, I usually like check a few and… I kind of take everything like with a grain of salt 'cause, I mean, in PT school we took a whole class on Advanced Pace Practice?, and you know, even stuff that's from medical sources isn't always you know legitimate as far as like you know if it was a study or whatever. Um so… usually like if the majority of it is like saying that it's good or bad or whatever. Um usually I'll check more than one site uhm… I won't really go to like the blogs and personal (laughs) unless I'm looking for entertainment. But um… yeah, I like… uhm if it's something that I'm really wondering about, I'll go to like the MayoClinic or like something that I trust a little bit more than just straight-up Google (laughs). But uhm, yeah usually I just kind of compare a few different sites and see.
INTERVIEWER: Makes sense.

ADL12: Yeah.
INTERVIEWER: Uhm and where do you typically access the Internet from?
ADL12: Uhm, usually my phone. But if it's a day I'm at home, I'll use the home computer. But typically it's my phone.
INTERVIEWER: And do you have a smart phone?
ADL12: Mhm.
INTERVIEWER: Okay.
ADL12: I have an iPhone.
INTERVIEWER: And you can just ask Siri. She knows everything (laughs) Uhm, do you recall any of your more recent searches that you looked for? What types of information? Doesn't have to be the specific source. Just a general idea.
ADL12: Uhm… I think I did Google the salmon. (laughs) Uhm… well not really during this pregnancy, but I did Google about transitioning from formula to milk for my daughter. I remember I did that just the other day. I'm trying to remember if there's any other pregnancy… I can think of…
INTERVIEWER: It's hard to remember. I can't remember what I ate like the last meal so… kudos to you for coming up with two (laughter). And so you mentioned that you feel like you can trust the MayoClinic. Are there any other websites that are maybe go-to websites if you're not finding what you looking for from whatever Google found.
ADL12: Uhm… well depending on what it is but uhm… like he CDC. I know with my daughter, as far as like you know, opinions on like different vaccines and just like, more medical type things. Uhm… let's see, what other websites do I trust? I do tend to be more skeptical than trustworthy (laughs) of websites. So there probably aren't any other like specific go-to ones.
INTERVIEWER: Uhm… have you ever gone to Babycenter.com at all?
ADL12: Mhm.
INTERVIEWER: Is, do you find that that's a useful resource?
ADL12: I do, but once again I think it's mostly just like blog-type you know like parents' opinion. They do have like the uh… different things that you can click on as far as like you know information for the different stages of pregnancy. But a lot of times, if it's like a specific question, it seems to come back with like you know, specific like conversations that have gone on about it, or stuff like that.
INTERVIEWER: I see.
ADL12: But yes, I have. (mumbles) that one and the bump, pretty much I have been on them all at one time or another
INTERVIEWER: Okay uhm and … you already kind of answered these… have you joined any email or text message or Twitter apps at all? Or I guess on Twitter you're actually following.
ADL12: Uhm… I get… I think I get emails from the whattoexpect like through the app, and I do get some alerts… I just got one randomly the other day… I…yeah it was like the first one that I got, so it was like hm, I don't really know where this is coming from. It was something about the baby's legs growing or something. Um but I'm assuming it must have been through whattoexpect because I don't remember signing up for anything else.
INTERVIEWER: Okay. And uhm I know that you said you're skeptical of things that are on blogs, but do you follow any blogs or Facebook groups for more of like… a group of woman and?
ADL12: Yeah. On the app, you can join like the month that you're due. (INTERVIEWER: Mhm) So like… I'm in the month I'm due and the month I had my daughter. So I go on those on

a daily basis and just kind of see like what people are asking and. Especially with my daughter like… it's just like very helpful to see like, you know how people are listening to this or what kind of foods they're feeding or uhm… so I do go on those on a daily basis (laughs).
INTERVIEWER: That's good.
ADL12: Yeah.
INTERVIEWER: And I'm just curious, is it something where members of your kind of delivery group talk amongst each other and like it's supportive or… is it more just like, I have this question and people answer?
ADL12: Uhm… well it's funny cause it kind of transitions like in the beginning… it's very like, I don't know, it starts off very cut-throatish, like people are not very nice. (INTERVIEWER: Oh..) And it's basically like anyone can start a conversation, and when you go into a group, you can see a list of like the most recent conversations. And if the more people comment on it, it stays up toward the top or it kind of like falls to the bottom. Uhm… so the group for like August, you know like someone will post a question saying, "I dyed my hair. Like… do you think I should be worried?" And you know some people write like, "You're so stupid. Like you know your baby's not going to die if you dyed your hair." You know like, and other people are like, "No, you're fine" or "I would call your doctor." So it's kind of like all over the place in the beginning I feel like, but now like my daughter's age group, they're turning one, I feel like it's now like everyone's now really nice and it's much more like supportive. Like maybe all the meanies died off by now (laughs). Uhm but basically anyone can post any you know topic and you just click on it if you want to read. But I wouldn't say it's an overly supportive group. (laughs)
INTERVIEWER: Would you say that your engagement on that site is more passive? Where you read, you don't really contribute to the…
ADL12: Yeah.
INTERVIEWER: Okay.
ADL12: Like I probably commented a couple times, just on like, you know, cloth diapers, or you know something that's very neutral. (laughter) But no, I don't post on it a lot. I just like to read it and kind of see what you know peoples' opinions are and whatever. Not so much the hot topic type conversations (laughter).
INTERVIEWER: Uhm, do you think that there's a certain type of person that responds like regularly to those sorts of things or posts compared to someone who's more of a passive engager?
ADL12: I don't know. I've wondered about that. Like… cause there's some people that literally, like, the mean people. Like they know them. Like they know their screenname, cause like they're always posting about it. And I'm just like, I don't know like what kind of person would just like be a mean blog poster like so often that like, that's what they're labeled as. And they're okay with it… so I don't know if. Just maybe people who don't have support around them, they are searching for it there? Or I don't really know but I have wondered that myself. Why? Cause there are some people that like they know each others' screennames by name cause they post so often whereas… I think posted like twice and I don't really associate any specific name, um but I don't really know about well… personality. I think for me, if I like really have a question, I just ask like you know someone that I know and someone that has had babies rather than someone who posts it on there… so I guess maybe that's one difference. (laughs)
INTERVIEWER: Uhm in the e-mails that you have gotten from what to expect, do you find that information is helpful?

ADL12: Yeah, I like it. They send them once a week, uhm, and… it's just like helpful little tips. They're usually telling you like you know, what's going on for growth and development for the baby… uhm and then they'll usually have like some topic at the end. You know, something uhm random, but yeah I always like to read em, and kind of see what, what size the baby is and what they're currently growing, and all that kind of stuff.
INTERVIEWER: And so as far as people go, um you talked about your sisters and that they're a good resource for you to talk to and ask questions. Uhm how does your midwife kind of fall into the whole realm of y'know, talking to them for advice and uhm… I don't know. I'll just let you talk about it. (laughter)
ADL12: Uhm I feel like I ask them the bigger questions, or uhm more things that are not so like trivial, like can I eat this or not (laughs). Um and I mean, I've been lucky. My last pregnancy was very normal so to speak, and so far this one has been too, so you know, I don't really like have tons of issues and questions. Uhm… but usually you know, if I do think of something I will ask my sisters first. Like this little red dot I've been getting on my face since I've been pregnant. Um so I asked my sister about it, and she said, "Oh, mention it to your midwife." So I was like okay. (laughs) So yeah, typically just like bigger, more important questions (laughs) I save for them.
INTERVIEWER: That makes sense. Uhm have you been given any advice about weight gain during pregnancy?
ADL12: Uhm… just kind of a range. Uhm 25-35 is kind of what I should gain given my like height and weight and… I mean that's kind of normal across the board, but that's what they said I should.
INTERVIEWER: And who, who said that?
ADL12: Uhm in the first midwife appointment, um they talk about kind of like expectations of um what to gain. Because with my daughter, uhm I did gain 35, and my OB at the time told me that I was gaining too much weight, but my midwives here said that that would be fine. Um so yeah, that's kind of the range I'm hoping to stay in.
INTERVIEWER: Uhm and is that something with each visit they kind of check in and just talk about the progress, or…
ADL12: Uhm I mean you get weighed every visit. Uhm in the beginning I lost weight cause I was so sick, so they were a little concerned that if I kept losing, which I knew I wouldn't cause the same thing happened with my daughter, you know I initially lost ten pounds, and then you know um gained normally. So other than talking about that, they haven't specifically uhm mentioned anything. I think if it was like a big jump you know, so if in one month I gained ten pounds or something like that, they might mention it. But so far this like visit she didn't say anything about it cause I'm finally back to my starting weight so… (laughs)
INTERVIEWER: That's good. I'm glad to hear that. Uhm have—have the midwives ever talked about what kind of… the… downside of gaining too much or too little is… if there's like, what happens to you or the baby?
ADL12: Uhm… I don't think so.
INTERVIEWER: Okay.
ADL12: I don't think they specifically…
INTERVIEWER: Have you heard of any sorts of health issues that may arise if, due to weight gain?
ADL12: Uhm I mean know gestational diabetes is a concern. And that that can lead to an increased birth weight. Um other than that I mean obviously the more you gain the more you

have to lose (laughs) after the baby. Uhm… but as specific like to the growth and health of the baby, I'm not really sure.
INTERVIEWER: Uhm… and you said that this weight range was something that your midwife told you about. But did you already know that before… had learned that from some other resource?
ADL12: Uhm… yeah. I've kind of heard that that is the normal range uhm if you're of normal like weight and everything before the baby. Uhm I'm pretty sure in What to Expect, they say similar if not like the same range. But I know like, in my last pregnancy they told me 25. Like they wanted me… that was what they wanted me to gain. Uhm and I obviously gained more than that, but uhm I think… I think I did read that (laughs).
INTERVIEWER: Okay. And… I mean it sounds like you understand that the more weight you gain, the more harder it can be to kind of lose that after so… is weight gain something that you Google search things about at all online to kind of make sure you keep yourself in check or… uhm…
ADL12: Uhm, not really. I know that like on average, if it's like one pound a week, that that's okay. Especially now that I'm 20 weeks, if I do that for the rest, then I'll be golden. (laughs) Uhm but I mean I wouldn't say that I like look up stuff about it um, especially given that this is my second pregnancy, um but I try not to stress out too much about gaining weight um just cause I know that it's bound to happen. But as long as… I feel that as long as I'm eating healthy and still like exercising, I shouldn't have to worry about it.
INTERVIEWER: I think that's a good assumption. Um so that's a good segway to talking about diet and exercise, so um just to start, who generally prepares the meals and snacks that you eat?
ADL12: Uhm, me.
INTERVIEWER: Okay. And do you also do most of the shopping?
ADL12: Yep.
INTERVIEWER: And… uhm anyone in your household that offers any help in the decision making of what kind of groceries that you buy?
ADL12: Uhm I mean every once in a while, my husband will give me some specific things, but for the most part, by this point I kind of know what… what we eat and I do most of the meal planning anyway so… he's not very picky.
INTERVIEWER: That's good. Um how much money do you normally spend each week on groceries?
ADL12: Uhm I would say, probably around $100 a week, on average.
INTERVIEWER: And… can you tell me a little bit about how you've been eating during this pregnancy? So I know that you mentioned that you had some nausea in the beginning, but…
ADL12: Yeah, so I was pretty sick uhm mostly in the mornings from like 6 to 17 weeks, so uhm I would throw up usually once or twice every morning. So typically I wouldn't really eat until like 10 or 11 depending on when I could keep food down. Uhm and… early on, like not much appealed to me, so I was eating basic like bagels. You know usually I would toast a mini bagel and try to eat that around 11. Uhm, I typically eat cereal for breakfast but could not do that until just recently just cause of the milk. Um and then… I usually could eat uhm… some lunch. I've liked fruit all along, especially like frozen fruit in the beginning when I was nauseous. So I would eat a lot of like frozen fruit um… I would also eat a lot of popsicles (laughs) Uhm and then dinner, like smells really bothered me in the beginning so I didn't do a lot of cooking for my poor husband. Um so I would just, I mean like, peanut butter and jelly. Just like very basic in the beginning. Uhm… but then once I was feeling better, um like around week 17, now I'm kind of

back to my normal eating habits. So now I usually would have oatmeal or cereal for breakfast. Uhm… I try to keep a lot of fruit around um cause my daughter um yeah, she eats a lot of fruit too. So yeah, usually in the morning, I'm hungry like an hour or so after I eat. So I'll have like a banana or an apple or something like that. Uhm… and then… lunch is usually like some sort of like sandwich, like chips. Uhm, granola bars. Usually if I'm working, I'll have a stash of granola bars. Uhm, and then dinner I try to… make a variety. I mean, we eat a lot of chicken mostly, but uhm, I do make like ground beef stuff, fish every once in a while. Not on a super regular basis. Uhm and then just like rice, pasta… we're very not picky people and we kind of eat a little bit of everything. Uhm, but yeah. That's pretty much it so far. I didn't eat many vegetables until probably just a couple of weeks ago just because nothing… no vegetables appealed to me. But now I can get back to them.
INTERVIEWER: Was it the smells of the vegetables, or the taste? Or…
ADL12: Uhm not really the smell, but yeah just the taste uh didn't appeal to me at all. Fruits have always for the most part, unless they were really acidic, but fruits have been fine. But yeah, they just didn't… I just couldn't get them down. (laughs)
INTERVIEWER: (laughs) So now that you're um feeling better and able to eat more regularly, are there any modifications to your diet that you've made to I guess… a conscious change… because you know that you're pregnant, that you feel that something you've added or changed has had a health benefit for you?
ADL12: Uhm the biggest thing is really trying to make sure that I drink enough water. Uhm… cause I find that, esp- when I'm at work, I actually think I drink more water. But when I'm home, like I just don't think of it as much, which would probably be the opposite. But um. So that's the big thing that I make sure I try to drink um water. Uhm… I typically drink coffee so, I just limit myself to my coffee in the morning. If I want coffee later, I just have decaf. Um and as far as eating habits, I mean I cut out… um you know like the things that you're not supposed to have. And I do microwave my deli meat because I … that's like my go-to lunch. I love like turkey sandwiches, so I um do microwave my deli meat. But um other than that, I try to eat mostly healthy in general, so uhm… I do if I like have a day where if I knew I ate bad, I would really try the next few days to make sure I'm eating like really healthy (laughs) Which I may not do.
INTERVIEWER: Um so the microwaving of the lunch meat, can you tell me more about that as to why?
ADL12: So… they… I think listeria is the bacteria that grows, so they say that um by microwaving it, you kill it. But apparently it's uhm listeria itself can um… I think they say it can actually like kill the baby? But it can cause a lot of harm, but you don't get sick at all. So it's like the baby can get sick, and you have no idea. Uhm, but I mean a lot of people disagree and say that.. .you know, um that it's not really as much of a risk. I mean it's mostly like if the meat is left out or you know like not…
INTERVIEWER: Not refrigerated…
ADL12: Yeah, like not properly. Uhm refrigerated throughout like you know the process of the store buying it and all that stuff.
INTERVIEWER: Oh… I didn't really think about that.
ADL12: Yeah, so um, but for me, it's an easy thing to do you know like. So… uhm that is something that I do. I'm not sure if I have any other modifications.
INTERVIEWER: How long do you have to microwave the meat? Not very long?

ADL12: Uhm I usually just microwave it for like 20 seconds, cause usually after that it starts popping and (laughs) Uhm and I mean I figure if it gets to the point of steaming, that it has gotten to the point where it can kill the bacteria (laughs)?
INTERVIEWER: That's probably a safe bet.
ADL12: So yeah.
INTERVIEWER: And in terms of the quantity of food that you're eating, how does that compare to… prior to pregnancy?
ADL12: Now, I'm definitely snacking. I'm eating more snacks. I do try to eat healthy snacks. I try not to you know eat cupcakes every time I get hungry. Um but I definitely like will eat like breakfast an an hour or two later… Like I feel like I'll need to eat again. But yeah, that's definitely the biggest thing.
INTERVIEWER: And just out of curiosity, what, whatdo you think it means when your body is telling you that you're hungry? I mean like… I guess just the first thing that pops into your mind. That's probably a weird question, I'm sorry (laughs).
ADL12: (laughs) Uhm… I don't know.
INTERVIEWER: Like do you think it means anything specific, or is it just…
ADL12: I mean there will be times when I'm like really hungry and I can tell that like my body needs some like sugar, you know like I start to feel shaky. Especially if I have like coffee in the morning and haven't had anything and then I start to feel like I really need to eat something. But other than that, uhm, maybe it has something to do with my blood sugar or just my stomach is just not as full as it wants to be.
INTERVIEWER: Do you think that if you were hungry and and you chose not to eat instead, do you think anything bad will happen from that?
ADL12: Uhm, no. I think my stomach will just growl (laughs). Umm…
INTERVIEWER: Makes sense.
ADL12: I don't think anything probably would happen.
INTERVIEWER: Uhm and do you think that a, a pregnant woman needs to be careful about what she eats? I know we talked about lunch meat and listeria, but are there any other things you need to be really careful about?
ADL12: Uhm… I mean I try to make sure like most things that I'm eating are like whole grains uhm… and eating like a variety but, as far as like things that I shouldn't eat… (mumbles) (laughs) Yeah I think for me lunch meat is the biggest one.
INTERVIEWER: I know you mentioned salmon earlier, that you had looked that up.
ADL12: Yeah, I know that there's like a bunch of different uhm… I don't know all the ones that you're not supposed to eat. I just know that salmon is okay… I think they say that that once a week uhm is fine, but maybe that's the crab or the shellfish. Um but like tilapia… we do have that uhm. I think they've said that's fine. Um just making sure everything's cooked through, like burgers, if we go out to eat that they're well done.
INTERVIEWER: That makes sense.
ADL12: Yeah, uhm yeah.
INTERVIEWER: Uhm and what is it about um fish? Is it bad for you or bad for the baby? Uhm do you know what it is about it?
ADL12: Uhm I know that certain fish like I think shark is one of them have a high mercury level, so um that can be transferred to the baby I'm pretty sure. Um so that's… I don't know if there's any other reasons. But I know that mercury level fish and like different sea life have different mercury levels… are the ones that you're supposed to avoid.

INTERVIEWER: Okay. Uhm and have you ever heard anything about um needing to have your diet change as you move through pregnancy that certain foods become less or more important as your baby develops?
ADL12: I don't think so.
INTERVIEWER: Um and have you changed how you prepare foods because you feel it's better for the baby or um worse for the better? An example would be like mu how you cook meat. So baking versus frying, or those sorts of things.
ADL12: Uhm for the most part, I think I try to um like bake or grill most of the time. Like typically if we have chicken, it's grilled you know whether it's like the foreman in the winter or the actual grill itself. Um I really don't, I will rarely fry things and… um and I kind of consider baking and grilling both healthy options (laughs)
INTERVIEWER: I would agree.
ADL12: But I don't really fry many… every once in a while I would do a breaded chicken fry but, not very often.
INTERVIEWER: And is salt and sugar you think more about now that you're pregnant? Or do you not really worry so much about that? It's more about if you're hungry and if it sounds good to eat, then you just eat that.
ADL12: Uhm I try to be conscious of it, and basically how I try to be more conscious of it is eating more fruits and vegetables than like… even though I always do have granola bars. You know like I know those do have sugar and preservatives cause they're you know on the shelf for who knows how long. So I try to eat more like natural I guess, so to speak, snacks than like the packaged, um processed snacks. I wouldn't say that I like compare so much as far as like which granola bar is the healthiest. Uhm and then like salt… I don't ever really add salt to my food. And then as far as like eating, I guess I don't think about salt as much as I do sugar (laughs). Uhm… yeah. I think I think more about sugar.
INTERVIEWER: Has your dairy consumption changed at all? I know you mentioned that you weren't able to have milk and cereal early on because of nausea but is that um… I guess do you like it less or more, or is it about the same?
ADL12: Uhm probably about the same as before. Uhm I usually don't drink milk, like typically I won't just have milk in my cereal. But you know I like to eat yogurt um and cheese, but I would say it's probably about the same as before.
INTERVIEWER: Uhm and have you had any cravings for nonfood items like paper, clay, uhm…
ADL12: (laughs) Thankfully no
INTERVIEWER: Um chewing on ice at all?
ADL12: No (laughs).
INTERVIEWER: You had mentioned that you had liked popsicles. Is that just because they're sweet and fruity? Or is it because you like chewing? And like frozen fruit, you said also.
ADL12: In the beginning, well like for the fruit it just… I'm not sure why I could eat like frozen peaches, but to eat an actual peach wouldn't appeal to me. Uhm… and then the popsicles… they were lime flavored popsicles that I buy. It was like if it was kind of later in the morning and I was not sure if my nausea was quite gone, nothing would really appeal to me, usually if I had one of those, that would like taste good to me and then after that I would feel better. Not sure what it was about the lime. Uhm I also read that raspberry was supposed to help with nausea. But I didn't find, I mean those popsicles were good but… for some reason I felt like the lime really did help my nausea to like subside. But uhm I haven't (coughs) I haven't had any popsicles since

my nausea went away. Um but those were kind of just a go-to. Something that I knew would most likely be okay. (coughs)
INTERVIEWER: Um and just…
ADL12: (coughs) etc. Sorry
INTERVIEWER: Um so along cravings kind of go with smells that you like more or smells that you really can't stand? Is- so beginning of pregnancy some things were bothering you…
ADL12: Like everything.
INTERVIEWER: Everything?
ADL12: Even like toast.
INTERVIEWER: Oh gosh.
ADL12: Like I would open the bag of bread, and it would make me throw up.
INTERVIEWER: Aww that's no fine.
ADL12: But now I'm good.
INTERVIEWER: Is anything else though that's like popped up that you liked the smell of more or something that you still don't like the smell of?
ADL12: Uhm I don't really think there's anything I liked the smell more. And I don't think anything still bothers me.
INTERVIEWER: That's good.
ADL12: Yeah!
INTERVIEWER: Uhm and are you taking any vitamins or extra supplements right now?
ADL12: Um just prenatal vitamins.
INTERVIEWER: Okay. Uhm so let's talk a little bit about physical activity. Um have you heard that a woman needs to change her physical activity when she's pregnant? Or I guess what have you heard about physical activity during pregnancy?
ADL12: Uhm, I think… well I know they say like as far as different activities, if you were doing it before, you can continue along with it as long as it's not like, a contact sport or uhm, you know like… too strenuous causing pain or anything like that. Uhm, I assume they recommend that pregnant people exercise. I don't really know what they specifically recommend. Uhm and as far as like changing I guess, if you didn't exercise before, they would probably recommend that you do some sort of exercise. Um but I don't know specifically what's recommended.
INTERVIEWER: That's okay. Um could you tell me what you do during a typical day that gets you up and moving around?
ADL12: Uhm… I try to go on walks as much as possible now that the weather's nice, hopefully we can go on walks almost everyday. Uhm, on days when I'm home, you know just everyday things with my daughter but then I do try to um, do just some light you know like, squats, lunges, things like that. I was doing the uhm, this app on my phone, I think it's seven minutes where you do stuff for 30 seconds where you like keep it up. I was doing that because since we've moved back to Rochester, I haven't had a gym membership uhm, because I just wasn't sure with Lilia how you know how much I would be able to get there. And I got pregnant and I would probably use that even less. I would just wait until after the baby's born. Uhm I know when I work—I work in a hospital so I would just always take the stairs. I feel like that's the one little good thing. That I'm walking all over. I work all day.
INTERVIEWER: Um has your activity level changed with this pregnancy compared to before or with your previous pregnancy?
ADL12: Uhm I mean in the beginning I did not do much of anything, uhm just because between being not feeling good and between being like just so tired, and uhm and then once I started to

feel better and get my energy back, I just kind of wanted… you know I feel like I want to be more active. So I'm kind of in that stage right now where I'm feeling better. I'm like okay, let's get out and do some more stuff.
INTERVIEWER: That sounds nice, especially now that the weather's getting a little bit nicer, too. It makes it easier to actually do something.
ADL12: Yeah.
INTERVIEWER: Uhm… how much did you exercise before being pregnant compared to what you can do now that you're pregnant?
ADL12: So before… I mean before I had my daughter, when we were just living the dream, being childless, I would go to the gym 3-4 times a week. Um I did spinning once a week, would run, lift weights. Uhm… I was very fit. And then after I had my daughter, I still ran uhm… you know once I was cleared (I think, six weeks, you're cleared), uhm but I definitely didn't do as much lifting. And now I don't really do any lifting. I basically do bodyweight you know stuff like squats, planks, pushups, stuff like that. And then I haven't been running so I probably won't really run um just 'cause you know, they say that anything you're doing, you can continue. So I worry about not running (????) and then starting back out. I don't wanna, I don't know. It probably would be okay if I took it pretty slow. But that worries me so. Uhm, usually I just walk, and I can do that with Lilia so… (laughs) it works out.
INTERVIEWER: That's good. It does seem confusing, because if you took like a break from something, then how do you know when it's something that you did before versus when you took a short hiatus and then technically you're still in… they don't really tell you much about what it is.
ADL12: Right. Cause like for three months basically, I was really not doing anything. So yeah, running is the one thing that I like worry about doing.
INTERVIEWER: Yeah. That makes sense. I've heard that your balance changes, and like your joints are a little bit different.
ADL12: Yeah, and I also have some SI issues in my lower back. So… usually if I take a long walk, that night I'm kind of like hobbling around so I'm like… I don't really know if I'm quite stable enough to run.
INTERVIEWER: (laughs) Uhm, let's see. So how, how much… how many days a week do you think that you're doing something now that you're feeling… up to kind of… back to your normal self I guess.
ADL12: As far as exercise?
INTERVIEWER: Mhm.
ADL12: Uhm… I try to do something every day.
INTERVIEWER: Okay.
ADL12: Uhm… you know sometimes it's just, some stuff in the living room while [name redacted] is napping or um… you know. Taking a walk. But I do try to do something every day that makes me feel like I'm doing something exercise-like.
INTERVIEWER: Mhm. Have you seen anything in the news about research or anything that shows physical activity has some sort of a benefit for you or the baby?
ADL12: Uhm I've definitely read, and I'm not sure if this is like expert or just opinion that the healthier you are, the more strong and fit you are, the better labor will be. I felt like I was in great shape when I had my daughter and (laughs) the labor was horrible and very long. Um but I mean I think it makes sense. Obviously you need your abdominal muscles, your hips, you know everything to push out a baby. So the stronger things are, it seems like the better uhm… that's

probably the biggest thing. And also getting into shape after the baby uhm… you know. I think a lot of people say that. It helps.
INTERVIEWER: And are there any exercises that are maybe best or better to do when pregnant, as far as benefit for the baby?
ADL12: Uhm… I know kegels, uhm. And just stabilization um exercises like planks and things that still like work on your core and arm force and you flex like a… crunch would. Um squats and like hip strengthening exercises or something. I try to do especially since I have some SI pain um and issues… uhm I'm a physical therapist so I think about those things anyways. So I know the stronger my pelvis and core is, the more stable I'm going to be as my ligaments start to loosen and things start to separate, essentially.
INTERVIEWER: Um and any activities a pregnant woman should not do. I know you talked about contact sports, but any others that you can think of?
ADL12: Uhm I know that you're supposed to avoid like um ab exercises like crunches, things like that. Uhm… I know once you get to be like a certain… I'm trying to remember about when. But once the baby gets to a certain size, you aren't supposed to do a lot of stuff. Laying flat on your back, cause they can lay on your main artery and occlude blood flow. Uhm… so I know once you get towards the end, they say not to do as much laying down. Uhm… and typically I… well in my last pregnancy, was cutting down like my weights um when I was doing like lifting and things like that. And just um being more conscious of the load on my body.
INTERVIEWER: Uhm and then just one final question. Uh so just being in this study, what are some things that… I mean you know it's a social media study with Facebook and text message… uhm and thinking about that. What sorts of things are you thinking to get out of it?
ADL12: Uhm I'm really hoping just to get more information on the nutrition aspect. Cause I consider myself educated, but when it comes to like… specific nutrition things, I feel like I kind of have a broad knowledge and not really like just a specific like… I don't really know how to say it. Like the questionnaire I filled out that first day, I was like oh my goodness. This is really sad; like I don't know the answer to these questions. But uhm… just I think when you're pregnant, you're more conscious. So… you know just getting more knowledge about nutrition and uhm proper eating in general. Uhm…
INTERVIEWER: Our goal is hopefully to make sure that you know all the answers, and if you don't, we probably did a bad job so… (laughter)
ADL12: I told my husband. I was like oh my goodness, there were these questions about like… I don't even remember what they were. I was like I didn't know the answer.
INTERVIEWER: That's okay. You don't have to know the answer. It gives us something to tell you about then.
ADL12: (laughs) Yes, that's what I'm looking forward to.
INTERVIEWER: Um and is there any way you like to receive information, whether it be links to a website or an article or a video or a picture or just a sentence or two?
ADL12: Uhm I guess everything. There's not really any like specific uhm you know, usually if someone sends me an article, I'd like a little synopsis so that I don't need to read all of it if it's like…um
INTERVIEWER: So you can decide if you wanted to.
ADL12: Yeah. But no, I'd say all those forums sound good.
INTERVIEWER: That's good (laughs). Those are our plans. Um and then anything else that you maybe thought of after a question that you didn't get to say that you just want to go back and touch on really quickly?

ADL12: Um I did wanna ask like when the text… are they supposed to like start at a certain time?
INTERVIEWER: As soon as you're done with this interview, (ADL12: Oh okay. Okay) so probably tomorrow would be the first day you'd get messages.
ADL12: And then as far as the Facebook group, um is it just like you guys post stuff and we like comment about it, or is that mostly the…?
INTERVIEWER: Um so I guess I should say our, our goal is that… I mean, it's a group so um. We post things and a lot of the times it's a question or a poll, and we try to engage people.
ADL12: Okay.
INTERVIEWER: Um I'll be honestly and say most people seem to just look at it and I think passively engage. So there's not a lot of conversations, but it's not because we don't want there to be.
ADL12: Yeah.
INTERVIEWER: Um we just sort of put things out and see what people do with it.
ADL12: Okay.
INTERVIEWER: We're not, we don't require that anyone ever participate or answer, but um if we do like a poll question, I think a lot of people normally answer those. Um and I know on the Facebook agreement we said that we would like people to try respond if they can, but it's not obviously required, um so. So then the text is more of a just to provide information, and then Facebook was to have more of a group dynamic support network. That was our goal anyways. I'm not sure if we achieved that but, it's a work in progress.
ADL12: Okay. Gotcha.
INTERVIEWER: Um any other questions or anything?
ADL12: Um I don't think so.
INTERVIEWER: Okay. Well I think that's.

RDS27

INTERVIEWER: So I am going to start by asking you some questions to describe you and your pregnancy. The first question is: How old are you?

RDS: 18

INTERVIEWER: Okay, and how tall are you and how much do you weigh?

RDS: 5’2” and I weigh 198

INTERVIEWER: And is this your first pregnancy?

RDS: Yes

INTERVIEWER: and would you classify your pregnancy as one that was planned?

RDS: no

INTERVIEWER: what hope and goals do you have for your baby?

RDS: to be healthy and that’s basically it

INTERVIEWER: do you ever imagine what it will be like in the future?

RDS: no

INTERVIEWER: just waiting for the day

RDS: yeah

INTERVIEWER: so once you have the baby any challenges you expect to face?

RDS: waking up in the middle of the night, the sleeping part

INTERVIEWER: any challenges you’ve faced while being pregnant?

RDS: no I don’t have too many challenges

INTERVIEWER: anything that changed in terms of your schedule?

RDS: probably sleeping, I used to be able to sleep in the middle of the night but he’s up and active so I get up and then I try to go back to sleep

INTERVIEWER: What do you do to try to go back to sleep?

RDS: toss and turn

INTERVIEWER: what are some important things to be doing for baby's health?

RDS: Eating healthy, resting, making sure to take all the pills I am given, iron, prenatal and study pills

INTERVIEWER: Given the things you've put value on like eating and sleeping and taking your supplements, what has your care been like at the clinic so far?

RDS: It's been good, everyone is really nice and helpful so I've had a good experience so far.

INTERVIEWER: Have they given any advice on eating, resting and pill taking?

RDS: Yeah I don't take pills besides the ones I'm prescribed, they rather I eat healthy more than fatty foods and take out. I eat a lot of take out cause recently I work so they'd rather me cut back are trying to keep me on a healthy diet and maintain a good diet

INTERVIEWER: Lets talk a little more about eating and diet because it sounds like they've given you a little advice about that. So you said that you've changed you've eaten a little, so can you elaborate on that?

RDS: At first I ate probably 3-4 times a day, more healthy meals, fruits for snacks and stuff, but because I work and my hours, they way that they are, I don't cook or taking anything from the house cause I wake up early to catch the bus so I order food but she told me to try to eat more healthier and see it helps

INTERVIEWER: So what have you tried to change specifically that are helping you do that?

RDS: Um, nothing yet

INTERVIEWER: Still working on that?

RDS: Yeah

INTERVIEWER: What challenges are there from keeping you from making changes?

RDS: Not really a challenge I mean I could eat healthy but instead me cooking I just order food so I've been tired my hours are just not good hours so I don't have time to do too much, probably it the hours that I work make me tired so I don't cook anything or prepare anything so I order food.

INTERVIEWER: What kinds of things do you normally order?

RDS: I had burger king this week so like fries and burger, and I had a fettuccini from work out of one of little freezer things they had in there that was probably it, or I usually just eat fries

INTERVIEWER: How do you eat your fries?

RDS: They fry them at the job so that's probably why it's not a good idea

INTERVIEWER: Where are you working?

RDS: [place redacted]

INTERVIEWER: So even though it sounds like you're tired and time is an issue, time is important, have you ever changed anything you order in thinking about trying to be healthier?

RDS: Yeah yesterday I had a yogurt, a yogurt parfait with granola, and they have fruit containers, not strawberries, they have like watermelon, I try to eat less fatty food or foods that aren't good

INTERVIEWER: How long have you been trying to make those changes?

RDS: Just since yesterday

INTERVIEWER: Ok. Is there anything that would help in making healthier choices when you're out, what are you looking for in food when you make your choices?

RDS: Nothing specifically I don't really have a preference. I don't get home until 1 so I'm not up trying to prepare something I'm just trying to go to sleep

INTERVIEWER: What are the most important things? Like taste? You've mentioned time

RDS: I don't know. Can you ask in a different way?

INTERVIEWER: If a restaurant where you eat out at or order in from had two of the same option but one was healthier would you ben open to trying new one or would you stick to your favorites?

RDS: Yeah I'd be open to the new one but if I don't like it I probably wouldn't eat it again

INTERVIEWER: Okay, so when you are home if you have cooked who does the shopping for that food?

RDS: Me

INTERVIEWER: Do you have any help in making decisions for the groceries that are purchased?

RDS: No

INTERVIEWER: Are you living alone?

RDS: Yes

INTERVIEWER: How much money do you spend on food per week?

RDS: I don't spend much money cause I have food at home but this week I probably spent like $20 cause we don't get to many breaks we do have a break the cafeteria is closed so I have a couple snacks in my bag but this week that's when I ordered food cause I haven't prepared anything

INTERVIEWER: Do you have to pay for food in cafeteria?

RDS: Yeah

INTERVIEWER: Okay, so you mentioned that before you were pregnant you were eating 3 meals a day, how has your eating changed since you became pregnant?

RDS: Like before, I ate like 3 meals a day not too much of healthy foods like I like fruits an certain vegetables so now I don't eat like 3 times a day I eat when I get a chance to eat basically I have snacks here and there like ill go get a yogurt cause I like yogurt and then ill have a banana and that's it.

INTERVIEWER: All day?

RDS: No like ill eat probably a meal and then depending on what time we have our break ill have a banana or snack until I get home and then when I get home its 1 so I have like a cereal or something and then that's it

INTERVIEWER: So what about breakfast, do you eat breakfast?

RDS: No, cause when I wake up by the time I get to work they're done preparing breakfast and by the time I'm ready to go I have to get on the bus.

INTERVIEWER: Can you walk me through a typical day for you? I guess pick an average day and say I would get up eat this at this time

RDS: I would probably get up in the mornings, when I get up I never really eat breakfast cause I get up at like 10 I be tired from getting off so late, get off at 1 sleep till 1 get up and shower and stuff then its time for the bus. I get on the bus I go downtown I wait for the bus probably go in the store and grab a bag of chips and I don't eat it then and there I wait until work and then I go to the cafeteria and get a cappuccino and a banana. We don't have a break until like 7 we have a 15-minute break at 5 and during that time I be on the computer. Then at 7 we have a 30 min break and the cafeteria is closed I go to the vending machine and when I get home if somebody would have cooked ill probably warm something up or eat a bowl of cereal and go to sleep

INTERVIEWER: You said somebody would have cooked, who would have cooked?

RDS: After work I go to my grandma's house before home

INTERVIEWER: What does she normally cook that you eat?

RDS: I don't know, yesterday she cooked macaroni. Usually she does cook a whole meal. I don't really eat everything that they cook so I don't know it depends on what I like

INTERVIEWER: Why don't you eat everything they cook?

RDS: Some things I just don't like

INTERVIEWER: What don't you like?

RDS: I don't know depending on the way things are cooked I don't like it. I don't like how everybody cooks personally its not the food jus the way they cook it or seasonings its just the way they cook

INTERVIEWER: That makes sense. You said that you liked some vegetables, which vegetables do you like to eat?

RDS: Carrots, salad, broccoli, corn, tomatoes cucumbers that's it, cauliflower yeah that

INTERVIEWER: Raw or cooked?

RDS: Both

INTERVIEWER: Me too. Tell me about your favorite fruits too.

RDS: I like apples, strawberries, watermelon bananas

INTERVIEWER: How often do you fit those sorts of food into your diet?

RDS: I usually have a fruit everyday, probably now I have a fruit about twice a week

INTERVIEWER: Have you changed how much f and v you've eaten since you became pregnant?

RDS: No

INTERVIEWER: In terms of quantity, would you say you're eating more food now or has that changed?

RDS: On my days off I eat more food, on my regular days I eat less, appetite increased but I am not able to eat every time I am hungry. I don't think I should because I would probably gain more weight then necessary

INTERVIEWER: How much weight do you think is necessary to gain?

RDS: I don't know they gave me a range they said between 15-20lbs so trying to stay in between there

INTERVIEWER: How's that going?

RDS: I haven't went over 15 I gained 14lbs all together so far they told me to try to maintain that, at first a lost weight it took me a while to gain it and then I gain it and all I have to do is maintain it

INTERVIEWER: Why do you think that you lost weight initially?

RDS: I have no idea

INTERVIEWER: Were you feeling sick at all?

RDS: Not really I just I don't know

INTERVIEWER: How about emotionally, were you sad?

RDS: No I just sometimes have an increase and then decrease and then it will stay

INTERVIEWER: We in terms of who gave you advice for weight gain range was that the clinic?

RDS: Yes

INTERVIEWER: Did they tell you anything else like why it's important to stay in that range?

RDS: Um basically said like something about health issues and if baby gets bigger than I will have a harder labor and I have no idea why else

INTERVIEWER: So you alluded to this but would you say you're hungrier now than before pregnancy?

RDS: yeah

INTERVIEWER: how would you describe the difference?

RDS: I would say I get hungry fast, not too long after I'm hungry and before I was very active so I didn't really sit down enough to think that I'm hungry and I just ate

INTERVIEWER: Were you working before you were pregnant

RDS: No

INTERVIEWER: So you started working after?

RDS: Yeah

INTERVIEWER: Have you finished school?

RDS: I do like young mothers, I had started already but since I moved to my own apartment they haven't set up my bus yet but eventually once that's in place then I will be going to school again

INTERVIEWER: How will working fit into that?

RDS: My new schedule will be 330-12 and now I work 2-11 and the bus cause I work in [place redacted] I have to catch a bus an hour earlier cause I wont make it to work

INTERVIEWER: Okay, have you received any dietary or weight gain advice from people other than clinic?

RDS: No

INTERVIEWER: What about grandma does she offer advice from when she had a baby

RDS: No

INTERVIEWER: Friends in young mothers and clinic?

RDS: No I don't really know anyone from the clinic and young mothers

INTERVIEWER: Do you feel like people are very supportive of your pregnancy?

RDS: Yeah

INTERVIEWER: You don't sound very happy

RDS: I'm tired

INTERVIEWER: Okay, so what about phone or Internet, do you look up pregnancy questions

RDS: No I get emails so I just read about it then and if I have questions I ask the doctor

INTERVIEWER: Where do email come from?

RDS: I don’t know from the study, some are websites, I don’t even know what I’m signing up for, they send baby books and I looked through those

INTERVIEWER: Have any of them had content you liked or was memorable?

RDS: No my memory is bad I don’t remember a lot

INTERVIEWER: You said baby center, I’m familiar with that one do you remember any other names of any other books?

RDS: No and I just looked at one this morning.

INTERVIEWER: Do you remember any topics that stand out to you that you enjoyed reading about or were helpful?

RDS: I didn’t get to read the books that came in recently but usually talk about food to eat to have healthy pregnancy and breast feeding and I’m interested in that so that’s good

INTERVIEWER: Based on what you’ve read about or heard about at clinic are there any things that you think pregnant women should be careful about doing?

RDS: Not exercising, lifting heavy items, maintain diet eat less potato chips and less soda.

INTERVIEWER: Are those things that you’re trying to incorporate into daily routine?

RDS: Yeah some things, I don’t even drink soda and potato chips like last week I had a lot and this week I probably had one bag.

INTERVIEWER: Are there any foods that are really good or really bad for baby?

RDS: I don’t know

INTERVIEWER: Have you had cravings?

RDS: No like I like sweet stuff but um I don’t really have too many sweets

INTERVIEWER: Do you think you craved sweet things more than before pregnancy?

RDS: Yeah

INTERVIEWER: What kind of sweet things?

RDS: Probably chocolate

INTERVIEWER: That’s a good one, are there any things you don’t like anymore?

RDS: Probably Chinese food

INTERVIEWER: Is it the smell or taste?

RDS: Smell and taste its something I don't like to eat anymore

INTERVIEWER: Do you crave chewing on ice and non food items?

RDS: No

INTERVIEWER: You talked about reducing fat that you eat, so if you have prepared food or if grandma prepared food is there any effort to reduce fat?

RDS: Yeah sometimes she bakes instead of fries, and usually I like salad if could eat it everyday I would

INTERVIEWER: Have you changed foods high in salt and sugar?

RDS: No

INTERVIEWER: I know you said you're craving sweets how about salty foods?

RDS: Umm I put salt on a lot of things but like when my grandma makes food I don't taste any flavor

INTERVIEWER: what dairy food do you like?

RDS: Yogurt, cheese and I drink milk

INTERVIEWER: How often do you eat those things?

RDS: Yogurt I eat yogurt probably twice, last time I ate it was yesterday but I used to eat it on a daily basis cause I have this big container its just a plain vanilla yogurt and I tried the Greek yogurt

INTERVIEWER: You didn't like it?

RDS: Yeah I liked it

INTERVIEWER: And you mentioned that you've been taking prescribed supplements can you tell me a little about those?

RDS: The prenatal one is one that I take, I forgot what it said my mind is terrible, but I also take vitamin D they gave me iron because they said I'm a tad bit anemic and I need more red meats and I think that's it

INTERVIEWER: Have you changed red meat consumption?

RDS: No I just haven't eaten it recently

INTERVIEWER: Did you try to eat more red meat when they told you you were anemic?

RDS: They told me today so

INTERVIEWER: When did they give you iron?

RDS: At my last appointment

INTERVIEWER: What meats do you usually eat?

RDS: I probably eat chicken, that's the most meats that I usually eat I don't eat them often

INTERVIEWER: Not everyday?

RDS: No

INTERVIEWER: How often?

RDS: Probably twice a week.

INTERVIEWER: So if you're eating meat twice a week and yogurt and bananas and a latte and fries what about bread and pasta and grain

RDS: I like pasta I don't eat it too often but I ate fettuccini yesterday I has spaghetti 2-3 weeks ago

INTERVIEWER: Do you ever choose to eat foods that are fortified?

RDS: I don't know what this is

INTERVIEWER: So a lot of cereals from the box they have added vitamins and minerals that they like to add so fortified means that they add vitamins and minerals to it, they don't occur naturally in the food. So fortified things would be like milk is fortified with vitamin D cereals are fortified with tons of stuff. You said you eat cereal, what cereal do you like?

RDS: I eat cheerios, I like the oats you know those little granola things it's a cereal like that and I like those so I eat those cereal and sometimes I eat sweet cereal like captain crunch

INTERVIEWER: That's a good one. Do you ever think about the nutrients in those foods being better for you or worse for you?

RDS: I don't even think about it I just eat it cause I like it

INTERVIEWER: okay, that's fine. And has anyone in your family or friends offered advice on things to eat because they heard that its good for your pregnancy.

RDS: No, I don't really see too many friends or family besides at night and they're asleep I usually just see my coworkers and that's it.

INTERVIEWER: Do any co-workers share things with you?

RDS: Um, no.

INTERVIEWER: Do you ever do Google searched on the computer or phone?

RDS: yeah when I Google search foods I probably juts look at like fruit salads not stuff that's healthy just stuff to learn how to make

INTERVIEWER: so you like to look up recipes and fun ways to eat?

RDS: yeah

INTERVIEWER: Are there any websites that you go to most often for that

RDS: no I just look on Google I don't really know any other websites that do anything like that

INTERVIEWER: do you ever look stuff up that is a concern for when you're delivering the baby?

RDS: No, cause I don't really ask too many questions about it cause I'm scared

INTERVIEWER: Do you feel comfortable asking the midwives those questions or do you keep them to yourself?

RDS: Yeah I usually ask the midwives if I have a question, I just don't ask other people cause the first thing they say is how horrible it is

INTERVIEWER: What are you most scared of?

RDS: Labor, pushing it out.

INTERVIEWER: Are you excited for everything after that?

RDS: Yeah

INTERVIEWER: A while back you mentioned that it's important to exercise too, can you tell me about your general feeling about exercise?

RDS: I don’t not like to exercise, usually before I would like to exercise but not I get a good amount of exercise cause I walk downtown and in order to get to the bus its on [redacted] and I have to get to east [place redacted] and Its long and I don’t like it

INTERVIEWER: How long does it take?

RDS: Probably like 25 minutes

INTERVIEWER: And you walk that twice everyday?

RDS: No every night except sat and sun

INTERVIEWER: are you up moving around a lot at work?

RDS: At work not really, well I am cause if not ill fall asleep so ill get up and take a bathroom break and walk around a little

INTERVIEWER: So how much walking do you do if you add the 25-minute walking home at the end of the night?

RDS: I am not sure, I just know I catch the bus downtown or sometimes I am not able so I catch a ride downtown I walk to Hazelwood I don’t know how long it takes I know I just don’t like it.

INTERVIEWER: Do you do any other sorts of exercise?

RDS: nope

INTERVIEWER: what about in the young mother program do they have anything for you to exercise?

RDS: Well yeah there they have like treadmills and whatever the other machine is called, you have to exercise cause they give us gym

INTERVIEWER: Do they make it fun?

RDS: yeah like they have those balls, those pregnancy balls, um the exercising part and stuff like that.

INTERVIEWER: Do they have any dancing games?

RDS: Yeah.

INTERVIEWER: Do you like those? Um have you ever heard any recommendation for exercise during pregnancy?

RDS: No

INTERVIEWER: Is that something that the midwives talk to you about

RDS: I don’t think recently we talked about any exercise, its just mainly eat healthy eat healthy

INTERVIEWER: Okay, do you think there is anything that a pregnant woman should or should not do in terms of exercise?

RDS: I don’t know exercise too much

INTERVIEWER: Do you think that they could exercise too little?

RDS: Yeah

INTERVIEWER: What do you think would define exercising too much?

RDS: I have no idea I just don’t like all this walking so that’s why I think that’s too much exercise for me

INTERVIEWER: That’s too much exercise?

RDS: For me I know in reality its not but my legs be tired so that’s how I think in my head

INTERVIEWER: Are you legs tired from sitting at work?

RDS: Yeah they get tired from sitting, like if I sit for too long I have to get up and walk around

INTERVIEWER: Do you think there’s anything that you could do diet wise or exercise wise that would give you more energy that would make your legs feel better?

RDS: Um I don’t know, its not even the energy, it doesn’t come from diet wise it comes from lack of sleep and that’s my problem and then Ill go have a cappuccino and its sweet and it drains me after 20 minutes 30 minutes.

INTERVIEWER: How many hours are you getting a night?

RDS: I don’t know cause when I get home I’m tired and I don’t go right to sleep I take a shower or bath or warm water cause my body be sore and it takes me a while to fall asleep and when I wake up it feels like I just fall asleep.

INTERVIEWER: And you said earlier that you wake up a lot because the baby’s moving around?

RDS: Yeah

INTERVIEWER: If you wanted to know more about exercising where would you go to look for more information?

RDS: The doctors, Google, or just ask peers that exercise

INTERVIEWER: Peers in young mothers?

RDS: Anyone

INTERVIEWER: How about diet? Where would you look for information on that?

RDS: Probably like some of the magazines I receive, doctors, Internet, or ask somebody that has had a similar experience or I don't know

INTERVIEWER: How has your activity level or energy level changed since before you were pregnant?

RDS: before I was pregnant I was active I was always in the go, I don't know it probably hasn't changed too much I still walk the same amount I always walked before. It probably decreased now cause I don't do as many activities as before

INTERVIEWER: How come?

RDS: Cause I be tired now and I don't really think about anything but going asleep

INTERVIEWER: Do you ever make decisions about exercise or eating or weight gain because you're thinking about the baby or is your being tired having a bigger impact on your decisions?

RDS: No like I don't feel that I need to exercise anymore like too much I get enough exercise in my mind, and if I don't get sleep I wont be able to get up and exercise cause I will be totally drained.

INTERVIEWER: What effects do you think diet and exercise have on your baby?

RDS: I don't know, diet probably obesity or could come out with I don't know

INTERVIEWER: So you think diet has more of an effect than exercise?

RDS: I just really don't know

INTERVIEWER: Okay, when you search the Internet do you use computer or cell phone?

RDS: Both at work usually computer cause your not allowed to have cell phones out

INTERVIEWER: Do you have a computer at home too?

RDS: Yeah I'm not even at home enough to do anything

INTERVIEWER: Okay, and so for your cell phone when you can have it out or are on the bus, what sorts of things do you do on your cell phone?

RDS: Turn the music on and take a nap on the bus

INTERVIEWER: Do you ever text message and use data plan?

RDS: Yeah I text sometimes

INTERVIEWER: Not a big texter?

RDS: No

INTERVIEWER: Have you ever searched for pregnancy info on your phone?

RDS: No I have this app downloaded that gives me info called pregnancy tracker it updates me on things about pregnancy, how far along things to expect.

INTERVIEWER: how did you find out about that one?

RDS: My sister, she has a son and that was one of the things that she used so I just downloaded it.

INTERVIEWER: Has your sister ever shared anything else with you?

RDS: She doesn't really talk too much about anything about how horrible her labor was and how she doesn't want to experience it again

INTERVIEWER: Do you think that you guys have bonded at all now that you have both gone through a pregnancy?

RDS: No because she works practically as much as I do

INTERVIEWER: So you don't get to talk to her?

RDS: No

INTERVIEWER: Did she tell you about baby center?

RDS: No I don't know where I think she said it was from emails she sent me but I don't know. She didn't tell me about baby center

INTERVIEWER: other place you might have looked: blogs, Facebook or twitter?

RDS: No I don’t look on Facebook for baby stuff and I don’t have a twitter

INTERVIEWER: But you have a Facebook account?

RDS: Yes

INTERVIEWER: Are you excited to have your baby?

RDS: Yeah

INTERVIEWER: What’s most exciting?

RDS: Getting done with this pregnancy

INTERVIEWER: Can you think of anything else you’ve changed or want to try to change before your delivery?

RDS: Um nope, I don’t

INTERVIEWER: Are you happy with how things are going?

RDS: Yeah

INTERVIEWER: Two more questions, what do you hope to get from the texting study that you signed up for

RDS: Hopefully that I can be able to change the way that I eat, to change my perspective on the way I think about the foods that I eat, to eat healthier so the baby could be healthy and information I can give to someone else one day.

INTERVIEWER: Is that something you look forward to, sharing your experience with someone else one day?

RDS: Yeah

INTERVIEWER: That’s very nice of you. And how often do you think that you would need to receive texts for it to be effective?

RDS: Um probably once or twice a week when I’m not tired my memory is so good but I am tired and I cannot think straight today.

INTERVIEWER: Would you like to receive information that would help you rest throughout the day and help you be less tired?

RDS: Yeah

INTERVIEWER: And does your cell phone get charged for individual text messages?

RDS: No

INTERVIEWER: If you received messages everyday would that be okay.

RDS: That would be fine

INTERVIEWER: Would you be open to responding if we asked you a question?

RDS: Yeah

INTERVIEWER: Okay and is there anything else that you hope to get out of this study that we can provide to you?

RDS: Um no

Focus Group Interview with RDS029 and RDS031
Participants wanted to complete the interview together due to time constraints and clinic hours

(RDS029: deeper, more enunciated)
ASSUMPTION: 2=31 1=29

Interviewer: Okay, so we're going to start out by going over some really basic questions that describe your pregnancy. So the first is just asking you your age.

RDS029: 16

RDS031: 16

INTERVIEWER: Okay, both 16. Uhm, and then, how tall are you and how much do you weigh?

RDS031: Uhm, I'm 4'11" and now weigh 107 pounds.

RDS029: (laughs) you're so cute. Right now, how much we weigh? Okay 5'2" and I think it was 145.

INTERVIEWER: Okay, and then is this your first pregnancy?

RDS031: Yes

RDS029: Yes

INTERVIEWER: And would you describe your pregnancy as one that was planned?

RDS029: Mmm no.

RDS031: No.

INTERVIEWER: No? Okay. And so, I guess if not planned, how do you… what was it like to find out that you were pregnant?

RDS029: It was scary…

INTERVIEWER: Scary.

RDS031: Hurtful… I don't know. It was like… I mean… yeah, it was scary. It was scary. Cause I thought it was something else when they called me back. And they were like… I was pregnant. It was shocking.

INTERVIEWER: So given that now that you've decided to keep your babies, obviously you're planning for the future. So what goals and dreams do you have for your babies?

RDS029: Well first, to get them through the six weeks without you know, having any problems and stuff. And then hopefully I'm trying to… I don't know. (laughs)

RDS031: What goals do I have for the baby?

INTERVIEWER: Yeah. How do you envision life with a baby?
RDS031: I know it's going to be hard, but… I just want him to be healthy and as long as we're like a family and uhm… playing on his iPod over there. Yeah.

RDS029: Yeah, she said take it day by day. It's going to be hard.

INTERVIEWER: That's usually the best way to approach anything I think. One day at a time. Uhm… so it seems you both want to have healthy babies, happy babies… uhm. So what challenges have you faced during pregnancy as you planned to be a mother?

RDS031: All the appointments and like getting new insurance and everything… I feel like my mind is so busy trying to remember all these things I have to go to and call people and stuff. Not so much anymore, but in the beginning when everything was getting set up.

RDS029: And remember to take all your medications and stuff. And remembering to do that. So.

INTERVIEWER: How do you usually remember those things?

RDS029: When I open up my eyes, they right there. They right there by my alarm clock, so.

INTERVIEWER: That's a good idea. Do you use cellphones or anything like that?

RDS031: I have mine by my bedstand so I just remember.

INTERVIEWER: Uhm, and what do you think the important things are that you should be doing to make sure your baby's healthy?

RDS029: Eating healthy (laughs) That doesn't sound right, when she say what are some of the healthy… but…

RDS031: Taking your vitamins.

RDS029: Yeah

RDS031: And not doing drugs or alcohol.

RDS029: Attending all your appointments

INTERVIEWER: Uhm, so from the time that you started coming to the [location redacted] and seeing the midwives and nutritionists and social workers and the gang that's in the new building now, uhm can you kind of tell me about your overall experience working with all those people and…

RDS029: It's better than I thought it would be.
RDS031: Yeah, I didn't even know there were groups that prefer teenagers. I just asked one of my friends, she's like in her 20s but I asked her where she went, and she said like… it wasn't [place redacted]. But she said something, and I called the number and they immediately set me with the [place redacted]. So like, I don't know. I felt like… guided and comforted cause they knew what to do with me, and I didn't have to go through a lot of trouble. Yeah.

INTERVIEWER: Yeah, that sounds really helpful. So in terms of your experiences in the clinic, what sort of experiences have you had in relation to diet or nutrition? Any advice that they've given you…

RDS031: Yeah, they give us papers in there.

RDS029: Yeah, make sure you like eat something that has a lot of vitamins in it. And iron, too.

RDS031: It's like three servings of dairy, protein, and fruits and vegetables and five servings of fiber. I think (laughs) That's what it was.

INTERVIEWER: Uhm, so they give you paper handouts every time you're in group for that and uhm… what else do you talk about in group in relation to prenatal care?

RDS031: Everything (laughs)

INTERVIEWER: Everything? Uhm so mostly what I'm interested in is things that relate to weight gain uhm, diet and exercise. So uhm I know you probably learn a lot about the delivery process and preparing for that so uhm those aren't things we necessarily have to talk about in this

interview. But uhm so, it sounds like they give you quite a bit of nutrition information and advice so… is that advice you've actually been able to put into practice in your own lives?

RDS029: Yes.

INTERVIEWER: What things have you been?

RDS029: Even though like… even though I'm in prenatal care and everything, I mean like sometimes when you go to doctors and everything you still have problems that happen during pregnancy and everything, and they still give you stuff about like eating healthy and everything. You should automatically still be healthy… like try to be healthy even though you're not pregnant.

INTERVIEWER: Yeah, that's true. You were going to say something too.

RDS029: I was just going to say I tried to eat all the things they say you should eat, so… and like I stopped, I stopped doing things that they say you shouldn't like, a lot of coffee and stuff, I don't drink. I used to drink coffee like every day. Now I barely drink it.

INTERVIEWER: Are there any other things that they suggested you not eat?

RDS031: Certain kinds of fish and stuff

RDS029: Oh yeah.

RDS031: Yeah

RDS029: But I didn't eat that stuff anyways earlier. Like unpasteurized whatever, mil—cheese.

RDS031: Yeah, the types of cheese, too.

INTERVIEWER: Oh, okay. Do you remember what kinds of fish they said not to eat?

RDS029: Swordfish.

RDS031: Yeah

INTERVIEWER: Swordfish? Do you know why?

RDS029: Uhm…

RDS031: I mean, but they said it's okay to uhm, I think they said the—the the tuna… the tuna… I think it was unpacked or packed. I don't know. I can't remember. But they said one of them, we could eat it but not as much as like, kind of like once or twice a week.

INTERVIEWER: So, how has your appetite been or changed since before pregnancy?

RDS029: I eat more.

INTERVIEWER: All throughout the day, or are there certain times of the day that you're more hungry?

RDS029: Uhm… no, just all throughout I guess.

RDS031: For me, certain kinds of days. Like it would certain—uh kinds, uh types of times of day. Like it could be like night time and morning or it could be afternoon and night. If I don't feel like up to eating, like if I probably feel too lazy or something like that, I just have to drink more water or something until I'm ready to actually eat.

INTERVIEWER: So, how specifically has diet changed? Do you eat more meals, more snacks? More of certain food items?

RDS031: More broccoli and stuff. Broccoli and cheese and Hot Pockets (laughs)

RDS029: Well I eat… before I was pregnant, I ate like… I ate really healthy and not nearly as much and then now that I'm pregnant, I started eating more like a regular person because I was so hungry, I couldn't take time to find something that was extremely… I don't know how to—like in my… uh, what's it called? Like my idea of healthy, I was too lazy to find something that was like… that was like almonds or something, so I would just eat something else, if that makes sense.

INTERVIEWER: So looking more for whatever was convenient because you were hungry then and… it might not be easy to find the things that you would have liked to eat before?

RDS031: I get fuller now, too. Now that I'm like getting more—

RDS029: Yeah, and it hurts up here.

RDS031: You get fuller—it was like, dang. When I was like 2 months, I would just eat and eat and you never got full. But then now, now that you're further in pregnancy, you just get… you get smaller portions of stuff, but you still eat junk food and crave.

INTERVIEWER: So uhm, I want to talk about cravings but I just wanted to ask. Uhm, so I owe you one question really quick. You had said before you ate really healthy, I'm just curious. Like can you give examples of foods you ate before and foods that you eat now that you didn't normally eat?

RDS029: Uhm… I was just like really picky. Uhm and I would only eat like fruits and vegetables and really uhm… nothing that was packaged, like uh. Like now I would eat a Hot Pocket, but I wouldn't have before, and I wouldn't eat like Rice Krispies. I would only eat like bran flakes. Now I eat like healthy cereals still but… does that make sense? I was kind of like clean eating. I just ate really like bland and really healthy foods.

INTERVIEWER: Kind of more like an organic, natural approach.

RDS029: Now I just… I didn't have the time. Now I would just be so hungry in the beginning of my pregnancy, I would just—I don't care, just give me food. So now I eat more like a regular foods I guess. And I wouldn't eat the things my mom made for dinner because they weren't like healthy enough. I would just eat my own dinner, but now I eat whatever she makes, except I don't eat cow or pig. Or I try not to, but sometimes I'm like ah that looks really good, and I go for it.

RDS031: Yeah.

INTERVIEWER: (laughs) But do you both eat chicken, then?

RDS029: Yeah.

INTERVIEWER: Okay. So cravings. Are there things that you crave more or less now that you're pregnant?

RDS031: Less chocolate, more fast food. More rice. I don't know. Less stuff that has meat in it too, so.

INTERVIEWER: Do you crave things with meat in it less?

RDS031: Mhm. If I get fast food, I would get like uh a Caesar salad and I would be like, no little strips of meat or anything. But I just like fast food.

RDS029: I haven't really noticed any cravings, but like I said, my whole eating has like changed altogether. So like, I want things now that I wouldn't have months like… last year. But that's cause I'm in the habit of eating them now I think.

RDS031: (laughs, mumbles)

RDS029: Last… six months ago. No… I was pregnant six months ago. Eight months ago.

INTERVIEWER: (laughs) So would you say that it's more that you're hungry then, it's the convenience of having those foods? Or that you actually do crave them?

RDS029: I think it's just that I eat them… like. Before I was pregnant, I like… I… let's say McDonald's. I never had McDonald's, so I would never want it. Cause I went with eating what I ate, but now that I eat McDonald's, sometimes I'll be like oh, I want McDonald's.

INTERVIEWER: Okay.

RDS029: So it's just like, I don't know.

INTERVIEWER: So it's this… your pregnancy has opened up the opportunities to have tried new foods that you've found that you've like…

RDS029: Sure (laughs)

RDS031: Yeah that sounds about right.

INTERVIEWER: Just making sure I understand. So have you ever had any aversions to food? Like where you can't stand the smell of it, or the taste of it?

RDS029: Uhm I used to love vanilla almond milk, and then in like my first trimester it made me really sick any time I smelled it, and I never drank it ever since then. I just haven't been in the mood.

RDS031: Nothing really grosses me out, so.

INTERVIEWER: But you said you eat a little bit less chocolate and… a little bit less meat.

RDS031: Yeah, I don't go around it. That's why (laughs). I don't want to go around it cause I know that… I know that I would try to eat it, but I need to eat more food instead of chocolate because if I eat chocolate, that's not going to fill me up or anything. I'm just going to keep eating it and… it just I don't know. I would be looking at myself like, look at you. You're so fat. You're just eating chocolate—that's not healthy. You just need to eat a full course meal.

INTERVIEWER: Uhm, so in terms of the quantity of food, how has the amount that you've eaten either throughout the day or in one setting changed? You both mentioned now that your bellies have gotten bigger, it's harder to eat as much before you get full. But do you think overall you eat more now than you did before?

RDS029: I think I just eat more often.

RDS031: Yeah.

RDS029: Now I eat like, double of my old dinner. Just more snacks kind of.

INTERVIEWER: And so, what's a typical day like for each of you in terms of how often and when you eat?

RDS029: Breakfast at 6. No, 7. And sometimes a snack before lunch, depending.

RDS031: My breakfast turns into a breakfast turned into lunch. Cause I'll be taking Benadryl and junk, and I'll be knocked out. I (won't be?) waking up til 11 o'clock, so. My breakfast turns into lunch.

RDS029: Do you go to school?

RDS031: Hm?

RDS029: Do you go to school?

RDS031: No, I have a tutor.

RDS029: How?

RDS031: If you transfer to Young Mother's or uh if your school provide you…

RDS029: Honeyway. It's like this big, so… we get nothing offered to us. (laughs)

RDS031: Oh snap. I got to have a tutor. I can't go to school. It's too much.

RDS029: You're lucky.

RDS031: I know.

INTERVIEWER: So what about after lunch? When you've eaten your brunch or lunch, what's the rest of the day like for eating?

RDS031: I'll be too busy, so I'll like an apple or something, and then I'll eat like probably like 8 o'clock, cause if I eat like really really late, or if I eat like oily foods late, I'll start to have nightmares and stuff. I try not too eat too late.

RDS029: Uhm I eat after I get home from school at like 3. And then dinner.

INTERVIEWER: Any snacks before bed?

RDS029: Yeah, if I'm hungry. It just depends on how big my dinner was and stuff.

INTERVIEWER: So how do you think the things that you eat affect your baby?

RDS029: They get the nutrients.

RDS031: I agree (laughs) I was going to say that, yeah. And all the vitamins.

RDS029: But it won't make him fat if I'm eating a lot of ice cream and anything. And if I did, it wouldn't be really bad because babies are supposed to be fat (laughs) and they have to gain weight. Like gaining weight is healthy for them anyways.

INTERVIEWER: So in… gaining weight, if you're gaining weight, does all of that transfer over to the baby?

RDS031: Uhm, I mean they do say that… wait hold on. See, it just popped out of my head.

RDS029: I don’t think if you’re fat, your baby will be fat. But they’ll have the same like if you eat too many cookies and not enough broccoli, then they’ll be lacking the nutrients—

RDS031: --in the broccoli—

RDS029: But they won’t necessarily be like obese. (laughs)

INTERVIEWER: So do you think there’s anything really bad that you can do in terms of your diet for your baby?

RDS029: Not eating the healthy foods and… just the same thing—and just eating healthy. Not eating a lot of junk foods.

RDS031: Like just soda, all day. Just sitting down, eating. Not moving. (laughs)

INTERVIEWER: Is there anything that a pregnant woman should be careful about eating? Like you mentioned fish. Anything else that you can think of?

RDS031: (laughs)

RDS029: The unpasteurized… or things that have uhm… that certain bacteria, and then alcohol.

RDS031: Uncooked meat. Like make sure the meat is cooked. You know like hamburger meat, or... chicken and all that stuff too. Like you have to cook it and make sure that it’s on like… (laughs) just trying to picture it now.

INTERVIEWER: Uhm so is there anything that both of you are specifically avoiding or adding to your diets to try to improve the health of your baby?

RDS029: I make sure that I pack a fruit every day for lunch. Just to make sure, and I drink a lot more milk and… yeah.

RDS031: I try to keep soda out.

INTERVIEWER: Anything that you’ve added?

RDS031: No, I’ve always been drinking Polar Spring water so, I’ve taken a lot of stuff out. Cause I… oh yeah I did add the broccoli and cheese steamers thing… What do you call those?

INTERVIEWER: Oh, the steamer bags?

RDS031: Yeah, the steamer bags.

INTERVIEWER: Those are good. Uh… (pause) who does the shopping for both of you for the food that you eat?

RDS031: My… mostly my baby's father; my boyfriend.

RDS029: My mom does.

INTERVIEWER: So do you both get input into any like groceries if there's something you want?

RDS029: Yeah.

RDS031: No. I mean like if I say like I got to go grocery shopping, he won't say like anything, but he'll like go for me and bring it to my house. And I'll be like what is this? Like if you like… for real though, if you have like a house full of food but then you look at it and be like, this is nothing that I eat on a regular day or daily basis. This is like (laughs) they just make you mad, because that's the only food that's there so you just have to try—you just have to try—you just have to mix stuff together and everything, so. That's why I start going off on my own, but… my mom do the same thing, so. Just buy stuff she think I eat—the chocolate chip waffles. Like I don't want to eat that! Hot Pockets. And French fries and a lot of other stuff. I don't want to eat that. Like just because I'm pregnant I crave for a lot of stuff… no. I just want to eat something that'll fill me up, you know? I have to eat like… six Hot Pockets to get full, so.

INTERVIEWER: (laughs) Uhm so for nutrition information, where would you go if you had questions about something?

RDS029: Online. I look everything up online.

RDS031: Me too.

RDS029: All day I'm just looking things up.

INTERVIEWER: Are there any specific sites that you found helpful?

RDS031: BabyCenter.

RDS029: I just go on Google and whenever—go through all the results. Read through all the—

INTERVIEWER: One by one? So you just look through everything that pops up?

RDS029: Pretty much.

INTERVIEWER: Have there been any websites where you made a mental note that might be good to go back to for other things?

RDS029: Uhm well yeah, Baby Center. Whenever I look something up on my pregnancy or the baby, that's always one of the top results. It's pretty good.

INTERVIEWER: And any people that you've asked for advice, or… uhm can talk to about their experiences in during pregnancy and have given good advice?

RDS029: My mom.

RDS031: My sister. Bad, negative things.

INTERVIEWER: She gives bad advice?

RDS031: But it be true. But it be negative to me, like… I don't want to hear it. You know people be telling you stuff like… oh it's gonna hurt. It's really gonna hurt. You have to push and push and towel after and stuff like that. You don't have to say it like that, but… other than that, that's the only person that really tell—I mean my boyfriend really telling me stuff and everything, like you never had a baby, but he do be telling me things that I didn't really know, so that's good for him to be looking deep into…

INTERVIEWER: So he's been reading?

RDS031: Mhm

INTERVIEWER: That's good. So anyone else you can think of that you've gone to to ask advice from… or

RDS029: Our midwives, and nurses and stuff.

INTERVIEWER: And have you both met with the nutritionist uhm at [location redacted] too? I think her name is [name redacted]?

RDS029: Yeah.

INTERVIEWER: No?

RDS031: I probably did, but I don't remember the name.

RDS029: Is she blonde?

INTERVIEWER: Mhm.

RDS029: Yeah.

INTERVIEWER: Yeah, longer hair? Was that helpful? Like meeting with her? Or was it so fast, that—

RDS029: I don't remember what we did.

INTERVIEWER: Okay

RDS029: I don’t know

RDS031: It’s really hard to remember things.

INTERVIEWER: Oh, I know.

RDS029: I think it was way back, like in my second month.

INTERVIEWER: The very beginning?

RDS029: Where she just said what I should I don’t know. Drink a lot of water and stuff.

INTERVIEWER: Uhm, so changing… oh no. Some more nutrition questions. So we’ve talked about things that you’ve changed, but just to kind of probe for specific items, so how have you changed your consumption of fried foods?

RDS029: Definitely increased (laughter)

RDS031: um, decreased for me

INTERVIEWER: Decreased? What about sugary or salty foods?

RDS031:Can't eat those period, I swell up like a pumpkin

RDS029: I like salty a lot more now, but I don't really like sweet things, they always make me sick(laughter)

INTERVIEWER:Just like everything over there

RDS029:and sometimes I'll be like eh, looks good, but I always regret it afterwards becuase I always get sick.

INTERVIEWER:is that even before pregnancy too?

RDS029:Yea, I've just, yep

INTERVIEWER:Um, and then kassina you had said that you were trying to drink more milk now, how about more dairy foods? like yogurt, cheese ice cream

RDS031:yogurt, like the oreo things

INTERVIEWER:the one with the oreo crumbles on top?

RDS031:mhm, low fat yogurt, I mean, at least it says that right

RDS029:Uh, I don't eat yogurt but, I mean I eat cheese, I drink a lot of milk, and I have cereal almost every day so

INTERVIEWER:Still bran flakes still?

RDS029:yea(laughter)

INTERVIEWER:no those are good for you

RDS029:and shredded wheat and rice krispies and cheerios

INTERVIEWER:what brand of brand cereals? is it like total or wheaties?

RDS029:um

INTERVIEWER:not sure?

RDS029:um, I don't know

INTERVIEWER:Its ok, I was just curious

RDS031:Theres honey bunches

INTERVIEWER:Honey bunches of oats? those are good too. um and, I can't remember if I asked this already but have you been craving any non-food items, like to chew on ice or eat baking soda, anything like that

RDS031: clay

INTERVIEWER: Eating clay or

RDS031:no, I don't eat it (laughter)

INTERVIEWER:ok, just asking

RDS029:You want to eat it?

RDS031:yea, I don't know, I look at it and it just be

INTERVIEWER:Why do you think you have this craving?

RDS031:Um, maybe because I be picturing something like different over it,like it can actually be clay, but

RDS029:like picture it as a hot dog?

RDS031:Yesterday, I don't know, I like put my nose up against clay and stuff

INTERVIEWER:Have you ever

RDS029:Where do you see clay?

RDS031:huh?

RDS029:Where is clay?

RDS031:uh, I be going to like this um, little pottery thing all the way out in [location redacted]. Its like the um, I forgot, no, the plaza, its in a plaza where we like, make cups and stuff and everything and I be going there on fridays or thursdays sometimes

INTERVIEWER:and you think about eating the clay

RDS031:like when I get home, I be like, um, clay was the verdict today

INTERVIEWER:So, have you ever heard of any like nutritional things thats associated with it at all?

RDS031:no

INTERVIEWER:no?

RDS031:no

RDS029:Iron deficiency, I don't know, that was a guess

RDS031:I mean, no, no, I'm not going to lie to you

INTERVIEWER: Have you ever talked to the mid wives about your iron status or anything like that?

RDS031:Yea, it was very low, so I had to take iron supplements

INTERVIEWER:ok

RDS029:Wait, so was I right?

INTERVIEWER: yea, you were right, it’s associated

RDS031:ok, so I'm about to eat clay then (laughter)

INTERVIEWER:Well, its just sometimes that craving can happen when you have low iron status

RDS031:oh, ok

INTERVIEWER:yea, its a normal thing to happen, sometimes people chew on ice a lot too

RDS029: My mom, used to be literally addicted to ice, and she would eat it all the time, like constantly and it was a problem

RDS031:Yea, my grandmother used to have cups in her car with ice, like regular ice, like crumbled ice, and all they would do is chew on it all day every day, like they would just come to my mom house cause my mom had the refrigerator with the, you know, the refrigerator that make the

RDS029:yea

RDS031:they come there every day and be like, can I borrow some ice?

INTERVIEWER:um, so supplements then are you both taking supplements?

RDS029:like prenatal?

INTERVIEWER:prenatals yea, and vitamin D cause you're in the Vitamin D study. Are you pretty good at remembering to take them, or is it hard to remember to take them

RDS031:They right by my other medication so

RDS029:I remember usually

INTERVIEWER: and then you mentioned you have an iron pill?

RDS031:mhm

INTERVIEWER:just take that at the same time?

RDS031:yea, and my vitamins, my chewy vitamins

INTERVIEWER:Do you feel any differently taking them?

RDS031:mhm, like if I, like say if I missed them one day, or didn't take 2 for 2 days, like I don't know, like I feel different when I don't take them, for some reason. I don't know why

INTERVIEWER: so what effects do you think your supplements have on your baby?

RDS029: Good effects

INTERVIEWER:Are there any nutrients in the supplements that you think are most important for your baby?

RDS029:Iron, no that’s for the blood, well maybe, cause they need blood. Like blood, because we have more blood circulating

RDS031:And its because of the red, no not, I'm about to say the red blood cells, you know how about the red and the blue and the white

RDS029:Red and white?

RDS031:no, red and blue

INTERVIEWER:So you're thinking veins and arteries

RDS031:Man in the um, the umbilical cord, never mind, cause I can't explain it any better

INTERVIEWER:Any other nutrients that you think are more important in your prenatals?

RDS029: I never read the nutrition facts, but uh, vitamins and minerals, not minerals, wait minerals? (laughter)

INTERVIEWER:And since you've become pregnant, have you tried to eat any fortified foods, like cereals have, like all the extra added vitamins and minerals and other foods, do you every seek out foods that have the extra things in them

RDS031: mhm, cause that’s all my boyfriend has

RDS029:The orange juice with the calcium

INTERVIEWER:Yea, something like that, that's fortified. Do you drink that?

RDS031:You're really driving him crazy, look at him

INTERVIEWER: he's like can we go now, this is boring

RDS029:hang in there buddy

INTERVIEWER: we're more than halfway done

RDS031:Wait how long it lasted?

INTERVIEWER: about an hour

RDS031:ok, that means its probably 40 minutes until the end right?

INTERVIEWER:36. we might get done early. ok so

Guy:There's a couch over there

RDS031:You can go there if you want, you don't have to be here

Guy:Nah, I'm just kidding

INTERVIEWER:Ok, so next question. Has anyone ever offered you advice about recommendations for eating, things that a pregnant women should do or not do. Anyone tell you interesting stories about when I was pregnant, blah blah blah

RDS029:Just craving stories of what they craved

INTERVIEWER:Ok, so how do you think that all the things that you eat in terms of, well no, scratch that, ok um, what do you think about, or how do you feel about the weight gaining experienced during your pregnancy

RDS031:That I actually did a good job because I didn't gain a lot of pounds or anything and I'm not underweight anyways, its all baby

RDS029:um, I guess I'm in the normal range, so that's good, I don't want to gain a lot of weight but I don't really care because its not about me, its about the baby, so as long as he's healthy I don't care if I'm fat

INTERVIEWER:So what do you think, so you said you're in the normal range, what range are you referring to?

RDS029:Like you're supposed to gain 25-35 pounds

INTERVIEWER:And it was the same recommendation for you too [name redacted]?

RDS031:mhm

INTERVIEWER:ok

RDS031:theres also those little things that they gave us too. The vitamin D thingy, no, was it in the Vitamin D thing? it had to be. Like in the little things, got the banana, and uh, you don't remember?

RDS029:banana?

RDS031:The Vitamin D study thingy, which she gave which had a crossword puzzle at the bottom

RDS029:oh the paper that they

RDS031:yea that said that you should gain 25-30 pounds

RDS029:this thing?

RDS031:is it that one? or is it the other one?

RDS029:it’s not on this one,

INTERVIEWER:um, so is that the amount that all pregnant women should gain, or are there other recommendations as well

RDS031:They might tell you to gain weight if you started underweight, they might tell you to eat more, or if you're overweight, they'll tell you to eat less, like gain less

RDS029:it depends, like overweight people sometimes don't gain any weight at all, but underweight people should gain more. Normal people just, in the normal range, gain 25-30 pounds

INTERVIEWER:Do you know why that is, like what happens if you don't gain enough or gain too much?

RDS031:It’s not healthy

RDS029:Well gaining too much is bad because only so much is baby, and then the rest would be, well sometimes its water weight and stuff, but then it’s like fat and then you get diabetes and those veins

RDS031:Varicose veins?

RDS029:yea, and other bad symptoms from fatness

INTERVIEWER:so what about for the baby? do you think excess weight affects the baby at all? or it affects more the mom

RDS031:I don't know

INTERVIEWER:not sure?

RDS029:yea

INTERVIEWER:That's fine, you don't have to know. um

RDS031:I mean, they could be telling you for your own health, but then they could be telling it for the baby's health but they never say it so

RDS029:Well if you're like gaining too much weight, and not exercising, then you're not going to be in good condition to give birth and make birth harder, and if you're not eating a lot, then the baby won't be getting the nutrients if you're under eating

INTERVIEWER: Yea, that's true. Um, so you mentioned exercise, what do you mean not exercising enough?

RDS029:Like just laying around, being lazy cause you're pregnant.

INTERVIEWER:So, what have you heard are good recommendations for good exercises for women who are pregnant?

RDS029:Swimming, walking, and pregnant yoga

INTERVIEWER: Anything that you should more or less of?

RDS031:picking up like heavy

INTERVIEWER:weights?

RDS031:yea,like groceries, your baby, you can pick those up, not overweight stuff that you have to struggle to bend over , and they tell you to um, bend, did they say like? to squat down with your knees, no bend down with your knees, not your back?

RDS029:yea, it sounds like it, I can't bend that way anymore either

RDS031:yea me neither that's what I said

RDS029:you gotta like squat, wait what was the question? cause I had an answer

INTERVIEWER:so, types of exercise that you should do more of or less of

RDS029:Oh yea, don't push yourself too hard, it’s not that good to lay on your back, so crunches are not good (laughter)

RDS031:oh I'm sorry

RDS029:and um jumping up and down on a trampoline, and kegels, were another exercise

INTERVIEWER:are good or bad?

RDS029:good

INTERVIEWER:good, um so how long should pregnant women exercise each week?

RDS029: Like short, um increments so that, cause your heart can't be, like you can't get too worked up, you have to make sure you rest enough. So just for maybe half-an-hour a day or take breaks in between if you want to do it longer.

RDS031:uh yea, usually like I exercise every day, but not for that long. about 30 minutes

RDS029:and low intensity
RDS031: yea

INTERVIEWER: How has each of your energy levels or physical activity amounts changed since before pregnancy?

RDS029: Lowered. Like I go to bed really early now. Sometimes I need naps.
RDS031: I take naps. I mean, I could be asleep and then my boyfriend will call my name, and I won’t hear him and then I’ll wake up and then like 3 seconds later, I’ll could just be asleep again. I’ll be like taking naps back to back to back. And then like my energy is like wheeeee!
INTERVIEWER: When you’re up?
RDS031: When I wake up by night time.
INTERVIEWER: So, how much sleep are you getting now do you think?
RDS029: I’m sleeping from like 9 to 6. So, like 9 hours.
RDS031: I’m sleeping from like 11 or 12 to like 11 or in the afternoon.
INTERVIEWER: So, twelve hours? Do you think there’s a good amount of sleep that is recommended?
RDS031: At least 6, 7 or 6, 7 hours of, right?
INTERVIEWER: I (laughing)
RDS031: Well, they should tell you that, they should tell like any way. Like if you gotta get up in the morning, you should at least have 7 hours of sleep.
INTERVIEWER: Thoughts?
RDS029: I would say at least 8 hours. Especially if you are pregnant, probably more like 9 or 10 cause you need extra energy to get you through the day.
INTERVIEWER: Is there anything that found that keeps your energy up at all?
RDS029: Snacks? If I don’t have my snacks, I get really, really cranky and tired and headaches and stuff, so.
INTERVIEWER: Me too. Um, has anyone given you exercise or physical activity advice?
RDS029: Hm, like walk and swim.
INTERVIEWER: Any advice from parents or friends?
RDS031: No, they don’t like giving me advice.
INTERVIEWER: How come?
RDS031: Um, I don’t know. They just not helpful. They just care about themselves.
RDS029: I went swimming the other day and um, in the school pool and my dad was like, see, this is good for you. That’s like all. And one time he was like, you should go for a walk, it’s good for you.
Man: Tell her about what I bought you.
RDS029: Oh yea. [name redacted] bought me this bouncy ball thing, and I bounce on that. It’s pretty cool.
INTERVIEWER: So, if you had to pick your favorite exercise what would it be?

RDS031: Walking.
RDS029: I like swimming.
INTERVIEWER: I am not a swimmer. I'm a sinker.
RDS031: You can try walking around in the water.
INTERVIEWER: That's not good for me.
RDS031: (laughing)
RDS029: Well, I get really bad cramps when I walk. So, that's like, I would want to walk, but I can't.
INTERVIEWER: So, have you changed your physical activities at all after you became pregnant? I remember you saying your energy levels went down after you became pregnant compared to before, but how about the activities you've done? What's changed?
RDS029: I used to play volleyball and cheerlead. And I worked out.
RDS031: I used to run track. I stopped. I didn't do no basketball or stuff like that. Oh, and I'll go bowling too—but, now I can't do that. The ball is too heavy.
INTERVIEWER: And, so, not too many more questions left. So, what do you think physical activity does for your baby?
RDS029: Hm, it makes its heart healthy. I don't know.
RDS031: I heard that like, physical activity… I heard that when you walk a lot or jump like your muscles when you lift a lot, delivery is.
INTERVIEWER: So, are you saying that when you work out a lot, you will have an easier delivery?
RDS031: Hm-hm.
INTERVIEWER: Okay.
RDS029: Probably just makes the baby happy, because like when you work out it makes you happier. And when you are happy, the baby is happy.
INTERVIEWER: Um, and then, you both volunteered to be in the text study and I guess I'm just curious as to what you expect to get out of this study from us.
RDS031: I expect to get like a lot of information on health. Cause sometimes I can't find like good information on the internet that has to do with gaining weight and diet and everything to do with nutrition, and everything that has to do with your heart sometimes to find stuff like that on the internet. I know that some stuff can be helpful, but.
INTERVIEWER: So, when you are looking for things, are you guys mostly looking on your computer or are you looking on your cellphones.
RDS031: Cellphone.
INTERVIEWER: Cellphone? How about you (1)? Anything you were kind of expecting or hoping to get out of this study?
RDS029: Um, just helpful tips and to eat healthy and um, I think you said you would send us messages.
INTERVIEWER: Yes, yes we will. And, so, in order to be effective or motivational to you and to kind of think more broadly for other girls that are your age and having babies, what do you is the best frequency of receiving a message? What do you think is important and how often do you think we should be sending messages for it to be effective? If that made no sense, let me know, and I'll try to reword it.
RDS031: 1 or 2.
INTERVIEWER: One or two days a week? Or 1 or 2 a day?
RDS029: I'd say one or two a day would be kind of annoying. I'd say once week maybe.

INTERVIEWER: Once a week?
RDS029: Well, I'd say every other day.
INTERVIEWER: Um, well it's just that, I'm just asking.
RDS031: Yea, every other day.
INTERVIEWER: Would it depend on the tone of the messages?
RDS029: Well, if it um, like if it was helpful, I would like getting them. Like in the Vitamin D study, I would get texts about my baby. And sometimes, I would forget what week I'm in, so it reminds. So, if I mean, they are like good and helpful, yes.
INTERVIEWER: Anything else you can think of? If you got a message everyday would that be bad?
RDS029: Hm, I mean for now that's the best, I mean it would not annoy me because I like getting information about everything that I gotta do. I just like, I don't know. Yea, I would like that information. But, everybody is different so.
INTERVIEWER: That's true. Okay.

RDS030

INTERVIEWER: Alright, I'm going to start by asking you questions to describe your pregnancy and those will be helpful for us later on when we look back at how you responded so we can match them up with the stage of pregnancy that you're in. So the first question is how old are you?

RDS030: 17

INTERVIEWER: Okay, and how tall are you?

RDS030: 5'2"

INTERVIEWER: And is this your first pregnancy?

RDS030: Yeah

INTERVIEWER: And you said you're 35 weeks…

RDS030: 31

INTERVIEWER: Sorry 31 weeks, my bad. Um, and was this pregnancy planned, or…?

RDS030: No

INTERVIEWER: No, okay. Uh, so were you using contraception at the time?

RDS030: No

INTERVIEWER: Okay. And what hopes and goals do you have for your baby to be?

RDS030: What do you mean?

INTERVIEWER: Um, like, do you think about, like, what it'll be like in the future for your baby, and, um, what things do you hope that she'll have or achieve, or he sorry. Um…

RDS030: It's a girl

INTERVIEWER: Oh, okay. So anything that you think about that you want your baby to have in the future?

RDS030: Not really.

INTERVIEWER: Okay. Um.

RDS030: I don't really think about the future.

INTERVIEWER: Okay.
RDS030: I like to live in the present.

INTERVIEWER: That’s good. Um, so in terms of the present are there any challenges that you faced so far in your pregnancy or even like right now in this moment, um, that make it, that you think will make it difficult for you to be a, a mother or um, carry a baby now?

RDS030: Well, like, I don’t talk to the baby dad so…

INTERVIEWER: Okay. Do you ever, um, think about anything, like health related as a challenge, like nutrition, finding food…?

RDS030: No

INTERVIEWER: Okay. And what things do you think are important that you should be doing for your health and the health of your baby?

RDS030: Eating more fruits and vegetables

INTERVIEWER: Okay. Anything else?

RDS030: Exercise more

INTERVIEWER: Um, where do you typically look for, or have you ever looked for information related to diet or exercise or anything about pregnancy?

RDS030: No, I’ve never looked at information about that.

INTERVIEWER: Okay, um, have you ever searched the Internet for anything even if it’s like looking up, uh, clinics to visit, like the [name redacted] clinic?

RDS030: No, my friend was the one that told me about this.

INTERVIEWER: Okay. Well, that’s a good friend! Um, do you ever get on the Internet for other things?

RDS030: Yeah

INTERVIEWER: What kind of things?

RDS030: Like, um, if I have homework, you know, I Google some answers

INTERVIEWER: Yeah

RDS030: Um, to get on Facebook and check my email.

INTERVIEWER: Okay, and can I just ask since you do have Facebook account, is there a reason that you wanted to do the text messaging versus the, the Facebook group?

RDS030: No, it's just because I don't be on Facebook all the time so if you were to send me a message I probably wouldn't see it for like 2 weeks after that. And like, the text message, you know, my mom would be like, oh this came for you.

INTERVIEWER: Okay, awesome. Uh and, where do you typically access the Internet when you get on the Internet?

RDS030: At school or on my moms cell phone.

INTERVIEWER: Okay, so you don't have a computer at home. You just use the data plan on the cell phone. And you said typically you look for school related, homework answers…

RDS030: Yeah

INTERVIEWER: …Facebook. So it's kind of a social thing, but also for homework.

RDS030: Mm-hm.

INTERVIEWER: Okay. Um, do you think that if someone told you about websites that were helpful for prenatal care that you might visit those sites?

RDS030: Oh, um, I have an, a BabyCenter account I think its what its called.

INTERVIEWER: Okay.

RDS030: I forgot about that. I haven't really been on there.

INTERVIEWER: When you were getting on there was there anything that you saw that you thought was interesting or that you enjoyed reading about?

RDS030: Yeah, it would like, um, tell you how many weeks you were…

INTERVIEWER: Mm-hm.

RDS030: …and then it would show you a picture of the baby.

INTERVIEWER: Oh, cool.

RDS030: Not the baby, but like it'll say like it says big as a pea or you know stuff like that.

INTERVIEWER: Yeah

RDS030: And it showed, like, videos of people having their babies or C-sections. Yeah, it was cool.

INTERVIEWER: Anything else that you can remember that you really liked from that site?

RDS030: No. Cause then they just have a lot of ads so…

INTERVIEWER: Oh

RDS030: …that’s like, yeah.

INTERVIEWER: Overwhelming. How did you hear about BabyCenter?

RDS030: Um, when I used to, when I first found out I was pregnant I was going to, um, general at the women’s center and the, um, social worker told me about it.

INTERVIEWER: And did you sign up on your moms cell phone?

RDS030: Mm-hm.

INTERVIEWER: Okay, awesome. So, how-, over the course of time since you registered for that, how many times did you think that you’ve been on that site to look at information?

RDS030: I think I see about 100 times.

INTERVIEWER: Okay.

RDS030: Cause I used to go on a lot, but then I just forgot about it so

INTERVIEWER: Um, do you think that receiving text messages from us might be a good reminder and like actually motivate you to go and look at that site?

RDS030: Yeah

INTERVIEWER: Okay. Um, and so you have access to a cell phone, um, do you own your own phone or plan to own your own phone in the future?

RDS030: I don’t own a phone now, but I would like to in the future.

INTERVIEWER: Okay, um, and we had kind of already talked about how you use it so text messaging, searching the web, uh talking, do you ever watch YouTube videos, or anything like that?

RDS030: Yeah

INTERVIEWER: Okay, um, and in terms of health information, baby center is the only way you've really been looking for health information for pregnancy.

RDS030: Yeah

INTERVIEWER: Not any other sites that you can think of?

RDS030: No.

INTERVIEWER: Okay, and have you ever been on twitter or blogs or anything like that?

RDS030: Yeah, but I don't like twitter.

INTERVIEWER: Okay, um, but not specifically for prenatal related stuff, just for fun.

RDS030: No, yeah.

INTERVIEWER: Okay. Um, so based on your experiences so far is there a place or person that's provided you with the best information about pregnancy?

RDS030: The midwife's area.

INTERVIEWER: Okay, what about family members?

RDS030: No

INTERVIEWER: No? What kind of information do you think is most helpful from the midwives?

RDS030: Like when they give me like sheets, and you know, you like read them and stuff. There's some helpful things in there.

INTERVIEWER: Okay. Do you save the sheets? Or do you just read them…

RDS030: Yeah

INTERVIEWER: …and then toss them?

RDS030: They're, they're all in my room.

INTERVIEWER: Okay.

RDS030: I'm a hoarder.

(Both laugh)

INTERVIEWER: I think back to my teen years, I think I might've been too! It gets better with time I think. Um, so was there anything on those sheets that were…do you have favorites?

RDS030: No I don't think so.

INTERVIEWER: Okay, um, so now I'm going to kind of change gears, so we asked a lot of questions on electronic media and internet, um, so the next set of questions are about weight gain, and the first question is have you ever been given advice about weight gain during pregnancy?

RDS030: I don't know what that is.

INTERVIEWER: So, um, how, as you go through pregnancy, you obviously, your belly gets bigger, and so sometimes you gain weight, so um, so you might have started out at like 120 pounds and now you may be like 130, 140 pounds, um did anyone ever talk to you about a certain amount of weight that you should or shouldn't gain?

RDS030: No

INTERVIEWER: No? Um, so when you visit the clinic, they weigh you every time that you come in?

RDS030: Yeah

INTERVIEWER: Okay. And, um, do they talk to you about eating more or less at all?

RDS030: No

INTERVIEWER: Okay, um, so there are actually weight gain guidelines from what's called the institute of medicine. And so it's based on your, your body mass index, which you calculate from your height and weight and I don't know if they have ever told you from your medical chart what your body mass index is.

RDS030: No

INTERVIEWER: Um, but, so if your generally like at a healthy weight prior to pregnancy vs. overweight or underweight, it can change, um, how much they recommend that you gain so let's say that you were a healthy weight before pregnancy, then you would be in this category and they would recommend that you gain 25 to 35 pounds. Um, and then you actually gain less, so if you were overweight prior to pregnancy then you're gaining like 15 to 25 or 11 to 20 and then the most is if you were actually underweight. Um, but just kind of look at these ranges, and see if, like tell me what that makes you think about? Does it seem like a lot or a little?

RDS030: For these ones yeah, they seem like a lot and they seem like less.

INTERVIEWER: So do you think that, does, does this concern you at all or make you, like in comparison to yourself?

RDS030: No

INTERVIEWER: No? If—

RDS030: The nurse at my school told me that, um, um, my weight range is good for, um, how far along I am.

INTERVIEWER: That’s good, di-did she say how much weight at all or just said it’s good?

RDS030: Yeah, she just said it was good

INTERVIEWER: Okay. Perfect. So do you think about your own weight gain at all like over the course of pregnancy as you’ve gotten a bigger baby bump?

RDS030: No, I’m just scared that I’m not going to be able to lose it.

INTERVIEWER: What makes you scared about that?

RDS030: Cause I know some people that were pregnant, and now they’re like f-, like fat and they say that it’s hard for them to lose weight.

INTERVIEWER: Have you heard of anything that can help you lose baby weight faster?

RDS030: Breast feeding, but I don’t, I’m not doing that.

INTERVIEWER: Okay. How come?

RDS030: That to me feels uncomfortable.

INTERVIEWER: Okay. That’s okay. Um. And so, on your baby center account or in the papers that the midwives have ever given you, ever said anything about how many pounds to gain or not gain during pregnancy?

RDS030: No, I never seen anything like that.

INTERVIEWER: Okay. And overall how comfortable do you feel about the weight that you’ve gained during pregnancy?

RDS030: I’ve been like, I’m fat now. (Laughs)

INTERVIEWER: Aw. Don’t say that! Do you feel positive that you can lose your baby weight after?

RDS030: No

INTERVIEWER: Is there anything that, um, if you received more things from baby center or um, messages from us that, d-is there anything that you think might be helpful and motivating or teaching you how to lose that weight?

RDS030: Depends on what the motivation is.

INTERVIEWER: Mm-hm. Um, okay, so let's talk about a little bit about eating. Who typically prepares the meals that you eat?

RDS030: My mom.

INTERVIEWER: Okay. And who goes shopping for the, those meals?

RDS030: My mom.

INTERVIEWER: Okay. Do you ever get to have a say in what groceries that she buys?

RDS030: Well, she asks me what do I want. So.

INTERVIEWER: Are there any things that you usually always ask for?

RDS030: Orange juice. You know how at, at Samson's, they have the big 24 packs.

INTERVIEWER: Mm-hm. Uh, how much money do you think that your mom spends each week on groceries?

RDS030: Well, she, like she gets food stamps, so you get'em like once a month.

INTERVIEWER: Mm-hm.

RDS030: S-um, I think she gets about 400. And when she goes shopping, she buys everything that same day.

INTERVIEWER: So maybe she just shops like maybe once a month.

RDS030: Mm-hm.

INTERVIEWER: Okay. And can you tell me about how you've been eating over pregnancy?

RDS030: I think I've been eating good.

INTERVIEWER: Okay. Have you made any changes at all from when you found out that you became pregnant?

RDS030: No.

INTERVIEWER: Do you think you're eating more or less?

RDS030: More.

INTERVIEWER: More? A lot more a little bit more?

RDS030: A little bit more.

INTERVIEWER: And are you eating because you feel hungry or because you have to eat for two?

RDS030: Because I feel hungry. (Laughs)

INTERVIEWER: Okay. Um. In terms of the amount, how uh- do you eat more meals or do you just eat maybe more snacks?

RDS030: More meals.

INTERVIEWER: More meals? How many meals a day do you think you eat?

RDS030: I think four.

INTERVIEWER: Four? Sneeze?

RDS030: Mm-hm. My eyes are getting watery.

INTERVIEWER: (Laughs) Sometimes if you look at the light it'll make you sneeze. That doesn't-no?

RDS030: No- makes my eyes more watery.

INTERVIEWER: (Laughs) Oh no. Um, so let's see. So more meals, how many meals d-would you say you eat now?

RDS030: Four.

INTERVIEWER: Four? Did you start doing that right away after you found out you were pregnant or was it kind of gradual?

RDS030: No it was kinda like, I was getting hungry like before I found out I was pregnant I was getting hungry more so I just started eating more.

INTERVIEWER: Okay. So it's sort of been during the whole course of your pregnancy. Um, have you made any changes in the foods that you eat?

RDS030: No.

INTERVIEWER: So you haven't tried to add or remove anything?

RDS030: No.

INTERVIEWER: Are there any foods that you can't stand the smell of or the taste of now?

RDS030: No.

INTERVIEWER: Okay. Um, and you said that you were hungrier now?

RDS030: Mm-hm.

INTERVIEWER: Um, why do you think that you're hungrier ?

RDS030: I don't know. Because it's seem like you get full quick and then you wait a little bit and then you're hungry again.

INTERVIEWER: Um, do you think that as a pregnant woman that you have to be careful about what you eat?

RDS030: Yeah.

INTERVIEWER: Uh, so what have you heard that pregnant woman should be careful about eating?

RDS030: I've never heard of stuff that you shouldn't eat.

INTERVIEWER: Anything specific? Okay. Anything you should eat more of?

RDS030: Like fruits and vegetables. (Laughs)

INTERVIEWER: Are there any kinds that you think are better than others?

RDS030: Like corn and broccoli.

INTERVIEWER: Okay. Do you have a favorite?

RDS030: Well, if you do broccoli and cheese, I'll eat that a lot.

INTERVIEWER: Okay. Um. Are there any foods that you've heard are bad for a growing baby?

RDS030: No.

INTERVIEWER: Okay. And do you, kind of said this but I'll ask one more time just in case it prompts you to think of anything. Is there anything that you've been avoiding um, because you have um, it doesn't taste good anymore?

RDS030: No.

INTERVIEWER: Okay.

RDS030: Cause stuff I didn't like then, I don't like now.

INTERVIEWER: Okay. And, in-in addition to adding fruits and vegetable s are there any other things that a pregnant woman should change about her diet during pregnancy?

RDS030: Less meat.

INTERVIEWER: Less meat?

RDS030: Yeah, less meat, like you know, frying and stuff like that.

INTERVIEWER: Okay. So sort of the way you cook foods.

RDS030: Mm-hm.

INTERVIEWER: And why do you think that that is?

RDS030: Cause too much grease is not good for somebody.

INTERVIEWER: What do you think that, is it not good for the mom or the baby?

RDS030: For both.

INTERVIEWER: For both?

RDS030: Cause you know when you're not pregnant they say not to eat a lot of fried foods cause it'll make you gain weight and, you know, you can get something about the cholesterol something like that.

INTERVIEWER: Yeah yeah. Sounds right.

RDS030: (Laughs)

INTERVIEWER: Um, so have you changed the way you prepare foods at all? So you've talked a lot about fried foods, so have you tried to-

RDS030: Some of them I bake.

INTERVIEWER: Okay. And that's just been since you've became pregnant?

RDS030: No like, I would say like, now.

INTERVIEWER: Just more recently?

RDS030: Yeah. Like more, like more recently, yeah.

INTERVIEWER: Okay, so maybe how many weeks do you think that you've been trying to bake things more?

RDS030: I'd say about two months..

INTERVIEWER: Two months? Okay. Um, are there specific foods that you tried to bake instead of fry?

RDS030: Like meat.

INTERVIEWER: Okay.

RDS030: I-I don't know. Just meat. Cause, you know, other stuff you have to fry. Like fries. You bake them, they're gonna take way too long to be done.

INTERVIEWER: Yeah. So what kinds of meat do you normally eat?

RDS030: Um, like, like drumsticks, chicken wings, um, pork, um what's those things called, like you know the things that come in, they're pork something, I forgot.

INTERVIEWER: Uh pork tenderlions?

RDS030: I dunno, they're called chuletas in Spanish.

INTERVIEWER: Oh, I don't know. (Laughs)

RDS030: Um, they have like a bone in the middle. Like a t-bone.

INTERVIEWER: Oh, so like a pork chop?

RDS030: Yeah.

INTERVIEWER: Maybe.

RDS030: Yeah that's what I meant.

INTERVIEWER: Do you bread your pork chops?

RDS030: No I don’t like meat like that.

INTERVIEWER: Okay.

RDS030: I feel like when you season it, it takes the flavor away. If you put like flour and stuff on it.

INTERVIEWER: Okay. Uh, and so all those are things you’re trying to eat baked…

RDS030: Mm-hm.

INTERVIEWER: …within the last two months more. Okay. Do you ever, um, think about changing foods that are, have more sugar or salt?

RDS030: No.

INTERVIEWER: And, how much dairy foods are you quickly consuming. So like yogurt, milk, cheese.

RDS030: Well I drink milk every day cause you know school. They give you milk, so. I don’t really like cheese.

INTERVIEWER: Okay. And do you eat yogurt or ice cream at all?

RDS030: It depends. What kind of yogurt it is. But ice cream, like be like once a month.

INTERVIEWER: Okay. Uh then have you been craving or have you eaten any nonfood items like baking soda, couch stuffing,

RDS030: No.

INTERVIEWER: Okay.

(both laugh)

RDS030: Ew, that’s nasty.

INTERVIEWER: It happens sometimes when people get really low on iron and then they just crave weird things and they can’t help it. Um, and so, and then no smells are bothering you or seem more appealing to you.

RDS030: Well, I don’t like barbeque sauce, so when I smell it , it like get me nauseous.

INTERVIEWER: And still makes you nauseous?

RDS030: Mm-hm.

INTERVIEWER: Okay. But nothing changed since you became pregnant.

RDS030: Mm-mm.

INTERVIEWER: Okay. Um. Are there any special foods that you're taking as supplements while you're pregnant? So do you have a prenatal vitamin?

RDS030: Mm-hm.

INTERVIEWER: Do you take that every day?

RDS030: Mm-hm.

INTERVIEWER: Okay. Um and..

RDS030: And the pills…

INTERVIEWER: Vitamin D pills?

RDS030: Yeah.

INTERVIEWER: And, um, do those give you any side effects at all?

RDS030: No.

INTERVIEWER: And it's pretty easy to remember to take them?

RDS030: Yeah. Um I have this alarm on my mom's phone and it rings at 9:30 and then it tells me when I have to take it.

INTERVIEWER: Awesome. That makes it really easy. Uh and then do you ever eat foods that are four to five…so like a lot of cereals have vitamins and minerals added. So do you ever eat box cereal?

RDS030: Yeah, but I don't know if they have that stuff in them.

INTERVIEWER: What kinds do you eat?

RDS030: Um, Frost Flakes, um, Lucky Charms, Fruity Pebbles…

INTERVIEWER: All the good ones.

RDS030: Yeah.

(both laugh)

RDS030: The Fruit Loops.

INTERVIEWER: Nice.

RDS030: Anything that's not chocolate, cause I hate when the milk turns black.

INTERVIEWER: Oh yeah? When you eat, um your cereal do you put milk on it?

RDS030: Not always.

INTERVIEWER: Not always?

RDS030: It depends on what cereal it is.

INTERVIEWER: Um, have you received any advice about taking supplements or eating other things that are high in any vitamins or minerals?

RDS030: Yeah, the nutritionist told me. But I don't remember what she has said.

INTERVIEWER: Okay. You don't remember the nutrient or the food or anything?

RDS030: No. (Laughs)

INTERVIEWER: How often do you talk to the nutritionist?

RDS030: Well that was the first time I talked to her ever since um I'm at [?] so.

INTERVIEWER: Okay. So you've only spoke to her once?

RDS030: Mm-hm.

INTERVIEWER: Okay. And did you remember any recommendations that she gave you at all for things to do?

RDS030: Like she told me to eat more dairy.

INTERVIEWER: More dairy. Anything about meat or fruits or vegetables, grains?

RDS030: Well you know, I eat more fruits and vegetables.

INTERVIEWER: Mm-hm. What about bread or pasta?

RDS030: No. She didn't say anything about that.

INTERVIEWER: Okay. Um. So of all the people that have talked to you about anything food related or nutrition related, what source do you think has provided you the best information? It can be a website, or a person.

RDS030: The nutritionist.

INTERVIEWER: The nutritionist?

RDS030: (Laughs) Yeah. That's the only person I can think of right now.

INTERVIEWER: Have the midwives ever talk to you about nutrition?

RDS030: N-no.

INTERVIEWER: No? And have you ever used the internet to look up anything about food like recipes or…

RDS030: No.

INTERVIEWER: Okay. Um, and Baby Center, does that ever have anything about food that you can remember?

RDS030: Not that I can remember.

INTERVIEWER: What about the handouts that you've gotten from [ramp]?

RDS030: Well those I don't read. The little…pamphlets I don't read those. I just read the papers.

INTERVIEWER: Okay.

RDS030: But, [I've been….] foods on there.

INTERVIEWER: What are the pamphlets about? How are the pamphlets different from the papers?

RDS030: I don't know. I just don't like pamphlets so I just throw those away.

INTERVIEWER: Okay.

(Both laugh)

INTERVIEWER: Is it the folding or?

RDS030: Yeah.

INTERVIEWER: Oh, okay. Um, okay so, last section is about physical activity. So, um, do you think that a woman needs to change the amount of exercise or physical activity that she does during pregnancy?

RDS030: Yeah.

INTERVIEWER: How should she change it?

RDS030: Like, if you don't like, if you didn't exercise before, you should exercise now.

INTERVIEWER: Okay.

RDS030: Or if you did too much exercise, cut back on some.

INTERVIEWER: Okay. Um. And did you hear that somewhere or read it somewhere?

RDS030: No, but I know that exercise is good for people, so.

INTERVIEWER: Okay. Um, have you ever heard of specific recommendations about the types of activities that people should do?

RDS030: No.

INTERVIEWER: Anything that people should not do?

RDS030: Not that I know of.

INTERVIEWER: Okay. Has anyone at the clinic tried to give you advice on things to do or not to do?

RDS030: No.

INTERVIEWER: What about Mom? Has she ever said anything based on like her own experiences when she was pregnant with you?

RDS030: No.

INTERVIEWER: No. And if you wanted to know more about exercising, where would you go to look for information?

RDS030: The internet or ask the midwives.

INTERVIEWER: Okay. Are you currently exercising?

RDS030: Yeah. Um, they have gym at my school, but it's different cause I go to the young mother's program.

INTERVIEWER: Mm-hm.

RDS030: So it's like, for pregnant people and stuff like that. So they only have like treadmills, they have a wii, and a tennis table.

INTERVIEWER: So which are your favorite activities to do for that?

RDS030: The wii. (Laughs)

INTERVIEWER: The wii? What games do you have for the wii?

RDS030: Um, dancing games, um, wii sports, um, and the one with the board that you put on the floor and you gotta like step on it and stuff like that.

INTERVIEWER: Okay. D-So you like all those? So you like dancing?

RDS030: Mm-hm. I like that one better than all those.

INTERVIEWER: Awesome. Um. How much have you exercised after becoming pregnant? Is it every day, every other day?

RDS030: Every other day.

INTERVIEWER: Okay. How long do you think you exercise each time that you do something?

RDS030: Well, my gym class is like 45 minutes, so…

INTERVIEWER: Okay, that's pretty good!

RDS030: Yeah, cause when I get home I got to sleep, cause I be tired. (laughs)

INTERVIEWER: Uh, and so has that changed since before you were pregnant?

RDS030: No, it seems like after school, like I be tired so I got to sleep.

INTERVIEWER: Okay.

RDS030: Even when I wasn't pregnant, I used to do the same thing.

INTERVIEWER: Okay. And did you still have gym for 45 minutes in the regular school program?

RDS030: No, in the regular school, it was actually 30 minutes.

INTERVIEWER: Oh, wow. So that's nice. So you get a little bit more. Um. So you said that you've been, you get tired, and you sleep when you get home, but do you think overall, just throughout the day that you have more or less energy than before you were pregnant?

RDS030: Less energy.

INTERVIEWER: Less energy? So do you fall asleep during the day…?

RDS030: Yeah.

INTERVIEWER: Like during school?

RDS030: Yeah.

INTERVIEWER: Okay. And what kinds of activities did you do before you were pregnant? Just the things in gym?

RDS030: Well, um at my regular school we go swimming, you play basketball, they have um volleyball, they have badminton, they have rollerskating.

INTERVIEWER: Are those after school activities? Or…

RDS030: No they're during school.

INTERVIEWER: Okay. Did you do any after school activities?

RDS030: I used to play softball.

INTERVIEWER: Okay. Um, when did you stop playing softball?

RDS030: Well, I didn't play last year.

INTERVIEWER: Okay.

RDS030: I played like the years before.

INTERVIEWER: What position?

RDS030: First base.

INTERVIEWER: Nice.

RDS030: I was scared though, cause you know like, everything depends if you get them out or not so.

INTERVIEWER: It does, that's, that's a, but you have to be good to be the first base, so you must've been really good. Do you think you'll play again after the baby's born?

RDS030: Hopefully.

INTERVIEWER: That'll be fun.

RDS030: Yeah, I like that sport.

INTERVIEWER: Um, so, do you, um, would you like to hear about other ideas for exercising and how you can fit that into your day?

RDS030: Yeah.

INTERVIEWER: And what, what motivates you most about certain activities?

RDS030: It depends if they're fun or not, cause some of them be boring so you just don't want to do them.

INTERVIEWER: What makes them fun versus boring?

RDS030: Like um, you know, like, the treadmill versus the wii, you know. And the wii, I know, you move more and stuff like that, but you know, it's more fun than just sitting in the treadmill and just walking in place.

INTERVIEWER: Do they have treadmills next to each other where you can chat with a girlfriend?

RDS030: Mm-hm. But not everyone in the class likes to participate so.

INTERVIEWER: Oh okay. Do you have friends that you do activities with more often?

RDS030: No, I have friends, but I don't exercise with them or nothin like that.

INTERVIEWER: Okay. Would you if you had a, your friends were interested in doing that with you?

RDS030: Yeah.

INTERVIEWER: Um, so what effects do you think that physical activity has on you and also your baby?

RDS030: Well I have asthma so uh I run out of breath a lot. I don't know how that affects the baby, but.

INTERVIEWER: So if, if you did an activity that didn’t cause your asthma to act up a lot, um so, and you can do it comfortably maybe like one of the wii games. Um, what things would that do for your baby?

RDS030: Well, you’re exercising so.

INTERVIEWER: Do you think it does anything specific though?

RDS030: No, not…

INTERVIEWER: Does it make your baby strong? Or does it give it lots of energy?

RDS030: No.

INTERVIEWER: Not sure?

RDS030: Not sure.

INTERVIEWER: Okay. Um, and how do the things you do currently affect your baby? Do you think that you’re doing a lot of good things? Or you could do things better?

RDS030: I could do things better like going to sleep. I can try to not go to sleep after school.

INTERVIEWER: Do you um, once you go to sleep, do you stay asleep all night?

RDS030: No I wake up like around 9. And then, you know, I go eat stuff, and I can’t go back to sleep until 1 o’clock in the morning.

INTERVIEWER: Okay. Um, if you had to pick certain activities that were best for someone who’s pregnant, what do you think they’ll be?

RDS030: Yoga.

INTERVIEWER: Yoga? How come?

RDS030: Um, you know how you have to do all those movements and you stretch and stuff. I think like people that their back hurts and stuff like that think that that will be able to help.

INTERVIEWER: Have you ever tried yoga?

RDS030: No. (laughs)

INTERVIEWER: Do you think that you’d like to try it?

RDS030: Yeah. At my school we have like these yoga balls, the red ones, the big ones, and the teacher has us like lay on our backs on them, and like you know, roll back and forth. But then it's difficult to get up.

INTERVIEWER: (laughs) Does it help back pain though?

RDS030: Mm-hm.

INTERVIEWER: That's nice. Um, okay, so, just two more questions. Um, what do you hope to get out of the health messages that you've signed up to receive from us? Anything specific about topics or…?

RDS030: Um, like more information on stuff I know. But like, you know, uh, inform me more. Like give me more information than what I already know.

INTERVIEWER: So, you know what you know better than me. So tell me what things that you've thought about oh it'd be cool to know more about that.

(both laugh)

INTERVIEWER: It's a hard question. You can think about it. No rush.

RDS030: Like, um, what kind of exercises.

INTERVIEWER: Okay.

RDS030: What stuff to eat and not eat. Things that help the baby and things that hurt the baby.

INTERVIEWER: Do you have any favorite foods that you'd like to know how to make healthier, that mom could help make healthier so that you can still have your favs?

RDS030: No, cause then it won't taste right.

INTERVIEWER: (Laughs)

RDS030: If you try to make it healthy.

INTERVIEWER: Um, and if we send you messages and they're meant to help motivate you to make choices that will be best for you and your baby, how often do you think you would need to receive messages for that to be beneficial?

RDS030: I say every day.

INTERVIEWER: Every day? So once a day, more than once a day?

RDS030: Yeah, like once a day.

INTERVIEWER: Okay. And overall, is there a certain way that you would like messages to be? Do you want them to be, like, we're talking to you as if we're friends or do you just want them to be straight to the point?

RDS030: Yeah, yeah you can talk like we're friends.

INTERVIEWER: Okay.

RDS030: Cause that'll make me feel more comfortable.

INTERVIEWER: Okay. And um, would you like to receive links to websites and youtube videos and news articles that might be interesting to you? And would you feel comfortable replying to a message that was a question?

RDS030: Yeah.

INTERVIEWER: Okay. Alright!

RDS032

Interviewer: How old are you?
RDS032: I am fifteen years old.
Interviewer: Ok. And, how tall are you?
RDS032: I am 4’11”.
Interviewer: 4’11”. And, how much do you weigh?
RDS032: I weigh… 160 something.
Interviewer: Ok. And is this your first pregnancy?
RDS032: Yes.
Interviewer: And was your pregnancy planned?
RDS032: No.
Interviewer: Ok. And, um, what hopes or goals do you have for your baby to be?
RDS032: Uh, I think …(I think something didn’t record)
Interviewer: Anything else you can think of?
RDS032: No.
Interviewer: Ok. Uh, and what challenges have you faced as you planned to become a mother?
RDS032: Uh, I didn’t hear you. (There was some yelling in the background by someone else).
Interviewer: Um, so, are there any challenges that you have faced after finding out that you are pregnant and planning to be a mom?
RDS032: Uh, yea.
Interviewer: Can you tell me a little bit about those?
RDS032: Um, school, um, telling my family, andddd, that’s about it.
Interviewer: Ok, is there anything particular about school or telling your family that was hardest?
RDS032: Um, no not really.
Interviewer: Just kind of the time and nerves?
RDS032: Yea. yea.
Interviewer: Ok, and uh ok, the last question is this section is what do you think are important things that you should be doing to make sure your baby’s healthy?
RDS032: Um, making sure that I’m healthy. Um, making sure what I can do and I can’t do.
Interviewer: What do you mean by that?
RDS032: Like, um, like lifts and stuff. Making sure I don’t do that.
Interviewer: Anything else?
RDS032: Nope.
Interviewer: And you mentioned that it is important for you to be healthy, can you tell me a little bit more about what you mean by that?
RDS032: Like, um, making sure that I’m taking my medications and eating the right foods.
Interviewer: And for meds, are you referring to the medicines that your doctor gave you or more like supplements with vitamins and minerals?
RDS032: Um, some more my doctor’s prescribing.
Interviewer: Ok.
RDS032: And the vitamin D…
Interviewer: How is taking your vitamin D going?
RDS032: Um, going good.
Interviewer: Are the pills hard to swallow?
RDS032: Yea.

Interviewer: Yea? Do you ever sprinkling it on your food?
RDS032: Yup.
Interviewer: Sometimes that helps doesn't it?
RDS032: Yea.
Interviewer: Ok, so, in the next section it's kind of a whole bunch of things mixed in together. But, I'm going to ask you about nutrition and food um, a little bit about exercise and using the internet on your cellphone. But, it will start with just a really broad question. And that is, I just want you to tell me about um, what it's been like to be pregnant?
RDS032: Alright. … Oh, I could start?
Interviewer: Sorry, yea I probably didn't word that clearly. So, can you just tell a little bit about what it's like to be pregnant and how things have progressed as you have gotten further along?
RDS032: Umm, it's been a little hard. Um, like just the [I don't know what she said]. Like getting up for school … and doing other stuff.
Interviewer: Um, so, in terms of the care that you have been getting at the [name redacted] clinic.
RDS032: Yea.
Interviewer: When did you first visit the clinic?
RDS032: Uhhhh, like a month after I found that I was pregnant.
Interviewer: And, what's the care been like there? Has…What sorts of things
RDS032: (said something in the middle)
Interviewer: I'm sorry. Go ahead.
RDS032: It's been really good. Um, like helping me and stuff.
Interviewer: What do you like most about the clinic?
RDS032: Um, (something) and like going to my appointments.
Interviewer: And, what kind of um, things do the people at the clinic talk to you about, in terms of having a healthy pregnancy?
RDS032: Um, like eating the right foods.
Interviewer: You said fruits?
RDS032: Nope, like eating the right foods.
Interviewer: Ok, so eating the right foods.
RDS032: Watch out for like, what am I doing during, throughout the day. Like walking.
Interviewer: Anything else that they have talked to you about or taught you about?
RDS032: Like stay away from sugary foods.
Interviewer: Ok, um, so based on the information that they have shared with you, so, I want to dig a little deeper into that about food. So
RDS032: Ok.
Interviewer: Can you tell me how you have been eating during pregnancy?
RDS032: Um, I eat like, I eat like snacks. I eat like four, four snacks a day. I eat, um, breakfast, dinner, and lunch. And, throughout the day, I eat like, I eat like four things of snacks.
Interviewer: What's a normal snack like? What do you have for that?
RDS032: I have like, an apple.
Interviewer: And can you tell me a little bit about what you breakfast, lunch, and dinners are like?
RDS032: Um, well, in breakfast, I eat like eggs, poached, or cereal.
Interviewer: And how about lunch?
RDS032: For lunch, I make like a sandwich.
Interviewer: Any snacks with the sandwich, like um, cookies or chips or anything?

RDS032: Um, some chips.
Interviewer: Well, those are good aren't they?
RDS032: Hm-hm.
Interviewer: What flavor?
RDS032: Um, Doritos.
Interviewer: Oh, nice.
RDS032: Yea.
Interviewer: Um, and then, do you ever have fruits and vegetables with your lunch?
RDS032: I have some carrots.
Interviewer: And how about dinner?
RDS032: Um, I eat like pasta. –pause-
Interviewer: So, what kind of?
RDS032: Or
Interviewer: Sorry, go ahead?
RDS032: With some like meat and sauce on it.
Interviewer: Ok. What kind of meats do you normally eat?
RDS032: I eat like ground turkey.
Interviewer: Anything else?
RDS032: Uh-uh.
Interviewer: So, in terms of how you are eating now, how is that different from before you were pregnant?
RDS032: Um, before I was pregnant I wasn't that. I would eat so much.
Interviewer: Did you say you ate not as much before or more?
RDS032: Um, not as much.
Interviewer: Not as much?
RDS032: Yea.
Interviewer: And what about the types of foods? Did that change at all?
RDS032: No.
Interviewer: And since you were eating more, is that, so, you said you eat breakfast, lunch, dinner, and four snacks.
RDS032: Hm-hm.
Interviewer: Which one did you add, so are the amounts at each sitting bigger or did you add snacks or a meal, now that you are more hungry?
RDS032: …Um, like I'll eat like, so for dinner I'll eat like um, more portions. For dinner.
Interviewer: Ok.
RDS032: And that's about it.
Interviewer: So, you were still eating about four snacks before pregnancy as well?
RDS032: Um, no.
Interviewer: No, ok, how did that change?
RDS032: Um… I usually like ate 1 to 2 snacks before I got pregnant, um, and it just feels like I get more hungrier. So, yea it changed to like four.
Interviewer: Ok, did you eat breakfast and lunch before as well?
RDS032: Yea.
Interviewer: And would you say those amounts changed? Or did those stay the same?
RDS032: Those stayed the same.
Interviewer: Ok. So, have you made any changes to the types of foods that you eat?

RDS032: Um, no.
Interviewer: And um, are you hungrier, well you said that you are hungrier now?
RDS032: Yea.
Interviewer: Do you think that there are any foods that a pregnant woman should be careful of eating?
RDS032: Uh, yea.
Interviewer: Can you tell me a little bit about those?
RDS032: Um, so like, stay away from like, really, really salty foods. Um, that's about it.
Interviewer: Any examples about the salty foods you're thinking of?
RDS032: Um, not really.
Interviewer: Ok. Um, what about foods that a pregnant woman should eat more of?
RDS032: Uh, fruits. Vegetables and like, salad.
Interviewer: And, did you just know those things or did you hear those somewhere?
RDS032: I just know those things.
Interviewer: Ok. And, can I ask how you know that?
RDS032: Um, b/c it's more healthy for you.
Interviewer: Just b/c you're pregnant or just in general?
RDS032: Just in general.
Interviewer: Ok. Are there any
RDS032: nope
Interviewer: Things that you have been avoiding or adding to your diet b/c you really like the smell or you really don't like the smell or taste of them?
RDS032: Um, no.
Interviewer: So, no cravings?
RDS032: Nope.
Interviewer: Ok. And how does a pregnant woman need to change her diet and different times during her pregnancy?
RDS032: Um, I don't know.
Interviewer: Or, have you ever heard that they should do that? I guess that is a better question.
RDS032: Yea, I heard that they should.
Interviewer: Ok, do you think that that changes at a certain time or
RDS032: Um, like over time.
Interviewer: So, kind of just like continuous over time?
RDS032: Yea.
Interviewer: Ok. Um, since you've become pregnant has the way that the foods you eat, the way that they are prepared. Has that changed?
RDS032: Umm, no, not really.
Interviewer: So, how um, how do you normally cook a lot of the foods that you eat, or actually, who does most of the cooking for you? You or someone else?
RDS032: Umm, my mom.
Interviewer: Your mom?
RDS032: Hm-hm.
Interviewer: And, does she also do the shopping?
RDS032: Yea.
Interviewer: And do you ever get a say in what foods she buys?
RDS032: Yea.

Interviewer: Do you have any favorites now that you are pregnant?
RDS032: Um, not really.
Interviewer: Is your phone making noises?
RDS032: No.
Interviewer: Ok, I was just hearing feedback. I just wanted to make sure it wasn't affecting your side of the phone.
RDS032: Oh.
Interviewer: So, so you said that you get to help on some of the decisions made on grocery shopping, and you may or may not know the answer to this, but I just want you to make a wild guess, how much money do you think that your family spends on groceries each week?
RDS032: Like, between like $20 and $80.
Interviewer: Ok. Um and then kind of going back to the idea of if you've changed any, the way that you prepare any foods, I want to just ask you about a couple of specific foods. So, after you found out you were pregnant, did anything change about the fried foods that you eat?
RDS032: Um, yea.
Interviewer: Can you tell me how it changed?
RDS032: They told me stay away from them.
Interviewer: Ok. And has that been hard?
RDS032: Uh, not really.
Interviewer: So, how is the amount of fried foods that you eat now different from before?
RDS032: Um, like before I just ate a lot of like fried food, but um, but now I eat like less fried foods.
Interviewer: Can you give me an idea of what you mean by less?
RDS032: Like if we go out, like French fries. Because I can eat a lot of French fries before I got pregnant, but now I eat like, I eat like one or two.
Interviewer: Just one or two fries?
RDS032: Yea.
Interviewer: Ok. And what motivated you to eat less fries?
RDS032: Um, all the salt it.
Interviewer: And what about during pregnancy did you want to reduce your French fries intake for?
RDS032: Uh, yea.
Interviewer: Have you heard of anything specific that salt is bad for during pregnancy?
RDS032: Um, no.
Interviewer: Ok. What about sugary foods? How have those changed since you have become pregnant?
RDS032: Um, like I don't know. I usually don't eat like sugary foods.
Interviewer: Even before?
RDS032: Yea, I didn't.
Interviewer: Ok. So, how about dairy foods? Have you changed how much dairy food you consume?
RDS032: Um, yea.
Interviewer: How has that changed.
RDS032: Um, well I'm like lactose intolerant.
Interviewer: Oh. Ok.
RDS032: So, it is very easily.

Interviewer: So, you don’t eat a lot of dairy food before or after pregnancy.
RDS032: No.
Interviewer: Ok. Do you try to eat anything or drink anything that is lactose free that has the same nutrients in it?
RDS032: Um, not really.
Interviewer: Ok. And
RDS032: I could still like drink milk and eat yogurt. But, I only drink a little bit of it.
Interviewer: Ok. Are you able to eat cheese?
RDS032: A little bit.
Interviewer: A little bit?
RDS032: Yea.
Interviewer: And so, during your pregnancy did you have any cravings for none food items like, liking to chew on ice or eating baking soda or clay or anything like that?
RDS032: Um, no.
Interviewer: Ok. And are there any smells that you like more or less now that you are pregnant?
RDS032: Um, I don’t think so.
Interviewer: Ok. And so you said that you have been focused on taking your vitamins, have you, is it pretty easy to remember to take those?
RDS032: Yea.
Interviewer: Yea? So, how often would you say you remember to take those to take your prenatals?
RDS032: Um, once or twice a day.
Interviewer: Uh, is your pill one that you can take twice a day?
RDS032: Uh, I take the two of them.
Interviewer: Ok, that makes sense then.
RDS032: So, I take them like once or twice a day.
Interviewer: Ok. So, how have your vitamin D pills been? Because I know that you have said that those are a little hard to swallow.
RDS032: Yea, um, I take them like once a day.
Interviewer: And what helps you remember to take it?
RDS032: I use my phone.
Interviewer: Is that an app or did you just make an alarm?
RDS032: Um, I have, I use my alarm.
Interviewer: And, is there anything that motivates you to remember to take those? Other than the alarm?
RDS032: Not really. No, not really.
Interviewer: Did you ever think about your baby when you take your vitamins?
RDS032: Um, no.
Interviewer: Ok. And, the next question is about fortified foods. And I am just curious, have you ever heard about fortified foods?
RDS032: No.
Interviewer: Ok. Well, you said that you eat cereal.
RDS032: Hm-hm.
Interviewer: And you know how a lot of cereals on the side of their box they have lots of different vitamins and minerals listed?
RDS032: Yea.

Interviewer: So, that would be an example of a fortified food because those vitamins and minerals don't happen all by themselves. Someone has to put, add them to the food.
RDS032: Alright.
Interviewer: So, cereal is an example and have you seen the orange juice at the store that says that it has calcium added?
RDS032: Yea.
Interviewer: So, that is another example.
RDS032: Alright.
Interviewer: Do you know of any foods in your diet that have those sorts of labels on them that you know that you eat?
RDS032: Um, a box of pasta.
Interviewer: Ok. Do you know what nutrients, just on the top of your head, are on that box?
RDS032: Like wheat.
Interviewer: And what type of cereal do you like the most?
RDS032: Um, I eat like Fruit Loops.
Interviewer: Ok. Do you put any sort of milk on yours or do you eat them dry?
RDS032: Um, I put a little bit of milk.
Interviewer: Ok. And what advice have you received about how to eat during pregnancy?
RDS032: Um, none.
Interviewer: None?
RDS032: Yea.
Interviewer: So, at the prenatal clinic, do they talk to you about nutrition there?
RDS032: Oh, yea.
Interviewer: Ok. What things can you remember that they told you?
RDS032: They said that nutrition is very, very good. Then, that me and baby needs it.
Interviewer: And did they say why?
RDS032: Um, they did, but I forgot why.
Interviewer: Ok. Do you remember if they said that the benefits were for you or for your baby or for both?
RDS032: Uh, I think that they said for both.
Interviewer: Ok. And what about at home? Has any in your family given you any advice about things to eat or not to eat?
RDS032: Um, not really.
Interviewer: What about anyone sharing experiences about when they were pregnant?
RDS032: Nope.
Interviewer: Um, and what about friends? Do you have any friends that you have talked to about eating during pregnancy?
RDS032: No.
Interviewer: And what about looking on the internet. Have you ever search for anything related to pregnancy online?
RDS032: A little bit.
Interviewer: A little bit? What did you look for?
RDS032: Um, I what to eat. Um, what to do.
Interviewer: What do you mean, what to do?
RDS032: Like, what to do on a regular basis?
Interviewer: When a baby whats?

RDS032: On a daily basis.
Interviewer: Oh. ok. Do you remember where you found that information?
RDS032: On Google.
Interviewer: On Google?
RDS032: Hm-hm.
Interviewer: Are there any websites that you found more helpful than others?
RDS032: Uh, not really.
Interviewer: Ok. Have you ever signed up for email services or blog things where you get updates about your baby?
RDS032: No.
Interviewer: Ok. Um, so you said that you've looked for things for what to eat and what to do. Do you remember any specific searches that you've done?
RDS032: Um, no.
Interviewer: And why did you look for things on those two topics?
RDS032: So, so, yea, like, what do I want and what not to eat.
Interviewer: And do you remember what you found?
RDS032: Uh, not really.
Interviewer: And do you think the information you found is accurate or correct?
RDS032: Um, kind of.
Interviewer: What about it did you think was or wasn't correct?
RDS032: Like, it told a bunch of different things that I should eat or should not eat.
Interviewer: But, you didn't agree with all of them?
RDS032: Yea.
Interviewer: What didn't you agree with?
RDS032: Um, like a bunch of stuff that I should eat.
Interviewer: What were they telling you to eat?
RDS032: Um, I don't know.
Interviewer: Was it specific nutrients or specific foods?
RDS032: Not really.
Interviewer: Um, did you change your behavior after reading about those things?
RDS032: No.
Interviewer: Ok. Um, so let's talk a little bit about physical activity. Have you ever heard any recommendations for how much a pregnant woman should exercise?
RDS032: Um, yea.
Interviewer: What have you heard?
RDS032: Um, like walking.
Interviewer: Anything else that you heard?
RDS032: hold on, hold on (talking to someone in the background) "Hey, I'm on the phone" Hello?
Interviewer: I'm here.
RDS032: Um, like (health??)
Interviewer: What does it help with?
RDS032: Just like your muscles.
Interviewer: Does it do anything for baby?
RDS032: Not really.
Interviewer: So, if it's good for your muscles, what does that mean for your pregnancy?

RDS032: Um, I have no clue.
Interviewer: Oh ok, that's alright. Um, let's see. So do you know of any other recommendations? Is there a certain amount of time or anything that a pregnant woman should exercise?
RDS032: No.
Interviewer: No? You don't know, or there is no recommendation?
RDS032: Uh, I don't know.
Interviewer: Ok. Is there anything you have heard that is not safe for a pregnant woman to do?
RDS032: To lift up heavy boxes.
Interviewer: Ok, and who told you that?
RDS032: Um, I heard it.
Interviewer: Do you remember where you heard it?
RDS032: Um, no.
Interviewer: Has anyone ever tried to give you advice on exercise?
RDS032: Yea.
Interviewer: Who is that?
RDS032: School.
Interviewer: School? Oh, what'd they say?
RDS032: They said um, that I should actually exercise once a day.
Interviewer: And what that just for general health or specifically about pregnancy?
RDS032: Just general health.
Interviewer: Ok. Has anyone in your family or one of your friends given you advice about things to do when you're pregnant?
RDS032: No.
Interviewer: What about the midwives of the [name redacted] clinic?
RDS032: Um, not really.
Interviewer: Ok. And if you wanted to know more about exercising during pregnancy, where would you look or who would you ask?
RDS032: I, um, I would ask my, um, midwife.
Interviewer: And, anywhere else you would look?
RDS032: Um, no.
Interviewer: And, are you currently exercising?
RDS032: Yea.
Interviewer: What do you do?
RDS032: I walk.
Interviewer: How much would you say that you walk?
RDS032: Um, I walk like a mile.
Interviewer: A mile each day?
RDS032: Once a day.
Interviewer: Ok, and did that amount change from before you were pregnant?
RDS032: No.
Interviewer: No, same? Where do you walk to?
RDS032: Um, to (I think she said my house??)
Interviewer: Do you walk anywhere? To school or to the corner store or anything like that?
RDS032: I walk to the corner store.
Interviewer: Ok. And did you do any sports or anything before you were pregnant?
RDS032: Um, no.

Interviewer: How would you say that your activity level has changed since you became pregnant?
RDS032: It didn't.
Interviewer: It didn't change?
RDS032: No.
Interviewer: Do you ever, hmm, feel tired or less tired now that you are pregnant?
RDS032: I feel a little bit more tired.
Interviewer: I'm sorry, what?
RDS032: I feel a little bit more tired.
Interviewer: You're more tired?
RDS032: Yea.
Interviewer: Ok. Do you sleep more or do you just feel tired?
RDS032: I sleep more.
Interviewer: How has your sleep changed?
RDS032: Um, I sleep more throughout the day.
Interviewer: How many hours a day do you sleep now do you think?
RDS032: Like 16.
Interviewer: And, how does that compare to before you were pregnant?
RDS032: Nothing really.
Interviewer: Can you say that one more time?
RDS032: Nothing really. It didn't change or anything.
Interviewer: So you still slept 16 hours a day?
RDS032: Yea.
Interviewer: Ok. What have you heard that exercise does for your baby?
RDS032: Nothing.
Interviewer: You haven't heard anything about that?
RDS032: No, not yet.
Interviewer: Ok. And how does your current amount of exercise affect your baby?
RDS032: Hmm, it doesn't.
Interviewer: Ok. And have you heard of any advice about how much weight you should gain when you pregnant?
RDS032: Yes.
Interviewer: What have you heard?
RDS032: Um, not to gain that much weight.
Interviewer: Is there a certain amount that's best?
RDS032: Um, they told to gain weight of about 5 to 15 pounds.
Interviewer: Hm, who told you that?
RDS032: My midwife.
Interviewer: And does she check in and tell how you are doing with that every time you visit?
RDS032: Yes.
Interviewer: So, how is that going?
RDS032: Going good.
Interviewer: Good. So, you are within the range she wanted you to be?
RDS032: Yea.
Interviewer: Did she say why it's important to gain within that range?
RDS032: Cause I'm so short.

Interviewer: Can you say that again?
RDS032: Cause I’m so short.
Interviewer: Oh, because you’re short?
RDS032: Yea.
Interviewer: I didn’t think you were short.
RDS032: Well she said compared to how small I am.
Interviewer: Did she say anything about um, the weight gain in terms of how your, how your pregnancy will go?
RDS032: Um, no.
Interviewer: Ok. And would you say that you’re going your midwife’s advice on weight gain?
RDS032: Um, not really.
Interviewer: Not really? How so?
RDS032: Wait, I didn’t really hear that question.
Interviewer: Um, so, I was asking how difficult it is or easy it is to follow the advice that your midwife gave you about gaining weight.
RDS032: Oh, it’s, um, kind of easy.
Interviewer: Well, what makes it easy?
RDS032: Um, I don’t know like some foods make me gain weight and some foods doesn’t.
Interviewer: And you’ve been able to figure out which foods help you gain when you need to and those
RDS032: Yea.
Interviewer: Ok. Um, do you think that the amount of weight that you were told you gain is a good recommendation for you?
RDS032: Yes.
Interviewer: And, how come you think that?
RDS032: Um, I don’t know.
Interviewer: Um, and so, as for weight gain, you said that you talked to your midwife about that—have you ever looked up any information on the internet about that?
RDS032: No.
Interviewer: If you wanted to, how would you look that up?
RDS032: On Google.
Interviewer: On Google? Is there any website you might pick to go to from the Google listserve?
RDS032: Um, I don’t know.
Interviewer: Do you just click on all the links on Google from the top to the bottom?
RDS032: Yea.
Interviewer: Do you ever skip over any that would be helpful?
RDS032: Not really.
Interviewer: Not really?
RDS032: Yea.
Interviewer: How many links do you normally click on when you search a topic?
RDS032: A few.
Interviewer: A few? Like 3?
RDS032: Yes.
Interviewer: Or do you think more or less than that?
RDS032: Like 3.
Interviewer: 3? Ok.

RDS032: Hm-hm.
Interviewer: And when you are looking at the links online, do you have a way of deciding which ones you will click on?
RDS032: No.
Interviewer: Do you just click on the first three?
RDS032: Yea.
Interviewer: And have you ever read anything on the internet that made you change your beliefs or behaviors during your pregnancy?
RDS032: No.
Interviewer: Ok. Um, and then when you get on the internet, do you use the computer or do you use your cellphone?
RDS032: I use my cell.
Interviewer: Your phone?
RDS032: Hm-hm.
Interviewer: And have you joined any email or text messaging things that you get information from, like a daily pregnancy message?
RDS032: No.
Interviewer: Ok. And do you have a home computer?
RDS032: Um, no.
Interviewer: Ok. And based on your experiences, is there a place or a person that provides you with the best information about pregnancy?
RDS032: Um, I don't know.
Interviewer: Who, who do feel most comfortable to talk to about pregnancy issues?
RDS032: Um, school. (said something else)
Interviewer: Who do you talk to at school?
RDS032: Um, one of my teachers.
Interviewer: Hm, do you stay after school to talk to her about that?
RDS032: Um, no.
Interviewer: Would you mind telling me a little about what things you talk to your teacher about?
RDS032: Um, I don't know like anything.
Interviewer: Well, what things about your pregnancy?
RDS032: Just the basics.
Interviewer: What do you mean by basics?
RDS032: Just like basic stuff, like what I need to know.
Interviewer: For the delivery?
RDS032: Yes.
Interviewer: She sounds like a nice teacher.
RDS032: Yea.
Interviewer: What does she teach?
RDS032: Um, she's a (I couldn't hear).
Interviewer: What did you say?
RDS032: She's a (I couldn't hear).
Interviewer: Are, are you in the young mother's program?
RDS032: Yes.
Interviewer: Oh ok. And so, I've heard they have some nice exercise stuff for you guys.
RDS032: Yea.

Interviewer: What is your favorite thing to do there?
RDS032: Oh um, I usually don't do them.
Interviewer: Oh, you don't?
RDS032: No.
Interviewer: How come?
RDS032: I don't know.
Interviewer: Are, are close with any of the other girls in that program?
RDS032: Yea.
Interviewer: Do you guys ever talk about the things that you are doing for getting ready for the babies?
RDS032: Yea.
Interviewer: What do you talk about?
RDS032: Like what..
Interviewer: I'm sorry what?
RDS032: Like what we got for the baby.
Interviewer: Oh ok. Do you ever talk about your experiences being pregnant?
RDS032: Uh, not really.
Interviewer: Why do you think that is?
RDS032: Um, I don't know.
Interviewer: Are there people that are, that are in that group that are not very friendly?
RDS032: A couple.
Interviewer: A couple?
RDS032: Hm-hm.
Interviewer: So, is it hard to talk about things that because you don't want them to know or is it something else?
RDS032: It's like more about stuff I don't want them to know. (This is not perfect)
Interviewer: Stuff you don't want them to know?
RDS032: Yea.
Interviewer: In, in the young mother's program is there any classes that you take about nutrition at all?
RDS032: No.
Interviewer: No? Um, and you said that you are close to a couple of girls in the young mother's program group. Do you ever hang out with them outside of the group?
RDS032: No.
Interviewer: Ok. And what about your mom or a sister or aunt. Have you ever talked to them about any of your concerns about your pregnancy?
RDS032: No.
Interviewer: Ok. Alright, well I just have a couple more questions. And then we will be all done and you can get on to your awesome weekend.
RDS032: Ok.
Interviewer: Sound like a plan?
RDS032: Yup.
Interviewer: Ok, so, so you signed up for the Facebook group and you are already linked up to that which is great, I'm just curious, so you signed up for this study and I want to know what you hope to get out of this study. What kinds of things do you expect us to send you?
RDS032: Uh um, good information.

Interviewer: Information about what?
RDS032: About anything.
Interviewer: Anything? You don’t care what it is?
RDS032: No.
Interviewer: Is there anything you want to know more about?
RDS032: Not really.
Interviewer: Um, and how often do you think you will check the Facebook page?
RDS032: Uh, like once or twice a day.
Interviewer: Ok. And then, so you know yourself better than I know you, so I’m just curious, how many messages do you think we should send a week to motivate you and have the best pregnancy you can?
RDS032: At least once.
Interviewer: At least once?
RDS032: Uh-huh.
Interviewer: So, if you, in a perfect world, you got to say how many times we sent a message, how many would you want ultimately?
RDS032: Just one.
Interviewer: Just one per week?
RDS032: Yea.
Interviewer: Would sending messages more than once a week be too much?
RDS032: Um, no.
Interviewer: How about everyday?
RDS032: Yea.
Interviewer: That would be too much?
RDS032: A little bit.
Interviewer: A little bit? What, so, what number would say between once a week and more than a once a week would be the right amount of days to send messages?
RDS032: I don’t know. Like twice?
Interviewer: Twice? Ok.
RDS032: Uh-huh.
Interviewer: And just out of curiosity, have you checked the study site so far?
RDS032: Yes.
Interviewer: What did you think?
RDS032: Um, it’s ok.
Interviewer: It’s ok? What would make it better than ok?
RDS032: Um, if there was a little bit more stuff on there.
Interviewer: If there was a little bit more stuff there? Well, hopefully we will have more stuff once we actually started, so um, stay tuned.
RDS032: Alright.
Interviewer: Ok, so is there anything else that you were thinking about while we were talking that you wanted to add to anything?
RDS032: Uh, not really.
