# Supplementary material for: A social media intervention to improve nutrition knowledge and behaviors of low income, pregnant adolescents and adult women
Source: PLoS One. 2019 Oct 24;14(10):e0223120. doi: 10.1371/journal.pone.0223120 (PMC6812786; doi:10.1371/journal.pone.0223120)
Supplement: S7 File — (PDF) [file pone.0223120.s008.pdf]

S7 File Post-Interview Transcripts

ADL1

INTERVIEWER: So, I'd just like to kind of start by asking how things have been going uhm since we last talked which was awhile ago so… Any thoughts you have on that, or…

ADL01: Things have been going good. It's been really good. It's been a much more uncomfortable pregnancy, like hips and everything, but other than that, things have been going pretty good. Moving along fast.

INTERVIEWER: Good. Uhm what would you say has been the best part of this pregnancy?

ADL01: Oh boy. To be perfectly honest, I hate being pregnant, so (laughs) that's a tricky one. Uhm the best part, maybe just seeing NAME? get excited about him and his sibling. Yeah.

INTERVIEWER: Oh, that's really cute.

ADL01: Yeah

INTERVIEWER: Uhm and last time we talked I've asked you uh what hopes and dreams you had for your baby to be, and uhm have any of those changed?

ADL01: No, I don't think so. At this point, my hope and dream is that we get a name. (laughs, mumbles)

INTERVIEWER: Do you have some choices or narrowed down to some choices?

ADL01: We have it down to four, and every time we settle on one, another feels better… and then we'll switch to that one and then the first one—we just can't land on anything. We think we'll have to see him.

INTERVIEWER: I think that'll definitely help.

ADL01: Yeah

INTERVIEWER: Okay. So have you changed the way that you're preparing foods since we last talked?

ADL01: No. I don't think so.

INTERVIEWER: Okay. Uhm… any changes in cravings that you've had?

ADL01: I've started to crave sweet things more which I didn't at all before, and I didn't really tolerate well so I've liked frozen yogurt a lot and things like that where usually I'm not a fan of that at all… so more sweet things unfortunately.

INTERVIEWER: And any non-food item cravings?

ADL01: No.

INTERVIEWER: Okay. And any challenges you've faced with this pregnancy compared to your past pregnancy?

ADL01: Uhm I would like to say my hips and tailbone have hurt much much more than last time, and I've also swollen more than I ever did. But I think that has more to do with ending in the winter with the other one as opposed to this one being warmer weather.

INTERVIEWER: Uhm and have you found anything that helps kind of overcome those feelings?

ADL01: The swelling and things like that?

INTERVIEWER: Mhm

ADL01: Really just laying in bed overnight makes the ankles go back, but they just swell again. Uhm and then with the hips and stuff, I just put IcyHot on a lot of the time and hope for the best (laughs).

INTERVIEWER: And uhm… let's see. So what would you say are some things that you specifically did to make sure that your baby was healthy?

ADL01: Uhm well just taking care of your body in general. I don't like to eat a lot of… superprocessed candy or food… or in general I don't really like food like that, but I'm more stringent about it when I have a baby. Other than that, I've been staying healthy by exercising, too. Nothing too out of the ordinary I would say, though.

INTERVIEWER: Uhm and as far as advice goes, has where you looked for advice changed at all?

ADL01: No. Just asking friends, asking—checking things online and what not. But uhm, yeah that's about it.

INTERVIEWER: Okay.

ADL01: Yeah.

INTERVIEWER: Uhm let's see… and are you still accessing the Internet from mostly your cellphone?

ADL01: Mhm, yeah. Yeah.

INTERVIEWER: And do you have a home computer with Internet as well?

ADL01: We—yeah, I have a laptop, but my husband takes it to work so it's never home when he's working, but it's home every night, so…

INTERVIEWER: Okay. Uhm and as far as sites where you looked for information, uhm can you just tell me again what sites that you have used and what things that you visit?

ADL01: Sure, a lot of the time it's babycenter.com. Sometimes I'll even Google things and then look for a site that looks trustworthy. Uhm most of the advice I would say I get from is just friends though—personal friends. But that's really the bulk of it.

INTERVIEWER: Uhm and so… you've gotten about I think nine or ten mailings now. So… tell me a little bit of what your thoughts were on the content of that information.

ADL01: Sure. Yeah I thought they were helpful. I've actually enjoyed getting them each time and reading through them. Yeah I think they have some good ideas and things. Most of the time I'll say, "Oh yeah, I think I knew most of it." But there's always a couple things you learn about and little tips or ideas you get. So I think they're helpful.

INTERVIEWER: Okay. Were there anything from those that you tried if they have an idea?

ADL01: In particular I can remember the one on dairy. It talks about doing coffee with a lot of milk just to have extra milk. And I'm not a milk drinker, but I've started doing an almost 50:50 with coffee, so that was cool.

INTERVIEWER: Okay. And what about some of the links that were embedded within documents? Did you ever go to any of those links?

ADL01: Uhm I did one of them for the relaxing music one time. It was actually more for my daughter than for me cause she hates getting in the bathtub and getting out and getting dried off. (laughs) to relax her I thought I'd give it a shot. (laughs)

INTERVIEWER: But otherwise you're—it sounds like you're more reading the content and—

ADL01: Yeah, yeah.

INTERVIEWER: Okay. Were there any topics you found were more helpful for this pregnancy than other topics?

ADL01: Mmm… not in particular, no. They kind of just hit up different points like just about equally interesting I would say.

INTERVIEWER: Okay. Are there any topics that uhm in thinking more broadly for all pregnant women that you think would be more important to focus on more regularly than having sort of a hodge podge of topics?

ADL01: I don't think so. I like that it's been different things each time. I think it keeps it more fresh, and you want to read the different ones rather than being too repetitious, so…

INTERVIEWER: okay. And as far—so different amounts of text. Sometimes its lists for really short items…

ADL01: Oh can you hold for one sec? (Interruption)

INTERVIEWER: Uhm so I wanted to ask you of all the things that we sent, what do you think if you tried anything from any of the letters or the sheets, what was the most motivating about the things that you did try?

ADL01: Mm… let me think. Like which tip was the most motivating you would say?

INTERVIEWER: Like what about a message motivated you to try it?

ADL01: Gotcha. I think that it was just from all credible sources and that it was very like, friendly and not pushy or… saying like you should've been doing this by now. It was more like "give this a try!" I like the language of them.

INTERVIEWER: Okay. Uhm and were there any things you didn't understand cause it wasn't written well, or…

ADL01: No, I don't think so.

INTERVIEWER: Okay. Uhm and for you personally, what information do you think was most relevant to you?

ADL01: Uhm… I'm trying to think. I think I just overall I'm thinking in particular like those little sheets that have ten ideas based on whatever nutrient you're talking about… and I like those and I always look over them. They're very quick uhm and they're very helpful and there are days and weeks when you're pregnant and you go to make dinner and you just don't want to do anything but you know you have a nice thing about having half your plate be fruits and veggies and it kind of really encourages you to stay on track, you know… so. That's what I'm thinking most about. Or having good dairy or… you know each thing each week was like a great reminder.

INTERVIEWER: Uhm and did you change any of your eating behaviors because of something that you received?

ADL01: Uhm like I said, the milk with the coffee much more. I'm not a very big dairy person, uhm. Well particularly milk. I love yogurt and things like that. Uhm but that would be a big change that I've done, especially if I do iced coffee. It's like a 50:50 split and it wasn't anywhere near that before so… that would probably be the biggest one.

INTERVIEWER: And what about exercise? I know that you were a big exerciser before.

ADL01: Uhm well generally by the end, you have to lessen it you know more and more… I'm still doing it five times a week, but it's much much more modest and… if things start to hurt I just stop. So instead of going the average 30-40 it's probably more like getting 15 to maybe 25 but more like 15 to 20 I would say, so… especially with the tailbone and hip things. Like I was telling you a lot of my stuff is like side-stepping you know and it just hurts too bad after awhile so.

INTERVIEWER: I can imagine. That would not be pleasant.

ADL01: yeah

INTERVIEWER: Uhm did you ever share any of the information you received with other women?

ADL01: Uhm… no other women. I would kind of bounce it off with my husband and stuff, but not other women. Although I've had a ton of pregnant friends at the beginning of this pregnancy, and I'm like the last one at this point… so since I've been getting them, I haven't had a lot of pregnant friends that I would share it with.

INTERVIEWER: Okay. And did your husband agree or disagree with any of the information?

ADL01: He uhm is not as big of a health fan as I am so you can't disagree with it, but he's not usually as excited about it we'll say (laughs)

INTERVIEWER: Uhm and I know that you had received a couple recipes along the way. Did you ever try any of the recipes?

ADL01: With the—I remember only receiving recipes with the kind of St. Patrick's Day theme. Those are the only ones I remember getting. Uhm and it was after by the time I received it. Cause we actually made Irish soda bread, and I think it was a whole wheat one, which I would've given a shot to but it was after we had already done it. Uhm but I kept a couple of them that I thought I might use like… there was an apple crisp or something like that, too. But I haven't cooked them yet.

INTERVIEWER: Okay. Uhm… was there anything that was sent that you felt you had better information somewhere else on the Internet or in a book that you've read?

ADL01: Uhm I can't say that was the case. I have to say it was pretty good overall. Like pretty standard, but pretty good.

INTERVIEWER: Okay.

ADL01: Yeah.

INTERVIEWER: And if we had wanted to be a little bit better than standard, what things would you suggest adding?

ADL01: uhm all I can think is if you wanted to just either do more recipe ideas to give people—or even adding grocery shopping lists to give recipe ideas—uhm or I'm always looking—this is just for me personally—but for more fruit and vegetable side dish ideas and recipes and things. So those are always great and helpful to try out. And then maybe just… I'm trying to think. Maybe talking about like importance of organic food and if it really makes too much of a difference… that'd be really great when you're pregnant to be looking for… or just in general if you're craving this—say in the past couple of weeks, I've been craving a lot of sweet things. Say something like if you're craving this, try this. It's very close in taste but much, much better… you know what I mean?

INTERVIEWER: Mhm, yea, those are really good ideas. Thank you.

ADL01: Sure.

INTERVIEWER: Let's see… How did the information that you received influence your own feelings about weight gain?

ADL01: Uh it didn't influence it too much. Uh I've gained more than I would like to with this pregnancy, but I've tried to keep the thoughts of that at an arm's distance and not worry too much. I know I'm kind of on the top of on track, so it's not like bad or anything. But I think—[name redacted]—I don't know if I remember telling you—pregnancy was a little bit different cause the weight gain was so weird. She was so small. Uhm that I kind of went through all the wondering and worrying with that one and this one I'm just more like… hmm it'll work out, you know, so…

INTERVIEWER: Uhm… do you think that the information you received influenced your weight gain at all?

ADL01: Uhm… I don't. I think uhm, I think I'm ending really close to how I ended with her, and I think it's just my body at the end of the pregnancy, so… I don't particularly think of it.

INTERVIEWER: Okay. Uhm and… we kind of talked about this a little bit, but I'll just ask again in case you think of anything else. Did you change any of your eating habits uhm so in relation to whole grains, fiber, fruits and vegetables… I know you added the habit of the coffee and milk.

ADL01: Sure. Yeah, with the fruits and vegetables I've always been more of a stickler but unfortunately even well before the beginning of this pregnancy, I've pushed my husband as

far to the line currently as he'll go, so I couldn't go a little farther with him. It's a give and take. Uhm but in regards to whole grains, him and [name redacted] love popcorn, and generally I'll be like I don't know… but I read that it was a whole grain snack on one of your things, so I've been letting them have more popcorn (laughs) but also just thinking about in general, we try to do more whole grains, too. Uhm so because I would say, I was familiar with about 60 to 70 percent of the stuff that you sent, uhm and other things I'm familiar with. My husband's not as excited to change. If I had a very pliable family, I probably would've changed a little bit more but… I'm just one day at a time exposing them to new things. They're both very picky.

INTERVIEWER: Uhm so did you learn anything new about any like research on different things that physical activity or nutrition have on either your health or the baby's health?

ADL01: Uhm one thing, because of having the swelling in the last week, I looked that up a little bit more uhm not in the things that you've sent, but just online and I was curious, you know. Is it better to put your feet up and rest or is it better to get out and be doing things with the swelling? And it's been a mix that I've found, anyway, but it's—one of the things was getting up and walking around should help your swelling too. So that's one thing I learned, cause I was really severe with swelling and didn't know how to deal with it because I didn't have it with her.

INTERVIEWER: Okay.

ADL01: Yeah.

INTERVIEWER: And did you ever try any of the exercise related links—there might have been some YouTube videos on yoga workouts or poses that are good for back pain… those sorts of things?

ADL01: Honestly I didn't, only because I was kind of already set in my way with my routine. But if I hadn't already established a routine, I probably would have given everything a try. If this was my first pregnancy, I would of just because I have really liked—and it's been working for me. The thing that I have… I haven't really checked it out much.

INTERVIEWER: That makes sense. And have you added any additional pregnancy apps to your cellphone at all over the course of pregnancy?

ADL01: Not this time. I actually kept everything that I used with [name redacted] uhm from the last one. I have one that would be good for when I go into birth—uhm labor—and another one that's good for breastfeeding afterward, but I don't have any like pregnancy apps.

INTERVIEWER: Okay.

ADL01: Yeah

INTERVIEWER: And have you heard of any text messaging programs that exist for pregnant women?

ADL01: Just the one we talked about last time. I think it was called Text for Baby or something like that.

INTERVIEWER: And if you remember you said that you had started it, and it was not very helpful?

ADL01: It was a little bit too behind when they were sending you updates based on where you were, so it wasn't helping at all.

INTERVIEWER: Okay. Uhm… So based on your experiences, is there a place—so a place that on your own gets you to books or internet sites uhm or a person that has provided you with the best pregnancy information?

ADL01: I would have to say, if it had to be one source, I would pick the website baby center, but if it could be a collective, like conglomerate my friends put together. I would have that being the top one. It's not one particular friend, but I like to bounce off a lot of friends with different problems or you know, questions and things like that.

INTERVIEWER: And for you personally, what do you think is the biggest motivator for you to want to eat healthy and exercise?

ADL01: I think just… coming from a place—having time in my life when I wasn't in shape, I value how good you feel when you are in shape, and you just are much more alert, and your mind is more focused because you're not thinking about how you don't like that you're not in shape. So for me, I wouldn't want to go back to that.

INTERVIEWER: And is that the same thing that motivates you during pregnancy you think or is it something else?

ADL01: Yeah, I try. Even if I have strong cravings for food that's terrible, I try to remember that this is not even a year. It's not going to be worth gaining a ton of weight for a short time that you'll take years to try to work off, you know? And I try to balance it in the long term scheme of things.

16:41
INTERVIEWER: And do you think the types of mailings that you received would be good things for health care providers to provide to all women during pregnancy?

ADL01: It could be, yeah.

INTERVIEWER: And what uhm frequency do you think that women would want to receive that sort of information?

ADL01: You know, I think it's going to depend on the pace of life for the person. Uhm… maybe even if you wanted to have it work out for everybody, do like a packet a trimester and then let them go through it however quick they want to. But I don't know for sure. I like weekly because it's little bit-sized bits that I could kind of work through. But other people, you know they have really busy times, and they might want to work more with their schedule.

INTERVIEWER: Do you think it would be helpful to have more information that's interactive, like the challenges that you received, or the papers that you can fill out and track weight gain and those sorts of things?

ADL01: Yeah, I think those were helpful, and it keeps you definitely more focused, like with the water and everything. It helped me… pay more attention to how much water I drink. And I did take pictures of plates. I'm realizing now I never emailed you those pictures, but I did participate if that's probably too late but…

INTERVIEWER: Oh no. It's not too late. You can still send them.

ADL01: I only got two that week, but I'll send you three if you want.

INTERVIEWER: Sure, sure. Yeah, that would be great. And yeah. Still open, definitely (laughs)

ADL01: Actually, would it ever work, or maybe for the study you have to do it particularly but can I text you though? Because they're right on my phone.

INTERVIEWER: Yeah, yeah. That'd be fine.

ADL01: Great

INTERVIEWER: Yeah you have my—number cause I called you from—

ADL01: Yep.

INTERVIEWER: So looking back, I'm just curious. So the study had originally offered a couple different ways in which you could receive information. So receiving paper—would that still have been your choice? Or would you have opted maybe for the Facebook group or text messaging?

ADL01: Uhm I'm just a paper girl. I know I'm an old soul and not many people are like that. Texting is fine, too. I just do better when something's right in front of me. I can't forget about it, and I put more attention toward it. But I know I'm in the super minority of that, so…

INTERVIEWER: That makes sense. I also enjoy paper (laughs)

ADL01: Yeah.

INTERVIEWER: You can interact with it and write on it…

ADL01: Yeah. That's—when I see something in print, I remember it much better. And my husband's not like that at all. He battled me to get rid of our paper calendar forever, but I'm a paper person. Yeah.

INTERVIEWER: You can always be you have your digital one, and I have my paper one… So is there anything else that I didn't ask you about that maybe you thought about the mailings or things that you changed or didn't change because of something that you received?

ADL01: Not really that I could think of. I think we covered just about everything. I was really excited to win this—I love this, by the way. Like love… yeah. I have it almost every day now. Yeah. Thank you.

INTERVIEWER: You're a very good hydrator, so I figured that would be a good thing to have.

ADL01: It was. I was looking for a nice big one like this, and I was like oh my gosh. Cause it was like perfect. It was exactly what I was looking for.

INTERVIEWER: Awesome. That's perfect. Uhm… so I mean I think we covered a lot of the questions that I had, and it sounds like… you were really vigilant about making really healthy choices from the get-go, so…

ADL01: Thank you

INTERVIEWER: I realize a lot of the questions maybe don't apply somewhat but uhm… so I don't think I have any other questions, really, once I go and piece both the interviews together.

ADL01: Sure

INTERVIEWER: But uhm I did want to say we could do uhm your final diet record now… uhm if you want. And then that'll be over with. So that takes like 15, 20 minutes. So uhm… before we do that though, just so I don't forget, so I think we had mentioned the timing of the focus group to you at some point.

ADL01: Yeah. [name redacted] told me it was going to be late, but I didn't know exactly when it was going to be.

INTERVIEWER: So they were planned actually for next week on Tuesday and Thursday.

ADL01: Okay, and uhm I'm just looking at my calendar. Uhm and what were the times of those?

[arrangement for focus group meeting]
[diet recall]

ADL05

Interviewer	um so since it's been a while since we've last talked um I just wanted to catch up a little bit and so how has your pregnancy been going?
ADL05	it's been going good (laughs)
Interviewer	Any, um exciting moments or not so exciting moments?
ADL05	Um I get excited sometimes (Interviewer: Mhm), most of the time, as I'm getting closer
Interviewer	And this is going to be a boy right?
ADL05	Yes.
Interviewer	Okay, um what would you say has been the best part of your pregnancy?
ADL05	Um, probably just feeling him kickin, stuff like that.
Interviewer	And um last time we talked I had asked about what your hopes and dreams were for your baby to be, and um can you tell me a little bit about how you foresee the near future?
ADL05	Um well I just hope right now that he comes out healthy and strong and that I pull through this um labor, healthy and strong also and after that I, hope how it's gonna be I'm not sure
Interviewer	Yeah that seems to be a pretty common answer, do you have any concerns about the delivery at all?
ADL05	Not at this moment, not really, I'm kinda nervous about the pain, but that's about it.
Interviewer	I would be too haha, that's definitely understandable (both laugh). Um, so let's talk a little about food. Um have you changed the way that you've been eating since the last time that we talked?
ADL05	Um, I just don't eat late at night anymore, or like after a certain time. That's about it.
Interviewer	And is there a reason why?
ADL05	Just because of, if I eat um close to bedtime I won't be able to go to sleep really and then I have heartburn.
Interviewer	Okay, um and are there any particular types of food that you eat more of now, than you didn earlier in pregnancy?
ADL05	Yes, I really ate a whole lot of celery for some reason.
Interviewer	Anything else that you eat more of?
ADL05:	
Interviewer:	Um not really, just the celery.

ADL05	And anything that you've stopped eating?
	Um. (Child mumbles in background, ADL05 says be quiet) Um not really nothing that I stopped eating, I mean I cook certain foods, like I just ate the fried chicken but I don't cook fried chicken anymore. Like if my mom didn't make it I would have never ate it, so I usually don't really eat fried foods like that.
Interviewer	And is that just because you choose not to cook using that method or is it the smell from the cooking?
ADL05	It's kind of, I just, I don't want it and I haven't been having the taste for really like that.
Interviewer	Okay. And is that something you would've eaten more regularly before pregnancy?
ADL05	Yes.
Interviewer	Um and can you tell me a little bit about your dairy food consumption?
ADL05	Um I eat cheese, no milk or anything like that, I can't, I don't drink milk, but I do, I will eat it with cereal. That's about it. Um.
Interviewer	But it doesn't make your stomach upset. You just don't-
ADL05	I just don't want to drink it haha
Interviewer	Don't care for the taste?
ADL05	Ya
Interviewer	Um so you mentioned that you've been chewing ice, have you had any craving or thoughts about eating non-food items, like powders or dirt or anything like that?
ADL05	Yeah it all comes to your mind like the powder and the dirt throughout the day. It's just so weird.
Interviewer	Um any particular kinds of powder?
ADL05	Um baby powder.
Interviewer	Oops can't spell haha. And are those things that, the craving has been so strong that you have eaten them, or have you been able to avoid eating them?
ADL05	Um I actually tried baby powder one time, so that's how strong it was but then I talked to the doctor and then I had to really just not, not do it.
Interviewer	What did the doctor say about?
ADL05	She didn't say too much about the baby powder, she just said you know, you don't want anything to harm the baby, so I said "okay" I will not, and even the smell, like what is it, is it comet, like I like to smell it and she said you shouldn't consume that smell, but you know it smelled so good, but I just stopped smelling stuff also.
Interviewer	And did she mention anything about your diet that could cause those things?
ADL05	Um she didn't, I don't think so, but I know I just found today that I 'm anemic, but they didn't tell me this whole time, so I thought okay maybe that had something to do with it, I'm not sure.
Interviewer	And did they give you anything for the anemia?

ADL05	Yeah they just did today haha so.
Interviewer	Are they just um iron pills?
ADL05	Ya.
Interviewer	Okay. So you haven't gotten to try taking them yet?
ADL05	No.
Interviewer	Um. Let's see have any foods given you a hard time, I know you mentioned heartburn, are there any foods that specifically caused the heartburn?
ADL05	Um, really anything, everything causes the heartburn, even if I eat celery, anything that's.
Interviewer	And that only happened after pregnancy, and you didn't have the heartburn before?
ADL05	No.
Interviewer	Okay, um and have you found anything that helps with the heartburn?
ADL05	Um sometimes TUMS.
Interviewer	Um, what things have you done throughout your pregnancy to make sure that your baby is born healthy?
ADL05	Um, just trying to eat healthy, trying to not to stress, stuff like that.
Interviewer	Are there any um, people who have helped you make sure that you eat healthy and not feel too stressed during pregnancy?
ADL05	Yeah, probably uh the dad.
Interviewer	Mm how does he help with those things?
ADL05	He makes sure that I eat, like If I'm hungry, if I'm just like "nah I'm just not gonna eat" he'll make sure I eat even with heartburn and things like that.
Interviewer	And goes on Tums runs and (both laugh). Um how has the internet played a role helping you eat healthy and not stress too much?
ADL05	Um probably just, it just being available with whatever questions you need, ask, you just type it in, in google and anything like that.
Interviewer	Mhm, and when you um, type in questions on google, how do you choose which of the listings that come up to look at, because I know you get so many and it can be hard, so do you have like a system of how you decide on which ones to look at?
ADL05	I usually just go with the first one and the second one, just on the first page. I'll look at all of them on the first page.
Interviewer	Mhm, do you ever feel like any of the ones you click on don't have good advice?
ADL05	Sometimes, you know. Just depending on…
Interviewer	How do you know the difference when you, after you look at it if it's a good source or a bad source?
ADL05	Um, usually if, if it's not from a doctor's point of view and somebody's opinion or what they're going through I'll see if it's similar to me, if it is similar to me then it isn't a good source.

Interviewer	Okay, um, and where do you typically gain access to the internet?
ADL05	Um, at my mom's house
Interviewer	And does she have a computer?
ADL05	Um, well she just has wifi, so I just get on my phone with the wifi.
Interviewer	Okay. Um, and can you remember what types of information most of your google searches have been about?
ADL05	Um, heartburn, and uh mmm probably I know I said before when can you find out the gender of the baby.
Interviewer	Anything that has to do with food or exercise?
ADL05	Um, I think I um, looked up "can you…what is it…some dance at the YMCA with my mom" I forgot what it's called. I know it's fast dancing.
Interviewer	Zumba?
ADL05	Yeah, I wanted to go there, and they were just saying like, if you have been active before then you can still do that kind of stuff, but if you haven't then you shouldn't so. I haven't been active at all "so I was like okay I'm just going to wait" so I don't worry about the baby.
Interviewer	Oh that'll be fun though. I tried Zumba once, it didn't go well. (both laugh) Haha I'm too clumsy I fell over the coffee table. Haha. But it, it looks fun. Haha. Um are there any particular sites that you remember whenever you search that kind of always come up?
ADL05	Um, babycensor.
Interviewer	Any others?
ADL05	No
Interviewer	Um and how often do you think that you visited websites about questions that you had?
ADL05	Um, about 20 times.
Interviewer	Over the course of your entire pregnancy?
ADL05	Mmm, probably.
Interviewer	Okay, um and you said that Facebook wasn't working. Okay so um did you ever lose access to your cellphone during your pregnancy?
ADL05	No.
Interviewer	Okay, um and is it your own phone or do you share it with anyone else?
ADL05	My own phone.
Interviewer	Okay, and how often would you say that you read the text messages that were sent during pregnancy?
ADL05	Um whenever it was texted.
Interviewer	Okay so you probably looked at all of the messages?

ADL05	Yeah
Interviewer	Um, and were there any messages that had the biggest effect on your pregnancy?
ADL05	Mmm, I'm not sure I don't have that good memory of the text messages. I know I still got them on my phone so I can probably just go back and look at them.
Interviewer	Okay. If you want you're more than welcome to look at them.
ADL05	Okay (laughs). Oh yeah I liked this one, um the good posture because, uh, I always had kinda back pain though.
Interviewer	Anything else?
ADL05	I know I looked at the one about the fruits and vegetables, and they do go bad quickly and they do, I was just reading that and I was like "wow, cuz every time I'd buy grapes like it'd be like 3 days, 4 days later it starts turning brown, or anything like that". But I was thinking about, you can't really buy I don't think grapes frozen, but other fruit, would it be the same though would it taste the same? I was kinda thinking about that like hmm? Would it try it? I 'm not sure, if it's smarter because it does go bad quick.
Interviewer	You can actually freeze grapes when you get them, if you like to chew ice, you can just chew them. Yeah you should try it. They're good especially now that it's so humid out. Yeah but you're right some fruit freezes a little weird but frozen pineapple is pretty good I like that, and some berries are okay but better mixed in things they get a little mushy. So were any messages you didn't like or made you uncomfortable or anything like that?
ADL05	No
Interviewer	Did you ever feel inspired to try any of the ideas that were written in the messages?
ADL05	Um, I guess to go exercise. I mean I haven't done it, but I've thought about it, you know and it makes you want to.
Interviewer	Um, what do you think would push you past that point of thinking about it and actually trying something? Is there anything specific? I know that's kind of a hard question.
ADL05	Probably if this if it, the baby wasn't coming, and I had to walk and stuff like that. It's just that when I walk I get cramps and it's just like I don't want to walk anymore.
Interviewer	Yeah, um, does it help to have someone to do things with? Like if they came over and said "hey let's go do this"

ADL05	Yeah probably.
Interviewer	Um, were there any other things that you tried or things that you thought about trying from the messages?
ADL05	Mm, I know the fruits and vegetables, I mean I'm gonna to try those regardless, I like fruits and vegetables. Um, probably I think like when you like if you, like if you texted the orange carrots, I'd want to get carrots because it's just in my mind now, like. Stuff like that (child makes noise in background).
Interviewer	Um, did any of the messages influence how you feel, felt about weight gain during pregnancy?
ADL05	Um, no not really.
Interviewer	Okay, and I, I know from your first interview that you had mentioned about you were worried about gaining weight because of the difficulties of losing it afterwards, are you still having those feelings or have those changed at all?
ADL05	Um I'm probably just dealing with it, cause I know that you have to gain weight (Interviewer: Mhm) so, I'm just going to see how it is after really.
Interviewer	Are you comfortable with the amount that you gained during this pregnancy?
ADL05	Um, uh it's just I can feel the weight now. Like the first pregnancy I couldn't feel it, like now you know my ankles are getting swollen my feet everything, I feel heavy. So it's harder now than before.
Interviewer	Do you think you gained more this pregnancy than the last one?
ADL05	I think I will through, through the whole thing yeah.
Interviewer	A lot more or just a little more?
ADL05	Probably just a little more
Interviewer	Okay, and do you have a plan after you deliver to lose the weight?
ADL05	Um, I wanna, I wanna treadmill that's what I want. I want that or I'm just gonna to have to go to the Y.
Interviewer	Um and did any of the messages change how you felt about certain foods that you were eating?
ADL05	Mmm, probably just better cause I still eat you know healthy vegetables and fruits.
Interviewer	Mhm. And have you changed how you prepare foods at all?
ADL05	Mmm no.
Interviewer	Umm and did the messages influence how often you take prenatal vitamins?
ADL05	No.

Interviewer	How have, how's that been going? I know sometimes they're so huge they're really hard to swallow.
ADL05	I know I haven't been taking them. I don't take pills at all really good at all. So I haven't tooken them. I just got a new prescription today so we'll see how that goes.
Interviewer	Is that one for the iron pills?
ADL05	Um it's not for the iron but it should be smaller prenatal pills.
Interviewer	Oh okay. Have you ever tried crushing them, and sprinkling them into cereal or anything?
ADL05	No
Interviewer	I know sometimes that can help. Um let's see so you said that a lot of the messages made you think more about being physically active um but that you didn't necessarily act on them. Was there anything where you clicked on a link and you watched a video about exercise or anything like that.
ADL05	Well I couldn't just because like I don't have internet on this phone, unless I'm at wifi, but if I was at wifi I would see it but probably not all the time.
Interviewer	Okay and anytime you did have wifi, did you ever click on the link whether it was about physical activity or food or weight gain.
ADL05	Um I'm not sure. I'm not sure.
Interviewer	Okay. Have you heard anything about what diet and exercise can do for your baby?
ADL05	mm I think I heard that the baby would be stronger,
Interviewer	Anything else?
ADL05	Not really.
Interviewer	Um if you had to rate on a scale of 1-10 where 1 is not helpful at all and 10 is really helpful um how helpful would you say that being able to use your phone to look up health information is, on that scale.
ADL05	I would say an 8.
Interviewer	Okay, um have you ever shared any of the information in text message, or from different prenatal websites like baby center with any friends or family?
ADL05	Um no.
Interviewer	Okay. Um and apart from um maybe checking the baby center website have you joined any other text message, phone apps, or um online programs about pregnancy?

ADL05	Um, yeah. One is called pregnancy haha.
Interviewer	And is that one a weekly or daily one?
ADL05	Um weekly.
Interviewer	Okay. And did you sign up for any email list serves?
ADL05	Um no.
Interviewer	What about um, following any blogs, twitter, facebook groups.
ADL05	No.
Interviewer	Okay. Um what was most helpful about your pregnancy app? I mean what did you like about that one?
ADL05	It teaches you about like stuff that can be wrong, like uh, if your veins are bulging out or something like that. Like it'll tell you things to look out for and warning signs of things that could go wrong.
Interviewer	Um and based on your experiences is there a place or person that has provided you with the best information on pregnancy?
ADL05	Um, is that a person or a place?
Interviewer	Mhm. And the place could be an internet site if.
ADL05	Um nah they really all say the same things. So. Yeah.
Interviewer	Yeah, um how did you feel about the frequency or the number of messages that you received each week from the study, was it too much? Not enough?
ADL05	I think it was a okay amount probably just like if it was the morning I'd be like "ahhh" hahaha.
Interviewer	So in the afternoon is best (both laugh). Um would you change the amount of messages at all?
ADL05	Um, probably.
Interviewer	How would you change it?
ADL05	Hmm, um probably to once a week, cause I know like how it is, like 34 weeks that's when you tell you all the information, like that Monday they'll tell you all the information, but you can like look up each day, or I can't explain it, it's like they text it to your phone once a week, but it's a lot of information, you know. So if you wanna read it throughout the week you have all you need to read throughout the week so.
Interviewer	So you don't mind if it's longer sets of information so you'll just come back to it if you had time?
ADL05	yeah

Interviewer	Do you wish some of the messages had been longer? Or cuz I guess since a lot of the messages went from the short and simple where you if you only get 160 characters for a text that's all that you got for that day.
ADL05	I mean it was it's good though. Mean it's you know, you'll sleep like a baby if you don't have caffeine, that's a good hint like a good something to know you know. I think it's good though. It's it all depends on the person, like. Everybody doesn't want to read a long you know text, so I think what you guys do is good though.
Interviewer	Do you think that during pregnancy, that's a time where women are willing to read longer types of information?
ADL05	Yeah. Yeah.
Interviewer	Um and do you think it would be helpful if it was the healthcare provider or midwives that were sending these sorts of updates, so that you know it tracked with your pregnancy or how to do specific things about the clinic?
ADL05	Um I think that would've been good too.
Interviewer	Um, and I know that the Facebook thing didn't really work out but would you recommend text message or Facebook or some other sort of social media as a better way of sending messages about prenantal care?
ADL05	I would say probably Facebook is probably best, which I didn't get it, but I know that if you wanna go look it's usually on the internet so if you're on Facebook and you see you know, pregnancy you're going to look at it. So I think that's, good.
Interviewer	Um better than text message?
ADL05	Well it all depends on if you have internet though. Cuz like I don't have internet so it's better for me to have text message so, but if you have internet then um it's probably best for people to have internet.
Interviewer	And does your text messaging, is it included in your plan, so you don't have to pay per text message?
ADL05	Yeah.
Interviewer	Okay, and are there any other mobile apps or um internet sites that you have thought of that you've used that you maybe hadn't mentioned so far?

ADL05	Um I got another which is "I'm expecting" it's just I don't even, I have to be on the internet to see everything, but I use it sometimes.
Interviewer	But the pregnancy app you don't have to have the internet for?
ADL05	No
Interviewer	Okay that's nice, um and then the "I'm expecting" is that a weekly update as well?
ADL05	Yeah.
Interviewer	Um is there anything else we talked about that you didn't get to say everything or you thought of something later that you want to go back and add?
ADL05	No.
Interviewer	Okay, um well that is all that I had to ask so do you have any questions for me or?
ADL05	No, not at this point haha.
Interviewer	Okay!

ADL06

Interviewer: So It's been a while since we last talked, and it's always good to catch up, so um, can you just tell me a little about how your pregnancy has progressed from the last time we talked until now?
ADL06: Sure
Interviewer: Alright.
ADL06: I think we spoke maybe six to eight weeks ago, I'm not sure.
Interviewer: I think so.
ADL06: Um, things have been going well. I feel probably the best in my third trimester than I felt all pregnancy surprisingly, and um, I feel, you know, excited maybe, so enjoying and preparing.
Interviewer: Ah, what would you say has been the best part of your pregnancy?
ADL06: Just getting pregnant because it, we struggled in fertility. So, just kind of that slow process of really realizing that this is actually real.
Interviewer: That's completely exciting.
ADL06: It is.
Interviewer: And last time we talked, I asked you what your hopes and dreams were for your baby and I just want you to touch on that again.
ADL06: Um, just to be healthy and have a life where he or she gets to do something they enjoy and find happiness.
Interviewer: Those are good goals.
ADL06: Yeah, I think so.
Interviewer: Um, so kind of changing gears, have you um, changed the way that you have been preparing food since we last talked?
ADL06: I don't think that I have, you know I've pretty much still maintained my philosophy of fresh ingredients, low fat, you know and you know, when possible, organic.
Interviewer: Um, what has your dairy food consumption been like?
ADL06: Overall, it has been on the low end because I'm lactose intolerant. I can do yogurts or hard cheeses, but um sour cream, cream cheese, milk, things like that tend to upset my stomach a bit.
Interviewer: Ok, and did that change at all during pregnancy?
ADL06: It didn't, I thought maybe it would. I heard that it can.
Interviewer: Yeah.
ADL06: It did not.
Interviewer: Ok. Um, and were there anything, so, it sounds like you generally try to make healthy choices in how you prepare things, um, but, did you find any differences in maybe you were craving fried foods or anything like that?
ADL06: Frankly, I was craving more spicy things but they tend to upset my acid reflux even more so Mexican food or spicy food like tacos always sounded good, but usually would keep me up at night.
Interviewer: um and were there any smells you liked more or less in the last couple weeks?
ADL06: Onions are more appealing to me now than they were. I couldn't even be in the house when my husband was like trying to cut them up or put them in something; it was just really offensive to me.

Interviewer: and when you say, um, earlier, that they were more offensive, did you mean the beginning of pregnancy?
ADL06: Um, first and second trimester.
Interviewer: Ok. And then have you had any cravings? I know you mentioned Doritos…
ADL06: …I know that's so awful. I'm ashamed of that. I tried to buy a healthier version and it just did not satisfy.
Interviewer: Have you had any cravings for any other foods?
ADL06: Um, more salty than sweet. I would say chips and salsa kind of thing. Whether it's Doritos or even like a tortilla chip. It's been sort of a standard. Even when I… early on when I was vomiting. I would vomit and say like "I really want some chips and salsa!" and my husband would say "I don't understand… you just threw up and you don't want apple sauce or toast? "No, I want Doritos!"
Interviewer: Um, and did you have any cravings for anything that was a non food item like dirt, um, baby powder?
ADL06: Never.
Interviewer: Ok. Anything you felt was like a real challenge with food during pregnancy?
ADL06: Um, getting meat in. I'm not a big meat eater like red meat. So, um, been trying to eat red meat, because you know it is a good source of protein. But I've just, I've never been a big red meat person. So, and the other challenge would just be the acid reflux that's kind of kicked up in the third trimester. It's made it hard to eat at some points.
Interviewer: Um, have you found that anything that worked for helping you fit in red meat or an then anything also helping with the acid reflux?
ADL06: Um, for the acid reflux I ended up going on Pepsid from my midwife. It helped sometimes, but it does not really take it away. I've tried like everything over the counter that's safe. So I was just enduring it or avoiding things that I know were going to cause it although it's not predictable necessarily, um, I like sparkling water and I can't drink that at all because it may be irritating to my stomach. You know like those, not the ones with aspartame in them, but you know just the sparkling flavored water?
Interviewer: Yeah. Like the…
ADL06: I can't… Poland Springs or whatever. I can't have any of that. It's just like every time I do, it just hurts a lot.
Interviewer: That is really bubbly.
ADL06: It is. Yeah, I even tried like stirring it, shaking it a little to get some of the bubbles out. Nope. In terms of the meat, I think it's just harder for me if it's like a piece of steak. I would really much rather have it cut up and put in something like meat in a salad or in a wrap or something like that a little more palatable.
Interviewer: Uh, and I always preface this question with it being loaded and it's more a lack of my ability to come up with a better way to word it, but…
ADL06: It's okay.
Interviewer: … Um, ah, I think a lot of women make conscious decisions during their pregnancy because they feel the choices they make will be healthier for them or for the baby…
ADL06: Yes.
Interviewer: and so I'm just curious what things throughout pregnancy you did um, for diet or exercise to make sure you had a healthy baby?

ADL06: I feel guilty that I had such a hard time for like the first eighteen weeks eating. I feel guilty a lot that I ate just like crackers for five months and I don't know what nutritional value that was for my poor baby, so, um I really made a very conscious effort to think if I literally had this child here would I really feed this, would I theoretically feed that food to this baby? Doritos? No I wouldn't. Even though you see it all everywhere. So that's been sort of how I make my decisions and just, I mean that you have to just have a little treat here and there I guess, but just basically I'm thinking about the nutritional value of everything that I eat. Whether this has fiber or this has particular vitamin or this mineral or this has protein, things of that nature and I feel like I went on a tangent and didn't answer your question.
Interviewer: I think that it did. Um, so, were there any things in terms of um, I don't… you can call it physical activity, exercise, or anything like that, that you've thought about during pregnancy that you've heard would be healthier?
ADL06: Um, I've done my best to at least maintain the level of activity I did before which included yoga at home. I did not go to classes like I used to, but um because I'm a nurse, I have a very physical job so for me to come home and exercise at night because it used all of my energy just up and down and move my patients around all day.
Interviewer: That makes sense.
ADL06: So I think of that as a kind of activity. I don't have a desk job and my husband and I try to go to walks in the evening. I've heard you know swimming is healthy, but I never did swim, not even once in this pregnancy. I have to find someone with a pool so I can just float in it. But, I just, getting your heart rate up a little and stretching and things of that nature I've been told are good for the baby.
Interviewer: Um, and as you learned things about how to prepare for the baby, are there certain places that you've gone to look for advice or questions?
ADL06: Um, I think I've used the Internet as a tool when I'm not certain. Not really too many books necessarily because those tend to be out of date by the time you read them and I don't know I think I came into this pregnancy with a lot of knowledge just being a nurse and having wanted this pregnancy for a long time. I, there was a lot of forethought in decisions about being pregnant and raising a baby and so there weren't a lot of things I felt uncertain about but I also, you know, would call a midwife about questions that I had.
Interviewer: You mentioned that you do use the Internet for information, how do you usually access the Internet?
ADL06: from my phone primarily.
Interviewer: and do you also have a home computer?
ADL06: I do. I have a laptop as well.
Interviewer: What, um, Internet at home?
ADL06: yes.
Interviewer: ok. And I know it's hard to think back to this but can you think of any of the things that you've searched for online?
ADL06: My husband and I disagreeing of whether or not a medicine or food was safe we would look online for it . Um, He was very involved in a good way, very nervous as well, so. I remember the first time I wanted to take Tylenol and he would be like "you can't take that." And I was like "No, I know I can" "We'll have to look it up."
Interviewer: are there any sites that you found that frequently pop up when you did do searches that seem to be more helpful than others?

ADL06: It's hard to find real information because a lot of it seems to be online forums where women are giving more of an opinion and I think being a nurse makes me question the validity of the answers that people post so um, I consider myself a little bit more internet savvy than a lot of people so I was looking for more academic articles or websites that had edu attached to them, or something by a name that I would recognize and I didn't go to thebump.com for information.
Interviewer: and how often do you think that you did do searches online?
ADL06: um, maybe once a month.
Interviewer: Ok. And how often did you use the Facebook site from the study?
ADL06: um, not very frequently. I think I looked at it like a handful of times especially when I first got onto it. I think I looked back at all the postings.
Interviewer: and do you frequent… use Facebook just for personal use?
ADL06: yeah.
Interviewer: Ok. Um, so overall, I guess on a scale of 1-10 where 1 was not helpful at all and 10 helpful, how would you rate the usefulness of the information that was provided on the Facebook site?
ADL06: Um, I would say maybe like 5. I think as I stated before, because I have a lot of fore knowledge in sort of health and pregnancy, a lot of what was, the information that was given to me, I already knew. Um they were nice reminders like your vitamin D and I was like ooh, does my prenatal have vitamin D in it? And I went and looked, but some of it was just stuff that I'm already aware of. So I can see how in general it would be great for the majority of people who don't have a health background.
Interviewer: That makes sense. Um, of all the things that you can remember seeing on the Facebook site, what would you say that was your favorite and least favorite parts were?
ADL06: Um, I made the smoothie that you posted and then I loved it and I make it all the time and I make it for my family and um made it for an ill relative who was like "this is so good, nothing tastes good and this is great!"
Interviewer: well that's good.
ADL06: so that was nice. Um, I don't know what I liked the least helpful. I think we got one yesterday or the day before that was like drugs and alcohol are bad for your baby and I was like yeah no crap. We know that. But yeah you know, if maybe you're like 15 maybe you don't really know that so I understand that you have to address the community that you're talking to.
Interviewer: that makes sense. Um, so you said that you maybe looked a lot more in the beginning and maybe all at once kind of scrolling back to things were posted. Um, as things were posted more frequently what motivated you to look at those new messages or demotivated you to kind of keep going back.

ADL06: Um, just the distractions of late pregnancy kind of.
Interviewer: mhm,
ADL: a perpetual to do list of things to worry about and get ready as opposed to mid pregnancy where I was kind of feeling like well I don't need to wash baby clothes yet or I don't need to fill out FMLA paperwork for my job or you know those kinds of things. So, time I guess.
Interviewer: mhm, and when you were on Facebook and you saw more recent messages was it just because it popped up in your newsfeed or did you actually go to the study page?

ADL06: They didn't pop up in my newsfeed I was noticing actually so I didn't know if that was something that I needed to do to make them pop up but I um, think a couple of text messages ago I realized that I haven't' looked at it in a while and went back and just took a peek.
Interviewer: ok , um and of the topics that were covered in messages, was there a certain topic that you felt that was most relevant to your interests or your pregnancy and your needs during pregnancy?
ADL06: I think about food intake were most interesting as opposed to lifestyle choice or exercise. Again that's because that's' been a focus of my own during this pregnancy
Interviewer: Ok, and did you change any of your behaviors after seeing any of the posts to Facebook?
ADL06: I wouldn't say so other than making smoothies.
Interviewer: Were there any other things that you recall, um, trying that you saw like a recipe or watching a video or anything like that?
ADL06: I linked to the playlist that you posted. I was playing it and my husband was like what is this? Oh this is a playlist for being active. Like ok.
Interviewer: did you ever feel that you disagreed with any of the information that you received?
ADL06: No.
Interviewer: Ok and in terms of weight gain I know for a lot of women, weigh gain just in general is a tough thing. So pregnancy I think can be... some peoples embrace it because they know its' good and other people are you know can reach a point were it's too much, um how did you feel about weight gain during your pregnancy.
ADL06: Um, very conscious of it. Um in the beginning because I lost weight, I was worried that I wasn't gaining what I should and then I had in my sixth month I gained a ton of weight and then I've slowly been gaining weight ever since. So just would call myself subconscious of it and I've spoke with a midwife a few times just because I did gain weight trying to get pregnant with my fertility treatment so, I'm about 40 pounds more than I'm comfortable being and so I'm just I have been worried about overall weight gain in terms of my long term health so just trying to be conscious not obsessive or your know, but conscious of it.
Interviewer: So in terms of how much you've gained during this pregnancy, so you feel that it's a good amount, is there range that you're trying to be in or…
ADL06: I do. Originally, the midwife said that I should aim for about 20 pounds; I'm at 22 right now. I'm not going to not eat obviously; it is what it is I have no idea what's going to happen in the next three weeks. I would gain ten pounds. You know just having an awareness of it and making conscious decisions you know but at the same time, It's hard to discern what the baby's needs are versus your own needs versus the cravings. So, It's going to be what it's going to be so then I'll just have to do the hard work afterwards, so, you know?
Interviewer: um, so there were a few messages on Facebook and text messages that discussed some sort of topics in relation to weight gain, were those ever ones that stuck out to you?
ADL06: I can't say that I remember them so I don't think that they did.
Interviewer: Ok that's fine. I know some people too, they worry a lot more about things and it seems like you're just taking things in strides

ADL06: I'm doing what I can and just recognizing this is a unique situation. Um I've struggled with my weight with most of my adult of my adult life and my older sister put on a lot of weight in her pregnancies and never lost it so I worry about that. I want to be a healthy mom and I want to be a good example of health for this baby and I don't think being 40 pounds over weight is a good example. So you know, its' just on my mind so. And my husband's put on a little bit of weight too. So he tried to put on his bathing suit the other day and was like ughh did you shrink this and I was like no honey, I didn't shrink it…
Interviewer: knowing that that fabric is not shrinkable.
ADL06: Yeah exactly. So um you know we are both aware of the work we'll have to do as a family to be fit afterwards.
Interviewer: It sounds like a good team of working together so that's good…
ADL06: I think that we are. I think it's funny that he thinks I shrunk his bathing suit though. You can't shrink that.
Interviewer: sounds like a typical guy.
ADL06: Yeah I know.
Interviewer: Let's see… so I know you said that a lot of the messages that have been the most of most interest to you but were there any that you you recall inspiring you to go for a walk or kind of I know a couple of video links.
ADL06: Sure, I'm not sure that I got the video links. Sorry, I have a big smudge on my glasses? I think that the one we got that was like you know taking a walk every day did inspire me to have a conversation. I don't know if it inspired me to walk more but at least it inspired me to say to my husband you know I should really be walking more. And uh, so every once in a while he'll refer back to that and say remember when you said we should be walking more and were watching Top Chef instead that maybe be should get off of the couch. So it's affected us a bit. It's motivated him to motivate me, I guess.
Interviewer: oh, that's great. Sometimes I think we need that.
ADL06: Indeed.
Interviewer: and kind of along those lines, I know a lot of women say that as they get further along in their pregnancy they feel more fatigued and…
ADL06: Definitely. Yes.
Interviewer: so that's something that you've been seeing?
ADL06: Definitely, yeah.
Interviewer: um so I always find it interesting to talk about what motivates you to make yourself get up and go for a walk or eat that healthier snack choice when you just don't feel that great.
ADL06: Uh, my husband's definitely a factor in that. When I say like let's go get ice cream sundaes and he's says like umm let's go get some fruit. So you know he's well aware of my feelings about excessive weight gain and health of the baby and so and obviously he's invested in the success of it too. So we motivate each other because sometimes he's the one being let's go get an ice cream sundae. So I think he's my primary motivation in making healthy decision you know when I'm feeling fatigued and its 100 degrees and I just want to lie on the couch.
Interviewer: Yeah, It's going to be really bad tonight.
ADL06: Yeah, I'm not going for a walk tonight.
Interviewer: Yeah, that's probably a good idea.

ADL06: No, we have the kitty pool that the dog likes to go in and I'm going to put my feet in it. That's my plan.
Interviewer: That will be nice.
ADL06: Yeah.
Interviewer: Um, so during pregnancy switching kind of to cell phone use, um, did you have the same cell phone through the whole time that you've been in the study?
ADL06: Yes.
Interviewer: and you've had constant access?
ADL06: yes, I know that's a an issue for some people.
Interviewer: and let's see um, how often do you think you read the text messages that were sent?
ADL06: um, every time they were sent.
Interviewer: Ok. And would you say that any of the text messages had a bigger effect on your pregnancy than others?
ADL06: mmmm, no. I wouldn't say so. No.
Interviewer: I know too, it's hard to remember which came from which so if we already covered it with the Facebook stuff, that's fine too.
ADL06: yeah. There's some overlap sometimes in your mind.
Interviewer: yes. And we sent a lot so it would be hard to remember them all. Let's see. Do you think there was a difference between receiving messages on Facebook and through text messages in terms of I guess the likelihood that you would use the information?
ADL06: umm I'm not as Facebook addicted as some other people I probably check it once a day, so if that once a day was in the evening when I was already tired, I probably just skimmed it where as a text message, my phone alerts me and says hey you have a message and so I think the texts were probably a little bit more effective in terms of me reading them and having the information available at that moment.
Interviewer: um did any of the messages change how you felt about specific foods or get you to try something new that you haven't had before?
ADL06: well we did try the smoothies. Not that I've haven't made smoothies before just for some reason on that particular day it just was like oh, I have all the ingredients and it sounds good.
Interviewer: that works out well.
ADL06: yeah.
Interviewer: um and when you do your Internet searches with questions is it usually, has it usually been on your phone or more on your laptop?
ADL06: more on the laptop at home
Interviewer: ok. Have you ever shared any of the information that you received with anyone other than your husband?
ADL06: Yes, I have a cousin whose expecting a baby 4 weeks after us and I originally shared with her that I was in the study and she wanted to be in it , um but, I made her a smoothie when she was at my house and I gave her the recipe.
Interviewer: um and have you joined any other email, text messages, twitter, blog, or Facebook groups about pregnancy?
ADL06: No I have not.
Interviewer: Ok and what about phone apps?

ADL06: yes. I think its called Pregnancy… I have to look… it's not a very intellectual program but it's Pregnancy Plus, health and pregnancy. Basically it has a count down tells you how far you are into your pregnancy, has like a daily tip, and then a weekly tip and then a weekly kind of a like this is what's happening with your baby this week. So. It's been more entertaining then informing you know just kind of just something to check out. There have been a few things on there that have said like oh, you should probably pack your bags this week and we're like oh! We should pack our bag! You should probably have your birth plan written, things like that, so just a set of reminders.
Interviewer: that's nice.
ADL06: yeah.
Interviewer: that's helpful. Um ok, so based on all of your experiences so far, would you, well which person or place do you feel has given you the best information on prenatal care?
ADL06: um, I mean I would say the midwives, you know I'm really confortable with them specifically Helene. And the nurses, the triage nurses are really lovely too. They're obviously used to talking with overwhelmed or panicked people. They're really calm.
Interviewer: that's good and since you did Facebook and text messages, you receive anywhere from two text messages a day anywhere from 6 days a week up to I know more recently It's been um, a little less. Um, is there I mean so you have a nice range to gauge things and you can compare to the pregnancy app, but is there an optimal number of messages that you think would be good to send?
ADL06: umm, I'm not sure if I ever got two a day…
Interviewer: I guess it would have been the one on Facebook.
ADL06: oh, ok, ok. Um, I don't know. It certainly there's no polite way to say this. It wasn't like an annoying amount. It wasn't like ugh this person's texting me…
Interviewer: That's not really what we're going for.
ADL06: yeah, I figured. And I think because they were very. They were written with a very specific amount of information: not overwhelming, not too long like some you could glace at even if it had been twice a day, I don't think it would have been annoying.
Interviewer: ok.
ADL06: it was presented well. So I think it was adequate or whatever word you want to choose.
Interviewer: ok. and did any of the way the words were written, the wording, were there any that they were worded in a way that was demeaning or made you feel uncomfortable at all?
ADL06: No, I thought that they were well written. You know and I could see, I chuckled about the drug and alcohol one, but I can see how that would be a hard one to write and I think it went, came off just fine.
Interviewer: that's always something that we worry about becaue it is a hard topic to touch on…
ADL06: it is a challenge to address that issue to address that issue in a way that's not offensive to people even though that are doing that, it's a really terrible decision. You know so without sounding critical I guess. No, I think it came off nicely.
Interviewer: ok, that's good to know. And so a program like this would this be something that you feel a health care facility should provide to their pregnant women who seek care at their clinic?

ADL06: I think it would be great. Um, I don't know if you're familiar, but at my previous job they had a um.. I forget the word they used but it was like Group Visits almost, so they did it for, they did it for when babies were born in a group and they would pick like six families and they would all come as one as if it's together and give a chance to chat and mingle. Which I think if they did prenatal care especially for teens in that kind of setting, this might be a nice way for people to interact and possibly connect as well. I don't know that it's necessary for every group of people, but I think it could be valuable for certain sets.
Interviewer: That makes sense. And if a heath care provider was the one facilitating sending messages and maybe even having this group care idea that you mentioned, is there a certain type of information that you feel would be most important to emphasize in this?
ADL06: that's very hard because it depends on who the clientele is and it depends on what kind of knowledge they're coming to the table with and you know, how open they are to information. So I know that I was sitting with a teen girl but we would have very different prospective about birthing and like a labor plan. They might not even have one and I'm kind of obsessed with mine. So I think it can be helpful but I think it would be, it would depend on the clients. You know? Some people I think might not have any interests in something along those lines, I'm not sure.
Interviewer: Yeah, It's probably very individualized. For you specifically what is the best motivator for you to make healthy decisions?
ADL06: The baby's future health and a healthy birth experience too. I feel really strongly about what kind of birth experience I want and setting myself up for that has been um, a goal during pregnancy.
Interviewer: and since one of our major goals for this study was to reach teens, it sounds like you have some experience working with teens at the pediatric clinic, do you have any advice of whether Facebook or text messages or some other sort of app program online would be the best way to reach them?
ADL06: I have to admit I'm not really cool enough to know what's cool, but I think texting is a major form of communication for most teens in fact like I have a 21 year old brother who 99% of the time will text me rather than call me so it's a generation thing. It's also cultural. It's also less expensive because some people have unlimited texting where they don't have the minutes. I think everything you guys did to make it, the information available was perfect. I can't think of anything to make it easier.
Interviewer: So I know that you said you know, that they don't show up for appointments and those sorts of things, are there anything that in your experience in working with them that in your experience motivates them to show up and be more engaged in their health?
ADL06: Yeah its' going to sound terrible but food, money, and transportation. So if you provide people with transportation because that's a huge issue. So if you can feed them or provide them with some compensation, that's going to be motivators. Unfortunately, it's those are baseline motivators for most people anyways. Um, I don't know. And getting their friends involved, not that you want to encourage like teens to get their, have their friends get pregnant, but I don't know too much about whether or not there's support for teens who are pregnant within the community but creating friendship within that group might be a motivator because if their friend is going to show up, they might show up.
Interviewer: that's true. Have you ever felt like a lot of them seemed closed off in like kind of the world is kind of against them?

ADL06: Yes. Part of it is culture. The culture coming in from, um, for me I had an opening by saying I went to city schools that I grew up in the city that broke down some barriers for them and you know to hear I don't come hear from the greatest family background so to be able to share some of those things about myself like a father who wasn't there or a mother who had issues um kind of allowed them to parallel themselves with me more than let's say that if I shared that I grew up in Pittsburgh and went to a private school, you know. So having common ground you know and I think that frequently they're feeling judged all around anything and so those walls are going to be even higher than. And I'll through it out there, there are racial and culture elements that I cannot break down. I don't speak Spanish so I can break that down. I'm white, so I can't break that down, um, so I have to use what I can which is my own experience to say yeah, I went to school 34 where did you go? Or things like that. And it takes a long, long time to create a trust relationship and unfortunately in a study setting that might not be enough time to get that information.
Interviewer: That makes sense. Right that definitely a challenge that we've had
ADL06: Yeah I'm sure.
Interviewer: And we have offered prizes and things like that and they just still don't' do it. I think its jus.
ADL06: Going to them might help I mean I don't know if you want to walk into peoples homes but going to them might be another option.
Interviewer: That's hard because a lot of them are so transient and getting them to even responding but yeah definitely being flexible in our ability to find them
ADL06: yes, yes, yes, and if they are in a school you know going to their school or you know things like that
Interviewer: That makes sense
ADL06: Yeah it's tough, really tough on so many levels. So many barriers, so
Interviewer: Um well those are all of the questions that I have is there anything that we talked about earlier that you though of an additional response to that we can go back to before we wrap up?
ADL06: I think it would be fun if you were to do this again to have almost a cooking class? Kind of like an opportunity to, for people to maybe try healthy foods or healthy cooking styles maybe? Um I don't know. I think it might be fun. I couldn't make it to the group. I know you had two group sessions and I was working both times. I don't know what that format was but it would be interesting if people had the chance, to kind of, especially people who might not have access to the freshest ingredients to make their own smoothie or something like that. It might be interesting to them.
Interviewer: Yeah, thank you that's helpful
ADL06: I think that's it that I can think of.
Interviewer: Ok, yeah.

ADL07

Part 1

(Skype Ringtone)

ADL07:…(background noise)…Hello!

Interviewer: Hi! How are you?

ADL07: I'm okay, how are you doing?

Interviewer: not too bad.

ADL07: it looks like you may have gotten married recently…(laughs).

Interviewer:…(laughs)…I did…(laughs)…on Saturday.

ADL07: congratulations!

Interviewer:…(laughs)…so, yup, it's been a whirlwind, so I'm sorry if you haven't received quite as many messages as usual in the last week.

ADL07: that's OK, I'm kind of impressed that you're doing this right now…(laughs)…

Interviewer:…(laughs)…actually my husband, I just drove him to the airport he has a…ehm…a trip for work so…

ADL07: oh no!

Interviewer: it works out pretty well, and we were gonna wait for a honeymoon…um…until Christmas, so…

ADL07: ahh.

Interviewer: give me something to do, 'cause I don't really have anything to do, my stuff hasn't gotten here yet so I can't really unpack anything, so…ehm…it's nice…(laughs).

ADL07: alright!

Interviewer: so..umm…how have things been going with you?

ADL07: things are going okay, you know I'm definitely getting close to the end, and just… bigger…and more aches and pains and what not, but you know, not horrible I guess.

Interviewer: well that's good.

ADL07: a little bit of stress here and there, like panic that I'm not gonna be ready in time…

Interviewer:…(laughs)…I'm sure that you will…I think it all comes together in the end.

ADL07: hopefully. And everyone keeps reminding me the baby doesn't really need a whole lot of stuff…

Interviewer: that's true…

ADL07: but…

Interviewer: that's true…

ADL07: but I don't want to spend all my time post baby…you know I wanna like bond with baby, and sleep and stuff like that instead of be organizing and setting things up and running to the store so…trying to get as much done now as possible, and still work and I have a big project due on Thursday, so I'm kind of…I need to get through this week and then life will be okay…(laughs)…

Interviewer: well hopefully it all goes well and you get that done, and then hopefully feel a little less stressed after your…(incomprehensible)…is turned in…

ADL07: yeah…

Interviewer: ehm…so…I'll try not to keep you too long, ehm…

ADL07: awesome…

Interviewer: but…um…the goal of today is kind of similar to your first interview so we'll talk a little bit about how you've been eating and exercising…um…how those things have changed since early pregnancy and…um…the other goal is to talk to you about the messages that you received and…(background noise)…if there is anything that really stood out to you, if you disliked any…and…um…if you made any changes because of the messages. So, if that sounds good, then…

ADL07: yeah!

Interviewer: we can go ahead and get started.

ADL07: okay.

Interviewer: okay. Um…so, its been a little while since we talked so it's good to kind of catch up on how things are going and soo…just the first couple questions are…um…just really general…kind of how you think about your pregnancy, and…um…any goals that you

might have…um…so the first question I have for you is: so how has your pregnancy been going since early pregnancy?

ADL07: Um…I would say pretty good, especially compared with some other people that I've spoken with about their pregnancies, and I feel like overall I've been pretty healthy and able to do the things I wanna do, and I feel pretty good…(laughs)…so I think I'm pretty lucky.

Interviewer: that's good. Um…and what would you say has been the best part of pregnancy?

ADL07:…oh gosh…I don't know there are a lot, I mean it's just exciting, it's…you know…its…its been really fun just for my husband and I that…kind of be able to share this together, and I don't know we're really excited to…have a baby and…um…I think just the whole, you know pregnancy is just the anticipation for that…and…um…I don't know…it hasn't…it has been kind…you know there are certain ways where it has been kind of fun…like some people you..like… you talk to say: "oh my gosh, pregnancy is horrible and I hated every minute of it and I never want to be pregnant again;"…but there definitely has its ups and downs, and it's…again…as things progress it's getting more difficult but…overall it's kind of fun 'cause it's just a new experience and…you're preparing for another little person, so that's the best part I guess.

Interviewer: um…and last time we talked I asked you what your hopes and dreams were for your unborn child and…I was…just want you to answer that question again for me.

ADL07: my hopes and dreams for my unborn child…(sighs)…that's a big one. Um…(laughs)…just that they are born healthy and that they continue to be healthy throughout their lives, and that they're happy and…that's, that's the biggest thing, healthy and happy.

Interviewer: okay, so changing gears, um, let's talk a little bit about how you've been eating…

ADL07: um

Interviewer: have you changed any of the ways that you prepared food since…um…either before pregnancy or early pregnancy?

ADL07: um…a little bit I think. Early pregnancy was though because I just…I had no energy and no motivation, and the things that sounded good to me were kind of like high fat comfort foods…(laughs)…that I didn't normally eat a lot of…um…so I found that my eating habits had changed a bit and I feel like now, in the second and third trimesters I've had a little more energy and…a little more awareness to remind myself that: "oh yeah I need to make an effort to try and eat a little bit better and get more a variety of stuff and add vegetables back into my diet…(laughs)…and…um…its been hard 'cause my husband just started a business so he's working all the time, and we actually got his mother to help us

with some of the cooking we…'cause she's retired so we kind of pulled her in to help us make healthy meals so…we're trying!

Interviewer:…(incomprehensible)…and in terms of preparing food, have you changed how you prepare food, like choosing baking over frying or…

ADL07: um…

Interviewer: things like that…

ADL07: we were never big fryers to begin with so for me its really just been…um…you know I was reaching, in the beginning I was kind of reaching for convenience foods or I would let myself go out and get like get a grilled cheese sandwich from somewhere, and now I'm really trying to make sure that we're cooking our own food…um…and we usually like…you know…I usually like roast vegetables or steam them or eat them raw…um…normally anyways, so I think in that regard it hasn't really changed too much, it's just been more of a…kind…eat the whole foods that I used to eat…(laughs)….and prepare my own foods more than…than eating out or reaching for something that's a little more convenient, or like ordering pizza, or something like that….so.

Interviewer: um…and have you found that you…had more interest in eating salty or sugary foods?

ADL07: um…in the beginning definitely salty foods, and I feel like that's….that's kind of evened out a little bit and I've always had a bit of a sweet tooth, and I feel like my sweet tooth is kind of coming back a little more towards the end here…um…but yeah, I feel like my eating habits are kind of…trying to come back to baseline a little bit as I am getting to the end of my pregnancy, certainly in the third trimester.

Interviewer: um…what's your dairy food consumption been like across pregnancy?

(long silence interval)

(skype call activated sound)

ADL07: Oh sorry ok I've figured it out…(laughs)…my…my husband called…
10:37

Interviewer: ok

ADL07: and I clearly am…ehm…technologically challenged…(laughs)…and so I tried to decline his call but then it like put you on hold and I couldn't figure out how to get you back…(laughs)…sorry about that…(laughs)…

Interviewer: oh, okay…(laughs)…I was thinking…I wondered what happened… It says it's still going, but…

ADL07:…(laughs)...I mean, she's on hold but it wouldn't let me like get back to her, ok sorry about that…(laughs)…

Interviewer: no that's ok…um…so I think the last thing I asked you about was, um, what your dairy consumption has been like.

ADL07: my dairy consumption?

Interviewer: mhm…(agrees)

ADL07: I…was…not eating any dairy prior to getting pregnant actually…(laughs)…and my dairy consumption has gone up significantly since, that was like one of the things that I, like one of the only things I wanted in my early pregnancy days…(laughs)…was cheese, and then I basically eat yogurt every morning for breakfast…at this point soo…But I started out…I was, I've always been lactose intolerant, or for a while, so I usually don't eat tons of dairy and I found that also was, I think contributing to headaches…but apparently I don't have any issues now that I'm pregnant…(laughs)…so…

Interviewer: I've heard of that happening, it's really interesting how much your body changes

ADL07: mhm…(agrees)

Interviewer: um…have you had any cravings throughout your pregnancy?

ADL07: um…I haven't had any like, you know, wake me up at 2 o'clock in the morning and make my husband go out to the store and buy pickles and ice cream cravings but I do find that, not as much now but again like in the beginning I would see somebody eating something and I would think that it looked really good and then I would obsess over it for a while until I got to eat it and then it was kind of like: "okay I've eaten that now I can move on with my life," and then I'd find something else to obsess over…

Interviewer:…(laughs)…

ADL07: so, it was, it was very strange, like stuffed shells for instance, there was one like my co-worker was eating stuffed shells, I can't remember the last time I ate stuffed shells, and they just looked so good and I was like:" I need to eat those," so I made my mother in law, you know, cook some them me and then I ate stuffed shells for like a week…and grilled chee…grilled cheese sandwiches and pizza definitely popped into my head frequently…(laughs)…I would say, and again it's like all this dairy stuff that I didn't typically eat prior to pregnancy, so….that was a little different for me.

Interviewer: um…and have you had any cravings for non-food items like dirt, clay…um…

ADL07: no.

Interviewer: powders, okay.

Interviewer: and what about smells…are there, are there any smells that you've liked more or less with pregnancy?

ADL07: um…the biggest thing that I can think of is, like, coffee breath really offended me even more than normal…(laughs)…but I wasn't, I don't know that I was super sensitive smell wise…but I just, I remember like coffee breath was one of them and I just couldn't…I was really sensitive to and couldn't tolerate it at all…and as far as liking any smells…no…I don't think that changed.

Interviewer: okay…um…so this next question is…it's a loaded question and it's probably, could be worded much better so…

ADL07:…(laughs)…

Interviewer: um…I know and I'll just preface it with that. I think a lot of women when they become pregnant really try hard to make changes to be…

ADL07: mhm…(agrees)

Interviewer:…healthier, because they want their baby to be healthy…um…so along those lines the question is: "what things have you done throughout your pregnancy to make sure your baby is born healthy?"

ADL07: um…so I actually was surprised because I felt like I was eating better prior to being pregnant, and then I think which is with the hormones and…the…aversions to food, and strange things sounding good to me…um…I feel like my nutrition especially in the beginning was a little bit worse so I would like to say that I, you know, I thought I was gonna go into pregnancy and be this super healthy eating you know, salads every day kind of person…um…and that's gotten better as it's gone on, but I guess otherwise I've tried to increase my activity as is safe and recommended…um…definitely staying away from whatever toxins I make sure that…I try and eat like organic fruits and vegetables and I'm a little more careful now than I probably was before…um…I'm making sure that I don't take any…medications other than my prenatal vitamins, I think I've taken like 1 Benadryl…because I was desperate and they said that was okay…um…my husband won't let me paint…(laughs)...

Interviewer:…(laughs)…

ADL07: you know just anything that could possibly expose the baby to any kind of toxins that could harm it…is kind of been my big thing…um…and then just making sure, you know it's been hard to balance work and sleep and that stuff though, you know there have been times when I've kind of had to remember that I'm growing a little baby that needs to be healthy and that I need to maybe take a step back at times when I wouldn't…um…to make

sure I get enough sleep or to go ahead and allow myself that nap, or…um…you know say no to some kind of project or an extra shift or something like that that I would have normally taken on.

Interviewer: and over the course of pregnancy, whenever you had questions about anything…is there a place that you found most helpful for advice or information?

ADL07: um, it kind of depends on the question…obviously, you know, I've asked my midwives various questions…um…but I have, you know I have that book like "What to expect when you're expecting," which is been a little bit of a resource, and then also just online there are a bunch of resources, um, that I've used, as well as my friends who have gone through this before…have been a good resource…so, I don't know when it comes to certain things like: "are certain things safe for babies? Can I drink chamomile tea? Can I exercise?" You know those things sometimes I go to like multiple sources just because sometimes there is conflicting information or, you know, I'm not yet due to meet my, meet with my, met…excuse me…my midwife yet or something like that so I would sometime check things out online just to get an idea

Interviewer: um…and when you use the internet, how do you typically gain access to the internet?

ADL07: um…I have a laptop computer or I use my phone.

Interviewer: and you've mentioned tea and exercise, are there any other types of information that you specifically remember searching for on the internet?

ADL07: um…definitely, just like symptoms…um…

(skype disconnected)

Part 2

(skype ringtone)

ADL07: Hi!

Interviewer: Hi!

ADL07: oh, I'm not showing up am I?

Interviewer: um no…but you weren't before, I think…

ADL07: oh, really?

Interviewer: yeah my skype is sort of, it's been acting weird with video calls recently but the sound always works so…

ADL07: okay

Interviewer: I just go with it…(laughs)…

ADL07:…talk about that, I don't know what happened that time, it just spontaneously stopped and than it wouldn't let me reconnect to you, so…

Interviewer: yeah, mine did the same exact thing, so…but…maybe…

ADL07: okay.

Interviewer: someone had a blip in, in their net or something…

ADL07: who knows…(laughs)…

Interviewer:…(laughs)…yay technology!

ADL07: yeah, yay!

Interviewer:…um…

ADL07: so…(laughs)…

Interviewer: so I think we were talking about…um, oh information that you've looked for on the internet and the last thing I heard was that you mentioned that you searched for symptoms

ADL07: mhm…(agrees)…um…yes, so various symptoms of, you know, am I feeling, is what I'm feeling normal or, you know, that sort of thing…um…but again its been a while since I've looked anything up so…oh and I also looked up different...um…like prenatal exercises, that's a big one that I've looked up online, for like back stretches and stuff like that. And even… Infamil? had, I think actually they sent me like an email and it had some links to exercises, and they had this really cheesy, like step aerobic…

Interviewer:…(laughs)…

ADL07:…video, but it was good, I did it a couple of times and it is like they showed the various women in different stages, you know like the first trimester, third trimester, post partum women doing them, so it was just good to, you know, in a day when I needed like a 15 minute exercise and couldn't leave the house…(laughs)…

Interviewer: um…are there any sites that you thought were most helpful?

ADL07: um…I think the ones that are the most helpful are the ones that send me e-mails regularly…(laughs)…because it's kind of easy access…um…and so those were like, oh my gosh, I think it's "everyday family" is one of them and "fit pregnancy"…um…and I can't think what else…there was "the bump," but I mean that's more just kind of like discussion from other people, and so…you know, it's not the most…I didn't, I always took whatever I read with a grain of salt, you know…(laughs)…it's like…um…and then I think probably I stumbled upon like "webmd" or something a couple of times to look some things up...

Interviewer: how often would you say that you did internet searches?

ADL07:…um…probably in the first half of the pregnancy like multiple times a week…I don't know 2 or 3 times…um…and then now as things have progressed probably every couple of weeks, like once every couple of weeks or something.

Interviewer: um...earlier you mentioned that sometimes when you're searching online you find conflicting information…

ADL07: mhm…(agrees)

Interviewer:…so I am just curious how you go about deciding which sites have more accurate information and which things to believe or not believe.

ADL07:um…that's kind of tricky….(laughs)…but definitely, you know, anything that…I mean I guess a site like webmd probably has to back themselves up with actual medical knowledge and a basis for their information, so I feel like that, you know, might have better medical information…um...and then if I go to a bunch of different sites and they all say the same thing than that kind of might help me to feel a little more comfortable with the decision or…if I just don't know…then I'll just go to my midwives and ask them what they…you know I guess they are the ones that I have to fall back from trusting the most…'cause it's hard to know…I mean…with some, you know some of them like the everyday family one or the fit pregnancy you know they find studies that say such and such but it's always hard to know where really…they're getting their data from, so I guess if a lot of places are saying it…(laughs)…and if, you know, it's been a study, or a doctor that's kind of given the information, then I guess that helps maybe to feel a little bit more comfortable that it is accurate.

Interviewer: um…so the next thing I wanted to talk about were the facebook and text messages that you received…

ADL07:mhm…(agrees)

Interviewer: um…and so we can kind of lump those together 'cause I know sometimes it can be harder…

ADL07: okay

Interviewer: hard to remember if you received the message on facebook…

ADL07: oh…you froze…(sighs)

Interviewer: so we can just…if I say facebook just know that I mean, um, both places…um…but if you do remember specifically that, you know, one was from one place or the other, please feel free to share that…

(skype disconnects)

Part 3

ADL07: sorry, this is just like…(laughs)…oh you have a friend…(laughs)…

Interviewer: yes…(laughs)…you saw that I was done with my cup of coffee and was like, wow, mine mine…(laughs)

ADL07: …(laughs)…

Interviewer: um…

ADL07: okay, sorry about that.

Interviewer: oh no! It's okay…this is one of the…

ADL07: okay

Interviewer:…the perks of using skype..(laughs)…

ADL07:…(laughs)…we'll it's funny because like, I've talked to my sister, the really the only time I ever use is when my sister is in India, and I don't think we've, like, ever had a disconnection for some reasons despite the fact that she is in India and who knows what her internet connection is and…I don't know…so I find this kind of strange…(laughs)…

Interviewer: …(laughs)…

ADL07: we're not that far away from each other and I know my internet is okay, so I am like why is this not working…(laughs)…

Interviewer: yeah, I mean it could be ours, I don't know much about the internet here yet, so…um…it looks like it's good but who only knows…

ADL07: yeah

Interviewer: you never know with these things…um…so, I wanted to ask you, um, before we talked more about facebook and text messages…

ADL07: mhm…(agress)

Interviewer: how often you used the facebook site versus text message

ADL07: um…I guess the same, 'cause I mean it seems like frequently when I would get a text message then later on that day if I'd go on facebook I'd also have a message on facebook…

Interviewer: mhm…(agrees)

ADL07: though…I don't know, I mean I, you know, I visit text messaging and facebook frequently throughout the day.

Interviewer:mhm…(agrees)…um, and overall on a scale of 1 to 10 where, oh, 1 was "not helpful at all" and 10 was "very helpful," how would you rank the facebook site and the text messages?

ADL07: um…like as one, as we're combining them?

Interviewer: um, or you can…

ADL07: or

Interviewer: you can rate them together or separately, whichever is easier for you

ADL07: um…I'm gonna say like a 7 maybe…

Interviewer: okay. And can you tell me a little bit about why you would give it a 7?

ADL07: though I think they were definitely helpful and it was a good reminder, and especially on days when I was feeling…unmotivated…(laughs)…and you happen to send something like "oh! exercise is good for your baby," or "oh, here's some yoga poses you can do," or something…um…that was definitely very helpful, and the nutrition stuff as well, just as a remainder that is like "oh, no I'm not gonna reach for the pizza, I'm going to make a salad instead or get some veggies or something." So it was just nice to have, uhm, those gentle reminders…(laughs)…that it's like "oh, it's good for me and my baby." Um…and some tips, and the only thing I wish was that, like, I had gotten them during the first trimester. So…'cause I feel like kind of at the end now some of the information is becoming a little more redundant, but it's still good, I mean there are definitely still days when I'm like "oh, I don't want to take a walk," and then I'll get a little peppy text message, and it's like "oh, yeah I need to remember to do this kind of stuff it's good for me and baby," so…

Interviewer: um, and did you have a favorite part of the facebook and text messages?

ADL07: um…no, I don't know…I mean I definitely think that there were some that were just…you know I was able to incorporate more into my lifestyle or, you know there were some that I, some things that I didn't know already, and those were the ones obviously that were most helpful, or just kind of the ones that…um…you know, for me maybe the information I had heard it before but it was just like another reminder and so it was a good "get off your butt and get moving" motivation piece, so…

Interviewer: and on the flipside were there any things about the facebook and text messages that were your least favorite parts?

ADL07: um…gosh this is hard!

Interviewer:…(laughs)…

ADL07:…(laughs)…sorry…

Interviewer: no that's okay!

ADL07:…I'm really bad…(laughs)…um…'cause it's such, I mean it's such a simple thing, it's like, you know, the text message comes and if you're in the right place at the right time and the information's right you kind of take it, or if it's…you know, ehm, take a nap before I have to go to work, and I'm not paying attention it's kind of like "ehm, whatever"…um…but… I don't know, I mean nothing was…I wouldn't necessarily say like least favorite 'cause I'm sure it's all…even if I didn't find that like one specific tip of the day, if I had already heard it, or…um…you know, I know there was like the thing about pica, that's not really something I'm experienceing, so it's not really pertinent to me, like it's probably pertinent to someone else so…and still I'm sure it's a good thing to be aware of…

Interviewer: um, and what would you say motivated you to keep visiting the facebook page and reading the text messages?

ADL07: um, 'cause they would just appear there, it was really easy…(laughs)…but, you know, I mean I think that's the biggest thing is that, you know, when I would pick up my phone, "oh, there is the message, let's see what it is! Oh, this is…" you know, there's something new for today let's see!

Interviewer: um, and there were any topics that were covered that you felt were most relevant to you and your pregnancy?

ADL07: um…I think I appreciated the things about exercise or any tips on that and then just…um…you know there were a couple links for like healthy recipes…um…and just, you know again the reminders of making sure…I don't…I think, you know, for the most part a lot of it was relevant to pregnancy, you know it's relevant to everyone's pregnancy to eat healthy…um…and luckily for me its been a pretty healthy pregnancy and I've been able to exercise so that was relevant as well…um…so yeah, I think it was just, you know, the little

tips and the reminders and the “oh, yeah I need to make sure I’m getting my vitamins and, and…ehm…you know, here’s some healthy recipes I can try and stuff like that so…

Interviewer: um, and would you say that the messages influenced you to change any of your behaviors?

ADL07: um, I think it definitely helped to, you know, especially on those days when I was feeling not so motivated…(laughs)…um, that it definitely helped as just another kind of nudge to be like “oh, yep, this is worth I need to remember to put forth a little extra effort to make smart choices,” so, I think in that way, like it didn’t drastically changed my eating habits so much, but it definitely helped on a day-to-day basis to make sure that I was trying to make a better choice.

Interviewer: mhm…(agrees)…um, and have you ever shared any of the information that you received with friends, family or other pregnant women?

ADL07: that’s a good question! Um, not that I know of…(laughs)…

Interviewer: and, um, as you mentioned there can be a lot of conflicting information out there, were any of the messages that were sent something that you disagreed with?

ADL07: um, not that I can remember, I mean I think everything coincided with things that I have, you know, heard or read somewhere else, um, yeah no I don’t think, I can’t remember that there was anything that I disagreed with…

Interviewer: are you still there?

ADL07: I’m here

Interviewer: oh, okay…sorry just making sure…

ADL07:…(laughs)…it’s okay

Interviewer: it made a weird little blip noise, and I was like “uh oh”…(laughs)…um, and how did the messages influenced your feelings about weight gain during pregnancy?

ADL07: um…how did they influence my feelings about weight gain…it was definitely something, you know, that I’m aware of, obviously that’s something that you hear people talking about: “oh, I gained such and such amount of weight during pregnancy,” and you go in and, you know in the first visit, the doctor, midwife or whoever, recommends that you gain a certain amount, so it’s definitely something that you’re aware of, every time you go you get weighed, um, so I guess it was, you know, something in the back of my mind, and I knew that gaining a healthy, you know recommended weight was good for both me and baby, and um, I don’t know, I mean I guess with the, the messages and the tips and stuff it just kind of helped to make sure that hopefully I was staying on track with an appropriate weight gain. So I guess in that way I kind of used it to, again try and make, you know, better

food choices and hopefully by doing so I would gain the appropriate amount of weight at a nice steady pace…

Interviewer: um, and was weight gain something that you were concerned at all about…

ADL07: um…

Interviewer:…during pregnancy?

ADL07: not too much, I mean I knew going in that I was gonna gain weight, and, um, you know there were some, like…there were a couple of weeks when all of a sudden I gained, you know, 5 pounds, or 8 pounds or something in 4 weeks and I was like "wait a second, where did that come from," you know, and then in the next couple of weeks I'd only gain like two…

Interviewer:…so…

ADL07: it was a little, those were a little stressful, 'cause it was like "oh, if I gain 8 pounds every 4 weeks then I'm gonna be huge"…(laughs)…that's not good for anybody…um, but it wasn't really a huge stress going into being pregnant and it hasn't been a huge stress, or, you know, issue during the pregnancy so…and I saw that there is a post about how to get rid of my baby weight…(laughs)…like "oh, I have to save that one."

Interviewer:…(laughs)…um, so, overall how do you feel about the amount of weight that you've gained so far in your pregnancy, do you feel that it's the right amount, do you feel that it's not enough, too much…

ADL07: um, I guess I feel like it's the right amount, it is, you know, about, so far, following the recommendations, you know, early on in pregnancy that I gained like the 25 to 30 pounds, um, so as far as I can tell I'm pretty much on track and again even those weeks when I would go in and it seemed like the baby had a growth spurt, or I had a growth spurt, or something, you know, the midwives don't seem to be too alarmed and it seems like it just, you know, it all kind of works itself out…

Interviewer: mhm…(agrees)

ADL07: um…and it also seems like, you know, I have some control over it but not always that much control over it, so…

Interviewer: and would you say that receiving the messages changed your eating habits at all?

ADL07: um, I think it helped to get me back on track to eating kind of in a healthier way…(missing audio)…that I felt like I was prior to pregnancy, 'cause it was, you know, soon as I got pregnant I just kind of got stuck in this rut of comfort foods, and went from being a person that ate salads everyday, to not wanting to eat salads…(laughs)…so now I'm

trying to, you know, be a little bit better, um, so yeah it's just good to definitely have those reminders to eat, eat, you know, a variety of colors and…(laughs)…things like that, and different reasons why I would want different fruits or vegetables, or whatever, you know, different vitamins and stuff like that that both the baby and I need.

Interviewer: did you, did your role change at all during pregnancy in preparing and shopping for food?

ADL07: um, I typically did the majority of it prior to pregnancy, and I still do, but we just, we've had some other changes going on….(laughs)…in the house, with my husband's work and, so we kind of pulled in his mom to help us, because we're both trying to, you know, do the right thing with eating, so we had her a couple of times come in and help to shop and prepare some healthy meals that we had picked out.

Interviewer: um, and how did the messages that you received influence when and how you take your prenatal vitamins?

ADL07: um, I don't think that it did, I was pretty good, I was taking prenatal, I had been taking prenatal vitamins for a while prior to getting pregnant, so I think that it was something that was already established in my daily habit…um…and I knew it was something that was very important, especially if I'm eating grilled cheese and pizza…(laughs)…so, um, so I've always I think been pretty good about that, so I don't know that that necessarily had an impact, but, you know, I went in being educated that prenatal vitamins are important, and again I was kind of, I had already established a routine with them on a daily basis, so that wasn't really affected, I don't think…

Interviewer: um, and did you change your physical activity levels at all after seeing the messages?

ADL07: um, I think kind of right around the time that I started getting the messages was when I was trying to increase things, and again that's where I feel like had I gotten the messages a little sooner in my pregnancy, it would have been helpful, because for the first 3 months I really was not very physically active, and I wasn't really sure how physically active I could be, or what kinds of things I could do, um, and it was the middle of winter, and it was a really cold winter, and uh…so I think, during my second trimester is when I kind of started to seek out information about activity and then kind of shortly after I started looking is when I started getting the messages, um, which kind of coincided with that, with the other information that I had found about exercise and, um, things like that so…

Interviewer: um, and so now that you have been feeling a little bit more energized…

ADL07: mhm…(agrees)

Interviewer: how does your activity level compare to prior to pregnancy?

ADL07: um, so…(laughs)…a couple years ago, I was really really active, and then just prior to pregnancy I was, you know, active on a daily basis, but I wasn't, like I hadn't been running for a while, you know, I pretty much just walked the dog, and now, I would say, um, you know, I walk the dog, I do, um, water aerobics class, I've been better at stretching and things like that, and then I've also added in like a prenatal yoga class. So right now I'm a little more active as far as, you know, specific like exercises…(laughs)…um, but I do find that, as far as just day-to-day activity, of like getting stuff done around the house, I'm kind of losing my stamina for that sort of thing…that's kind of come and gone…

Interviewer: um, let's see what would you say are the most…are the biggest things that have motivated you to try to increase your physical activity later in pregnancy?

ADL07: um, basically just hearing that it's good for the baby, you know, um, again getting the information, the reminders that it's gonna help with appropriate weight gain, hopefully prevents gestational diabetes, or blood pressure issues, you know, there…I think you sent a thing about how it's good for babies' brains and read somewhere else that it's good for babies' brains, to kind of, you know, be active and, um, moving, so…the biggest thing is like hearing that it's good for the baby, that makes…makes it easier to wanna go out there and do it.

Interviewer: Um, and during pregnancy did you have access to a cell phone the entire course of your pregnancy?

ADL07: yes

Interviewer: and your number, like the cell phone didn't change or anything like that?

ADL07: no

Interviewer: and do you have a smartphone?
ADL07: yes

Interviewer: okay

Interviewer: um, so…thinking back to all the messages that you've received, and…

ADL07: mhm…(agrees)

Interviewer:…if it helps to actually look at them you're more than welcome to do that, I'm just curious which messages maybe stood out the most, or if you ever remember going back to a message to look at more than once

ADL07: um…I'm sure I probably did go back and looked at a message more than once…but I don't know…(laughs)…yeah I'm going back to my little…I'm going to facebook now, I have to, like, remember…um…(pause)…yeah I don't know if I went back to one more than once or not, and then what was the other…sorry…what was the other question?

Interviewer: um…

ADL07: I feel like there were two parts to that.

Interviewer: just really similarly like ones that, um, were favorites or, you know…

ADL07: okay

Interviewer:...they instilled some sort of feeling in you that, you know, that might made you smile or made you get up and go do something.

ADL07: um, yeah it's hard to remember because, especially with my pregnancy brain…(both laugh)…it's like everything is in the moment, it's like I do it and then it's gone… (laughs)…um… yeah I don't, I don't know… (laughs)

Interviewer: um, so along those lines, this might jog your memory…

ADL07: oh oh

Interviewer:…some of the messages had links that you could click on…

ADL07: mhm…(agrees)

Interviewer:…do you actually recall clicking on any of the links?

ADL07: I think I clicked on a lot of the links, 'cause whether they be like a recipe link or an article link, um, usually like when I would see, you know, if I'd go to facebook for instance…um…and I'd see that there was like a new addition to the, you know, if you'd posted something then I usually would look at it immediately, and then kind of browse, to whatever the link was then in there…um…or same thing with the text messages, I usually would kind of look at it immediately and say: "oh, is this something I need to read or is this not pertinent," or whatever, so…

Interviewer:ehm, and of the types of links that you got there were links to maybe news articles…

ADL07: mhm…(agrees)

Interviewer:…webpages, videos…or did you find that you enjoyed receiving links to certain types of information more than others, like a video versus a website?

ADL07: um, as long as the information was interesting it didn't…I mean I think getting a variety of things is good, you know, depending on what the information is then…a video or a website would be helpful I guess…I guess maybe for me just like a link to a website that I can read, you know, that way I can look at it when I'm at work or I can, you know, when I

first wake up in the morning, or, um, I guess it's easier to…kind of look at the information on a website, or if then that website has more information then it's nice to have the link to it, and then you can kind of keep going back and looking at it, as opposed to video that you have to like have the time and the quiet to be able to watch it, so it's a little more restricting maybe…

Interviewer: and do you ever recall watching any of the videos?

ADL07: um, I probably…I don't remember that there were that many…I feel like there were more, just tidbits of information, and links and other ways…

Interviewer: um, and based on how you use your computer and your phone…

ADL07: mhm…(agrees)

Interviewer:…if you used the link to a video would it be better to receive it on facebook, or your…on, you know, maybe a computer, or is it easier to watch those on your cell phone?

ADL07: um, I don't think it matters, I think either way is probably easy enough.

Interviewer: um, have you joined any other email, text message, twitter, blog or facebook groups about pregnancy that you haven't talked about yet?

ADL07: um, my friend signed me up for some, like, [name redacted] breast feeding group, or added me to a [name redacted] breast, breast feeding group, and they talk about breast feeding but they also have a lot of, um, you know, pregnancy questions and answers, some post-partum questions and answers, and stuff like that so…you know, but it's mostly just people going on and posting their questions, and then…a crazy amount of answers to follow…(both laugh)…and again some of them are crazy…(laughs)…so….

Interviewer: um…the email listserves that you are signed up for…

ADL07: mhm…(agrees)

Interviewer:…how often do you typically receive information from those sites?

ADL07: um, I think they usually send things, like some of them send every week, you know, it's an update of "oh your pregnancy, 36 weeks," um, and I think some are a little more frequently, I don't really pay that much attention…but it's usually, I mean I'm usually getting something every couple of days at least…

Interviewer: um, okay, so since you were in the facebook and the text message group…

ADL07: mhm…(agrees)

Interviewer: you…at some points if we were sending messages to each your phone and facebook every day…

ADL07: mhm…(agrees)

Interviewer: you could be getting like up to 12 messages a week, so I'm just curious what you though about the quantity of messages, was it too much, not enough, just right…

ADL07: um, I felt like there were times where I would get a message and I would think: "oh, I haven't gotten anything for a little while," it seem like…um, and then there were some days like "oh, here's a message on my phone and here's a message on facebook, and they're different"… (laughs)…you know, so…I don't know I mean I think getting a message everyday is good, I mean its been fun for me, I like the information, and then, you know, I have a little app that I check everyday that gives new information as well, so…

Interviewer: mhm…(agrees)

ADL07:…I don't know, I…I don't think you can ever have too much information sometimes…it wasn't, it definitely wasn't overwhelming, it was definitely welcomed information.

Interviewer: okay

ADL07: you know, and again some of it was just simple, and some of it's not necessarily pertinent to me and I kind of just skipped over it, some of it I felt like, you know, I tucked in the back of my head and remembered to make smart choices that day…(laughs)…

Interviewer: um, where there any topics that you wished that you had seen in what was included in the study's facebook page and text messages?

ADL07: um…hmm…not, nah, I think, I mean, I think…I think…well your goal was kind of nutrition and…fitness, and I feel like they were covered, and I think those are both important things…um…and all the other little topics, their other, you know, symptoms and what not are easy to find in other places or to talk to your practitioner about or something like that, so…I think kind of sticking to the basic things about just kind of trying to be healthy, and eat right, and making sure you're exercising and stuff like that are good topics to focus on…

Interviewer: um, do you think that something like a facebook group or a text messaging program would be a good thing for health care providers to provide to pregnant women?

ADL07: yes, probably, because they have access to so many women, and they see them kind of from the start, and I think…that… it would be a nice addition to something that they could provide.
Interviewer: um, and have you ever heard of text for baby?

ADL07: text for baby?

Interviewer: mhm, it's a text messaging program offered, um, through the government, that also has a lot of corporate partners…

ADL07: I don't think I have.

Interviewer: okay...and, um, if you remember, from when you signed up for this study, one of our primary goals was to create messages…

ADL07: mhm…(agrees)

Interviewer:…that would be useful for pregnant adolescents…

ADL07: mhm…(agrees)

Interviewer: And, I mean, thinking about the messages that you received…um…do you think that, you know, that these facebook messages and text messages would be a good way to reach that population?

ADL07: um, yes, 'cause from what I know about teenagers these days, which is very little…(laughs)… but I do know that they're kind of attached to their technological devices, their phones, and facebook, and they're very involved in that, um, and I think a lot of the little things that, you know, just a lot of the information about certain vitamins or minerals that are…(audio issues, distorted and incomprehensible)

(skype disconnects)

(phone ringing)

ADL07: sorry…(laughs)…

Interviewer: no, it's okay

ADL07:… I don't know what's going on…(laughs)…

Interviewer: it's probably because this involves me, I am not really great with technology, so I'm sure that the computer is like "wuahaha, I'm just gonna spite Interviewer as much as I possibly can,"…(laughs)…ahm, I mean, I think those are really all the questions that I have but I just wanted to ask, is there anything that we talked about earlier that you thought of another answer to that you didn't get to say that we could go back to at all?

ADL07: Uhm no, I'm sure something will pop into my head like at 5 o'clock this evening and I'll be like "Oh! I should have said that," or, you know, but at this point I don't think so…

Interviewer: okay, um, and do you have any questions about anything that I can answer before I let you go?

ADL07: I guess, what are you, so what are you doing, like, once you get all of the information, you finish all the interviews and stuff, like what's your next step with this project…

Interviewer: um…

ADL07: if I can ask…

Interviewer: yeah, yeah, our goal is to, once we have all the interviews to transcribe into written text, we'll go through and we were gonna look for general themes across all of the women, and then wanted to compare our adult women to our teen cohort, and see if there are differences in motivators, in just general health and wellbeing behaviors…

ADL07: mhm…(agrees)

Interviewer: um, and then our goal is to write a paper, um, for a scientific publication…so, I mean that would be probably a year from now I would say…

ADL07: mhm…(agrees)

Interviewer: 'cause it always takes way longer than you think it will, um…and…

ADL07:…(laughs)…

Interviewer: and I think, once that is out there, and we have like some of the data analyzed then we could even add on like new projects and build off of this, so one thing I would like to do it was maybe add, I mean from what I can tell so far…I mean teens are a very unique group, and I don't think that just sending them little reminders is enough to motivate them to make the healthiest decisions…

ADL07: mhm…(agrees)

Interviewer:…all the time, so I think I would really like to try some sort of mentoring program where you're still sending the messages but maybe they are matched up with an adult volunteer who sort of takes them under their wing and adds…(audio missing)…that extra piece to motivate them…

ADL07: oh yeah…

Interviewer: um, because I think it's hard at that age for them to make healthy decisions on their own, I mean they have so many things influencing every little second of their day that…

ADL07: yeah

Interviewer: um, and they're, I don't know, easily influenced at the drop of a hat so…

ADL07:…(laughs)…well it's hard! I mean, you know, I'm in my thirties and I had lots of education on nutrition and, you know, I…I…for the most part I think I'm a pretty healthy eater, you know typically try and be a generally healthy person, um, but when I was a teenager, I used to like come home from school, and make brownie batter, and divvied it up into two bowls, and my friend and I would like sit in front of the TV and eat brownie batter for a snack, I mean it was disgusting…(laughs)…you know…

Interviewer: that sounds exactly like me…

ADL07: but like the choices I made as a teenager were horrible and nobody taught me…(audio missing)… an, you know, I didn't feel great, and I didn't know why, and my parents didn't really know what I was doing, or, you know, like they would give me a healthy dinner but the rest of the day I was on my own to do what I wanted and it was never a good choice so… um, you know, I think that something like that would probably be a good thing for teenagers, definitely…and then also... yeah just to, I think the other thing too… just… in hearing people who in general, not pregnant people, but just in general who wanna be healthier, that it's so hard to… if you don't know how to do it, like if you're not used to doing it, and your whole life it's been eating like comfort foods, or fast foods, or really, like, fried foods and you're, you know, you don't know how to go to the store and pick out a healthy meal and cook it, so to have someone maybe that can like say "hey, today we're going to take a walk and then go to the store and make this really delicious salad and it's gonna be good for you and your baby," sounds like a cool plan to…

Interviewer: cool, so that's good to know…(laughs)…

ADL07: I support that idea…

Interviewer: yeyy! Thank you…

ADL07: 'cause I think that, that's probably one of the challenges too, as it like you can put the information up there but then people can still say "well I don't get it," or like "okay so my baby needs zinc and iron and yeah maybe I can eat beans, but how do I make beans? Does that mean I go to Taco Bell and get a bean burrito or…" (laughs)…you know, so…

Interviewer: yeah, I mean I think WIC has been helpful to a lot of the girls that they are able to sign up for that, um…

ADL07: mhm…(agrees)

Interviewer: so, a lot said "I started eating more fruits and vegetables because that's what I'm allowed to buy with this money"

ADL07: oh nice!

Interviewer: and I'm like "wow ok!" but at least you're eating it so…it's nice to know that some of our outreach programs are actually working…so that's good.

ADL07: yeah and that's the other thing, I don't, you know, I'm so far removed, you know, I grew up in a house with, you know, opportunities, financial, you know, we…we had food on the table and we have education and, um, you know, I was able to go to college, and to receive more education and so, you know, I don't know what it's like to be a teenager who is living a completely different lifestyle from myself, so… kind of… to take into consideration all of those struggles that it must be to be a teenager who maybe doesn't have the financial means or the educational means to even know like where to begin, that would… that's… just.... you know, I don't think about that on a daily basis…(laughs)… so…

Interviewer: yeah…that's definitely hard, it's been hard for me to connect with them too…

ADL07: mhm…(agrees)

Interviewer: I mean I think I relate more to you than I do to them so…

ADL07:..ahm…

Interviewer: but it's…it's been very…I guess…educational all around, for everyone…(laughs)…

ADL07: yeah

Interviewer: probably…so it's been good, I'm glad that we did this project, its been exciting…

ADL07: well…well hopefully it will make a big difference in lots of babies' lives…

Interviewer: I hope so…um…

ADL07: and hopefully with my next baby I will be healthier from the beginning…that's my biggest goal…it's like learn from this pregnancy and then…(laughs)…get really healthy after I have the baby and stay healthy during the next pregnancy…(laughs)…

Interviewer: I think that you're on a good track for that so…I can't imagine that you would have any trouble…um, so, um…this is the last interview and the only two things that you have left are our final dietary recall and then that nutrition knowledge survey that you also filled out at the beginning of the study…

ADL07: mhm…(agrees)

Interviewer: and so I think what would be easiest is if I have the study recruiter maybe come to your next appointment and then maybe she…

ADL07: okay

Interviewer: she could snag you while you're in the waiting room for the survey 'cause that doesn't take very long to do, and then maybe just like 15 minutes after your appointment do the diet record, and then…

ADL07: okay

Interviewer: …and then I'll have her give you the remaining gift cards for all 3 things so you should get 1 $30 gift card and then 2 $10 gift cards.

ADL07: okay

Interviewer: um, so I'll actually email her right after this, and you're at the point where you go weekly right?

ADL07: I am!

Interviewer: okay…(laughs)…so then maybe, do you have an appointment later this week?

ADL07: no. Yeah I have an appointment on Monday, I just went yesterday…

Interviewer: okay…um, so she's usually…

ADL07: (distorted)

Interviewer: actually, you know what she might be on vacation, I will… I'll text you when I know when abouts… I think that she's actually starting vacation next week through…I wanna say the [date redacted] or something it's a pretty long vacation, but you'll definitely get that…

ADL07: okay

Interviewer: and if anything happens as in like you haven't gotten them within the next three weeks for sure, please let me know and…

ADL07: okay

Interviewer:…I'll make sure that they get to you…um…but I do know that she was going on vacation towards the end of [date redacted], so that just might extend it a little bit longer, since I am…

ADL07: okay

Interviewer:…right now in [location redacted], so I can't drive up and give it to you, um…

ADL07:…(laughs)…

Interviewer: um, but if that sounds okay then, I guess you're all done with this study, so, except for the last two things…

ADL07: okay

Interviewer: but best of luck with the rest of your pregnancy, and the delivery, I hope everything goes really well…

ADL07: me too…(laughs)…

Interviewer: I'm sure it will, and are you having a boy or a girl, I don't remember by…if you (ADL07 talks over)…

ADL07: don't know, we don't know…

Interviewer: okay, so surprise, that will be fun…

ADL07: its…(coughs)… from what I can tell it's a tap-dancing octopus, but that's all I know…(laughs)…
Interviewer:…(laughs)… well that could be interesting too…

ADL07: yeah…(laughs)…

Interviewer: um, so you'll probably get lots of green and yellow things…

ADL07: yes!

Interviewer: but that's ok, they say that a long long time ago pink used to be the color that they used for boys and blue was for girls…

ADL07: oh really?!

Interviewer:…yeah, I think like in…like…like a 100 years ago or something like that, I don't know…

ADL07: oh my gosh…

Interviewer: I don't know where I read that, but I read that recently and I was like "that's really interesting"…

ADL07: well my sister…so she's been in India, and she just came home recently and she says that there they, because boys are…um…more revered, you know, everybody obviously wants a boy, and, um, that they dress their boys up like girls or they dress them like really…in these like ugly clothes so that the evil eye won't, you know, curse the baby, or hurt the baby, or anything like that…

Interviewer: oh my goodness…(laughs)…

ADL07: they put their boys in these like ridiculous dresses and in these like hideous outfits…(laughs)…

Interviewer: oh my god…(laughs)…

ADL07: to protect them…

Interviewer:…(laughs)…it's so different from here…alright well I guess I'll let you get on with your day, but thank you so much for participating, I really appreciate it, and I hope everything goes well and, um, that you enjoy all of your time with the baby, they grow so fast so…(laughs)…

ADL07:…(laughs)…I know, that's why I'm like trying to work right up until the baby is born so that all of my time off is gonna be spent with the baby, so…

Interviewer: that would be really nice…

ADL07: yeah…so, alright, I'm just looking at my schedule and probably…(missing audio)…most likely appointment so hopefully…we can catch up with someone then…it's usually, I usually go on Mondays or Tuesdays…

Interviewer: okay, and those are good days for [name redacted] I know, because the…(ADL07 coughs over Interviewer's word)…all come in on Wednesday, so her other days are pretty open, so…

ADL07: okay

Interviewer: I'll just email her after we hang up, just to see what's going on, and I can text you and let you know for sure, um when she'll be around.

ADL07: okay

Interviewer: cool, alright, nice talking…

ADL07: alright

Interviewer: have a good rest of the day…

ADL07: thanks! Good luck with everything!

Interviewer: thank you! You too…

ADL07: bye!

Interviewer: bye!

ADL08

ADL08: Hello
INTERVIEWER: Hi, How are you?
ADL08: Good, how are you?
INTERVIEWER: Not too bad. How are things going, it sounds like you've been really busy lately.
ADL08: Yeah, I've been busy and just really tired
INTERVIEWER: yeah?
ADL08: yeah
INTERVIEWER: your due date is really soon so that makes sense haha. Um so I know that, I don't know, did [name redacted] have you fill out the surveys yet?
ADL08: yeah no I think I have like 2 left or something like that
INTERVIEWER: yeah there's the diet recall, and then there is the, so you filled out that nutrition survey at the very beginning of the study and then you do that again. Its just 10-12 questions about nutrition and food and there was something that you were supposed to sign awhile ago that you get $10 for it and it's just a signature page.
ADL08: okay
INTERVIEWER: so I was going to do the interview with you and also the diet record. I don't know if you had a preference on which order we do them in
ADL08: let's do the interview first
INTERVIEWER: okay! That works! Um okay, so do you remember from last time that our goal is to just kind of look at where you go for information about health and fitness, nutrition during pregnancy, uhm so specifically the internet and any phone apps that you might have um and then also the second interview is really to find out what you thought about the messages you received and if any of them really stuck out to you or if you tried any thing any of the ideas or clicked on links and stuff like that.
ADL08: okay
INTERVIEWER: Uhm so if that sounds good, I'll just check to see if you have questions before we start
ADL08: um, I can't think of any
INTERVIEWER: okay, perfect! Well, we can try to be fast because I know you have a couple of other things to do and I don't want to keep you all morning. Um, so just to kind of like introduce talking again and just to follow up from last time, how do you think your pregnancy has been going?
ADL08: uh, good
INTERVIEWER: any exciting events or worrisome events?
ADL08: no, I don't think anything really stuck out I think that the first trimester was a little rocky um but after that I think it kind of smoothed out.
INTERVIEWER: that's good, um and what would you say the best part of this pregnancy has been?
ADL08: um I'd say the fact that it wasn't as uncomfortable as some of my other ones, um it was a little easier than my second and my third so I think that was probably the best part
INTERVIEWER: last time we talked I asked what hopes and dreams you had for your baby. And could you just reflect on that question again, and so when you think about the future for…

ADL08: I think I don't want to put any specific expectations on my kids, just so that they're happy and healthy and able to be themselves in whatever they choose to be. I'm not sure what my answer was before just nothing specific just that they're happy and that they're healthy and that they get to express themselves.
INTERVIEWER: I think that was the same answer you had last time. Um Have you changed the way you prepared food since you became pregnant?

ADL08: Still just trying to be aware of… I haven't had a really good appetite this pregnancy just trying to be aware that I get nutrients like I'll drink a lot of green juice things so even when I'm not hungry I'm kind of making it count when I do eat something, so I guess that's a little but of a change, before I was eating healthy but I had more of an appetite; now I am aware that I need to eat and that I need to get my nutrition so I make sure I can have those things and make sure I'm getting them when I'm not hungry

INTERVIEWER: you mentioned green juice smoothies. Were there any other foods that you tried to fit in when you weren't hungry but knew you needed to eat something

ADL08: um a banana was always kind of a go to uh. (Skype sound) Are you there?

INTERVIEWER: um yes, it says there's some sort of Internet problem. I don't know if you can see me anymore, but…

ADL08: um I can't see you but I can hear you

INTERVIEWER: okay same here so lets just keep going with that haha umm

ADL08: either that or like you know if I had stuff for a salad on hand, uhm I ate quite a bit of avocado and tomato throughout the pregnancy so that banana, either a green juice smoothie or a blue juice smoothie, just something that I knew that had a lot of vitamins and stuff in it.
INTERVIEWER: and are those things that you had eaten before or things you kind of switched to cause you knew they were better during pregnancy?

ADL08: uh some of them I had eaten before. Bananas I'm not a huge fan of so that I'm not as inclined to eat if I'm not pregnant so that, and I usually would go for the blue juice smoothie before the green juice smoothie so that um and with pregnancy I try to incorporate the green more cause it has a lot more vegetables and I'm not a big raw vegetable eater other than salad. So I either try to put it in a salad or I'll drink the green juice

INTERVIEWER: so since you said you haven't been as hungry as you have been in previous pregnancies, has weight gain been an issue that you've thought about or worried about at all?

ADL08: um I think toward the beginning of the pregnancy just because I wasn't really gaining but I think I gained a total of about 15 lbs so uhm I don't think it was something I was worried about, but it was something I was aware of in the back of my head which is

why I made sure that… Cause I know the number isn't as important as the nutrition which is why I made sure I was eating something that had nutrition in it because I knew I wasn't gaining all that much

INTERVIEWER: do you feel happy with the amount you have gained during the course of your entire pregnancy?

ADL08: yeah I'm not, I'm not um too concerned with the weight change, huh? [kid talks in background] I know that the quality is more important than the quantity, so I guess there was a part of me that was satisfied it was only 15 cause its easier to loose but I think by the 4th one you're not as preoccupied with that kind of stuff.

INTERVIEWER: That makes sense. And have you had any cravings for specific foods that are maybe not typical for you to eat before?

ADL08: um donuts, I think that's the only thing I think before this I cant remember (talks to kids) I think beforehand I cant remember the last time I had a donut and its like the one thing in previous pregnancies it had always been chocolate or pizza or something like that [kid whining] [talking with kid] yeah so I think donuts is the only thing that I really craved

INTERVIEWER: any particular flavor of donut?

ADL08: Just the regular glazed donut. I will usually eat that with yogurt. And crunchy food so not anything in particular but just stuff that's crunchy

INTERVIEWER: what about ice, have you had cravings to chew ice?
ADL08: no
INTERVIEWER: and then any non food cravings like for?

ADL08: just still smelling soaps. I just love to smell soaps, but not actually ingest anything

INTERVIEWER: Okay, um let's see, has there been a person or a like place a book or internet site or phone app that you felt had the best information about pregnancy?

ADL08: Um I think it kind of varies. I think American pregnancy has pretty great information, what to expect I think has some good information. Uh I think its kind of touch and go. I don't think there's one website that's overall really really good, like if I had a question about something I would usually Google it and see the top couple sites that would pop up (yawn) and there wouldn't be one specific one I would go to because I'd click on one and it would have the best information for that particular question, but maybe another so I think that those were the two that stood out the most. What to expect and American pregnancy.

INTERVIEWER: okay, um and so when you have Googled things do you feel that you Google things very often? I know this is your 4th pregnancy so how does the amount that you search for information compare to previous pregnancies?

ADL08: umm, not quite as much. I think more so this time around if I'm bored or cant sleep for some reason where as before I didn't know as much but I think every time there is something new that you experience that you kind of look to see either who else is experiencing it or what information is out there, so I would say less but you know there is still that curiosity and that boredom that you just still end up searching stuff just because.

INTERVIEWER: and do you remember any searches during this pregnancy that specifically had to do with physical activity or diet or nutrition?

ADL08: Um, I don't think so. Nothing for exercise because I I—can't remember if it was before or after the last interview but I had a concussion and I tore a ligament in my ankle so I haven't been able to be as active in this preganancy as I have in past pregnancies. As far as nutrition I think… I think I may have searched like knowing that I needed more calcium and magnesium in my diet. I may have searched like the best foods to get those. Similar to some of the messages that were sent that had a particular nutrient and the best places to find them, so I think those were the only searches I did concerning nutrition.
INTERVIEWER: Was there anything like specific that prompted you to look for food sources of calcium and magnesium?

ADL08: Again, just being aware that I needed to… I think that in past pregnancies I wasn't as aware of that even… if you're not eating right the baby will take the nutrition from you I wanted to put it in my body extra instead of having the baby take it from my system. So I think that awareness made me what to know (interrupted) as much as I could about calcium and iron and those types of things [talking to kid]

INTERVIEWER: um so just to kind of continue to follow up on that were these things that you were prompted to look at because of the messages from study or were these things that your doctor had talked to you about?

ADL08: I think the messages from the study were just going hand in hand with what I was doing already. I don't think it was more so the doctor; the doctor was making sure I was taking prenatal vitamins and I don't think it was anything prompted from the doctor. I think it was just me basing off from experience. And then I think just the messages about the nutrients just kind of went hand in hand with it and were just a helpful piece of information to have.

INTERVIEWER: Um and so and then thinking specifically about the messages that you did receive through text, um was there any type of information that was sent that you felt was most relevant to you?

ADL08: I think that nutrition was most relevant to me the little pictures that would come through, like I remember folic acid and the little pictures that would come through that had a lot of information about different nutrients.

INTERVIEWER: um and did you change any of your behaviors after seeing some of the messages?

ADL08: um I think that just trying to include you know more things like different fruits and vegetables that contain those things

INTERVIEWER: were there any messages that you disagreed with the information that was sent?

ADL08: no I don't think there was anything that I disagreed with. I didn't pay as much attention to the weight and fitness ones but I don't think there anything that I disagreed with

INTERVIEWER: um so you mentioned that when the messages had to do with weight or fitness those were of less interest than nutrition. Could you tell me a little bit more why some were of greater interest than others?

ADL08: I think just for me, I don't know if it's just me or if it's because it's my 4th but some things I think are… when you're pregnant when it comes to wanting certain foods I think it's a really fine line cause sometimes when you want something when you're pregnant you don't really care… you don't care about the nutrition, you don't care, you know. You just want what you want when you want it and I think so for me this time around I didn't want a whole lot of stuff that was junky other than the donut, but at the same time when you're pregnant, you just don't really care about uhm making a whole lot of restrictions to your diet especially if you have a craving, you're not thinking about it from that end. I think the nutrition part was something I was able to do it this time around because it was my 4th pregnancy, but I remember from subsequent pregnancies you just don't really care about that kind of stuff. You're doing your best to keep food down or doing your best to get enough food or you're just completely satisfied with whatever craving that you have, and I guess I just don't believe in putting much emphasis on stressing over diet during pregnancy because I think it's the one time you listen to your body and not be too hard on yourself as do on something even if it might not be the best for you.

INTERVIEWER: um and then on the flip side what about fitness. So I know that you said you had a little bit less energy then before so when someone sends messages about being active how… what are the things that go through your mind?

ADL08: I think that the activity I think that those would have been more useful and relevant… but I think just specific to my situation because I couldn't… I think from November through March I was incapable of activity so I think if that would have been different I would have paid more attention to them. So I think that for my specific situation, like for a while I couldn't even walk, I was on crutches. And um where as past pregnancies I had been playing softball until like 5-6 months so uhm I think that that was just specific to my particular situation this time around.

INTERVIEWER: okay that makes sense. Um and then for weight gain, so I know that you said that's something that you know you worry a little bit less about with each pregnancy because you realize which things are important and those sorts of things, but did any of the messages prompt you to think differently about weight gain?

ADL08: um no I don't think that about weight gain. I think that um no, I would say no but then again I think that's just because this is number 4 for me.

INTERVIEWER: was weight gain something that you worried about in previous pregnancies?

ADL08: um I don't think it was ever anything I worried about during pregnancies I think the pregnancy that I gained the most it was something that bothered me after the fact, but I don't remember you know, putting that much attention on it during the pregnancy (talks to kid)

INTERVIEWER: um, and so when you received messages you got a lot of different kinds pictures, short sentences, uhm… sometimes they were pictures, sometimes they were website links. Do you think you had a favorite of the different types of messages you were receiving?

ADL08: I think the picture ones caught my attention the most (talks to kid)

INTERVIEWER: um and did you ever click on any of the web links that were sent?

ADL08: um I don't think so. I think for me I'm always kind of in the middle of a million things so (distracted by kid) like a readily accessible piece of info like the pictures fits into what I have going on rather than to actually click on a link and pay that much attention to it

INTERVIEWER: Ok, and thinking back through all of the messages are there any that really stood out to you that you remember uhm and even went back to more than once?

ADL08: I don't think there were any I went back… to but the nutrition ones and that's just the type that I just have overall interest in knowing you know more about like vitamins and minerals so those were the ones that stuck out the most for me.

INTERVIEWER: and what were your thoughts on the number of messages you received each week. Was it too much, not enough, just right?

ADL08: It was definitely not too much I would say just right. Uhm I don't know if I would say it was not enough, but I would definitely say it wasn't too much.

INTERVIEWER: okay, and then the course over your pregnancy were there any other email, text, websites that you joined where you would received information on a daily or weekly basis?

ADL08: no

INTERVIEWER: okay, um. Is there anything that maybe you had thought about during pregnancy that you don't remember receiving a message from us about that you thought would be good for us to incorporate in the future?

ADL08: umm, maybe I mean I don't know, um I guess I don't know like what if all the info was supposed to be you know more so nutrition and exercise, but um I don't know, maybe more about uhm… like different things that are normal to experience. I think for pregnant women that's the biggest thing you look for, like is what you're experiencing uhm the same that everyone else is experiencing. So having cravings that maybe aren't the most nutritious but being able to indulge in them in moderation anyway just knowing that you're not alone in that might be… It's always helpful as a pregnant woman to know that you're not the only one going through something, so maybe just more messages that kind of emphasize that part of it. 29:14

INTERVIEWER: um is there anything else that you maybe thought of about the messages that I didn't specifically ask about that you want to share

ADL08: no I guess some (stutters) just some I like I said the ones I didn't pay attention to most were just, cause again it was my 4th pregnancy were the ones that… you know, encouraged you to pay more attention to weight gain and diet than what I would do and some I felt kind of funny because, like I said, when you're pregnant you are just do not care and you're not thinking about um… you're just not thinking about things the way that you would if you were not pregnant. For me it was just not being new at it; it just was not as helpful for me personally.

INTERVIEWER: so I think that I've heard a lot of women say things like that where, you know, you have a craving and whatever's happening… that it's just easier and better to fulfill whatever that craving is instead of eating something healthy, but it sounds like you have a good balance in being with tune with knowing you need these nutrients, fitting in more nutrient dense foods. Is there something you think that could really motivate women when they want the donut but maybe they should have the banana instead? Is there something that motivates pregnant women to switch to the healthier option?

ADL08: I think maybe that rather than encouraging switching, encouraging adding in. So more so encouraging women not to have the donut because sometimes what I'll do, I will have the donut and the banana and the yogurt. So you know even though I know that the donut is not the best thing in the world, you know I am getting the protein from the yogurt and you know I am getting the nutrition from the banana. Uhm so maybe just encouraging them to just work around those cravings instead of resisting them. I think that resisting cravings is, to a pregnant women almost comical because, again you got so much else going on with hormones and everything else that stopping yourself from having something that your body is craving is… to a pregnant woman it almost sounds ridiculous so I think that by encouraging them add in more nutritious foods rather than… because I know with, for instance one of my pregnancies I craved chocolate all the time and telling me not to eat chocolate would just… be funny for me because there is no way that I was going to not eat something that my body was craving that strongly. But encouraging me to maybe have the

chocolate but also maybe have some berries with it. You know, encouraging making those modifications so you're including the craving, but you're also including something nutritious with it. I think that might be more helpful.

INTERVIEWER: okay, thanks that's really helpful. That's most of the questions that I had for you, but is there anything you thought of that you might want to add to a previous answer or anything else that we talked about that you would to share?

ADL08: Um, I don't think so. My brain is a little foggy right now so I don't think I can pull anything extra out of it other than what I am being asked.
INTERVIEWER: Okay that's fine. I think we can go ahead and switch to your diet record and that shouldn't take more than like 15 minutes at the most, and then we'll get you out of here

ADL08: okay

INTERVIEWER: okay, so for the diet record we'll record everything that you ate yesterday so ill just start by asking you what time you woke up

ADL08: yesterday, I woke up around 7:30

INTERVIEWER: okay, and when was the first time that you ate or drank something after you woke up?

ADL08: um well I usually always have a glass of water right when I wake up um so that was the first thing I had to drink and then I had a protein bar and some vitamin water at about 8 o'clock

INTERVIEWER: ok so when you woke up, the glass of water would you say it was 8 ounces? 12 ounces?

ADL08: I think it was a 10 oz glass, I think
INTERVIEWER: the protein bar, was that a specific brand and flavor that you can recall?
ADL08: I actually have one right here, it was Nugo chocolate chip
INTERVIEWER: and the vitamin water, did you drink the whole bottle?
ADL08: uh about 3 quarters
INTERVIEWER: and then do you remember what flavor it was?
ADL08: orange
INTERVIEWER: did you finish the bottle later in the day
ADL08: yeah I think by the afternoon
INTERVIEWER: okay, and that's a 24 oz bottle?
ADL08: umm 20 oz
INTERVIEWER: anything else that you ate or drank at 8 with the protein bar and vitamin water?
ADL08: no
INTERVIEWER: okay so when was the next time that you ate or drank something

ADL08: the next time was about 12 I had a um bagel sandwich
INTERVIEWER: was that something that you made at home or did you get it from a restaurant
ADL08: no I made it was a plain bagel with cream cheese and a veggie sausage patty, like a non meat sausage patty and a little bit of taco sauce and then just water with that
INTERVIEWER: um was the cream cheese regular cream cheese or low fat
ADL08: just regular
INTERVIEWER: was it whipped or non-whipped
ADL08: non whipped
INTERVIEWER: and just the top part of your thumb is a tablespoon, how many tablespoons of cream cheese did you use?
ADL08: umm like one and a half
INTERVIEWER: okay and then for the veggie sausage patty you had one of those
ADL08: yeah it was a morning star sausage patty
INTERVIEWER: um and for the bagel was there a specific brand
ADL08: um just Wegmans brand
INTERVIEWER: and it wasn't the smaller bagels it was the regular size?
ADL08: yeah
INTERVIEWER: and then you said you had water with that?
ADL08: yeah
INTERVIEWER: about how many ounces of water did you drink?
ADL08: about 8
INTERVIEWER: any sides like fruit or veggies a yogurt?
ADL08: no a little bit later I had a bowl of applesauce
INTERVIEWER: what time was that?
ADL08: that was about 1:30
INTERVIEWER: and was it the single serve packet of applesauce?
ADL08: no I poured it out of a big jar, I would say about two cups
INTERVIEWER: and it was regular?
ADL08: yeah, the natural applesauce and then I just add cinnamon to it
INTERVIEWER: okay, did you have anything to drink with the applesauce?
ADL08: um just more water I usually have a couple of waters on hand so just another 4-8 oz
INTERVIEWER: okay and then when was the next time that you ate or drank something?
ADL08: um, it wasn't until about 7
INTERVIEWER: okay and what did you have then
ADL08: half of a sub sandwich with lettuce tomato Swiss cheese and another veggie sausage patty
INTERVIEWER: um and what kind of cheese did you say?
ADL08: Swiss cheese
INTERVIEWER: okay,
ADL08: (talking to kid)
INTERVIEWER: um so for the sub sandwich you said that you ate half so for the part that you ate how many inches long would you say it was?
ADL08: well the full thing was 7, so I would say about 3.5
INTERVIEWER: okay, and was it white bread, wheat bread
ADL08: uh white bread

INTERVIEWER: um and the veggie patty was the same as at breakfast
ADL08: yeah
INTERVIEWER: and how much Swiss cheese would you say that you added?
ADL08: there was like a slice on there
INTERVIEWER: and for the lettuce and tomato, was it like sheets of lettuce and slices of tomato?
ADL08: it was like a small handful of shredded lettuce and like 2 slices of tomato
INTERVIEWER: so would you say that you had about a quarter cup of lettuce
ADL08: yeah I would
INTERVIEWER: did you add any condiments to that?
ADL08: there was a little bit of mayo and a little bit of mustard
INTERVIEWER: regular mayo?
ADL08: yeah
INTERVIEWER: and was the mustard just yellow mustard or a Dijon mustard
ADL08: just yellow mustard
INTERVIEWER: okay, and then about how many tsp or tbsp. of mayo and mustard did you add
ADL08: I think about a teaspoon on each
INTERVIEWER: um and I forgot to ask, when you had the bagel sandwich at noon, you added salsa to it how much salsa did you add?
ADL08: um it was about a tsp of taco sauce
INTERVIEWER: or right taco sauce. Um and did you have anything to drink with the sub sandwich
ADL08: um I had ½ of the same type of vitamin water and then I had one of those quarter sixed bags of those cheese puffs
INTERVIEWER: you said you had half of one
ADL08: yeah half of the orange vitamin waters
INTERVIEWER: and then the cheese puffs will you say that again?
ADL08: those little ones, those tiny snack bags
INTERVIEWER: okay, and were they white cheddar, yellow cheddar
ADL08: um yup the yellow ones
INTERVIEWER: okay, and was it a certain brand
ADL08: um I think they were like Cheetos
INTERVIEWER: okay and anything else you ate with the sandwich?
ADL08: no
INTERVIEWER: okay, and did you eat anything after 7 pm before you went to bed?
ADL08: yeah about an hour later I had a small piece of chocolate cake and a scoop of ice cream
INTERVIEWER: okay, so the chocolate cake did it have frosting
ADL08: yes chocolate frosting
INTERVIEWER: was it um a single layer of cake or two layers or cake
ADL08: uh two layers
INTERVIEWER: was it cut in a square shape of a wedge shape
ADL08: wedge
INTERVIEWER: if you had to guess, this is sort of an odd question: how tall was the wedge and how wide was the widest part of the wedge?

ADL08: umm lets see, maybe 3 in tall and 2 in thick
INTERVIEWER: okay, and do you know what size pan it was cooked in?
ADL08: no I have no idea it was just a store bought slice
INTERVIEWER: oh okay, perfect. And for the scoop of ice cream what kind of ice cream was that?
ADL08: vanilla
INTERVIEWER: and was it a certain brand
ADL08: uh wegmans
INTERVIEWER: and how much ice cream do you think it would be in cups
ADL08: maybe like ¼ of a cup one of those scooper things
INTERVIEWER: did you have anything to drink with that
ADL08: another glass of water, maybe a glass and a half so like 12 oz
INTERVIEWER: okay, and then anything to eat or drink before bed?
ADL08: no
INTERVIEWER: okay and then what time did you go to sleep
ADL08: uh last night I didn't go to sleep until about 12
INTERVIEWER: okay, and nothing to drink before then
ADL08:umm I may have had another half glass of water so another 4 oz of water
INTERVIEWER: alright well that looks perfect, thank you so much
ADL08: um so that's everything that I needed to do with you and then I think [name redacted] will have you sign that other sheet, do that last questionnaire which will take you 5 seconds. You get 30 for the interview 5 for the diet, 10 for the other thing and 10 for the nutrition questionnaire so 60 in total
INTERVIEWER: do you have any question for me?
ADL08:no I don't think so
INTERVIEWER: okay well thank you so much for being in this study I am so glad that you participating and best of luck with the delivery and afterwards
ADL08: okay thank you
INTERVIEWER: you're very welcome have a great day
ADL08: you too thank you

ADL09

Interviewer	Okay, um so it's been a while since we last talked and um we talked about a lot of things, nutrition and exercise wise earlier in pregnancy. Uhm how has your nutrition and exercise stuff been going since then.
ADL09	Um, You can tell from my double chin here that I've been eating more. Haha my appetite is definitely a lot better than it was in the beginning, uhm I've been able to eat more and enjoy food more. Um And so nutrition wise, Uh I don't know, I haven't been too mindful of exactly what I've been eating just that I've been eating more. And um exercise wise I feel better and I feel better when I'm up walking than vs sitting so I've been doing a lot of walking which has been good.
Interviewer	And I remember that you had mentioned that you were taking boot camp classes before pregnancy?
ADL09	Yes
Interviewer	And is have you have been able to find any classes that are geared towards pregnancy that you could enjoy that are similar to that
ADL09	No I mean, I know you posted a lot of videos and there are some Yoga poses that I know I could do but I haven't done it. I've just been walking, just about it.
Interviewer	Um what would you say has been the best part of your pregnancy?
ADL09	Um What do you mean, like…
Interviewer	Um anything, it doesn't have to be in relation to food or exercise, it could be something that you were just really excited about. Um in relation to the baby.
ADL09	Oh gosh, I don't know. I feel like I'm excited for the kids, for my other kids to get to meet the baby and be, you know, big brother and big sister. I'm excited about that. It's been really fun to watch them watch me grow and change and feel the baby move and I'm going to miss that. And feeling the baby move inside. Although now the movements are getting a little painful - not a lot of room in there.
Interviewer	Hehe I bet, and um and so I asked this question last time too and I just wanted to ask again and it's kind of a weird question so um don't feel like if you don't have an answer that you have to but I had asked what your hopes and dreams were for your unborn child.
ADL09	Um, Hopefully last time I said the same thing but I hope the baby is healthy and um grows up feeling safe and loved by you know family and friends and is uh… I don't know mostly just happy.
Interviewer	I think that was about the same as last time.
ADL09	That's what I hope, so. That's what I hope for him or her -
Interviewer	It's a pretty common answer, which I mean every Mom wants their, wants those things for their baby.
ADL09	To be healthy yeah.

Interviewer	Yeah, um so let's talk a little about food. Have you changed the way that you way that you prepare foods at all?
ADL09	Uh… not really my husband does most of the cooking at our house so, he pretty much caters to what I'm in the mood for that day haha which is nice.
Interviewer	Um so I know appetite in the beginning wasn't great um so as your appetite improved what things do you remember adding to your daily routines?
ADL09	Well I know like in the beginning I was mostly focused on fruit, that's all I can really tolerate and was craving and now it's more pasta. So that's been different. Um I'm trying to think what else changed. Um I work out of my car a lot so a lot of the time I stop, I still stop at like Wendy's and get lemonade or sweet things that I've been craving too so. But I still feel like I get full really fast so I feel like I'm not eating as much even now, even before I got pregnant. Because I--I feel full faster. This baby's big haha.
Interviewer	haha did they give you an estimate on how big the baby will be?
ADL09	Now I wish, they say they'll measure two weeks ahead of time, but my babies were big and measured 5 or 8 lbs and they were early so I'm anticipating the same for this baby too.
Interviewer	And did you… Are you one waiting to see if it's a boy or a girl?
ADL09	Yeah I am, we're going to be surprised this time. I think that's one of the most like exciting things about the pregnancy, we don't know what it is, and it's kind of fun to see everyone's reaction. Some people get annoyed like "Uh I want to know what you're having" And some other people are like " oh my gosh that's so great I wish I would've done that" you know, so it's kind of fun to get the mixed reactions.
Interviewer	Yeah it seems like you know like once you hit adulthood, there aren't many surprises left so it's kind of fun to like, make it so you get a surprise.
ADL09	Yeah that's what we were thinking. Plus I'm hoping to have a v-vag, so if I do end up having a C-section again I think it would be a nice thing to look forward to in the end. Of course you want to have a healthy baby but, it'll be a fun thing to uh you know look forward to if I have to have that.
Interviewer	Yeah, yeah, definitely. Um so you said that you've been craving sweeter things, have you had any cravings for salty foods at all?
ADL09	Well I'll tell you what I eat every night before I go to bed.
Interviewer	Okay haha.
ADL09	Well I eat a pickle, and uh cheese, and uh strawberry wake cereal, a bowl of cereal. That's pretty much my every night snack.
Interviewer	And is that because you crave all those things? Or did you hear something about combining them or
ADL09	No that's just—that's just what I Want to eat haha
Interviewer	Okay haha.

ADL09	It's been pretty consistent over the past—I don't know, gosh couple months, so I make sure I'm always stocked up on that stuff.
Interviewer	Nice.
ADL09	Cheese is different I wasn't a really big cheese fan before I got pregnant, so that's different but I am now.
Interviewer	Um, So do you have milk with the cereal?
ADL09	Yes
Interviewer	Okay
ADL09	And we have whole milk in the house because my kids are really skinny so they need the extra fat.
Interviewer	Um have you had any cravings during pregnancy for non-food items like um corn starch, couch stuffing, dirt, haha
ADL09	No, no no dirt haha
Interviewer	Okay that's good. Any smells that you've found that you like more or less that during pregnancy?
ADL09	Um no, not really. In the beginning maybe, but at, right now for a while now nothing has really been--I've never had an aversion to anything lately.
Interviewer	Okay, um have you had any challenges in relation to food during pregnancy?
ADL09	Nope
Interviewer	And um when you have, when your husband prepares food is it more common that things are pan-fried, baked, um broiled, grilled.
ADL09	Um a lot of sautéing like whatever our meals is, we have staples in our house like we kind of have the same meals every week, because it's kind of what I like so we have a lot of tacos, fajitas, of course this summer we did it with hot dogs and hamburgers, salads that kind of stuff. At picnics um I'm trying to think what else I like uh we have a lot of pasta with sautéed tomatoes and broccoli um with olive oil which is one of my favorites. um I really like chicken fingers and French fries right now. One of my favorites. Though that's pretty much it. I mean things we have, they are things we eat every week.
Interviewer	Well that sounds like they're all kid friendly too, which is good.
ADL09	Yeah they're happy yeah.
Interviewer	Haha
ADL09	The kids have to eat what we make, but we make things that they like so we're not like giving them crazy food you know.
Interviewer	No yeah they all sound like things , when I babysat kids they're all like "Yayyy"

ADL09	Yeah pasta is huge in our house so that bodes over well but I feel good because I give them tomatoes and broccoli on top of it.
Interviewer	Yeah yeah
ADL09	It helps.
Interviewer	Okay, so I know when a lot of women become pregnant they make a lot of changes to diet, and exercise and other things that they're doing, and so I'm just curious I know that you know you've been through pregnancies before so I know a lot of women tend to worry a little bit less with each pregnancy, and… but are there any things that you specifically decided to do or changed about your health habits with this pregnancy?
ADL09	Um I think with this pregnancy I wanted to walk, I definitely wanted to exercise more and walk more because I wanted to prepare my body for V-back and I really wanted to because I was so into working out and eating healthy before I got pregnant so I wanted to make it less or easier to lose the weight after the baby this time around because I know how hard it is for my body to shed those baby pounds. But I still felt short of what I really wanted to do when I was pregnant. I really wanted to do the prenatal Yoga, I really wanted to do swimming, but I haven't. it's been crazy with my schedule and the kids and trying to fit that stuff in, so. But I feel like this is the best pregnancy I've had like feeling wise I've been able to do as much and feel as good during my pregnancy. I think that has a lot to do with the walking and being active. I don't have time to come home and take naps anymore in the afternoon like I did my first pregnancy.
Interviewer	Well that all sounds great and I'm glad that you're feeling really well.
ADL09	Yeah
Interviewer	So um when you've looked for advice about exercising or nutrition and this can be from your current pregnancy or anything that you remember from previous ones, um are there any places that you've gone to for information or advice about prenatal health?
ADL09	Um, my friends really, um well with this pregnancy being in the study you've sent a lot of great um messages on Facebook and text messages which has been helpful. Um you know I don't know I feel like I know a lot fitness-wise to begin with so I kind of just adapt to it with what I can do being pregnant.
Interviewer	Mhm, did you ever have any concerns about what's safe or not safe?
ADL09	Um in the beginning I really wanted to continue running, I did for a little while, um and then I did ask my midwife about that several times and then when I felt like it didn't feel right I stopped and just walked so. Although I'm craving a good run.
Interviewer	Haha

ADL09	Like everyone on the road running, "I'm like Ohhh I Wanna be out there doing that!" and so it's hard.
Interviewer	Do you typically walk by yourself or do you have friends that go with you.
ADL09	Well my kids like to go on bike rides, so that's some of the walk but I usually like to go by myself like put music on and just go, so.
Interviewer	Some me time, to reenergize
ADL09	I just need that.
Interviewer	Um it sounds like you have good friend support and that the messages have been helpful, how would you say that the internet has played a role in your ability to stay healthy during pregnancy?
ADL09	Well it's just a fingertip away, you know it's just a touch away, and you find anything out that you wanna know, whether it's' true or not you kind of have to decipher that. It's pretty uh, I find it pretty convenient that I Can just look something up real quick, you know, especially with my iPhone.
Interviewer	IS there anything specific that you remember looking up during this pregnancy?
ADL09	Yep the running, um you mean anything or just to do with health and fitness?
Interviewer	Uhm, anything.
ADL09	Um blood pressure, numbers, um swelling, I looked up uh of course all my numbers because I'm 35, so the AMA have been a little abnormal so there's a time when my numbers came back high for the AFP. So course I was stressed and worried about that, looking online for everything I could about that. And my numbers for that um down syndrome were abnormal as well so. It's just if I had the test done like last year, when I was 34 I think the numbers would've been fine, but since I'm 35 they came back abnormal but baby's good so. That's good. Yes (14:22)
Interviewer	Awesome, um let's see so you said you often use your often use your phone for the internet?
ADL09	Yes.
Interviewer	You also have a home computer that you use occasionally?
ADL09	Well, uh no, I can't remember the last time I used my home laptop. It's been a long time, cause I have an iPad, my iPhone, my laptop, my work computer I can go on. I don't really use it at home.
Interviewer	Um let's see, in the times that you have searched for information online have you felt that particular sites keep popping up and that those have better information than other sites?
ADL09	Well, I like the birth uh the birth center comes up a lot.
Interviewer	Mhm

ADL09	Um, let's see, birth center like what's the med, um the mayo clinic one comes up a lot. And there's another one I can't, WebMD comes up a lot.
Interviewer	And how often would you say that you visited those sites during this pregnancy.
ADL09	Well I'm on the baby boards everyday um, not necessarily looking for information but just following the posts that people put on there so probably every day.
Interviewer	Um and so when we started talking about internet you had mentioned that sometimes you have to decide if a source has quality information or not quality information, do you have a way that you go about deciding which sites are gonna have good information or bad information?
ADL09	Well I tend to take what's on birth center with a grain of salt because it's mostly just moms posting on there with their experiences um and I also work with a bunch of nurses and doctors in my field so I can use them and I kind of you know kind of run things by them if I need to, but if I really have a medical related question like the blood pressure question I would go to you know the mayo clinic or web MD, or one of those that I feel are more trustworthy.
Interviewer	And what role do you feel that your midwife has played in this pregnancy you know that you feeling healthy, and better than maybe past pregnancies.
ADL09	I think I felt more supported with them, I feel like I could call them and ask them questions like with the nurses and midwives versus how I felt in previous practices. Because this is my third practice I've been to. This is by far my favorite, mostly because of the support and I feel like they, they're right on it, like you know if I e-mail them a question they respond quickly and if I call them they wouldn't be like "oh.." they don't brush you off which is nice. And I feel like that it's my third time around I'm not as you know worried or calling them about every little thing so that probably helps but I feel like if I did call them about every little thing they would still be supportive and um not brush me off you know, if that makes sense.
Interviewer	Yeah that makes sense. So next I wanted talk a little about the Facebook and the text messaging sites. So how helpful overall do you think that the Facebook and the text messaging programs were? 18:09
ADL09	Um I think it was nice that I got them, I didn't always open up the links or cause you know they come in the middle of the day and I'd be in the middle of something and I just didn't get the chance to look at them, but it was really nice knowing they were coming and I could if I needed to look them up and read them. And they're encouraging, it was kind of nice. You kind of felt like you had extra people, you know, kind of on your side.
Interviewer	Um so if you had to rate on a scale of 1 to 5 how helpful the messages were, where 1 is not helpful at all, and 5 is very helpful and 3 is kind of meh maybe it was maybe it wasn't

ADL09	If this had been my first pregnancy I think I would've like followed things to a T and read every little thing, um so I would say still say a 5 even though I didn't do that, because I can see how it could be really helpful with uh with the first pregnancy, with my third I just haven't had time to look them up, but it wasn't like "ugh a another message" it wasn't just another message. it was like "oh another message great, I'll have to read it later" you know what I mean so like, I think, yeah a 5.
Interviewer	You've got a lot going on so I can totally understand that.
ADL09	I think, but I think I Would've really appreciated it my first pregnancy. Like even more so.
Interviewer	Um so of the messages that you had time to look at, did any, do you feel like any sort of stick with you that you used the advice or you thought that it was something you hadn't known before.
ADL09	I think that the fitness ones that you sent out were the most helpful.
Interviewer	Okay
ADL09	And I think some of them were just encouraging, they were just, things that I kind of knew about different foods but you kind of forget you know or there was one time when you sent a message about water "I was like ah yeah I got to drink more water today" So that was helpful. I mean, that was one day but it still reminded me to go fill up my water bottle.
Interviewer	Um and then on the flipside what would you say was your least favorite part or the messages that were the least useful to you
ADL09	Only because I've been there before, some of the um the food ones and if I hadn't known about nutrition stuff before but I wouldn't say they weren't not useful, like just I kind of knew about them already but it was fine getting them still.
Interviewer	Okay.
ADL09	They're a good a reminder.
Interviewer	And so I know with Facebook it's harder to get those messages because you actually have to be logged in and some of these people don't have the auto messaging to remind them that they have something waiting, so how often do you think you check the text messages vs the Facebook messages.
ADL09	Oh texts I would get right away, I mean they're in my face. Facebook I think it's easier to overlook them. And I didn't really use the page to talk to other moms or anything which I think in hindsight I think would be good if I had questions about nutrition and stuff, which I didn't this time but I can see myself using that a lot the first time around.
Interviewer	And when you did read the Facebook messages, would you actually go to the page, or would you just read them from the newsfeed.

ADL09	If they were fitness related I would go, because some of the stuff I can just use anyways without being pregnant you know, always looking for different exercises to do or new ways to incorporate stuff at home because obviously with 4 kids I'm not going to be able to go to the gym a whole lot.
Interviewer	That makes sense
ADL09	Yeah
Interviewer	Um let's see, would you say that you changed your behaviors or beliefs about eating and fitness during pregnancy because of the messages?
ADL09	Uhm, probably not, no. I mean I'm more mindful of them but I wouldn’t say it necessarily changed.
Interviewer	So this is something that I think about a lot in terms of health regimens and um I'm just curious about what your thoughts are because pregnancy is a time where people seem more open to change and it's really hard to motivate people to, you know, join an exercise program or eat more fruits and vegetables, and what do you think it is, about pregnancy specifically that motivates women to make healthy changes.
ADL09	Well because you have something else growing in side of you, to have take care of someone else, it's not just about you anymore, it's about you know providing a good start to life for this baby that's growing inside you. (stuttering)
Interviewer	And so on days where you come home and you're just really tired and you haven't gotten in your walk, or you know you ate out more times than you would've liked, how do you make yourself do something still at the end of the day? Because I know that can be really hard.
ADL09	uhhh I can't say that I always do, haha.
Interviewer	That's okay.
ADL09	Uh yeah so that baby moving around, or I'll feel like "ugh if I sit down already, that's it for the end of the day. I need to keep going". So I, I just have it at back of my mind, “okay stay active. You'll feel better if you stay active.” That's kind of it, and that might not have to do with the baby, it’s more my body needs to feel good. My back can't be hurting, like I just can't get anything done when I'm hurting, so I stay active and I’ll feel better.
Interviewer	Okay um, I had something and it disappeared, haha. Oh so you had mentioned that the messages we sent were very positive and encouraging. Do you think that those are different from things that you have read or seen in other places?
ADL09	Well, I mean I don't have people telling me, you know people aren’t going to be like, "Oh you know, eat this for the baby; this is really good". That's why I thought they were very positive an encouraging, because otherwise you don’t really get that. Plus the thing is "you're a third time mom, you already know all this stuff". And some cases I do, but some cases I don't.

Interviewer	Did any of the messages change how you felt about your weight gain during your pregnancy?
ADL09	Um, I wouldn’t say that they the messages necessarily made me felt good about it, but I feel good about it in general about the weight gain because I feel like it's been, I've gained 18lbs and I feel good about that. Because last time around I was already at like 30, at this point. Of course I know I still have a month to go, so I could still gain weight, but um I think, I they just keep me mindful of that.
Interviewer	Um, did you have ever use any of the links about tracking weight gain or losing weight after?
ADL09	No I haven't.
Interviewer	Um, and then of all the different food messages that you received, did you try any of the ideas. I know you said that there was a day of a hydration method you refilled your water bottle, can you think of anything else.
ADL09	I've been doing the one you said about whole wheat pastas or something, we have it in our cupboard and we don't always make that, but I do tell my husband "Hey, there's a box of that, let’s make that". Like I said I'm mindful of that so the messages you sent were like "oh hey yeah we have that" so it was good for me.
Interviewer	Have you been taking prenatal vitamins regularly throughout pregnancy.
ADL09	Well no, I've been really bad about that, really bad actually. My iron levels came back a little low, so I started taking the Flintstone vitamins with iron. I'm just really bad, I don't know if it's because I'm lazy, I'm just really bad at taking medicine or pills or vitamins or anything even when I’m not pregnant. I know I really should be more considerate of that, being pregnant and taking my vitamins, but I haven't been.
Interviewer	Do you think that women can still get everything they need, if they have a healthy diet?
ADL09	Uh, maybe not the iron part, and I feel like I haven't, I mean it's not like I track my vitamins and stuff like that, but I get it through my food. So probably not unless you're really vigilant about it I don't think so.
Interviewer	Have you ever tried the gummy vitamins, they kind of taste like cake?
ADL09	Yeah that's what I thought, because I hate, well I mean I don't hate not swallowing pills, but I thought that those vitamins I would be more motivated to take, but it doesn’t really matter what form the vitamins are in, It’s just, I don’t know I just forget to take them or am just lazy about it.
Interviewer	well you're busy too, running all over the place.
ADL09	I know, but the Flintstone vitamins uhm I feel like my kids eat those, so when I give them to my kids I take mine too. I mean it's not a prenatal vitamin but at least it's some kind of, you know it's got the iron added, so I feel better about that.

Interviewer	Yeah every little bit counts so
ADL09	That's what I thought and you know, I'm not as tired this time and I actually feel really good, so I think before my iron levels were lower so I'm doing something I think that's helping my body.
Interviewer	Awesome. Um let's see, so kind of going back to physical activity, I know you were doing a lot before pregnancy and so can you give me an idea about how many days of the weeks, you were doing things at the beginning of pregnancy and how long you would exercise and how that's changed up till now.
ADL09	You know I feel like in the very beginning, I was just so tired and I really didn't do anything in the very beginning, I feel like I do a lot more now. Once the 2nd trimester hit I did a lot more than I did, you know, the first you know six weeks. I kind of gave myself, um, I don't know what's that word "permission" to relax a little bit to relax in the first trimester and afterwards I said "oh I felt good" so I needed to something.
Interviewer	Is the amount of physical activity that you do now, do you have a guide that you use as a recommendation of how much you should be doing each day or…
ADL09	No I just do whatever I can fit in and what feels good.
Interviewer	Yeah
ADL09	Yeah
Interviewer	And why do you enjoy, physical activity?
ADL09	Um, honestly I enjoy like the "me-time" that comes along with that and I can just escape everything else and kind of get in this zone. And it just makes me feel better. I actually like this pregnancy, I've had a lot of upper back pain. And the more I'm up, everyone's like "sit down! Sit down". I'm like, "No, I actually feel better standing up and moving around and being active versus than just sitting there". That's been motivating me to keep going, the other day if my back hurts or something or ughhh I know if I go for a walk, I'll feel better vs sitting down on the couch and watching TV or whatever, you know. 31:12
Interviewer	Um and so throughout pregnancy, your cellphone, it didn't change and it's the same phone you've had the whole time?
ADL09	Yeah
Interviewer	An iPhone I'm assuming.
ADL09	Yeah.
Interviewer	Oh have you joined any email/text message/ or any other internet pregnancy groups or messaging programs?
ADL09	No just uh, birth center board.

Interviewer	Okay and thinking about all the people, you know your midwife, the doctors that you work with, family friends, um even you know the study and internet sites, what place do you feel has the absolute best information on pregnancy if you needed to ask a question.
ADL09	Um, probably my midwife's.
Interviewer	And you were in Facebook and text messaging parts of the study and so sometimes you were getting 2 message a day, sometimes 1, you know we kind of switched it up a lot. Is there an amount of messages or posts that you think would be ideal?
ADL09	One or two a day is fine I think, because they come to you phone and they're on my phone anyways, so if I don't have time to read it, I just won't get to it and that's alright but I wouldn't want like 10 everyday, but you know the two is good.
Interviewer	Um and as far as who sends this information, um did you feel like the information that was sent, was quality information, or did you ever have concerns that it wasn't.
ADL09	No I thought it was quality stuff, I never thought "oh what the heck are they talking about" no.
Interviewer	And do you think a tool like this would be even more helpful and improve your relationships with say your midwife if the messages were coming from the clinic?
ADL09	Um yes, however I feel like I would want to write back and ask questions that you know I would ask them to open them up to a lot more time consuming, because I would probably ask them more questions instead of just nutrition or exercise stuff. But yeah I would love to have an open, being able to text my midwife anytime I wanted to. That would be great.
Interviewer	And so if you remember the, one of the primary goals of this study was to actually try to improve the health of pregnant adolescents because they haven't always have bet best social support at home, and they often have trouble making it to their appointments, so having more contact with them through social media is maybe one way you can improve at least the information that they're getting. So now that you were in a Facebook group and received text messages. Do you think one is better than the other for a teenage population?
ADL09	Yom know I think kids are like, connected to their phones all the time, so I probably feel like text messaging. Is something that would be, something they would use, I mean they're on Faceboo too so I don't know. Probably text messaging though, I think it would be more beneficial for them. And targeting even like first time moms, I think, because you know when I was a first time mom, I would soak up everything I could read or find about being pregnant. Because you're so excited and you have time to really invest, and research and look at all this stuff up because you wanted to know about this stuff. But being pregnant the 3rd time you're just like around I feel health so I'm probably alright. But like my sister in law she's pregnant she's younger,

	she's pregnant for the first time and this would be a great, she would appreciate something like this.
Interviewer	So what I hear you saying is that a lot of women, their first pregnancy they have a lot of concerns and want to make sure they're doing all the right things and um how do you think. I don't know do you think that if a message was sent that a first time mom would be more likely to make changes of we were saying "bananas" are the miracle fruit you should all be eating bananas that they would be.
ADL09	Yeah
Interviewer	Ok
ADL09	Yeah I do just because I think they, I mean not that I don't want the very best for myself and the baby but I have other kids to worry about and other things to do, but first time moms are so, but I'm just thinking about my experience and my friends but first time moms are just so eager to learn everything they can and so excited that I think if something is coming from trusted source they would soak that up, do you know what I mean?
Interviewer	Yeah. Do you think that first time moms enjoy having advice from women who have already been through the experience for pregnancy?
ADL09	Um,
Interviewer	So you know every pregnancy can be very different
ADL09	Do you know unsolicited advice or advice from how to raise a kid, but I mean stuff like that, I think they would be open to and learn about ways to keep health and how to lose that baby weight quickly. All that fitness stuff that I didn't know before, I would've really wanted to know all that.
Interviewer	And from all of your experience with um pregnancy and talking to friends and family, what do you think is the biggest motivator for women to want to want to be healthy. I know you said earlier that it's knowing that you have that baby to take care of too, but for if you think about like why would women would make these changes for themselves. What would motivate that?

ADL09	I mean just feeling better, you're not eating crap all day, you're not sitting on the couch all day, for me that's motiving that I'm going to feel better if I eat better and exercise and it's motivating for me to know that the more in shape I am, the better labor will and the more I'll be able to recover from labor afterwards. And get back to my not so wonder probably shape but better than pregnancy shape you know.
Interviewer	um well I think those are all the questions I had for you, but I just wanted to check is there anything that we talked about earlier that you though of another answer to, or what to go back to and talk more about?
ADL09	Nope I do have a question like, at what point you will know, once I have the baby, will I be taken off the fb page, or what if I…
Interviewer	I did' get that I'm sorry, my phone cut off sorry.
ADL09	Oh yeah mine's cutting out too, um so the Facebook site, and the nutrition questions, can I still go to it afterwards? After I've had the baby, or is it just strictly while I'm pregnant.
Interviewer	So the fb group, the agreement with, so we have a review board that makes sure that we follow you know all the rules to treat participants well so their thing is, they're very into internet safety and so once uhm the Facebook side, once you have done this inverview and completed your surveys, then you've completed the study and for safety reasons we're supposed to remove you from the fb group so your would not be able to see the messages after that point but anything you've received in text, you would be able to look up still.
ADL09	Okay
Interviewer	And if, if you wanted a list of the, all the messages that you've received, I can actually send that to the study recruiter and have it printed off you and that way you would have everything that you got on fb as well.
ADL09	Okay. I think that would be helpful
Interviewer	Okay I'll write myself a note to do that.
ADL09	Okay
Interviewer	Um, any other questions?
ADL09	I don't think so.
Interviewer	Alright well, I will check with the study recruiter about visiting you on Tuesday to finish up everything and I'll have her bring those messages for you, and you should get 2 $10 cards, and 1 $30 card. Um, cause I think you have a diet record left to do, and a nutrition survey and each of those are $10 and $30 for the interview.
ADL09	Yeah I've been waiting for her to corner me you know "oh what did you eat the last 24 hours" and I'd have to be like "oh no" I haven't done that in awhile, but thank you so much
Interviewer	I hope everything goes well with the delivery and the little ones enjoy having a baby brother or sister.

ADL09	I think so, thank you so much, Interviewer.
Interviewer	Alright bye bye
ADL09	Bye bye.

ADL10

2:46
Interviewer: It's been a little bit since we've talked, and I wanted to catch on how things have been going. Uhm, so can you just tell me a little bit about how your pregnancy has been since earlier?

ADL10: Uhm, it's, it's been the same. Uhm, nothing really changed as far as the pregnancy goes at all, except the baby growing. (Laughs)

Interviewer: (Laughs)

ADL10: It's getting bigger, but other than that, nothing really changed

Interviewer: What would you say has been the best part of this pregnancy?

ADL10: Uhm, seeing my baby growing and feeling him move and stuff. It's been the best thing ever. I just was telling, uhm, what's her name?

Interviewer: [name redacted]?

ADL10: [name redacted], I was gonna call her [name redacted]. The other- I was gonna call her [name redacted]. [name redacted], I was just telling [name redacted] that it's been a real good pregnancy for me, and I haven't had like no problems or complications with him or nothing. And how people be over exaggerating with the babies and how they make them feel. Well probably not over exaggerating, but my situation is way different than other people. It's been like real good for me.

Interviewer: That's awesome! That's really good to hear.

ADL10: Mhm.

Interviewer: Uhm, so the last time that we talked, I had asked if you have had any hopes and dreams for your baby to be, and uhm, you said you haven't really thought a lot about it, but now that things have gotten closer to the delivery, have you thought about the future at all and what things will be like for your little one?

ADL10: Not really, I still… It's still like uhm unbelievable probably to me. I don't know, I think it's probably like I don't know it's probably unbelievable until I see him, you know? I is. I don't know, I don't know. It just don't seem real. I don't know but it don't seem like a baby is really gonna be here and nest in like a in like a month and a half. Like… I don't know. But I just think about the things like when I see him and how and how I'm gonna react. You know, I don't know. It's my first time going through it at all. I never thought about nothing about it—about…

Interviewer: Sometimes taking it one day at a time is best way in that situation, so, you know. Especially when it's your first pregnancy and everything.

ADL10: Yeah, yeah.

Interviewer: Have you thought about names at all?

ADL10: Yeah I'm gonna name him after his dad. His name is gonna be [name redacted].

Interviewer: Oh that's really cute. I like that a lot

ADL10: Yeah

Interviewer: Uhm, so kinda changing topics and switching over to nutrition. Have you changed the way that you've prepared food at all since you've became pregnant.

5:45
ADL10: Uhm, it's the same as I was telling you. I baked—I baked for uhm, sauté it, sometimes I do fry foods still because I'm used to doing that before. Uhm, yeah it's the same as last time when I was talking to you.

Interviewer: Are there any foods that you added because you heard that they had benefits for pregnancy?

ADL10: No but I still eat the same stuff, all the time. I'm addicted to chicken. So the only thing I've been eating a lot since I've been pregnant is chicken. Chicken. Chicken, I like steak. My doctor told me too about steak. She told me it has uhm, what did she tell me about red meat? I forgot what she told me about the red meats, but uhm she told me to eat like steak and stuff that it was good for the baby or red meat. And chicken and steak is one of my favorites so uhm.

Interviewer: And were those favorites before pregnancy as well?

ADL10: Yeah, so nothing changed.

Interviewer: Okay. Were there any foods that you liked before that you just couldn't really stand once you became pregnant?

ADL10: No, that's weird, but you know one thing I didn't know is shrimp. I like shrimp still but the alfredo smell gets to me and when I like go to like Red Lobster and I smell the like alfredo. I used to get the little meal with the alfredo, but now I substitute it cause I can't stand the smell of it. And when I was at work, I tried to uhm eat the alfredo, and when I went home from work that was like my first time throwing up since I've been pregnant, from the alfredo sauce, and it wasn't even shrimp, it was chicken alfredo but it was just the sauce smell that got to me and I threw it all up, and that was the first time in my whole

pregnancy, so I know it was because of that. So the only thing that I liked before that I noticed I don't like now.

Interviewer: Mhm

ADL10: Mhm

Interviewer: Uhm, how much dairy food have you been consuming during your pregnancy?

ADL10: Uhm, I still do the same, but milk , I do like cheese, and yogurt, like stuff like that. I don't know how much would I say.

8:22
Interviewer: And of all the things that you've uhm eaten throughout pregnancy are there any that you felt were the best for your baby in making sure that they were born healthy?

ADL10: Yeah like you just said. I like the milk and stuff for the calcium for the bones and stuff. Uhm, like the type of cereal I eat… with the uhm what is it? The oats and honey cereal. I've been eating it's basically like what I said last time basically what they supply with the WIC stuff, the WIC cereals, the Cheerios, and corn flakes, honey and oats, any type of cereals, and mm… fruit like apples and stuff like that.

Interviewer: How has your vegetable consumption been?

ADL10: I still like only broccoli. I don't eat other vegetables so, but I choose broccoli with basically with everything. I don't like salad, I don't like cauliflower, mixed vegetables. I never was like that. I never liked vegetables like that. I just eat broccoli. Broccoli, corn. Which corn? I don't know if corn really counts as a vegetable vegetable, and corn.

Interviewer: Uhm, have you ever used the internet to look up information about food and nutrition at all?

ADL10: Uhm, I see it like going past my apps and stuff but not just like going there and search the food, I really don't. But I do do it if was to hear something. Like before when I was hearing about seafood, I would search that for what harm it could do to the baby and stuff. But not no… I just don't go there and see basically got. It's just too see what's harmful for the baby, like the deadly meat. I heard stuff about the deadly meat. I would look stuff like that up to see what causes and risks its takes. Other than that, no I just don't look up food.

Interviewer: Uhm so when you did look up the information like that, were there any particular sites that you remember going to because you felt they had good information?

ADL10: No I just go to Google and check on them and click on the sites. They usually have the same information. And like I said before, the doctor gave me a book… the book and it

tell you too what foods are harmful. And a packet too they gave me, and basically got the same information that the Internet had.

Interviewer: Uhm, so you mentioned that you had some apps that you've been using. Can you tell me a little bit more about those?

ADL10: Uhm, I have baby bump, which is a daily one. It's a daily one that gives you daily uhm daily things on the baby like the growth and stuff. And I have weekly ones too. I mean the baby bump got a daily and a weekly, I'm sorry. And uhm it tells you like the baby growth, what the baby should be doing, it also tells you like what type of foods and stuff to eat that would be good. Uhm, its just an app that's about the baby and your body and stuff. I have that one, I have "I'm Expecting" and I have another one that I just recently deleted because they were basically telling me the same thing as the other one. It just filled up the space. And they both, basically, they all the do same thing with the baby and tell me about the baby and stuff and tell me how long I got left and the calendar and little fun little facts. And then the Baby Gaga is a website I go to. It's not uhm app that I got, I just visit the website.

Interviewer: And is that one uhm more like a forum where women share their own stories or is it information?

ADL10: Oh the apps that I got got forums on there too.

Interviewer: Mhm

ADL10: But uhm, Baby Gaga is the same thing. It got forums on there and it also tells you the weekly and the daily stuff about pregnancy. But yeah this one has forums uhm a lot of forums on there too. I look I look at some of the stuff but I never uhm comment on there. I just look at the ones the people like me in their third trimester. The same as me, and see what they're saying.

Interviewer: Mhm. Uhm, so you were a part of the Facebook group in the study-

ADL10: Mhm

Interviewer: -and uhm how often would you say that you looked at the Facebook site?

ADL10: I wouldn't even look at it as much, but it was half of the time the stuff would pop on my newsfeed and I would like scroll at it but I never really went on the site on the on the page and wrote anything on there but your messages. Are you the one who sends the messages or is it just like a somebody like a generated thing?

Interviewer: Uhm, we have like a team who posts the messages.

ADL10: The messages on the phone I pay attention to daily when y'all send them, but not the Facebook thing. But I notice sometimes that they be the same sometimes. Like the things that uhm you put on the messages-

Interviewer: Mhm

ADL10: -will be sometimes the same as the things on the Facebook.

Interviewer: Same topics or exactly the same?

ADL10: Same topic. Topics and stuff

Interviewer: Okay, uhm, were there any messages that really stood out to you that made you try something new or you learned something that you didn't already know

ADL10: Uhm, it was one of them before with uhm I think it was talking about the different food categories and stuff uhm I forgot what exactly it was saying but that one I really liked to go and I was talking to my friend at work cause he's going to school to be a nurse and he was telling me more about uhm the food and like how what it does for your body and stuff. Cause some of that stuff I never paid attention to even before being pregnant. Honestly I've never paid attention to what it does, what it does for you, or how it makes you better.

Interviewer: Does it make you think differently about any of things you were already eating?

ADL10: Uhm, not really. No, it really didn't.

Interviewer: Uhm, and out of all the messages you received, were there more topics you enjoyed reading about more than others?

ADL10: Hm no, no. All of it was helpful information though, so it wasn't like a topic just that I liked it better. All of it was helpful.

Interviewer: And along those lines, you received a lot of different types of messages, sometimes they were short sentences, others times they were sentences with a web link, or a picture, or a video. Were there a type of message that you found the most helpful?

ADL10: I've never seen a video from you. I've seen pictures, but I've never seen a video unless they wasn't coming through.

Interviewer: Ok

ADL10: I've never seen a video on the phone. But the pictures--the pictures was helpful. I liked the pictures. But yeah I never got a video, unless I had to click on something, I never got it.

Interviewer: Uhm, I think they would have come through they it would have been a link you would have to click on.

ADL10: Okay

Interviewer: It wouldn't actually have popped up the video unless you clicked on it. Did you ever click on any of the links?

ADL10: Yeah but not that one. I never seen no videos on the phone. I only see the like the website you know weblinks, but never a video one on there. I've never seen videos on there. Unless I missed them.

Interviewer: Uhm, do you remember which links that you did click on?

ADL10: Hmm, no not really. I still got every messages on there cause I don't delete them. I still got all of them on here, hold on.

Interviewer: Okay

ADL10: Yeah I still got all of them on my phone. I could probably tell you which one I was talking about too with the food. That I was just saying. Oh yeah and the other day, when they text about the caffeine, I kinda laughed at that one cause I just had a little, a little caffeine before, before uhm the message came. But that was helpful about that with the caffeine thing too when I've seen it because when you said before 3 pm for a good night sleep because one time I had drunk caffeine and it was real late and I was up all night long.

Interviewer: Oh no!

18:37

ADL10: Me I was up and the baby was up that night. He kept moving and stuff, so. Yeah, another thing too about the meals which I had already knew about that I had paid attention to more about the snacks and stuff. But yeah see the ones that with the yoga and stuff I never click it I never click on there so if it was like a video on that one I never clicked on them

Interviewer: Oh yeah. I think those ones--a few of them were videos.

ADL10: Yeah, yeah I've never seen them.

Interviewer: Uhm, so about the messages that had to do with physical activity or exercise, how di-

ADL10: Mhm

Interviewer: What did you think of those messages?

ADL10: Just the same things that I've knew. The doctors telling me or me seeing in my book that I have.

Interviewer: Mhm

ADL10: The stuff that I knew already

Interviewer: Uhm, did you try any of the suggestions at all?

ADL10: No, I didn't. I be trying to make I've been lazy honesty I be trying tell myself to go to the cause I have a little gym in my uhm complex that I live in. I've been trying to tell myself to go there more and walk the treadmill and stuff, but I've just been being lazy. Like when I'm off of work, I'll just be home or not doing too much, so.

Interviewer: Yeah, have you been tired?

ADL10: Yeah. I have been, but before I was pregnant, I used to be sleepy a lot, and I don't know why I'm just like that so I do sleep a lot. I can pick up and be tired like in less than an hour. And I can sleep like a lot all day if I don't have too much to do.

Interviewer: Did that get, uhm, worse as your pregnancy progressed?

ADL10: Uhm, it's stopped like in the middle, and now like it's getting back to it now. Probably because the baby is getting bigger and stuff

Interviewer: Mhm

ADL10: And and and in my book it told that uhm, usually the last couple of weeks and stuff you get tired like you was in the first week and stuff. I probably feel like that.

Interviewer: Uhm, so I know that you said that you've been tired. But can you tell me a little bit about what a typical day looks like for you in terms of moving around and when you're moving around and-

ADL10: Uhm. Well… majority of the time I work, I work uhm five days of the week and I work with people with developmental disabilities so I stay like up and moving when I'm really at work and stuff I'll take a walk in the community and go shopping you know like I walk and stuff at work, and if I'm home on my days up and if I get up I'll go places but I usually don't just like go walking and doing stuff I just stay active during the day, when I am up. But yeah, the most walking is stuff I do probably now if I'm at work and stuff which is five days out of the week when I'm at work and I work like 9 hours, 9 hours a day. My shortest day is on Wednesday, which 6 hours so I work like 9 hours the other days.

Interviewer: Uhm, the messages that you received, did those change how you thought about the weight gain you were experiencing during pregnancy?

ADL10: Uhm, yeah it has. But my weight gain. I thought I was honestly gonna be worst gonna be worst than what I actually am because through the whole thing so far, last time when I needed when I need to pay attention this time because last time she didn't tell me, you didn't tell me. Last time I went it was like around, 16 pounds, 17, 16 pounds that I have gained. And usually when I share my information with people that have been pregnant, they always tell me that I'm good cause they would gain like double or triple because they used to eat everything. And I don't find myself, I told you I eat the same, I don't eat more, or I don't eat extra things that I don't feel like I'm hungry, you know? I don't feel like oh I'm eating for two. Like my doctor also told me too that, uhm, you really don't eat for two it's just being in people head and they think like that just because it's a baby and they were eat. But no I don't overeat and stuff so I've been I think I've been alright with my weight gain as far like what it thought I was going to be, so like and every time I go to the doctor, it be like a pound or two I didnt have like a high gain or anything like that.

Interviewer: That's good

ADL10: The most I've gained since I went there was for in one month and that caught me by surprise was like I think like six or seven pounds and that that's when a few pounds like I've been told you it's been like 16 and 17, a lot of them came from one time when I went so I don't know what I did that month but after she told me that's when I was drinking like uhm pop and stuff

Interviewer: Mhm

ADL10: I stopped doing that and that's when I started doing the one or two pounds or gaining like bit at a time. So I stopped with the sugar and all the pop.

24:50
Interviewer: Uhm, uhm, In terms of weight gain too did you ever use any of the resources that were provided and message about tracking your weight gain online?

ADL10: No, I didn't, because I had I had stuff at home too with the uhm that I could check my weight gain and the apps that I have but I just never I-

Interviewer: Okay

ADL10: And the reason why I don't track it on like paper because when I go to the doctor they give me uhm my sheet of paper and they tell me like how much I gained on that paper like you know just like the times that I've been there so I already know what I've been gaining and stuff so I never track it online or in my books that I do have at home.

Interviewer: Mhm

ADL10: Because I already got that information if I do need to go back and look at it

Interviewer: Uhm, and in terms of eating habits… were did you ever have any cravings for non-food times like powders or dirt?

ADL10: No

Interviewer: Okay. Uhm, who basically does the shopping for your groceries?

ADL10: Me, the same like last time. Only me… I live I live by myself, so its only me.

Interviewer: Uhm and how much would you say on spend on an average each week on groceries?

ADL10: Uhm, I don't go weekly I go monthly so… uhm maybe like one something I don't know exactly how much but it be like one something.

Interviewer: Okay.

ADL10: When I ran out of some last month, you know.

Interviewer: Uhm, and how do you normally access the Internet?

ADL10: On my phone, or from at work, I get on a computer.

Interviewer: And do you have a home computer as well?

ADL10: I have a laptop but my screen… my screen is cracked and I never got it fixed because my phone do just as much.

Interviewer: Okay. Uhm, did any of the messages that you received make you change how you felt about certain types of food, like whole grains or fiber or anything like that?

ADL10: Mmm, not really because like I said before with the uhm the cereals and stuff I uhm… the most things that I be eating with the whole grains like with the bread with uhm the wheat bread the cereals and stuff that I've been eaten I've been eaten it before the messages came through with the WIC stuff. Then I get on with that's the things that they supply. I like the wheat bread and I like the cereals with the whole grains. You know them types of things.

Interviewer: And so you listed a couple phone apps that you have and just to kind of cover all of our bases, did you join any email list serves or anything where you get pregnancy information through email?

ADL10: Uhm, those apps… those apps email me too and the Baby Gaga website that I told you-

Interviewer: Mhm

ADL10: And they uhm are websites that email me but I honestly don't remember the name of it cause it's some parenting, I forgot the name of it, but yeah.

Interviewer: And do you follow any other pregnancy websites or blogs and Facebook groups?

ADL10: Mm-mm (No)

Interviewer: Okay, and so thinking about all of your experiences during pregnancy is there one Internet website or app or a person that you feel gives you the best pregnancy and nutrition help and information?

ADL10: Um, no they all be the same honestly. They all giving me the same information. Like I said they usually give me how I'm progressing in the pregnancy. And they usually be the same stuff with that, with the food and stuff. Yeah they're the same.

Interviewer: Uhm, and comparing the number of messages that you've got in the study with the number of messages that you're getting from your phone apps via email, do you think that there's a good number of messages that you should get daily, weekly, or how often?

ADL10: Uhm, like you mean, how many messages I should get daily? Or –

Interviewer: Yeah, like is there a point at which it's just too many?

ADL10: No not really cause all of the information do be helpful. And like I said with some of the messages that you sent me, I knew some of them already because of the apps or whatever I'm looking at on the internet, so, it still was like helpful seeing it though… seeing that everybody is the same. You know like all of the information I be getting is the same and none of it was different.

Interviewer: Uhm, do you think something like the Facebook group or the text messaging program would be a good thing for you health care provider to have available to all pregnant woman?

31:11
ADL10: Yes, the text messages because I pay way more attention to them then the, um, facebook ones. Yup, I like the text messages one. I think more people anyways would pay attention more to the messages on the phone.

Interviewer: Mhm. Was there any information that you thought was more helpful than other topics?

ADL10: No, not really. I just thought it was all helpful. None of it was useless information; all of it was helpful.

Interviewer: Uhm, is there anything else that you thought about the messages that I didn't ask you about that you would like to share?

ADL10: Uhm, no. I told you, this was all helpful and I know I like the messages that you all did provide.

Interviewer: Mhm

ADL10: I don't think you all need to add anything or you know there's nothing bad to say about it at all. Everything was good with the messages. All that information was good information.

Interviewer: Uhm, so one thing that you mentioned earlier on was that you had felt tired and maybe haven't exercised as much as you would have liked to. I was just curious if there was anything we could have done to motivate you to exercise more?

ADL10: (Giggle) No, it's not y'all. It's just me.

Interviewer: (Laughs)

ADL10: (Laughs) It's not y'all. Everybody be saying the same thing. My doctors tell me, my apps tell me, the Internet tell me. It's not y'all. It's just me. Even before I was pregnant, it's just me. Its not it has nothing to do with or what y'all were telling me.

Interviewer: No, I know it's hard cause you're tired. I do it too. I go home and I sit down and I say "I should exercise, but I know I'm not going to."

ADL10: Yup.

Interviewer: It's really hard to figure out what motivates people.

ADL10: And then I tell myself, "ok well I'm at the last couple weeks, at least let me walk more, cause they do say when you walk and stuff it help the baby come, comes quicker and you know basically it won't be lazy and stuff during, uhm, during the final couple of weeks or when the baby ready to come they tell you basically to walk more, so…

Interviewer: Mhm

ADL10: But I still haven't did it. And I need to to really really need to start to do it. I'll see how it works. Maybe when I hit 34 weeks, or when I hit 36 weeks when I only have a month left, I'll probably try to start it.

Interviewer: Do you think it would be helpful if you had kind of like a cheerleader or friend to come and get out and drag you out?

ADL10: Yeah, yeah it would be very helpful, it would be way helpful. Cause even before I had uhm a membership that I paid for for a year, and it would be better if I go with somebody have somebody to talk to. I don't like going by myself.

Interviewer: Mhm

ADL10: I think it could be better to say come on and get me up to go, it would be better.

Interviewer: Is there anyone in your network of family or friends right now that could be that person?

ADL10: Mmm, if they could be that person they should be doing it, so. Probably not, they all say the same thing I'm sayin they all wanna do it but they don't do it, so

Interviewer: Yeah.

ADL10: Yeah.

Interviewer: It's hard. It definitely is hard

ADL10: Mhm

Interviewer: Is there anything else that we talked about that you've thought of an additional answer that you want to go back to and discuss a little bit more?

ADL10: No, I think I've said everything. I think I've said everything so far, about, yeah I think I've said everything. Mm… I don't think I have nothing else to say. But it's funny cause every time when uhm people ask do you got anything else to say then later on you think about things you know that pop up in your head, but right now I can't think of anything else I have to say about it.

Interviewer: Well, I just have one more question then. Uhm so we hear lots of advice about things to do while you're pregnant, have a healthy baby, or to make the delivery easier, uhm things to eat, ways to exercise. How important do you think it is that you do all of those things to actually have a healthy baby?

ADL10: I think it's very important, but it still be hard. But at the same time people say with the things they eat and with the baby growth and stuff like that cause even if you're not doing that as often cause like I said with the exercise and the stuff like that I'm still fine cause when they measure the baby or hear the baby the baby still has a very strong heartbeat.

Interviewer: Mhm

ADL10: Every time I went nothing was ever wrong and I'm doing very good so when they tell those things I'm like okay, well... You know, I don't know it's good but then it's like I

don't know accurate it honestly is with the baby, like the strong baby or the healthy baby because like my baby is perfectly fine with some of the stuff that I don't do, so…

Interviewer: Mhm, yeah that's a good point.

ADL10: Yeah it could be perfectly fine, some people tell me they drunk caffeine like the doctors tell you not to drink caffeine as often and that it's bad for the baby and the heart rate and all that stuff. I was scared to drink caffeine until a little while ago. I would drink a little here and there. But I won't drink a lot of it uhm some of my friend that have been pregnant or some that are pregnant now say they drink caffeine every day and they did the whole time and it never did nothing and their babies came out perfectly fine. You know its just things that people say that don't really be true or accurate.

Interviewer: Do you think that people just say that because they want to create the best case scenario where if you did all of these thing it will for sure be ok, or?

ADL10: I don't know. I don't know. I just think honestly they take risks and they're okay. I know I don't do it with the caffeine and all the other things that you know you're not supposed to do. I won't do it, I will be paranoid and like if I was to drink a little bit of caffeine right now and look up on the internet… I will look up on the internet how much caffeine or what caffeine do to the baby. You know, I would look stuff like that up.

Interviewer: Mhm

ADL10: And it basically do what it do to you to the baby, so that's why I really won't do it. But like my doctor told me when I was there last month I had a littlw scare I hadn't felt the baby in a while so I went to the doctor and she told me all the things to do know and she said that would be the only time she gonna say it but if I don't feel the baby moving drink a little caffeine and it will get it moving, you know?

Interviewer: Mhm

ADL10: So she told me it would really get the baby moving. Don't do it often but, just sometimes it's okay. A little bit won't hurt, so that's one thing that scared me and one thing I did, but other than that with the baby being healthy, I think the baby is perfectly fine as long as… I don't know you know you could be just fine cause like I said my baby's just fine.

Interviewer: Yeah

ADL10: Mhm

Interviewer: It makes sense. Uhm, well I think that was all that I had to talk to you about, so unless you have anything else to say, then I guess we're all done.

ADL10: Okay, and it seems like you just froze up cause the screen just froze right now.

Interviewer: Oh, can you still hear me?

ADL10: I can still hear you but, yeah okay, it's going slow like with your uhm, your face.

Interviewer: (laughs) Um, I think, so we're all done with the interview and then you just have the two surveys left: the diet record and then short nutrition questionnaire that you filled out at the beginning of the study.

ADL10: Okay

Interviewer: Uhm, and so I just texted [name redacted] to let her know that we're all done and she'll come down and then do those two things with you. And just so we're on the same page, and you don't get shorted uhm any gift cards, you get $30 for the interview, and then you get $10 for each survey, so you should have a total of $50 when you leave, so…

ADL10: Thank you again. Sounds good, thank you.

Interviewer: Yeah you're welcome. Um, I'll just hang out until she gets down there but it sounds like things are going really well, that's so exciting.

ADL10: Yeah, it is, and it's almost over. I just told you like I told you I just hope these last couple week that's I have won't be bad.

Interviewer: No, I don't think so, so. Well I just wanna wish you good luck with the delivery and send all my well wishes for lots of fun afterwards.

ADL10: Thank you.

Interviewer: You're welcome. And just make sure you do both surveys with [name redacted] and then you'll be all done with the study.

ADL10: Okay thank you for all the information you gave me.

Interviewer: Yeah, thank you for participating. We appreciate it.

ADL10: You're welcome.

Interviewer: Alright, well have a great rest of the week, and enjoy the fourth of July weekend coming up.

ADL10: Okay, you too.

Interviewer: Yup, okay bye.

ADL10: Bye.

ADL11

INTERVIEWER: So the plan for this interview is to sort of touch up on the same topics from last time, so uhm, how you've been eating, if you've changed anything, what your physical activity's been like if that's changed at all… uhm since early pregnancy. And then also uhm the big differences talking about the messages that you've got and then how those those have influenced any changes that you've made or how they make you feel. Those shorts of things. So, no right or wrong answer. It's all about how you feel and what you think. So uhm, don't be shy or afraid to hurt my feelings or anything like that.

Both: (laugh)

INTERVIEWER: And uhm normally the interview's supposed to take about an hour, but it's not been taking that long. So, just for a time frame if that's been helpful. Its been 45 minutes on average, so…

ADL: Sounds good

INTERVIEWER: do you have any questions before we start?

ADL: Nope

INTERVIEWER: So its obviously been a little while since we've last talked, and so it's always good to catch up. And… so I'm curious how your pregnancy's been going since we last talked?

2:45

ADL: Uhm, it's much better (laughs)

INTERVIEWER: Oh, that's good.

ADL: Yeah, I've a lot more energy, and I don't have heartburn anymore. Uhm, my appetite came back. (Laughs) So yeah I just feel a lot better than I did my first trimester.

INTERVIEWER: That's great.

ADL: When did we meet last? I think I was still in my first trimester.

INTERVIEWER: Yeah, yeah. I think you were right at 12… somewhere between 12 and 13 weeks so it was really pretty early.

ADL: Yeah. (laughs)

INTERVIEWER: Uhm, so what would you say is the best part of your pregnancy so far?

ADL: Uhm, feeling our baby's kick. That's really neat.

INTERVIEWER: I bet.

3:33
ADL: Last night… I sometimes felt like, kicks, but last night I felt a hand going across my stomach… it was just weird. It was cool, but it was weird. (laughs)

INTERVIEWER: (laughs) that is really cool. And then last time we talked, I asked what hopes and dreams you had for your baby, and so I just want to ask the same question again.

ADL: Uhm… Yeah I think just similar. Like I hope we have a healthy baby who you know is happy in life and, I don't know, that's a hard question. (laughs)

INTERVIEWER: Yes, it is, and those are the answers that most people give.

ADL: I just want to go through the delivery (laughs)

INTERVIEWER: That also would be… that's a good good hope. Uhm, so those are kind of just the intro questions um so… the next thing I wanted to talk about is eating so uhm. It sounds you've had some improvements there, uhm with how you've been feeling. So can you tell me a little bit about how things have changed and what things you've added, removed from your diet… anything like that.

ADL: Yeah, uhm. I definitely have an appetite, which is great. Uhm, I've definitely noticed tat I can't eat big meals, like I kinda have to eat a little all day. Just cause I feel kind of, well it kinda (mutters) It started out that I would feel full really quickly, and I don't feel full quickly anymore. But uhm, I need variety. Like I get sick of what I'm eating. It's really strange because I'll be eating something and I'll be like, this is so good, and then I would really like, put it down and like, uhmmmm… I'm done (laughs) But I'm still hungry so I have to go find something else. I'm a total pain in the neck. So I feel it's like, uhm, yeah. (mumbles)

INTERVIEWER: Have you started eating any foods that you didn't typically eat before pregnancy?

ADL: Uhm I've strated eating a lot more meat. Uhm, I really only eat fish before. But uhm, yeah like I don't really make much meat at home, but when I'm out now… Like I don't want to order like a burger because that's too much of one thing, but like if we're ordering appetizers, like maybe I'll have like a piece of chicken. You know, like something small. Uhm, the only new thing… my friend just introduced me to kefir, that yogurt drink. And it's really good. And I've never had it before. For some reason I thought it was goat's milk and I just thought that would be gross, but it's not. It's really tasty and y'know, you can drink your nutrients… which is nice (laughs). Uhm so yeah, I started drinking that and I like it.

6:54

INTERVIEWER: Yeah, I like that stuff, too. It is really good.

ADL: Mhm.

INTERVIEWER: Uhm, so. That's obviously a dairy product. So are you having trouble with like other sorts of dairy products that aren't fermented like milk and, uhm, some cheeses?

ADL: No, I eat a lot of cheese (laughs).

Interviewer: Okay

ADL: It also goes that it's so easy because I'm eating all the time and I go… Ah! Cheese! Great.

INTERVIEWER: Yeah… and have you had any cravings for things that maybe like didn't like before or didn't eat a lot of? Other than the meat.

ADL: Uhm… I mean I eat a lot of fruit now. I mean, I ate fruit before, but I eat fruit like all the time now. I think that's because I'm always grazing, and that's something that's quick and easy and healthy, uhm, oh and also it being hot out. Y'know, it's going to be cold… I don't have to cook it. So, but, I ate fruit before, but I eat a lot now.

INTERVIEWER: Uhm, okay. And any non food cravings like dirt, uhm baby powder…

8:20
ADL: Nah. Uhm, no (laughs)

INTERVIEWER: Uhm, are there any smells you like more or less at this point in your pregnancy?

ADL: Not really. My first trimester, I didn't, uhm strong smells I could not take. But now uhm not that I've noticed so far.

INTERVIEWER: Okay. And have you felt like you've had any challenges with food since trimester one?

ADL: Uhm, I've noticed that I've started gagging more on my prenatal vitamins. Is that food? (laughs)

INTERVIEWER: Yeah, sure. That counts in that area, I think.

ADL: Uhm, any trouble… no, besides that sometimes I'll be something that's good and suddenly I want something else. So it's not like a full-on aversion but I just get sick of things easily.

INTERVIEWER: And did you have any trouble finding something to fill in that… like if you were liking it and didn't like it, is it hard to switch to something else or it's pretty easy to find something else to start eating?

ADL: Uhm, it's pretty easy. It's like when I food shop now, I just buy snacks (laughs). I don't actually like, buy an actual, like food for recipe just cause I know I need like quick and easy food that I can eat all day (laughs)

INTERVIEWER: And are you the one who's doing most of the shopping and preparing food?

10:22
ADL: Uhm, I would say I do most of the shopping, but my husband has started making more the food.

INTERVIEWER: Okay.

ADL: Last night he made me a kale strawberry smoothie. It was really good.

INTERVIEWER: Oh, that sounds interesting. I've never actually had kale in a smoothie, but I know that a lot of recipes call for it.

ADL: Yeah, I haven't really had it either but my friends swear by it. I think it sounds gross, but to me it was good. I think you have to make sure you chop it up really fine.

INTERVIEWER: Mhm. Well I'll have to try it (laughs)

ADL: Yeah! (laughs)

INTERVIEWER: Uhm, let's see, um, and so this next question is a loaded question, I think, and it's mostly because of my lack of ability to word it any other way (laughs). So I'll preface it with a lot of women purposely change or make changes during pregnancy because it's a time where you're influenced by wanting your baby to be healthy. And so the question is, what things did you knowingly change throughout your pregnancy to make sure that your baby was born healthy?

ADL: Uhm, well I didn't I stopped drinking completely. I never smoked or anything. Uhm, I guess probably eating meat was the biggest change. Like I pretty much had, like I never had, other than fish, eaten meat since I was in my early teens. So I think once I realized how much protein I needed and how hard it was for me to get, especially during my first trimester when I had no appetite. Uhm yeah, I started eating more meat. Uhm, I'm trying to think if there's anything else. I think I'm more consistent uhm, if I'm at work and I'm tired and I plan to go to the gym afterward but I'm tired and I don't want to do it and I'm pregnant, so… I should go home and take a nap. (laughs) So I'm better at not doing that and just forcing myself to the gym because I know ultimately it's good.

INTERVIEWER: Uhm and…

12:51
ADL: But I guess when I exercise now, I'll make sure I don't pull a muscle. You know cause I'm more susceptible to that. …But yeah. It's weird because a lot of people are like, "Oh, you're eating for two. You're pregnant. You need to relax." Which is…, I mean I'm not eating for two, but I do need to relax. But yeah, I feel like it's the opposite. Because I'm pregnant and I need to make sure…. Like my fetus doesn't need cake. I don't eat cake. I need to exercise a little bit every day if I can so. I guess I'm just more mindful of that stuff.

INTERVIEWER: And where are you typically looking for advice about nutrition or uhm, physical activity during pregnancy when you do have questions?

ADL: Uhm… well we have an app. I want to say it's the BabyCenter app, maybe. So that's kind of neat because it gives you like weekly updates of where the baby is, where you are, and just like stuff you might be dealing with. So that's kind of neat. Uhm… I ask my midwife… Uhm I think I ask friends who I think have a similar lifestyle or approach, and I do who have had kids. I use my friend who's been pregnant a few times who introduced me to the kefir. Uhm… who else? I get your texts (laughs). So yeah, that's probably about it. Sometimes I'll look up the nutrition content of a food like online. Like if I'm just curious. If I'm eating something and I'm wondering how much protein or whatever is in it.

15:02

INTERVIEWER: Uhm and have you ever Googled any other questions about prenatal care or health online?

ADL: Uhm… sure. I'm trying to think what that would be. Oh I had poison ivy in May…

INTERVIEWER: Oh no!

ADL: Yeah, it's terrible. So I like looked up a bunch of stuff on that. And then I just went to my doctor because I was like oh this is really bad. Uhm yeah I think I went to MayoClinic. I'll usually go to… or uhm… what's that other big one? Generic… uh. WebMD.

INTERVIEWER: Okay. Uhm, how do you typically gain access to the internet when you search for things like that?

ADL: Well uhm, I have the Internet at home and then also at work.

INTERVIEWER: And do you use a computer, a tablet, your cellphone?

ADL: Uhm, usually a computer, but sometimes my cellphone.

INTERVIEWER: Okay. And how often would you say that you've searched for prenatal information online throughout your pregnancy?

16:39

ADL: Uhm… well it's hard because I'll get "What to expect when you're expecting" e-mails… so it's not like me instigating the search. But you know I'll just browse through it and if there's something interesting that will come up… so I guess like three times a week?

INTERVIEWER: Okay. (mumbles) Okay, and your cellphone is a smart phone?

ADL: Yep.

INTERVIEWER: And you've had the same phone throughout all of pregnancy?

ADL: Yeah. Yup.

INTERVIEWER: And no dips on service or anything like that?

ADL: Nope.

INTERVIEWER: Okay. How often would you say you read the text messages that were sent from the study?

17:38
ADL: Uhm, I mean I usually always skim through the message. I don't always go to the link if there's a link. … How do you guys decide like what messages to do and when to do them? Because I kind of noticed a lot of them were similar to the pregnancy app that I had used. So like similar nutritional information. Which was good. (laughs). But I didn't know like if there's a certain standard you have to follow.

INTERVIEWER: Uhm, we weren't really following a standard. We were trying to vary the topics so that it didn't get boring, so we sort of had an idea that out of six messages a week, we would have one on supplements, one on nutrition, or something to do with food so that it can be a recipe also. Uhm, we have a miscellaneous day, which is (laughs) our kind of our category where we're not really sure what the message is—where they fall. A physical activity day… uhm, let's see… something maybe in the news, or something to think about more… so that might be like a research finding. And then we called it our Feel Fab category, which was, uhm, kind of like mental health… relaxing… uhm. Making sure that you take you know time for yourself. So, we're kind of all over the place. And a lot of the things we were recommending, we try to base off of evidence-based recommendations that already exist so that we're not… we're like using actually quality…

ADL: …Making stuff up, yeah.

INTERVIEWER: Exactly.

ADL: That's good.

INTERVIEWER: Yeah, that's our goal. You know we don't want to mislead anyone.

ADL: Do you ever sort of repeat messages but word them differently? You know how people need to hear things a couple times. I do at least.

19:52
INTERVIEWER: I would definitely say that a lot of our messages… are they're technically, if you compare the sentences, they're different, but they're about the same topics. And we're still pushing the same underlying message of, you know if it's like hydration, we're still talking about a different aspect but it's still hydration. So uhm yeah, some of them probably look like the same sorts of things. And since the main goal of the study was to work with pregnant adolescents, and we've recruited adult women as a comparison group, I think the teens especially—the messages were designed more for them to be repetitive and constant reminders because they get, you know, busy and easily distracted. I mean, I am, and I'm an adult.

ADL: And so am I! (laughs)

INTERVIEWER: And our other goal was to be motivating without being nagging. I mean, cause we all know we should eat healthy and exercise, but there's days where it's hard. Like you mentioned, you know, you're tired and you want to go home but you're like, oh! I need to exercise because of the baby and so it's those things we're trying to get people interested in and get over that hump. That they can actually do this stuff, so.

ADL: Cool.

INTERVIEWER: Yeah. Let's see. So you said you didn't always click on the links. Could you tell me a little about what would keep you from clicking on a link or what would inspire to click on a link?

ADL: Uhm… I mean so part of the time it's just when I'm looking at the text, you know like I'm not always like, sitting down, and not doing like… I'm usually doing… I'm usually like multitasking you know and, like I get something on my phone, and taking a quick look and you know, I'm running around. So it's sort of competition for attention span. And then… just if I skim through the message and if it's something that I understand and I feel that, you know, I got. Maybe I'm not going to click on the link to read more. Uhm so yeah, it's just like bandwidth and then how much I already know. Which doesn't mean I know it.

INTERVIEWER: No, that makes sense. Uhm, would you say any of the messages had a bigger effect on you than others?

ADL: Uhm, honestly I like the little like quizzes that you send cause I feel like it makes me have to stop and have to think about what my answer's going to be. Um, so, yeah, I think that the texts that are more interactive force me to think a little bit more or as the text where I'm reading it, I'm just like… okay. Get protein. Got it. You know like, it doesn't stick as much.

INTERVIEWER: Okay, that makes sense. Did you have any topics that were your favorite topics to read about?

23:11
ADL: Uhm… I think I like the ones about… that concept of eating for two and how you're really not. And like how many additional calories you need and how it's not really that many more calories that you need just cause I was trying to figure out… because I'm constantly like grazing all day, it's hard for me to figure out how many calories I'm getting. But I mean I'm gaining weight at the right level, so... I think it's good. But yeah, those were kind of interesting.

INTERVIEWER: And how about least favorite?

ADL: Uhm, I mean probably just ones that I already know. Like hydration… uhm… partly it's also—and obviously you have no influence over this, but—because it's been hot recently and twice a day people are like, "Are you drinking enough water?" And I'm like, "I don't even know you. Like I'm fine. I'm drinking enough water." So I think if I ever get a text from you guys about hydration, I'm like, "I GOT IT!" (laughs) I know people mean well, but it's just funny. I'm like yes. I'm drinking enough water!

INTERVIEWER: Oh no. Everybody wants to be helpful (laughs)

ADL: I know… and it's very well meaning and very sweet but I'm just like, god, I am going to be a not-a-nice person in my eighth month (mumbles)

INTERVIEWER: (laughs) Did any of the messages inspire you to change behaviors or try something new?

25:17
ADL: Uhm… well the calories thing was good, because I did try to track my calories and totally failed. But I tried… mostly because it was just hard for me to figure out portions. Like I'll bring a Ziploc of almonds to work and eat them throughout the day and like what I don't finish just stays at my desk. So I have no clue. Did I have half a cup? Did I have a cup? You know so, yeah, it kind of got me more motivated to think more about portions. And you know, like I think I'm getting a cup of food, but I'm really getting half a cup. Or you know, figuring that out a little bit more.

INTERVIEWER: Well yeah, those are hard things to visualize when you're not actually you know sitting there with measuring cups, so.

ADL: Yeah, and then I'll start eating and get sick of it and put it down, so… I don't know…I'm taking my pre-natal.

INTERVIEWER: That's always good. Good assurance. I mean it sounds like you're eating really healthily even if you don't eat the whole thing that you packed in the bag. I think it's better to that you force yourself to eat it just because you know it's good, but you're not

feeling it. That sometimes—that just makes you miserable. Uhm… so anything else that you can think of that… a message where you clicked on a link and watched a video or um tried a recipe or anything like that?

ADL: Uhm, I uh I think I clicked on recipe links, but I'm just like not making any recipes these days. So I'll look at it and be like, "Oh that looks good." And then I don't make it (laughs). I don't make any recipes, so…

INTERVIEWER: No, that makes sense.

27:24
ADL: My husband's made me this smoothie last night and it's four things in a blender and… I couldn't do it.

INTERVIEWER: (laughs). So, some of the messages had to do with weight gain. And I'm just curious how you felt about those messages and if they had any influence on the weight that you gained during pregnancy.

ADL: Uhm… I mean it's funny cause I, I think I was always mindful that I needed to eat healthily and (excuse me) exercise… but I've been… my midwife just laughs at me because I'll be nervous that I'm not gaining enough weight. And she's just like, you know, as long as you're still eating enough and exercising and you're still not gaining that much weight and your baby's a normal size…like that's a good problem to have (laughs) So… but I think it's going to catch up. I don't know. I'm going to start my third trimester in a couple weeks and I think that's where all hell breaks loose in terms of weight (laughs). Like I think the baby gains half a pound a week or something.

INTERVIEWER: Mhm.

ADL: So wait, that's like six pounds?

INTERVIEWER: In the third trimester?

ADL: I think so.

INTERVIEWER: Uhm, so it sounds like you've been a little nervous about weight gain, but would you say that you're happy with where your weight gain's been and feel that's a healthy amount for your pregnancy?

ADL: Yeah, yeah. I mean I feel really good. Uhm… I don't really feel like I've exploded all over. Like most of the weight is in my stomach or my chest. Uhm… so varicose veins run in my family, so that was another reason that I didn't want to you know go above the 25 or 35 pound range. Uhm… just because uhm… they seem kind of painful so. Actually when I went to my midwife appointment this week, she said she saw a couple that were starting and I was like, "Oh, god." So… I might have to buy support hose. Which I don't know about if they're going to be gross in the summer, but…

INTERVIEWER: Those run in my family too, so I feel your pain.

30:08
ADL: Yeah. Yeah…

INTERVIEWER: uhm, let's see. Uhm. Did any of the messages influence how you felt about foods that you were eating?

ADL: Uhm… I think that definitely that, it's funny. Probably this thing that a group of food that I'm not getting as much as I should is vegetables which uhm… they don't really. I don't know why I'm like this but they take a little bit more effort. Uhm… to like clean and prepare. So like, just like texts about variety or getting your veggies are good reminders. Like yesterday I realized, like when I was having the kale smoothie, that that was the first vegetables I'd had all day. Just cause I'm like, oh fruit's so easy. Yogurt's easy. Cheese is easy. So I just need to be a little bit more mindful. I mean vegetables aren't that difficult. I just need to do it.

INTERVIEWER: Uhm. Were there any foods that you avoided eating because of the messages?

31:39
ADL: Uhm. I think I was just a bit more moderate. Like it's funny. Everyone wants to like feed you ice cream or like cookies. And I've been lucky. I haven't really been craving a lot of that stuff. But with the weather getting hotter, I just want to sort of eat colder things so, like ice cream is good for that. But, um yeah. I think I've been just trying to be moderate and not go crazy.

INTERVIEWER: Mhm. And I know you said that food prep's been pretty minimal… uhm, but your husband has maybe prepared some meals so, during pregnancy have you or your husband changed how foods are prepared, so for example… if you used to fry chicken, now maybe it's grilled or broiled. That sort of thing.

ADL: Uhm, not that I could think of. I mean I don't think we fried a lot of food before I was pregnant.

INTERVIEWER: Mhm.

33:01
ADL: Or just as an example.

INTERVIEWER: For your prenatal vitamins, have the text messages had any effect on the vitamin that you take or how often you take your vitamins?

ADL: Uhm… I don't think so. I mean I like… I don't do the gummies cause they don't have as much or as much of a range of vitamins. Uhm, but pretty much from the beginning yeah I've been taking a prenatal daily… a calcium supplement and cod liver oil.

INTERVIEWER: And you don't have any trouble remembering to take them everyday?

ADL: Uhm… there's times when I'm like trying to get to work. That I don't, you know I'll like pack them in my bag and I'll be like, I'll take them and of course I forget. But… I mean once I remember that I've forgotten, then I'll take them. So.

INTERVIEWER: Okay.

ADL: It hasn't been like days where I'd forget.

INTERVIEWER: That's good. And uhm how do, would you say have the text messages affected your activity level?
34:29
ADL: Uhm… yeah I think they're just good as extra motivators when I'm tired, I want to go home, to make myself still go to the gym. But like I definitely… I don't push myself in the gym. Like I don't want to pull a muscle. I don't want to overheat. So... yeah. I think I've gotten better at making sure I exercise consistently but not killing myself.

INTERVIEWER: Mhm. Did any of the messages motivate you to try a new activity that you haven't done before?

ADL: Not that I could think of.

INTERVIEWER: Okay. And have you heard of any benefits of physical activity on uhm mothers or their fetuses?

ADL: uhm, well yeah just been thinking about the varicose vein thing, you know. Keeping moving. Keeping my circulation moving… uhm. I heard that babies like the boost that you get from exercise too cause it ups their circulation and their systems I guess. Uhm… and then just like some of my friends have really talked about how they just felt like they were in better shape going into their labor and that helped either make their labor a little faster or their recovery a little easier and then getting back into a pre-baby body… if that's possible at all. Uhm, you know that the weight came off and things came back a little easier, they thought.

INTERVIEWER: Mhm. Have you ever shared any of the information that you received from this study with a friend or family member?

36:59
ADL: Uhm… yeah, I think that especially when people are like, "Oh! Here's cake!" And I don't really want to eat cake… if they think I'm dieting or… I'm not dieting. I'm just trying to eat normal. You know like I think I'll talk about like what you need and don't need and

uhm… they you know usually back off a bit. It's so funny that people, you know, they try to like cram food down your mouth. We're so weird with like the way that we eat, it's like we don't ever do anything in moderation. You know it's either like an extreme diet or eat everything.

INTERVIEWER: Yeah, we're very much a society of excess, I think (laughs)

ADL: Yeah. On like both ends. It's a lot.

INTERVIEWER: Have you joined any other e-mail, text message, Twitter, blog, or Facebook groups about pregnancy that you didn't mention yet?

ADL: Uhm… I have not. My friend like signed me up for like, "New first time pregnant mom" Facebook thing where we like… we're due a week apart. So she's like, "Oh! I signed you up. If you don't want to do it, that's fine." I lasted like a week. I was just like… like I was just getting like people would post so much. And I would see on my phone like, oh! I have eight things on Facebook. And I would look and it's just always people like, you know, I'm trying to dye my hair. Can I do it? And I'm just like, "I can't. Too much information." So no, you're the only one. And the pregnancy app that I signed up for.

INTERVIEWER: Okay. And so this Facebook first time moms group is mostly just a forum for women to share what's going on and ask one another questions?

ADL: Yeah and I just I couldn't take it. (laughs)

INTERVIEWER: Um. So based on your experiences after having received information from apps, text messages, your midwife, family, friends, is there one place where you found provided you with the best information on pregnancy?

ADL: Uhm, I think I really like talking to my midwife, just cause there's so many things that you'll read about or like you'd be told. And I could be like, "Beth, is this true?" And half the time she'd be like, "No." (laughs) Good… so yeah she's like my last my arbiter of truth. My friend's getting married and her bridal shower's tomorrow night and so I got the e-vite and it's like, we're going to take pole dancing lessons. (laughs) And so… I was like laughing, e-mailing my midwife. I was like here's a fun Monday morning question. Can I go to pole dancing lessons? The answer's yes. Just don't do anything stupid. Yeah, like don't do a lot of core… strenuous core stuff. And be careful of your balance. But she was just like, "What are you doing?"

INTERVIEWER: (laughs) that's awesome.

ADL: Yeah, I don't know how much… I'll probably just. I don't know how much I'll be able to do. But… I'm going (laughs)

INTERVIEWER: Yeah, at least you'll be moving around. Getting some activity.

ADL: Yeah. I'm not going to do any of the lifts.

INTERVIEWER: So you said the Facbeook group just had too many posts. So this next question will be a good follow-up to that. So in terms of the study where you're getting text messages, what do you think a good number of posts would be um per week to send out? And you were getting anywhere between like two or three, up to six a week. So what are your thoughts on that?

41:39
ADL: Uhm. I don't remember ever thinking it was too much. You know like I think it was good. It's hard because you don't want to bombard people to a point where they'd check out but you know, if like you only send a text once a week, people might not be as engaged. Um. But yeah, I don't… I don't even remember like being like, "Oh! I only got two this week. Or wow, we had a lot this week." You know, like it just always seemed like a good—good amount.

INTERVIEWER: Okay, that's helpful. Thank you. And I mean our goal was always to always give constant reminders to motivate positive health behaviors.

ADL: Right.

INTERVIEWER: So it sounds like you got a really good relationship with your midwife. So one thing that I'd like to ask is something like this texting program. Would it be a good thing to have the midwife staff or somebody at that office do to… you know where they're sending it? And I know some people are worried that maybe the information isn't uhm… as valid um or that the links aren't safe. I have like been told that. So I don't know if you've ever felt like anything that we sent… you didn't know was coming from a safe source.

43:17
ADL: A safe source… You know, I think it's been fine. Yeah no, I think it'd be good for midwives and OBs like for their practices to use it. Uhm… yeah. I'm trying to think. Like I remember in my OB's office, I think she had like text for babies cards… so… I mean it's probably something that you know. That'd be good. But they don't want to take it on.

INTERVIEWER: Have you heard anything about the text for baby program at all?

ADL: Uhm, I haven't looked into it. I know it's… I think the CDC runs it, right?

INTERVIEWER: Yeah, them with a number of other industry partners I think.

44:10
ADL: Yeah, yeah. No I haven't I haven't used it but, I've heard of it.

INTERVIEWER: Okay. And you don't know anyone who's ever used it?

ADL: No… or.

INTERVIEWER: I guess just out of curiosity, what uhm you know, I mean you were interested in being in the study. What… why were you not interested in checking out the text for baby program?

ADL: Uhmm… well that's not a study right? I mean that's—

INTERVIEWER: No, it's just a thing that you can sign up for for free.

ADL: Uhm, I think I just like research studies. I just think they're interesting. Uhm… and I never qualify for them and now I'm pregnant. So yayyy I qualify! (laughs)

INTERVIEWER: I know exactly what you mean.

ADL: (overlap) Text for baby seemed a little bit more impersonal, a big national program. I mean like this is a more local study; it's a little bit more interactive. So.

INTERVIEWER: Mhm. Okay. That's helpful. Uhm… and since I did mention that the study is—you know our goal really is to try to help motivate healthier behaviors in teens…

ADL: Mhm

INTERVIEWER: What advice would you have, based on your knowledge of social media, is there a better way to reach adolescents? Uhm, is you know Facebook better? Text message? Is there another app?

ADL: It's so hard because it changes so quickly. Uhm… you know like more and more I think Facebook is more used, actually by adults, and like teens are on like Instagram or Tinder or something like that. But again, in like two years, the platform that they use is going to be different. You know it keeps… like SnapChat or… you know it keeps changing. I mean text seems to be pretty consistent. You know like that platform hasn't really changed. Uhm… and then you can get the teen who don't have a smartphone… who has a phone phone?... uhm do you find that all teens have a cellphone? Do they have to have a cellphone to be in the study?

INTERVIEWER: Uhm, so they actually didn't. We offered a paper mailing option as well because we didn't want to—from an like ethical perspective, we wanted to offer them all the chance to have access to health information. Uhm I think and we pulled about 40 of the teens who went through the clinic and 93% of them had a cellphone. And the greater majority were actually smartphones, too. Which is..

ADL: Oh wow. That's crazy.

INTERVIEWER: Yeah, it is. So uhm we didn't have any of them who enrolled that ended up choosing paper. So, most of the ones who enrolled chose cellphone and/or Facebook or a combination of both.

ADL: Right.

INTERVIEWER: So, it's interesting. So they even though they're maybe at a disadvantage to have the same amenities as other people, they all seem to have cellphones, so.

47:52
ADL: Yeah. Huh. So did they open… can you track if they opened… can you track opening texts? Or I guess no. Is it like email in that sense?

INTERVIEWER: There are some programs. And I looked into it. But given the cellphone service plan that I was allowed to use through the business office for research purposes, the phone unfortunately was not eligible with that. So that was kind of a bummer (laughs)

ADL: Yeah, yeah.

INTERVIEWER: So we don't have a way to measure text reading, at least. But Facebook tracks that, so I can track those things. But yeah, I really wish I would've pushed harder for a different cellphone package so I could of used it… I think it's called Tiger Text.

ADL: Oh okay, okay. Well it's hard though, too. Cause sometimes I can see my text like across the top of the screen on my phone without actually opening it. So it seems it wouldn't be totally accurate anyway.

INTERVIEWER: Yeah I think when you actually use that program, it encrypts them and closes them so you have to go in, and then it would say "would you like to read this message?" and you click on a link. And I almost wondered, too, if that's a definite con because it requires extra work. And to me, that feels less personal, like it wasn't actually meant for you. Like you have to, you know, click in. I don't know. So.

49:22
ADL: Mm. okay.

INTERVIEWER: So yeah. There's pros and cons to it, but…

ADL: hahaha. True.

INTERVIEWER: Uhm, so I think that's the majority of everything I had to ask you, but is there anything that we talked about previously that you thought another answer or something that you kind of wanted to talk about that I didn't ask about?

ADL: Uhm… not that I can think of.

INTERVIEWER: Okay. Uhm well then I guess that's it for the interview. Uhm, do you have any questions about anything else in relation to the study?

ADL: uhm… is there a… I can't remember. Is there a postpartum part or a third trimester part… or is this it?

INTERVIEWER: Uhm so this is it. And you'll just have one more questionnaire. It's the—you had already filled it out in the beginning of the study.

ADL: Oh yeah.

INTERVIEWER: It's the nutrition knowledge questionnaire. And then one more diet record. And then so I'll have [name redacted] coordinate with you to do that sometime.

ADL: Okay.

INTERVIEWER: And then after that she'll give you—you should get $30 for this. And then another $10 gift card for each of those two things. So you should get $50 total. So, just so that it's on your radar.

ADL: yayyyy (laughs)

INTERVIEWER: (laughs) Yep, baby shopping spree at Wal-Mart. WHoo.

ADL: Whooo (laughs)

INTERVIEWER: Uhm so yeah. So will get in touch with her today and have her reach out to you, and uhm so that you can finish up.

ADL: Okay, cool.

INTERVIEWER: So I guess this will be the last time that I will talk to you, so best of luck with the delivery and I hope that everything goes well and that you enjoy your time with your little baby in a couple of weeks.

ADL: Thank you.

(closing remarks)

INTERVIEWER: Um, so it's been a while since we last talked, um, one on one and I wanted just catch up a little bit about how things have been going, um so can you tell me a little bit about your pregnancy since we…first met earlier?
ADL12: Um, overall it's been going well, um… I…had nausea until about 17 weeks, but since then I've been good. Um just recently in these last few weeks I've been just feeling more tired, so um I just talked with my midwife we're gonna start um an iron supplement just a couple times a week not like daily, my numbers are still like normal but they're lower than they were at the beginning of my pregnancy so, um she's thinking that might help…but other than that that's really only my only complaint I guess I would say, or issue that I've had, so um.
INTERVIEWER: What would you say has been the best part of this pregnancy?
ADL12: Umm…I think, I mean I have a daughter she's 15 months, so…she thinks her belly button is a baby (Interviewer laughs) and she points to my belly and kisses it, so I think just experiencing it with a…little one has been…
INTERVIEWER: That's really cute
ADL12: …fun. Yeah it's adorable.
(Interviewer and ADL12 laugh)
INTERVIEWER: Um and then last time we talked, I had asked what your hopes and dreams were for, um your baby to be and um can you tell me a little bit more about what those are and if anything has maybe changed as things have progressed?
ADL12: Umm, well you know just hoping as far as labor goes, umm for an unmedicated, uneventful, um…labor and delivery, and then just a healthy baby, no complications, um…hopefully we can get out of the hospital as soon as allowed and, that he's just healthy and that he breastfeeds well.
(Interviewer and ADL12 laugh)
INTERVIEWER: All good goals. Um, have you changed any of the ways that you prepare food? Um since early in pregnancy?
ADL12: Umm, just trying to… be…healthy, overall, um…so as the pounds keep adding on, um, you know, just trying not to eat out as much, umm…we did order pizza last night but we used to, every (laughs) weekend have pizza and we haven't done that. Um, but we did have it last night, so…um just kind of as a whole my husband and I are both trying to…eat healthy and um not have as much prepared food, stuff like that.
INTERVIEWER: Mhm. That sounds good. And then is that different from earlier in pregnancy?
ALD12: Ummmmmmm
INTERVIEWER: Or just different from before pregnancy?
ADL12: Umm…I would say probably in the last couple of months, we have been, um eating a little healthier, my husband's also kinda on a big health kick, cuz his blood pressure was…high when he went to the doctor last, which…I mean he's very young and fit so we were kind of surprised, but he has a lot of heart issues in his family, so..
INTERVIEWER: Mhm.
ADL12: since then, um both of us has been trying to kinda (laughs) eat healthier so.
INTERVIEWER: Well sounds like you guys are making good progress with that.
ADL12: Yeah.
INTERVIEWER: Um, so…in terms of when you prepare food at home from scratch, have you changed anything about how you do that?

ADL12: Um…I'm not sure I've changed anything since pregnancy…um…I try not to use, you know, lots of oils and all that stuff. Um…but I would say preparation ways would probably been about the same.
INTERVIEWER: Um how's your dairy consumption been?
ADL12: Um…I typically, I've drank more glasses of milk. I would say in the last month I'm typically not someone who…drinks milk. I have it on my cereal, and that's it. Um…but I have noticed that, every once in a while I just…want to have a glass of milk! (Interviewer laughs). That's not my norm but um…other than that, it's probably been about the same.
INTERVIEWER: And is that just milk or has…have you also felt like that about cheese and, um, yogurt as well?
ADL12: Umm I like to eat yogurt so I usually have yogurt, I would say at least 3 or 4 times a week. Um and I always have if I have a sandwich I always have cheese. Um so I like cheese and yogurt. And it being summer I also like ice cream. (laughs) So.
INTERVIEWER: It's a good time of year for that. Have you…I know you said that you didn't have any cravings yesterday, um, but have you had any cravings throughout pregnancy for non-food items? Like baby powder, dor- dirt, chewing ice…
ADL12: Nope.
INTERVIEWER: Okay. Um, and are there any smells that you like more or less?
ADL12: Um, I don't think so.
INTERVIEWER: Okay. Um, and any challenges that you've faced in terms of eating or exercising during pregnancy?
ADL12: Umm…When I was pregnant with my daughter I was at the gym 3 times a week, you know I hadn't had other children, so.
INTERVIEWER: Mhm.
ADL12: Um, I'm a stay at home mom 3 days a week and then I work, um, Thursdays and Fridays, so um I have just recently I did a like prenatal exercise which you got a free gym membership with,
INTERVIEWER: Mhm.
ADL12: so um I have been able to do that cuz I can take her there to the um childcare, but it's definitely more challenging to…work out, um, you know besides just being able to walk and do things at home, um…now that I have her, cuz on days that she's at daycare I'm at work. So, um, that's…been more difficult this pregnancy, just to get good workouts in. Cuz to me, walking is…it's exercise but it's not a workout. (laughs)
INTERVIEWER: Mhm.
ADL12: So.
INTERVIEWER: What motivated you to join the, or to do the prenatal exercise class?
ADL12: Umm…it just sounded kind of fun, um, we just had moved to [location redacted] and it was through like the [name redacted], so I figured, you know, I might meet some moms who are also due around the same time as me, and it's like a yoga and then there's um a pool portion after that.
INTERVIEWER: Mhm.
ADL12: Um, and you've also got the free gym membership for the duration of the class, so, um…I've always been someone who's joined in a- who's been a gym member and who has worked out. So not having done that for the past…year or so…it was kind of a cheaper way to um get back to the gym at least a little bit. So…
INTERVIEWER: Yeah.

ADL12: …it's been very nice.
INTERVIEWER: That does sound nice. Was that a promotion that they were doing to try to make-
ADL12: Um they just do like [name redacted] um, it's just like a community program and they like join up with different gyms and do like, they offer like senior classes and…so it just kind of was one of their things that they do, um, in the summer. So.
INTERVIEWER: Nice.
ADL12: Yeah!
INTERVIEWER: Um…let's see. This is kind of a loaded question, but..,
(INTERVIEWER and ADL12 laugh)
INTERVIEWER: Just whatever you know you feel that you have knowingly done to have a healthy baby. But what things have you done throughout pregnancy to make sure that your baby is born healthy?
ADL12: Umm…I think just trying to be active, um…trying to eat healthy, making sure I'm taking my vitamins and…doing all my prenatal care…
INTERVIEWER: Um and ha- prenatal vitamins, do you have trouble swallowing those at all?
ADL12: Mm mm. (vocalization for no)
INTERVIEWER: Okay.
ADL12: No, I did take the gummies in the beginning, just cuz some people said that it's ha- better when you're nauseous, like they're just easier to absorb, so um. But now I'm, I'm just like the regular pills and they're fine.
INTERVIEWER: Um, are there any places you have gone to for advice about health during pregnancy? Can be people or Internet sites, books…
ADL12: Um, not as much this pregnancy, just cuz I just did it a year and a half ago (Interviewer and ADL12 laugh). Um, but…I mean I definitely didn't like read and look up stuff as much as I…did the first time.
INTERVIEWER: Mhm.
ADL12: Um…I'm not sure I really…sought out much information. (laughs).This time
INTERVIEWER: That's okay. You already have the experience so, now you're the expert. Um, did you ever use the Internet to look up anything during this pregnancy?
ADL12: Yeah. Um…I think the one thing I remember is just looking up the fish um like what fish is allowed, how much. We did have crab. I can't remember when that was but I remember googling it quick, don't remember if that was okay or not. (Interviewer laughs)
INTERVIEWER: Anything else that you can remember looking up?
ADL12: Um…I've just I have been reading and looking up about um just like natural childbirth, cuz I did remember reading a book before I had my daughter but it did not go well. And it was not, (laughs) it did not end how I planned. So, um, I'm just reading more different things this time and trying to uh, um prepare and hoping that you and the midwives will help that as well. Um but that is one thing that I have been reading about as much as like the actual labor, birth part of it. (laughs)
INTERVIEWER: Mhm. Um and where do you typically gain access to the Internet when you search for things like that?
ADL12: Umm, usually my phone. If I'm home I will use our home computer, but typically my phone.
INTERVIEWER: Okay... Um and are there any sites that when you search for things that you found are most helpful? For providing quality pregnancy information?

ADL12: Umm…I feel like for the few things that I've looked up, um...I've just used babycenter.
INTERVIEWER: And that includes the natural childbirth as well?
ADL12: Umm that I just, um, kind of looked up different books.
INTERVIEWER: Mhm.
ADL12: Um, and, kind of asked you know some of my friends and people I know that have um read different things, so that, I'm just reading actual books, not um stuff on the Internet about it.
INTERVIEWER: Um and are there any other sites that you found to be helpful other than babycenter?
ADL12: Umm…I guess not that I've really used…
INTERVIEWER: Okay. That's fine. Um let's see. And…so you were in the Facebook group and text message so you've gotten both messages.
ADL12: Yes. Mhm.
INTERVIEWER: So, um, first I want to talk about Facebook.
ADL12: Mkay.
INTERVIEWER: And, just to start, how often would you say that you read the messages or checked the site?
ADL12: Um I would say for the most part every day.
INTERVIEWER: Mhm.
ADL12: Um…there were a couple of times um like days that I work I'm usually not on my phone that much. But I would say for the most part, I saw it every day.
INTERVIEWER: And did you normally check them in your newsfeed or did you actually go on to the group page?
ADL12: Um I usually would see it in my newsfeed and click to the page.
INTERVIEWER: Okay… Um so overall how helpful would you say that the group was? And that can be in comparison to text message since you did um both of them if that helps.
ADL12: Um. Do you want me to like, rate it? (laughs)
INTERVIEWER: Um sure if that helps so we could do like on a scale of 1 to 10, if 1 was not helpful at all
ADL12: Okay.
INTERVIEWER: and 10 was very helpful. Um so pick on a scale
ADL12: Um I would say…like a 6 or 7?
INTERVIEWER: Okay. Um and why would you say 6 to 7?
ADL12: Um…I really liked the um like exercise tips especially if there were like videos. Um there was one video on there. It was a while ago, but it was like a second trimester workout that I did many many times (both laugh). Um so that was nice. Um and then I liked the like nutrition tips but also like the actual like eat this try this. I like different recipes and things like that. Um…yeah.
INTERVIEWER: What would've made you give it a 10? So what was missing that would help you rate it higher?
ADL12: Um…For me I think some of the posts were just a little too generic to be helpful.
INTERVIEWER: Mhm.
ADL12: Um, and…I think the more specific they were the more helpful, so some you know just the more broad, um, topics or posts um just kind of weren't as helpful.

INTERVIEWER: Mhm. Can you…think of any specific examples of things that would've been more helpful if they were more specific?
ADL12: Um, well like I think with the exercise thing there were only what I can remember like 3 actual like links to like videos or whatever. Um.
INTERVIEWER: Mhm.
ADL12: And then…
INTERVIEWER: So it could've had mo- more links?
ADL12: I think so, yeah. And then I was looking for like, cuz I think that one video was like a second trimester, I think they did um last week maybe but there was a third trimester one. Um…
INTERVIEWER: Mhm.
ADL12: But just for I mean I kind of look them up on Youtube on my own, but um just for like the variety of things to do…
INTERVIEWER: Did you ever have um any doubts that any of the links were unsafe to click on?
ADL12: No I just kind of assumed they were okay. (laughs)
INTERVIEWER: I like that answer (laughs). Um let's see. What was your favorite and least favorite part of the facebook group?
ADL12: Um, I would say my favorite part were the um the exercise videos. Um, my least favorite part? Um…I don't know I guess I would just say not all the osts were helpful
INTERVIEWER: Okay.
ADL12: But I mean I think that's kind of to be expected. You can't please everyone with every post. (laughs)
INTERVIEWER: Um…what motivated you to continue to check the messages and go to the group page?
ADL12: Um I just like um you know, like, learning new things, getting new tips, um…you know it's kinda like why I buy exercise magazines, even though you know a lot of it says the same stuff. But, you know you might learn a new exercise, or be bough onto a new food or new way to cook something. I think at one point there was a post about roasting broccoli or roasting something. And I was like, oh I've never done that. Um and it was really good! So um I don't know I just like getting tidbits about things.
INTERVIEWER: Um and so you said that the exercise videos were your favorite part. Um so this is kind of a similar question so it may be the same answer. But um, of all the different types of information that were provided do you feel that some topics were more relevant to you?
ADL12: Yeah. Um, you know like there were um…some posts about like you know listening to calm music, and um, you know like avoiding depression and things like that. Which weren't as relevant to me but you know that doesn't mean they're not helpful to someone else, so.
INTERVIEWER: Mhm. Um and so it does sound like you tried some of the ideas and watched some of the videos.
ADL12: Mhm.
INTERVIEWER: Are there any other um behaviors in relation to exercise of eating that you changed because of something that was posted to the Facebook site?

ADL12: Um…I'm not sure there's anything that like I changed doing overall, but um…I mean I did you know try the videos and try different recipes and things like that…if that answers the question.
INTERVIEWER: Yeah! Um how often would you say that you clicked on links that were provided with a post?
ADL12: Um…I would say probably…seventy-five percent of the time?
INTERVIEWER: Um and did you ever disagree with any of the information that you received?
ADL12: Um…I don't think so.
INTERVIEWER: Okay. And in terms of when information maybe wasn't the best for you, or if it was too general, um is- are there any sites that you could suggest that would have better information that could be used as examples to improve the messages? If not that's okay.
ADL12: Mm…I don't know (both laugh). Probably not.
INTERVIEWER: Okay. Um, and I know that for many women weight gain can be a touchy topic and it it's hard to gain weight even when you're not pregnant. So, how did the messages play a role in how you felt in gaining weight throughout pregnancy?
ADL12: Um…I mean for me, it's…I don't know, it's not something I stress about.
INTERVIEWER: Mhm.
ADL12: So, I don't feel like I, s-…like really seeking…I don't know, encouragement or support about like that particular thing.
INTERVIEWER: So those weren't necessarily the messages that really stuck out to you.
ADL12: Right.
INTERVIEWER: Um overall, how do you feel about the amount of weight that you gained. Do you feel it's right for your pregnancy and your body?
ADL12: Um…yeah. I…gained two more pounds than what my home scale said at this visit. So I wasn't mentally prepared for that. But, um since I just was pregnant with my daughter I kind of expected to um gain about the same. Which I have. I'm bigger, like my stomach is bigger this time but my weight gain seems to be about the same
INTERVIEWER: Mhm.
ADL12: So, I'm not at all surprised. Um and I didn't weight gain that much in the beginning. And then kind of once I started feeling better is when I started kicking more which is what happened with her too. So um I think if it had been drastically different, then I may have had more of an issue.
INTERVIEWER: Makes sense. Um, and so you mentioned that you tried roasting vegetables because of the post. Are there any other things about how you were eating that you changed or something else you tried that you can remember specifically?
ADL12: Um…I did start buying more like um almonds, I made like trail mix, um, just to kind of I think there were a few posts about varying um, like protein sources and things like that. Um…and my husband really likes trail mix, so we kinda got into doing that a little bit more.
INTERVIEWER: Mhm.
ADL12: Um and just kind of having almonds as a snack, you know as a quick thing. Um, you know as an alternative to maybe a granola bar or a less, a less processed alternative.
INTERVIEWER: Um…has your role changed in preparing and shopping for food at all?
ADL12: Um…well I'm the one that shops and prepares and I have always been. (Both laugh) So no change.

INTERVIEWER: So no change! Um and…did the Facebook site affect how you take your prenatal vitamins at all?
ADL12: Um…no.
INTERVIEWER: Okay. It sounds like you've been doing a really good job with those, so it'd be hard to make it any better. (ADL12 laughs). Um and so physical activity levels. So you tried some of the videos. Um how often have you been exercising?
ADL12: Um I try to do something every day, even if it's just a walk.
INTERVIEWER: Mhm.
ADL12: Just some like conscious decision you know. Um so like if I do like one of those videos during my daughter's nap, or something. Um, so I would say at least you know six days a week I'm trying to do something.
INTERVIEWER: Mhm.
ADL12: Just to give myself a little leeway but um it's not always something extensive. Which is sometimes a struggle when I feel like some people aren't, like, to them a walk can be a workout. But if you're someone who has always been like a sweating, you know, like a workout needs to be…you need a real workout. Um I'm definitely that person. So, I'd say activity every day, it may not actually be a workout but um yeah. I try to do something daily.
INTERVIEWER: Um, and…did the Facebook messages at all change how you felt about fitting in some sort of activity each day? Or influence that?
ADL12: Um, I think any of the posts as far as like exercise, being active, you know, it just kind of helps to reiterate or motivate, um, you know to continue and not…get lazy. I don't think. I wouldn't say it changed, but I think anything helps to encourage it.
INTERVIEWER: It sounds like you're very motivated. And I know for some women when they get really tired they struggle to stay motivated to do any exercise, so I'm sure that you've had days where maybe it was like that. So what's the difference between the good days and the bad days, and like if you have a bad day, and you are able to get yourself up, what is it that makes you get up and go for that walk?
ADL12: Um…well like I said I had talked to my midwife cuz I just have like, like every day I take a nap when my daughter takes a nap in the afternoon which is like so not me. Um, and so I have been like a lot more tired and it has been hard, but like yesterday, like I worked Thursday, Friday, Saturday. Um, and I was like oh, Saturday when I get home from work I'll go to the gym, and I…did not. I mainly still did the stuff um like outside and whatever, but so Sunday when I put my daughter down, I like really wanted to lay down. I was like you know, I'm gonna find a video, and I'm just gonna at least do like twenty minutes of something. Cuz I just for me it's a like a cycle. If I like do not work out and eat well, then I just like it gets harder and harder and I just like feel worse and worse overall. So I know that if I do something, even if I'm tired, I'll at least feel better about it (laughs).
INTERVIEWER: Mhm.
ADL12: So I did, I just did like a video um, I don't even think it was 20 minutes, I think it was just like 15 minutes. And then I did go take a nap (laughs). But I at least felt like I got something in so.
INTERVIEWER: Mhm.
ADL12: I think it's just I mean I know that myself if I don't um, like, make myself, if I go like a few days saying I'm tired, I'm pregnant, I'm not doing anything today, and I don't care.

You know if I do that consecutively, then it just gets harder and harder. Where if I find that if I do it daily, it's easier just to do it.
INTERVIEWER: Mhm.
ADL12: So.
INTERVIEWER: What, what do you think would motivate a woman who is the opposite, where, you usually have good intentions but it just never really happens. And- or maybe she was never fit to know what it feels like to have that opposite where like ugh, I- I feel so good when I work out, why am I not doing it.
ADL12: Yeah.
INTERVIEWER: I mean do you have any friends who…have a those sorts of experiences?
ADL12: Yeah, um, I mean I think that the biggest thing that I can say is just, even if you only like work out consecutively like three times a week for like one week, I think you notice that that is a better week.
INTERVIEWER: Mhm.
ADL12: You know, like, I know for me even when I got the gym now when I'm like you know hugely pregnant, and like I still like try to wear something that I feel good in. And that I you know like, wanna work out in. and then afterwards I just feel more energized, I just feel better about myself, and the more you do it, like even if you're physically not changing, you just feel like, you feel stronger, you feel better.
INTERVIEWER: Mhm.
ADL12: Um, its just one of those things that you have to I feel like, do, before you can, like, feel oh yeah I do feel better after that.
INTERVIEWER: That makes sense.
ADL12: So I think that's the one thing I could say is that you just have to do it (laughs). Then you'll realize. But you have to constantly do it, it's not like you know, you work out once and the whole rest of your life you're like, oh, yeah, this is no problem (laughs). Have to keep reminding yourself.
INTERVIEWER: Um so switching gears to cell phone stuff. Um did your access to a cell phone change at all during pregnancy?
ADL12: Nope.
INTERVIEWER: Okay and so you own your own phone?
ADL12: Mhm.
INTERVIEWER: You don't share it?
ADL12: Nope.
INTERVIEWER: Okay. Um, lets see. Um how often do you think that you read the text messages that were sent to you?
ADL12: Every day.
INTERVIEWER: Okay.
ADL12: Those I could not miss.
INTERVIEWER: And, um, so let's do the same scale thing where 1 is not helpful at all and 10 is very helpful. How would you rate the messages that you received through text?
ADL12: Um, I probably rate them about the same.
INTERVIEWER: Okay. And can you tell me about why you would give them that score? And if it's the same you can say the same.
ADl12: Yeah I would say it's probably about the same. I can't um, say that I noticed a huge difference as far as content, um.

INTERVIEWER: And I know with text message you're limited to 160 characters, which really makes it hard to be um specific and that's one of the things that you mentioned for the Facebook, where you can be longer.
ADL12: Mhm.
INTERVIEWER: Um so, how would you feel about in order to be more specific it would have to be more than one message at a time? Is that something that it would be too much? Coming in at a time?
ADL12: No.
INTERVIEWER: Or would that be okay?
ADL12: Yeah that would be fine.
INTERVIEWER: Okay.
ADL12: (indistinctive)
INTERVIEWER: Um and, so it's similar to Facbook, where there any messages on the phone that had the biggest effect on your pregnancy or that really stand out to you, something that you remember even now?
ADL12: Um I just think because the phone messages were smaller, um, like just a little nutritional like tidbits. I guess I would call them, were helpful. You know, just like little reminders about, you know, filling up your plate with this, um, try to eat more of this, um, so and especially like you know when you get a text, you're not necessarily like sitting down and consciously checking it. Like you are on like Facebook.
INTERVIEWER: Mhm.
ADL12: So you know, like just a short thing, just to kind of be like, Oh that's a good tip. You know but you don't necessarily have to like, click on them. Think about you know if I get a message when I'm at the grocery store and it has a link, I probably will forget to go back and click on it (INTERVIEWER: Right) if it's on my phone so, um, just I don't know. Like I said before, I just find like the little tidbits of information…
INTERVIEWER: Mhm.
ADL12: ...helpful!
INTERVIEWER: Um and do you recall any of the text messages being something that you did not like? Or t-topics that made you uncomfortable or anything like that?
ADL12: Um I don't think so.
INTERVIEWER: Okay… And um again did any of the messages inspire you to change any of your diet or activity behaviors?
ADL12: Um, I would say not change, but just more um, like as far as reminders and like encouragement just to keep, keep doing the exercise and healthier eating and all that.
INTERVIEWER: Um and are there any foods that you tried that you had never had before that you had read in one of the text messages? Read about in one of the text messages?
ADL12: Um, I'm not sure if it was text or Facebook, but there was a post about um I think the post was about doing like chips with like I think it was actually about collard greens?
INTERVIEWER: Mhm.
ADL12: But I made um kale chips. Um after reading that post.
INTERVIEWER: Did you like them?

ADL12: Um, they were okay. I like saw a million recipes on Pinterest of like different ways you can flavor them and stuff.
INTERVIEWER: Mhm.

ADL12: So I think I made sure I like to have different flavors (INTERVIEWER: different flavors) and see if some of them are more or less… Um I also used like I got the pretty like cut up kale, so I think that it might have worked better if I had like the bigger that I like could make into pieces.
INTERVIEWER: Mhm.
ADL12: Cuz some of them were like…
INTERVIEWER: Tiny tiny?
ADL12: Yeah. So um they weren't the um I don't, ideal size (laughs). But I did try it. And my mom really liked them so.
INTERVIEWER: Well that's good. Um let's see. And so you had mentioned that you did watch some of the videos um I'm assuming on a computer?
ADL12: Both.
INTERVIEWER: Both.
ADL12: On my phone too.
INTERVIEWER: Okay. Um so comparing I mean I know the phone screen is a lot smaller but phones can be more convenient. So if, since you participated in the Facebook group and the text message group, is there a way that, do you think it matters that we sent links for videos to uh both places? Or would one be better?
ADL12: Um I think for me, just depending on what I was doing when I got the text…
INTERVIEWER: Mhm.
ADL12: would determine whether or not I would click on it. Like if I was home and not really doing anything, then I would. But um you know if I was out and about or whatever, I usually wouldn't. whereas when I checked Facebook I was like consciously checking it because I had time to do so.
INTERVIEWER: Mhm.
ADL12: So I think I would be more apt to watch a video link through Facebook than through text.
INTERVIEWER: That makes sense. And did you ever share any of the information that you got through Facebook or text with um friends who have hildren or are currently pregnant?
ADL12: Um, not not that I can think of.
INTERVIEWER: Okay. Um and have you joined any email text message Twitter blog or other Facebook groups about pregnancy?
ADL12: Um I ave the app on my phone the what to expect I hthink it's called. And so I there's like a group of people all due in August. Um and then I get weekly emails. I thnk that's through babycenter. And like you know development and all that.
INTERVIEWER: um and so, if you had to pick one or the other would you prefer to receive text messages about prenatal health or messages through Facebook?
ADL12: Hm that's a good question. Um I guess I would say text just because I didn't ever miss a text, just because it came to my phone. Whereas if I don't go and check Facebook then I might miss something on Facebook.
INTERVIEWER: And do you have Facebook on your cell phone?
ADL12: Yeah.
INTERVIEWER: And so do you have it set up so that you get notifications when new things pop up?
ADL12: Mm mm (vocalization for no).
INTERVIEWER: Oh okay.

ADL12: No, I don't get any notifications on any of my apps. That's one of my pet peeves.
INTERVIEWER: Um and based on your experiences is there a person or a place, so that could be a book or um an Internet site. Is there one place that has provided you with the best information on pregnancy?
ADL12: Um I would probably say just from my first pregnancy. The what to expect when expecting book kind of, I feel like has been around for a while and covers pretty much anything and everything um related to pregnancy if you do like actually read it all.
INTERVIEWER: And do you think that that book is more helpful than advice that you got from any one person?
ADL12: Um, I would say yes just because it's so comprehensive. Um I think you know any advice I got is just kind of here and there. Um, so.
INTERVIEWER: And is there a person that you feel like you can go to with any questions?
ADL12: Um I usually just ask one of my sisters, I mean they both have multiple children. My one sister's a nurse, um so typically if I had any, I did experience a fall down the stairs so I called my sister and asked like, I felt the baby move, I don't really think…She was like, you need to call your midwives, they're gonna want you to come into the hospital. And sure enough they did, but otherwise I don't think I would've gone cuz not as a big of a deal as they said so.
INTERVIEWER: And everything was fine I hope?
ADL12: Yes.
INTERVIEWER: That's good.
ADL12: Thankfully.
INTERVIEWER: Um so, what would you say a good number of posts would be to add to either Facebook or send through text message? It can be I guess, per week, per day, however you prefer to answer.
ADL12: Um I think the text messages…did they do one a day?
INTERVIEWER: Sometimes it was one a day, and then (ADL12: Okay.) towards the end it was less.
ADL12: Okay. Cuz I um, I kind of thought they weren't one a day, but days go by and I don't realize um…
INTERVIEWER: It's just been like the last two weeks that they haven't been daily.
ADL12: Okay. Okay. I like um the every day text messages.
INTERVIEWER: Mhm.
ADL12: Um.
INTERVIEWER: And so, even though you were in Facebook too, then you were actually getting like two a day? And then that still didn't feel like too much?
ADL12: No.
INTERVIEWER: Okay.
ADL12: Cuz I still would you know go on my blog and read what those posts are and so. No.
INTERVIEWER: Um do you think that it would be best for healthcare provider to manage something like the Facebook group or send the text messages, or do you think that it matters who is sending it?
ADL12: Um, I mean I think the important thing is is that it's just someone who is making sure that the information is validated. Um…
INTERVIEWER: Mhm.

ADL12: You know like, they're not just Googling third trimester workout and sending out that link, like they actually, you know are making sure that it's you know not exercises that are contraindicated, (INTERVIEWER: Mhm) or um. So I don't necessarily think it would have to be a healthcare provider, um, but just as long as it's someone who's actually you know, like educated about pregnancy and making sure that the information that they're sending out is correct.
INTERVIEWER: Mhm.
ADL12: But.
INTERVIEWER: And do you think I mean being in a study I feel like maybe people assume that that it is that way and that the people have the right credentials to be providing the information.
ADL12: Uh huh.
INTERVIEWER: But is there something that would've helped make sure that everyone knew that you know that everything came with a stamp of approval? So to speak.
ADL12: Um I mean I guess if you had you know a healthcare provider, then that would kind of make it seem more legitimate so to speak.
INTERVIEWER: Mhm. But you didn't ever feel like any of the information that was provided was not safe or okay.
ADL12: No.
INTERVIEWER: Um…I think those are all my questions actually.
ADL12: Alright.
INTERVIEWER: Um is there anything that we talked about earlier that you thought of another answer to that we could go back to before we wrap up?
ADL12: Um, no. I think overall I found them both helpful and convenient.
INTERVIEWER: Well thank you.
ADL12: So yeah! (both laugh)

RDS027 and 031 Focus Group
Participants completed the post-interview together due to time constraints and clinic hours.

INTERVIEWER: Ok, so its been a while since we've talked, and I just want to catch up on a few things to see how things have been going. So tell me a little bit about how your pregnancy has been going since we last talked.

31: I've been eating less. (dietary changes)

INTERVIEWER: Eating less? How come?

31: Cause I get full fast.

INTERVIEWER: Yeah, that definitely could happen. Do you eat more snacks because of that?

31: Yeah.

INTERVIEWER: More meals, too?

31: yeah

INTERVIEWER: How about you, RDS27?

27: I don't know, I've just been tired. Yeah. I don't eat less. I gained eight pounds.

INTERVIEWER: Since the last time we talked? Or… your last appointment? That's good though, right?

27: No!

INTERVIEWER: No? How come?

27: Because I eat like… I think I eat too many like… snacks. And fatty foods, probably.

INTERVIEWER: Like what kinds of foods?

27: Like fried chicken and stuff like that. Like fried foods.

INTERVIEWER: Has that changed since early pregnancy?

27: Yeah. Cause I didn't really care for like, fried foods. But now, I like them.

INTERVIEWER: Has anyone ever said that certain cravings mean certain things at all?

27: I don't really have cravings. I just… like I used to, but I probably like fruit the most out of anything.

INTERVIEWER: Any particular kinds?

27: Mm mm.

INTERVIEWER: So, uhm, you're eating the same amounts, and you think you're eating a little bit less, but maybe more times throughout the day. Has anything changed in the types of foods that you're eating, RDS31?

31: No, not really.

INTERVIEWER: Not really? Uhm… anything else that has happened during your pregnancy that's maybe influenced how easy it is to get the foods that you do want to eat or uhm finding time to do things for yourself?

31: No, still the same.

27: I'm confused on what you just said.

INTERVIEWER: Oh, okay. Sorry. Sorry, I probably said too many things at once. So uhm, so… what you're eating has changed a little bit. Has anything else changed that… any stressors, anything that's making things hard for you? Easier for you?

31: No.

INTERVIEWER: What about social support, friends, family… do you think you're getting the support that you need.

31: Yeah, too much support.

INTERVIEWER: Too much support? How can you get too much support?

31: Cause people be doing too much, sayin too much. Like you're not eating enough, you're supposed to eat like chips and stuff. So why aren't you eating it, and… yeah I know you're trynna help me out, but you're doing too much.

INTERVIEWER: Anything people have said that you've found helpful and made you feel special at all or more like the chip comment?

31: My family be doin too much. They got smart mouths. I can't eat around them.

INTERVIEWER: Uhm, so, up to this point, what has been the best part of pregnancy?

27: Sleep.

INTERVIEWER: Sleep? Are you sleepy more?

31: No.

27: It's hard to sleep, but I like to sleep.

INTERVIEWER: Do you feel better you've taken a nap, or?

27: Mhm.

INTERVIEWER: How much do you think you're sleeping on a daily basis?

31: I could sleep all day.

INTERVIEWER: All day?

31: And I'm anemic so…

INTERVIEWER: So that makes you tired? Yeah. How many hours do you think, on average?

31: Two? I don't know, man. Cause I have dark curtains so I don't even know if it be light outside or it be dark outside. Like usually when my mom cooks it'd be after 5 o'clock. So… prolly like.. .I don't know. I would sleep for like an hour, wake up, eat something, then go right back to sleep. Like the hours change.

INTERVIEWER: Throughout the day and night?

31: Mhm.

INTERVIEWER: Okay. Are you still in school, RDS31?

31: No, tutoring.

INTERVIEWER: And how about you, RDS27?

27: Mm mm (no).

INTERVIEWER: Not school? And last time you said you had a job, right?

27: Mhm.

INTERVIEWER: Are you still working?

27: I'm out of work right now.

INTERVIEWER: Ok. Uhm, and then how many hours of lseep do you think you're getting each day?

27: I don't know. Probably like a lot cause I sleep a lot. Cause when I sleep, I sleep, then I get up… probably find something to do for an hour, eat, then go right back to sleep.

INTERVIEWER: Ok, so would you say that you're both sleeping three quarters of the day? Half of the day?

31: Mhm.

INTERVIEWER: Total? Three quarters?

27: Yeah.

INTERVIEWER: When you are up, what kinds of activities are you doing?

31: Staring at the wall, reading a book, reading a baby book, watching TV…

27: Yeah, I prolly watch TV or cook.

INTERVIEWER: Do you ever go for walks or uhm…?

27: Yeah, I walk around the mall, but that's about it.

INTERVIEWER: But no planned exercise or anything like that?

31: My legs are my transportation.

INTERVIEWER: Your legs are your transportation? Well that's good, though. Uhm… And last time I asked this question and I'm just curious again what your answers are. I had asked you what your hopes and dreams are for your unborn baby and I just wanted you to share your hopes and dreams again or how you imagine the future for your baby.

31: I just hope my baby healthy and… in the future, like, what we want in the future for the baby or?

INTERVIEWER: Mhm

31: I don't know. Iunno.

27: Well I hope for a healthy baby. I ain't trynna have no preterm labor, none of that premature labor and then… hope she get the education that she need. You never know how it could turn out. So…

INTERVIEWER: That's kind of some of the excitement, too. You don't know and you get to hope… (laugh) Uhm, so what have you each done throughout your pregnany to kind of help make sure that your baby's born healthy? Cause that's an important thing to both of you.

27: I try to eat healthier and get a little more exercise cause I'm just lazy.

INTERVIEWER: Can you tell me more about that? Like the specifics?

27: Like exercise, I didn't like walk… cause where we walked at, we just sit and use our phones so… I didn't have much exercise. And I would get off school late. I would just go shower and go to sleep and then… that'd be my day. Like I didn't really move around too much. Now, I'm active. I get up, I cook, probably go walk around the mall and… that's probably all the walking I do. That's probably it.

INTERVIEWER: That still counts though. And you said trying to eat healthy. Could you tell me more about that, too?

27: Yeah, like… ok. Like… before my father cooked… but when I cook, I just cook what I want to eat. And then, when he cook… he try to put like… he said he can't eat like two starches on a plate, like that. So I guess that's my way of trying to eat healthier. Well he try to help me out but, I don't really… I don't know, think too much about starches. I don't look at that stuff.

INTERVIEWER: Are there any particular foods that you try to eat more of that you think are healthy?

27: Probably like. I don't like too many vegetables. I'll probably eat like, broccoli or… carrots, corn, like. Other stuff… I've tried like cauliflower, those white things. It was okay. I didn't eat it cooked, though. I don't know if you cook those. I ate it plain like, that was alright. But I don't like too many vegetables. Like peas, I don't like those.

INTERVIEWER: And you said you like fruit a lot, so have you been eating more fruit?

27: Mhm.

INTERVIEWER: Okay. How about you, RDS31? What have you changed to make sure your baby's healthy?

31: Iunno. Like I said, my feet is my transportation. Like I'll walk like.. uhm I did that before I was pregnant so uhm… and I eat healthy and stuff like that. I just… iunno. I try to eat a little bit more, but I try not to stuff myself so I don't throw up. Cause I don't want my baby to be like small… stuff like that. Cause then he'll have to stay in the hospital longer than he supposed to. I ain't trynna have no skinny baby.

INTERVIEWER: (Laugh)

31: No, forreal. I'm not trying to have a skinny baby. Cause I'm small and my baby father is skinny so… but y'know. Like I said. I ain't gaining that much weight. I probably gained like 20 lbs out of my whole pregnancy, and that's basically in my stomach, in my breasts. So…

INTERVIEWER: Well at least you know then that it's at least mostly going to the baby, so that's a good thing, right?

31: Yeah.

INTERVIEWER: Uhm, anything else that you can think of that you made a decision to change or do that you think is good for your baby?

31: I started taking this uhm, this tea junk.

INTERVIEWER: Like a drink?

31: It got a lot of vitamins and minerals in it, and uhm. Raspberry Leaf Tea.

INTERVIEWER: oh ok. Is it a mix or the little bags of tea that you…

31: They got capsules and they got tea, so you can take either one.

INTERVIEWER: Mhm.

31: But uhm, they said like.. .it's healthy for a person that's not pregnant and a pregnant woman too so… it don't matter like if you take it or not if you pregnant, but it's good. And it causes contractions too, so it can help your baby come out. It'll help you go into labor.

INTERVIEWER: Do you remember what nutrients are in it that are most helpful?

31: No, I read it like one time. I read like all that stuff one time, and then I forgot about it. But all I know is that it's like healthy and good… and doctor recommended to pregnant woman.

INTERVIEWER: Ok. Did your midwife recommend it?

31: I asked her about it today. She's like, "Yeah. You should…" I had already started taking it… last Saturday so.

INTERVIEWER: And do you drink it once a day or more than once a day?

31: I drink like a capsule… I switch out sometimes. The capsules… I take like three once, twice daily. Or the tea I drink like 4 to 6 cups a day.

INTERVIEWER: That's probably nice when it's cold out. How about you, RDS27? Anything else you can think of that you've changed?

27: Mm mm (no)

INTERVIEWER: No? Okay. Uhm… and as far as good preparation goes, who's the person that's preparing most of your meals?

31?: Me or mom or both or grandfather.

27?: The same.

12:50

INTERVIEWER: Same? Okay. And… has how you prepared foods changed? I know you kind of mentioned that a little, RDS27, about the starches on the plate. Uhm anything else that's changed how you've prepared foods or the types of foods that you're preparing?

31: I try to put like Brussels sports and…

INTERVIEWER: Those are my favorite.

31: I know, I love those. I try to eat those every meal I have. They're so good.

INTERVIEWER: That's good. Uhm, and so, for fried foods, uhm, how have fried food changes… fried foods that you eat changed?

27: I think I ate fried foods more than I did before.

31: I eat fried chicken, or nothing fried in like… four months I think.

INTERVIEWER: Okay, so… what kinds of meats are you eating?

31: Steak.

INTERVIEWER: Fried steak or baked steak?

31: Baked steak.

14:03
INTERVIEWER: What about salt? Salty or sweet foods? How have those changed?

31: Sweets I eat a lot of more sweets but salt… I don't.

27: I'm I'm sorry. I just oversaid I ????
14:23

27: But I know best for salt?? It fills me up and stuff.

31: I like sweets at night time.

INTERVIEWER: Any particular kinds of sweets that you girls like?

27: Yeah I like the lemon heads, stuff like that.

INTERVIEWER: Sour stuff?

27: Yeah, Mikes and Ikes and….

31: Yeah, I like fruity candy. Not too much chocolate.

INTERVIEWER: Uhm and.. have you changed the amount of dairy foods that you're eating at all?
14:53
31: I probably didn't.

27: I prolly have more cause now I'm not workin and everything so… I prolly have more time for dietary foods and… especially my mom bringing it cause she work at the hospital and she work at the dietary area. So she would bring mad stuff home and everything I be eating.

INTERVIEWER: What kind of stuff?

27: Uhh I don't know. She putting a lot of stuff together. Like she be… she be putting a lot of together. Like she can make a sandwich and there be poppin mad stuff on there. I aint be paying attention to what there be on there, I be eatin it. So.

INTERVIEWER: What about milk? Are you guys drinking milk at all?

31: Mhm.

INTERVIEWER: More milk or less milk?

31: I probably drink less milk. I used to drink like milk probably every day. I don't drink it as much. I eat yogurt a lot… a lot of yogurt.

27: More.

INTERVIEWER: More?
16:02
27: Yep. Cause I don't know how to say it??? But WIC????

INTERVIEWER: Do you eat yogurt too?

31: Mhm.

INTERVIEWER: How about cheese. No cheese? Not so much?

31: Unless I eat a grilled cheese. That's it.

INTERVIEWER: A good cheese?
31: A grilled cheese.

INTERVIEWER: Oh, sorry. Yeah, those are good. Uhm, so… for cravings, have you had any nonfood cravings?

31: I've been wanting to drink Fabuloso.

INTERVIEWER: What's Fabuloso?

31: Pinesol, stuff like that.

INTERVIEWER: Oh, cleaner?

31: Yeah, I've been wanting to drink that.

INTERVIEWER: I know that you have mentioned clay last time too. Uhm, still?

31: Yeah.

INTERVIEWER: Are you taking anything for your anemia?

31: Yeah. Iron supplements.

INTERVIEWER: And that's going okay?

31: Yeah.

INTERVIEWER: Do they bother you at all?

31: No. Uh uh. But if I don't take them, I be asleep all day. Somebody might think I be dead. Cause you get really really tired like…

INTERVIEWER: Mm. How about you, RDS27? Anything you've had cravings for that are maybe…?

27: I just like to chew plastic. Like straws.

INTERVIEWER: Ok. How about ice? Do you like chewing ice?

27: Mhm.

INTERVIEWER: Is that something that you've always liked to chew on, or is that more during pregnancy?

27: mhm.

INTERVIEWER: More during pregnancy?

27: Mhm

INTERVIEWER: And kind of along the same lines, how about smells? Sometimes pregnant women dislike or really like different smells. Has that happened to you at all?

27: Probably in the beginning, not now.

31: The Fabuloso smell.

INTERVIEWER: What does it smell like? Lemons?

31: Iunno. It just smells like.. .when something just smells good. I mean I just go for any human. You know I'm saying? You smell something and all your braining is telling you or all it knows is that it smell good. And when something smells good to the brain, well the brain tellin you know, your body that it smell good. Of course I'm gonna be like, "oh, I want to eat it. Drink it." Cause, it's good. That smell is good to you or…

INTERVIEWER: But you've had more of those feelings now that you're pregnant now than before?

31: Mhm.

INTERVIEWER: Uhm… and have there been any challenges that you've faced… in food access? Or anything?

27: No.

INTERVIEWER: No? So plenty of food at home.

31: mhm.

INTERVIEWER: And… in terms of weight gain, last time I had asked you if you were aware of any recommendations that exist and I know that… you had said that your midwife had talked about, y'know, whether you are gaining the right amount of weight or too much, just to let you know how you're doing. So, have they been giving you updates on that?

31: Mhm.

INTERVIEWER: How's weight gain been going?

27: It took me a long time to gain weight throughout my pregnancy, but at the end that's when I gained… started gaining at weight because at first, well two appointments ago, I didn't gain any weight then… and at the last appointment, I didn't gain any weight. But this appointment I gained 8 pounds.

31: I gained like one or two pounds.

INTERVIEWER: Total?

31: No!

INTERVIEWER: Just in like the last few weeks?

31: Mhm. But my baby been measured, though.

INTERVIEWER: That's good. How did they measure the baby? Did they measure your belly?

31: Like they just be feeling like… Your baby could be like… this size… I'm guessing like… it's probably this size or whatever. But I might have to get an ultrasound or something to make sure cause I don't think I gained a lot of weight. But… heheh. Like I said, me and my baby father mass small, so she prolly gonna come out skinny.

INTERVIEWER: heheh. Uhm… so… based on how much you've gained, do you think that's a healthy amount or an unhealthy amount?

27: I think mine's unhealthy cause it's probably coming from a lot of fatty foods and sweets. 20:44

31: I'm good. At least I've gained at least 20??? pounds. I'm good.

INTERVIEWER: Have you ever heard of anything that how much weight you've gained can have a positive or a negative impact on the baby?

27: Mhm.

INTERVIEWER: What have you heard?

27: The sound??? That you've been sending in the mail.

INTERVIEWER: Do you remember what it was?

27: I don't know. You just… it was just a paper like.. if you like, I unno. If you start off before you go pregnant whatever if you start like, overweight, you should gain less and then… if

you like underweight you should gain more. It said that if like you eat too much, that I don't know. Iunno. They just said it was unhealthy for your baby. I don't know why.

INTERVIEWER: Do you think that those are helpful guidelines, though?

27: Mhm.

INTERVIEWER: Okay. And have you heard of anything that weight gain do to the baby, positive or negative?

31: Mmm. I don't really know. I just know that sometimes, it could be healthy… I know the doctor was telling me like, the more weight you gain. IF you were like, if you weigh more and you gain a lot more weight that I would have a greater risk and would have to get a C-section or something like that.

INTERVIEWER: And was weight something you worried at all during your pregnancy?

31: (incoherent.)

INTERVIEWER: You said yes? What kinds of things did you worry about? I know you said--

31: I don't know like you said something… ???? pounds and everything. And… but they said I'm fine though.

INTERVIEWER: 20 pounds is a good amount to gain.

31: Okay now I'm just saying the same thing over and over and—

INTERVIEWER: It's fine! Sometimes you think of other things, but uhm, and… okay. So, changing the question a little bit, so you've been receiving messages. Have any of those messages changed how you view your weight gain? Some girls feel worried that they're not going to lose the weight afterwards and… how has that been for you? Are you worried about that?

27: I'm fine. Like when I'm not pregnant, I'm active. Like I walk a lot. But I think I just got lazy. Cause I drive… but. In the summer, I walk the majority of the time.

INTERVIEWER: Okay. How about you, RDS31?

31: I'm gonna pop back in shape. My weight gonna go back down… and. Yeah.

INTERVIEWER: So you're not too worried about weight? That's good. Uhm, so… Do you think that you've changed any exercise or eating habits for the better?

…
INTERVIEWER: What are those?

27: Wait you said what again?

INTERVIEWER: Positive changes that you've made in exercise or eating.

27: Well… I walk so… I'm used to that. So. It ain't much changes, but I know that I keep doing it so I can be healthy and everything and… just keep on exercising.

31: Probably eating. That's about it.

INTERVIEWER: Okay, uhm..

24:45
INTERVIEWER: And… so RDS27, you are receiving text messages and RDS31, you are getting Facebook and you got a couple of things in the mail. How did receiving those messages uhm, affect you?

And if they didn't you can say that. Don't be shy. It will not hurt my feelings.

27: It probably made me have a more positive outlook on the way that my diet was trying to change… so my eating habits or… mm cause really when I came here, I was so tired and things like that, some of the things that—the doctor told me some of the things that may have caused me to be like so tired was… could have been due to the sweets, eating too many sweets or some of the stuff that I ate. Or lack of exercise or maybe I just needed more sleep or… mm now, I probably now will try to eat more healthier than before and actually care more because then, it didn't mean as much to me. And that's probably about it.

INTERVIEWER: How about you, RDS31? What influence did the messages you received have?

31: Well… I unno but what you writing, the stuff on Facebook… man, I talk ghetto. I'm sorry. I about to say jump and all that. Uhm, I don't know but y'all just writing and helpful things on Facebook like… it just opened my eyes to like like there is like a lot of uhm… important things and… uh changes that you have to uh… I can't explain it! Iunno like.

INTERVIEWER: Things that you should do? Like just pointing out things that you should do? Or..

31: I can't explain it (laugh). Iunno there is just a lot of important things that you should know about pregnancy and… like what things you should eat, cause that's important like. People just be like, be pregnant and don't care like about what they eat. They just be like," Okay… I don't care."

INTERVIEWER: Like the eating for two sort of mentality?

31: Yeah, and eat healthy. Like you should wanna be—you should wanna be healthy even if you're not pregnant but I just… some people…

INTERVIEWER: Do you think being pregnant is a good motivator to try harder at being healthy? For all women?

31 27: Mhm.

INTERVIEWER: And so, we talked about eating healthy. What about some of the physical activity messages? Were there any things that you changed because of those messages?

27: I don't think I exercise any more except like walking. Like I live in an upstairs apartment so I walk up and down the stairs, so ususally I like go so… that's about it. Unless I'm at the mall walking around. Otherwise, no. I need more exercise.

INTERVIEWER: Did you ever try any of the yoga moves that we sent in any of the messages?

27: Mm… no. I don't really try too much that… Iunno. My body be sore.

31: And you have to be flexible.

INTERVIEWER: Well if you're sore, some of the yoga moves are more to help with back pain or if your legs are sore, they just sort of stretch the muscles out so they don't hurt as bad. So you don't have to be flexible for all types of yoga. Advanced yoga, those are the crazy moves. Those you kinda do. But these are very simple things.

31: I know but for some people… like for some people, people could be stuffed up like… not stuffed up but like… like stuffed up knee. Like I can hardly move because I… my baby is just.. like all you feel is. I don't know. I just can't move a lot because I feel stuffed up and… people that eat be stuffed up with food and their baby, so that's just putting more weight on to them so… and they got to carry that. So either way it goes, people that's underweight and people that are not underweight.

INTERVIEWER: And is that something that got worse? The moving around as you got bigger? Has it got harder to be moving around?

27: Yeah, for me, cause I feel a lot of pressure like down there. Like when I walk, the little body like my stomach at the bottom, it just feel like. I don't know. It's harder to walk. It's just harder like… it didn't hurt before.

INTERVIEWER: Same? Different? What do you think?

31: I been feeling a lot of pressure since like the fifth month anyway cause my daughter head was already down there like…

INTERVIEWER: Just hanging out, trying to squeeze your bladder?

31: Yeah she was just already down there like… they already said her head was already down there. So … and she was stubborn too, so. Like. She got scared and moved. Like why you gotta be moving? But it ain't much room to move around because I'm tiny.

INTERVIEWER: (Laugh) Uhm, so let's talk a little bit about access toFacebook and text messages, whichever you are receiving. Obviously if you didn't get the other, you wouldn't have access to it but… uhm, so… did you have your own phone during the study? And that's how you got your messages? …. SO you did and?

31: Facebook.

INTERVIEWER: So how did you access Facebook?

31: Phone.

INTERVIEWER: Your phone, or someone else's phone.

31: My boyfriend's phone.

INTERVIEWER: And how often do you think that you checked the messages that you got?

31: Whenever I could. Like, whenever I could… cause I got tutoring, and then I'd be asleep… and I don't be around my boyfriend that much anyway cause he work 24/7 so…

INTERVIEWER: Okay. How about you, RDS27?

27: Probably not often because I used to have my phone with me all the time but now, I just leave it in the car or wherever.

INTERVIEWER: So when you look at the messages, how much did you focus on them? Did you just sort of glance at them, or did you spend time reading them or clicking on the links?

31: Mhm.

27: Like reading them and clicking on things.

INTERVIEWER: Okay.

31: Usually if I didn't get on in awhile, I'll just… I'll just read every little thing that I ain't seen before. Or if I ain't read it I'll go down the page and read everything.

INTERVIEWER: And so when they had the little web addresses after a message, how often do you think that you clicked on those?

31: If I don't do it, I do it the next time. It depends on if I'm in a rush or not.

27: Mhm. I probably just look at everything cause I don't know. It interests me… some of the things probably interest me.

I unno. The majority of everything. I am learning new stuff so… everything. And I like to read so it didn't really bother me.

INTERVIEWER: Did you like when the messages were short and sweet or a little bit more detailed?

27: Probably detailed cause I like to ask a lot of questions so… I don't know. If it be a little bit, and I probably have questions, I just be asking myself well, "I wonder this, or I wonder a lot and it probably don't' give enough details so I won't have so many questions.

INTERVIEWER: What about you, RDS31?

31: Uhm…

INTERVIEWER: Do you want me to repeat it? … I Don't even remember what I said now. Must've been a lie. Uhm, well kind of…

31: Oh, do you like when the messages are short or… detailed?

INTERVIEWER: Short or more detailed messages?

31: More detailed.

INTERVIEWER: More detailed? So if I—if you would've received a shorter message and it had the little web address that you know, you clicked on it, it takes you to a website, how often would you click on those little web links?

31: You just asked us that didn't?

INTERVIEWER: But I don't think you really answered it.

31: Oh, I said yeah I did!

INTERVIEWER: Every time?

31: I said it depends if I'm in a rush or not. If I'm like just trying to check up on the page or if I'm just sitting there and I know I'm going to be by that person for a long period of time, I'll go to the website. But if I'm in a rush or something, and it's not my phone, then… I won't.

INTERVIEWER: Is it easy to read those websites on your cellphones when you have access to the internet and it's fast? I know this phone, it sucks. It takes like ten years so… but those sites they show up okay? Okay.

Do you have any favorite messages that really stood out to you?

31: That pear message.

INTERVIEWER: The pear message.

31: I can't get that out of my head.

INTERVIEWER: That's what I do in my office in my free time.

31: Really? (laugh)

35:02
INTERVIEWER: You have to make fun out of the little things in life. Did you get that? It might've only been on Facebook. It was a picture of a pear that I had printed out little eyeballs and I glued the eyeballs to the pear right on top of the stem, so the stem looks like it's nose…

27: I don't think so.

INTERVIEWER: On the Facebook site, I had posted it's okay to play with your food (laugh) You're never too old for that. Uhm, anything else that's stood out?

31: Iunno. I liked how you, how you had the multiple choice. You asked a question and … that was good. ??? like writing a big paragraph or stuff like that.

INTERVIEWER: Yeah, but you like participating but if it's short and sweet, it won't take a lot of time or…

31: Yeah, cause I won't finish it or I won't do it. Like, period. So it's better, Iunno.

INTERVIEWER: No, that's helpful though.

31: yeah, so like I can at least write something down, it don't make me seem like I'm ignoring the page. So I try to…

INTERVIEWER: Are there any messages that motivated you to try something that you hadn't tried before?

31: Kinda fill my plate up with vegetables. Uh, half.

INTERVIEWER: Did you succeed? Sometimes?

31: Man… mm mm. no. I'm not going to lie to you, nope. But like I said, I try to fill it with Brussels sprouts or everthing that I eat.

INTERVIEWER: That's good though. Or at least you were thinking about half your plate as fruits or vegetables, so every little step counts I think. How about you, RDS27?

27: Yeah, I try. It's just certain vegetables I just won't eat. But like, not all vegetables I hate so, it's been…

INTERVIEWER: And fruits count, too. The half can be fruits and vegetables, so… uhm, thinking back, cause you've gotten a lot of messages. Are there any that you didn't like or didn't think were helpful?

31: Everything is helpful.

INTERVIEWER: How about the way that they were written?

31: Yeah, I was cool with it.

27: Yeah, it's fine.

INTERVIEWER: Fine? Well, if you had a friend who became pregnant, do you think that the messages we sent would be helpful to them to have a healthy pregnancy?

27 31: Mhm.

38:03

INTERVIEWER: And I know we kind of talked about access to cellphones and Facebook, uhm but if you think about in just a weeklong period, if you werent' checking your messages every single day, how many days out of the week do you think you checked or how many times a week?

27: Probably like, it used to be every day for me, but now I probably do like.. every other day. Iunno. I don't really pay attention to my phone too much any more.

INTERVIEWER: Okay.

31: Twice or three times a week.

INTERVIEWER: Okay. Do you think that sending daily messages is too many messages?

31: No

27: Not really, cause even if it's just on there, I'll check it like when I go look at the phone.

INTERVIEWER: Okay. Andu hm, RDS27, did you like having messages where there were questions and there was the option to respond?

39:05
27: Yeah, but I don't know like… when I be on the phone, that's why I don't have it too much. But sometimes I'll be on the bed and like I read through questions, and then… I didn't really respond to them. Like I didn't really respond. I don't know if I forgot. My mind is just bad…

INTERVIEWER: No, that's okay. But do you think that if there was one that was really interesting to you that… then you might have responded?

27: It's not that it wouldn't be interesting, it's just that I have a bad memory. Like I could… read it, and then get sidetracked, and I get up and do something else and I forget. But, Iunno.

INTERVIEWER: But you're not opposed to it. Okay. Do you think any of the messages had a really big effect on your pregnancy?

27: Yeah, they are beneficial to me like… I don't know. It's something that can help me or help me change something that I'm doing or for something positive, it doesn't really… it's not anything that would affect me. It's all good to me.

INTERVIEWER: How about you, RDS31?

31: It's all good to me.

INTERVIEWER: All good to you?

31: Mhm. (incoherent)

INTERVIEWER: What?

31: It shouldn't affect me but (incoherent)

INTERVIEWER: Affect what? I'm sorry, I can't hear you.

31: I said it shouldn't affect anyone.

INTERVIEWER: It shouldn't affect anyone like in a negative way? So they only have the potential to have a positive effect?

31: Mhm. I'm just saying… I don't know what there is to…
27: What's so negative about it.

INTERVIEWER: Well I mean some people might not like receiving messages or they might not think that some of them are really useful information.

31: But ain't nobody gonna come out of nowhere and start sending somebody messages. They either have to choose to receive them or… if you don't want to be bothered, don't give the number up.

INTERVIEWER: Well, yeah, that's true. It's always good to ask.

27: We got a lot of pages left.

INTERVIEWER: No, this one and the other one. But some of these I already asked so I'm skipping over them.

41:22

INTERVIEWER: Uhm, did any of the messages make you do a Google search for something that sounded interesting?

31: I like to Google everything.

27: Right? Who don't, though?

INTERVIEWER: I know, Google should be a word in the dictionary because people always use it as a verb. "I Googled that…" Have you ever shared any of the information that we sent to you? Uh with a friend or family member?

27: Yeah, my mom knows.

31: yeah, like my baby father he like to… I don't know. He like asks me a lot of questions, so instead of me answering all the questions, I just let him look at it, too.

INTERVIEWER: Yeah?

31: Well he still asks questions but…

INTERVIEWER: And have you joined any other sort of messaging programs for information, like a Facbeook site or twitter account, an e-mail account, anything like that?

31: No, it's just this thing that come to my boyfriend phone, though. Text Baby or some junk.

INTERVIEWER: Text for baby? What did you think of those?

31: He always be trynna read them to me. Like he be like, "Ohhhh look look look look look. They send them to me!" And I be like, "Lemme see." And he be like, "No."

INTERVIEWER: Oh.

31: Like just…

INTERVIEWER: So you never got to see any of them?

31: No! I'm just like what's the point of… like, "I need to know these things. You're pregnant." Well, I do, too. He just like… I don't know. He just like to read them for himself. And then like, I don't know. I be trying to read them after, but he still be saying "no."

INTERVIEWER: Do you guys get along with your baby's fathers okay? Happy? Both working hard towards waiting for baby to come? All that stuff?

27 and 31: Mhm

INTERVIEWER: and you think it would be helpful if messages like these came from your midwife or one of the health care providers at the Culver Clinic? Uhm… anything else that you may have thought of but didn't get a chance to say about the messages or your eating or the exercise activities?

27 and 31: no

INTERVIEWER: you think you're ready to have these babies and watch them grow up.

27 and 31: Mhm, yep.

INTERVIEWER: Alright.

RDS029

Interviewer: This interview will be really similar to the last one that you did, it will be 30 to 45 minutes and we are going to talk about how things have been going with your pregnancy and if you've tried any new dietary or, um, exercise things and, um, like usual, I will record the interview, so I don't miss anything that you say. Anything that you say is, there is no wrong answer, so whatever you think or feel—there is no wrong answer. So, don't be shy if I ask you something—and you think it is stupid, you can tell me it's stupid, it won't hurt my feelings. So, um, sound good?
RDS029: Hm-hm.
Interviewer: Ok. So, it's has been a while since we have talked, so I just wanted to catch up on things that were happening with you. And I will start with some really general questions, um, about your pregnancy. So, how have things been going with that?
RDS029: Pretty good.
Interviewer: Any exciting happenings or anything that you didn't expect?
RDS029: Um, no, I don't think so. Everything has been moving along.
Interviewer: Well that's good. Um, what would you say has been the best part of pregnancy so far?
RDS029: Um, just looking forward to having a baby. And every day I'm closer. And it gets more and more exciting and feeling him kick.
Interviewer: Are you all ready for the baby?
RDS029: Yes.
Interviewer: Do you have a place set up for the baby to sleep and everything?
RDS029: Yea.
Interviewer: That's good. Did you pick a theme at all for the baby's room?
RDS029: His room doesn't have a theme. It has colors.
Interviewer: Which colors?
RDS029: Um, like light green.
Interviewer: Oh pretty. That sounds nice. And last time we talked, I had asked you what your hopes and dreams are for your baby. And, um, you had just pretty much emphasized that said that you were hoping that the baby is healthy and happy and I just wanted to see if you felt the same or added anything on to that.
RDS029: Yea, I just want him to be happy and to love his family.
Interviewer: And did you pick a name yet?
RDS029: Yes, I did. But, we are not telling anyone until he's born.
Interviewer: That is exciting. Surprises are always fun. Okay, we'll change topic then. And, we'll talk a little bit about food. So, how's your diet been since the last time we talked? Has your eating habits changed at all?
RDS029: Um, if anything, it'd probably gotten better.
Interviewer: How so?
RDS029: Because, I don't know, because I used to eat a lot more fast food I think. Like a few months ago.
Interviewer: And what are you eating now?

RDS029: Um, normal food (laughter)
Interviewer: Normal, like, closer to how you ate before you were pregnant?
RDS029: Yea, closer. I still don't eat as healthy. But, you know, I have chips and stuff sometimes.
Interviewer: That's okay. Chips are good. Um so, how often are you eating meals and snacks?
RDS029: Um, I eat breakfast, lunch, and dinner, and I have a snack usually between breakfast and lunch. Lunch and dinner and then after dinner and before I go to bed. Because I get really hungry in the middle of the night.
Interviewer: And is that different from earlier in pregnancy?
RDS029: No, I don't think so.
Interviewer: So, still eating about the same number of meals and snacks every day?
RDS029: Yea.
Interviewer: And um, just kind of going back to what you said about eating less fast food. And you kind of described that you are eating more normal now. Um, when do you think that changed?
RDS029: Ah, maybe like a month ago … or two.
Interviewer: Was there anything that made you make the change or did it just happened?
RDS029: Um, well, we are just trying not to spend a lot of money, so we don't buy food out anymore. I mean, um, what did I say. We are trying not to spend a lot of money, so we don't eat out anymore um, and I don't know, I'm just trying to make sure that it's not so hard to start eating better again after I'm not pregnant.
Interviewer: That makes sense. And you said we. Would you describe who we is?
RDS029: Me and [name redacted].
Interviewer: Ok. Did your family eat out a lot or is that not kind of a normal thing for your family?
RDS029: Um, we don't really eat out like a lot. Mostly we just ate dinner at home.
Interviewer: Um, and so you mentioned fried foods. Oh, no, you said chips. But, eating out—so, a lot of times there is fried food, so how have maybe you're, how much fried food do you think you are eating now compared to earlier?
RDS029: Less.
Interviewer: Do you have an example of maybe what you would have eaten when you were still eating out versus now?
RDS029: Um, before we probably ate out like once a week, but now it's probably only like one or twice a month.
Interviewer: And what were your favorite places to go?
RDS029: Ah…I don't know. I was like, I liked curly fries and uh, chicken nuggets.
Interviewer: So, curly fries, I know that they have those at Arby's.
RDS029: Yes.
Interviewer: Yea?
RDS029: That is where I get curly fries.
Interviewer: Yea, they are good. Um, what about your salt and sugar consumption. Has that changed at all?
RDS029: We're talking about since the last time we talked right?
Interviewer: Um, mostly, but whatever helps you jog your memory. You can talk about your whole pregnancy, it doesn't matter for me.

RDS029: Ok, um yea, it has probably changed a little. Cause I don't eat out anymore.
Interviewer: So, do you ever crave candy or ice cream or anything?
RDS029: Chocolate and my parents got me an ice cream maker for Christmas, so I make ice cream now.
Interviewer: Nice, that is exciting. What about dairy foods, are you still eating dairy?
RDS029: Yes.
Interviewer: How much dairy do you think you eat a day?
RDS029: A lot. I drank a lot of milk. Like, probably like 6 cups a day. Like, measuring cups.
Interviewer: And just all throughout the day? Or do you just consume all of that in like one or two meals?
RDS029: Well, I usually have cereal in the morning with milk and then, I have milk with like one or two meals a day.
Interviewer: And what about cheese or yogurt?
RDS029: I don't really like yogurt, but I eat cheese.
Interviewer: And have you had any cravings since the last time we talked or what I would call non-food items? Like dirt, clay, paper?
RDS029: No.
Interviewer: No? Have any smells appealed to you? Or made you feel sick to your stomach at all?
RDS029: Um, no.
Interviewer: And, have you had any challenges specific to finding food or getting the foods that you are craving at all?
RDS029: No.
Interviewer: Plenty of food in the house. And whatever you want, you are pretty capable of finding somehow?
RDS029: Yea.
Interviewer: Ok. What things have you done in terms of your diet to make sure that your baby is born healthy?
RDS029: Well, I drink a lot of milk. And I make sure I get my fruits and I eat a lot of fruit too.
Interviewer: Has that changed at all over the course of pregnancy? Or are you still eating about the same amount of milk and fruit?
RDS029: Well, I think my milk has probably increased. I don't know why, I just drink a lot of milk now. I don't think I used to drink that much. But, um and,… fruit—I don't know, I just make sure that I have fruit every day, I won't go a day without fruit. So, I don't know if I used to do that before. Probably not. Probably just
Interviewer: How much fruit do you think you eat a day?
RDS029: Hmm, one or two pieces. Like servings, like two servings probably.
Interviewer: And what kind of fruit do you eat most often?
RDS029: Apples and bananas.
Interviewer: Um, ok, so changing to the new topic. Where do you find the best advice for pregnancy? Or, where have you gone to look for information?
RDS029: I go online a lot.
Interviewer: Are there any websites that are most helpful?
RDS029: Uh, babycenter is where I usually end up going to. And, then I ask my doc.., my midwives and stuff.
Interviewer: And has anyone in your family been able to give you any helpful advice?

RDS029: Um, yea, my mom.
Interviewer: And is, how often do you talk to your mom do you think about that?
RDS029: Um, not very often. Uh, about pregnancy?
Interviewer: Yea.
RDS029: Yea, we don't really talk about it.
Interviewer: How come?
RDS029: I don't know. Because I don't have any questions I guess? Cause she is like, she's old, so we like grew up, so like things are so different.
Interviewer: Um, but, do you feel like your family's been supportive and helpful throughout this experience?
RDS029: Yes.
Interviewer: That is good. And so, in terms of the internet, how often do you think you visited websites like Babycenter?
RDS029: Oh, all the time. Like every day.
Interviewer: Anytime you think of something and want to know the answer, you pop on the computer?
RDS029: Yea.
Interviewer: Then you have internet access pretty much all the time?
RDS029: Yes.
Interviewer: So, you have a home computer with internet, and what about a cell phone that has internet?
RDS029: Yea, I use my IPhone.
Interviewer: Ok. And it has a data plan?
RDS029: Yea.
Interviewer: Ok. Um, is there any type of information that you look for more often than other topics when you were online?
RDS029: Hmm, I don't know. I…um, I don't know.
Interviewer: Um, so, if you had to compare and the lists of topics that you were looking up for were about information about delivering the baby and kind of what to expect with that or exercise and nutrition, which of those do you think you searched for more often?
RDS029: Delivering the baby.
Interviewer: Has nutrition and exercise been a big concern of yours during pregnancy?
RDS029: Um, well, I'm a pretty healthy person. And I, I exercise, well I did. I'm pretty big now, so it's hard. But,um… What was the question?
Interviewer: Um, so, we were saying that you are pretty healthy, that's what it sounds like, so maybe you were more concerned about what to expect just in relation to pregnancy. What it's like to deliver, what it's going to feel like. And maybe you were less worried about what you should be eating and how much you should be exercising, is kind of what I got.
RDS029: Um, yea, I'm not really worried about because, cause… Well, I have done pregnant exercises to do and stuff, but my, um, my midwives always gave us sheets and stuff of what we should be eating. So, I wasn't really looking it up because I already knew. And I think it's basic.
Interviewer: Um, yea, that makes sense. Do you, can you give me some of the types of information that your midwives shared with you?

RDS029: Um, they like, they gave us like 3 or how many servings of each food group we should have. Um, If we had like constipation, what we could eat. If we had lack of iron, um they gave us a list of foods. Yea.
Interviewer: And is that something that they just give to you or is that something you asked for?
RDS029: They gave it to us.
Interviewer: And did you get that in the group meetings or in your individual meetings?
RDS029: In my group meetings.
Interviewer: Ok. And you were enrolled in the Facebook site for this study. And so, I was curious how helpful you thought this resource was?
RDS029: Um, well, I don't think I saw all the posts. And it didn't notify me when they were up. They, they were just on my newsfeed. And so, I didn't always know when it posted, because I don't use Facebook that often. Um, but, I think I knew a lot of the stuff that was already said, it said.
Interviewer: That is good. Um, so, if you, the things that you did see, do you think that it would be helpful for someone who doesn't maybe know as much about nutrition and exercise?
RDS029: Yea.
Interviewer: Um, and are there any messages that you did see that stood out to you that you remember?
RDS029: Um, no, I can't remember.
Interviewer: That's okay. Um, was there anything that you didn't like about the messages that you did see?
RDS029: No.
Interviewer: And I know occasionally there were links attached to the messages. Have you ever click on any of those?
RDS029: Um…I think I did once or twice.
Interviewer: Do you remember which messages those were? If it was exercise or nutrition related?
RDS029: I think exercise.
Interviewer: And do you think that any of the messages that you did see influenced any, um, of your own personal behaviors?
RDS029: No.
Interviewer: Okay. And any messages that you saw that you disagreed with?
RDS029: No.
Interviewer: And given the places that you have looked online do you think that there is a better source or a source that we could use to help us to give the best information on a site like that?
RDS029: Like a website?
Interviewer: Hm-hm.
RDS029:Um, I don't…, I just go to Babycenter, I don't know, it has some pretty good info I guess.
Interviewer: Yea, I have been there. It looks nice. Um, and so, some of the Facebook messages were about weight gain and I'm just curious what your thoughts are on weight gain during pregnancy. Is it good or bad or doesn't matter? What do you think?

RDS029: It's good to gain weight during pregnancy because that is what your body is supposed to do. But, if you are overweight then… Ok, if you are normal weight, then you should gain 25 to 35 pounds, around there, and if you are underweight, you should gain more than that. And if you are overweight, you should gain less than that. But, weight gain is normal.
Interviewer: And do you know why they have those ranges?
RDS029: Um, well, cause your body is supposed to like store water and fat I think when you're pregnant, so when you're underweight, then you don't have a lot of fat and then you don't store a lot of water. I guess.
Interviewer: Yea, there are no wrong answers remember? So, is there anything else that you've learned about weight gain during pregnancy?
RDS029: Uh, no. Well…
Interviewer: I'm sorry. What?
RDS029: No, you can say what
Interviewer: Ok. So, you mentioned that someone who's overweight should not gain as much as someone who is normal weight or underweight, so from what you've read, what happens in a woman who is overweight and gains more than she is supposed to? Or even a normal weight person who gains more than the 35 pounds that you have said?
RDS029: You could end up with diabetes, gestational diabetes, and it makes breathing harder when you gain extra weight. And you are not as fit.
Interviewer: Have you heard of how it affects the baby at all?
RDS029: Um, I mean it probably is, I don't know though.
Interviewer: And how do you feel about your weight gain?
RDS029: Um, I'm… my weight gain is normal, so I'm okay with it.
Interviewer: That's good. I know sometimes for girls it could be hard, gaining weight, since you see the magazine covers for skinny, beautiful people –so, I'm just curious how being young and gaining weight kind of influences how you feel about yourself. Has that changed at all?
RDS029: Um, no, I don't really care what my body looks like anymore. Just as long as my baby is healthy and it's kind of motivated me after the baby, I will want to the lose the weight and so, it's, it's, it's yea… It's probably just motivated me to want to just start exercising a lot again and get back in shape.
Interviewer: And have you heard of anything that you can do after you deliver that can help you lose weight faster?
RDS029: Um, just eating healthy, breast feeding, um and walking and stuff. And once you are ready to start exercising again, then you can start exercising again.
Interviewer: And do you have any plans for any sort of regular exercise?
RDS029: Um not yet, but I'll probably get into a routine.
Interviewer: Any specific activities that you think that you would be probably look forward to doing?
RDS029: I want to start running again, cause I used to jog.
Interviewer: Anything else?
RDS029: Just exercising in general. Like crunches, I can't do crunches.
Interviewer: Yeah, that would be kind of hard.
RDS029: Yeah.

Interviewer: And, I know we kind of already talked about this, but just in case you think of anything else, uh have you changed any other eating habits? I know you were saying you were eating healthier again, but can you think of anything else that you might have changed during your pregnancy that you have not already told me?
RDS029: Um, well, I, um, in my first trimester, I really badly, because I was so sick, I only wanted like restaurant food and then, I got better. I think I have gradually got better and then I eat, I eat more now than. Oh, I know, here's something. Ok my first trimester I was really hungry, like really hungry and then second trimester, it kind of went again like somewhere down a little bit, but now I'm really hungry again. And I...
Interviewer: Oh, sorry, go ahead.
RDS029: And uh, oh yea. I didn't drink coffee at all in the beginning of my pregnancy, I didn't want it. But, I drink it a little bit now. Like, maybe like two times a month.
Interviewer: Do you drink it because you're tired?
RDS029: No, just cause I like the taste.
Interviewer: Did you drink coffee before you were pregnant?
RDS029: Yea.
Interviewer: I can't survive without my one cup a day, at least one cup I should be honest. Um, so, what about preparing and shopping for food? I know you said that your mom does most of the shopping, but are you involved in that at all?
RDS029: Yea, I go with her a lot and pick out what I want.
Interviewer: And I remembered that you said that you like to cook and try out different recipes, so do you help out with preparing food at all?
RDS029: Sometimes.
Interviewer: I know that you are probably busy with school and everything must be hard.
RDS029: Well, me and Adam help sometimes.
Interviewer: Anything that you like to make the most, or favorite recipes?
RDS029: Um… no.
Interviewer: Um, I know you kind of mentioned this, but just in case you have anything else, you said you haven't exercised as much, now that you are bigger, but and obviously before you were jogging, but are there any other activity or exercise things that you changed that you could think of?
RDS029: From before I was pregnant?
Interviewer: Uh-huh.
RDS029: I was a cheerleader and I played volleyball. Um, so, I don't do those anymore. And, I ran, I ran up until I was probably like 5 months or 6 months. Um, then, I stopped and yea, I was walking for a little bit, but it's just gets harder now. Because I go to school, my knees get tired out from walking the hallways and stuff, so I don't really exercise anymore cause I'm busy and often I don't really have the energy.
Interviewer: Yea. And you said that you've looked up a little bit of exercise on, uh, BabyCenter? Do you remember what any of those were or did you try anything that was on there?
RDS029: Um, It must have just said that walking around is really good during pregnancy, so yea, and kegels – but, I don't really do them cause they feel weird.
Interviewer: And um, I've seen a lot about yoga, have you ever looked up that or tried any yoga moves?
RDS029: No, I've never done yoga ever in my life.

Interviewer: Um, and so you talked about increasing your exercising after you deliver and what do you think motivates you to want to do that?
RDS029: Well, I'm just really excited to not be pregnant anymore cause it's hard to move around and stuff, so I think just being back in my normal, normal-ish body and being able to bend over and stuff, I'll be just happy to do that, so I'm want to move around and exercise.
Interviewer: So, another topic change, you said that you have an IPhone and is that your own phone or a family member's phone that you share?
RDS029: It is mine.
Interviewer: And, does it receive text messages?
RDS029: Yea.
Interviewer: And you can access the internet so, you can I guess go to Facebook if you wanted to?
RDS029: Yea.
Interviewer: Ok. But, I know that you weren't receiving text messages. And did you ever access the, um, BabyCenter website from your cellphone?
RDS029: Yea. I type the question in on Google and BabyCenter is always one of the option things.
Interviewer: Did you every click on any of the other options?
RDS029: Yes.
Interviewer: Do you remember what any of those are?
RDS029: No.
Interviewer: I'm just curious, so you know how, when you Google something you get tons of options that you can click on and lots of places to go. How do you decide which link to go to when you search for things?
RDS029: Um, whatever one is the closest to the question that I asked?
Interviewer: And do you ever worry that any of the sites might have bad information?
RDS029: Um, yea. Well, a lot of them have other moms saying their experience. Um so, I know that all of it isn't like fact because I don't know, but like, there are a lot of myths about pregnancy and I like to read them.
Interviewer: Yea, it is probably helpful to read about other people's experiences because they're going to know best about what something feels like. So, it sounds like you use a combination of both, what real people are saying and what then kind of like what the official website is saying?
RDS029: Yea.
Interviewer: Have you ever felt like the information other people were having been not accurate?
RDS029: No, I just think most, a lot of the information isn't true for everyone. So, maybe it's accurate for them, but not for me.
Interviewer: Ok. And so, between your phone and your computer, would you say that you search for health information on your computer more often than on your phone?
RDS029: Um, well no, I don't even use my computer at home.
Interviewer: So, you pretty much always search on your phone?
RDS029: Yea.
Interviewer: Ok. And do you every share any of the information that you find with any of the other girls in groups with you?
RDS029: No.

Interviewer: And, I know that some websites let you sign up for email services, where they'll send you an email and it keeps up with what date of pregnancy you are in, have you signed up for any email services?
RDS029: Um, I have, I have apps on my phone and I do that. But, not emails.
Interviewer: Ok. Can you tell me what apps they are?
RDS029: Sure, let me look. Um… oh, one's a BabyCenter app, but I don't know how to see the title because I don't know, it might just be BabyCenter.
Interviewer: Ok.
RDS029: Um, cause the name is too long. So, it doesn't show the whole thing. It starts, I think it's MyPregnancy. So yea, just BabyCenter.
Interviewer: And, just those two?
RDS029: Yea.
Interviewer: Have you ever, um, looked and blogs or Twitter sites at all?
RDS029: No.
Interviewer: And what about text message services?
RDS029: Um, well, I think that Lauren sends me texts.
Interviewer: Yup, a reminder about your Vitamin D pills?
RDS029: Yea.
Interviewer: Do you get text messages from anything else?
RDS029: No.
Interviewer: How often do your apps send you information?
RDS029: Every Monday when I turn in another week.
Interviewer: And so, it automatically sends that?
RDS029: Yea.
Interviewer: And then, are you also able to search for topics on there?
RDS029: Uh, I don't think there is like a search bar, but they have information.
Interviewer: Ok. With like links that you can just click on that are just on the front screen?
RDS029: Yea.
Interviewer: So based on your experiences, is there a place or a person that gives you the best information about pregnancy? And you can only pick one?
RDS029: I would say BabyCenter.
Interviewer: Over your, over you midwife as well?
RDS029: Oh well… well… uh, they are probably more accurate, but, but I get more information from BabyCenter I think. Well, maybe not. It may just be that I already knew the information because I was on BabyCenter. So, then they just tell me the same thing.
Interviewer: And, in terms of the Facebook group, I know that you said that you didn't get all of the messages, but given what you have seen and your experiences with the apps that you had, how many messages show we send a week for it to be helpful?
RDS029: Um, a few, like 3 to every other day or every day.
Interviewer: Ok. And do you think that that number would help another adolescent in the future to have a better health during pregnancy?
RDS029: Well, yea. It would educate them at least.
Interviewer: Ok. And, I know that sometimes it can be hard to stay motivated when you are tired and you are carrying around extra weight and you have school and everything going on, so in your opinion, I would love you hear your advice on what would be the best way to

motivate future pregnant teens to read the messages and actually try some of the messages that we send?
RDS029: To motivate them?
Interviewer: Hm-hm.
RDS029: Um, I don't know. If you have good recipes that taste good.
Interviewer: And what if the sky's the limit and we have tons of money to buy stuff and give prizes, do you think that that would be good motivator?
RDS029: Yea. Um, I don't know. Maybe if you promised we could get our pre-pregnancy bodies back. That would be a good motivator.
Interviewer: I'll have to figure out how to do that. That's, that's a challenge. But, a good one. Thank you, I like that idea. Just a couple more questions, so how often do you think that your midwife or other healthcare providers should give you information about pregnancy health?
RDS029: Well, on how you are doing with the messages?
Interviewer: Well, not necessarily. So, you were said that in groups they gave you handouts on nutrition and other things, so let's say that—I mean, do you get papers from them every time that you visit?
RDS029: Um, we got them almost every time. But, I don't go to group anymore, so I don't get them anymore. But, they used to give us papers on whatever we were talking about, whether it be breastfeeding or nutrition or exercise.
Interviewer: Do you think it would be helpful if they mailed you additional papers so you got them at home for you to read and in the in-between times when you didn't have appointments.
RDS029: Um, no, I think what they gave us was enough.
Interviewer: Ok. And would you recommend using text messages and Facebook as a way of reaching teens in the future?
RDS029: Um, yea. I think text messages are probably more read. Because you have to read them in order for them to go away usually.
Interviewer: And are there any other social media sites like Facebook that you think would be better to send messages?
RDS029: Um, well, I don't… My favorite is Instragram and but, I don't know how well you could send a message through a picture, but.
Interviewer: Any other sites that you can think of?
RDS029: No.
Interviewer: Ok—well that was my last question for you. So, I just want to open it up really quickly to, if there was anything else that happened to your head that you kind of want to add to any of the other questions that you gave?
RDS029: I don't think so.
Interviewer: Ok. Well, thank you very much [name redacted].

RDS030

INTERVIEWER: Alright. It's been a while since we last talked and, I wanted to catch up on what's been happening with you and so I'll just start with a few basic questions. Um, so the first one is, how has your pregnancy been going?

R: I'm good.

INTERVIEWER: Good? What do you mean by good?

R: Like, I don't have pains or, like, nothing like that, so, to me that's good.

INTERVIEWER: That's good. And all your appointments have been going well?

R: Yep

INTERVIEWER: And everything's progressing as everyone had hoped?

R: Yeah

INTERVIEWER: Perfect. Um, so, what has been the best part of being pregnant?

R: Uh, when I feel my baby move for the first time.

INTERVIEWER: Yeah? When did that happen?

R: When I was 6 and a half months.

INTERVIEWER: Wow, that's so cool. Was she moving a whole bunch? Or just a little bit?

R: No, just a little bit.

INTERVIEWER: Just a little?

R: Yeah

INTERVIEWER: Did she move a lot since then?

R: Yeah

INTERVIEWER: Ever get like a karate chop?

R: No

INTERVIEWER: No that's good. Um, so the last time we talked, I asked you what your hopes and dreams were for your baby girl. Um, so last time you told me that you hadn't really

thought a lot about it and that you kinda just live in the moment. And I was wondering if you could tell me if that's the same or if it's changed and if it has, what changed?
R: It's the same.

INTERVIEWER: The same?

R: Yeah.

INTERVIEWER: Um, so have you changed any of the ways that foods are prepared since you last talked?

R: Um, my mom bakes the chicken now so.

INTERVIEWER: Okay. Did you help her find any recipes for that or did she just?

R: No I just, like, sometimes when I eat foods with oil, it makes me like nauseous.

INTERVIEWER: Ah.

R: So, she just stop frying stuff.

INTERVIEWER: What other things have you had that you've kinda changed to not frying them.

R: Just like the meat.

INTERVIEWER: Just the meat?

R: Mm-hm. Cause that's the only thing we fry in the house.

INTERVIEWER: Okay. Gotcha. Um, so what about foods with salt or sugar? Like potato chips or candy.

R: I haven't had any in a month.

INTERVIEWER: Whoa.

R: Yes.

INTERVIEWER: Just cause you weren't craving it? Or?

R: I dunno, I just haven't had any.

INTERVIEWER: Okay. Um, but you still have them in the house, so you could have eaten them, they were available?

R: Yea.

INTERVIEWER: Okay. What things have you been eating more of then?

R: Like fruits.

INTERVIEWER: Yeah? What kinds?

R: Grapes, strawberries, oranges.

INTERVIEWER: Cool. And what about vegetables? Any change in your vegetable consumption?

R: Well they give more vegetables in school and I also…

INTERVIEWER: What kinds of vegetables?

R: Broccoli

INTERVIEWER: Yeah? Do you like broccoli?

R: Yeah, broccoli with cheese.

INTERVIEWER: That is good. Definitely best with cheese. Um, so what about dairy? Any increase or decrease in, you said you like cheese with your broccoli, so that's dairy. Um.

R: I drink milk everyday, cause you know school, they give you milk.

INTERVIEWER: So you don't get to pick your drink at lunch, it has to be milk?

R: No, unless, cause they have vending machines, so unless you just buy a drink from there.

INTERVIEWER: Oh okay. Um, do you eat breakfast and lunch at school?

R: Yeah.

INTERVIEWER: Okay, and milk at both meals?

R: Mm-hm.

INTERVIEWER: Do you ever eat yogurt?

R: No, I don't like the yogurt that they give.

INTERVIEWER: Okay. And what about ice cream?

R: Well yeah, at my house.

INTERVIEWER: At your house?

R: Yeah

INTERVIEWER: What's your favorite flavor?

R: Vanilla with like the chocolate syrup on top.

INTERVIEWER: Oh nice. Like the Hershey syrup?

R: But, yeah. But I don't eat chocolate ice cream. But I eat it like that.

INTERVIEWER: The chocolate syrup tastes a lot different than the chocolate ice cream.

R: Mm-hm.

INTERVIEWER: I'm in your boat. I'm a vanilla girl. Um, so in terms of any, like, cravings, any foods that you wanted to eat a lot of recently?

R: No

INTERVIEWER: Anything that you crave less? You mentioned oil. Oily foods kinda made your stomach upset. But anything else like that?

R: No

INTERVIEWER: Okay, and what about craving like non-food items? Like—

R: No, that doesn't happen to me.

INTERVIEWER: Okay.

R: I think that was disgusting. I know people that eat soap and stuff like that.

INTERVIEWER: It happens sometimes. Um, have you ever heard about, like, anemia, where your, you haven't gotten enough iron in your diet, and those people can crave like clay and dirt. And, it's weird. But yeah it happens.

R: Yeah. I know someone that used to eat the ashes of the cigarettes.

INTERVIEWER: Oh gosh. That doesn't sound great.

R: Yeah, that's nasty. That's so nasty.

INTERVIEWER: I'm glad that didn't happen to you. Um, so what about—so we talked about, kind of like, taste, but what about smells? Any smells that you like the smell of more of, or less?

R: No, to me, everything is still the same. Like, since before I got pregnant. Like, the same smell.

INTERVIEWER: That makes it easy. Um, and any challenges that you've faced during pregnancy? Let's say in relation to eating or exercise.

R: No.

INTERVIEWER: Okay.

R: I went up 6 whole flights of stairs today at school.

INTERVIEWER: Whoa. That's a lot.

R: Yeah.

INTERVIEWER: It's amazing how no matter how fit you are, walking up stairs is always hard. I think that everyday cause my office is on the third floor. And I'm always going up and down to meetings, and I'm like, can't breathe. Um, let's see. So what things have you, like kind of, in purposely done, to make sure that your baby is as healthy as possible.

R: Eat a lot.

INTERVIEWER: Eat a lot? Any specific foods that you tried to eat more of than others?

R: No.

INTERVIEWER: No? Just to eat a lot?

R: Yeah.

INTERVIEWER: And, when you say eat a lot, is there a reason for eating just like large quantities or I guess, let me rephrase. Um, so how many like meals a day are you eating?

R: Like from three to four.

INTERVIEWER: Three to four? And, what about snacks?

R: Like, two. But, like, sometimes I eat dinner like maybe like two hours later I feel hungry again, but usually I go to sleep. But I just eat something, just in case.

INTERVIEWER: Have you--

R: Cause it's not—sorry for cutting you off.

INTERVIEWER: No it's fine.

R: It's not like hungry, like your stomach is growling and stuff, but you know you feel that you need to put something in your stomach like that.

INTERVIEWER: Yeah? And it's better just to do it then than wait until it is growling.

R: Cause then in the morning, oh I be so hungry.

INTERVIEWER: I do not like that feeling. Um, so in terms of the number of meals and snacks—has that changed at all? Over the course of your pregnancy?

R: Yeah, I don't think I eat a lot of snacks now.

INTERVIEWER: Okay. So less snacks, but the meals are the same?

R: Well, I used to not eat breakfast either.

INTERVIEWER: Okay, but you, are you eating it regularly now?

R: Yeah.

INTERVIEWER: How come? Just cause you're hungry?

R: Yeah. And because I'm pregnant, so.

INTERVIEWER: Yeah. And have you heard anything about like what eating breakfast can do for your pregnancy?

R: No.

INTERVIEWER: No? What about for health or anything like that?

R: No.

INTERVIEWER: No. So, um, is that just a choice that you made cause you thought it would be better for your pregnancy?

R: Yeah, and because like you know when I wake up I be so hungry I gotta eat something.

INTERVIEWER: Yeah. It's hard to last to lunch, that's for sure. Is that something you'll keep doing?

R: Probably not.

INTERVIEWER: No?

R: Cause I don't like eating breakfast. And you have the taste of the breakfast in your mouth all day and stuff.

INTERVIEWER: That's true. Um, let's see. Have you noticed like the amount of food that you eat? Has that changed at all, like now that your belly has gotten bigger, have you felt like the meals have to be smaller?

R: Well, the reason why I eat smaller meals cause I get full quick.

INTERVIEWER: Okay. And did you always get full quick or is that more--

R: No.

INTERVIEWER: Okay. So that's just as you, baby's been growing, meals have gotten smaller. That makes sense. So, as far as, like, asking for advice about nutrition or exercise, have you ever had any conversations with your midwife about um, anything in those areas?

R: No.

INTERVIEWER: No? Um, and we kinda talked about this last time so using the internet to find information. Have you ever looked up any information about pregnancy?

R: Well when you put the websites on there, I click on it.

INTERVIEWER: Okay. Do you remember which ones you clicked on?

R: No, not really.

INTERVIEWER: No, anything…

R: I also look at the pictures and one of them was like the wheel and I was looking at those things.

INTERVIEWER: The one that had the splitting of like vegetables and fruit and did you try that at all on your plate?

R: Kinda. Yeah.

INTERVIEWER: Kind of? It's hard to get it perfect every time.

R: Mm-hm.

INTERVIEWER: But, little, little steps that make the big difference I think. Um. So um, any other like not the exact websites, but like maybe like topics from the messages that stuck out to you? Like a favorite?

R: Like, I like when you send pictures and stuff, you know, I'll be reading all this stuff and things like that.

INTERVIEWER: So the pictures you like better than just the words.

R: Yeah.

INTERVIEWER: Okay. Yeah I'm like that too. A lot of them are really bright and colorful.

R: Yeah that's what makes you wanna click on them.

INTERVIEWER: Um, and so, for, when you do click the links, do you look on the phone?

R: Yeah.

INTERVIEWER: Do you have a computer at home?

R: Yeah. But my mom hasn't paid the internet bill.

INTERVIEWER: Okay, so just the cell phone internet. Perfect. It's easier that way anyways.

R: Yeah.

INTERVIEWER: Cause you just have the links already there.

R: And it takes forever to load, and the computer and stuff.

INTERVIEWER: I know, that's the worst. It's having to wait. We live in a

R: Maybe impatient—and just clicking and clicking—and that makes it go even slower.

INTERVIEWER: Totally know what you mean. Um, so, but you can't remember like specific ideas or things that have been sent that you clicked on. Do you think…How often do you think that you clicked on the links? Every time or?

R: Yeah, like every time you sent it.

INTERVIEWER: Okay, cool

R: Cause my mom always be like, oh here's a text.

INTERVIEWER: And, um, so some of the links have more writing than others and I'm really curious about like, I know it's a lot of reading, and sometimes I, ugh can't read that now.

R: I be like skimming through.

INTERVIEWER: Skimming? So, um, the ones that, if you had to pick though would you have a preference on like what kinds of links that people sent?

R: Like what do you mean?

INTERVIEWER: So, I sent a news article or um, if it was just a link to you tube video. Um, does it matter to you like what the type of attachment is or?

R: I would like videos and stuff.

INTERVIEWER: Yeah, videos?

R: Yeah.

INTERVIEWER: I think I sent some videos to you. Did you watch those?

R: I think so.

INTERVIEWER: Okay. Did you ever revisit a link in the messages? Or the one time you got it?

R: No. Just the one time when I got it.

INTERVIEWER: Okay. Um, and did you ever like try any of the ideas about like? I know there was one that said like switch to try to have mustard instead of mayonnaise, cause it has fewer calories.

R: I don't eat none of those.

INTERVIEWER: Okay. Anything else though that you though, "Oh I should try that. That sounds easy." Like, there were some recipes.

R: No.

INTERVIEWER: Okay.

R: Cause I don't like cooking by recipe, so.

INTERVIEWER: Yeah, sometimes it's easier to just see what you have and kind of. I call them hodge podge meals. I just throw things into a container.

R: And if you don't do the right thing, it doesn't come out right.

INTERVIEWER: Yep, I know, definitely. Um, so last time, we talked a little bit about weight gain too, and how like what you've talked to your midwife about and I was just wondering if you could tell me a little bit about how that went and what they've been telling you.

R: Well I don't really ask about that. But they tell me like, oh you gained a pound, or two pounds. But I don't really ask them about that.

INTERVIEWER: Um, do they ever tell you if you're gaining the right amount of weight or if you need to watch how much you're gaining? Anything like that?

R: Yeah, one of the midwives, they go to my school, tell me that my weight is fine for how far along I am.

INTERVIEWER: That's good. Um, and so, compared to like when you first became pregnant and kind of looking at how much weigh you've gained. How have your, like, kind of like personal image has changed or not changed?

R: Yeah, cause I feel like I'm fatter now. I'll be uncomfortable cause I don't like my weight or weight problems so yeah.

INTERVIEWER: So is that something that you thought a lot about during pregnancy and the choices that you were making at all?

R: Yeah, but I knew my stomach was gonna get bigger regardless so.

INTERVIEWER: Kind of happens, yeah.

R: Yeah.

INTERVIEWER: It's a good thing though. Cause if it didn't, that would be a problem. Um, so, is there anything that you did to try to make choices so that you kind of stayed in that healthy range of weight gain?

R: Not really, because everything like, at first everything that I used to eat then, I still eat.

INTERVIEWER: Mm-hm.

R: Like a lot of chips and candy and stuff like that. Yeah, I used to still do that in the first months and stuff.

INTERVIEWER: But then, more recently though you said you hadn't really been interested eating those things.

R: But I didn't start gaining weight until I was like 6 months.

INTERVIEWER: Okay. Um, are you, have you thought at all about like after you deliver?

R: Yeah, I'm gonna exercise.

INTERVIEWER: Exercise? What about breastfeeding?

R: Mm….

INTERVIEWER: No? Have you heard that that can help…

R: Yeah

INTERVIEWER: …you lose the weight faster? But still don't want to try?

R: Mm..

INTERVIEWER: Yeah. It's not for everyone, that's for sure. So that's totally fine. Did you gain the amount expected that would happen?

R: I didn't, I didn't really expect, like, how much I would gain. I just knew I was going to gain some weight.

INTERVIEWER: Yeah. So you mentioned that you were going to exercise after pregnancy. Do you have anything in mind for what you liked to do?

R: Well, they have like treadmills and like the bikes at my school so I probably do those.

INTERVIEWER: Are you in the Young Mothers Program?

R: Yeah.

INTERVIEWER: Okay, and you get to stay in that afterwards?

R: Yeah

INTERVIEWER: Oh, that's nice.

R: But I'm gonna graduate in January, so.

INTERVIEWER: Cool, congratulations. That's exciting.

R: Thank you.

INTERVIEWER: Um, what are your plans for after?

R: I'm going to college afterwards.

INTERVIEWER: Yeah?

R: Yeah.

INTERVIEWER: Where at?

R: Um [name redacted].

INTERVIEWER: Cool. Do you know what you're going to major in yet?

R: Um, like you know the ladies at the front desk? Like that.

INTERVIEWER: Uh-huh. Yeah. Oh that'll be really cool.

R: You know I speak Spanish so you know that

INTERVIEWER: That is a very useful thing

R: You know when the people come in and they don't speak English or they don't know certain things in English so.

INTERVIEWER: That's awesome. Aw, I wish you the best.

R: Thank you.

INTERVIEWER: So we talked a little bit about eating habits and changes and um, like physical activity, so after you kinda have plans to ride the bike and walk on the treadmills, and um, do you have plans to like, maybe go for walks with friends outside of school?

R: I'll probably be going that.

INTERVIEWER: Yeah?

R: But, not in the winter time.

INTERVIEWER: Yeah, no, it's way too cold here.

R: Cause in the summer time we used to always walk. I used to walk from Hudson all the way here.

INTERVIEWER: Yeah?

R: Mm-hm. And that's far.

INTERVIEWER: Did you get the message that had like a yoga pose in it?

R: I think so.

INTERVIEWER: Would yoga could be something good to do inside. Have you ever thought about doing that at all?

R: No, cause sometimes I would be uncomfortable sitting down and laying down so I just have to find somewhere to be comfortable.

INTERVIEWER: Yeah

R: I mean you know when you do yoga you gotta do that stuff.

INTERVIEWER: Yeah

R: It'd be tight up here and so I can't do nothing.

INTERVIEWER: What about after you deliver? Would that be something that you'd be interested in trying?

R: Yeah. I mean when you join the gym, usually they have that stuff so.

INTERVIEWER: Yeah

R: Probably

INTERVIEWER: That'll be nice. And do you know if the college where you're going to go to school has a. Sometimes they have like a fitness center.

R: I'm not sure, cause um, I was supposed to go to an interview to check out the college, but the guy told me to call them and I still don't call them.

INTERVIEWER: Oh okay.

R: Yeah

INTERVIEWER: Well that would be something good to ask. Like what sort of resources did they have. I bet they have a lot of cool stuff that you can check out. That'll be exciting. Um, did you, uh, change the amount of physical activity that you were doing while you were pregnant because of any of the messages?

R: Not really, cause like, I wasn't doing any of the exercises. But I went up the stairs today.

INTERVIEWER: Yeah

R: So

INTERVIEWER: Every little bit counts, so.

R: Yeah, I was thinking about going up the stairs at least once a day cause it's 6 flights. By the time I get to 31, I be like (heavy breathing).

INTERVIEWER: That definitely works.

R: Yeah my friends were gonna be like you're going to be in labor on the stairs.

INTERVIEWER: Um, let's see, what else do we have here? Do you think that any of the text messages had a big effect on your pregnancy?

R: Yeah.

INTERVIEWER: How so?

R: Like, some of them say information that I didn't know about.

INTERVIEWER: Do you remember like which ones those were?

R: Not really.

INTERVIEWER: No?

R: No, but like some stuff you do send, I be like oh like I didn't know that.

INTERVIEWER: Um, if I showed you a list of the messages that I sent you, would that help jog your memory?

R: Probably

INTERVIEWER: Can I do that?

R: Yeah

INTERVIEWER: Okay. Let's see. So these are kind of the, they go in order of dates so um, actually, I can let you scroll if you like, cause they go down a little bit further. It's kind of a gist.

R: Like you know the breakfast one.

INTERVIEWER: Mm-hm

R: I didn't know that. I just thought you gonna eat breakfast when you feel hungry.

INTERVIEWER: Yeah? Did you look at the cross-fit mom? Article? It was about a mom that does that cross-fit exercise when she was, I think like 8 months pregnant. There's a picture of her squatting with a big bar of weights on her shoulders.

R: I think so. I'm not sure, but I think so. See like the thanksgiving one. I was looking at that. And if you don't drink caffeine, you sleep longer. I didn't know that.

INTERVIEWER: Do you drink soda at all?

R: Yeah

INTERVIEWER: Yeah? After 3?

R: But not all the time.

INTERVIEWER: Yeah

R: But I do

INTERVIEWER: Did you, um, do you ever find that it's hard to sleep after you've had like soda or anything with caffeine in it?

R: Well I don't really go right to sleep after I'm done eating cause then when I'm laying on my back I start burping and it hurts my chest so.

INTERVIEWER: Yeah, no that definitely happens. What did you think about the poll questions where we asked you things? I know that you answered which is really awesome. Thank you for that.

R: Like that one right there?

INTERVIEWER: Mm-hm. I know you said that you really didn't care for dairy. But that's fine. Um, and um, was it helpful to get things like that kinda talked about, you got a picture that was whole grains and then there were definitions of iron and folic acid and--

R: Yeah I didn't know what none of those meant at first.

INTERVIEWER: So

R: Yeah the iron stuff, I didn't know what that was either so

INTERVIEWER: Normally they only talk to people about that when they have those weird cravings so it's good that it makes sense that they probably didn't talk to you cause you were doing pretty good all by yourself. Um, but, did the definitions make sense? And they weren't too hard to understand? Sometimes it's really hard to define some of those things.

R: Yeah.

INTERVIEWER: Um, did you ever try to eat grains that were whole grains? Like whole wheat bread or?

R: Well that was the only thing that was given at my school. Whole grain like pizza, they make it out of whole grain bread so.

INTERVIEWER: Do you like it?

R: Mm-mm.

INTERVIEWER: No? What don't you like about it?

R: I feel like they don't cook it all the way.

INTERVIEWER: Oh, it's still kinda doughy?

R: Yeah, so I don't eat it.

INTERVIEWER: Do they ever have sandwiches on whole wheat bread? What do you think about that?

R: Yeah, those are good, but I don't like the cheese so, I just tell her, don't put on cheese.

INTERVIEWER: How come?

R: It, that cheese to me tastes nasty.

INTERVIEWER: Is it American cheese?

R: I dunno. It's yellow and square, but I think that's fake cheese.

INTERVIEWER: It's possible, that's for sure. Um, but you definitely liked getting the pictures and videos you said would be your preference over written things and um, did you ever track your pregnancy weight gain at all on that one site that, I think that was sent at the beginning. It was, uh, this one. No?

R: But I know, um, I think I gained like 24 pounds or something like that. Cause I used to way 180 before and now I think I weigh 117 or 114 or something like that. Not one, but at like 217.

INTERVIEWER: Oh I knew what you meant.

R: Yeah. I just caught that.

INTERVIEWER: Do you think that the amount of weight that you gained during your pregnancy was influenced at all by the messages that we sent?

R: I don't think so.

INTERVIEWER: Okay. Um, and did the messages make you feel differently on how you thought about food or exercise? Make you look at things you were doing differently?

R: Yeah, some of them.

INTERVIEWER: Can you tell me about them?

R: Like, one of them say something about fast food was healthy. Something like that. I don't really remember. But I don't really fast food so.

INTERVIEWER: Do you remember if you clicked on that link?

R: I dunno.

INTERVIEWER: Okay.

R: But I seen it somewhere around here.

INTERVIEWER: Yeah. It was um.

R: Right there.

INTERVIEWER: It had some options that were healthier, so.

R: Cause usually, if you go to McDonalds you see the thing right here, see how many calories each thing. But I don't really pay attention to that.

INTERVIEWER: You just pick whatever sounds good on the menu.

R: I usually get chicken nuggets and fries. Cause when they do the burgers, they put all that sauce and all that extra stuff. I don't like that.

INTERVIEWER: Just plain chicken nuggets and fries.

R: With ketchup cause I don't eat barbeque sauce.

INTERVIEWER: Yeah, I like ketchup too. Um, do you think that your diet changed because of the messages? Or do you think it was just what you were currently craving?

R: I think like the oil stuff was because it didn't taste right to me.

INTERVIEWER: Mm-hm. What about the fruits and vegetables? You said that you were eating more of those. Do you think that was influenced by the messages or more by your pregnancy?

R: Oh it was influenced by the school. Cause you know, the school serves more vegetables now.

INTERVIEWER: Mm-hm. And do they require you to eat everything on your tray?

R: No

INTERVIEWER: Okay, do you normally eat them anyways though cause they're there?

R: Yeah, cause I eat broccoli and they have broccoli so. But when they make peas and stuff like that or corn. I don't eat that.

INTERVIEWER: And they still put that on your tray.

R: No, we don't want it, they don't put it on your tray.

INTERVIEWER: Okay

R: They ask you like what you want.

INTERVIEWER: And they have like different choices so you can pick which one you want?

R: Yeah

INTERVIEWER: That's nice

R: And they also have like salads in these little plastic containers.

INTERVIEWER: Mm-hm. Do you like salad?

R: Yeah. But I can only eat it with ketchup.

INTERVIEWER: No salad dressing?

R: No, I don't eat ranch or none of that.

INTERVIEWER: Well that's probably a lot healthier though. Cause ranch has a lot more calories than ketchup does.

R: The girls at my school. Oh they have like ten packages. For that one little thing. Doesn't bring that much salad, so, but they be putting all ten packets in there and shaking like this and

INTERVIEWER: And it's more like would you like a piece of lettuce with your dressing?

R: Right. It be all white. Like white white. And I just be like ugh…that's nasty.

INTERVIEWER: Um, do you think that the messages or the choices that you made for food and exercise have an effect on your baby?

R: Yeah

INTERVIEWER: How so?

R: It's not healthy if a lot of grease or fat. And um when you exercise it helps the baby.

INTERVIEWER: What have you heard about that? Anything specific?

R: No.

INTERVIEWER: You just know its good?

R: Yeah.

INTERVIEWER: And whenever you got a message and you clicked a link or it didn't click a link. Did you ever search for anything on the phone that made the message made you think about something and you googled it?

R: Not really.

INTERVIEWER: Okay

R: Cause when you google something, a lot of things pop up and then its probably not what you're looking for and you just get frustrated.

INTERVIEWER: Mm-hm. Um, and have you, do you have like any other friends at the clinic? Were you in one of the groups that they have here?

R: No, because my school doesn't get out until 3 so.

INTERVIEWER: Okay. What about in the young mothers program? Are you close to any of the girls?

R: They, yeah, at my school, every 2 weeks, on Wednesday, this lady comes from here and everyone that goes to that, they talk and stuff like that. She listens to our heartbeat and measure the stomach.

INTERVIEWER: Have you ever talked about any of the messages you got with any of the people in Young Mothers?

R: No, because they just started doing that so.

INTERVIEWER: Mm-hm

R: The next group isn't until next Wednesday, but I probably could bring some of the stuff up.

INTERVIEWER: What about the other girls that you kinda take classes with? When you're exercising, do you ever talk about experiences throughout your pregnancy? Swap stories?

R: Sometimes, it depends on what we're talking about.

INTERVIEWER: Yeah

R: Cause usually they be talking about sex all the time. Like you know how always uncomfortable to do this and that.

INTERVIEWER: Yeah. Let's see. So last time you told me that you had joined the babycenter.com site. So you get emails and um have you joined any other emails or groups?

R: No, just that one

INTERVIEWER: Just that one?

R: But I haven't checked it in like two weeks so

INTERVIEWER: Okay. Um, what kind of information have you found most helpful from that?

R: They send you like videos of people having their babies and stuff like that. Like when they put the epidural on the back. That's nasty. And they were pulling it out.

INTERVIEWER: But it's helpful just to kind of know what to expect.

R: And it tells you what size the baby is. Like it's as big as a watermelon. They say stuff like that.

INTERVIEWER: Did you think that was hard to believe?

R: Yeah cause I think one of them said it was as big as a grain of rice and stuff like that. And a piece of rice is like this big. And you be like the heck?

INTERVIEWER: And you see how big your belly is in comparison and you're like what? Um, and based on your experiences through your pregnancy. Is there a place or a person that's provided you with most helpful information about how to prepare for delivery and kind of what to expect?

R: Like the people here.

INTERVIEWER: The people here?

R: Yeah

INTERVIEWER: What about--

R: I don't really talk to my mom about that

INTERVIEWER: How come?

R: Me and my mom like we don't really get along like that so I just. I never acted like what she thought about me having a baby and like that.

INTERVIEWER: Okay. Um, but she cooks the food for you, and--

R: Yeah

INTERVIEWER: So it sounds like she's supportive in some ways. But you just don't talk about the kind of the private stuff.

R: Yeah. I don't feel like we have that type of relationship.

INTERVIEWER: Okay. Um, is there anyone else in your family that you are closer with on that level?

R: My sister. She had a baby and she's pregnant again so.

INTERVIEWER: Did she have lots of helpful advice for you?

R: Something like that.

INTERVIEWER: Or like the scare tactics where?

R: Yeah

INTERVIEWER: Oh okay. Have you ever tried to talk to her about anything you were nervous about?

R: No

INTERVIEWER: Not so much? What about friends? Any friends that you can confide in?

R: Yeah but, I don't talk to people about that. To me it's strange.

INTERVIEWER: So you just do it all by yourself? You're so strong.

R: Yeah

INTERVIEWER: I'm amazed

R: You know, it's just some things you just be like ugh I can't ask that person that. That's how I be feeling.

INTERVIEWER: Is there ever anything that you were worried about and needed advice or? No? You're just going with the flow. Um, so in terms of the number of messages you got. It was about one a day. With one day off a week. What did you think about the number?

R: To me it wasn't that many.

INTERVIEWER: Okay. So not too much, not too little? Um, if you had to pick a number what would you say the magic number would be?

R: Of how many did you send?

INTERVIEWER: Or no how many would be best to send so that it would motivate someone to make changes?

R: I say like one in the morning and afternoon.

INTERVIEWER: Okay. Um, and would it be helpful if these sorts of messages came from your midwife? Or do you think it matters?

R: No, it doesn't matter.

INTERVIEWER: Okay. And what kind of information do you think after seeing the types of messages that we send are most helpful for someone your age, you know who's kind of figuring things out and getting ready to have a baby.

R: I dunno. Cause you know everyone thinks different and not everyone you know learns the same so.

INTERVIEWER: Mm-hm. No that's definitely true. Um, so for you personally any of the types of information that were most helpful or things that you didn't know before?

R: Yeah, like, you know like the folic acid or whatever it's called iron pills. I don't…

INTERVIEWER: Sorry. I don't even know where it is. Oh my gosh, this thing is so loud and possessed. Okay let me turn this down. Okay, so sorry. Um, actually we're like really close to the end. Um, so I know that you received text messages, but we also offered Facebook as an option to receive messages. And so I know that you can only talk about texting. But do you think that's an effective way to reach pregnant adolescents?

R: Yeah cause people be on Facebook 24/7. So yeah.

INTERVIEWER: So what about texting?

R: Yeah, you know, like teenagers be on their phone all the time.

INTERVIEWER: Most everyone that you know has text message and often Facebook?

R: Yeah, yeah. They be just on their phone like. I can't do that, then my eyes start hurting or I get tired holding the phone.

INTERVIEWER: Um, and are there any other sort of apps like Facebook or ways of communicating with friends that you know of that might also be good ways to talk to teens about health and pregnancy.

R: Like twitter.

INTERVIEWER: Anything else? I know that they're always adding new ones so it's hard to keep up.

R: Instagram

INTERVIEWER: Instagram?

R: Some people check their emails so.

INTERVIEWER: Mm-hm. Do you have an email?

R: Mm-hm.

INTERVIEWER: Okay. And how often do you normally check it?

R: Like once a week.

INTERVIEWER: Once a week. Hey that's good.

R: But every time I go to check it, there be like 900 messages and stuff and the spam. It be the spam and stuff like that, like whoa.

INTERVIEWER: How do you weed through all that?

R: I just erase them all. I look at the side and if they're not important I just erase them.

INTERVIEWER: Yeah.

R: Cause you know usually they be junk mail. Also,

INTERVIEWER: That's true. There's a lot of that.

R: Yes, a lot.

INTERVIEWER: So in terms of participating in the study, do you think that this sort of thing would be helpful for clinics that have a special program for adolescents?

R: Yeah

INTERVIEWER: Okay. Um, I don't know where I was going with that. Um, anything else that you thought of about a previous question, that maybe I interrupted you or anything else that you can add to anything you said?

R: No

INTERVIEWER: Okay.

RDS032

Interviewer: Tell me a little bit how your pregnancy has been going since the last time we have talked.
RDS: Uh, it's been okay. I'm going through pains.
Interviewer: Oh, that's not good.
RDS: Yea.
Interviewer: Are they getting better?
RDS: Hm-hm.
Interviewer: That's good. You are getting ready to pop though. Are you ready to have the baby?
RDS: Yes. Yea. (laughter)
Interviewer: When do you think you're going to deliver? Right on time?
RDS: Hopefully.
Interviewer: Hopefully. Um, what's been the best part of being pregnant?
RDS: Nothing really.
Interviewer: Nothing?
RDS: No.
Interviewer: Are you excited about having a little baby though?
RDS: Yea.
Interviewer: Yea, that's good. Um, last time we talked, I asked you, um, what your hopes and dreams were for your baby. And I was just wondering if you could answer that question again and.
RDS: Just doing good things.
Interviewer: Doing good things?
RDS: Hm-hm.
Interviewer: Being healthy and happy? Are you having a boy or girl?
RDS: Boy.
Interviewer: Boy. Did you pick out a name?
RDS: Yea. Um, his name is going to be [name redacted].
Interviewer: Cute. I like it. That will be really cute. Um, okay. So, those are just kind of the intro questions. And now we'll talk about food. Cause food's always good. We all love food. Um, so, how has like eating been going?
RDS: It's been going good.
Interviewer: Good? Has the amount that you eat changed at all?
RDS: Not really.
Interviewer: So, you're eating, you think the same amount that you were eating at the beginning of pregnancy?
RDS: Well, it changed a little bit.
Interviewer: How'd it change?
RDS: Um, I'm eating more snacks.
Interviewer: More snacks? Same number of meals?
RDS: Hm-hm.
Interviewer: So, just really quickly, like kind of run me through like a, what a normal day is of eating meals and snacks.

RDS: Um, like get up, eat breakfast, then eat a snack. Get up eat breakfast, wait a little bit, um, probably eat a snack and then eat lunch, and then wait a little bit more, eat a snack, and then eat lunch or (pause) eat dinner, then a couple more snacks.
Interviewer: Um, are there certain things that you find that you eat regularly at meals and snacks or does it vary?
RDS: Vary.
Interviewer: Um, let's see, how has the, um, the amount of fried foods that you are eating?
RDS: I don't eat fried foods.
Interviewer: You don't eat fried foods? Um, what about foods that have, um, salt and sugar? Do you like those more or less?
RDS: Hm, less.
Interviewer: Less? What things do you like more now that you are pregnant?
RDS: Nothing really.
Interviewer: No cravings?
RDS: (I guessing she nodded her head no).
Interviewer: How about dairy food? Do you have a lot of dairy?
RDS: No, I'm lactose intolerant.
Interviewer: Ok.
RDS: So.
Interviewer: So do you, I know that you mentioned that you drank milk with your lasagna the other day, um, do you drink like the special milk that has the lactose intolerant stuff in it?
RDS: Hm-hm.
Interviewer: Ok. Um, what about like yogurt or cheese. Do those bother you?
RDS: A little bit.
Interviewer: A little bit? But, you can eat them, like, cheese on pizza and like, well not yogurt on pizza, like cheese on pizza?
RDS: Hm-hm.
Interviewer: Um, have you been craving any non-food items? I know that I asked you that on the other thing.
RDS: Not really.
Interviewer: Ok. And any smells that you like or dislike more?
RDS: No.
Interviewer: No. Um, any food challenges that you have faced? Like finding food or not getting the foods that you want to eat?
RDS: Not really.
Interviewer: Ok. And, so just thinking about food, what things have you done to make sure that your baby is healthy and happy?
RDS: Um, I don't know. Just… do basic stuff.
Interviewer: What is basic stuff?
RDS: Like, I don't know. I really don't know.
Interviewer: Have you heard of, like, any certain foods that are like good for pregnancy or not good for pregnancy?
RDS: Not really.
Interviewer: Ok. Um, if you had a question about what to eat during pregnancy, who would you normally ask?

RDS: My doctor.
Interviewer: Your doctor? Um, did you ever get to meet with the nutritionist at the clinic as well?
RDS: No.
Interviewer: No? Um, what about friends or family? Anyone?
RDS: My mom.
Interviewer: Has she been helpful for getting ready for the delivery and everything?
RDS: Yea.
Interviewer: Does she have any stories from when she was pregnant with you?
RDS: Not really.
Interviewer: And did she share any ideas about how to eat and when to eat?
RDS: (Guessing she shook her head no)
Interviewer: Ok. Um, did you ever use the internet to look anything up when you were able to talk to your doctor?
RDS: No.
Interviewer: No? Um, what about any blog sites or anything? You never Googled anything?
RDS: No.
Interviewer: How about text messages or like apps that you can get on your phone. Did you get any apps on your phone about pregnancy?
RDS: Hm-hm (as in no).
Interviewer: And, ok so specific to the Facebook site, um did you find the Facebook site useful?
RDS: A little bit.
Interviewer: Can you tell me more about what you thought about it?
RDS: It was okay. At first.
Interviewer: At first?
RDS: And then, um I don't know, it just start going through all the other stuff.
Interviewer: So, what was good about it at first? Like things that stood out and you liked?
RDS: Talked about how, um, what things to do when you are pregnant. Um, and it would say fun things. I don't know.
Interviewer: And then, it got not good?
RDS: Well it got a little bit boring.
Interviewer: It got boring. Ok. Um, do you remember like around time that it got boring? Or like a message specifically that you were like "man, that is a boring message"?
RDS: No. Not really.
Interviewer: Ok. Um, so how many times do you think that you looked at it before you kind of stopped looking?
RDS: Like once a day.
Interviewer: Once a day? And so, like how many weeks did you look and then stopped looking?
RDS: Um, at least like twice a week.
Interviewer: Twice a week for like how many weeks do you think you looked that much?
RDS: Um…I don't know. Like 4.
Interviewer: 4? And then, they got kind of boring after 4?
RDS: Hm-hm.

Interviewer: Ok. Um so, when you think about the messages that you liked in the beginning and then how they got boring, was it just, was there something specific like about the topics or the number of posts?
RDS: A little bit the number of posts.
Interviewer: Too many?
RDS: A little bit.
Interviewer: Ok. What do you think a good number of posts would be?
RDS: Like 3 or 2.
Interviewer: Oh, per week?
RDS: Yea.
Interviewer: Oh. And in terms of like being boring, are there any topics that you think would have been more interesting to hear about?
RDS: Not really. I don't know. Not really.
Interviewer: Was the Facebook something that such like you were busy and there just wasn't a lot of time for it?
RDS: A little bit. I usually didn't, when, when I was looking at it, I wasn't really looking at it, cause I was doing something else. Doing other things, so that's why I just stopped looking at it completely, cause getting busy.
Interviewer: Yea, so it was just like really hard to read the messages and really get what they were saying cause you were getting so busy doing other things.
RDS: Yea.
Interviewer: Um so, kind of thinking about what it was like to receive the messages and like whether or not that they were helpful, um how I'm trying to think what I'm trying to say here, I lost it. No, it's not your fault. Um, I guess like if you are busy, like what things take up a lot of your time that kind of the Facebook messages are competing with? Cause I know that you have just so many hours in the day.
RDS: Cleaning, making sure that I got everything ready.
Interviewer: Ready for what?
RDS: The baby.
Interviewer: Ah, yup, that is important. Very important. Um, are there any things that you are worried about for your baby or for your own health that we could have provided any messages about?
RDS: Not really.
Interviewer: Ok. And of the messages that you saw, so there were ones that were internet links and just sentences, sometime pictures, sometimes videos, which type of message would you like the most or did you like the most?
RDS: The sentences.
Interviewer: Just the sentences? Short and simple? Um and, so why would you prefer sentences over pictures or videos?
RDS: Um, simpler to read.
Interviewer: Ok. Um, so of the messages that you saw, were there any favorites messages that stood out to you?
RDS: Not really.
Interviewer: Any messages you saw that made you uncomfortable or unhappy?
RDS: Hm-Hm (as in no).

Interviewer: And, did you change any behaviors like how you were eating foods or exercising at all from the messages?
RDS: Hm-Hm (as in no).
Interviewer: Is there anything that would have motivated you more to try some of the ideas?
RDS: No, not really.
Interviewer: Ok. And did you ever disagree with any of the messages that you did see?
RDS: Hm-Hm (as in no).
Interviewer: And um, how's your view of weight gain during pregnancy? Positive or negative?
RDS: Good.
Interviewer: Good? Good how?
RDS: Um, gaining some weight and then, just that is it, gaining some weight.
Interviewer: Have you heard that there is a wrong or right amount to gain for a healthy pregnancy? And have your doctors been talking to you about that for your pregnancy? What did they say?
RDS: I had to gain between 10 and 15 pounds.
Interviewer: Hm-hm. And is that how much you gained?
RDS: (I'm assuming she nodded her head yes).
Interviewer: Well, that's good. Um, what about physical activity? How was your physical activity compared to now?
RDS: It was okay.
Interviewer: Are you being less active or more active now?
RDS: Less.
Interviewer: What type of activities did you do before?
RDS: I went outside and I used to basketball all the time.
Interviewer: And what kinds of activities do you do now?
RDS: Nothing. Just stay home.
Interviewer: Stay at home? What do you do at home?
RDS: Um, nothing really.
Interviewer: Watch TV?
RDS: Pretty much.
Interviewer: Um, do you have a cellphone?
RDS: Yea.
Interviewer: Internet, Facebook, that kind of stuff?
RDS: Yea.
Interviewer: Do you have any hobbies?
RDS: Not really.
Interviewer: And who does the cooking and preparing of food?
RDS: Sometimes me and sometimes my mom.
Interviewer: What kind of things do you like to prepare when you get to cook food?
RDS: What everybody wants.
Interviewer: Do you enjoy cooking?
RDS: Sometimes.
Interviewer: Sometimes? What do you enjoy cooking?
RDS: Anything.

Interviewer: Anything? Do you have a favorite food that you like to make?
RDS: Not really.
Interviewer: And did you get, did you see any physical activity messages on the Facebook site?
RDS: Yea.
Interviewer: Do you remember what they were?
RDS: No.
Interviewer: Did you ever click on any of the links that were included?
RDS: Sometimes.
Interviewer: How often do you think that you clicked on the links?
RDS: Like once.
Interviewer: Once? Do you know what the link took you to? Was it a video or a website?
RDS: Sometimes it was a website.
Interviewer: Ok. Do you remember what website at all or what the message was about?
RDS: No.
Interviewer: No? Was, um, exercise something that you thought about a lot during pregnancy?
RDS: Not really.
Interviewer: What about healthy eating or what you are eating?
RDS: A little bit.
Interviewer: A little bit? What kinds of things did you think about?
RDS: What I should eat and what I shouldn't eat.
Interviewer: And could you tell me some of the things that you thought were good to eat and not good to eat?
RDS: Um, hm, Fries.
Interviewer: Fried foods? To eat less of those?
RDS: Hm-Hm.
Interviewer: And what other things did you think about?
RDS: How to eat goods, good food.
Interviewer: What's good food?
RDS: Healthy. Basically.
Interviewer: What foods would you categorize as healthy?
RDS: I don't know. More vegetables I guess.
Interviewer: Ok. And are those things that you were able to do? And what sorts of vegetables did you eat more of?
RDS: Um, I don't know. I really don't know.
Interviewer: Do you have a favorite fruit or vegetable?
RDS: Hm-hm (like no).
Interviewer: Do you have dislike fruits or vegetables?
RDS: No.
Interviewer: Um let's see, how did you access the Facebook site when you were using it? On a computer or cellphone or both?
RDS: My phone.
Interviewer: Your phone? And is it your phone or do you share a phone?
RDS: It is my phone.

Interviewer: Ok. And, when you receive messages did it give you like a, like a notification that a message posted?
RDS: Hm-hm.
Interviewer: And, did any of the messages motivate you to change your behavior? Like exercise or diet?
RDS: Not really.
Interviewer: Ok. What do you think would be a good motivator that we could add to the Facebook page? That would make people want to make changes?
RDS: I don't know.
Interviewer: What motivates you?
RDS: Nothing really.
Interviewer: Nothing? I don't believe that.
RDS: Nothing.
Interviewer: Nothing? Do you have favorite TV shows or anything like that that you like to watch?
RDS: Hm-hm (as in no).
Interviewer: Did any of the messages make you feel positive or negative about food?
RDS: Not really.
Interviewer: And, so I know that you joined the Facebook group for the study, but did you join any other pregnancy groups or phone apps or anything like that?
RDS: No.
Interviewer: No? Um, and I know that I asked this before, but just in case you didn't think of it before. What about like searching on any websites or anything for certain pregnancy information?
RDS: Hm-hm (no).
Interviewer: No? Do you have any friends that you talked about your experience with at all?
RDS: No.
Interviewer: Ok. And so you said that our doctor was a good source of information for you, and I was just wondering if you could tell me what information she gave you that you thought was like most helpful.
RDS: I don't know. Um, basically…I don't know.
Interviewer: Information about like what to expect during pregnancy?
RDS: Basically.
Interviewer: Have they talked to you at all about nutrition? Do you remember what kinds of things that they talked about?
RDS: Hm-hm (no).
Interviewer: And from your healthcare provider, do you think that it would be useful if they sent messages like we did on the Facebook site so what it was coming from your doctor rather from than, I guess me, a random person?
RDS: Not really.
Interviewer: No? Doesn't matter?
RDS: No.
Interviewer: Do you think that text messages and Facebook pages are a good way to communicate with teens?
RDS: A little bit.
Interviewer: Do you know of anything that might be better than those options?

RDS: Hm-hm (no).
Interviewer: And have you heard of any like cell phone apps or any other programs online that might be good for pregnancy education?
RDS: Hm-hm (no).
Interviewer: Ok. So, is there anything else that you thought of that could answer one of the questions we talked about that you thought of after the fact?
RDS: No.
Interviewer: No? Um, any other thoughts or ideas about the Facebook site?
RDS: Hm-hm (no).
Interviewer: And just one more question. Overall, during your pregnancy, how have you felt like emotionally?
RDS: Been ok.
Interviewer: Been ok? Have you ever felt sad or really happy or?
RDS: No
Interviewers: Just pretty even feel?
RDS: Hm-Hm.

RDS034

INTERVIEWER: Hi
RDS034: Hi.
INTERVIEWER: How are you?
RDS034: Fine.
INTERVIEWER: I saw the picture of [name redacted] on Facebook. She's so gorgeous.
RDS034: Thank you.
INTERVIEWER: You're welcome. Congratulations.
RDS034: Thank you.
INTERVIEWER: Um, so, I just want to make sure that you got your, um, gift card for doing the interview from [name redacted].
RDS034: Hm-hm.
INTERVIEWER: Ok. Perfect. So, then, then you are all set. Um, so then, hopefully this won't take like more than like 30 or 40 minutes.
RDS034: Ok.
INTERVIEWER: And then you'll be free to relax, ok? So, um, the goal of the interview is to ask you about, um, where you have been eating and what things that you've changed since you became pregnant for food
RDS034: Hm-hm.
INTERVIEWER: And also for physical activity, so if you changed your activity levels, if you started or stopped any sports because of the pregnancy, those sorts of things and then we'll talk about the Facebook group and the text messages and if you thought those were helpful.
RDS034: Ok.
INTERVIEWER: So, um, anything that you say is, there's no right or wrong answer, so feel free to say whatever you think, and don't worry about hurting my feelings. If you thought that some of the messages that we sent were really stupid, I would rather have you tell me that and you know, help us make them better in the future than just say that they were good. Ok?
RDS034: Ok.
INTERVIEWER: All right. So, the first set of questions is just to kind of talk to you about how things are going and what your goals for nutrition and exercise were during pregnancy. So, the first question is, um, how, how would you say that your pregnancy went from start to finish?
RDS034: (didn't record well)
INTERVIEWER: Just cause of the size of your belly?
RDS034: Hm-hm.
INTERVIEWER: Um, did you have any other sorts of experiences with food or exercise that made it easier or difficult?
RDS034: Um, (didn't record) was easier.
INTERVIEWER: Ok. Um, was that something that you just started doing on your own or did someone tell you about that?
RDS034: Someone told me about this.
INTERVIEWER: Um, would you mind tell me who?
RDS034: Um, one of good friends.
INTERVIEWER: Ok, cool. Um, and how often were you, doing those to see a benefit?

RDS034: Um, I was looking at them about six times a day. (I don't know if this is correct)
INTERVIEWER: Ok, and how many did you do each time that you did them?
RDS034: 10.
INTERVIEWER: Ok. Um, and then squats, what specifically did they help with?
RDS034:
INTERVIEWER: Ok. And were you doing that for a lot of your pregnancy or did you start more recently?
RDS034: I started recently.
INTERVIEWER: How, how recently would you say?
RDS034: Like, (don't know)
INTERVIEWER: Ok, awesome. Um, so the next question is what do you think that the best part of your pregnancy was?
RDS034: Um, (I could not get it)
INTERVIEWER: Was that exciting?
RDS034: Hm-hm.
INTERVIEWER: Did you ever make you think
RDS034: (something)
INTERVIEWER: Oh, yea? That's cool.
RDS034: Yea.
INTERVIEWER: Um, when you felt the baby move, or her heart beat—what sort of things did that make you think about?

RDS037

Interviewer:uh the interview takes about 45 minutes to an hour to finish
RDS037:ok
Interviewer:So, is that, does that work in your schedule?
RDS037: I think so
Interviewer: ok, awesome. Well I'll try to go fast so that I can get you all done. I'm sorry that I never got to meet you. Um, it never worked out with my schedule to be up there when you were in the clinic. But um I work with [name redacted] and then I'm based out of [location redacted] so I just visit like once a week
RDS037:Um, ok
Interviewer: Um, but I just wanted to talk to you about what you thought of the, the messages you received and um learn a little bit about like what you did for eating and exercise during pregnancy and if you found any really good resources that you used online or any good people to talk to about your own pregnancy experiences and I um am going to tape the session just so that I don't miss anything that you say and then we go back and we type out everything that you said and everything that I said and then we remove your name so it's all confidential and only our research team will be able to see the transcripts from the interview. Does that sound ok?
RDS037:yes
Interviewer: ok. Um so I just have a few quick questions to start the interview. They're really um kind of broad and general so the first question is um, just to tell me a little bit about how your pregnancy went.
RDS037:um it went well. I had like, I had started walking and stuff to help me exercise
Interviewer: And what would you say that the best part of your pregnancy was.
RDS037:um me being alone to sleep
Interviewer: uh did you sleep more while you were pregnant than before?
RDS037:yes
Interviewer: and, what hopes and dreams do you have for your baby
RDS037:um well first to grow up and be a successful person
Interviewer:um and so, you chose to do the, the text message part of the study and if you remember there was also a facebook option. And I was just wondering if you could tell me why you chose the text messaging instead of facebook or both.
RDS037:um because I'm better with text messages. Because I text a lot of people
Interviewer:ok, and but do you have a facebook account?
RDS037:yea, but I don't really get on it like that
Interviewer:ok so for the, the messages that you received, um, how often would you say that you checked those
RDS037:I checked them daily
Interviewer:and were there any messages that really stood out to you
RDS037:the exercise ones
Interviewer:do you remember any favorite messages?
RDS037:huh?
Interviewer: um, I'm not sure if you heard me. Did you um have any favorite messages that you can think of.
RDS037:um about the um, the um foods you eat during pregnancy

Interviewer:do you remember what those ones were?
RDS037:when they said what was your favorite food um during pregnancy
Interviewer:oh yeah, it was a poll question, where you could say a, b, c, d or e.
RDS037:yes
Interviewer: and um, for the exercise ones you said that you liked those too. Did you ever um click on any of the links or watch any of the videos?
RDS037:no cause' my phone won't let me do that
Interviewer:oh ok, and did your cell phone allow you to see anything that was like a picture message, or do you only get text, text messages?
RDS037:yea,it didn't let me see the pictures
Interviewer:oh ok, that's good. Um, in, while you were pregnant, did you um, change how you were eating or exercising at all because you heard that something would be good for your baby?
RDS037:yes
Interviewer: can you tell me more about that?
RDS037:I ate like foods that I normally don't eat, I exercised more
Interviewer: What foods did you eat that you didn't normally eat?
RDS037:Like different vegetables
Interviewer: were any of the vegetable ones any that you never tried before
RDS037:no, they're ones that I normally don't eat because I really don't like them
Interviewer: oh, do you have any specific examples, like broccoli or peas or anything like that
RDS037:um, yea
Interviewer: and um, what sort of, what other things did you change about your diet?
RDS037:um I, I stopped eating like, less fats
Interviewer: and was there any foods that you started to crave or started to dislike
RDS037:yea, I stopped liking chicken
Interviewer: can you say that one more time?
RDS037:I said I stopped liking chicken
Interviewer: oh chicken. Anything else that you can remember?
RDS037:no
Interviewer: what about um, drinks? Did you change the things that you drank at all?
RDS037:um, I just, kept drinking caffeine, and I drank more water
Interviewer: and, was that for a specific reason? or is it just that you were thirsty?
RDS037:um, because I wanted my baby to be healthy
Interviewer: and was that something that you learned from the messages that you got, or did someone tell you that that was good for the baby.
RDS037: yes, my um, doctor
Interviewer: what, how much did they tell you was good to drink?
RDS037: like 8 glasses of water a day
Interviewer: and from the messages that you received, were any of them messages that you, that you didn't like or didn't think were helpful
RDS037:no
Interviewer:and you said that some of them made you um, exercise more and change what you ate, so, um ,how did those motivate you to make those changes?
RDS037:um, well I really haven't use them

Interviewer:the messages, you didn't use them to change any of your behaviors?
RDS037:no
Interviewer: so, um, what was it that happened during your pregnancy that motivated you to make those changes
RDS037:um, talking to people, that I know.
Interviewer: Talking to people that you know?
RDS037:Yea, that were pregnant
Interviewer:ok, so are those um, people that are in a club or a group that you are in
RDS037:no they go to my school
Interviewer: ok, are you in the young mothers program?
RDS037:yes
Interviewer:ok, so they were girls from young mothers?
RDS037:yes
Interviewer: was that a helpful program for finding like support and friendship?
RDS037:yes
Interviewer:that's good, it sounds like a really nice program. Um, and I was just curious, what have you heard about weight gain during pregnancy?
RDS037:um, that um, you were supposed to weigh like, you're only supposed to gain like 20-30 pounds or something
Interviewer: and is that something that you thought a lot about during your pregnancy?
RDS037:yes
Interviewer:and how, how did that go for gaining weight? I know sometimes it can be really hard to gain weight and other people don't have trouble gaining, so what was your experience?
RDS037:um, I didn't gain that much weight like I thought I would
Interviewer: Did that worry you at all
RDS037:no
Interviewer: um, did anything that you read or saw during your pregnancy make you feel more positive or negative about the foods that you were eating, or how much you were exercising
RDS037:yes
Interviewer:can you tell me more about those things that you found?
RDS037:yea when they um said that exercising can help give you a easy labor
Interviewer:and where did you hear about that?
RDS037:at my school
Interviewer: and what kinds of exercising were you doing? Cause you said that you were exercising and I think that you did say walking
RDS037:yea, walking. I walked like 40 minutes
Interviewer:every day?
RDS037:like twice a week
Interviewer: oh that's awesome. Um, were there any other types of activities that you learned about or tried because you learned about those in your program
RDS037:um no
Interviewer: have you ever tried, or did ever you try yoga, or um, stretching or anything like that?
RDS037:no

Interviewer: and, whenever you have a question, had a question about health during pregnancy whether if it was for you or the baby, where did you go to ask those questions?
RDS037:To my midwife
Interviewer: To your midwife, did you ever use the internet?
RDS037:no
Interviewer: and have you ever signed up for any sort of email or texting programs other than this study?
RDS037:no
Interviewer: and one last question about the internet, did you ever look on any websites that were about pregnancy?
RDS037:yea, I'm, it's called I'm expecting
Interviewer: I'm expecting?
RDS037:yea
Interviewer: any other websites that you can think of that you might have visited?
RDS037:baby bump
Interviewer: baby bump. And when you searched for those, did you do that on your phone or a computer
RDS037: my phone
Interviewer: ok and did you ever share the information that you found on those websites with your friends and young mothers
RDS037: yes
Interviewer: um, do you remember what kinds of things you shared?
RDS037:about the exercising and the food you eat
Interviewer: um, and based on your experiences with this pregnancy, what person or place gave you the best information about diet and exercise
RDS037:um, your study
Interviewer:our study? Even more than the midwives?
RDS037:yes
Interviewer:ok, and for the messages that we sent to you, we were sending about 6 a week, does that seem like it was too many messages or do you think fewer would have been better?
RDS037:huh?
Interviewer:um, so we sent you about 6 messages a week through text messaging was that too mnay messages or not enough messages or just the right amount.
RDS037:it was just the right amount
Interviewer:ok and do you think that it would be helpful if your health care provider would send messages like that
RDS037:yes
Interviewer:and, from what you remember, what kind of information was most helpful to you
RDS037:um, the um, whole exercise thing
Interviewer:did you like the pictures that said, had like the 7 tips about exercising during pregnancy?
RDS037:yea

Interviewer:and of all the types of messages, so some were short, some were a little bit longer, some were questions, some were pictures, some had video links, which kind of message was easiest for you or did you like the most
RDS037:the pictures
Interviewer:the pictures? Ok. Um, and so you said that you didn't like chicken as much while you were pregnant, what about um fried foods, did that change at all when you were pregnant
RDS037:um, I really didn't eat that many fried foods cause, I felt like nauseous
Interviewer:and what about foods with salt or sugar, did you like those more or less?
RDS037:more
Interviewer:more, so did you like chips and candy, stuff like that
RDS037:yea
Interviewer:ok, and what about dairy food, did that bother you at all?
RDS037:no, I love dairy
Interviewer:what's your favorite kind of dairy?
RDS037:ice cream
Interviewer:ice cream? Me too haha, and did you have any cravings for non food items like chewing on ice or eating like baking powder or anything like that.
RDS037:no
Interviewer:no, and what about smells, did any smells, did you like them a lot, or you couldn't stand them
RDS037: what'd you say
Interviewer:were there any smells that you liked more or less during your pregnancy than before?
RDS037:um, no
Interviewer:ok, um and did you have any challenges with exercising or um nutrition during your pregnancy that you can think of
RDS037:no
Interviewer:and what things did you do throughout your pregnancy to make sure that your baby was born healthy and happy?
RDS037: um, eating healthy, exercising
Interviewer: um,and can you define for me, what eating healthy means
RDS037:it means eating more fruits and vegetables
Interviewer: and do you have a favorite fruit and vegetable
RDS037: yea, my favorite fruit is oranges and my favorite vegetable is corn.
Interviewer: and so were there ever days where you thought you should've eaten more fruits and vegetables and something motivated you to choose those instead of something else, like candy?
RDS037:um no
Interviewer:no, did you feel like it was pretty easy to choose healthy foods and exercise regularly?
RDS037:yes
Interviewer:and, did you have a, a good support network of friends that you could hang out with and talk to about your pregnancy?
RDS037:yes
Interviewer:did they every time they go on walks with you?

RDS037:um no
Interviewer:and when you, the meals that you eat did you make those, or did someone else make those for you.
RDS037:what'd you say?
Interviewer:The food that you eat, did you make the food that you ate when you were pregnant, or did someone else make it for you.
RDS037:your mom
Interviewer:your mom? and does your mom do the shopping to.
RDS037:yes
Interviewer:did you ever go with her, and pick out foods?
RDS037:yes
Interviewer:what kinds of things did you, um help pick out?
RDS037:the fruit
Interviewer:the fruit? And was that something that you and your mom always did together, or did you start doing that after you were pregnant.
RDS037:we always did that together
Interviewer:oh, that's fun. Um, let's see. And, when you became pregnant, did you and your mom change how you made food, like so, if before you maybe like had fried French fries, but then instead you like baked them, that's like one example.
RDS037: yea
Interviewer:what kinds of preparation methods did you change?
RDS037: like, we um, used less salt
Interviewer:less salt? and it sounds like your mom has been really helpful so, did she every give you any advice about having a healthy pregnancy from her own experiences?
RDS037:yes
Interviewer:what kinds of things did she tell you about?
RDS037:um, to stay away from junk food
Interviewer: um, and, did you try any of the ideas in the text messages about eating fruits or vegetables
RDS037:um, no
Interviewer: is there anything that we could have done differently that would have made it easier or made you want to try things that we put in there.
RDS037:hmm. Yea like the more multiple choice.
Interviewer:more multiple choice?
RDS037:yea
Interviewer:and, I think at one point there was a challenge that you could've participated in, but I don't recall that you participated, so if we had like a challenge where you could win a prize is that something that you would enjoy doing or is that too much work
RDS037:yes
Interviewer:what do you mean yea?
RDS037:yea I would enjoy doing it
Interviewer: ok. um, is there anything else that you can think of, about your pregnancy in relation to eating and exercise that we didn't talk about that you think might be helpful to me?
RDS037:no
Interviewer: no, ok, um well I think I asked all my questions

RDS042

INTERVIEWER: Um, I usually start by asking how old everyone is.

RDS042: 17

INTERVIEWER: Okay, and, um, how tall are you?

RDS042: 5’ 4”

INTERVIEWER: Okay, and how much did you weigh today at your appointment?

RDS042: Um, 185.

INTERVIEWER: Okay. And, um, how’s your pregnancy been going since I never got to talk to you in the beginning?

RDS042: It, it was horrible at the beginning.

INTERVIEWER: Horrible?

RDS042: And then it was just like mellow. And then now it’s just like draggin.

INTERVIEWER: Mm-hm.

RDS042: It’s taking forever now.

INTERVIEWER: What do you mean by horrible?

RDS042: Not, just because I was like throwing up a lot.

INTERVIEWER: Oh.

RDS042: And like I couldn’t hold food down and I just, I’m not a throw up person so.

INTERVIEWER: Yeah. It’s not pleasant.

RDS042: So it was just like, oh god, I need to just, this part need to be over. And I’m glad it’s over. And then during the middle part, it was everything was just fine and when he started kicking it was fun, now it’s just like okay, fun time is over, it’s time to come out now.

INTERVIEWER: (Laughs) Um, how long did your morning sickness last?

RDS042: For only for the like, like for the first month and half of the second month.

INTERVIEWER: Okay.

RDS042: And then it just stopped.

INTERVIEWER: Oh, that's a pretty long time.

RDS042: But it was certain things still during the whole pregnancy that I, that just wouldn't stay down.

INTERVIEWER: Mm-hm.

RDS042: Like steak-subs.

INTERVIEWER: No.

RDS042: He's just not a big fan at all.

INTERVIEWER: (Laughs)

RDS042: At all.

INTERVIEWER: Um, and were those things that you really liked eating before pregnancy?

RDS042: I've never really, I ate steak-subs, and it was like, I could keep them down and it was fine and everything, but once I got pregnant, mm-mm. Steak-sub it just out of the question, like, and like certain juices I can't drink, like. I like to drink cranberry juice, only cause I like the aftertaste.

INTERVIEWER: Mm-hm.

RDS042: But I can't drink cranberry juice. The only juice like is apple juice and orange juice. Like any other thing won't stay down. And water.

INTERVIEWER: What was the cranberry juice doing? Just, making you feel sick?

RDS042: It just, like I would just puke it back up. It would just.

INTERVIEWER: Oh, I see.

RDS042: It just wouldn't. Oh no I don't want that. That's how he would be, just like, nope. I don't, we not keeping that.

INTERVIEWER: (Laughs) Get it out of here. Um, so what would you say has been the best part of pregnancy?

RDS042: Um, ultrasounds.

INTERVIEWER: The ultrasounds?

RDS042: Yeah. I like looking in there. I wish I could get more, but, they don't give me no more unless I'm overdue.

INTERVIEWER: Mm-hm.

RDS042: But I like ultrasounds because he like to show out. He moving all over the place, and…

INTERVIEWER: Oh cool.

RDS042: It was just like, and then it was my first time seeing like an ultrasound, like being done. And it was fun. I like that.

INTERVIEWER: Did they print you a little picture each time?

RDS042: Yeah, I have a lot of them. And my little brother will…he always takes them from me.

INTERVIEWER: Oh no. (Laughs)

RDS042: He's like, this baby looks like me. I don't, okay.

INTERVIEWER: (Laughs) He must be really excited.

RDS042: Mm-hm. He got nephews and nieces already. But he just like, I'm gonna quit school to babysit the baby. Like, what you seven. How you gonna quit school, like? (Laughs) Get out of here.

INTERVIEWER: (Laughs) Aw, that's cute. Um, so the best part. What would you say is the worst part?

RDS042: This part. Right here.

INTERVIEWER: Waiting?

RDS042: Yes, like I got two weeks left and it's just like taking forever for [date redacted] to get here, like, any, I'm looking back at all the months like dang, I was just eight months. I was just seven months. And now is like, I'm nine months and still no baby.

INTERVIEWER: (Laughs)

RDS042: Like. It's taking forever. It's dragging. It's taking so long. Like, I just look at, I have a app on my phone, and it like count down days, like how many days you have left and stuff

like that. And I'm just like, (sigh) 18 days. 17 days. Oh my god, I need to be one day already. Like, it's taking so long.

INTERVIEWER: Do you think he'll be right on time? Or do you think he'll be early?

RDS042: I think he gonna be late.

INTERVIEWER: You do?

RDS042: Yeah. Cause all these people just. You know, just having all these contractions and all this, everything is have no, and he just relaxed, like, listen, not yet. I'm just like, please.

INTERVIEWER: (Laughs)

RDS042: Just do anything. I just wanna have contractions cause I'm a big crybaby, and I just want the worst part to be over. Like, and he's so heavy, and it's just like, oh god, it's just too much.

INTERVIEWER: (Laughs) How's your energy level been?

RDS042: Um, on hot days, like when it's warm outside, I talk a walk or something like that. Like my feet been getting a little swollen. So it kinda hurt a little to walk, but I still try to walk like take my little brother and sister for a bike ride or walk up to the store. Stuff like that, but I do get tired.

INTERVIEWER: Yeah.

RDS042: Um, most of the time I just sit around.

INTERVIEWER: (Laughs)

RDS042: And watch TV. But I do try to take walks like when I go to school. They got like the treadmill. And I walk on that for a whole period, and stuff like that.

INTERVIEWER: Are you on the young mothers' group?

RDS042: Mm-hm.

INTERVIEWER: Okay, that's nice. Sounds like they're really good at giving time to get some activity in.

RDS042: Mm-hm.

INTERVIEWER: Do you feel like the group of girls and young mothers is like a nice supportive group that you can talk to about things?

RDS042: They're weird.

INTERVIEWER: They're weird?

RDS042: Yeah, they just, they talk about like it's only a few girls that's like really … time. So it's just like us that talk about oh my god I just can't wait to get this over with. I'm scared, I'm nervous, and stuff like that. And other girl just like, well, we're not even there yet, so.

INTERVIEWER: Yeah, just going with the flow.

RDS042: It's just, it's like three of us. And my due date is [date redacted], one is [date redacted], and the other one is like [date redacted].

INTERVIEWER: Mm-hm.

RDS042: So it's just, talk about it, and they always ask me, am I scared or something. No I'm not scared, I'm just ready to get it over with. So, I mean it's a good place to any-like my teachers.

INTERVIEWER: Mm-hm.

RDS042: And that's how I learned about new bing.

INTERVIEWER: What's that?

RDS042: New bing where you get um, is sort of like an epidural, but it makes you fall asleep during a contractions and you wake right back up.

INTERVIEWER: Oh interesting.

RDS042: But it's not, more, it's not as effective as an epidural. So.

INTERVIEWER: Mm-hm.

RDS042: Like you, they teach you a lot different stuff in there.

INTERVIEWER: Are you planning on trying that or an epidural?

RDS042: No, I'm just going to get an epidural cause like it seems kind of weird to fall asleep during the contractions.

INTERVIEWER: It does seem weird.

RDS042: And then you wake up out of nowhere. Like, it just, I just want to stick to normal stuff that I know about already.

INTERVIEWER: Mm-hm.

RDS042: And I didn't, they taught me what epidural was because I was thinking the whole time, like even before I got pregnant, oh epidural like, you don't have no pain during pushing. That's not the case. I did not know it was just like to help your contractions. I thought it was like to ease the pain from everything, like every part of pregnancy. I'm just like aw man. I didn't not fool my own self.

INTERVIEWER: (Laughs)

RDS042: But I just, I just want it to be over.

INTERVIEWER: Oh you're close.

RDS042: I know.

INTERVIEWER: Really close.

RDS042: That means I'm going to have contractions sometime soon.

INTERVIEWER: Yeah?

RDS042: Yeah, I was thinking like, I wasn't going to school for like the week before my, um due date. But, it become late, and I wouldn't make no sense. So I would just go up and tell my due date, and then if it don't come, then I start going. And I wait until then.

INTERVIEWER: (Laughs)

RDS042: And hopefully they don't put me on bed rest or something like that.

INTERVIEWER: Everything's been going okay?

RDS042: Mm-hm.

INTERVIEWER: So you don't expect that?

RDS042: No.

INTERVIEWER: Okay, that's good. Um, so, um, I always ask everyone this, and um, so, it seems like a weird question, but some people like to imagine the future, so, um, what-what do you sort of imagine the future being like with your little boy and um…

RDS042: I don't know, it's gonna be fun cause I'm gonna do everything for him. I'm gonna be weird and everything. Like we gonna just, imma always play dress up with him. And I want to have long hair so I can always like put some twisties or some braids or something

in it. And I just, it's just gonna be fun. And I can take him everywhere and show him to everybody and it's gonna be mine.

INTERVIEWER: Mm-hm.

RDS042: It can't tell me what to do.

INTERVIEWER: (Laughs)

RDS042: So it just-I just-It's a whole bunch of-like I'm excited and then I'm nervous. It's just like ahhhhh dang, and then it's like, oh yeah I'm gonna have so much fun. And then I be thinking about crying and changing diapers, cause I just hate the smell of poop, like, it just gets to me, I got weak stuff.

INTERVIEWER: (Laughs)

RDS042: And I don't like little baby boogers, I just don't, it just, Oh Lord. I taught my little cousin, one years old, how to pick her own nose cause listen, I'm not gonna go get your boogers when you big enough to do it yourself.

INTERVIEWER: (Laughs)

RDS042: I told her how to get them out. So, hopefully he learns how to get them out himself. But I can get them out until he learns. And I don't really like the snot sucker thingy.

INTERVIEWER: Oh. Mm-hm.

RDS042: I know it's good for them. I just hate the sound of it. It's just weird. But I think I'm going to have a lot of fun. Is this-is this going to be so cool. Like a whole little bit. Like it's not gonna be like, oh can I hold your baby? It's like I can pick up a baby anytime I want.

INTERVIEWER: Mm-hm.

RDS042: And I can keep him in my arms all day. And do this to him, and do that with him. If I don't want you to see him, then you can't have him. And it's gonna be fun. I think so.

INTERVIEWER: Does it seem like you have good support from friends and family?

RDS042: Yeah. My mom went overboard. Like she bought him a pair of sneakers he can't wear until he about one.

INTERVIEWER: (Laughs)

RDS042: I'm like, Mom. Really? And they like, my-her and my aunt competing, cause my mom says she's going to buy the car seat and stroller, but my aunt got it first, so my mom start buying you know all these crazy different kinds of sneakers and diapers and wipes

and, I'm just like, can I do something, like what do I have a say so in this? But they just got everything, mostly everything. I don't think he really needs anymore diapers. And it's just like, my baby's father, he bought some stuff, but it's his first kid, so he don't know what he doing.

INTERVIEWER: Mm-hm.

RDS042: And he bought some preemie clothes.

INTERVIEWER: (Laughs) Oh no.

RDS042: I'm like, we're not gonna have a preemie, so. He like, I thought that was for small babies. Okay, not gonna be that small. I showed him a difference between newborn and preemie. He like, oh. I was like, you should just take me with you. But he wanted to do it by himself, so he got the wrong size. And he just like, well he can wear it when he first comes out. Alright, we gonna try, cause they really, I never noticed how small preemie clothes is until I seen it, compared to the other clothes.

INTERVIEWER: Mm-hm

RDS042: They super small. But for the most part, yeah he support me a lot. At first I didn't tell him until I was three months. I was scared. I told my father when I first found out. And then I didn't tell my mom and aunt until I was three months. Well, they told me, so it was just like, yeah we know so.

INTERVIEWER: (Laughs)

RDS042: And at first my mom wasn't too excited about it. And then she just got used to the fact that you're going to be a grandma, so. Get over it. And she did, so. It got better.

INTERVIEWER: That's good. Um, so I know one thing you mentioned was the, some of the teachers and mothers have give you some really helpful advice about epidurals and um, what other kinds of things have they taught you about?

RDS042: Basically about, mostly about labor since I'm so close to, they already teach me about um, like how to take care of yourself after you have the baby and stuff like that. Um, they taught me if I need, have a C-section, how to take care of the part right there.

INTERVIEWER: Mm-hm

RDS042: And how to, basically, I knew how to change diapers and make bottles and breast feed and stuff like that. I got a lot of brothers and sisters younger than me so. It was just like either learn it or don't. I like babies so. And they teach me how to make bottles, change diapers, like how to keep him safe from infections, even though like most of the time they gonna get a cold or ear infection and stuff like that, cause it's natural I guess.

INTERVIEWER: Mm-hm

RDS042: And they teach me how to prevent it and let it happen on its own. And they just, they just go through a lot of stuff about if you tear when you go in labor, and how to try not to tear and stuff like that. Just mostly about how to take care of a baby.

INTERVIEWER: Mm-hm

RDS042: And how to stay healthy and stuff like that. Excuse me (yawn).

INTERVIEWER: That's okay.

RDS042: Like they, they told me not to overshop, cause if you get a whole bunch of baby clothes like they say my mom she didn't get newborn clothes. She got three to six months. Because she said they grow fast. I got a couple newborn outfits, and um, 0-3 months, but they, my teacher said they grow fast. She taught me about the diaper thing. She told me to buy one case of uh newborn diapers because once the bellybutton thing fall off, it'll be easier to put the bigger diaper on cause they don't have to go um like under rear or nothing like that.

INTERVIEWER: Oh yeah.

RDS042: He gonna get bigger and so its just like yeah. Yeah, how to wash him, and when to feed him, like um, cereals. With the bottle, when to feed him at, and just how to take care of my baby mostly.

INTERVIEWER: Yeah. Um, have you received any um advice from those teachers or your midwife about foods that are good to eat during pregnancy?

RDS042: Um, my-I got one parent and teacher. So it's like she know everything, she got kids and she's been a parent-teacher for some time and she just basically tell me just to eat healthy like it's okay to eat like um, like chicken and greasy food sometimes, just not like all the time, it's okay. Just eat like, she tell me in the morning time when I come eat a banana or an apple, or orange, or something like that, like just eat mostly healthy. When we go to the pop machine, she tells me not to get Pepsi because, I think she said it causes heart burn or something like that. And she just basically she could, she sell water for 50 cents, so. She prefers cause the pop machine is 1.50.

INTERVIEWER: Oh yeah. So do you

RDS042: So she sell water for 50 cent. And it's just like dang which one do I really want?

INTERVIEWER: (Laughs)

RDS042: So, yeah.

INTERVIEWER: That always helps in decision-making.

(Both laugh)

INTERVIEWER: Um, have you changed how you've been eating since, from you know when you found out you were pregnant? And throughout?

RDS042: Yeah. Like certain things I don't like want to look at no more. Like, I can't, I can eat chicken, but I can't when it's fried or I would just puke like crazy.

INTERVIEWER: Is it the smell?

RDS042: The smell of it, yeah. And I don't really like, I mostly like seafood now. Like shrimp and crab legs and.

INTERVIEWER: Oh nice.

RDS042: And I didn't not used to eat that before. And I'm just like, what in the world? I really be cra-, I crave orange juice. Long time, cause I used to drink apple juice. That was my thing, and just like okay. No, and cranberry juice, I used to always drink those because I like the taste. Now it's just orange juice, orange juice, orange juice. And it gets me heartburn though. But I just gotta have some orange juice.

INTERVIEWER: (Laughs)

RDS042: Man, like even if I see it, my mouth would be like watering. I gotta get some orange juice, some kind of way or it not gonna work out.

INTERVIEWER: Mm-hm. Are there any foods that you've heard that are really good for you or the baby?

RDS042:, Um not- I probably did, but I just don't remember. But I've been hearing really no food, like I heard you could drink milk to give the baby calcium and stuff like that, so they don't take it from your teeth. Cause that's why my teeth would hurt so bad. And they can't really do nothing because I'm pregnant, I'm so far pregnant and it's just like I can't even do anything. I just gotta wait, and they hurt so bad, but I drank a lot of milk. They told me that was good. Foods, really, it's like, I mean he just said that bananas are good I guess, but not really no. Food, like a full course meal food, that you could eat to make you healthy and stuff like that.

INTERVIEWER: What about, um foods that can be, that aren't safe or could be less good?

RDS042: She told me not to eat like a lot of spicy food, like um, jerk or um, like Jamaican food, cause it's mostly spicy.

INTERVIEWER: Yeah.

RDS042: She told me not to really eat, I could eat it, but just not all the time, and every, like every day, all the time constantly.

INTERVIEWER: Mm-hm

RDS042: Just eat some and then save some. And if I want it later, just don't eat so much at one time.

INTERVIEWER: Mm-hm

RDS042: Um, and like food she said that was hard to digest and chips and stuff like that, don't eat that so often. And I haven't really been. I just when I go to the store I buy orange juice all the time. I spend all my money on orange juice so.

INTERVIEWER: (Laughs) Could you tell me what a maybe a typical day looks like for what you eat, you know, throughout the day?

RDS042: Um, on school days I eat like either, they give me a breakfast bagel, and a sausage, or I could pick between that and cereal. Like two little small things of cereal.

INTERVIEWER: Mm-hm

RDS042: With a fruit, and then for lunch if I'm at school, they always got pizza or a salad, and I could choose between those and they got like a small carton of juice, some milk, and like they always have a fruit up there somewhere. And sometimes they have like broccoli, but they don't use salt or nothing like that so. And when I get home, I just whatever my mom cooks, like rice. She mostly cooks rice, like it's always, I don't know why, but it's always rice somewhere in the middle everyday, all the time.

INTERVIEWER: (Laughs)

RDS042: And I don't have a problem with that, cause rice makes me full. So it's either rice and chicken, rice and pork chops, or yesterday we had rice and beef stew.

INTERVIEWER: Mm-hm

RDS042: That was good.

INTERVIEWER: It sounds good. Um, are there any foods that you've had that are, I know you're craving orange juice, but any other cravings?

RDS042: I used to crave big macs.

INTERVIEWER: Mm-hm

RDS042: Like, all the time, I would have a crying pully fit, if I couldn't get a big mac. Like I needed one at least everyday. And this was like, it started when I was like three months to seven months.

INTERVIEWER: Mm-hm

RDS042: Like I had to have a big mac and some fries. And if I didn't I'm going to scream and yell until I do. And that was the way it was. I had to have a big mac.

INTERVIEWER: What do you think that means?

RDS042: I do not know. I hope I don't look like no big thing.

(Both laugh)

RDS042: Because I don't know like I never really crave for like McDonald's that much, like it was an everyday thing, I want a big mac, I want a big mac, I want a big mac. Like everyday after school. And my baby father is like don't listen to me, going to be sick, like well, let me find it out for myself. I want a big mac. And it's just like, I had to have one.

INTERVIEWER: And what point in pregnancy was that?

RDS042: From like three months to seven months.

INTERVIEWER: Okay.

RDS042: And then after that it was just like, food is just food. Whatever I got was good.

INTERVIEWER: Mm-hm

RDS042: I would eat a whole lot. I had to stop eating so much, because I do not want to be over 200 pounds. This 185 is scaring me. And I'm just ready for him to be out so I cannot gain no more weight cause I'm, I'm really scared of being over 200 pounds.

INTERVIEWER: Why does that scare you?

RDS042: I don't know. It's like I got a phobia to be over 200 pounds. Like it's just, I just don't want to be fat. I'm scared, like I see people die every day like, oh this lady got diabetes and she this many pounds, or this girl was, my little cousin she weighs as much as her mom and she only eleven. And, my aunt is 30. And she got diabetes, like at eleven? No, I can't. That's just, at eleven I weighed like 98 pounds, but that was cause I'm skinny. Like real skinny.

INTERVIEWER: Mm-hm

RDS042: And I didn't get like weight on me until 8th grade. So it was like, and I always went up and down between 150 and 130.

INTERVIEWER: Mm-hm

RDS042: It was always between there, it was never over. Oh, it was never over 160. And I'm just, I don't want to get fat and die. I'm not saying fat people die all the time.

INTERVIEWER: I know, I know what you mean.

RDS042: It's just like I'm not used to that.

INTERVIEWER: Yeah

RDS042: I'm not used to being big. Like I'm used to no stomach and I just feel like I got to suck it in. I can't.

INTERVIEWER: So how do you feel about the weight that you gained during pregnancy?

RDS042: Like I'm not disappointed about it. I'm glad that my baby is growing and stuff,

INTERVIEWER: Mm-hm

RDS042: But the quicker I could get it off, I would be more happy. It's just scary. I've never been this big ever in my life and it's different for me so. It's kind of scary but I'm happy at the same time.

INTERVIEWER: Did anyone ever say that there's a certain amount of weight that you should gain?

RDS042: I have a baby book and I have an app on my phone that tells me, like, when, like, every doctor's appointment I get I'm supposed to put in what weight I am and if its green that mean okay you gained enough pounds or you gained too less pounds or you didn't gain enough pounds this week so.

INTERVIEWER: Mm-hm

RDS042: It's, they got information that I needed, cause I got the baby book in the beginning, and then I got another one, and then they give me like the folders between the trimesters. And then it tell me in there, and then I got the app. I google everything so.

INTERVIEWER: Um, for weight gain, what sorts of things have you googled?

RDS042: Like, I, sometimes I put in like how much am I supposed to weigh at this many months. Or how much am I supposed to gain in this week, and it really just, like I check a

whole bunch of different sites to make sure, ok if this not right then this gonna be a different answer.

INTERVIEWER: Mm-hm

RDS042: But the doctor is like um, it's a couple of them I went to. I got a, where is it, like, this, um the my chart thing,

INTERVIEWER: Mm-hm

RDS042: It tell you on there too sometimes.

INTERVIEWER: Okay.

RDS042: About how much you're supposed to gain, and how much you're supposed to like eat and stuff like that so.

INTERVIEWER: So do you think that, that you've gained the right amount of weight?

RDS042: Yeah, like, sometimes I think I be gaining too much, but then like, cause in the week I could gain like 12 pounds.

INTERVIEWER: Mm-hm

RDS042: And I'm like, holy crap I just weighed this much last week, like what is going on? And they like no it's okay, this is normal, me and the baby growing and I'm like he just playing games now.

INTERVIEWER: Mm-hm

RDS042: He growing too much.

INTERVIEWER: (Laughs)

RDS042: Like what's going on here. It's been more of through 8months to 9 months that I gained the most weight in a week. So it's just different. I don't know, it's just different.

INTERVIEWER: Have you heard of anything that the amount that you gain like effects it can have on the baby?

RDS042: Um, what do you mean?

INTERVIEWER: Um well you mentioned gestational diabetes, um so that would be something that could affect you.

RDS042: Yeah, I don't have diabetes, but first I was scared to get it during pregnancy because it run in my family like some of my aunts got it, some of my uncle, well I only got one of them, he died. And like, my aunts on my mom's side and my aunts on my dad's side, so it's just like. And my aunts on my dad's side, they is kinda heavy set, and I was just like, worried about, holy crap I'm going to be huge and I'm gonna get diabetes because it runs in my family. But I didn't get it so I was so happy, I was relieved.

INTERVIEWER: That's great.

RDS042: But one thing, I got the um sickle cell trait, and that scare me. Because I didn't know what it was or how I could get it, or but they just say you don't have the like disease, you just got the trait of it, cause my mom has it.

INTERVIEWER: Oh. Okay.

RDS042: So she don't, have the disease either, but she just got the trait. Just go down the line somewhere, somebody got it. And it just, its scary and exciting at the same time. Its different though.

INTERVIEWER: Um, have you had any cravings for nonfood items like ice or powder or?

RDS042: Sometimes I would like wanna see what dirt tastes like.

INTERVIEWER: Okay.

RDS042: Like I would just sitting outside and be like, I should eat some dirt. That's like, it was like a weird thought to me, like, why in the world would I want to eat dirt?

INTERVIEWER: (Laughs) It happens though.

RDS042: And I'm thinking, cause I would yell at my brother like, stay out the dirt, don't play in the dirt, you gonna get your clothes dirty. And then it just be like, I want to eat that so bad, but like, I just couldn't, I'm like, this not me, so, I'm not gonna eat some dirt.

INTERVIEWER: Is it something that you looked up?

RDS042: No, I actually didn't. I just stopped myself from trying to eat some dirt. I just like stay in the house cause I'm not looking at nobody dirt and try to eat it. And sometimes I try to eat like cigarette butt ashes. And I'm like, I will play with it on my hand, and smell it, and want to lick it but that aint good. Not about to do that, like, it just, it's scary.

INTERVIEWER: Did those go away?

RDS042: Yeah, I'm glad they did cause it scared me not, like, it was just the thought out of nowhere, like dang I should eat some of that.

INTERVIEWER: When in pregnancy did you feel like that?

RDS042: Like four months until about seven months is when I started craving the dirt and ashes and it was just like why. Like why in the world. And I be thinking of eating dirt. Never have I ever done this. Not even as a little kid, have I ever wanted to eat dirt. […]oh no. It's like sometimes you can't control it and you don't know what's going on, and that's scary for me like, okay, this ain't, something not right.

INTERVIEWER: Is that something that you asked your midwife about?

RDS042: Yeah. She said its normal to crave nonfood things, but try not to eat it. And that's what I did. I try my hardest to, I think I ate dirt one time.

INTERVIEWER: Did she recommend anything to eat instead when you craved those things?

RDS042: Nope, cause I just told her I would want it and want it, but I just wouldn't eat it. It's just like, no. My teeth gonna get all brown and no I don't want that.

INTERVIEWER: Are there any smells that you have found that you like a lot more than you did before?

RDS042: Detergent, like, before you wash the clothes. Not soap for like washing dishes. That smell is like regular. Like I will wash my clothes like I would put in one load and use a whole little, one of the little um, cases of detergent on my clothes just so I can smell it when it came out the wash, like oh yes this smell is good.

INTERVIEWER: (Laughs)

RDS042: And I would use, keep all the bottles and just take the tops off and set them around my room so it smell like that in the room when I walked in. I just love that smell so much.

INTERVIEWER: And was that something around the same time as the food?

RDS042: That was going on the whole time.

INTERVIEWER: The whole time?

RDS042: Not the first couple months, like not the first two months, but right around three months it was just like oh yeah this detergent, love it.

INTERVIEWER: Um, who usually does the grocery shopping at home?

RDS042: I used to live with my aunt and I would do it cause I have my own like way to do, like what I wanted and how and to get it and stuff like that. But now, my mom really goes grocery shopping. So she do most of the grocery shopping and cooking and stuff like that.

Like I just can't, I can't stand over the stove and cause I been to burnt some food, like cause I need breaks, I can't, if I could sit down in the kitchen chair and have a little long stick […] everything I would, but I just can't stand up over the stove and then, or run back from the sink to the stove to the table to the counter, and it's just, mm-mm.

INTERVIEWER: Yeah.

RDS042: And the grocery store, I like going to the grocery store. But then it just so much walking. You got to get this, and reach over and get that. And nope, I'm not going. You go by yourself. I hope you get the bags.

INTERVIEWER: (Laughs) How much do you think that um you were spending on groceries when you were doing the shopping each week?

RDS042: I would only spent like $200.

INTERVIEWER: Each week? Or each month?

RDS042: I didn't go each week, I just went each month. That would be enough to fill the refrigerator. Just like, and I would get like, I only get on thing of cereal, cause I don't eat cereal at home that much, only on the weekend.

INTERVIEWER: Mm-hm

RDS042: Cause I would be at school most of the time. Most of the days. And it'd be two days out of the week that I would be home. So I would not get a lot of cereal. I would just get like freezer food that I could cook, like, okay if we off of school this week, I know I got some food in the house, but most of the time it's only like, I only eat around dinner time, cause I eat breakfast and lunch at school so.

INTERVIEWER: Mm-hm

RDS042: It would just be like dinner time on the weekends.

INTERVIEWER: Yeah.

RDS042: And when we have off school so.

INTERVIEWER: Um, and so earlier you mentioned that you like to take your brothers and sisters out and go on walks. Um, tell me a little bit about how your activity level was before pregnancy and how it changed.

RDS042: Oh, like, when I started going to East High School that was the beginning of in September, that's around, like I found out right before school started that I was pregnant. And I wanted to be a cheerleader, and I wanted to be on the track team, well I didn't want

to be on track, that's too much work. I wanted to be a cheerleader. I thought about being on the track team though.

INTERVIEWER: (Laughs)

RDS042: I wanted to be a cheerleader. I wanted to do all this homecoming and all this stuff, and like, as maybe next year I'll try again. It was just like, I was like bored with the fact of being a cheerleader like, I'm too tired, I don't want to do that. I'm not going to do that.

INTERVIEWER: So you did do it for a little bit though?

RDS042: Like when I first, I used to, like when I first got pregnant, I still would play kickball and like all the running around sports, I would still do that because my teachers didn't know I was pregnant because I didn't tell them. And I would play kickball and stuff, up until around three months.

INTERVIEWER: Mm-hm

RDS042: And after that, it was quitting time. Like I didn't want to play anything, I was tired, falling asleep in class, like I'm trying to stay up and it just like dosing off and it was hard so. But after those first couple of months, like I wasn't interested in doing anything. Like it was just in my mind, maybe next year.

INTERVIEWER: Mm-hm

RDS042: Or I do it some other time. I just, not right now. I don't feel like it. I don't socialize. The only person I go see is my best friend. And she 11 days behind me.

INTERVIEWER: Oh. You guys are so close.

RDS042: But she the one who told me that I was pregnant. But she didn't notice she was pregnant. And then like maybe she was, I was three months, and she found out when she was six months.

INTERVIEWER: Oh wow. That's a long time.

RDS042: But she, she didn't like, just eat and, I kept asking her like how, you got a daughter, how you didn't know you was pregnant?

INTERVIEWER: (Laughs)

RDS042: And it was just, she just looked the same, her body didn't grow, and I'm like bestie, you look fat. Like don't call me that, don't call me fat. And she would just be so emotional, like stop crying, my best friend is tough as nails. Like, don't nothing offend her, you can't make her mad, she don't cry. And I'm like, okay yeah something not right, you need some meds.

INTERVIEWER: (Laughs)

RDS042: And she went to the doctor and found out she was pregnant. I knew something was wrong, cause you don't cry, you just don't cry.

INTERVIEWER: Mm-hm

RDS042: And that scared me like okay until you go see what's wrong I'm not coming back over. And then she found out that she was pregnant. She used to always tell me like, you come over here and all you do is just sleep. Cause in the summertime we were just party girls. Just like, everything we went to parties, we got tattoos, like, we went to the beach all the time and after that summer was over, after my birthday, [date redacted], all the time I would just sleep, and eat, and just sleep, like, I didn't want to walk nowhere and nothing. And she like yeah you're pregnant. Like don't tell me that. And she was like you are. Nope, I'm not. And then I found out that I was, and like, it was the beginning of September, before school started.

INTERVIEWER: Mm-hm

RDS042: So.

INTERVIEWER: So how's your activity level now?

RDS042: It, it dropped. Done for. No activities. On treadmill, I force myself to walk. Like the other day, it was like, sometimes we get double periods, sometimes we get gym, like, science then gym or science then lab. The other day we had gym, and I'm like I'm gonna go here, I'm gonna walk the whole period. And that's what it gonna be. And then it started getting closer to gym time, and aw man I do not want to walk, like, I just got done walking the hallway.

INTERVIEWER: Mm-hm

RDS042: But I force myself to walk on the treadmill, like, this baby gotta come out. I just don't do nothing anymore, like at all, like, the most I do is walk to the store, or like I walk down from the bus to here. So it just…

INTERVIEWER: So how long is that walk?

RDS042: Not even five minutes. Not even two minutes I don't think, so. It's just like, I don't like walking no more, mostly because my feet is hurting now. Cause they like fat, and I just sit around or I sit on the porch. And my little brother always want to go to the store. He's like please. Come on, lets' go to the store and yeah. I think he more excited…

INTERVIEWER: (Laughs)

RDS042: …about the baby than I am. Cause he's always, you can't touch the baby stuff until he get here. Like he will have a holy fit on anybody who touch the baby stuff, cause it's all of his room.

INTERVIEWER: Uh-huh

RDS042: And he be like, no no you can't come in my room, the baby stuff in there. And I'm like, okay you crazy.

INTERVIEWER: Like, it's my baby.

RDS042: Activity-wise is, like I keep saying I wanna be a cheerleader next year and I want to try out for this thing, and do this next year, but right now, nope, I don't even want to look at a cheerleader.

INTERVIEWER: (Laughs)

RDS042: I don't. I just miss my old body that's all. I've never been this big before.

INTERVIEWER: Are you considered about losing the weight afterwards?

RDS042: No, I already got that planned out. Once my six weeks is up,

INTERVIEWER: Mm-hm

RDS042: jogging, eating, carrots and celery stuff, because mm-mm, I can't. I just can't big. It's no. Once I get back down to like 160, that's fine. That's perfectly fine. But I am 185, I can't be 170 and over. Like that's just no. It's not.

INTERVIEWER: Is there a certain reason that you feel that way?

RDS042: I got a phobia of being fat, like I just can't see myself being fat, like, big ol' face.

INTERVIEWER: You don't look like that!

RDS042: arms, and it's just like I don't, like, I got fat, so it's not that it'll disgust me, it's just like holy freaking crap. This is me, I don't want to ever wear a size, if I got to go over size 12 in pants, I'm going to cry. I just want a body, like

INTERVIEWER: Mm-hm

RDS042: Like this, my legs are huge, my feet are huge, everything is getting so big, and I just want to go back to regular. And like the skinnier girls, like they was like 98 pounds at 17, she like oh my god, I'm so big, and you weigh 101 pounds, what do you mean? Are you just joking right? And she like, no I feel so fat. Girl, are we going to. You weigh 101. Who's bigger

here. And they just be like, you don't even look that big. I feel that big. And I don't, I just never been fat. I like, I was skinny as a stick like forever.

INTERVIEWER: Mm-hm

RDS042: And like 8th grade, that's when I start gaining some weight and like, I just never been over 150. I've been 153, but that was the highest I ever went.

INTERVIEWER: Mm-hm

RDS042: And I just don't want to go over the weight limit. I don't want to go over this weight limit ever again in my life.

INTERVIEWER: That sounds like you have a plan.

RDS042: Yes.

INTERVIEWER: So that's good.

RDS042: I like, I walk though, I walk the whole pregnancy so.

INTERVIEWER: How much do you think you are walking?

RDS042: Like all the time. I walk all the time. I think I've been in the car like 6 times since I've been pregnant. That's it.

INTERVIEWER: So you walk to school?

RDS042: Yeah. No I catch, they don't let you walk to school.

INTERVIEWER: Oh.

RDS042: Like nobody. Like I live right around the corner of school. They call the yellow bus for me to come. I can't cross the street or nothing like that. But like when I was catching the city bus. I would catch the city bus and I would get off like a few blocks before my school and just walk, walk up there. I walk from downtown actually a few times to my school, cause it's right there on the street.

INTERVIEWER: Okay.

RDS042: So I walk from downtown to my school.

INTERVIEWER: Where else do you walk?

RDS042: Mm, sometimes I walk from my mom's house to my aunt's house. And I used to walk from like one end of [name redacted] all the way to the other end because my baby father lived down here, my aunt lived down here.

INTERVIEWER: Mm-hm

RDS042: And

INTERVIEWER: That sounds like a long walk.

RDS042: It's a long walk. At first I used to cry like, I don't want to do this anymore. I quit. Like I'm about to catch the bus, but then I was just like, I might as well finish, I'm already halfway there.

INTERVIEWER: Well, what effect do you think all that walking has on you and your baby?

RDS042: Hopefully, he'll walk out.

INTERVIEWER: (Laughs)

RDS042: I never walk that much in my life. Like ever. I am lazy.

INTERVIEWER: Mm-hm

RDS042: And, I just, I'm like, okay, this got to, they told me walking is good, so I'm going to walk. And I just walk, and walk, and walk for a long time. I used to live with my friend and all she did was walk like cray-crazy. I'm not about to, listen I'm calling break today, I'm not walking nowhere with no, I'm not even walking to the store with you. I'm not about to walk outside. Because all we did was walk every single day like if there's nothing to do, come on let's walk. Okay. Like, I just walk the whole entire time. Like it wasn't day went by that I didn't walk.

INTERVIEWER: Even when you're feeling tired?

RDS042: Yeah, like I always either my brother would want me to take him to the store

INTERVIEWER: Mm-hm

RDS042: Cause he always end up with the money cause he the baby from my mom and my dad, well not my dad, his dad and he is spoiled. And he always want to go to the store and that's my only brother so okay [name redacted]. Just wait a minute. But you said that, okay come on, come on, let's just go to the store.

INTERVIEWER: (Laughs)

RDS042: Or let's just go to the park. Or let's just go ahead. And he's just like you gotta walk faster. I'm like oh my god.

INTERVIEWER: So something else that I wanted to talk about was um, what you thought of the messages that you received on text?

RDS042: Oh, I still got, like they…

INTERVIEWER: It sounds like the facebook thing wasn't working so well?

RDS042: Yeah it was just, I don't know

INTERVIEWER: Did you get anything?

RDS042: I got a friend request but I told you it's kinda like

INTERVIEWER: That was me. I think it had like a big hood on my head.

RDS042: Yeah I think

INTERVIEWER: Was it like red and yellow?

RDS042: Yeah

INTERVIEWER: Yeah that was me. Sorry.

RDS042: And I didn't like I didn't know like okay this is weird. Maybe a lot of creepers on facebook like old guys, weirdos

INTERVIEWER: That is true

RDS042: Just like okay, I don't even know who that is, never met that person in life, so.

INTERVIEWER: Okay, but um, cause I think I have your name in like the facebook group?

RDS042: Yeah, I text the number back to ask them to send it again and I don't think they did it.

INTERVIEWER: I-I feel like I did. But maybe something weird happened.

RDS042: Um, right, I'm probably not sure cause I wasn't getting the messages from the Facebook thing.

INTERVIEWER: Do any of you like have some pictures that we posted that you can look at.

RDS042: From the messages, I be I get this this one all the time

INTERVIEWER: You did get that one?

RDS042: Yeah.

INTERVIEWER: Okay. Did you see this?

RDS042: I don't think I seen that one. I get these though. I have it here and I got, yeah I got these, I got a couple…I got these

INTERVIEWER: Okay, so you saw this

RDS042: Um

INTERVIEWER: So those look familiar too?

RDS042: Yeah, not this one.

INTERVIEWER: Okay.

RDS042: But, like most, yeah these are, I seen these ones. Yeah.

INTERVIEWER: Okay. Awesome. Um, so one thing I thought would be cool to talk about was um, actually I'll let you hold onto these, but you have seen these

RDS042: Thank you

INTERVIEWER: So this is the list of all the text messages that you received

RDS042: Mm-hm

INTERVIEWER: And feel free to just take a, you know, a couple minutes, as long as you want to look through and what I want you to think about is if any of them you remember really well and why you remember them so well. If there was something about it that you know, you thought was cool or…

RDS042: I don't know.

INTERVIEWER: Okay

RDS042: I was really excited about, I don't even know why.

INTERVIEWER: And, I don't know, just in case, this is the list of anything that was on facebook. So if you think it was on facebook, you can look there too. So yeah, take as long as you need.

RDS042: This is, I think I, it was heart burn that could like, that's when I first started getting heart burn and then like the contractions.

INTERVIEWER: Mm-hm

RDS042: And just when I needed it, that text came in, and I'm like okay, and we text back for a minute. Right here this one. This one and this one about heart burn.

INTERVIEWER: I think you had asked a question about heart burn after the Braxton-Hicks maybe?

RDS042: Yeah, I probably did.

INTERVIEWER: So if that was the case, that won't actually show up on the sheet, but I, so that was helpful?

RDS042: Yeah.

INTERVIEWER: Okay.

RDS042: Cause I was scared, like, I'm like, cause sometimes I would be like a throw up feeling. It wouldn't be throw up, it would just

INTERVIEWER: heart burn?

RDS042: Yeah it would just come up a little bit, then it would hurt so bad, and I'm just like this ain't.

INTERVIEWER: Heart burn is serious.

RDS042: Yes, it just hurt so bad. I never knew it would hurt like, okay cause I never really got heart burn. I don't know, I just never got heart burn.

INTERVIEWER: Mm-hm

RDS042: And I'm just like ooh, no maybe that's a good.

INTERVIEWER: (laughs)

RDS042: And my mom was telling me that it was heart burn. I'm just like, this can't, mm-mm.

INTERVIEWER: Were there any messages about nutrition or exercise that really stood out? That maybe tried something new?

RDS042: I used to read the little picture things, that came with the picture, I used to read them all the time. And like give those a try, you should try, or mom you should get this when you go grocery shopping.

INTERVIEWER: (Laughs) Do you remember any specific things that you tried?

RDS042: It was um, a meat one.

INTERVIEWER: A meat one?

RDS042: I don't […] get my phone. It was a recent one that I just, …[area code redacted] number.

INTERVIEWER: Yeah, it's [location redacted].

RDS042: Mm-hm. I got one about yoga. I was doing that, the yoga one.

INTERVIEWER: Did you click on the link?

RDS042: Mm-hm. And I would like put a yoga chart up there. And like, okay, I could do this.

INTERVIEWER: (Laughs)

RDS042: And it comfortable.

INTERVIEWER: Yeah.

RDS042: Um, I read about this one, the cookies one.

INTERVIEWER: Uh-huh

RDS042: I read about that one. I clicked on that link. And it was, I did the yoga thing. Mmm. It was a couple of them that I did. And a couple of them I would just look at, like okay.

INTERVIEWER: Which ones were you just like okay?

RDS042: It was just the ones that tell you about like I could show you.

INTERVIEWER: Were they just boring?

RDS042: Like, the instant oatmeal for breakfast on chilly mornings, its quick pack. Like, it was really like okay, whatever. I already eat oatmeal. I eat oatmeal most of my mornings, so its just like okay. It would never be about yoga that I really be like oh yeah I could do that, cause they do it at my school, so I know I could do this one.

INTERVIEWER: Mm-hm

RDS042: And then the ones about like cookies.

INTERVIEWER: Of course

RDS042: So that wasn't hard to pay attention to.

INTERVIEWER: Um, was there anything new that you learned about nutrition?

RDS042: About heartburn.

INTERVIEWER: About the heartburn?

RDS042: Yeah, and like what I am and not supposed to eat at certain times like that.

INTERVIEWER: Mm-hm

RDS042: And cutting down portions of meals. Instead of three big ones, you should eat like six small ones, I think it said.

INTERVIEWER: Mm-hm

RDS042: Yeah. They taught me that, because I would eat huge meals to fill myself, like I would eat a lot and then I just like where did it go? Now this baby's gonna be huge, cause he eating all my food.

INTERVIEWER: Yeah.

RDS042: I would eat another big one. And the ones I start eating, the six different ones, and the snack in between sometimes, I would get more full just eating bigger meals and stuff like that.

INTERVIEWER: Was there a certain type of message that you liked the most? So for example, just like one short little sentence or a picture?

RDS042: I like the picture ones.

INTERVIEWER: You like the pictures?

RDS042: So it was giving me like, I'm more of a, you got to show me that kind of person. Picture ones. And I like where you can click on the link and they have like a website for it.

INTERVIEWER: Mm-hm

RDS042: I could read more about it. I like those kinds.

INTERVIEWER: Okay, that's helpful. Thank you. Um, how often do you think you clicked on the links?

RDS042: Most of the time. Like if I was, if I'm in a bad mood and I just don't want to hear nothing from nobody.

INTERVIEWER: That makes sense.

RDS042: But I would go back, and oh I didn't see this one before. I didn't go on this one. I would just click, most of the time I would click the link, read more about it and know what it was or how it worked something like that.

INTERVIEWER: Mm-hm

RDS042: So most of the time I would read the links. I just like reading about stuff that I don't, like this is my first kid so it's just like I want to know everything

INTERVIEWER: Yeah

RDS042: So I would just click on most of them and

INTERVIEWER: Um, so I just have a couple more questions. I know that you mentioned that you had a cell phone app that you were using and maybe some other things that you've signed up for, or you go to the websites. Can you tell me a little bit more about those and what they're called?

RDS042: I got a app that's called Expected and um, you basically put in your due date and you put in when you had your last period and stuff like that and it tells you your due date is [date redacted], and you got 18 days left, 37 weeks and 3days and stuff like that. And then um, like you could set when you got an appointment and when you don't

INTERVIEWER: Mm-hm

RDS042: It give you different options like it tell you put your weight in and it will tell you like if you gained good amount or bad amount. Um it's saying like the symptoms of, like did you have like back pains this week, or contractions and stuff like that. And what did the doctor give you for it. And the baby info like, how many kicks this week or this morning? How many pounds did they say the baby was? And like what's the estimate date. Just, it's different stuff and um, what were we talking about again?

INTERVIEWER: Um, so any other apps you have on your phone for pregnancy?

RDS042: That is the most, like that's the biggest one I really click on to all the time. Yeah cause its just, it basically got everything in one. Like sometimes I get a text message from you guys on what to eat and what not to eat. And I have the baby book at home so I read

that a lot. I just always go over the same pages over again. I don't know why, it's just like okay. I can't read about the first month cause one month again.

INTERVIEWER: (Laughs)

RDS042: So I just read the nine months, after the baby's born, and stuff like that. I read that over and over and over and over again. It don't get old to me.

INTERVIEWER: (Laughs) Um, are you signed up for any other like emails, newsletters, or something like that?

RDS042: I get, like I signed up, with my parent and teacher, my teacher signed me up, like we had to sign up for all these like, um you get, like free diapers or free magazines about what to do, and what not to do, when you go to labor and like it was different websites like um, Pampers, um, Huggies, different kinds of websites. I wish I had the paper. Cause we signed up for all these different websites and you get like free gifts or like they mail free diapers to you if you tell them like oh I don't have nothing, stuff like that. They mail you free diapers.

INTERVIEWER: Oh that's nice

RDS042: bottles, if you ever need a breast pump, like you get a chance, like you answer to win a chance to get a breast pump

INTERVIEWER: Mm-hm

RDS042: So, it just a whole bunch of different stuff, and it's like, and it was at school, it was an activity at school so I would never knew about it if I didn't do it at school.

INTERVIEWER: Yeah. And what about um websites that you've found most helpful when you're doing your google searches.

RDS042: Um, it's, like the you, it's like a doctor site. I'm not sure what it's called but it's the one I always click on, it's always highlighted because that's the one they have

INTERVIEWER: Mm-hm

RDS042: The most answers on, cause most people go onto that site or something like that

INTERVIEWER: mm-hm

RDS042: It's helpful. Not all the time, cause you like, that still don't explain it. But like the questions that I really be looking for, most the time they have the answer for me on here. I just click on a whole bunch of different sites to see like if this one is not right and this one. Like if these two are not the same, something not right here.

INTERVIEWER: Mm-hm

RDS042: And I'll ask my mom because she got five kids so. And then I go on the website and see like, let's see if she lying and it would be like around the same, same thing she says, so it would be helpful.

INTERVIEWER: Would you say that, when you have a question about your pregnancy, do you go to the internet first?

RDS042: No, I would go to my parents and teachers. Because I feel like, I know my mom got five kids but most of the time she guessed. Cause she started young. So most of the time she guessing about like what she needed to do and how she needed to do it and what was right and what was wrong. So my parents and teachers, she be doing this for a long time obviously, then she had kids on top of that. So either she got the right answer or.

INTERVIEWER: (Laughs)

RDS042: But most of the time I feel like most of her answers are right. I was like, let's double check this, that's just how I am.

INTERVIEWER: Yeah

RDS042: Like I ask her I believe you, but I'm gonna, you know, see this, see if this got the same answer in case one day you're not here and I want to know something, I'm going to google it.

INTERVIEWER: Mm-hm

RDS042: And on that certain website to see. (Yawn) Excuse me. On that app it has like 35 weeks, it up and like you can type in a question and it would have like other people's answers like when did you start getting heartburn or when did you start feeling the baby kick, when did you pack your bag for the doctor, did you go into labor early with your first child, or did you go late stuff like that. It would be on app cause you could ask questions about that.

INTERVIEWER: And does the app prompt you with all those little questions with kind of fill in the answers?

RDS042: Yeah.

INTERVIEWER: That's neat.

RDS042: You gotta click the from first month all the way to the last month. And you could clikc on whichever month you is. And if you see the answer, like they have all these different things, but I have something different. And you could write your comment on there and so other people can see it. That's helpful.

INTERVIEWER: Um, so in terms of the information that you received, do you feel that you know more about nutrition and exercise now than you did before?

RDS042: Yeah I definitely do cause before I would just eat anything that tasted good. And now it's just like oh maybe I shouldn't eat that because yeah that's not healthy or that's going to make me have heartburn. Or you know,

INTERVIEWER: Mm-hm

RDS042: orange juice that's my thing.

INTERVIEWER: (Laughs)

RDS042: It's gonna be always there. But yeah I know more now than I did before I got pregnant. So it's easier now.

INTERVIEWER: Do you feel more prepared?

RDS042: Yeah

INTERVIEWER: That's good. Um, is there anything that we talked about earlier that maybe you thought of another response or answer to you want to go back and add?

RDS042: Mm I don't think so.

INTERVIEWER: Okay. Um, any final questions or thoughts?

RDS042: Nope.

INTERVIEWER: Okay, well thank you so much for taking the time.

RDS049

Interviewer: Um, so obviously it's been a while since we last talked and it's nice to kind of catch up a little bit, so I just wanted to hear about how your pregnancy has been going.
RDS049: easy.
Interviewer: easy?
RDS049: I don't have discomfort really. Um, my butt hurts a little bit but… (both Interviewer and RDS049 start laughing) I don't really feel discomfort or I'm not really waddling, my belly's not super huge, so, I'm fine.
Interviewer: That's good. (RDS049 tries to say something but then Interviewer continues) Have you had any issues with nausea throughout pregnancy at all?
RDS049: No.
Interviewer: Oh, that's great. (RDS049 starts to laugh and then Interviewer laughs too)
RDS049: I don't have anything like that.
Interviewer: Ah, what would you say has been the best part of being pregnant?
RDS049: Having a belly and going out in public and people like "Oh, my god you're so cute!" (Interviewer laughs a little)
Interviewer: um, last time we talked I asked you what your hopes and dreams are for your baby and um, last time you had told me that, um, that you were really excited and that you hope that you'd be able to play piano and violin together.
RDS049: hmm
Interviewer: do you still have those same hopes? Or added any other ones?
RDS049: No, it's about the same.
Interviewer: Ok.
RDS049: I just want her to be whoever she wants to be and I'll support her.
Interviewer: That sounds perfect.
RDS049: um-hm
Interviewer: uhm… So… um… have you changed the way that you have been eating since the beginning of pregnancy to now?
RDS049: Now I'm more conscious about what I eat.
Interviewer: How so?
RDS049: Like recently, I get like the Boost drinks just to make sure I get all my nutrition. So I drink like nutritional supplements. I eat lots of potatoes, and sandwiches and things like that, except I think about like the health benefits of it more than just the taste, ahuh.
Interviewer: are there any other specific foods, uh, like potatoes that you've tried to add because you heard that they have a certain nutrient in them?
RDS049: No, not necessarily. I just like potatoes a lot. (Interviewer chuckles a little and then continues)
Interviewer: Me too. (laughs)
RDS049: Like, the main thing, I mean, I'm trying to think. What do I eat the most? I can't even think about it right now. I just eat a lot of stuff and I always try to think. The main thing that I've been really forcing myself to eat is cereal. Like milk and cereal. So, I, I eat some Honey Bunches of Oats and some, drink some milk. I don't really like it, but I eat it because I know it's good for me.
Interviewer: is it (RDS049 tries to say something but Interviewer continues) the milk that you don't like?

RDS049: I, uh, yeah, I really never been like a person to drink cow milk. I always was drinking soy milk, but I been trying to drink the cow milk just because, you know, they add the more vitamins I guess, uhuh, so…
Interviewer: um, and does that cause you any like, GI, eer sorry, stomach discomfort or anything like that?
RDS049: not that I think of unless I eat too much of it, but…
Interviewer: Ok. Umm…
RDS049: Just cereal will mess me up.
Interviewer: Let's see… so. Um, it sounds like you're eating a lot of dairy and a lot of things that have vitamins and minerals like cereal and the boost drinks. Um so are there are anythings that you've tried to stop eating? Um because you though that maybe they're maybe not as healthy?
RDS049: Fast food.
Interviewer: Fast food?
RDS049: McDonalds. I don't eat McDonald's burgers anymore. Oh gosh, no. It just taste like crap now. It tastes like fake and I just can't eat that stuff anymore. The most I'll eat is just some fries or get a Mcflurry, but I don't eat their burgers. I don't eat anybody's burgers really. Burger King is an exception, but other than that, I don't really like to eat fast food.
Interviewer: Ok.
RDS049: I used to be a fast food junkie.
Interviewer: How much fast food do you think you were eating before compared to now? Like in the number in the times you would go per week or per month?
RDS049: Three times per week.
Interviewer: Ok.
RDS049: three or four.
Interviewer: And then, how does that compare to now?
RDS049: Once a week. Maybe twice and if I go it's not even for a meal its just for a snack. Like a dessert.
Interviewer. Um.
RDS049: or I'll go there just to get an ice water.
Interviewer: and has the amount of meat that you've been eating changed over the course of pregnancy?
RDS049: I ate a lot during my second trimester and my third I don't really eat that much meat to be honest. But I've had a lot of steak the past couple of days, but steak is not really my thing but I just eat it because its just dinner, but I don't really eat as much meat to be honest.
Interviewer: Ok. Did you say something about like becoming a vegetarian previously? Ok.
RDS049: previously I was a vegetarian.
Interviewer: I'm sorry, what?
RDS049: My parents raised us to be vegetarian and we didn't really eat that much, we didn't really eat nothing like meat at all, so...
Interviewer: Ok and was that something that like, I know you mentioned that you were eating the fast foods: hamburgers and nuts decreased, um, but were you eating other kinds of meat like at home when you make your own meals?
RDS049: not really.
Interviewer: Ok

RDS049: Like if I go to lunch like if I'm at work and I have lunch ill get the little Stouffers with the beef and stuff but I don't' necessarily prepare meat. People prepare it for me so…
Interviewer: Ok.
RDS049: I won't cook it, but if somebody else cooks dinner and they have meat then I'll eat it.
Interviewer: Ok, um, so other than fast food is there anything else that you tried to remove from your diet because you heard that it could have health benefits for you or the baby?
RDS049: removed?
Interviewer: mmhm.
RDS049: yeah, just mainly fast food.
Interviewer: ok. Mm. what about the ways you prepare food at home? Has that changed at all?
RDS049: No. So I just all my onions and my peppers and all that other good stuff. I love my veggies and my broccoli and all that other good stuff.
Interviewer: how do you normally cook those? Are they microwaved? Boiled?
RDS049: I put a little oil in the pot. Oh if I make broccoli like my favorite thing to make are the little quick, well it's not really quick, but easy dinners to make so I'll boil the potatoes for like an hour and I'll put some butter in the skillet and put some broccoli and some onions and put some garlic salt and pepper. That's my favorite way to eat it.
Interviewer: Ok. Umm and then other vegetables do you stir-fry those?
RDS049: pretty much.
Interviewer: Ok. Have you had any challenges with certain types of foods making you feel sick or anything like that?
RDS049: no.
Interviewer: Sounds like the perfect pregnancy. Can't ask for much more than that.
RDS049: yeah, I had it easy, but I think I have an easy pregnancy because I have to worry about a whole bunch of other stuff so I can't be pregnant and have to go to work and so all this other stuff because then this really just wouldn't work.
Interviewer: there's always something isn't there?
RDS049: right.
Interviewer: um, so in terms of learning about pregnancy since this is your first one, um, was there a person or a place and by place I mean a book or the internet, um, and that person could be family, friends, doctor, um any person or place that has given you the best information on pregnancy and health?
RDS049: The best? March of dimes.
Interviewer: mhmm.
RDS049: And then I usually if I have a question, if I have a question and something happens to me, I just Google it and read a whole bunch of different takes on it so I go to like multiple websites. Like the other day I lost my mucous plug and I was like googling it and reading everybody else's take on it just so I could have my own idea of what's going on.
Interviewer: And when you Google things, how do you decide which links to click on?
RSD: Well, I don't click on forums. I don't click on forums unless I want to hear other people's experiences, but for the most part I'll click on like what to expect, or something that shows that it's informational; that's what I'll click on.

Interviewer: Ok. And other than March of Dimes, do you have any other websites that you thought have been helpful?
RDS049: Mainly What To Expect.
Interviewer: Um, and where do you typically gain access to the Internet?
RDS049: from my phone or my tablet.
Interviewer: So, do you have wireless at your um home?
RDS049: mmhmm.
Interviewer: Ok. And then of all the different topics that you've looked up on Google, which things, which topics do you think you most frequently searched for?
RDS049: labor.
Interviewer: Labor? (RDS049 laughs) That makes sense. I would have a lot of questions about that too. Um, and about how often do you think that you used What To Expect and March of Dimes and other websites like that?
RDS049: Probably like 5 out of 7 times a week. Well 5 out of, 5 days out of 7 days of the week.
Interviewer: Ok. Um, and so then… you did Facebook and text messaging for this study, and how often did you visit the Facebook site?
RDS049: Well, the notifications came to my phone so I had to look at it. (I think she said Urgence) is going to show one there so pretty much every time something was posted or if I happened to be scrolling through my newsfeed and I see something in the feed that I didn't pay attention to, I'll like it or something like that.
Interviewer: Ok, so the notifications were, sounds like, very helpful of knowing when to look?
RDS049: Mmhmm.
Interviewer: And then, what about the text messages? Do you just look each time they would come in?
RDS049: Yeah, pretty much. I look at them and I think like should I respond to this? I don't know if I should text back, I don't know. (both RDS049 and Interviewer laugh a little)
Interviewer: Um, so overall, how helpful do you think that um (there is a gap in the audio) Facebook and the text messages were?
RDS049: Very helpful because they give me something to think about. Something I might want to apply to my life, or apply to my eating habits. Or just yeah in general like the other day I seen something about exercise and I just went to uh, me and my best friend went to the basketball court and he was shooting hoops and I was just walking around the court and trying to do a little exercise. So, it felt good to do some exercise.
Interviewer: Um, are there any other things that you can remember that prompted you to try or do something specific? Some of the messages?
RDS049: I just remember something about yoga and about getting a good night's sleep and stuff like that, but I've been sleeping pretty good up until the past couple of days, but the whole time I can sleep through the whole night, get a nice long sleep, wake up early, go to work and come back and go to sleep like average. Uhuh.
Interviewer: Um, of all the different types of topics that you've received information on, was there a certain topic that you thought was most helpful for your pregnancy? So we sent a lot of fitness stuff, a lot of nutrition stuff, and then sort of I guess what I would call general wellbeing like mind and body…

RDS049: Just mainly the nutrition stuff because I've always been someone, I've never really been the one with good eating habits necessarily, I just been really, I just ate whatever eat junk food (audio gap) or anything like that because I was like well I'm not fat so I can eat whatever I want, but it's not like that so I try and eat and I care about like how it's benefitting me more, so how, what I'm eating it's benefitting me more so…
Interviewer: And did you learn anything new from the messages that you can think of?
RDS049: Um, I don't know, I got a lot of it. It's just basically, It's just basically like from foods or different vitamins. I think it was like something about greens. I remember this one text message that made me want to eat greens because it's like it had like about iron I think? (Interviewer interrupts with an umhm in agreement) like yeah, It was about eating your greens because it has iron so I was like well let me go eat some spinach. Or I went to Wegman's and got some garlicky kale and it was actually really good. And some green beans and some Chinese food.
Interviewer: Cool. Um so sometimes you received messages that had web links, did you ever click on the weblinks?
RDS049: Honestly, I don't trust web links. Like I always think it's gonna spam me so…
Interviewer: Ok. Um, so in relation to that um since the web links that were sent were from the study um is there a certain person or I guess way that you could receive a link that would make you feel comfortable clicking on it?
RDS049: On Facebook. Uhaha. Not, not on text messages though. I think that's going to spam my phone.
Interviewer: Ok. Um so some of the ones on Facebook probably had, um, web links so did you ever click those ones or, still like worried about like viruses and stuff like that?
RDS049: I do remember clicking on one on Facebook that was like a, like a contest that you like taking a picture of your meal and I was going to do that but I was just forgot about it and I just ate my food and then I didn't remember til the end so it was just like whatever.
Interviewer: mhmm.
RDS049: But… that's all I can really recall.
Interviewer: If um, your doctor was doing something like this study where they sent messages would you feel more comfortable clicking on the links knowing that it came from your health care provider?
RDS049: Probably, but I just really don't. If you know about viruses and stuff or like spam, it always somebody sending you a link and telling you to click there and then you get a whole bunch of spam. (Interviewer says ok in the background) So I'm always like skeptical like, even if it's somebody that I can trust, it's just like if it doesn't look like an actual website, like you know how when you copy and paste a web link and put in into like a short (Interviewer says mhmm in agreement) a short website it's the actual website then I don't really want to click it, but if it's like the actual whole website it's not like bics dot ly like you know that kind of stuff cuz that's when they shorten it then I won't, then I will click it, but if it has that, then I won't click it.
Interviewer: I see. Um so for ty- other types of messages, um so sometimes you got pictures sometimes um, it would say that it was a video and it would've been a shortened url for that as well um, and then also the short sentence long messages and then on Facebook obviously they would be longer. Are there certain types of messages that you liked the most?

RDS049: Just anything that had like a picture or something that looked like, catch your attention.
Interviewer: Okay. That's kind of cool. Um, and what motivated you to, to keep clicking on like the notifications to see what the message was or the text messages… because know sometimes just things get boring and you're just like ehh I'll look at that later and then…
RDS049: Nah, it's just because it's information. You can't go wrong with knowledge so that's why I just clicked it just to see what I could learn.
Interviewer: um, is it hard for you to, to balance all of the other things that are coming in from friends, from family, on facebook and texts like with the number messages that we sent?
RDS049: No, because if I'm at work I won't do anything, but once I'm out of work, I don't have anything to do so I'll actually take the time and just do everything on my phone cause I can't do anything on my phone while I'm at work so…
Interviewer: Do you think that the number of messages that you received each week was too much?
RDS049: No.
Interviewer: Ok. Um and were there any topics that you found yourself curious about that you wish we would've sent?
RDS049: Labor. Uhuh (laughing)
Interviewer: Labor? Um, so another um important topic that was kind of discussed fairly regularly was weight gain, and so I was just wondering what your thoughts were about the messages that related to weight gain?
RDS049: Well I paid attention to them simply because I didn't really gain much weight. I probably gained about 25 to 30 pounds which is like average but I would've expected more seeing that I have a baby inside me, but I don't know. I tried to gain more weight but, it's like I can't really control that. The only thing I can do is eat as much as I want to; that doesn't mean I'm going to gain weight.
Interviewer: So are you happy with the amount that you gained then? You feel like it's good to have a healthy baby?
RDS049: Well, it's so they say, I mean right now my baby is like six pounds and they say that she's measuring small which kind of makes me feel bad cause like why am I not gaining the weight that I need to gain, but I don't know. If I could be like 160, I'll be ok, I'll be happy. So it's like 35 pounds.
Interviewer: And has your midwife said anything about the weight gain? Like is she like concerned or?
RDS049: mhmm (in disagreement) not about my weight. I just feel, I just don't like it, but I wish I could gain like, I feel like, I'm at 150 so if I have ten more pounds, I'll feel okay.
Interviewer: mhmm. Um, did any of the messages that were sent change your view about weight gain?
RDS049: Um, well most people complain about weight gain and they feel like there is a healthy way to do it and there's a not healthy way to do it.
Interviewer: And so you aren't too worried about gaining weight because you knew it was good?
RDS049: mhmm
Interviewer: Ok. Um, and what about physical activity? How has your activity level changed over the course of pregnancy?

RDS049: Well, I was always active, always went for walks and things like that so I mean it's I mean I'm less active, but I'm still active. Before, I'd always like go out for a walk and always go walking somewhere. Now I'm more like busier, I'm tired and I just wanna eat, but I'll still go and meet my friends. I'll go for a walk with them.
Interviewer: And you mentioned yoga earlier. Um is that something you've tried from any of the messages?
RDS049: mhmm (in disagreement) I always read it but I was like I should try it, but it's too much work to find the time to do it, so I was just like whatever.
Interviewer: Ok. Um, and during the course of your pregnancy, did you have any cell phone trouble where like, um, your, you know you lost your phone, or um, didn't have access to a cell phone for a period of time?
RDS049: No, I mean the most that happened ever is that I got a new phone, but I just traded it. Like you know did my upgrade like right then and there. (Interviewer: mhmm) I always had like the same phone, so, same number and it was always on, so…
Interviewer: Um, and did you have access or did you download any pregnancy apps for your phone?
RDS049: mhmm
Interviewer: Can you tell me a little bit about those?
RDS049: Well, this my new phone. My other phone had more, but I had one that's called "I'm Expecting, WebMD, Pregnancy, The Bump, WhatToExpect," Yeah that's pretty much it.
Interviewer: Um and of all those apps, how often do you get messages from those?
RDS049: Once a week.
Interviewer: Ok. Do you have a favorite?
RDS049: umm, hmm. I like the "I'm Expecting" one just because it's like pretty pictures and the information is like conversation more than like coming at you with information, information, information. It's more conversational so it's like friendlier and it's like pictures of babies so I like that app the most.
Interviewer: And so then for the study that you were doing, how, so it sounds like "I'm Expecting" you said it's conversational and, and not just informational. Um, how do the way the messages were written in your apps compare to the messages you received in this study?
RDS049: The way that you sent them, well the way the text messages, they were conversational as well. It was just like, it was like uhh everytime I was reading it, it was like I was reading it with an exclamation point like "Hey! Bla bla bla bla bla!!" And then like I don't know. It was conversational as well too but it was just more like out there. (Interviewer: mhmm)
Interviewer: Um is there any way that you like would want to see the messages changed or could they have been written better or?
RDS049: No, they were fine.
Interviewer: Ok. Because I know some topics like, um, because I mean the program is meant for all different women who are coming from all different backgrounds and experiences so that we know there were messages about drugs and alcohol as just a reminder that they're not, not good, but I know they're hard topics that you know (RDS049: talk about) yeah, so I mean you would have gotten those as well because we sent them to everyone. But how, I don't know if you remember how they were written, but do you think

that that was a good way to write about things like that so that it's not like hey you're doing this stuff bad…
RDS049: (coming in) Hey don't do that remember that it has effects on your baby. And I was like whatever, I don't do that so…
Interviewer: Um, and did you ever, I don't know; do you have any other friends or do you do any other groups where you can talk to other pregnant women?
RDS049: Well another friend of mine is pregnant but she's like way way at the beginning and I'm way way at the end so it's like I just tell her if she has questions. But other than that, there's not like a group or anything.
Interviewer: mhmm, have any of the messages you received either from the study or your apps been things that you have shared with her or your own experiences also?
RDS049: Yeah, I'll just tell her like good foods to eat and things like that.
Interviewer: mhmm. Um and so , thinking back to all the messages, and you're welcome to look at your phone and take as long as you want, I'm just curious as if you had any favorite messages that really stood out to you or maybe even that you went back to and visited more than once?
RDS049: Umm, let me see… I would say, I guess, maybe the one about water, H2O, (Interviewer: mhmm) Like, I'd never been somebody who liked to drink water, but now I do. So it was just like a good reminder like better drink your water.
Interviewer: And were you drinking the water more because of the message, or, or was it something else like your midwife?
RDS049: It was just like a reminder. So it's like on a day I wouldn't feel like you know getting a cup of water and I seen that, I was like oh let me go get some water because I should be drinking water.
Interviewer: mhmm. Um, Can you think of any other examples of where you got a message and it actually prompted you to get up and do and try something different?
RDS049: Umm, the one, the little excerpt about carb, carbo- carbohydrates. I was like oh, let me see. The next thing I eat I'm gonna look at the nutrition facts and see how many carbs are in it and things like that.
Interviewer: And what about uh, fruits and vegetables. I remember the last time that we talked, you said that um, that you ate a lot of fruits and vegetables. Has that changed at all?
RDS049: No, I still do.
Interviewer: Ok. Umm, and before being in this study were you familiar with whole grains?
RDS049: Mhmm (Interviewer: Ok) that's the only type of bread my dad used to get.
Interviewer: (laughing) So you're well, very knowledgeable about whole grains then? Umm, would you have liked to receive more messages throughout your pregnancy?
RDS049: I mean I think that the amount that you send was pretty good, just because I already get notifications or like you know, from Facebook, or it's always something reminding me that I'm pregnant like every day. Like there's nothing new. But it's always good like that you know 2-3 times per week. I don't even remember I feel like twice a week? Yeah, twice a week like yeah it was perfect cuz it was like here's your friendly reminder, this is something good to do. Here's a tip. So, I think it was fine. I think more would've been too much and then less would've been not enough.
Interviewer: Ok, so kind of a daily reminder would sort of help things to keep you kind of thinking about all the different parts of pregnancy that are important. Um, so do you think that a program like this would be nice to have provided through your healthcare provider?

RDS049: Yeah.
Interviewer: Um, and so you, since you did Facebook and texts, do you think that for all um, teens who are pregnant there, that one is better than the other?
RDS049: Um, well Facebook because everyone is on Facebook. Well most teens I guess you could say are on Facebook, so at least if they're on Facebook they, they're gonna look at it because you don't want to look at that number, that one bubble (referring to notification center) so you have to click on it; you have to see what it is. So that's the way. Because I wouldn't leave the notification there.
Interviewer: That makes sense. Um and I know everyone is different and some people are, are more private and shy and so kind of our goal with Facebook was to allow communication with other girls, but from our experience a lot of people don't really interact that way on the site. Do you have any thoughts on why some of the teens may or may not have participated?

RDS049: They probably didn't really care, honestly (Both RDS049 and Interviewer start laughing) didn't really care just like whatever let me just do this. I actually like to learn stuff so that's why I would make sure that I would show that I'm paying attention, by liking stuff every once in a while.
Interviewer: Um and what is it about the messages that you liked compared to the ones that you didn't press the like button for?
RDS049: Um, If it pertained to something in my day (Interviewer: mhmm) So it was like if you were talking about drinking water, I was like, oh, I drank all my water today so I'm going to like that.
Interviewer: Um and so it could've just been to like the day that it was and that message just particularly fit in with what was going on?
RDS049: Mhmm.
Interviewer: Ok, umm so actually I think that's all of the questions that I have. Um but have you thought of anything else in response to a question that we already talked about that we could go back to?
RDS049: uhhmm (in disagreement)
Interviewer: Ok, um do you have any questions for me about anything related to this study that you were curious about or?
RDS049: Well what else do I have to do for this study?
Interviewer: Um so this is your last interview and then that survey that I was talking to [name redacted] about, that is the last thing that you have to do and then you're all done with it, so um (RDS049 begins to giggle a little then Interviewer joins in) um but you are in the vitamin D study too so that one, that goes through your delivery so then you'll still have the pills and then maybe a couple more surveys for that, but I'm not sure what you have left for that one, um but yeah, this smaller part is all done and actually, I think that you received literally every message that we had developed so (Interviewer and RDS049 begin to laugh) um, so, once you're done today, then there won't be any more messages which is kind of sad if you were really enjoying them and I apologize for that, but um thank you so much for participating. It's been great having you and honestly you were like an ideal participant by liking things and like responding. Very nice. (RDS049 and Interviewer begin to laugh). So thank you. Um, so we actually finished a little bit early so I'm gonna just send a text really fast to Melissa and see if she can head back down.

RDS049: She said she had to talk to Audrey before my appointment, so…
Interviewer: Yeah…
RDS049: (starts to laugh)
(long pause)
Interviewer: So you had a baby shower?
RDS049: mhmm.
Interviewer: yeah?
RDS049: yeah it was fun.
Interviewer: did you get lots of stuff?
RDS049: yeah, I can't wait to see my baby in all those cute clothes…
Interviewer: That's awesome.
RDS049: … and play with her Johnson and Johnson sets and I love Johnson and Johnson.
Interviewer: Are you having a boy or a girl?
RDS049: Girl
Interviewer: Did you pick a name yet?
RDS049: Her name is going to be [name redacted].
Interviewer: Oh, pretty. I like that.
RDS049: Thank you. I can't wait.
Interviewer: Only a couple more weeks!
RDS049: I know, but I just is excited so I can get it over with so that I can just relax and I don't have to go to work just sit there and be a mommy and my job could just be tending to her needs. If she's crying, feed her. If she needs a change of diaper, change her. So yeah.
Interviewer: That will be nice.
RDS049: Yeah.
End of interview between Interviewer and RDS049
