# Supplementary material for: A social media intervention to improve nutrition knowledge and behaviors of low income, pregnant adolescents and adult women
Source: PLoS One. 2019 Oct 24;14(10):e0223120. doi: 10.1371/journal.pone.0223120 (PMC6812786; doi:10.1371/journal.pone.0223120)
Supplement: S1 Table — (DOCX) [file pone.0223120.s009.docx]

| **Table S1**. Message Content for Text and Facebook Posts | | | |
| --- | --- | --- | --- |
| **Post Type** | **Delivery Mode** | **Topic Category** | **Message** |
| **Alternatives** | Facebook | Fast Food | Like McDonald's? Think the McChicken Sandwich is less calories than the cheeseburger? Think again! The cheeseburger has fewer calories. Source: http://webmd.com/diet/healthtool-food-calorie-counter |
|  | Text | Condiments | Instead of mayonnaise, choose mustard. It's a low-calorie option and has just as much flavor! |
| **Challenge** | Facebook | Hydration | Did you know that staying hydrated (drinking plenty of fluids) can help prevent constipation? Do a little research by drinking 6-8 glasses of fluid each day this week. A PRIZE goes to mommies who shares their progress on the Facebook page! |
|  | Text | Hydration | Fight constipation this week by drinking 8 glasses of fluid each day. Let us know how you did and earn yourself a PRIZE! |
| **Fact** | Text | Cravings | About 1/2 of women have a craving during pregnancy. Find out what it means here: http://www.babycenter.com/0_food-cravings-and-what-they-mean_1313971.bc and tell us about your cravings so far. |
|  | Text | Weight Gain | Eating for 2 isn't all it's cracked up to be! Believe it or not, you only need about 300 extra calories a day when you are pregnant. Source: http://www.marchofdimes.com/pregnancy/weight-gain-during-pregnancy.aspx |
|  | Text | Supplements | Prenatal vitamins are great. Take them every day to get enough folic acid and iron, two things your baby needs! Your baby will thank you. Source: http://www.mayoclinic.com/health/prenatal-vitamins/PR00160/NSECTIONGROUP=2 |
|  | Text | Fruits & Veggies | Go BANANAS! This tasty fruit is always budget friendly and packs a major health punch for you and your baby. |
|  | Text | Vitamin D | Getting down with vitamin D! This important nutrient can help fight infection and keep you and baby healthy and happy. |
|  | Text | Vitamins/Minerals | B-vitamins can help you feel happy during pregnancy. Get all you need for the day from one baked potato or one banana! http://www.youtube.com/watch?v=i7ikrxvC1q0 |
|  | Text | Fruits & Veggies | Today is Tasty Tuesday, so grab an apple to go with this fun fact. There are 2,500 types of apples in the USA and 7,500 kinds in the entire World! |
|  | Text | Grains | Try instant oatmeal for breakfast on chilly mornings. It's quick and packed with whole grains, fiber, and nutrients to keep you feeling strong all day long! |
| **Fun** | Facebook | Fruits & Veggies | Playing with your food is totally cool when fruits and veggies are involved! Love your baby by getting in the spirit with me. Share a funny pic or video! Here is a pic of a pear I just ate and a super cute video about loving veggies! YUM! http://youtu.be/on76n-8ffEQ |
|  | Text | Fruits & Veggies | Playing with your food is totally cool when fruits & veggies r involved! Love your baby by getting in the spirit with us. Here's a cute video! http://youtu.be/on76n-8ffEQ |
|  | Facebook | Weight Gain | Crazy fun fact: The average American consumes around 4,500 Calories on Thanksgiving! That is more than twice the amount a person should usually eat. Don't sweat one day of extra eating if you get to enjoy the day with friends and family! Happy Thanksgiving!!!! Source: http://newsfeed.time.com/2012/11/20/is-a-thanksgiving-dinner-really-4500-calories-maybe-not/ |
|  | Facebook | Feel Fab | Notice any differences in your hair? Thanks to pregnancy hormones, you might be losing less hair. That means, your hair will feel healthy and look fuller. Enjoy your fabulous locks with a cute new haircut or up-do! |
|  | Facebook | Feel Fab | Who doesn't love a relaxing bath once in a while? Pamper yourself with these. Do-it-yourself bath bombs! Not only do they smell good, but they also make your skin glow and feel soft. http://www.homemadesimple.com/en-US/Crafts/Pages/homemade-bath-bombs.aspx |
|  | Facebook | Grains | Most cereals have whole grains and fiber. Tell us your favorite type and we will let you know how much fiber you get from each delicious bowl. |
|  | Text | Feel Fab | Chillax a little this weekend with this music playlist. http://www.youtube.com/watch?v=kxkwnBX6LGg |
|  | Facebook | Relax | Make this Friday a movie night so that you can chill out in some comfy pajamas and enjoy some popcorn (a whole grain). What move will you watch? |
| **Information** | Facebook | Cravings | Cravings…Should you fight them or dig in? Whether you are craving pickles and ice cream, or crackers topped with icing, your feelings are very normal. Many women have one or more cravings during pregnancy and these feelings can change over time. Experts suggest that the normal hormone changes that occur while pregnant can cause these intense wishes for food. Some doctors think this may be your body telling you that you and your baby need a certain nutrient. However, if you start having cravings for ice or non-food items like ice or dirt it may mean something more serious. Visit your doctor soon if you have cravings like this so that they can make sure you have enough iron, an important nutrient for your baby. Learn more about what cravings mean here: http://www.babycenter.com/0_food-cravings-and-what-they-mean_1313971.bc |
|  | Text | Cravings | Whether you are craving pickles and ice cream, or crackers topped with icing, it's totally normal. Learn more: http://www.babycenter.com/0_food-cravings-and-what-they-mean_1313971.bc |
|  | Facebook | Supplements | Taking vitamins doesn't have to hurt your stomach. Eat them with a snack and drink lots of water to help avoid stomach pain. If you forget your pills, try placing the bottle next to your bed or by the sink in the bathroom. Seeing the bottle will remind you to take your pill each day. How do you remember to take your pills? Source: http://www.mayoclinic.com/health/prenatal-vitamins/PR00160/NSECTIONGROUP=2 |
|  | Facebook | Fruits & Veggies | Save big when you buy fruits and veggies that are in season! Check out this video for seasonal options during any season: http://www.youtube.com/watch?v=hwCl97ehfak |
|  | Text | Fruits & Veggies | Fresh fruits & veggies are costly & go bad quickly. So, buy them frozen instead! Not only are they cheaper, but they are just as healthy and last much longer! |
|  | Facebook | Fast Food | The next time you get hungry while you are out and about, try one of these healthy fast food options. Don’t forget to share your favorites with the group too! http://www.babycenter.com/0_10-fast-foods-a-pregnant-woman-could-love-and-five-to-avoid_1313899.bc |
|  | Text | Portion Control | Instead of aiming to eating a certain number of meals each day, try to eat smaller meals or snacks every 4 hours. This can prevent overeating. Source: http://www.webmd.com/diet/healthtool-portion-size |
|  | Text | Breakfast | Eating breakfast each day will keep you feeling great! So, fuel up with your favorite cereal or a glass of milk and apple. |
|  | Facebook | Hydration | Busy ladies need to drink lots of water! Your baby will feel happy and you will feel energized! |
|  | Facebook | Sleep | Trouble sleeping? Snuggle into a good sleep position at night with a pillow between your knees & a pillow or rolled up blanket under the edge of your baby bump. |
|  | Text | Sleep | You'll sleep like a baby if you limit your caffeine intake. Try drinking soda, coffee, and tea before 3 PM for a good night of sleep. |
|  | Text | Antioxidants | Brighten up your plate! Add some color—like ORANGE carrots, RED bell peppers, PURPLE eggplant, and GREEN collard greens. |
|  | Facebook | Easy Cooking | Make a large pot of soup or chili over the weekend and split it into meals for the week. You will save a lot of time! Check out a few of our favorite recipes. Sources: http://www.myrecipes.com/recipe/basic-beef-stew-with-carrots-mushrooms-10000001142004/ http://www.myrecipes.com/recipe/beef-pinto-bean-chili-10000001949758/ http://www.myrecipes.com/recipe/chipotle-chicken-tortilla-soup-10000001087094/ |
|  | Text | Vitamin D | Vitamin D pills are helpful to you and baby because most people don't get enough vitamin D in their diet. Make sure you take your pills every day to avoid illness. |
|  | Facebook | Vitamin D | Do you have that baby GLOW? Celebrities like Nicole Ritchie know how important 10-30 minutes in the sun can be for their babies. Just a short amount of time in the sun can create all the vitamin D mommies-to-be need each day. Catching some rays can be tough in the winter though. So, make sure you keep taking those vitamin D pills so that you can ROCK that soon-to-be mommy GLOW! Don't forget to eat foods like tuna, salmon, egg yolks and low-fat milk too. They are packed with vitamin D too! Source: http://www.webmd.com/food-recipes/guide/calcium-vitamin-d-foods |
|  | Text | Drugs & Alcohol | Your baby deserves the best in life. Quit smoking, drinking and using drugs today. We know you can do it! |
|  | Text | Drugs & Alcohol | Smoking, drugs and alcohol are not good for baby. Using these during pregnancy might result in your little bundle of joy having behavioral and learning problems. |
|  | Facebook | Development | How far along is your pregnancy? Tell us below and we will share a fun fact based on how far along you are. |
|  | Text | Supplements | Prenatal vitamins can be hard to swallow but don't give up. You and baby need all the nutrients you can get to stay healthy. |
|  | Facebook | Exercise | Have you ever thought about trying yoga? Pregnancy is a great time to test out some of the moves on page 2 of this article. As your pregnancy gets further along, be sure to listen to your body and hold onto a chair for extra balance if need it. Don't forget to check with your doctor before starting any new exercise plan. http://www.babycenter.com/0_great-pregnancy-exercise-prenatal-yoga_7862.bc?page=1 |
|  | Text | Exercise | Have you ever thought about trying yoga? Pregnancy is a great time to test out some of the moves on page 2 of this article. http://www.babycenter.com/0_great-pregnancy-exercise-prenatal-yoga_7862.bc?page=1 |
|  | Text | Grains | Great Harvest Bread Co. is a great place for a sweet treat that’s made with whole grains. YUM! Visit at 1890 Monroe Ave, Rochester, NY. |
|  | Text | Sleep | Trouble sleeping? Try drinking a cup of warm milk before bed time. The warmth will put you & baby on course for a night of restful Zzzzz's. |
|  | Text | Hydration | H2O will help you go! Drink 8-10 glasses of water each day to feel your best. |
|  | Text | Supplements | Folate, iron, calcium and vitamin D keep you and baby healthy and happy. Find them in green leafy veggies, red meat, milk and fish. |
|  | Facebook | Fruits & Veggies | Here is a new veggie to try: Cauliflower! This white veggie is a close relative of Broccoli and provides 100% of the vitamin C you need each day. Try it with cheese or in a fun recipe: http://healthymeals.nal.usda.gov/hsmrs/NJQuickSteps/NJ_Qk_Steps_Participant/Cauliflower.pdf http://allrecipes.com/recipe/roasted-garlic-cauliflower/ |
|  | Facebook | Breastfeeding | It is never too late to think about breastfeeding. It is great for baby and it can help you lose the baby weight much faster after you deliver. |
|  | Text | Exercise | Good posture is ur ticket to a pain-free back! When standing, sitting or kneeling be sure to keep ur shoulders drawn back & a small curve in ur lower back. |
|  | Text | Meals | Variety is the spice of life! Bring meaning to your meals by mixing colors, tastes and smells. |
|  | Facebook | Oral Health | Prep your smile for the arrival of baby with proper oral care. Keeping up with brushing, flossing and visits to the dentist will prevent the growth of bacteria that causes bleeding and soreness of the gums while pregnant. Isn't your baby worth brushing for? |
|  | Text | Vaccines | Pregnancy can make fighting off the flu tough. Getting a flu shot can prevent u from getting sick & also keep baby flu-free after birth! Now that's a win-win! |
|  | Facebook | Exercise | Check out this tough Crossfit mom! http://www.huffingtonpost.com/2013/09/19/pregnant-crossfit-mom-facebook_n_3956608.html?utm_hp_ref=mostpopular What do you think about this kind of exercise during pregnancy? |
| **News** | Facebook | Mood | Recent research shows that feeling depressed while pregnant can hurt your baby's brain growth. Try meditating or listening to peaceful music to blow those baby blues away. |
|  | Text | Fruits & Veggies | Brighten up your plate! Add some color—like ORANGE carrots, RED bell peppers, PURPLE eggplant, and GREEN collard greens. http://www.medicalnewstoday.com/releases/268631.php |
|  | Facebook | Exercise | Struggling to get in a good workout? Good news! Moving around most of the day can be better than a 30 min workout. Read about this surprising news! Source: http://www.fitpregnancy.com/exercise/prenatal-workouts/simple-way-keep-your-weight-check |
|  | Facebook | Exercise | An interesting study shows that working out makes your baby smarter. Read more about this new finding here: http://www.fitpregnancy.com/exercise/workout-makes-your-baby-smarter |
|  | Facebook | Supplements | Here is one more reason to keep taking those supplements! A recent study found that the vitamin D in your pills could decrease your chances of getting preeclampsia. This severe disease affects women after the first 20 weeks of gestation and causes high blood pressure and protein in the urine. Source: http://www.medicalnewstoday.com/articles/271768.php |
|  | Facebook | Drugs & Alcohol | Marijuana use while pregnant can impair your baby's brain growth, according to a recent study. Help your little one think like a champ by dodging this drug. No amount is safe. Source: http://www.sciencedaily.com/releases/2014/01/140127093140.htm |
|  | Facebook | Cravings | What have you been craving during your pregnancy? A) sweets, B) salty snacks, C) spicy foods like Mexican, D) meat, E) fruits and veggies, F) ice, G) other We'd love to know more about your cravings so post a comment below with all the details. |
| **Poll** | Both | Dairy | What is your favorite type of dairy? A) milk, B) yogurt, C) cheese, D) ice cream, E) All of the above, F) None of the above |
|  | Both | Shopping | Who does the shopping for the food you eat? A) Mom, B) Grandparent, C) Father, D) Boyfriend, E) You, E) Relative, F) Other |
|  | Facebook | Snack | What is your favorite snack? A. ice cream B. popcorn C. Chips D. Fruits E. Chocolate F. Other If you said other, share below! |
|  | Facebook | Fruits & Veggies | Like chips? Like collard greens? The new fad is green chips! These are super easy to make and packed with vitamins for you and baby! Just follow these easy instructions: http://www.foodnetwork.com/recipes/alton-brown/baked-greens-chips-recipe/index.html Upload a picture of your amazing chips! |
| **Recipe** | Text | Fruits & Veggies | Like chips? Like collard greens? The new fad is greens chips! These are super easy to make and packed with vitamins for you and baby! http://www.foodnetwork.com/recipes/alton-brown/baked-greens-chips-recipe/index.html |
|  | Text | Grains | Craving Grandma's fried chicken? Check out this crunchy, low-fat version that has whole grains! http://www.wholeliving.com/132183/cornflake-crusted-baked-chicken?czone=e&center=144884&gallery=142700&slide=132183 |
|  | Facebook | Grains | We are excited about all those buns in the oven out there. Celebrate with us by making whole grain sticky buns with your friends. This is our favorite recipe: http://www.tasteofhome.com/Recipes/Multi-Grain-Cinnamon-Rolls |
|  | Facebook | Fruits & Veggies | Frozen grapes are a tasty, low-calorie and low-fat treat that can even make the dessert lover's mouth water. Just stick some in the freezer, and they will be ready the next day! Source: http://www.google.com/imgres?imgurl=http://stylishcuisine.com/wp-content/uploads/frozen-grapes-1.jpg&imgrefurl=http://stylishcuisine.com/?p%3D1360&h=366&w=550&sz=73&tbnid=cmTQwT1rNVGrnM:&tbnh=90&tbnw=135&zoom=1&usg=__8MYIhVlRO9EBnrIF4q5n0oayci8=&docid=FF9k1LWRjiRKPM&sa=X&ei=31GiUtS1GvXJsQTNn4DQDA&ved=0CFMQ9QEwAw |
|  | Facebook | Fruits & Veggies | Love fruity drinks? Try making this yummy strawberry-banana smoothie! Not only is it really good, you will feel super after drinking it. Check out the recipe! http://www.ivillage.com/10-super-snacks-you-can-make-under-5-minutes/4-b-212237#212243 |
|  | Facebook | Low-fat, high-protein | Looking for a tasty meal that is also healthy? We've got you covered with this low-fat, protein-packed shrimp & pasta dish. It has lots of key nutrients like calcium and iron! Source: http://www.eatingwell.com/recipes/garlicky_shrimp_vegetable_pasta.html |
|  | Text | Snack | Who says holiday cookies can't be healthy and YUMMY? Here is a great low-fat cookie! http://www.myrecipes.com/recipe/chocolate-peanut-butter-cookies-10000001860055/ |
|  | Text | Fruits & Veggies | A chicken veggie noodle bowl makes a nice warm dinner on chilly days! http://www.familycircle.com/recipe/chicken/chicken-veggie-noodle-bowl/ |
|  | Text | Fruits & Veggies | Warm up with a hot breakfast that is berry, berry good for you! http://allrecipes.com/recipe/todds-famous-blueberry-pancakes/ |
|  | Facebook | Meals | Wrap it up for lunch ladies and see how easy it is to get 65% of the folate you need each day! This recipe also packs in tons of iron and B vitamins, so you feel great! http://bit.ly/MaCvBz |
|  | Text | Holiday | Share the love this Valentine's Day by enjoying a healthy yet yummy meal http://bit.ly/1bcU8fZ http://allrecipes.com/Recipe/Penne-Pasta-with-Spinach-and-Bacon/?prop24=hn_slide1_Penne-Pasta-with-Spinach-and-Bacon&evt19=1 …dessert included! http://www.foodnetwork.com/holidays-and-parties/packages/valentine-s-day/valentines-day-with-fn-chefs.0203422.html |
|  | Facebook | Holiday | Happy St. Patrick's Day Mommy! Throw on a green t-shirt and enjoy some healthy Irish comfort foods this St. Patrick's Day! Source: http://www.webmd.com/food-recipes/features/st-patricks-day-dishes |
|  | Facebook | High-protein | Make this awesome, healthy soup in just 10 minutes! The two most health-hearty ingredients are the protein-filled beans and the nutritious tomatoes! Source: http://www.myrecipes.com/recipe/spicy-tomato-white-bean-soup-10000001215922/ |
|  | Text | Dairy | Dairy…It's MOO-velous! Next time you hit up the drive-thru, treat yourself to a yogurt parfait or milkshake made with low-fat milk. |
| **Snack** | Both | Weight Gain | How much weight you gain during pregnancy depends on how much you weighed before you became pregnant. Click the link to see if you are gaining the right amount of weight: http://www.babycenter.com/pregnancy-weight-gain-estimator |
| **Tool** | Text | Weight Gain | Worried about losing that baby weight after your delivery? Here is an easy tool to help you reach your weight loss goals. Source: http://www.webmd.com/diet/food-fitness-planner/default.htm |
|  | Facebook | Shopping | Buying seasonal fruits and veggies can help you save money. Now that it is getting colder, look for bright and juicy apples or pears. http://www.youtube.com/watch?v=EF50oyEu6Hg |
| **Video** | Facebook | Meals | Thanks to Michelle Obama schools are serving up healthy lunches. What options are you trying at your school? http://www.doctoroz.com/videos/michelle-obamas-school-lunch-taste-test-pt-2 |
|  | Text | Exercise | Let's get this party baby bumpin' in here! Dancing is a fun way to exercise and keep your baby happy and healthy. Cute video -> http://youtu.be/R8ppI58-can |
|  | Text | Exercise | Find your inner warrior with this yoga pose! It strengthens and helps with backaches too. http://www.babycentre.co.uk/v1036704/warrior-pose-virabhadrasana |
|  | Facebook | Exercise | Here is a quick and easy workout that is great for your first and second trimester! Here is the best part...You will be done in less than 30 minutes! http://www.youtube.com/watch?v=XBRK_sUhUz4 |
|  | Text | Exercise | Work those arms ladies so they will be ready to carry your little bundle of joy! BONUS: Your arms will look fit and fab! http://www.youtube.com/watch?v=PiSp9fnekZc |
|  | Facebook | Exercise | Here is a great yoga workout for you to check out! http://www.youtube.com/watch?v=44fYnoSLL3c&feature=c4-overview-vl&list=PL2487EBF8CDB2B4C7 |
|  | Facebook | Vitamins/Minerals | Learn how easy it is to get all the nutrients you and baby need and avoid foods that might be harmful. Learn it all here: http://www.youtube.com/watch?v=i7ikrxvC1q0 |
|  | Text | Relax | Get your baby zen on with some relaxing tunes…Free on YouTube: http://www.youtube.com/watch?v=QvsW-BmFAE8 |
|  | Facebook | Exercise | Check out this 10-minute body-pumping workout! It is easy to do at home and if you do not have weights, you can just use two water bottles. Source: https://www.youtube.com/watch?v=A5P1a3u72jg |
|  | Facebook | Exercise | Yoga is a great way to work out when you are pregnant because it nurtures not only your baby, but also your body. Here is a 10-minute yoga video to do at home. Source: https://www.youtube.com/watch?v=B87FpWtkIKA |
